# Supplementary material for: Comparing structural and transcriptional drug networks reveals signatures of drug activity and toxicity in transcriptional responses
Source: NPJ Syst Biol Appl. 2017 Aug 25;3:23. doi: 10.1038/s41540-017-0022-3 (PMC5572457; doi:10.1038/s41540-017-0022-3)
Supplement: Supplementary file 1 — Supplementary Methods [file 41540_2017_22_MOESM1_ESM.pdf]

Supplementary Methods

Comparing structural and transcriptional drug networks reveals signatures of drug activity and toxicity in transcriptional responses.

Francesco Sirci[1], Francesco Napolitano[1,+], Sandra Pisonero-Vaquero[1,+], Diego Carrella[1], Diego L. Medina* and Diego di Bernardo*[1, 2]

1 Telethon Institute of Genetics and Medicine (TIGEM), Via Campi Flegrei 34, 80078 Pozzuoli (NA), Italy
2 Department of Chemical, Materials and Industrial Production Engineering, University of Naples Federico II, Piazzale Tecchio 80, 80125 Naples, Italy
+ These authors contributed equally to this work.
*co-corresponding authors

Random Forest model for the prediction of lysosomal stress and PLD from 128 VolSurf+ descriptors.

Random Forest is a machine learning method for classification that operates by constructing a multitude of decision trees. Every observation is fed into every decision tree. Trees are created by using bootstrap samples of the training data and random features.

We generated a Random Forest classifier to predict potentially PLD or stress-related drugs, based on a set of 128 physico-chemical descriptors. To train the model, we applied the r*andomForest* R package v4.6-12, which implements the Braiman random forest algorithm[1], using the set of 258 drugs that are neighbours of our PLD signature (distance < 0.8) (**Methods** in the main text) versus the remaining 1,051 (1,309-258) CMAP drugs. To avoid training problems due to class imbalance (i.e. 258 versus 1,051), we used a down-sampling of the majority class of 1,051 compounds. The down-sampling implemented in the r*andomForest* package takes a random sample of size $c \cdot nmin$, where $c = 2$ is the number of classes and $nmin = 258$ is the number of samples in the minority class. The RF model includes 500 trees and 11 variables (approximately the root square of the number of variables) tried at each split. In order to assess the model's performance, we used the out-of-bag error (OOB error), that is an estimation of the prediction performance on observations that are not used at training time. Variable importance (**Supplementary Fig. 16a**) was assessed exploiting OOB data, as implemented in the *randomForest* R package: for each tree, the error rate on the out-of-bag portion of the data is recorded. Then the same is done after permuting each predictor variable. The differences between the two are then averaged over all trees, and normalized by the standard deviation of the differences.

We also provide in **Supplementary Table 11** the complete matrix with the 128 Volsurf+ descriptors calculated for the CMAP drugs.

---

[1] Breiman, L. (2001), Random Forests , Machine Learning 45(1), 5-32.

Related R code

```
## "Comparing structural and transcriptional drug networks reveals
## signatures of drug activity and toxicity in transcriptional
## responses", Sirci et al., 2017.
##
## R script to derive physico-chemical features predicting
## phospholipidosis (PLD) inducing drugs

## Loading physico-chemical descriptors for CMap compounds.
VS_matrix_cMAP<-read.csv("PhysicoChemicalDescriptors_cMAP.csv",
                 sep="\t", header=T, stringsAsFactors = F,
                 row.names=1)

## Random Forest with downsampling
library(randomForest)

## training an RF model (results may vary)
RF_PLD <- randomForest(as.factor(VS_matrix_cMAP[,129]) ~ .,
                VS_matrix_cMAP[,1:128], proximity=T,
                importance=T, strata=VS_matrix_cMAP[,1:129]
                $PLD, sampsize=rep(258,2), verbose=T)

## or loading the published RF model
RF_PLD <- readRDS("RandomForest_PLD.rds")

## Extracting Variable Importance
varimpplot<-varImpPlot(RF_PLD, cex=0.7,n.var = 10)
varimpplot<-varimpplot[order(varimpplot[,1], decreasing = T),]
plot(varimpplot[1:10,1], col="red", pch=19,
   main="Variables importance", xlab="Physico-chemical descriptors",
   ylab="Importance (mean Accuracy)", xaxt="n")
axis(1, at=1:10, labels=c(rownames(varimpplot)[1:10]), las=2)


## Features boxplots

## importing the list of PLD signature neighbours
PLD_neigh <- readLines("PLD_neigh.txt")

## splitting CMap profiles in PLD neighbours VS PLD non-neighbours
PLD_pos <- VS_matrix_cMAP[PLD_neigh,]
PLD_neg <- VS_matrix_cMAP[
  setdiff(rownames(VS_matrix_cMAP), PLD_neigh),
  ]

## Figures
boxplot(as.numeric(PLD_pos$LgD10), as.numeric(PLD_neg$LgD10),
     names=c("PLD+", "PLD-"), main="LgD10")
boxplot(as.numeric(PLD_pos$LgD9), as.numeric(PLD_neg$LgD9),
     names=c("PLD+", "PLD-"), main="LgD9")
boxplot(as.numeric(PLD_pos$SOLY), as.numeric(PLD_neg$SOLY),
     names=c("PLD+", "PLD-"), main="SOLY")
boxplot(as.numeric(PLD_pos$LOGP.n.Oct),
```

```
    as.numeric(PLD_neg$LOGP.n.Oct), names=c("PLD+", "PLD-"),
    main="LOGP.n.Oct.")
boxplot(as.numeric(PLD_pos$LgS11), as.numeric(PLD_neg$LgS11),
    names=c("PLD+", "PLD-"), main="LgS11")
boxplot(as.numeric(PLD_pos$PB), as.numeric(PLD_neg$PB),
    names=c("PLD+", "PLD-"), main="PB")
boxplot(as.numeric(PLD_pos$D1), as.numeric(PLD_neg$D1),
    names=c("PLD+", "PLD-"), main="D1")
boxplot(as.numeric(PLD_pos$LgS9), as.numeric(PLD_neg$LgS9),
    names=c("PLD+", "PLD-"), main="LgS9")
boxplot(as.numeric(PLD_pos$LgD8), as.numeric(PLD_neg$LgD8),
    names=c("PLD+", "PLD-"), main="LgD8")
boxplot(as.numeric(PLD_pos$LgS5), as.numeric(PLD_neg$LgS5),
    names=c("PLD+", "PLD-"), main="LgS5")


boxplot(as.numeric(PLD_pos$LOGP.n.Oct),
    as.numeric(PLD_neg$LOGP.n.Oct), as.numeric(PLD_pos$LgD8),
    as.numeric(PLD_neg$LgD8),as.numeric(PLD_pos$LgD10),
    as.numeric(PLD_neg$LgD10), main="Hydrophobic descr.",
    names=rep(c("PLD+", "PLD-"), 3),
    col=rep(c("red", "blue"), 3))
text(x=1.5, y=6, labels = "LgP")
text(x=3.5, y=6, labels = "LgD8")
text(x=5.5, y=6, labels = "LgD10")

boxplot(as.numeric(PLD_pos$CD2),as.numeric(PLD_neg$CD2),
    main="Hydrophobic descr.", names=c("PLD+", "PLD-"),
    col=c("red", "blue"))
text(x=1.5, y=0.35, labels = "CD2")

boxplot(as.numeric(PLD_pos$D5), as.numeric(PLD_neg$D5),
    main="Hydrophobic descr.", names=c("PLD+", "PLD-"),
    col=c("red", "blue"))
text(x=1.5, y=35, labels = "D5")

boxplot(as.numeric(PLD_pos$CD5), as.numeric(PLD_neg$CD5),
    main="Hydrophobic descr.", names=c("PLD+", "PLD-"),
    col=c("red", "blue"))
text(x=1.5, y=0.07, labels = "CD5")

boxplot(as.numeric(PLD_pos$LgS9), as.numeric(PLD_neg$LgS9),
    as.numeric(PLD_pos$LgS8), as.numeric(PLD_neg$LgS8),
    as.numeric(PLD_pos$LgS7.5), as.numeric(PLD_neg$LgS7.5),
    as.numeric(PLD_pos$LgS4), as.numeric(PLD_neg$LgS4),
    main="Hydrophilic descr.",
    names=rep(c("PLD+", "PLD-"), 4),
    col=rep(c("red", "blue"), 4),
    ylim=c(-12,10))
text(x=1.5, y=6, labels = "LgS9")
text(x=3.5, y=6, labels = "LgS8")
text(x=5.5, y=6, labels = "LgS7.5")
text(x=7.5, y=6, labels = "LgS4")
```
