# Supplementary material for: Comparing structural and transcriptional drug networks reveals signatures of drug activity and toxicity in transcriptional responses
Source: NPJ Syst Biol Appl. 2017 Aug 25;3:23. doi: 10.1038/s41540-017-0022-3 (PMC5572457; doi:10.1038/s41540-017-0022-3)
Supplement: Supplementary file 2 — Supplementary Figures [file 41540_2017_22_MOESM2_ESM.pdf]

Supplementary Figures

Comparing structural and transcriptional drug networks reveals signatures of drug activity and toxicity in transcriptional responses.

Francesco Sirci[1], Francesco Napolitano[1,+], Sandra Pisonero-Vaquero[1,+], Diego Carrella[1], Diego L. Medina* and Diego di Bernardo*[1, 2]

1 Telethon Institute of Genetics and Medicine (TIGEM), Via Campi Flegrei 34, 80078 Pozzuoli (NA), Italy
2 Department of Chemical, Materials and Industrial Production Engineering, University of Naples Federico II, Piazzale Tecchio 80, 80125 Naples, Italy
+ These authors contributed equally to this work.
*co-corresponding authors

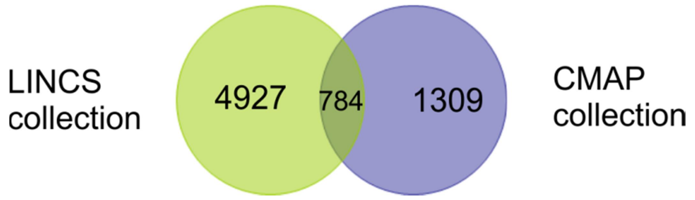

Supplementary Figure 1. Collection of compounds used in this manuscript. The compounds selected for this study were present in the LINCS collection (Broad Institute) and in the CMAP collection (Broad Institute). The union of these two sets resulted in 5452 compounds.

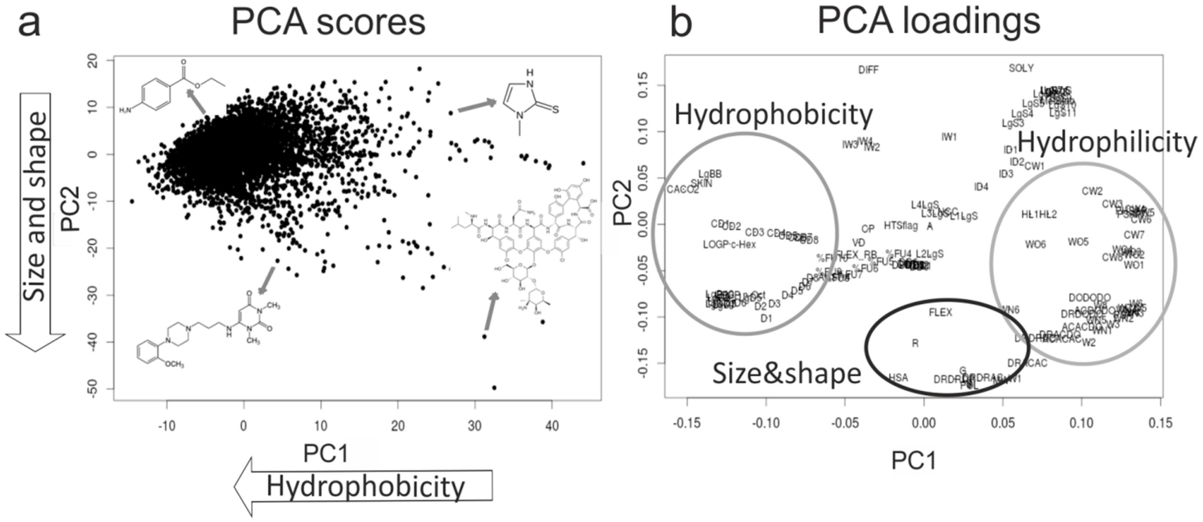

Supplementary Fig. 2 Principal Component Analysis of 5452 small molecules described by 128 physico-chemical features. (a) Distribution of the small molecules in the plane formed by the first two principal components (PCs) in the physic-chemical feature space. Small and hydrophobic compounds are found towards the upper left corner, while large and hydrophilic ones towards in the lower right corner. **(b)** The loadings of the 128 physico-chemical features on the first two PCs. PC1 explains 36% of the total variance and it is related to those features involved in the definition of hydrophobic and aromatic properties of the drug set. The PC2 additionally explained 17% of the variance and is related to size and shape.

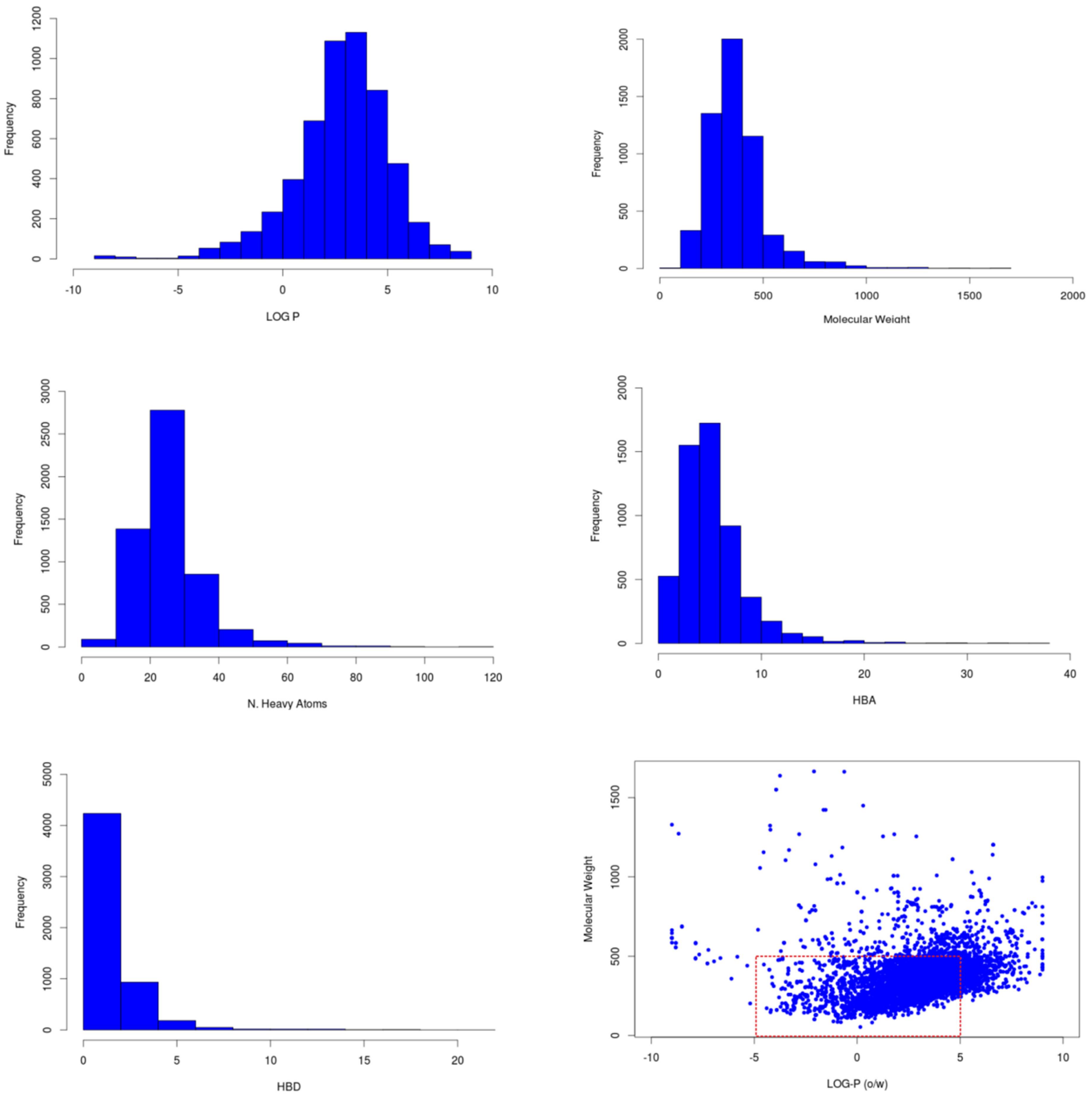

Supplementary Fig. 3. Overview of representative physico-chemical features of the 5452 compounds. Each histogram shows the values of the indicated feature across the compounds. LOG-P: (coefficient partition octanol/water), HBA: (H-bond acceptor), HBD: (H-bond donor). Dashed red line represents the limits of the Lipinski ‘Rule of Fives’ (RoFs).

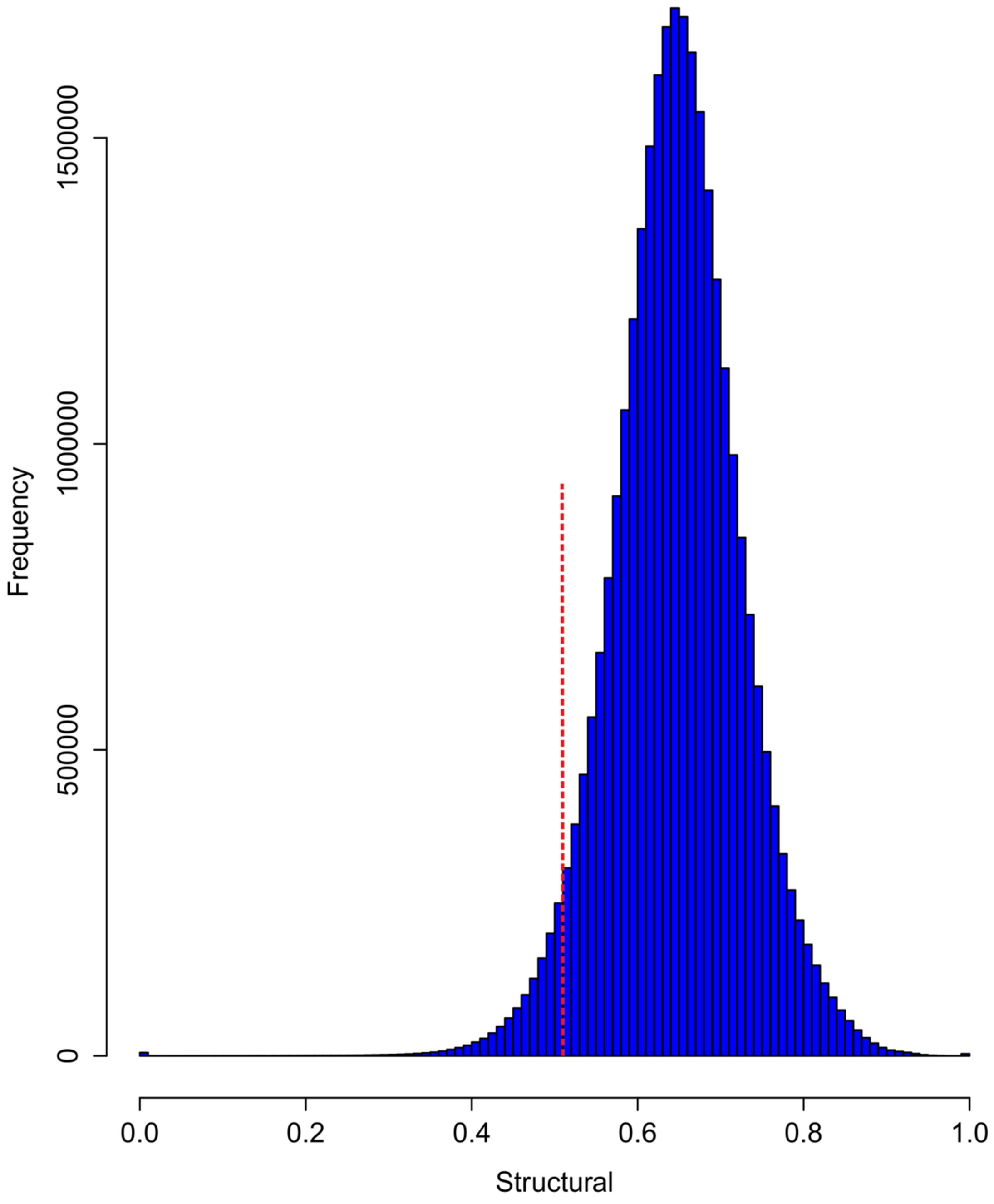

Supplementary Fig. 4 Distance distribution of the 14,859,416 edges of the LINCS and CMAP drug network (5452x5452 matrix). The structural distance threshold of 0.5 (vertical dashed line) was defined by sorting drug-pairs in ascending order according to their structural distance and then selecting as the distance significance threshold value the upper bound of the 5% quantile of this empirical distribution.

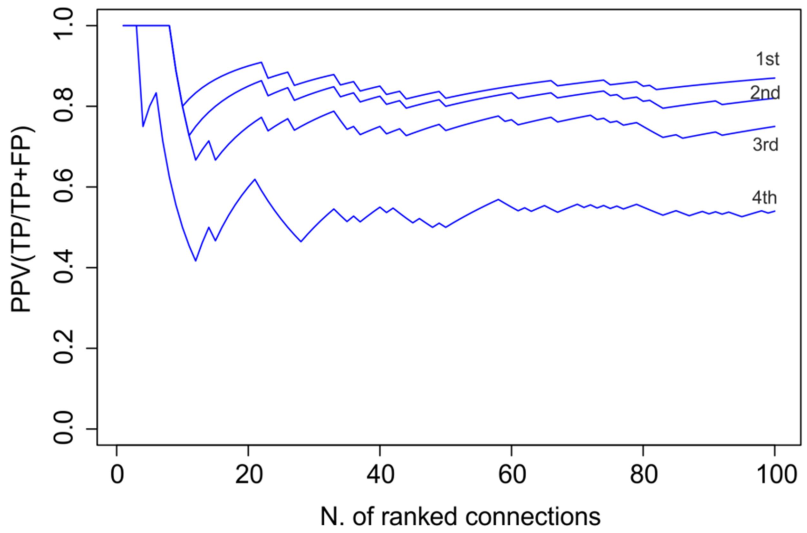

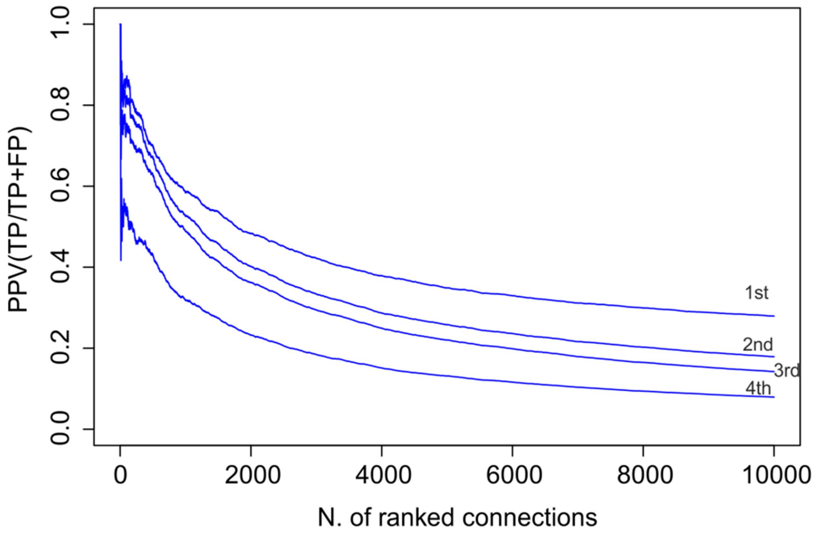

Supplementary Figure 5: Performance of the structural distance in detecting drugs with the same ATC code. The structural distance for each drug-pair across 5452 compounds was computed as reported in the Methods. Drug-pairs were then sorted according to their structural distance, with drug-pairs with the smallest distance towards the origin of the *x-axis*; the Positive Predictive Value (PPV) was computed as the percentage of True Positives over False Positives plus True Positives and shown on the *y-axis*. The PPV for the first 100 most similar drug-pairs (upper panel) and for the first 10,000 (lower panel) are shown for the first four ATC ontology level annotations.

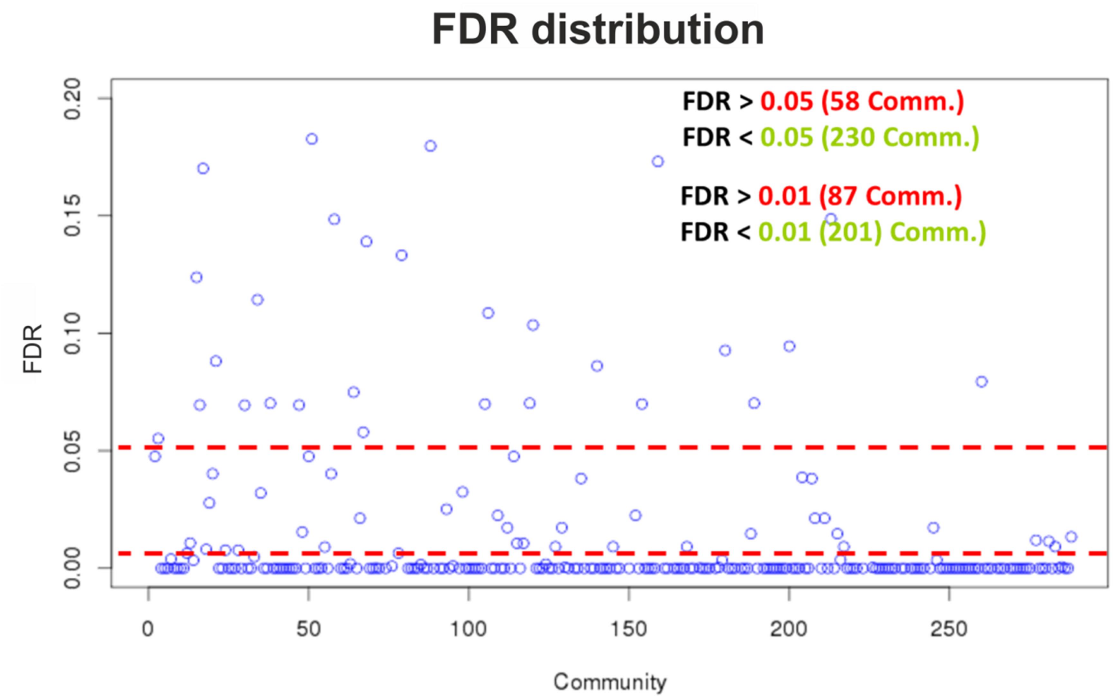

Supplementary Figure 6: Validation of the structural drug network communities. The 288 communities were tested for enrichment of compounds with the same ATC code. The FDR were computed using the hypergeometric distribution with the Bonferroni correction. 230 out of 288 (80%) structural communities were significantly enriched for compounds sharing the same ATC code (FDR <0.05).

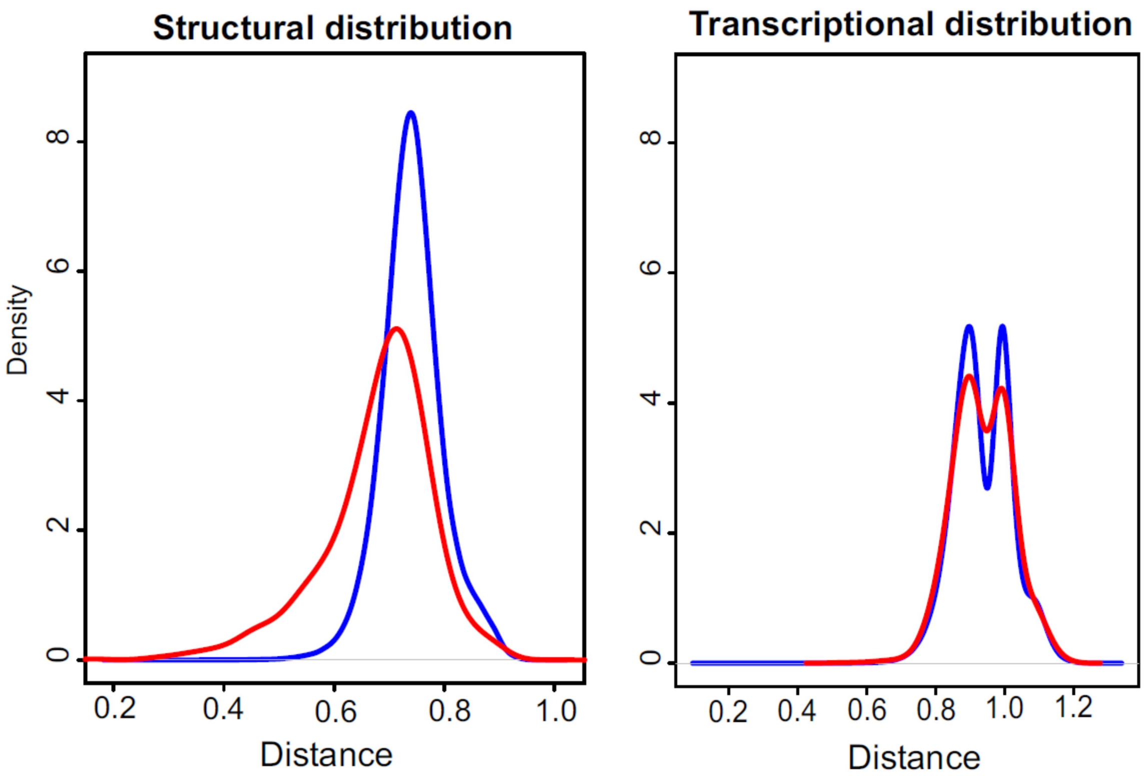

Supplementary Figure 7: Distribution of the structural and transcriptional distance among drug-pairs in Figure 2. The red lines show the distance distribution of drug-pairs sharing same ATC code (shown as red points in Figure 2), while the blue lines refer to drug-pairs with differing ATC codes (shown as blue points in Figure 2).

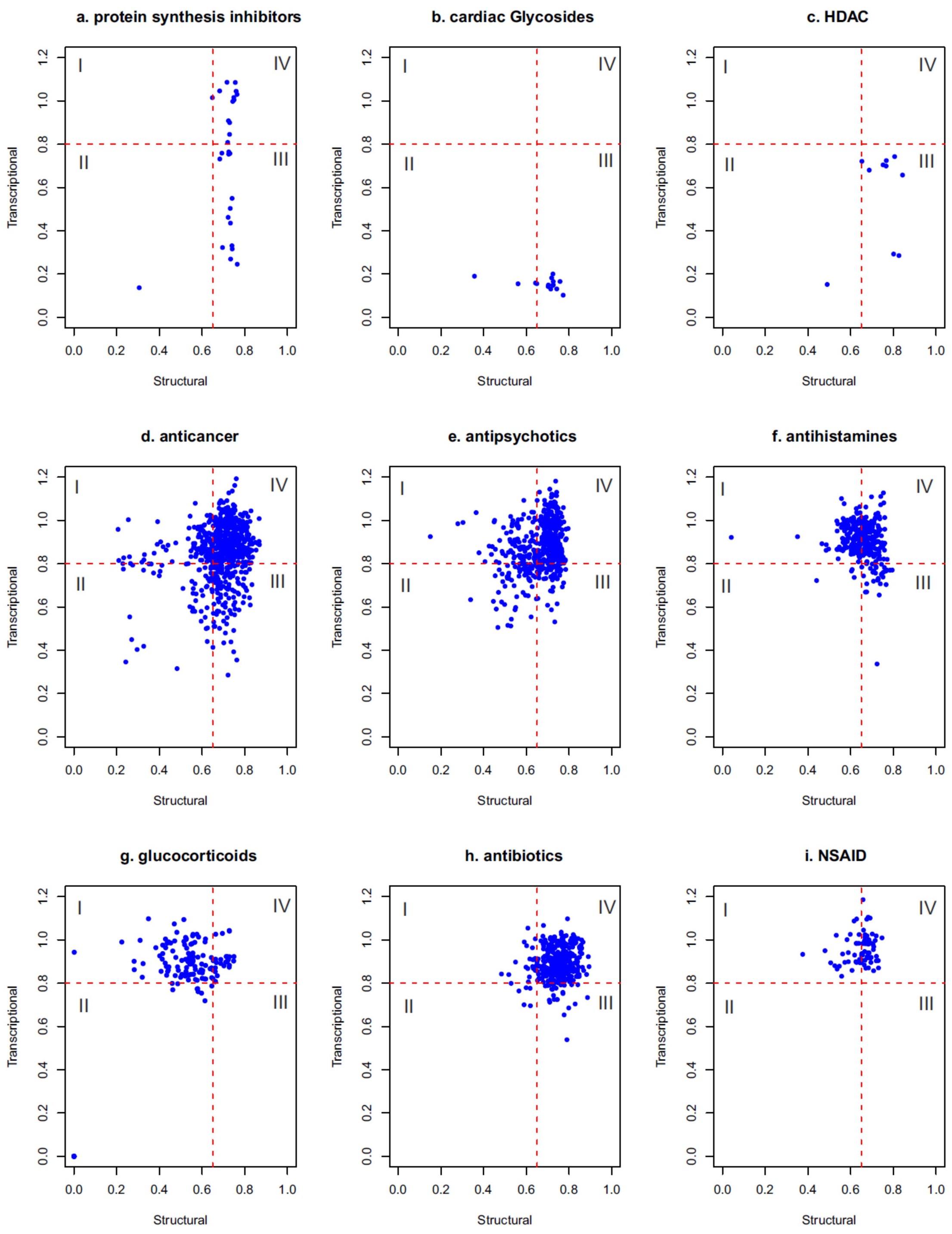

Supplementary Figure 8: **Comparison of transcriptional and structural distances between CMAP compounds belonging to different drug classes.** As in Figure 2 of the main manuscript, each dot represents the structural (x-axis) and transcriptional (y-axis) distance between two compounds. Dashed lines represent the significance threshold for the transcriptional (horizontal line) and structural (vertical line) distance, splitting the plane into four quadrants.

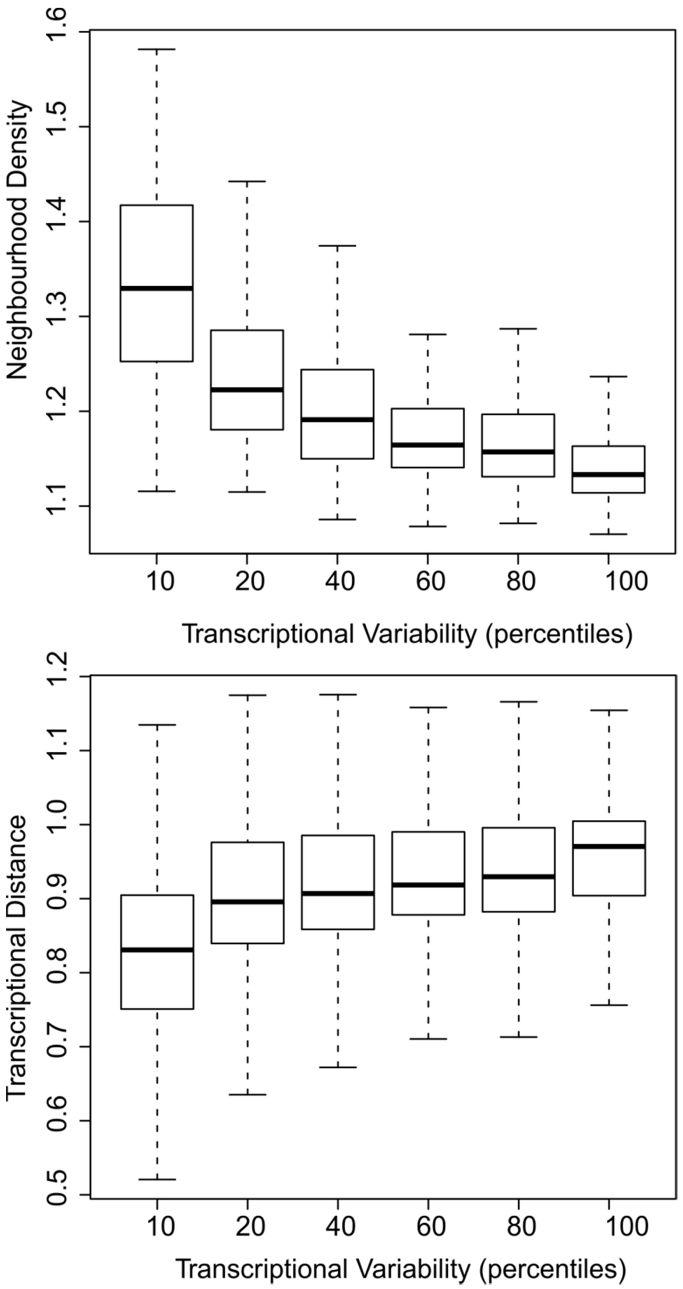

Supplementary Figure 9: Relationship between the Transcriptional Variability (TV) of a compound and its transcriptional distance to other compounds in CMAP. For each of the 1165 compounds in CMAP for which the TV could be estimated (i.e. at least two experiments in the same cell lines), we report the number of compounds inducing a similar transcriptional response (i.e. with a transcriptional distance <0.8) as a function of the TV of the compounds (UPPER PANEL), and the transcriptional distance of the compound to all of the other CMAP compounds as a function of the TV of the compounds (LOWER PANEL).

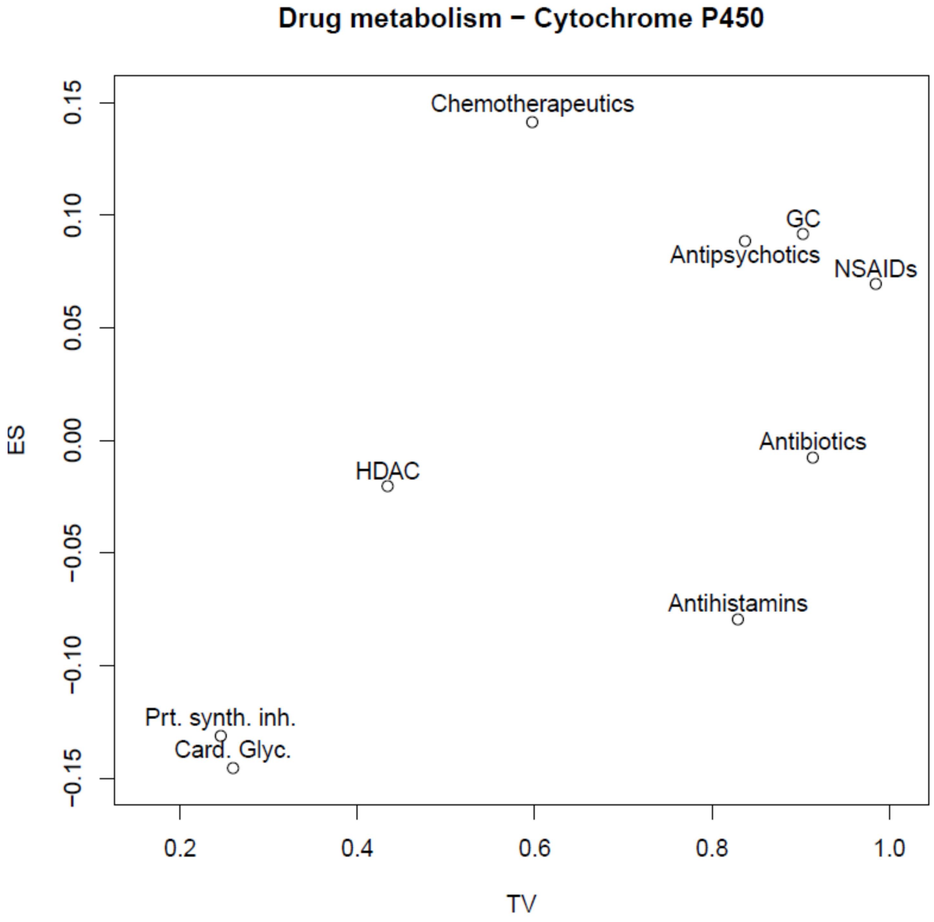

Supplementary Figure 10. Relation between Transcriptional Variability and expression of Cytochrome P450-mediated drug metabolism across drug classes. Each dot represents one of the drug classes shown in **Figure 3**. The KEGG pathway (hsa00982:Drug metabolism - cytochrome P450) was selected for this analysis. By means of Gene Set Enrichment Analysis, the Enrichment Score (ES) for this pathway was computed for each drug using the corresponding Prototype Ranked List of differentially expressed genes from CMAP. A positive ES implies that genes in the pathway tend to be upregulated by the drug and vice-versa. Each dot represents a drug class, with x-coordinate corresponding to the median of the Transcriptional Variability (TV) of drugs in the drug class, and y-coordinate the median Enrichment Score (ES) of the hsa00982 pathway for the drugs in the drug class.

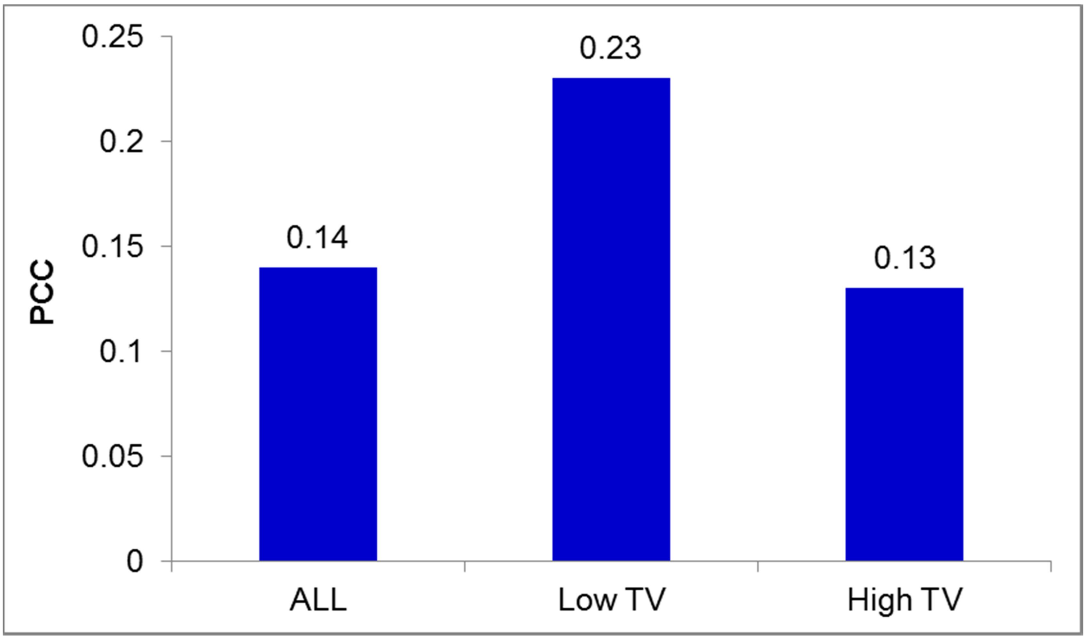

Supplementary Figure 11. The correlation between transcriptional and structural distances for the compounds in CMAP as a function of the Transcriptional Variability (TV). Compounds were divided into three sets: (All) the 1165 compounds in CMAP having at TV value; (Low TV) 582 compounds with a TV lower than the median TV among all the compounds; (High TV) 582 compounds with a TV higher than the median TV among all the compounds. The Pearson correlation coefficient (PCC) between structural and transcriptional distance for each of the three sets was then computed.

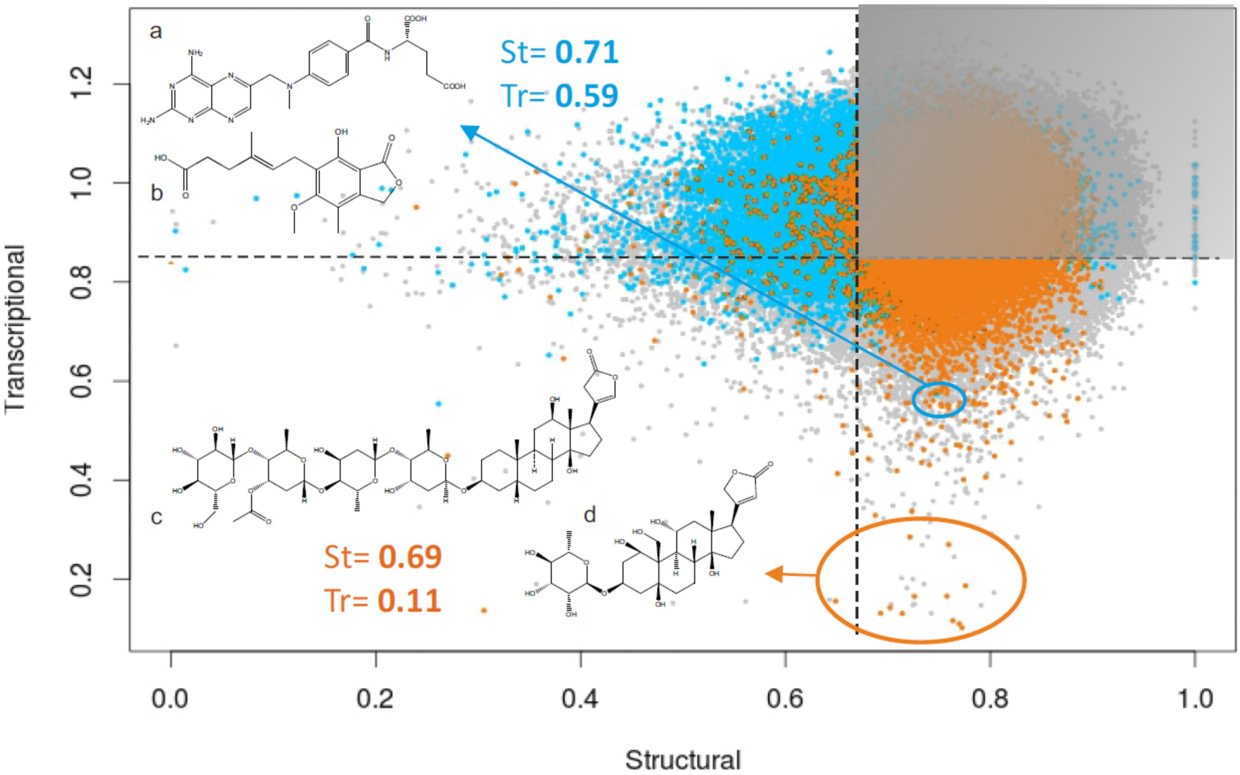

Supplementary Figure 12: Drug shape and size distribution in the transcriptional-structural space. Structurally similar drug-pairs (left-side of the plot) are enriched for drug-pairs consisting of two small-size drugs (MW less than 500Da; in blue), whereas transcriptionally similar drug-pairs tend to be made-up by at least one large-size drug pair (MW more than 500Da; in orange). **a)** methotrexate; **b)** mycophenolic acid; **c)** lanatoside C; **d)** digoxin

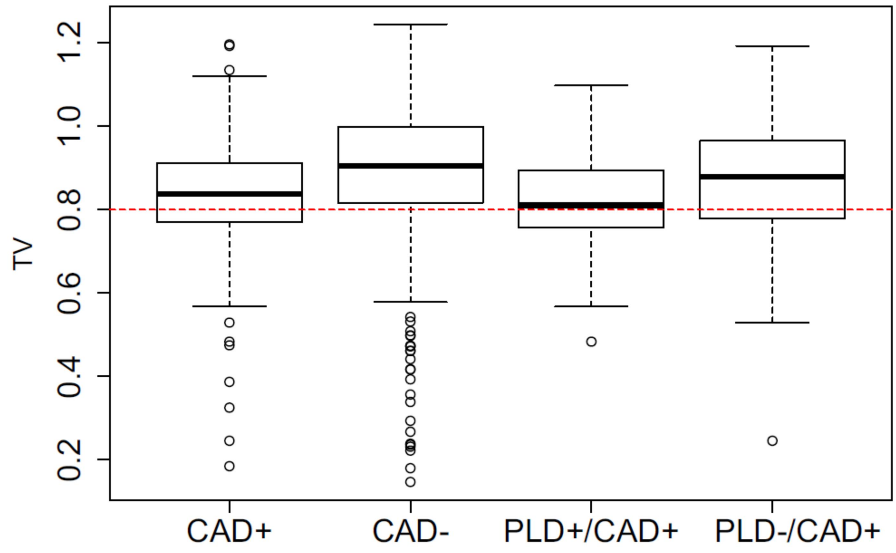

Supplementary Figure 13: Transcriptional responses among CAD+, CAD-, PLD+/CAD+ and PLD-/CAD+ compounds. Compounds were dived into four sets: (CAD+) compounds that are cationic amphiphilic; (CAD-) compounds that are not cationic amphiphilic; (PLD+/CAD+) compounds that are CAD+ and also induce phospholipidosis; (PLD-/CAD+) compounds that are CAD+ but do not induce phospholipidosis. The box in the plot for each set represents the median TV, the bottom and top of the box are the first and third quartiles of the TV, and the whiskers represent the lower and the upper 1.5 IQR.

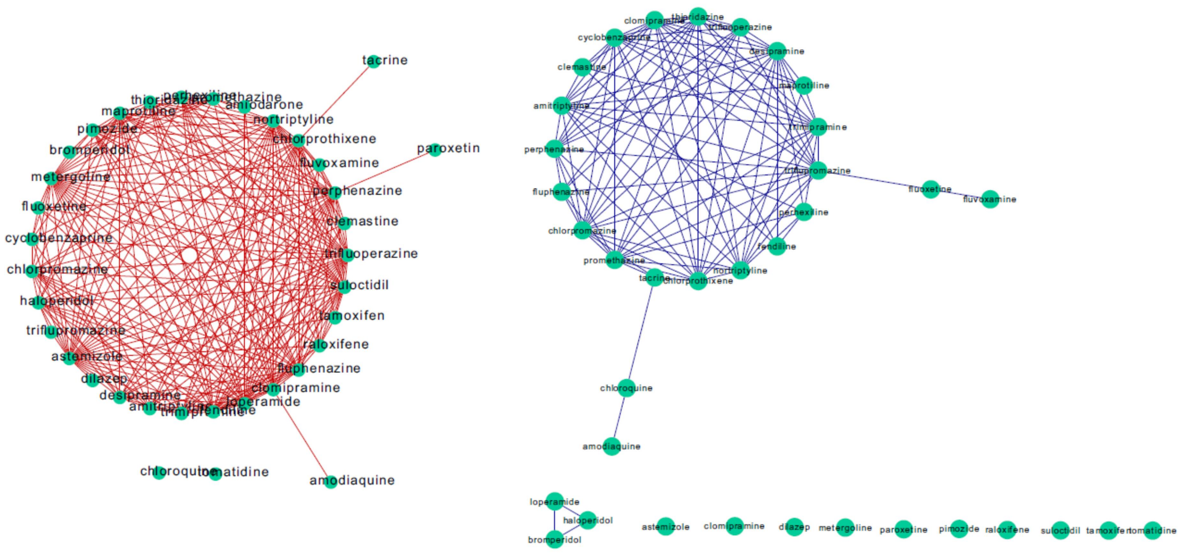

Supplementary Figure 14: Transcriptional and structural similarity networks among the 35 PLD/CAD compounds. The transcriptional sub-network (left) and the structural sub-network (right) showing that these drugs tend to be transcriptionally similar but less so structurally.

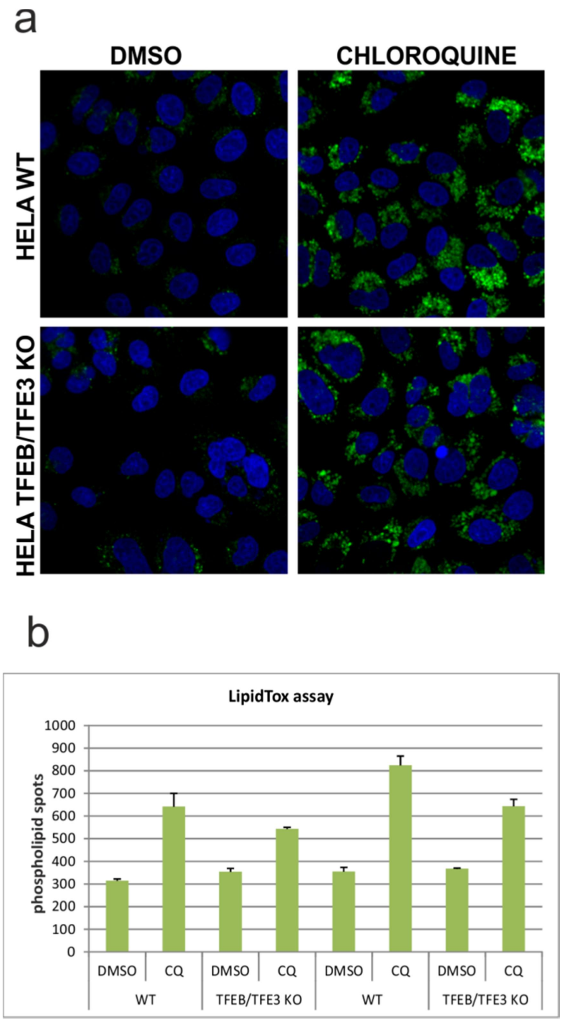

Supplementary Figure 15: Phospholipids accumulation in HeLa wt and HeLa TFEB/TFE3 KO cells. **a)** Cells were seeded, incubated for 24h and treated with DMSO 0.1 % (negative control) and chloroquine 50 uM (positive control) dissolved in culture medium with the LipidTOX reagent for 48h. Phospholipid accumulation was detected by the LipidTOX fluorescence (green). Nuclei were stained with DAPI (blue). Images are representative of three independent experiments; **b)** Quantification of the phospholipid spots intensity for two replicates.

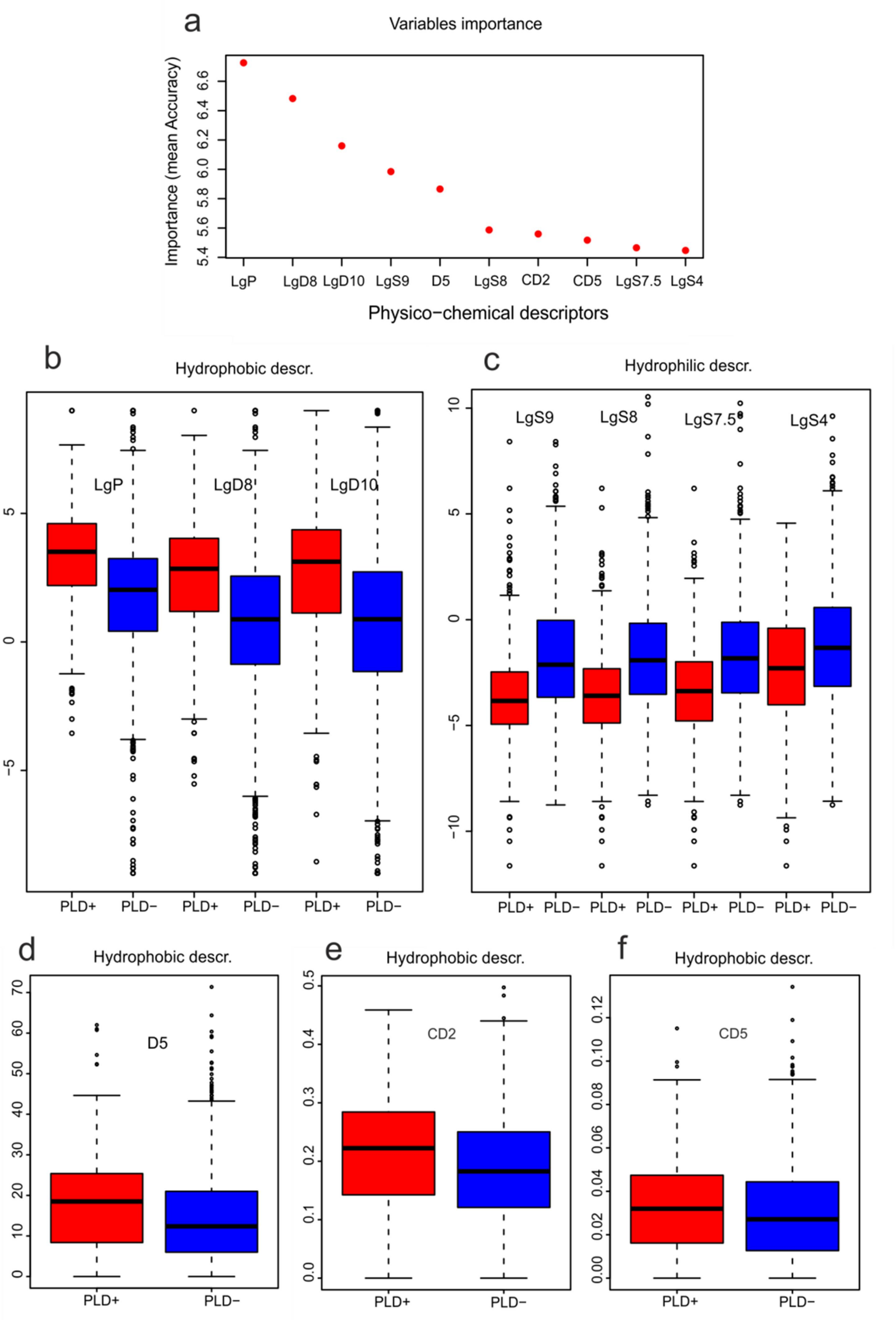

Supplementary Figure 16. Random Forest model using as features the VolSurf+ physico-chemical parameters. PLD+ refers to the 258 compounds in CMAP whose transcriptional distance to the PLD signature is less than the significance threshold (0.8). **PLD-** refers to the remaining 1051(=1309-258) CMAP compounds. **(a)** Importance plot of the first 10 VolSurf+ parameters according to the Random Forest model; **(b-f)** Comparison of the balues of the 10 VolSurf+ parameters in the PLD+ compounds versus the PDL- compounds. **LgP**: LogP octanol/water (LOGP N-oCT); **LgDx**: LogD at a pH of x; **LgSx**: Solublity at a pH of x; **D5**: the volume of hydrophobic interactions at -1kcal/mol; **CD2**: Capacity Descriptor, i.e. the concentration of hydrophobic interactions on the molecular at -0.4 kcal/mol energy level; **CD5**: the same as CD2 but at an energy lelel of -1kcal/mol.

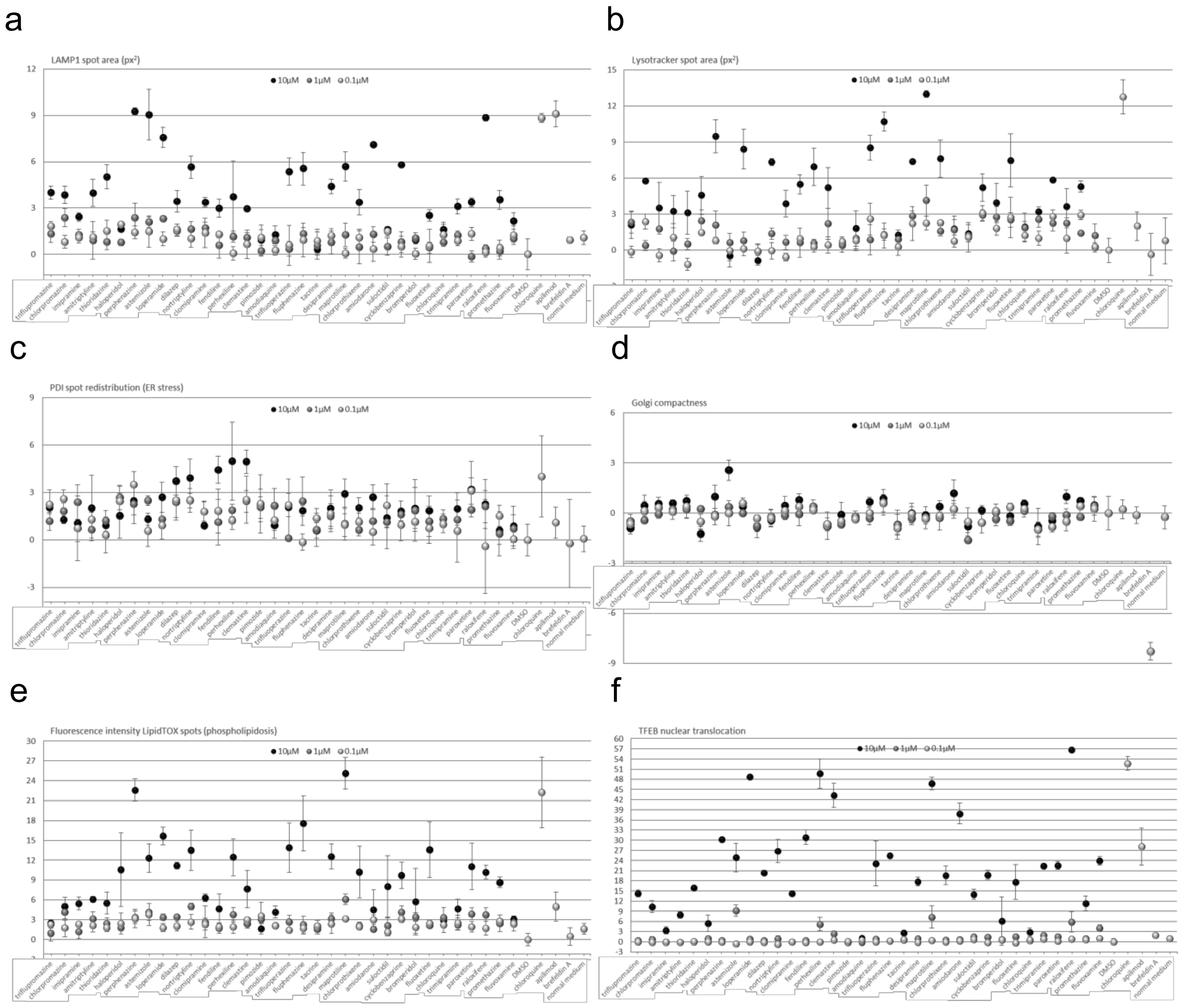

Supplementary Figure 17. Effect of CADs on lysosome area, ER stress, Golgi morphology, lysosomal phospholipid accumulation and TFEB activation. Lysosome area was analyzed by immunodetection of the lysosomal protein LAMP1 or Lysotracker staining. ER stress was determined by the redistribution of PDI in puncta structures. Changes in the compactness of Gogi were analyzed by the immunodetection of the Golgi matrix protein GM130. Phospholipidosis induction was analyzed by the intralysosomal accumulation of the LipidTOX green reagent. TFEB activation was determined by its nuclear localization. These parametres were analyzed by using an automated confocal microscopy (Opera High Content System, Perkin-Elmer) in combination with a dedicated script developed through the Columbus Image Data Management and Analysis Software (Perkin-Elmer). Chloroquine 50μM 24h and apilimod 100nM 1h were used as positive controls of lysosomotropism; brefeldin A 10μg/ml was used as positive control of total loss of Golgi compactness; DMSO 0.1% and normal medium were used as negative controls. Fold increase vs DMSO data are normalized by DMSO SD. Values upon 3 folds DMSO SD are considered positive. Data are represented as mean ± SD of three independent experiments.

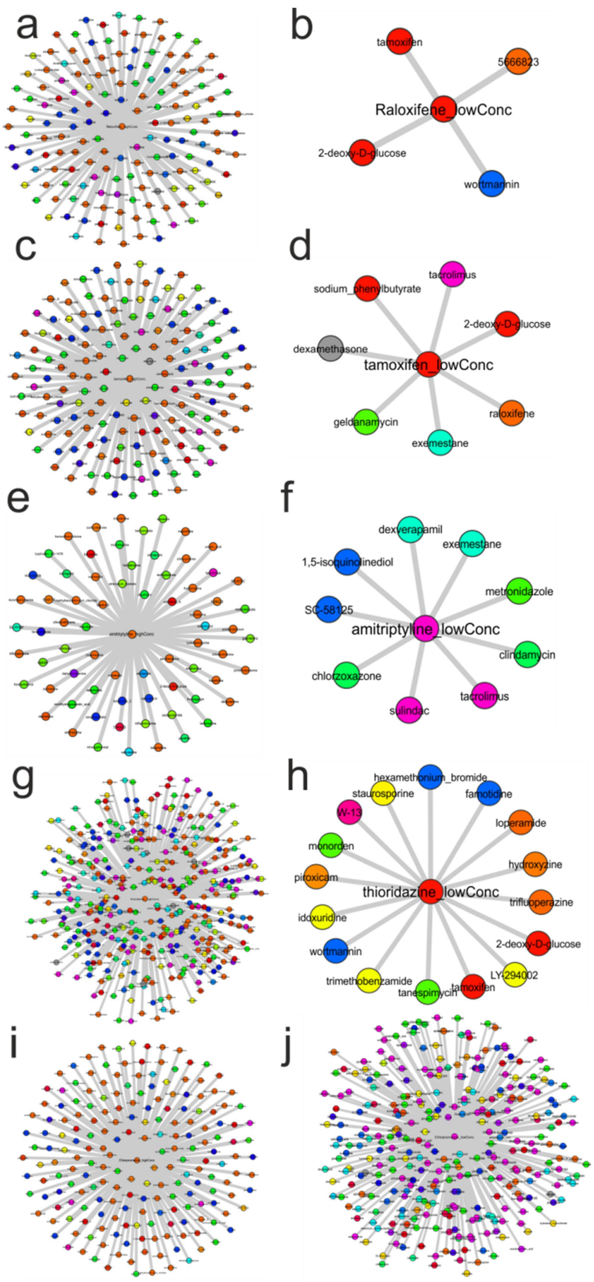

Supplementary Figure 18: Relationship between drug concentration and transcriptional distance. Each network represents the drug neighbours of four drugs for which treatments at low and high concentration were available in CMAP. **a), b)** drug neighbours of raloxifene (ER antagonist at 0.1 μM and 7.8 μM); **c), d)** tamoxifen (ER antagonist at 1 μM and 7.0 μM); **e), f)** amitriptyline (antidepressant 1 μM and 12.8 μM); **g), h)** thioridazine (antipsychotic at 1 μM and 10 μM); **i), j)** chlorpromazine (antipsychotic at 1 μM and 11.2 μM)
