# Supplementary material for: Comparing structural and transcriptional drug networks reveals signatures of drug activity and toxicity in transcriptional responses
Source: NPJ Syst Biol Appl. 2017 Aug 25;3:23. doi: 10.1038/s41540-017-0022-3 (PMC5572457; doi:10.1038/s41540-017-0022-3)
Supplement: Supplementary file 3 — Supplementary Table 1 [file 41540_2017_22_MOESM3_ESM.pdf]

	RC1	RC2	RC3	RC4	RC5	RC6	RC7	RC8
DIFF	0.990106	0.614086	0.509933	0.497169	0.156891	0.644239	0.763613	-0.99853
G	-0.98809	-0.49125	-0.45516	-0.32215	-0.23261	-0.54692	-0.77318	0.999267
W1	-0.97545	-0.62053	-0.30076	-0.28678	-0.46648	-0.63172	-0.76692	0.999084
CW1	0.666667	-0.25791	0.641394	0.392305	-0.64239	0.362857	0.28951	0.603663
CD5	-0.92213	-0.3255	-0.21063	0.362149	0.193275	-0.49294	-0.10969	-0.50952
W2	-0.71015	-0.62712	0.355955	0.163469	-0.65534	-0.28712	-0.52687	0.999084
CD4	-0.94137	0.338692	-0.26915	0.297567	0.226171	-0.46618	-0.08752	-0.56227
CW2	0.665934	-0.40478	0.662207	0.407921	-0.75045	0.397567	0.200126	0.850916
CW3	0.664651	-0.54009	0.65966	0.42513	-0.74129	0.388674	0.142971	0.924176
WN1	-0.56559	-0.8199	0.412871	0.273829	-0.67449	-0.198	-0.46189	0.999267
W3	-0.55148	-0.65723	0.441601	0.29511	-0.71054	-0.16719	-0.43388	0.999084
L2LgS	-0.6183	0.526882	0.290741	0.264869	-0.25411	-0.10224	-0.12907	0.924725
CD6	-0.92983	-0.2618	-0.15618	0.366434	0.184655	-0.50108	-0.12555	0.525458
PSAR	0.60932	-0.43121	0.680875	0.347416	-0.73314	0.411217	0.173656	0.979304
PHSAR	0.60932	-0.43121	0.680875	0.347416	-0.73314	0.411217	0.173656	0.979304
CP	-0.98809	0.67429	-0.54228	0.115976	0.409745	-0.44677	-0.08212	-0.74121
WO5	-0.33333	0.319612	0.623223	-0.1166	-0.46915	0.359077	0.15098	0.997802
WO6	-0.33333	0.319612	0.622482	-0.1166	-0.46915	0.358021	0.15098	0.998352
CD7	-0.96207	0.30566	0.136153	0.42303	0.206725	-0.51866	-0.14271	-0.50495
D4	-0.97508	-0.4553	-0.42249	0.146539	0.156198	-0.65527	-0.38351	0.989744
PSA	-0.45053	-0.55974	0.474249	0.2819	-0.73017	0.127697	-0.33575	0.998718
D5	-0.96702	-0.42757	-0.37189	0.202722	0.144679	-0.64714	-0.36939	0.990476
WN2	-0.38531	-0.83954	0.464077	0.277338	-0.70415	-0.11334	-0.38629	0.999634
D6	-0.96152	-0.40022	-0.2787	0.25497	0.153856	-0.63628	-0.35365	0.988095
ACACDO	-0.65647	0.249089	0.373643	-0.21569	-0.67774	-0.2692	-0.36988	0.997802
W4	-0.35288	-0.70938	0.482751	0.273709	-0.74154	0.103447	-0.3644	0.999451
DRDODO	-0.45731	0.450707	0.427862	-0.25222	-0.57859	0.261491	-0.25068	0.996154
DODODO	-0.41242	0.452687	0.438984	-0.21271	-0.65429	0.287373	-0.24054	0.998718
ACDODO	-0.41059	0.47178	0.43954	-0.21271	-0.65429	0.26963	-0.24054	0.998168
CW4	0.661903	-0.61059	0.635844	0.402554	-0.73677	0.393082	0.116913	0.964103
D7	-0.96665	-0.35194	-0.2318	0.298564	0.189199	-0.63844	-0.36937	0.964652
AUS7.4	-0.56736	-0.57555	-0.25881	0.357136	-0.37845	-0.34016	-0.17306	-0.87473
CD3	-0.9782	0.446471	-0.375	0.12358	0.281207	-0.47044	-0.07034	-0.67894
ACACAC	-0.69787	-0.21493	0.301809	0.345251	-0.65905	-0.2895	-0.46619	0.997985
%FU7	-0.55289	-0.58821	0.254343	0.371421	-0.39584	-0.35213	-0.15603	-0.91245
WN4	0.302247	-0.71085	0.530938	0.394958	-0.66041	0.093552	-0.29702	0.997619
%FU10	-0.54849	-0.63595	0.155368	0.209812	0.245198	-0.29488	-0.12944	-0.91245
%FU8	-0.55435	-0.54507	0.240866	0.29422	-0.3551	-0.31715	-0.16627	-0.91245
W6	0.345487	-0.65118	0.509018	0.344995	-0.68696	0.106944	-0.25535	0.997436
W5	0.350983	-0.74243	0.506429	0.286776	-0.72693	0.096713	-0.32786	0.998901
CW5	0.753939	-0.60878	0.631533	0.408333	-0.71608	0.406161	0.106911	0.965751
%FU9	-0.55344	-0.54048	0.205637	0.234346	-0.27798	-0.31451	-0.16069	-0.91245
MetStab	0.896482	0.381114	0.655756	0.40407	-0.29072	0.660269	0.616726	0.745421
WO4	-0.33333	0.352291	0.608316	-0.1785	-0.46915	0.420153	0.186194	0.995238
WN3	-0.33529	-0.80264	0.516061	0.318604	-0.70758	-0.07562	-0.34264	0.999084
%FU6	-0.49756	-0.64127	0.267265	0.422601	-0.46655	-0.37635	-0.17776	-0.91245
DRACAC	-0.95181	0.38295	-0.39023	-0.2813	-0.53003	-0.54912	-0.63975	0.995421
DRACDO	-0.78399	0.532574	0.278555	-0.3047	-0.57783	-0.43464	-0.49911	0.995604

CW6	0.799377	-0.58949	0.641823	0.361665	-0.68286	0.344275	0.106196	0.946337
D3	-0.98901	-0.35524	-0.53527	-0.23217	0.225834	-0.68605	-0.43855	0.978388
CD8	-0.96207	0.326589	0.16572	0.450673	0.261817	-0.55747	-0.1498	-0.55092
HTSflag	0.887871	0.374321	0.647044	0.395373	-0.24419	0.651478	0.607729	0.736813
WO1	-0.33333	0.465945	0.533342	-0.13588	-0.53069	0.344295	-0.08674	0.997802
%FU5	-0.33761	-0.70222	0.227599	0.388367	-0.51313	-0.32401	-0.19726	-0.91245
L3LgS	0.543056	0.836607	-0.19801	-0.1859	0.416067	0.275793	0.203676	0.962821
WO2	-0.33333	0.496787	0.551507	-0.20463	-0.46915	0.387965	0.17734	0.994872
D8	-0.96665	-0.33339	-0.16173	0.347423	0.227187	-0.63508	-0.3346	0.886996
WO3	-0.33333	0.451258	0.571711	-0.19982	-0.46915	0.399932	0.193762	0.993773
VD	-0.81019	0.716174	0.231098	-0.28349	0.571126	-0.15151	-0.08012	-0.99432
LgS3	0.880726	0.677804	0.478316	0.197248	0.39296	0.531412	0.46937	-0.98846
LgS4	0.899414	0.695612	0.474895	-0.21372	0.41636	0.575221	0.494223	-0.98645
LgS5	0.912789	0.675221	0.492875	0.193719	0.405113	0.606272	0.511243	-0.98645
LgD10	-0.76805	0.367358	-0.57235	-0.27177	0.408221	-0.54072	-0.38086	-0.78223
LgD8	-0.78655	0.480815	-0.609	-0.28972	0.252903	-0.55717	-0.4337	-0.8033
LgD7.5	-0.78728	0.424454	-0.62781	-0.2872	0.210161	-0.59396	-0.44207	-0.80568
LgD9	-0.78215	0.4698	-0.58528	-0.28972	0.366429	-0.54146	-0.41071	-0.79689
%FU4	0.395933	-0.70498	-0.22948	0.355542	-0.52404	-0.27661	-0.18345	-0.91245
LgD7	-0.7838	0.369378	-0.64376	-0.27932	0.166101	-0.60004	-0.43759	-0.80348
LgD6	-0.76292	-0.38517	-0.63574	-0.26922	-0.13936	-0.59255	-0.42095	-0.7848
CD2	-0.8952	0.457132	-0.54422	-0.25997	0.536592	-0.50942	-0.06326	-0.84139
LgS6	0.924698	0.59279	0.493664	0.184133	0.352288	0.626661	0.527506	-0.98553
L0LgS	0.933492	0.550014	0.49941	0.199677	0.248863	0.636787	0.540491	-0.9815
NCC	-0.6277	-0.66292	-0.29029	-0.26693	0.206133	-0.36715	-0.26659	0.823626
LgS7	0.930011	0.49145	0.515441	0.205562	0.259725	0.635549	0.543761	-0.98645
WN6	-0.73379	0.496197	0.224543	0.176714	-0.20338	-0.43367	-0.4743	0.995788
L1LgS	0.663796	-0.65082	-0.1688	0.232265	-0.54633	0.176247	0.119644	0.821062
LgBB	-0.37535	0.379659	-0.44855	-0.29818	0.623526	-0.17868	0.169948	-0.99927
LgS7.5	0.929828	0.451612	0.526191	0.219626	0.219938	0.630493	0.548148	-0.98645
FLEX_RB	-0.61323	-0.65356	-0.28746	-0.26874	0.218934	-0.4125	-0.29831	0.851832
LgD5	-0.73543	-0.48981	-0.62383	-0.24819	-0.21509	-0.57329	-0.40123	-0.75916
L4LgS	0.517406	-0.55551	0.221805	0.237952	0.236768	0.267371	0.185698	0.85696
HL1	0.987908	-0.59592	0.55971	0.269674	-0.65059	0.517076	0.107317	0.85989
IW1	0.94705	0.180204	0.160345	0.309949	-0.43255	0.420119	0.547897	-0.67784
D2	-0.98314	0.353038	-0.64399	-0.40949	0.444751	-0.71653	-0.49015	0.884432
PB	-0.97325	0.427942	-0.64181	-0.38233	0.385792	-0.65584	-0.48126	-0.94048
SOLY	0.990473	0.34441	0.707656	0.3895	-0.12545	0.7376	0.714201	-0.98242
LgS8	0.928362	0.4193	0.53861	0.229989	0.1766	0.616292	0.558924	-0.98626
CW7	0.784353	-0.51386	0.587143	0.403947	-0.58537	0.206741	-0.09881	0.960806
LgS11	0.893001	0.622545	0.49431	0.248351	-0.2964	0.580938	0.5089	-0.58645
LgS9	0.923232	0.407184	0.543986	0.239796	-0.19973	0.606929	0.560248	-0.98132
LgS10	0.912422	0.534055	0.527118	0.25897	-0.25943	0.591312	0.543475	-0.90714
LOGP n-Oc	-0.89337	0.40995	-0.71192	-0.3828	0.32792	-0.6061	-0.4985	-0.99304
ID4	-0.96079	-0.55607	0.523184	-0.19118	0.130491	0.352444	0.162855	-0.83278
CW8	-0.23348	-0.22855	0.531536	0.329145	-0.31423	0.173719	-0.26131	0.980037
IW4	0.669659	-0.34419	-0.18039	-0.11549	0.228208	0.25692	0.498154	-0.95879
CACO2	-0.55655	0.302919	-0.53811	-0.23582	0.578004	-0.38736	0.05178	-0.99908
WN5	-0.42451	-0.7125	0.409714	0.421199	-0.48332	-0.2759	-0.37677	0.997985
D1	-0.97563	0.274279	-0.68634	-0.42795	0.48357	-0.68456	-0.49905	0.782784

CD1	-0.75504	0.423706	-0.59683	-0.24525	0.623478	-0.49123	-0.0565	-0.92802
FLEX	-0.80487	-0.69174	-0.33514	-0.34646	-0.17339	-0.33683	-0.45501	1
ID1	0.994503	-0.46337	0.592555	0.238371	-0.28801	0.453841	0.417688	-0.7696
LOGP c-He	-0.56791	-0.24615	-0.67568	-0.35612	0.529434	-0.43638	-0.21775	-0.99505
DRDRDO	-0.79754	0.639421	-0.27192	-0.30336	-0.48541	-0.47664	-0.55819	0.994139
SKIN	-0.50507	0.214168	-0.62971	-0.26508	0.645862	-0.40264	-0.06247	-0.99835
W8	-0.45749	-0.3099	0.447802	0.302015	-0.33844	-0.10847	-0.31222	0.996337
W7	-0.28564	-0.62934	0.469405	0.397441	-0.5909	-0.16219	-0.28108	0.996337
DD8	-0.80432	-0.47466	-0.07031	-0.222	0.183221	-0.37285	-0.39972	0.898535
IW3	0.635581	0.223557	-0.35409	-0.13088	0.289543	0.176506	0.487256	-0.95714
DD5	-0.88604	-0.47448	-0.12015	-0.22003	0.128461	-0.24504	-0.38264	0.988278
ID3	-0.66319	-0.51974	0.620777	-0.13569	0.132737	0.437237	0.208656	-0.81227
ID2	-0.66319	-0.64363	0.669382	-0.05613	-0.12599	0.428992	0.309259	-0.84158
HL2	0.966838	-0.5671	0.509849	0.264086	-0.56136	0.487531	0.122437	0.81044
DD6	-0.68139	-0.47448	-0.13859	-0.21307	0.210205	-0.29814	-0.39464	0.961905
DD7	-0.77977	-0.47448	-0.10532	-0.2281	0.177524	-0.3274	-0.42261	0.98663
DD4	-0.86387	-0.47448	-0.14785	-0.19645	0.18808	-0.26349	-0.38968	0.877839
IW2	0.698974	0.277335	-0.32092	0.109727	0.120191	0.244301	0.506374	-0.89322
MW	-0.99908	-0.49584	-0.42263	-0.37356	-0.28921	-0.68279	-0.81078	0.998168
DD3	-0.75412	-0.47375	-0.18984	-0.25492	0.189057	-0.237	-0.3678	0.963004
DRDRAC	-0.99469	0.413425	-0.52833	-0.26445	-0.30826	-0.67307	-0.7527	0.992125
POL	-1	-0.48796	-0.58921	-0.4938	-0.14918	-0.70243	-0.79742	0.997436
DD2	-0.75779	-0.47191	-0.17533	-0.22807	0.213503	-0.17377	-0.3223	0.949084
DRDRDR	-0.99927	-0.29904	-0.58777	-0.31741	0.12075	-0.70954	-0.76147	0.9837
HSA	-0.9978	-0.43197	-0.68578	-0.47652	0.244809	-0.67868	-0.75593	0.992857
DD1	-0.58666	-0.45833	-0.1843	-0.20505	0.242936	-0.19743	-0.31342	0.960256
V	-0.99982	-0.53992	-0.58333	-0.48211	-0.17295	-0.67802	-0.80079	0.996337
S	-0.99927	-0.54249	-0.58305	-0.44528	-0.19662	-0.67091	-0.80726	0.998168
R	-0.99982	-0.27575	-0.58439	-0.46023	0.239179	-0.70262	-0.62701	0.810989
A	-0.66612	-0.57498	-0.23375	-0.41774	0.26807	-0.15156	0.107021	-0.64341

RC9	RC10	RC11	RC12	RC13	RC14	RC15	RC16	RC17
-0.97094	-0.34431	0.427304	0.719811	-0.72382	0.329205	0.249199	0.800479	-0.2795
0.814988	-0.37162	-0.52932	-0.67659	-0.55188	-0.27405	0.213477	-0.84663	0.161905
0.895984	0.309083	-0.4497	-0.62387	0.527783	-0.30684	-0.19275	-0.83488	0.281938
-0.62684	0.362403	-0.26623	0.264978	-0.65145	-0.13713	0.138014	0.444253	-0.17603
-0.72475	-0.79417	-0.44416	0.50826	-0.84651	0.450801	0.447008	-0.4909	0.471328
0.794756	0.442405	-0.41136	-0.38762	0.40033	-0.32655	-0.23932	-0.616	-0.15671
-0.75299	-0.73513	-0.39491	0.458865	-0.79204	0.43432	0.413246	-0.50639	0.440907
-0.28576	0.493696	-0.32565	0.2042	-0.588	-0.21933	-0.21808	0.445782	-0.26729
0.213512	0.439094	-0.44229	0.133112	-0.66741	-0.24661	-0.23822	0.457345	-0.27787
0.834236	0.449413	-0.43435	-0.38673	-0.46037	-0.30456	-0.23552	-0.47149	-0.15485
0.813877	0.40016	-0.49061	-0.35514	-0.52217	-0.33391	-0.24811	-0.482	-0.17914
0.220679	-0.52969	-0.45768	0.118577	-0.47937	0.281211	0.206356	0.195469	0.488772
-0.7251	-0.8021	-0.43582	0.46651	-0.86209	0.447204	0.442945	-0.49588	0.470581
0.500348	0.201562	-0.61763	0.122741	-0.66411	-0.31839	-0.1644	0.390641	-0.27837
0.500348	0.201562	-0.61763	0.122741	-0.66411	-0.31839	-0.1644	0.390641	-0.27837
-0.62304	-0.63848	-0.28957	0.412795	-0.42351	0.429596	0.414547	-0.52425	0.379667
0.474009	0.317571	-0.30472	-0.22965	0.362553	-0.19564	-0.30225	0.148393	-0.35849
0.474009	0.317571	-0.30472	-0.22965	0.362553	-0.19564	-0.30225	0.148393	-0.35849
-0.70177	-0.83622	-0.44361	0.436102	-0.8344	0.4849	0.408908	-0.51741	0.484305
-0.49193	-0.7713	-0.48157	0.264719	-0.73684	0.31235	0.409335	-0.72111	0.45258
0.881276	0.239527	-0.62929	-0.29258	-0.50951	-0.35344	-0.18279	-0.41717	-0.21364
-0.47808	-0.82202	-0.5147	0.298991	-0.81405	0.325777	0.44296	-0.66929	0.452961
0.83183	0.494812	-0.43174	-0.4532	-0.42461	-0.37959	-0.27768	-0.29372	-0.22165
-0.50152	-0.82423	-0.48526	0.29046	-0.84045	0.343962	0.413875	-0.64237	0.479468
0.826122	0.449773	-0.58763	-0.20611	-0.45156	-0.18127	-0.18496	-0.52264	-0.31898
0.811286	0.486697	-0.4534	-0.39631	-0.43726	-0.41141	-0.3155	-0.29741	-0.25931
0.799112	0.454766	-0.32787	-0.22026	0.762768	-0.27747	-0.16309	-0.38416	-0.3411
0.689984	0.408649	-0.59171	-0.18338	0.334082	-0.2555	-0.16717	-0.25197	-0.33029
0.79504	0.459737	-0.59171	-0.16561	0.335549	-0.2555	-0.18705	-0.32428	-0.33029
0.355029	0.517663	-0.36343	-0.08052	-0.56691	-0.36386	-0.29696	0.515087	-0.31755
-0.46799	-0.84508	-0.49638	0.266233	-0.8135	0.383926	0.369109	-0.61577	0.500058
0.221314	0.387812	0.683562	-0.1138	-0.61276	0.20209	0.279209	-0.30463	0.35651
-0.7619	-0.69307	-0.36955	0.447732	-0.71264	0.422566	0.379058	-0.56416	0.4168
0.858169	0.426368	-0.50966	-0.23623	-0.48622	-0.23141	-0.17635	-0.50362	0.145369
0.218908	0.489435	0.630784	0.105112	-0.53537	0.197737	0.317367	-0.24079	0.34167
0.839633	0.642364	-0.27864	-0.45791	0.538603	-0.39248	-0.22541	0.268176	-0.21703
-0.17899	0.237683	0.470407	-0.09332	-0.66686	0.27916	0.173048	-0.34104	0.467973
0.22977	0.429679	0.601537	-0.06308	-0.65879	0.222164	0.260094	-0.24079	0.370607
0.84426	0.610457	-0.31696	-0.43902	0.476435	-0.42165	-0.2338	0.301552	-0.24522
0.825936	0.563242	-0.36472	-0.41852	0.429488	-0.43414	-0.31846	-0.21699	-0.28249
0.481441	0.569305	-0.26956	-0.14469	-0.47082	-0.38337	-0.29819	0.630439	-0.29826
0.234838	0.357196	0.524144	-0.07453	-0.66631	0.256256	0.178601	-0.25738	0.394149
-0.61204	0.327521	-0.37528	0.48528	-0.69767	-0.15131	-0.27414	0.719213	-0.35613
0.523027	0.518401	0.251756	-0.20441	0.495943	-0.28942	-0.3398	0.159088	-0.35423
0.836116	0.566931	-0.33288	-0.44792	0.450578	-0.40649	-0.28941	-0.205	-0.2324
0.120195	0.525584	0.670777	0.131965	0.452778	-0.1765	0.323179	-0.24079	-0.29827
0.872791	0.458299	-0.35512	-0.42878	0.758115	-0.22517	-0.17498	-0.72469	0.215889
0.841084	0.449441	0.311569	-0.37745	0.837337	-0.28506	-0.18392	-0.6272	-0.33399

0.605574	0.647542	-0.24272	-0.18175	0.387493	-0.36479	-0.22704	0.687603	-0.27135
-0.36988	-0.72832	-0.47101	0.196984	-0.62443	0.28602	0.366543	-0.76887	0.487469
-0.64366	-0.82276	-0.38807	0.401451	-0.80451	0.480168	0.335506	-0.52578	0.484649
-0.61389	0.31996	-0.38306	0.476758	-0.69968	-0.16002	-0.24639	0.710546	-0.35479
0.589427	0.524303	0.265849	-0.2506	0.590203	-0.2953	-0.27899	-0.15269	-0.36535
0.155176	0.528535	0.690965	0.155172	0.548322	-0.19638	0.304411	-0.24079	-0.32572
0.18761	-0.3624	-0.35122	-0.10407	-0.85373	-0.23082	-0.12684	0.203296	-0.29245
0.542559	0.571702	0.341946	-0.22553	0.612576	-0.28734	-0.33474	0.155585	-0.4266
-0.42139	-0.82866	-0.43325	0.230714	-0.78837	0.392452	0.32015	-0.59591	0.511922
0.527411	0.568382	0.32618	-0.20824	0.603223	-0.29548	-0.32476	0.16167	-0.41028
0.189984	-0.24216	0.355644	0.127166	-0.58892	0.391317	0.160036	-0.48313	0.26598
-0.45428	-0.4963	-0.53637	0.358263	-0.88924	0.259091	-0.24277	0.409159	0.103604
-0.49842	-0.46218	-0.52026	0.351897	-0.88135	0.235422	-0.30557	0.45539	-0.12259
-0.48152	-0.39394	-0.49286	0.324773	-0.85458	0.196786	-0.33604	0.500937	-0.19754
0.170614	-0.32678	0.497479	-0.27136	0.55591	0.297541	0.217721	-0.6478	0.310692
0.158823	-0.33121	0.497849	-0.26536	0.697666	0.263636	0.303117	-0.57715	0.345539
0.158823	-0.32162	0.509143	-0.25942	0.754149	0.243376	0.323042	-0.53774	0.324793
0.1701	-0.33822	0.488592	-0.2826	0.583234	0.282965	0.27323	-0.66365	0.321819
0.184904	0.539417	0.695023	0.132336	0.642765	-0.2606	0.23847	-0.24079	-0.36875
0.151187	-0.29967	0.504141	-0.27054	0.797245	0.223821	0.33878	-0.51545	0.324236
0.154888	-0.19512	0.525063	-0.24683	0.845475	0.175146	0.351049	-0.47525	0.263527
-0.70783	-0.64532	-0.27494	0.283756	-0.66716	0.371435	0.313543	-0.46575	0.328863
-0.48004	-0.33583	-0.46972	0.32645	-0.7495	0.136	-0.36159	0.520114	-0.29659
-0.5018	-0.27349	-0.49064	0.332007	-0.59992	-0.1608	-0.36289	0.585285	-0.3042
-0.6077	-0.6195	-0.72226	-0.49921	-0.91876	0.14352	0.128825	-0.39923	0.200615
-0.62314	-0.22314	-0.46416	0.336412	-0.55848	-0.1805	-0.38196	0.545193	-0.3706
0.700192	-0.24123	-0.48561	-0.44112	-0.35137	-0.15854	0.117432	-0.28338	0.141135
-0.20422	0.520652	0.431939	0.123594	0.830254	-0.35685	0.157867	0.429894	-0.49154
-0.71261	-0.23271	0.587644	0.13919	0.612942	0.370674	0.227213	-0.18482	0.282132
-0.71154	0.229242	-0.45009	0.344193	-0.44893	-0.21233	-0.3727	0.57293	-0.38118
-0.59585	-0.60825	-0.71411	-0.52299	-0.91638	0.15848	0.13578	-0.46306	0.226955
-0.24493	0.261144	0.543208	-0.23939	0.875848	0.132578	0.321517	-0.4316	0.189485
-0.37615	0.32754	0.357705	0.089095	-0.67082	-0.21946	0.119625	0.12448	-0.30988
0.71893	0.707852	-0.20089	-0.3431	0.576563	-0.42219	-0.37142	0.667081	-0.4023
-0.52794	0.188047	-0.24401	0.263572	-0.55756	0.077799	-0.15458	0.59696	-0.2741
-0.21014	-0.64956	-0.3864	-0.2883	-0.55071	0.248316	0.297464	-0.66324	0.380006
-0.15824	-0.61675	-0.29549	-0.29614	0.677677	0.266693	0.262435	-0.71207	0.364817
-0.80864	0.294167	-0.31753	0.489211	-0.80616	0.125584	-0.29528	0.710099	-0.31696
-0.74208	0.235328	-0.44065	0.353185	-0.37539	-0.2127	-0.35076	0.614051	-0.38002
0.677784	0.589616	-0.27901	-0.23718	0.396364	-0.36762	-0.14588	0.61508	-0.24428
-0.72444	0.245108	-0.46398	0.353741	0.424239	-0.21041	-0.17971	0.627804	-0.39824
-0.72277	0.237726	-0.44843	0.367417	0.446429	-0.21117	-0.28911	0.665183	-0.3841
-0.72592	0.232008	-0.44972	0.373622	0.455598	-0.21025	-0.22959	0.655517	-0.38117
0.151927	-0.26053	0.380093	-0.34107	0.847859	0.216664	0.222448	-0.64569	0.244603
-0.22907	0.552904	0.31288	0.130198	0.35602	-0.23693	-0.30688	-0.39863	-0.23351
0.711626	0.135483	-0.54544	-0.23443	-0.28413	-0.33222	0.090154	0.418563	-0.29357
-0.712	-0.44631	0.151626	0.502735	-0.6457	0.309253	-0.12382	0.311776	-0.15927
-0.71835	-0.36013	0.579115	0.169234	0.381143	0.397197	0.295693	-0.28703	0.365042
0.853941	0.550521	-0.27363	-0.46878	0.348799	-0.32426	-0.14091	0.161753	-0.14597
0.266844	-0.56731	-0.28493	-0.41353	0.593687	0.243385	0.219913	-0.70396	0.351459

-0.62781	-0.50496	0.31824	0.187374	0.409018	0.34167	0.263788	-0.35888	0.313811
0.269164	-0.47157	-0.69393	-0.64813	-0.81368	-0.27541	-0.18507	-0.58561	0.236312
-0.43457	0.150105	-0.48135	0.189973	-0.73086	-0.15492	-0.10665	0.441827	-0.31328
-0.1926	-0.23882	0.26105	-0.19406	0.52345	0.221799	0.183908	-0.46722	0.172844
0.85441	0.363117	0.31658	-0.41588	0.827801	-0.25211	-0.16554	-0.65615	-0.34568
-0.73328	-0.42894	0.450451	0.154972	0.655121	0.327688	0.289547	-0.32539	0.225532
0.818603	0.149722	-0.54227	-0.34399	0.284866	-0.37256	0.091469	0.212404	-0.24905
0.844288	0.553084	-0.34844	-0.41365	0.489708	-0.39892	-0.16207	0.237274	-0.21717
0.158957	-0.40405	-0.29121	-0.24452	-0.41628	-0.07732	0.100533	-0.45711	0.231841
-0.66192	-0.3991	0.141446	0.373428	-0.5441	0.221729	-0.07663	0.37537	-0.12106
0.243221	-0.22093	-0.27521	-0.27252	-0.41023	-0.08205	0.106392	-0.45049	0.280224
-0.2206	0.401843	0.29659	-0.13496	-0.34091	-0.26782	-0.32802	0.355033	-0.22886
-0.26353	0.263542	-0.24677	0.095826	-0.59222	-0.22803	-0.22144	0.471806	-0.32624
0.761585	0.770191	0.310301	-0.38639	0.721988	-0.47201	-0.35417	0.680304	-0.39155
0.238555	-0.37822	-0.28879	-0.25059	-0.25516	-0.08205	0.075823	-0.49828	0.260964
0.187345	-0.3537	-0.32287	-0.25266	-0.34678	-0.08243	0.088894	-0.48179	0.202268
0.352863	-0.26558	-0.31336	-0.35282	-0.3628	-0.0813	0.076938	-0.37684	0.263357
-0.60865	-0.24306	-0.21104	0.266618	-0.41298	0.163964	-0.10833	0.4883	-0.17873
0.954285	0.36699	-0.38972	-0.68444	0.790391	-0.28583	-0.19914	-0.85037	0.276909
0.392684	-0.31348	-0.27653	-0.2995	0.334678	-0.08793	-0.0627	-0.36781	0.231637
0.923006	0.261158	-0.34549	-0.55487	0.729323	-0.19413	-0.12492	-0.78726	0.233437
0.967611	0.341912	-0.32677	-0.68968	0.876215	-0.24547	-0.1816	-0.83249	0.331828
0.350243	-0.23565	-0.24636	-0.33056	0.309807	-0.09287	-0.08155	-0.32777	0.221952
0.911502	-0.37051	-0.37397	-0.57451	0.682927	-0.14717	0.136943	-0.81147	0.263921
0.904041	-0.28438	-0.30788	-0.6404	0.701449	-0.22899	-0.17497	-0.82556	0.309564
0.372823	-0.222	-0.30063	-0.31778	-0.26444	-0.08512	0.058727	-0.27465	0.199345
0.966315	0.289745	-0.34732	-0.6903	0.833303	-0.29415	-0.17902	-0.83415	0.282497
0.948177	0.217447	-0.42303	-0.68554	0.700165	-0.29283	-0.1782	-0.84387	0.260609
0.912028	0.767793	0.651349	-0.49139	0.973226	-0.16952	-0.19482	-0.7008	0.341283
0.162151	0.166331	-0.26633	-0.13804	0.294586	-0.12023	-0.22954	-0.26384	-0.15147

RC18	RC19	RC20	RC21	RC22	RC23	RC24	RC25	RC26
-0.16011	0.489956	-0.99945	-0.96155	0.334747	-0.54338	0.29104	0.544654	-0.99322
-0.127	-0.39843	0.999451	0.829389	-0.30638	0.591515	-0.23031	-0.48684	0.991755
-0.12459	-0.30067	0.999267	0.866549	0.345431	0.545229	-0.30757	-0.4692	0.994137
-0.12083	0.377778	-0.89212	-0.75294	-0.19299	-0.10893	-0.03758	0.132663	-0.56926
-0.11889	0.367235	-0.89579	-0.51918	0.612636	-0.05473	0.061143	0.443766	-0.82778
-0.11517	-0.14028	0.998535	0.791045	-0.31879	0.390932	-0.25941	-0.41452	0.993587
-0.11415	0.344013	-0.9174	-0.56609	0.539694	-0.04711	0.058557	0.42095	-0.78014
-0.11404	0.330963	0.532418	-0.4005	-0.28644	-0.09164	-0.11673	-0.16186	0.635214
-0.11369	0.30937	0.745238	-0.28847	-0.29755	0.077156	-0.10711	-0.185	0.746977
-0.11302	-0.1415	0.997619	0.847314	-0.30768	0.32361	-0.23926	-0.38068	0.992671
-0.11091	-0.11115	0.998352	0.811074	-0.32782	0.318511	-0.20956	-0.39021	0.992305
-0.10983	0.266873	0.982784	0.319029	-0.67618	-0.1151	0.073402	-0.11523	0.76768
-0.10966	0.333732	-0.86978	-0.48412	0.673973	-0.06668	0.07079	0.470093	-0.87028
-0.10836	0.214365	0.950183	0.500493	-0.59195	0.097857	-0.05204	-0.22964	0.913888
-0.10836	0.214365	0.950183	0.500493	-0.59195	0.097857	-0.05204	-0.22964	0.913888
-0.10535	0.300627	-0.93626	-0.47302	0.282436	-0.07278	0.0898	0.439573	-0.91609
-0.10373	-0.13896	0.986813	0.492458	-0.39142	0.097679	0.049524	-0.35946	0.991206
-0.10373	-0.13896	0.950366	0.492458	-0.39142	0.097679	0.051307	-0.35946	0.97838
-0.10001	0.313768	-0.85751	-0.46969	0.713086	-0.09249	0.070682	0.448831	-0.87431
-0.09983	0.253241	-0.58645	-0.33322	0.563887	0.205387	-0.0717	0.260782	-0.35086
-0.09886	0.080832	0.998901	0.858455	-0.58894	0.277164	-0.13424	-0.38077	0.991572
-0.09817	0.264607	-0.63553	-0.33451	0.62633	0.173656	-0.08832	0.286873	-0.56944
-0.09773	-0.13575	0.997985	0.832046	-0.34458	0.316715	-0.21622	-0.41476	0.992488
-0.09664	0.263485	-0.63095	-0.33046	0.69184	0.142457	-0.09314	0.300487	-0.7347
-0.09044	0.189898	0.998168	0.846497	0.450066	0.192134	-0.23008	-0.38597	0.992122
-0.09004	-0.12477	0.998168	0.814018	-0.33892	0.293437	-0.19043	-0.4186	0.992671
-0.08829	0.179867	0.998718	0.732281	-0.48775	0.198054	-0.1731	-0.40302	0.995969
-0.08673	0.161281	0.998352	0.628712	-0.47246	0.200396	-0.13044	-0.36714	0.994687
-0.08673	0.19674	0.998535	0.72014	-0.47246	0.20036	-0.15662	-0.36714	0.993771
-0.08654	0.219371	0.880769	0.414849	-0.33647	0.099758	-0.09979	-0.27832	0.881825
-0.08642	0.221025	-0.66484	-0.34463	0.723033	0.117028	-0.09822	0.298648	-0.78582
-0.08634	-0.14182	-0.88718	0.279463	0.754409	0.119195	-0.06297	0.215412	-0.94833
-0.08445	0.276375	-0.92106	-0.59139	0.484683	-0.03506	0.06662	0.435642	-0.76933
-0.08394	0.211054	0.997619	0.792701	-0.27009	0.224688	-0.27983	-0.32743	0.991206
-0.08385	-0.10849	-0.94799	0.258565	0.881073	0.05545	-0.063	0.171294	-0.98021
-0.0774	-0.15201	0.999451	0.888889	-0.41254	0.265204	-0.22482	-0.37125	0.995053
-0.07701	-0.32293	-0.94799	0.265475	0.860569	-0.05477	-0.07478	0.235302	-0.98021
-0.07674	-0.15569	-0.94799	0.257932	0.880583	-0.0521	-0.072	0.190333	-0.98021
-0.07605	-0.14456	0.999634	0.86387	-0.37594	0.241527	-0.21323	-0.41723	0.99432
-0.07502	-0.11324	0.998718	0.850258	-0.32418	0.276699	-0.21066	-0.4385	0.993954
-0.0748	0.201876	0.934432	0.533319	-0.32081	0.108019	-0.10969	-0.31968	0.931294
-0.07476	-0.27669	-0.94799	0.307049	0.888688	-0.0574	-0.08636	0.21249	-0.98021
-0.07454	0.386559	-0.60037	-0.64392	-0.46062	-0.32232	0.172645	0.261201	0.602602
-0.07436	-0.06541	0.987729	0.684327	-0.54381	0.076441	-0.05498	-0.37782	0.992305
-0.07418	-0.11932	0.998352	0.864422	-0.35961	0.291038	-0.2432	-0.42156	0.993404
-0.07367	0.132964	-0.94799	-0.26104	0.8656	0.070463	-0.06479	0.191868	-0.98021
-0.07361	0.20767	0.998535	0.880795	0.413647	0.351166	-0.355	-0.40631	0.995236
-0.07345	0.161525	0.998718	0.904157	0.298104	0.277547	-0.25774	-0.35806	0.99542

-0.07101	0.204254	0.96044	0.639095	-0.40192	0.097093	-0.14907	-0.25454	0.957127
-0.07046	0.170955	0.614286	-0.29238	0.484988	0.257321	-0.08006	0.244445	0.683034
-0.06978	0.227866	-0.8359	-0.40659	0.741205	-0.1099	-0.05729	0.401387	-0.90161
-0.06818	0.3839	-0.60641	-0.64503	-0.46633	-0.32805	0.166872	0.254818	0.594174
-0.06507	0.103083	0.992308	0.753966	-0.63757	0.101324	-0.13387	-0.34394	0.991206
-0.06426	0.215256	-0.94799	-0.23302	0.849575	0.078742	-0.06228	0.173602	-0.98021
-0.064	-0.11925	-0.75476	-0.21316	-0.53601	-0.11595	0.091278	0.148888	-0.72389
-0.06321	0.078166	0.991392	0.678256	-0.67109	0.087084	-0.1039	-0.35186	0.992671
-0.06317	0.164009	-0.67381	-0.35722	0.749862	0.087372	-0.10425	0.28468	-0.85104
-0.06002	0.04494	0.989744	0.700515	-0.64438	0.083123	-0.09079	-0.37383	0.993221
-0.05677	-0.18799	-0.63077	0.184319	0.518696	-0.14865	-0.04863	0.17508	0.532613
-0.05555	-0.18866	0.809524	-0.54728	-0.69497	-0.23686	0.104509	0.095205	-0.81715
-0.04638	-0.14932	0.602747	-0.60008	-0.67471	-0.2533	0.119022	0.105388	-0.86992
-0.04503	0.153246	-0.55147	-0.60578	-0.65132	-0.26378	0.131677	0.107929	-0.90015
-0.04221	-0.3081	-0.7967	0.375747	0.590728	0.166963	-0.11202	0.113264	-0.88842
-0.03889	-0.23325	-0.81795	0.293436	0.599569	0.159999	-0.1171	0.117952	-0.76676
-0.03678	-0.20774	-0.8196	-0.27664	0.607674	0.161368	-0.11358	0.127713	-0.76035
-0.03401	-0.27116	-0.81172	0.363422	0.592754	0.163589	-0.11752	0.113735	-0.78967
-0.0337	0.265472	-0.94799	-0.22337	0.838771	0.086684	-0.06466	0.168103	-0.98021
0.04162	-0.18577	-0.81484	-0.29385	0.613384	0.160604	-0.10512	0.121657	-0.75394
0.047875	-0.15097	-0.79176	-0.26202	0.622041	0.147955	-0.10539	0.139771	-0.7314
0.055127	0.105488	-0.91868	-0.45904	0.44306	-0.06395	0.076774	0.476897	-0.8232
0.05671	0.189895	-0.72527	-0.60486	-0.62719	-0.26277	0.141	0.122844	-0.90528
0.058393	0.214871	-0.65586	-0.60339	-0.65463	-0.27972	0.167567	0.103517	-0.87761
0.058767	-0.44895	-0.79396	-0.53939	-0.28496	0.317018	0.16391	-0.25231	-0.88018
0.059876	0.222226	-0.80861	-0.61322	-0.6049	-0.2741	0.15865	0.139701	-0.8974
0.060188	-0.36937	0.999817	0.834253	0.381902	0.241411	-0.06547	-0.24778	0.832539
0.060685	0.29456	-0.75092	-0.41524	0.483514	-0.09897	0.05398	-0.10703	0.715097
0.061847	-0.14356	-0.9989	-0.81063	0.626145	-0.1779	0.100964	0.308319	-0.99432
0.063731	0.233165	-0.81593	-0.64261	-0.59772	-0.28641	0.166319	0.14552	-0.89007
0.064286	-0.4861	-0.7859	-0.56449	-0.26138	0.323859	0.163128	-0.28263	-0.87523
0.065778	0.115481	-0.763	-0.25007	0.635609	0.15144	-0.09391	0.132637	-0.7173
0.066208	-0.21257	0.826374	0.24741	-0.5618	-0.08539	-0.05184	-0.14025	-0.72243
0.066735	-0.13904	0.933516	0.654364	-0.38607	0.087583	-0.10955	-0.43043	0.860938
0.068574	0.123884	-0.91007	-0.748	-0.16788	-0.17736	0.076026	0.103891	0.48351
0.06918	-0.26784	0.764286	0.341008	0.393448	0.26226	-0.06243	0.226467	0.645841
0.06974	-0.22543	-0.93223	-0.15034	0.505192	0.246879	-0.10905	0.153965	-0.77244
0.070145	0.311469	-0.99615	-0.74753	-0.4869	-0.37249	0.211244	0.212899	-0.98626
0.070616	0.244166	-0.81941	-0.69982	-0.58777	-0.29319	0.174072	0.145003	-0.87871
0.071338	0.086942	0.98956	0.674864	0.37085	0.120863	-0.14683	-0.24411	0.976915
0.071914	0.33578	-0.53462	-0.76272	-0.62369	-0.2746	0.174692	0.137214	-0.69806
0.072818	0.27765	-0.80604	-0.7491	-0.58722	-0.29256	0.176077	0.148195	-0.83199
0.075086	0.326243	-0.73132	-0.75386	-0.59864	-0.29072	0.182495	0.155611	-0.74863
0.076181	-0.20411	-0.96044	-0.276	0.516681	0.214079	-0.10693	-0.10375	-0.48644
0.076585	-0.23361	-0.5511	-0.27717	-0.23123	-0.05076	0.047422	-0.29766	-0.64505
0.077422	-0.10523	0.998535	0.697859	-0.3011	0.125644	-0.08385	-0.18184	0.979663
0.077977	0.316975	-0.97289	-0.86553	-0.4597	-0.28546	0.145753	0.311766	-0.8384
0.079087	-0.21625	-0.9978	-0.76668	0.670353	-0.12858	0.115304	0.339756	-0.99322
0.079396	-0.18524	0.999634	0.912987	0.335922	0.274404	-0.19793	-0.32046	0.99432
0.081169	-0.36842	0.970147	0.312943	0.321986	0.271692	-0.05673	0.161003	0.757237

0.081276	-0.20128	-0.89451	-0.5545	0.357104	-0.10397	0.121408	0.444892	-0.85306
0.083465	-0.53729	0.982784	0.445489	-0.62074	0.540823	-0.11035	-0.54127	-0.6015
0.085828	-0.07642	-0.94249	-0.59444	-0.13177	-0.08896	0.040999	-0.10183	0.596372
0.086644	-0.24688	-0.98736	0.34106	0.607853	0.085224	0.043966	0.19944	-0.98223
0.08789	-0.14643	0.999451	0.946836	-0.29662	0.308089	-0.23266	-0.34806	0.996519
0.094384	-0.14373	-0.99249	-0.79756	0.504461	-0.10583	0.120468	0.30925	-0.99084
0.094807	-0.16726	1	0.863135	0.336716	0.20075	-0.137	-0.27556	0.993587
0.09929	-0.16266	0.999817	0.889993	0.341194	0.235595	-0.20735	-0.39471	0.99542
0.101495	-0.20587	0.918681	0.209993	0.36031	0.120207	0.059189	-0.39881	0.254306
0.102223	0.139008	-0.97344	-0.866	0.24848	-0.26689	0.14494	0.290276	-0.7576
0.105172	-0.23332	-0.75549	0.307932	0.284473	0.116839	-0.06014	-0.40252	0.365885
0.105413	-0.32249	0.56337	-0.35727	0.246707	-0.08208	0.065726	-0.31867	0.528765
0.107192	-0.28381	-0.67179	-0.58317	-0.12048	-0.10203	0.059086	-0.22274	0.603884
0.107227	-0.1925	0.944139	0.67975	-0.47879	0.096663	-0.13529	-0.41311	0.88384
0.107594	-0.20549	0.561538	0.266851	0.328019	0.108836	0.059623	-0.40252	0.486625
0.110783	-0.20568	0.967949	0.182031	0.302899	0.119817	0.059406	-0.40271	0.30982
0.111457	-0.23857	0.710989	0.426851	0.277048	0.126166	0.060274	-0.41707	0.383107
0.118553	0.05485	-0.95806	-0.80658	-0.24793	-0.2202	0.141078	0.210493	-0.57787
0.118641	-0.3625	0.998901	0.972222	0.388475	0.468098	-0.30447	-0.49332	0.991389
0.125	-0.25659	0.900916	0.350427	0.241987	0.147984	-0.06009	-0.41369	0.633932
0.126481	-0.17336	0.999451	0.935247	0.401179	0.422467	-0.30998	-0.36128	0.99542
0.128467	-0.42488	0.999451	0.974614	0.329843	0.466976	-0.31322	-0.48064	0.991206
0.128777	-0.22435	0.931502	0.267829	0.312719	0.138389	-0.0694	-0.41163	0.706486
0.130249	-0.22863	0.999267	0.93911	0.373244	0.407988	-0.26821	-0.38504	0.99432
0.131596	-0.43159	0.999451	0.930831	0.369319	0.48807	-0.24333	-0.45624	0.988091
0.133339	-0.26384	0.954762	0.370191	0.202581	0.095459	-0.06931	-0.40868	0.535483
0.137171	-0.42051	0.999451	0.973326	-0.29643	0.51342	-0.30513	-0.51871	0.992488
0.138414	-0.41034	0.999634	0.964312	0.319586	0.550947	-0.29571	-0.52486	0.993221
0.160563	-0.293	0.980952	0.921553	0.377124	0.176517	-0.2734	-0.24778	0.924881
0.163975	-0.29368	0.662088	-0.34191	0.17597	0.069393	-0.07603	-0.18373	0.982411

RC27	RC28	RC29	RC30	RC31	RC32	RC33	RC34	RC35
0.931243	-0.99927	-0.97051	-0.60368	-0.99982	-0.97783	-0.2733	-0.33291	-0.99285
-0.9835	0.998352	0.862979	0.350727	0.997985	0.99542	0.297866	0.350803	0.993954
-0.98368	0.999817	0.954937	0.675184	0.999634	0.993039	0.349835	0.334323	0.991206
-0.35345	-0.61172	-0.83971	0.552547	-0.79066	0.521524	0.07302	0.082066	-0.93789
-0.9659	-0.79505	-0.59755	-0.51065	-0.83333	0.671552	0.10186	-0.28983	-0.86625
-0.85974	0.999817	0.928375	0.799816	0.999451	0.994688	0.343582	0.312359	0.981129
-0.96388	-0.83938	-0.65525	-0.58878	-0.83993	0.610185	0.07924	-0.25636	-0.87065
-0.28891	0.760073	-0.56036	0.647759	0.695604	0.805825	0.127464	0.113706	-0.6343
-0.26086	0.875641	0.514014	0.720746	0.834799	0.909141	0.144706	0.152044	-0.4923
-0.69949	0.999634	0.92288	0.794485	0.999451	0.992673	0.33378	0.295337	0.980029
-0.72186	0.999634	0.92288	0.818374	0.999267	0.995787	0.311937	0.306449	0.974716
-0.66407	0.911172	0.966477	0.63069	-0.91575	0.94706	0.078682	0.087283	0.846647
-0.98001	-0.76557	-0.57996	-0.45992	-0.80476	0.693167	0.106079	-0.29537	-0.89831
-0.47983	0.983333	0.850522	0.891728	0.982784	0.98388	0.121897	0.221663	0.622023
-0.47983	0.983333	0.850522	0.891728	0.982784	0.98388	0.121897	0.221663	0.622023
-0.71085	-0.85458	-0.79722	-0.82554	-0.85073	0.566954	0.057572	-0.2409	-0.87431
0.305988	0.993407	0.646272	0.823346	0.993223	0.625389	0.036655	0.236967	0.599304
0.305988	0.99652	0.646272	0.814522	0.995055	0.625389	0.036655	0.236967	0.599304
-0.98001	-0.76996	-0.5098	-0.43824	-0.7619	0.674666	0.133762	-0.27815	-0.928
-0.97855	0.712271	0.597362	-0.41782	0.736447	0.985712	0.163277	-0.13493	-0.58465
-0.8524	0.999634	0.96721	0.93989	0.999817	0.997069	0.243102	0.337253	0.969219
-0.97671	0.739377	0.668804	-0.39871	0.626557	0.989375	0.179008	-0.16174	-0.65427
-0.57845	0.999451	0.908958	0.845763	0.999817	0.992123	0.28662	0.285136	0.975082
-0.98166	0.702747	0.700311	-0.36121	0.61044	0.988093	0.188789	-0.16511	-0.79168
-0.61051	0.999267	0.946877	0.86636	0.999451	0.996703	0.263767	0.254059	0.989557
-0.60137	0.999084	0.905477	0.863778	0.999634	0.993772	0.259805	0.318619	0.971418
-0.59385	0.999451	0.947426	0.869853	0.998901	0.983697	0.148977	0.179578	0.973983
-0.64705	0.998901	0.938817	0.931985	0.999451	0.828357	0.141961	0.194221	0.780689
-0.64705	0.999267	0.942847	0.906985	0.999451	0.993955	0.141961	0.199623	0.987908
0.268977	0.918681	0.581242	0.7875	0.921978	0.941748	0.103797	0.193653	0.536827
-0.98166	0.629121	0.75087	-0.30754	0.664103	0.978201	0.191231	-0.15724	-0.88201
-0.54767	0.544139	-0.85016	-0.74815	-0.88498	-0.8791	-0.03487	-0.11518	-0.51667
-0.8183	-0.90256	-0.70727	-0.65348	-0.87198	-0.50925	-0.056	-0.25588	-0.8679
-0.6366	0.999451	0.950724	0.806066	0.999267	0.998351	0.36594	0.283556	0.988274
-0.56601	0.515018	-0.93204	-0.82039	-0.9467	-0.94285	-0.07144	-0.07623	0.528948
-0.4303	0.999634	0.93094	0.880874	1	0.749954	0.184438	0.110775	0.973983
0.347635	-0.54744	-0.93204	-0.83768	-0.9467	-0.94285	0.06651	-0.10133	-0.74679
-0.52216	0.589011	-0.93204	-0.82039	-0.9467	-0.94285	-0.05678	-0.13222	-0.59674
-0.44387	0.999084	0.931856	0.895588	0.999817	0.864261	0.172523	0.118046	0.96812
-0.53408	0.998352	0.920315	0.8783	1	0.988643	0.223605	0.301777	0.970136
0.293729	0.938462	0.684008	0.853493	0.95348	0.898699	0.077229	0.177954	0.639612
-0.44387	0.589011	-0.93204	-0.82039	-0.9467	-0.94285	0.054079	-0.13525	-0.73598
0.980565	-0.91429	-0.86884	0.782896	0.614286	0.70654	-0.1765	-0.14452	-0.9945
0.409765	0.989927	0.816084	0.835478	0.992125	0.514014	-0.05219	0.155841	0.743679
-0.48952	0.999451	0.923246	0.870947	1	0.977285	0.237153	0.242096	0.973433
-0.62997	-0.5196	-0.93204	-0.82039	-0.9467	-0.94285	-0.11197	-0.08122	0.615427
-0.86157	0.999451	0.960432	0.644293	0.999084	0.99542	0.374406	0.280314	0.988641
-0.71195	0.999634	0.968492	0.841544	0.999267	0.99194	0.288669	0.217715	0.988457

0.264707	0.959158	0.769188	0.884007	0.96685	0.48104	0.05113	-0.08866	0.721693
-0.93729	0.661538	0.556879	-0.49667	0.814469	0.962631	0.127712	-0.11173	-0.41883
-0.98001	-0.74084	0.508518	-0.4136	-0.75238	0.610368	0.144052	-0.23787	-0.90216
0.971947	-0.91465	-0.87049	0.774256	0.605861	0.69793	-0.18037	-0.14832	-0.9945
0.314789	0.991758	0.878916	0.930147	0.995604	0.875985	0.090679	0.115006	0.786185
-0.68115	-0.59267	-0.93204	-0.82039	-0.9467	-0.94285	-0.12294	-0.11881	0.693111
0.677668	-0.68956	-0.91189	0.539128	0.971795	0.9597	-0.10877	0.201435	0.672591
0.438368	0.988645	0.845759	0.912316	0.993223	0.6939	-0.05839	0.119836	0.752657
-0.98166	0.613919	0.739513	-0.30257	0.600183	0.919399	0.199526	-0.14572	-0.86057
0.444235	0.988462	0.797399	0.861021	0.992674	0.576296	-0.06352	0.125239	0.734335
0.556289	-0.56575	0.576479	0.272418	0.595238	-0.69646	0.048169	-0.14949	-0.44155
0.93766	-0.99212	-0.81718	0.588218	0.774725	0.575014	-0.07669	-0.07078	-0.99872
0.944994	-0.99451	-0.90786	0.578843	0.785897	0.618428	-0.10968	0.063423	-0.99853
0.950312	-0.99762	-0.95256	0.634174	0.778022	0.648104	-0.1304	-0.06384	-0.99853
-0.45581	-0.8663	-0.71442	-0.55235	-0.79432	-0.78677	0.111245	-0.10914	0.692561
-0.72149	-0.87106	-0.62337	-0.54921	-0.80549	-0.80637	0.105862	-0.06165	0.924698
-0.76843	-0.87381	-0.55779	-0.55491	-0.80549	-0.80528	0.104135	-0.05978	0.941004
-0.60469	-0.87271	-0.7102	-0.52568	-0.80238	-0.80033	0.107032	-0.07691	0.836753
-0.685	-0.62582	-0.93204	-0.82039	-0.9467	-0.94285	-0.1214	-0.07839	0.735251
-0.87239	-0.86978	0.511999	-0.54848	-0.8033	-0.80308	0.103723	-0.06067	0.948699
-0.84708	-0.84304	0.634182	-0.51741	-0.78462	-0.78439	0.085229	-0.07151	0.951814
-0.6219	-0.95421	-0.8529	-0.77059	-0.85037	-0.76021	-0.13195	-0.2264	-0.85929
0.941878	-0.99908	-0.96208	0.711012	0.713004	0.672101	-0.1409	-0.07145	-0.99817
0.93106	-0.99908	-0.89943	0.754762	-0.52766	0.762411	-0.14822	-0.07744	-0.99689
-0.83697	-0.90165	-0.94944	-0.61286	-0.86465	0.49945	0.095508	0.214311	-0.8657
0.910891	-0.99945	-0.95146	0.732344	0.580037	0.694816	-0.15066	-0.09825	-0.99798
-0.33433	1	0.86994	0.517831	0.999634	0.467027	0.16245	0.146338	0.991938
-0.78218	-0.71667	0.811138	0.576812	-0.98626	0.847225	-0.10512	0.097951	0.835837
0.627979	-0.99835	-0.9564	-0.92978	-0.99963	-0.99505	-0.10541	-0.16764	-0.90436
0.898056	-0.99963	-0.93497	0.740616	-0.52875	0.705807	-0.15924	-0.11524	-0.9978
-0.97946	-0.89872	-0.94853	-0.59999	-0.85934	-0.85217	0.095621	0.223962	-0.86039
-0.82508	-0.79451	0.744459	-0.53507	-0.75897	-0.75875	0.086383	-0.09652	0.952913
0.56192	-0.79029	-0.88881	0.294223	0.906227	0.660377	0.048562	-0.13709	-0.83877
0.69527	0.927839	0.725591	0.782344	0.920513	0.752519	0.084932	0.226642	0.866435
0.96333	-0.94762	-0.86628	0.293732	-0.92143	-0.93039	-0.15821	-0.14392	-0.81458
-0.8733	-0.51923	-0.56366	-0.6226	0.946337	0.760396	0.06292	-0.09199	0.451081
-0.78786	-0.9033	0.793002	-0.73161	-0.9489	-0.74757	0.14173	-0.0633	0.809454
0.914741	-0.99963	-0.95402	0.452381	-0.99597	-0.39751	-0.24553	-0.08892	-0.9978
0.889989	-0.99963	-0.8987	0.731241	-0.70421	0.718996	-0.17987	-0.10684	-0.99762
-0.27233	0.982234	0.816999	0.89614	0.984432	0.302253	0.037803	-0.09921	0.820447
0.717638	-0.99927	-0.51236	0.869853	-0.97985	0.943579	-0.18833	0.048195	-0.97435
0.861569	-0.99963	-0.77597	0.752565	-0.90476	0.785309	-0.18769	-0.08008	-0.99524
0.774661	-0.99963	-0.59864	0.801646	-0.9685	0.88606	-0.19176	-0.05672	-0.98882
-0.54909	-0.96996	0.944312	-0.75385	-0.99048	-0.89888	0.128249	-0.07623	0.924331
-0.96241	-0.93388	0.636197	0.675	-0.90256	-0.91812	-0.14189	0.149606	-0.524
-0.33671	0.992491	0.849789	0.812684	0.990659	0.425169	0.053845	0.147606	0.931294
0.351249	-0.96648	-0.91683	-0.53731	-0.96374	-0.82689	-0.13359	-0.17288	-0.96739
0.355702	-0.99817	-0.91958	-0.9421	-0.99945	-0.99286	-0.13587	-0.23166	-0.90967
-0.48565	1	0.945961	0.851654	0.999451	0.48388	0.151192	0.133345	0.987175
-0.78236	0.678755	0.506503	-0.56082	0.942308	0.600843	0.046928	-0.09505	0.844998

-0.34947	-0.95989	-0.77487	-0.72168	-0.91374	-0.89339	-0.21218	-0.25874	-0.83034
-0.97488	-0.5152	-0.88295	-0.38161	0.836081	-0.56567	0.211741	0.401475	-0.47783
0.623946	-0.94139	-0.464	0.660093	-0.84725	-0.80528	-0.1477	0.107836	-0.73873
-0.31256	-0.98846	-0.79264	-0.82168	-0.99414	-0.98406	-0.03141	-0.17462	0.542323
-0.6663	0.999634	0.970874	0.745947	0.998901	0.976186	0.296448	0.158614	0.986625
0.257792	-0.99725	-0.89394	-0.9136	-0.99762	-0.98186	-0.16645	-0.21629	-0.87596
-0.51907	0.998901	0.929474	0.849449	0.999267	0.696281	0.125271	0.19698	0.98406
-0.4741	0.999267	0.939733	0.904228	0.999634	0.711302	0.145353	0.138873	0.975449
-0.92171	0.821245	0.588753	0.317271	0.772527	0.882213	0.127045	0.107486	-0.37889
0.858269	-0.97326	-0.92618	-0.45091	-0.97033	-0.97912	-0.19006	-0.22281	-0.96336
-0.92134	0.909158	0.803444	0.428834	0.953114	0.467485	0.12773	0.104361	0.4476
0.656897	-0.89139	0.645539	0.71636	-0.88004	-0.91885	-0.12405	0.153285	-0.47765
0.568731	-0.88828	0.618611	0.766903	-0.9	-0.91683	-0.11595	0.153335	-0.55881
0.930143	0.905861	0.744459	0.753668	0.912088	0.441106	-0.03456	0.103132	0.889703
-0.92134	0.844322	0.655065	0.324939	0.847436	0.420132	0.131183	0.1058	0.285941
-0.92153	0.900733	0.358857	0.344643	-0.64872	0.784759	0.133597	0.123908	0.323501
-0.92079	0.558242	0.763327	0.298853	0.929853	0.676131	0.099922	0.100616	0.610663
0.924826	-0.97253	-0.93332	-0.37812	-0.96245	-0.98498	-0.23378	-0.21231	-0.87889
-0.95966	1	0.976552	0.790257	0.999634	0.989742	0.32853	0.323273	0.990473
-0.91933	0.942674	-0.65891	0.300551	0.862088	0.501374	0.113431	0.095187	0.383657
-0.90117	0.999817	0.965745	0.574055	0.998535	0.981498	0.355254	0.214581	0.992122
-0.92299	0.999634	0.979667	0.652206	0.999817	0.980216	0.31477	0.261611	0.992488
-0.91199	0.867399	0.657446	0.370763	0.617766	0.383862	0.093881	0.08711	0.411995
-0.91144	0.999817	0.960432	0.588769	0.998718	0.914453	0.30541	0.159384	0.990656
-0.89824	0.997436	0.946144	-0.27755	0.998352	0.924528	0.24523	0.201712	0.997618
-0.86854	-0.62967	0.838798	0.143365	0.927656	-0.32277	0.097493	0.126375	0.633199
-0.93968	0.999634	0.974171	0.591176	0.999817	0.978751	0.282515	0.315203	0.994503
-0.95508	0.999267	0.968126	0.512316	0.999451	0.986994	0.306072	0.324186	0.994687
-0.67727	0.998352	0.993039	0.630882	0.999267	0.633816	0.170346	0.163131	0.96299
0.587249	-0.6674	0.657813	0.678107	-0.56245	-0.87214	-0.14968	0.132764	0.466593

RC36	RC37	RC38	RC39	RC40	RC41	RC42
-0.15056	-0.99194	-0.99963	-0.30273	-0.59306	0.996519	-1
0.210701	0.981129	0.999817	-0.3184	0.305867	-0.98736	1
0.270278	0.993404	0.999451	0.215276	0.481832	-0.99927	1
0.131112	0.438073	-0.91758	0.197871	-0.57403	0.55753	-0.90275
0.276817	-0.96867	-0.93315	-0.70699	-0.58497	0.49945	-0.66227
0.152251	0.99542	0.999267	0.287232	-0.23663	-0.99011	1
0.291486	-0.9542	-0.94322	-0.63996	-0.5192	0.528399	-0.68773
0.05976	0.81092	0.638828	0.295878	-0.51028	-0.51099	0.565018
-0.06183	0.886588	0.819231	0.249164	-0.56807	-0.62367	0.74652
-0.09184	0.99542	0.999084	0.319522	-0.34034	-0.95749	0.999817
0.095048	0.995053	0.999451	0.289694	-0.37726	-0.96079	0.999817
0.114938	-0.7893	0.982967	-0.55973	-0.76502	0.470319	0.912821
0.225934	-0.96537	-0.91172	-0.72457	-0.66011	0.534078	-0.6348
-0.13923	0.938073	0.944505	-0.15077	-0.75852	0.440027	0.930403
-0.13923	0.938073	0.944505	-0.15077	-0.75852	0.440027	0.930403
0.205085	-0.9793	-0.95293	-0.54619	0.455447	0.596128	-0.79231
0.134039	0.973983	0.989377	0.257353	0.399157	-0.72792	0.994322
0.134039	0.892451	0.994505	0.257353	0.399157	-0.72792	0.993223
0.195798	-0.95548	-0.89597	-0.73003	-0.67775	0.585379	-0.64725
0.340892	-0.893	-0.73132	-0.64722	-0.33989	-0.6561	0.994505
-0.11228	0.992305	0.999084	0.185974	-0.67242	-0.73049	0.999451
0.287347	-0.952	-0.75476	-0.69437	-0.43009	-0.64786	0.991392
-0.12131	0.996152	0.999267	0.406171	-0.31932	-0.9672	0.998901
0.24753	-0.95566	-0.76593	-0.70943	-0.56127	-0.61891	0.985897
0.182748	0.992488	0.999084	0.335503	-0.49831	-0.93459	1
-0.13483	0.994687	0.999817	0.384716	-0.35522	-0.95493	0.998352
0.173592	0.994137	0.999084	0.249217	0.514361	-0.7816	1
0.14101	0.995603	0.999084	0.191247	-0.50216	-0.93459	1
0.14101	0.993954	0.999084	0.226813	-0.50216	-0.93459	1
-0.14586	0.949249	0.915568	0.352304	-0.50745	-0.61982	0.795238
0.207632	-0.94943	-0.78187	-0.71703	-0.58313	-0.6114	0.96044
-0.0881	0.647307	-0.88846	0.645483	0.644691	-0.32429	-0.70696
0.305707	-0.95694	-0.95842	-0.5872	-0.34151	0.604984	-0.77784
0.150269	0.991572	0.998718	0.335188	-0.44723	-0.93368	1
-0.18242	0.841883	-0.94908	0.746114	0.693378	0.390619	-0.72234
-0.17588	0.996152	0.999817	0.577548	-0.17288	-0.91554	0.998901
-0.16803	0.682118	-0.94908	0.485692	0.528212	-0.30848	-0.52711
-0.1318	0.799377	-0.94908	0.684233	0.69742	0.273299	-0.68681
-0.15671	0.99487	1	0.52838	-0.21661	-0.92488	0.998535
-0.15137	0.994687	0.999817	0.462386	-0.27842	-0.95163	0.998168
-0.17888	0.966288	0.947436	0.453025	-0.42478	-0.62935	0.857875
-0.13112	0.749176	-0.94908	0.608892	0.576531	0.21638	-0.61355
-0.10048	0.641627	-0.53132	-0.21241	-0.63774	0.979113	-1
0.134039	0.991572	0.987363	0.30273	0.508839	-0.72371	0.989377
-0.13911	0.996519	0.999817	0.50875	-0.2154	-0.95511	0.997985
-0.16193	0.854892	-0.94908	0.783317	0.686213	0.505313	-0.81319
0.196043	0.991022	0.998718	0.400055	0.249689	-0.94265	1
0.25468	0.992488	0.998901	0.394149	0.386011	-0.77794	1

-0.15409	0.973983	0.964469	0.54596	-0.33244	-0.61946	0.905678
0.353913	-0.84591	-0.66557	-0.57852	0.268234	-0.73488	0.993223
0.17492	-0.95676	-0.85659	-0.73546	-0.70292	0.373947	-0.64744
0.063698	0.633199	-0.53828	-0.2212	-0.64086	0.970502	-1
0.198983	0.995603	0.992674	0.242906	0.513065	-0.67241	0.993223
0.237552	0.827409	-0.94908	0.786869	0.682538	0.570722	-0.86923
-0.32998	-0.64987	-0.75421	-0.40722	-0.40225	-0.377	0.644689
0.160788	0.99487	0.991209	0.278188	0.628993	-0.66874	0.98956
0.181026	-0.95163	-0.73095	-0.72985	-0.63292	-0.61103	0.9
0.15283	0.993771	0.989377	0.316191	0.631382	-0.68139	0.988645
-0.11136	0.55185	-0.80201	0.293261	0.456295	-0.50104	0.986081
-0.23479	-0.96812	0.785531	-0.56912	-0.63686	0.712349	-0.97711
-0.25462	-0.96702	0.576374	-0.53341	-0.62999	0.748076	-0.99505
-0.21565	-0.96537	-0.59451	-0.48093	-0.62375	0.777391	-0.99908
0.117976	-0.9269	-0.79799	0.300887	0.805183	-0.71345	-0.94524
0.161799	-0.92616	-0.81886	0.291346	0.719681	-0.72902	-0.89212
0.179464	-0.92745	-0.82033	0.294157	0.657766	-0.72169	-0.87527
0.136495	-0.92653	-0.81227	0.285177	0.750546	-0.72756	-0.93187
0.2266	0.807805	-0.94908	0.797899	0.677394	0.517589	-0.88645
0.175283	-0.92818	-0.81538	0.30373	0.643507	-0.7129	-0.85769
0.216068	-0.9203	-0.79267	0.336737	0.624002	-0.69751	-0.81813
0.200702	-0.96134	-0.97125	-0.49003	-0.32298	0.487541	-0.88626
-0.1608	-0.96372	-0.77106	-0.45708	-0.61989	0.801759	-1
-0.14247	-0.96775	-0.71227	-0.47634	-0.63165	0.834555	-1
-0.23052	-0.57842	-0.71245	-0.6847	-0.4876	-0.98461	-0.8881
-0.09708	-0.96299	-0.84414	-0.43292	-0.61199	0.819348	-1
-0.26813	0.603335	0.999267	-0.31271	-0.57539	-0.76896	0.999451
0.201176	-0.53719	-0.75073	0.483874	-0.45593	0.423415	-0.98077
-0.09979	-0.99285	-0.99945	0.274849	0.593984	0.61103	-0.99982
-0.11171	-0.96244	-0.84945	-0.42081	-0.60464	0.827593	-1
-0.22054	-0.56248	-0.70183	-0.67479	-0.46757	-0.98406	-0.8837
0.245434	-0.91682	-0.76355	0.371877	0.660563	-0.66581	-0.77582
0.301249	-0.67552	0.827289	-0.39278	-0.37223	0.276231	0.834982
-0.25216	0.971601	0.967033	0.541338	-0.26309	-0.76127	0.825092
0.308946	-0.43826	-0.95769	-0.17498	0.284157	0.940821	-0.95659
0.246726	-0.80267	-0.6293	-0.5394	0.397391	-0.83071	0.95696
0.273717	-0.95236	-0.9652	-0.56533	0.556311	-0.80945	0.508608
-0.19255	-0.95126	-0.9978	-0.30117	-0.66206	0.959692	-1
-0.0972	-0.96281	-0.84945	-0.41034	-0.60152	0.826127	-1
-0.17985	0.978197	0.987729	0.535865	-0.40448	-0.62404	0.955311
0.095881	-0.96775	-0.57234	-0.45442	-0.6822	0.812752	-1
-0.08563	-0.96189	-0.84267	-0.4088	-0.60023	0.82631	-1
0.08597	-0.96391	-0.77619	-0.42675	-0.65464	0.835654	-1
0.198967	-0.94192	-0.95842	-0.31224	0.812971	-0.85288	-0.92802
-0.0988	-0.64358	0.713187	0.532776	0.304173	0.405826	-0.8978
-0.26644	0.979113	0.995055	0.239559	-0.78986	-0.90876	0.991941
0.308658	-0.88384	-0.95073	-0.4732	0.191275	0.664834	-0.97509
0.097709	-0.99157	-0.99872	-0.13627	0.444506	0.51649	-0.99927
-0.19166	0.991572	0.999817	0.556281	-0.28345	-0.89648	0.999267
0.170552	-0.58666	0.703663	-0.37823	0.592984	-0.85947	0.992674

0.155231	-0.94247	-0.96447	-0.32449	0.489252	0.578417	-0.90366
-0.2073	0.964089	0.998352	-0.50278	-0.32978	-0.98223	0.582418
0.189242	0.535361	-0.99322	-0.11809	-0.49933	0.764566	-0.92289
0.096957	-0.98571	-0.98718	0.301962	0.364219	-0.65079	-0.98498
0.31326	0.988091	0.999267	0.311704	0.655449	-0.77849	1
0.109737	-0.99047	-0.99634	-0.23771	0.408833	0.663979	-0.99853
-0.25944	0.992122	0.999634	0.261761	-0.76635	-0.92873	0.999817
-0.20001	0.994137	1	0.533382	-0.316	-0.88182	0.999084
-0.06614	-0.7435	0.555311	-0.29044	-0.2961	-0.93313	0.944322
0.317395	-0.84591	-0.97711	-0.38481	0.290046	0.997985	-0.98626
-0.08758	-0.50055	0.904029	-0.2737	-0.28393	-0.93276	0.938278
-0.11003	0.572371	0.556227	0.429888	-0.41999	0.425064	-0.78022
-0.07275	0.619274	-0.74652	0.340332	-0.63138	0.316905	-0.82363
-0.22499	0.971418	0.963004	0.660581	0.235726	-0.62422	0.817949
-0.12543	-0.39831	0.888828	-0.27045	-0.3479	-0.93276	0.967399
-0.12237	-0.68487	0.579853	-0.28408	-0.21533	-0.93294	0.986813
-0.07811	-0.26341	0.915934	-0.25138	-0.27728	-0.93258	0.985348
0.28493	-0.72591	-0.9837	-0.25156	0.396003	0.968487	-0.98846
0.189681	0.992488	0.998718	0.323301	0.545767	-0.99743	0.999817
-0.1338	0.243435	0.800183	-0.21721	-0.19614	-0.93129	0.975092
0.217297	0.982594	0.999267	0.289445	0.484475	-0.97985	1
0.167431	0.990656	0.999267	0.389547	0.718354	-0.99505	1
-0.09594	0.380115	0.918864	-0.19674	-0.19762	-0.92653	0.973993
0.183083	0.976365	0.999451	0.247323	0.550544	-0.99212	1
0.16264	0.965555	0.999817	0.26841	0.724529	-0.98791	1
-0.13966	0.558446	-0.7652	-0.17762	-0.2861	-0.89154	0.968498
0.163351	0.989373	0.999267	0.35968	0.694654	-0.99634	1
0.172925	0.987908	0.999817	0.246575	0.578174	-0.99524	1
-0.16497	0.966471	0.958242	0.784342	0.797906	-0.99487	0.898901
0.373077	0.972517	-0.58407	0.150874	0.344979	0.528765	-0.52015
