# Supplementary material for: Comparing structural and transcriptional drug networks reveals signatures of drug activity and toxicity in transcriptional responses
Source: NPJ Syst Biol Appl. 2017 Aug 25;3:23. doi: 10.1038/s41540-017-0022-3 (PMC5572457; doi:10.1038/s41540-017-0022-3)
Supplement: Supplementary file 4 — Supplementary Table 2 [file 41540_2017_22_MOESM4_ESM.pdf]

Drug Name	TV score
5155877	0.71
5182598	0.46
5194442	0.73
5211181	0.77
5224221	0.40
5230742	0.91
5248896	0.74
5252917	0.70
5253409	0.81
5255229	0.93
5279552	0.70
5707885	0.77
(_)_atenolol	0.98
(_)_isoprenaline	0.79
(_)_mk_801	0.88
(+)_chelidonine	0.98
(+)_isoprenaline	0.87
(+/_)_catechin	1.06
0173570_0000	0.68
0175029_0000	0.66
0179445_0000	0.81
0198306_0000	0.90
0297417_0002b	0.65
0316684_0000	0.72
0317956_0000	0.74
10_methoxyharmalan	0.97
11_deoxy_16,16_dimethylprostaglandin_e2	0.91
15(s)_15_methylprostaglandin_e2	1.01
15_delta_prostaglandin_j2	0.54
16_phenyltetranorprostaglandin_e2	0.99
2,6_dimethylpiperidine	0.93
2_aminobenzenesulfonamide	1.00
3_acetamidocoumarin	0.95
3_acetylcoumarin	0.80
3_hydroxy_dl_kynurenine	0.97
3_nitropropionic_acid	0.89
4_hydroxyphenazone	0.99
6_azathymine	0.99
6_benzylaminopurine	0.99
6_bromoindirubin_3'_oxime	0.76
7_aminocephalosporanic_acid	1.27
8_azaguanine	0.76
abamectin	0.85
acacetin	1.08
acebutolol	1.09
aceclofenac	1.08
acemetacin	1.10
acenocoumarol	1.19

acepromazine	0.90
acetazolamide	0.81
acetohexamide	1.04
acetylsalicylic_acid	0.87
acetylsalicylsalicylic_acid	0.80
aciclovir	0.89
aconitine	1.03
adenosine_phosphate	0.98
adiphenine	1.00
adipiodone	0.81
adrenosterone	0.90
ag_013608	0.93
ah_23848	1.22
ajmaline	1.08
albendazole	0.74
alclometasone	1.00
alcuronium_chloride	0.78
alfaxalone	0.93
alfuzosin	0.81
alimemazine	0.93
allantoin	0.81
alpha_ergocryptine	0.88
alpha_estradiol	0.88
alpha_yohimbine	1.16
alprenolol	0.82
alprostadil	1.02
altizide	0.83
altretamine	0.82
alvespimycin	0.53
amantadine	0.89
ambroxol	0.99
amikacin	0.98
amiloride	0.79
aminoglutethimide	0.77
aminohippuric_acid	0.92
aminophenazone	0.83
aminophylline	1.11
amiodarone	0.77
amiprilose	0.88
amitriptyline	0.95
amodiaquine	0.90
amoxapine	0.81
amoxicillin	0.91
amphotericin_b	0.98
ampicillin	1.10
amprolium	0.77
ampyrone	1.00
amrinone	0.80
amylocaine	1.02
anabasine	1.17

androsterone	0.84
anisomycin	0.24
antazoline	0.81
antimycin_a	0.80
apigenin	0.50
apomorphine	0.98
apramycin	1.00
arachidonic_acid	1.02
arachidonyltrifluoromethane	0.80
arcaine	0.89
arecoline	0.97
ascorbic_acid	0.76
asiaticoside	0.87
astemizole	0.48
atractyloside	0.96
atracurium_besilate	0.86
atropine	0.81
atropine_oxide	0.80
azacitidine	0.39
azacyclonol	0.74
azaperone	1.01
azathioprine	0.94
azlocillin	0.99
aztreonam	0.90
bacampicillin	1.08
bacitracin	1.02
baclofen	0.97
bambuterol	1.00
bas_012416453	0.92
bcb000038	0.82
bcb000040	1.11
bemegride	1.07
bendroflumethiazide	0.96
benfluorex	0.72
benfotiamine	0.98
benperidol	0.83
benserazide	1.07
benzamil	0.91
benzathine_benzylpenicillin	1.07
benzethonium_chloride	0.77
benzocaine	0.95
benzonatate	1.00
benzthiazide	0.73
benzydamine	0.90
benzylpenicillin	0.90
bephenium_hydroxynaphthoate	0.89
bepridil	0.75
berberine	0.78
bergenin	0.81
beta_escin	0.89

betahistine	0.79
betaxolol	0.81
betazole	0.99
bethanechol	0.89
betonicine	1.10
betulin	0.91
betulinic_acid	1.01
bezafibrate	0.83
bicuculline	0.77
biperiden	0.89
bisacodyl	0.41
bisoprolol	0.88
blebbistatin	0.74
boldine	1.00
bretylium_tosilate	1.10
brinzolamide	1.11
bromocriptine	0.82
bromopride	0.90
bromperidol	0.78
brompheniramine	0.98
bucladesine	1.04
budesonide	0.87
bufexamac	0.82
buflomedil	0.77
bumetanide	1.07
bupivacaine	1.19
bupropion	0.99
buspirone	0.92
butacaine	0.90
butamben	0.93
butirosin	1.09
butoconazole	0.92
butyl_hydroxybenzoate	0.77
c_75	0.85
calcium_folinate	0.81
calcium_pantothenate	0.79
calmidazolium	0.64
calycanthine	0.99
camptothecin	0.27
canadine	1.07
canrenoic_acid	0.82
capsaicin	0.82
captopril	1.17
carbachol	0.83
carbamazepine	0.91
carbarsone	0.93
carbenoxolone	1.02
carbimazole	0.81
carbinoxamine	1.02
carcinine	1.12

carisoprodol	0.94
carmustine	1.01
carteolol	1.03
cefaclor	0.81
cefadroxil	1.11
cefalexin	0.81
cefalotin	0.80
cefamandole	0.80
cefapirin	0.79
cefazolin	1.02
cefepime	1.09
cefixime	0.99
cefmetazole	0.98
ceforanide	0.81
cefotaxime	0.91
cefotiam	0.96
cefoxitin	0.79
cefsulodin	0.78
cefuroxime	0.90
celecoxib	1.01
cephaeline	0.18
cetirizine	0.77
chenodeoxycholic_acid	1.07
chicago_sky_blue_6b	0.84
chlorambucil	0.80
chlorcyclizine	0.81
chlorhexidine	0.82
chlormezanone	0.88
chlorogenic_acid	1.00
chloropyramine	0.90
chloropyrazine	1.00
chloroquine	0.91
chlorphenamine	0.93
chlorphenesin	1.09
chlorpromazine	0.90
chlorpropamide	0.91
chlorprothixene	0.84
chlortalidone	1.08
chlortetracycline	1.01
chlorzoxazone	0.87
chrysin	0.67
ciclacillin	0.89
cicloheximide	0.23
ciclopirox	0.42
ciclosporin	0.82
cimetidine	1.12
cinchocaine	0.81
cinchonidine	0.92
cinchonine	0.92
cinnarizine	0.81

cinoxacin	0.80
ciprofibrate	1.11
ciprofloxacin	1.00
cisapride	1.03
citalopram	0.90
citiolone	0.93
clebopride	1.00
clemastine	0.89
clemizole	0.78
clenbuterol	0.90
clidinium_bromide	1.04
clindamycin	0.83
clioquinol	0.77
clobetasol	1.01
clofazimine	0.80
clofibrate	1.08
clofilium_tosylate	0.80
clomifene	0.69
clomipramine	0.76
clonidine	0.97
clopamide	0.89
cloperastine	0.73
clorgiline	0.76
clorsulon	1.00
clotrimazole	0.74
cloxacillin	1.08
clozapine	0.99
co_dergocrine_mesilate	0.90
cobalt_chloride	1.02
colchicine	1.00
colecalciferol	0.91
colforsin	0.70
colistin	0.82
conessine	0.99
convolamine	0.89
copper_sulfate	1.01
coralyne	0.91
corbadrine	0.82
corticosterone	0.80
cortisone	0.78
corynanthine	0.76
cotinine	0.81
cp_319743	0.84
cp_320650_01	0.84
cp_645525_01	0.90
cp_690334_01	0.73
cp_863187	0.90
cp_944629	0.86
crotamiton	0.77
cyanocobalamin	1.02

cyclic_adenosine_monophosphate	0.98
cyclizine	0.84
cyclobenzaprine	1.10
cyclopenthiazide	1.14
cyclopentolate	0.91
cycloserine	0.78
cyproheptadine	0.88
cyproterone	0.76
cytisine	0.89
dacarbazine	0.78
danazol	1.12
dantrolene	1.00
dapsone	0.99
daunorubicin	0.84
debrisoquine	1.00
decamethonium_bromide	1.13
deferoxamine	0.88
dehydrocholic_acid	0.97
delsoline	0.86
demecarium_bromide	1.17
demeclocycline	0.85
denatonium_benzoate	0.80
deptropine	0.81
depudecin	0.80
dequalinium_chloride	0.76
desipramine	0.74
desoxycortone	1.07
dexamethasone	0.96
dexibuprofen	0.99
dexpanthenol	0.91
dextromethorphan	0.98
diazoxide	1.10
diclofenac	0.96
diclofenamide	1.08
dicloxacillin	1.09
dicoumarol	0.95
dicycloverine	0.89
dienestrol	0.73
diethylcarbamazine	1.24
diethylstilbestrol	0.78
difenidol	0.78
diflorasone	0.81
diflunisal	1.17
digitoxigenin	0.23
digoxigenin	0.42
digoxin	0.29
dihydroergocristine	0.92
dihydroergotamine	0.77
dihydrostreptomycin	0.98
dilazep	0.76

diloxanide	0.92
diltiazem	1.00
dimenhydrinate	1.09
dimethadione	0.93
dinoprost	0.88
dinoprostone	0.90
dioxybenzone	1.00
diphemanil_metilsulfate	0.92
diphenhydramine	0.96
diphenylpyraline	1.01
dipivefrine	0.84
diprophylline	1.13
dipyridamole	0.85
dirithromycin	0.88
disopyramide	0.53
disulfiram	0.74
dizocilpine	1.13
dl_alpha_tocopherol	0.84
dl_thiorphan	0.77
dobutamine	0.87
domperidone	0.88
dorzolamide	0.81
dosulepin	0.79
doxazosin	0.77
doxorubicin	0.44
doxycycline	1.09
doxylamine	0.82
drofenine	0.77
droperidol	0.89
dropropizine	1.16
dyclonine	0.73
dydrogesterone	0.85
econazole	0.79
edrophonium_chloride	0.94
eldeline	0.81
ellipticine	0.39
emetine	0.25
enalapril	1.09
enilconazole	0.92
enoxacin	0.78
epiandrosterone	0.91
epirizole	0.98
epitiostanol	0.76
epivincamine	1.03
equilin	0.78
erastin	0.98
ergocalciferol	1.09
erythromycin	0.87
esculetin	0.99
esculin	0.92

estradiol	0.92
estriol	0.77
estrone	0.99
estropipate	0.83
etamivan	1.01
etamsylate	0.95
etanidazole	0.97
ethambutol	0.90
ethaverine	1.06
ethisterone	0.90
ethotoin	0.89
ethoxyquin	0.92
eticlopride	1.09
etidronic_acid	0.81
etifenin	1.15
etilefrine	1.01
etiocholanolone	1.00
etodolac	0.89
etofenamate	0.88
etofylline	0.93
etomidate	0.91
etoposide	0.60
etynodiol	0.89
eucatropine	0.84
exisulind	1.09
f0447_0125	0.72
famotidine	0.82
famprofazone	0.79
felbinac	0.99
felodipine	0.78
fenbufen	0.92
fendiline	0.67
fenofibrate	0.88
fenoprofen	0.85
fenspiride	0.94
finasteride	1.10
flavoxate	1.05
flecainide	0.86
florfenicol	0.86
flucloxacillin	0.93
flucytosine	0.95
fludrocortisone	0.99
fludroxycortide	0.80
flufenamic_acid	0.92
flumequine	1.02
flumetasone	0.86
flunarizine	0.73
flunisolide	0.83
flunixin	0.97
fluocinonide	0.89

fluorocurarine	0.97
fluorometholone	0.88
fluoxetine	0.88
flupentixol	0.89
fluphenazine	0.80
flurbiprofen	0.92
fluspirilene	0.68
flutamide	1.01
fluticasone	0.97
fluvastatin	0.91
fluvoxamine	0.89
folic_acid	1.06
foliosidine	1.00
fosfosal	0.81
fulvestrant	0.73
furaltadone	0.80
furazolidone	1.00
furosemide	0.93
fursultiamine	0.96
fusaric_acid	0.74
fusidic_acid	1.02
gabapentin	0.93
gabexate	1.01
galantamine	0.78
gallamine_triethiodide	0.97
ganciclovir	1.02
geldanamycin	0.47
gelsemine	0.94
gemfibrozil	0.97
genistein	0.90
gentamicin	1.10
gibberellic_acid	0.97
ginkgolide_a	0.78
glafenine	0.72
glibenclamide	0.88
gliclazide	0.79
glimepiride	0.93
glipizide	0.94
gliquidone	0.90
glycocholic_acid	1.03
glycopyrronium_bromide	0.80
gossypol	0.71
gramine	0.73
griseofulvin	0.81
guaifenesin	0.88
guanabenz	1.15
guanadrel	1.19
guanfacine	0.80
gw_8510	0.31
h_7	0.44

halcinonide	0.88
halofantrine	0.88
haloperidol	0.95
harmaline	0.85
harmalol	0.99
harman	0.85
harmine	0.47
harmol	0.63
harpagoside	0.98
hecogenin	0.77
heliotrine	0.92
helveticoside	0.22
hemicholinium	0.85
heptaminol	0.90
hesperetin	0.88
hesperidin	1.16
hexamethonium_bromide	0.91
hexestrol	0.84
hexetidine	0.72
hexylcaine	1.20
homatropine	1.01
homochlorcyclizine	0.71
homosalate	1.02
hycanthone	0.60
hydralazine	0.81
hydrastine_hydrochloride	0.80
hydrastinine	1.08
hydrochlorothiazide	0.92
hydrocortisone	0.96
hydrocotarnine	0.87
hydroflumethiazide	0.78
hydroquinine	1.09
hydroxyachillin	0.79
hydroxyzine	0.91
hymecromone	0.95
hyoscyamine	0.85
ic_86621	0.89
idazoxan	0.84
idoxuridine	0.78
ifenprodil	0.81
ifosfamide	1.01
ikarugamycin	0.89
iloprost	0.86
imidurea	0.91
imipenem	0.78
imipramine	0.85
indapamide	1.10
indometacin	0.99
indoprofen	0.81
iobenguane	0.94

iocetamic_acid	1.01
iodixanol	1.01
iohexol	0.89
ionomycin	0.59
iopamidol	0.73
iopanoic_acid	0.96
iopromide	0.97
ioversol	0.95
ioxaglic_acid	1.09
iproniazid	0.90
irinotecan	0.24
isocarboxazid	1.06
isoconazole	0.86
isocorydine	1.02
isoetarine	0.87
isometheptene	0.96
isoniazid	1.02
isopropamide_iodide	0.92
isosorbide	0.97
isotretinoin	0.70
isoxicam	0.76
isoxsuprine	0.98
isradipine	1.07
ivermectin	0.68
josamycin	0.99
kaempferol	1.01
kanamycin	0.78
karakoline	1.02
kawain	1.07
ketanserin	0.73
ketoconazole	0.98
ketoprofen	0.93
ketorolac	0.88
ketotifen	1.00
khellin	1.10
kinetin	0.80
l_methionine_sulfoximine	0.93
labetalol	0.92
lactobionic_acid	0.85
lanatoside_c	0.22
lansoprazole	0.88
lasalocid	0.77
laudanosine	0.91
leflunomide	1.08
letrozole	0.97
levamisole	0.87
levcycloserine	0.80
levobunolol	1.01
levocabastine	0.86
levodopa	1.10

levomepromazine	0.78
levonorgestrel	0.75
levopropoxyphene	0.94
levothyroxine_sodium	0.99
lidocaine	1.08
lidoflazine	1.06
lincomycin	0.98
liothyronine	0.77
lisinopril	0.82
lisuride	0.80
lithocholic_acid	0.99
lm_1685	0.93
lobelanidine	0.84
lobeline	1.00
lomefloxacin	0.91
lomustine	0.54
loperamide	0.82
loracarbef	0.89
lorglumide	0.81
lovastatin	0.76
loxapine	0.80
luteolin	0.58
ly_294002	0.73
lycorine	0.83
lymecycline	0.81
lynestrenol	0.86
lysergol	0.90
mafenide	1.02
maprotiline	0.85
mebendazole	0.64
mebeverine	0.99
mebhydrolin	0.89
mecamylamine	0.90
meclocycline	0.87
meclofenamic_acid	1.02
meclofenoxate	0.86
meclozine	0.79
medrysone	0.78
mefenamic_acid	1.01
mefexamide	1.10
mefloquine	0.66
megestrol	0.94
meglumine	1.09
melatonin	0.98
memantine	0.98
mepenzolate_bromide	0.91
mephenesin	0.88
mephentermine	1.00
mephenytoin	1.09
meprylcaine	0.80

meptazinol	0.80
mepyramine	0.79
merbromin	1.09
meropenem	0.89
mesalazine	1.00
mesoridazine	0.90
mestranol	0.90
metacycline	0.94
metamizole_sodium	0.87
metampicillin	0.92
metanephrine	1.02
metaraminol	1.11
meteneprost	1.11
metergoline	0.84
metformin	0.93
methacholine_chloride	0.94
methanthelinium_bromide	0.92
methapyrilene	0.79
methazolamide	0.96
methocarbamol	1.08
methotrexate	0.65
methoxamine	0.92
methoxsalen	0.90
methylbenzethonium_chloride	0.76
methyldopa	0.90
methyldopate	0.92
methylergometrine	0.89
methylprednisolone	0.90
meticrane	0.87
metitepine	0.76
metixene	0.85
metoclopramide	0.93
metolazone	0.96
metoprolol	0.82
metrifonate	0.89
metrizamide	1.19
metronidazole	0.89
metyrapone	0.82
mexiletine	1.01
mianserin	0.91
miconazole	0.77
midecamycin	1.12
midodrine	1.02
mifepristone	0.91
milrinone	0.80
mimosine	1.09
minaprine	0.89
minocycline	0.94
minoxidil	0.90
mitoxantrone	0.46

mk_886	0.91
molindone	0.83
molsidomine	0.79
mometasone	0.92
monastrol	0.85
monensin	0.67
monobenzone	0.68
monocrotaline	0.89
monorden	0.68
moracizine	0.92
morantel	0.77
moroxydine	1.04
moxisylyte	0.78
mycophenolic_acid	0.55
myosmine	0.99
myricetin	0.90
n_acetyl_l_aspartic_acid	0.87
n_acetyl_l_leucine	0.82
n_acetylmuramic_acid	0.90
n6_methyladenosine	0.82
nabumetone	0.98
nadide	0.99
nadolol	0.89
nafcillin	0.80
naftidrofuryl	1.02
naftifine	0.75
nalbuphine	0.85
nalidixic_acid	0.87
naloxone	0.80
naltrexone	1.17
napelline	0.87
naphazoline	1.14
naproxen	0.95
naringenin	0.88
naringin	0.90
natamycin	0.79
nefopam	0.88
neomycin	0.87
neostigmine_bromide	0.95
netilmicin	1.00
nialamide	1.10
nicardipine	0.88
nicergoline	0.88
niclosamide	0.48
nicotinic_acid	1.06
nifedipine	0.98
nifenazone	1.12
niflumic_acid	1.18
nifuroxazide	0.70
nifurtimox	0.79

nilutamide	1.09
nimesulide	0.98
nimodipine	1.09
nipecotic_acid	0.83
niridazole	0.98
nisoxetine	0.80
nitrendipine	0.98
nitrofural	1.05
nitrofurantoin	0.82
nizatidine	0.81
nocodazole	0.81
nomegestrol	0.99
nomifensine	0.88
norcyclobenzaprine	0.75
nordihydroguaiaretic_acid	0.90
norethisterone	0.80
noretynodrel	0.78
norfloxacin	0.80
nortriptyline	0.77
noscapine	0.91
novobiocin	1.00
ns_398	1.05
nu_1025	0.82
octopamine	0.82
ofloxacin	0.82
oleandomycin	0.81
omeprazole	0.74
ondansetron	1.06
orciprenaline	0.93
orlistat	1.03
ornidazole	0.90
orphenadrine	1.03
ouabain	0.34
oxamniquine	0.89
oxantel	0.83
oxaprozin	0.93
oxedrine	0.92
oxetacaine	0.88
oxolamine	0.75
oxolinic_acid	0.83
oxprenolol	0.92
oxybenzone	0.82
oxybuprocaine	0.87
oxybutynin	0.91
oxyphenbutazone	0.72
ozagrel	0.80
paclitaxel	0.91
palmatine	1.04
pancuronium_bromide	0.85
papaverine	0.80

paracetamol	0.86
parbendazole	0.71
pargyline	0.89
paromomycin	1.03
paroxetine	0.91
parthenolide	0.36
pempidine	0.93
penbutolol	0.90
pentamidine	0.89
pentetic_acid	0.79
pentetrazol	1.00
pentolonium	0.88
pentoxifylline	0.87
pentoxyverine	0.80
pepstatin	1.00
pergolide	0.88
perhexiline	0.61
perphenazine	0.74
pf_00562151_00	0.91
pf_00875133_00	0.89
pf_01378883_00	0.80
pha_00745360	0.86
pha_00767505e	0.64
pha_00851261e	0.84
phenacetin	1.02
phenazopyridine	0.93
phenelzine	0.89
pheneticillin	1.09
phenformin	1.00
phenindione	0.80
pheniramine	1.09
phenoxybenzamine	0.39
phensuximide	0.82
phentolamine	1.03
phenylpropanolamine	0.98
phthalylsulfathiazole	0.75
physostigmine	0.92
picotamide	1.02
picrotoxinin	0.91
pilocarpine	0.95
pimethixene	0.73
pimozide	0.66
pinacidil	1.01
pindolol	0.80
pioglitazone	0.99
pipenzolate_bromide	0.80
piperacetazine	0.80
piperacillin	1.01
piperidolate	0.81
piperine	1.01

piracetam	0.85
pirenperone	0.80
pirenzepine	0.89
piretanide	0.90
piribedil	1.00
pirinixic_acid	1.23
pirlindole	0.95
piromidic_acid	0.76
piroxicam	0.88
pivampicillin	0.86
pivmecillinam	0.88
pizotifen	1.01
pnu_0230031	0.79
pnu_0251126	0.60
podophyllotoxin	0.85
practolol	0.81
pralidoxime	0.82
pramocaine	0.89
prasterone	0.92
praziquantel	0.81
prazosin	0.95
prednicarbate	1.00
prednisolone	1.00
prednisone	0.90
pregnenolone	0.79
prenylamine	0.58
prestwick_1080	0.79
prestwick_1082	0.86
prestwick_1084	0.74
prestwick_1085	0.88
prestwick_1100	0.83
prestwick_1103	0.89
prestwick_559	0.76
prestwick_642	0.99
prestwick_664	0.81
prestwick_665	1.13
prestwick_674	0.90
prestwick_682	0.91
prestwick_685	1.01
prestwick_689	1.09
prestwick_691	0.96
prestwick_692	1.08
prestwick_857	1.04
prestwick_860	0.80
prestwick_864	0.77
prestwick_920	0.80
prestwick_967	1.00
prestwick_981	0.83
prestwick_983	1.00
prestwick_984	0.80

pridinol	0.81
prilocaine	0.93
primaquine	0.65
primidone	0.94
proadifen	0.71
probenecid	1.02
probucol	0.96
procainamide	0.81
procaine	0.93
procarbazine	0.93
prochlorperazine	0.75
procyclidine	0.97
profenamine	0.90
progesterone	0.90
proglumide	1.05
proguanil	0.80
promazine	0.91
promethazine	0.82
pronetalol	0.86
propafenone	0.88
propantheline_bromide	0.94
propidium_iodide	0.86
propofol	0.84
propoxycaine	0.89
propranolol	0.95
propylthiouracil	0.88
proscillaridin	0.18
protoveratrine_a	0.73
protriptyline	0.76
proxymetacaine	0.99
proxyphylline	0.93
pseudopelletierine	1.22
puromycin	0.36
pyrantel	0.99
pyrazinamide	0.81
pyridoxine	0.78
pyrimethamine	0.82
pyrithyldione	0.91
pyrvinium	0.51
quercetin	1.04
quinethazone	0.92
quinidine	0.91
quinisocaine	0.78
quinostatin	0.61
quinpirole	1.00
quipazine	1.18
r_atenolol	0.88
racecadotril	0.96
raloxifene	0.91
ramifenazone	1.02

ramipril	0.83
ranitidine	0.88
raubasine	0.80
remoxipride	0.90
repaglinide	0.80
rescinnamine	0.71
resveratrol	0.66
retrorsine	0.85
ribavirin	0.82
riboflavin	0.81
ribostamycin	1.03
ricinine	0.85
rifabutin	0.33
rifampicin	0.95
rilmenidine	0.99
riluzole	0.87
rimexolone	0.80
risperidone	1.12
ritodrine	0.82
rofecoxib	0.99
rolipram	0.86
rolitetracycline	0.93
rosiglitazone	0.99
rotenone	0.91
rottlerin	0.66
roxarsone	0.82
roxithromycin	0.79
s_propranolol	1.10
salbutamol	0.92
salsolidin	0.78
salsolinol	0.98
santonin	0.93
saquinavir	0.86
sb_202190	1.00
sb_203580	0.98
sc_19220	1.08
sc_560	0.76
sc_58125	1.00
scopolamine	0.81
scopolamine_n_oxide	1.00
scoulerine	0.80
securinine	0.71
selegiline	0.87
semustine	0.75
seneciphylline	0.70
serotonin	0.90
sertaconazole	0.80
simvastatin	0.79
sirolimus	0.71
sisomicin	0.86

sitosterol	0.88
skimmianine	0.95
sodium_phenylbutyrate	0.80
solanine	0.95
solasodine	0.91
sotalol	1.03
sparteine	0.89
spectinomycin	1.03
spiradoline	1.02
spiramycin	0.90
spironolactone	0.87
sr_95531	0.98
sr_95639a	0.90
stachydrine	1.08
staurosporine	0.77
stock1n_28457	0.71
streptomycin	0.92
streptozocin	0.95
strophanthidin	1.09
succinylsulfathiazole	1.13
sulconazole	0.70
sulfabenzamide	1.02
sulfachlorpyridazine	0.82
sulfadiazine	1.03
sulfadimethoxine	0.93
sulfadimidine	1.00
sulfadoxine	0.82
sulfafurazole	0.81
sulfaguanidine	0.82
sulfamerazine	0.80
sulfamethizole	1.07
sulfamethoxazole	1.06
sulfamethoxypyridazine	0.94
sulfametoxydiazine	0.91
sulfamonomethoxine	0.98
sulfanilamide	1.01
sulfaphenazole	1.12
sulfapyridine	0.91
sulfaquinoxaline	0.82
sulfasalazine	0.93
sulfathiazole	0.98
sulfinpyrazone	0.90
sulindac	0.97
suloctidil	0.57
sulpiride	0.91
suprofen	1.02
suramin_sodium	0.91
suxibuzone	0.99
syrosingopine	0.54
tacrine	0.88

tacrolimus	0.83
talampicillin	0.88
tamoxifen	0.92
tanespimycin	0.50
telenzepine	0.97
tenoxicam	0.97
terazosin	0.93
terbutaline	0.96
terconazole	0.85
terfenadine	0.47
terguride	0.83
testosterone	0.83
tetracaine	1.01
tetracycline	1.09
tetraethylenepentamine	0.92
tetrahydroalstonine	1.06
tetramisole	1.01
tetrandrine	0.72
tetroquinone	0.75
tetryzoline	1.11
thalidomide	1.00
thapsigargin	0.33
theobromine	0.89
theophylline	0.82
thiamazole	1.09
thiamine	1.02
thiamphenicol	1.10
thiethylperazine	0.90
thiocolchicoside	0.85
thioguanosine	0.47
thioperamide	0.78
thioproperazine	0.89
thioridazine	0.70
thiostrepton	0.58
tiabendazole	0.96
tiapride	0.83
tiaprofenic_acid	0.99
ticlopidine	0.78
tiletamine	1.00
timolol	1.04
tinidazole	1.01
tiratricol	0.86
tobramycin	0.78
tocainide	1.12
todralazine	0.90
tolazoline	1.05
tolbutamide	1.00
tolmetin	1.01
tolnaftate	0.79
tomatidine	0.75

tonzonium_bromide	0.77
torasemide	0.82
tracazolate	0.76
tranexamic_acid	0.98
tranylcypromine	0.80
trapidil	0.88
trazodone	0.77
tremorine	0.98
tretinoin	0.60
triamcinolone	0.89
triamterene	1.03
tribenoside	0.91
trichlormethiazide	0.80
trichostatin_a	0.21
tridihexethyl	0.99
trifluoperazine	0.78
triflupromazine	0.80
trifluridine	0.69
triflusal	0.79
trimetazidine	0.83
trimethadione	1.03
trimethobenzamide	0.93
trimethoprim	0.87
trimethylcolchicinic_acid	0.80
trimipramine	0.77
trioxysalen	0.96
triprolidine	0.78
troglitazone	0.88
trolox_c	0.81
tropicamide	0.85
tropine	1.13
tubocurarine_chloride	0.89
tyloxapol	0.91
urapidil	1.00
ursolic_acid	0.82
valdecoxib	1.02
valinomycin	0.71
valproic_acid	0.88
vancomycin	1.10
vanoxerine	0.75
velnacrine	0.87
verapamil	0.99
verteporfin	0.50
vidarabine	0.82
vinblastine	0.81
vinburnine	0.78
vincamine	0.99
vinpocetine	0.96
viomycin	1.01
vitexin	0.88

vorinostat	0.15
w_13	0.90
withaferin_a	0.49
wortmannin	0.70
xamoterol	1.04
xylazine	0.83
xylometazoline	0.92
y_27632	0.77
yohimbine	1.03
zalcitabine	0.89
zaprinast	1.07
zardaverine	0.90
zidovudine	0.96
zimeldine	0.91
zomepirac	0.79
zoxazolamine	0.81
zuclopenthixol	0.74
