# Supplementary material for: Comparing structural and transcriptional drug networks reveals signatures of drug activity and toxicity in transcriptional responses
Source: NPJ Syst Biol Appl. 2017 Aug 25;3:23. doi: 10.1038/s41540-017-0022-3 (PMC5572457; doi:10.1038/s41540-017-0022-3)
Supplement: Supplementary file 6 — Supplementary Table 4 [file 41540_2017_22_MOESM6_ESM.pdf]

| Drug A | Drug B | MANTRA | STR |
|---|---|---|---|
| digoxin | lanatoside_C | 0.131 | 0.693 |
| digoxin | proscillaridin | 0.166 | 0.758 |
| lanatoside_C | proscillaridin | 0.187 | 0.776 |
| rifabutin | vorinostat | 0.286 | 0.826 |
| astemizole | terfenadine | 0.337 | 0.724 |
| astemizole | mefloquine | 0.385 | 0.776 |
| doxorubicin | mitoxantrone | 0.414 | 0.651 |
| mefloquine | terfenadine | 0.421 | 0.767 |
| chlorzoxazone | clindamycin | 0.442 | 0.829 |
| chlorzoxazone | glibenclamide | 0.445 | 0.791 |
| terfenadine | trifluoperazine | 0.453 | 0.758 |
| irinotecan | phenoxybenzamine | 0.455 | 0.800 |
| suloctidil | terfenadine | 0.466 | 0.696 |
| astemizole | trifluoperazine | 0.469 | 0.718 |
| protriptyline | trifluoperazine | 0.472 | 0.713 |
| niclosamide | trifluoperazine | 0.472 | 0.688 |
| mefloquine | trifluoperazine | 0.478 | 0.674 |
| doxazosin | sulconazole | 0.481 | 0.776 |
| lomustine | phenoxybenzamine | 0.484 | 0.719 |
| clindamycin | glibenclamide | 0.487 | 0.745 |
| niclosamide | terfenadine | 0.488 | 0.737 |
| metergoline | trifluoperazine | 0.489 | 0.712 |
| nortriptyline | trifluoperazine | 0.496 | 0.657 |
| niclosamide | pyrvinium | 0.498 | 0.763 |
| astemizole | niclosamide | 0.502 | 0.758 |
| chlorzoxazone | dirithromycin | 0.503 | 0.908 |
| dirithromycin | glibenclamide | 0.509 | 0.795 |
| daunorubicin | mitoxantrone | 0.510 | 0.656 |
| benzethonium_chloride | trifluoperazine | 0.518 | 0.780 |
| disulfiram | mefloquine | 0.529 | 0.701 |
| astemizole | disulfiram | 0.530 | 0.767 |
| metergoline | terfenadine | 0.530 | 0.716 |
| fluspirilene | trifluoperazine | 0.531 | 0.733 |
| bisacodyl | digoxin | 0.537 | 0.828 |
| clindamycin | dirithromycin | 0.539 | 0.790 |
| digoxin | menadione | 0.540 | 0.875 |
| mefloquine | niclosamide | 0.542 | 0.710 |
| hexetidine | trifluoperazine | 0.543 | 0.735 |
| fluspirilene | terfenadine | 0.559 | 0.727 |
| pyrvinium | trifluoperazine | 0.559 | 0.763 |
| prenylamine | terfenadine | 0.560 | 0.717 |
| lanatoside_C | niclosamide | 0.561 | 0.864 |
| astemizole | perphenazine | 0.561 | 0.725 |
| loperamide | perphenazine | 0.562 | 0.691 |
| perhexiline | trifluoperazine | 0.562 | 0.740 |
| astemizole | suloctidil | 0.562 | 0.729 |
| mefloquine | suloctidil | 0.564 | 0.728 |
| bisacodyl | proscillaridin | 0.565 | 0.762 |
| disulfiram | terfenadine | 0.566 | 0.768 |

| | | | |
|---|---|---|---|
| astemizole | metergoline | 0.567 | 0.707 |
| astemizole | fluspirilene | 0.568 | 0.723 |
| fendiline | terfenadine | 0.569 | 0.726 |
| daunorubicin | irinotecan | 0.572 | 0.700 |
| niclosamide | suloctidil | 0.573 | 0.725 |
| fendiline | niclosamide | 0.575 | 0.725 |
| phenoxybenzamine | semustine | 0.575 | 0.733 |
| niclosamide | phenoxybenzamine | 0.575 | 0.706 |
| bisacodyl | lanatoside_C | 0.579 | 0.859 |
| astemizole | prenylamine | 0.580 | 0.709 |
| ivermectin | trifluoperazine | 0.581 | 0.821 |
| disulfiram | phenoxybenzamine | 0.582 | 0.752 |
| astemizole | mometasone | 0.582 | 0.763 |
| bepridil | niclosamide | 0.583 | 0.764 |
| loperamide | trifluoperazine | 0.583 | 0.708 |
| azacitidine | doxorubicin | 0.584 | 0.730 |
| chlorprothixene | terfenadine | 0.585 | 0.774 |
| metergoline | niclosamide | 0.585 | 0.723 |
| lanatoside_C | menadione | 0.585 | 0.900 |
| reserpine | trichlormethiazide | 0.586 | 0.776 |
| metixene | trifluoperazine | 0.587 | 0.717 |
| astemizole | nortriptyline | 0.587 | 0.749 |
| pyrvinium | suloctidil | 0.587 | 0.786 |
| astemizole | protriptyline | 0.588 | 0.750 |
| menadione | terfenadine | 0.589 | 0.812 |
| bepridil | trifluoperazine | 0.589 | 0.712 |
| protriptyline | terfenadine | 0.589 | 0.763 |
| methotrexate | mycophenolic_acid | 0.590 | 0.710 |
| digoxin | emetine | 0.590 | 0.814 |
| astemizole | fendiline | 0.591 | 0.729 |
| metergoline | protriptyline | 0.592 | 0.733 |
| pyrvinium | terfenadine | 0.592 | 0.797 |
| loperamide | maprotiline | 0.592 | 0.750 |
| mefloquine | perphenazine | 0.593 | 0.753 |
| medrysone | sulconazole | 0.595 | 0.748 |
| ambroxol | moroxydine | 0.595 | 0.739 |
| emetine | lanatoside_C | 0.595 | 0.840 |
| maprotiline | trifluoperazine | 0.595 | 0.726 |
| astemizole | ivermectin | 0.596 | 0.811 |
| prenylamine | suloctidil | 0.596 | 0.668 |
| niclosamide | prenylamine | 0.599 | 0.727 |
| perphenazine | terfenadine | 0.599 | 0.733 |
| loperamide | terfenadine | 0.599 | 0.692 |
| suloctidil | trifluoperazine | 0.599 | 0.731 |
| astemizole | chlorprothixene | 0.600 | 0.733 |
| fluspirilene | loperamide | 0.600 | 0.722 |
| cefalexin | pentoxyverine | 0.601 | 0.687 |
| astemizole | rescinnamine | 0.601 | 0.740 |
| astemizole | bepridil | 0.602 | 0.731 |
| loperamide | niclosamide | 0.604 | 0.732 |

| | | | |
|---|---|---|---|
| niclosamide | perphenazine | 0.604 | 0.714 |
| astemizole | phenoxybenzamine | 0.605 | 0.754 |
| mefloquine | phenoxybenzamine | 0.605 | 0.747 |
| raloxifene | trifluoperazine | 0.605 | 0.693 |
| benzethonium_chloride | niclosamide | 0.605 | 0.762 |
| gefitinib | loperamide | 0.606 | 0.760 |
| gliclazide | thiocolchicoside | 0.608 | 0.746 |
| metixene | perphenazine | 0.608 | 0.699 |
| azacitidine | mitoxantrone | 0.609 | 0.748 |
| astemizole | isoconazole | 0.610 | 0.755 |
| metergoline | perphenazine | 0.610 | 0.705 |
| chlorcyclizine | trifluoperazine | 0.610 | 0.699 |
| loperamide | metergoline | 0.611 | 0.694 |
| fluspirilene | niclosamide | 0.612 | 0.721 |
| fenoprofen | meclofenoxate | 0.612 | 0.707 |
| perhexiline | terfenadine | 0.612 | 0.771 |
| loperamide | protriptyline | 0.612 | 0.753 |
| fluspirilene | metergoline | 0.612 | 0.701 |
| mefloquine | metergoline | 0.613 | 0.746 |
| bepridil | terfenadine | 0.614 | 0.732 |
| maprotiline | metergoline | 0.614 | 0.758 |
| pimozide | trifluoperazine | 0.615 | 0.744 |
| digoxin | niclosamide | 0.615 | 0.849 |
| loperamide | nortriptyline | 0.615 | 0.765 |
| fendiline | suloctidil | 0.615 | 0.751 |
| nortriptyline | terfenadine | 0.615 | 0.771 |
| rifabutin | trifluoperazine | 0.616 | 0.783 |
| emetine | terfenadine | 0.616 | 0.756 |
| ajmaline | dirithromycin | 0.617 | 0.827 |
| cetirizine | omeprazole | 0.617 | 0.734 |
| oxprenolol | repaglinide | 0.618 | 0.726 |
| benzethonium_chloride | mefloquine | 0.618 | 0.788 |
| astemizole | trimipramine | 0.618 | 0.762 |
| astemizole | prochlorperazine | 0.618 | 0.742 |
| mometasone | niclosamide | 0.619 | 0.728 |
| azacitidine | daunorubicin | 0.619 | 0.709 |
| hexetidine | terfenadine | 0.620 | 0.751 |
| disulfiram | lomustine | 0.620 | 0.658 |
| rescinnamine | terfenadine | 0.620 | 0.739 |
| ivermectin | mefloquine | 0.621 | 0.822 |
| etoposide | irinotecan | 0.621 | 0.754 |
| fluvoxamine | trifluoperazine | 0.622 | 0.680 |
| clopamide | glibenclamide | 0.622 | 0.731 |
| menadione | proscillaridin | 0.623 | 0.849 |
| astemizole | pyrvinium | 0.624 | 0.751 |
| colchicine | mebendazole | 0.624 | 0.704 |
| phenazopyridine | trifluoperazine | 0.625 | 0.702 |
| gliclazide | medrysone | 0.625 | 0.742 |
| monobenzone | trifluridine | 0.626 | 0.743 |
| piperidolate | sulconazole | 0.626 | 0.716 |

| | | | |
|---|---|---|---|
| astemizole | hexetidine | 0.627 | 0.743 |
| lanatoside_C | terfenadine | 0.628 | 0.824 |
| menadione | phenoxybenzamine | 0.629 | 0.758 |
| propylthiouracil | tobramycin | 0.629 | 0.816 |
| prenylamine | trifluoperazine | 0.630 | 0.742 |
| hyoscyamine | oxolinic_acid | 0.630 | 0.696 |
| trifluoperazine | trimipramine | 0.631 | 0.669 |
| mefloquine | pyrvinium | 0.631 | 0.811 |
| mefloquine | prenylamine | 0.631 | 0.767 |
| niclosamide | proscillaridin | 0.631 | 0.787 |
| clomipramine | gefitinib | 0.631 | 0.742 |
| loperamide | zuclopenthixol | 0.631 | 0.731 |
| loperamide | metixene | 0.632 | 0.755 |
| phenoxybenzamine | terfenadine | 0.632 | 0.762 |
| doxazosin | medrysone | 0.632 | 0.720 |
| isoconazole | terfenadine | 0.632 | 0.755 |
| astemizole | miconazole | 0.632 | 0.744 |
| ambroxol | trihexyphenidyl | 0.632 | 0.708 |
| mometasone | trifluoperazine | 0.633 | 0.744 |
| astemizole | loperamide | 0.633 | 0.717 |
| pimozide | terfenadine | 0.633 | 0.702 |
| rescinnamine | trifluoperazine | 0.633 | 0.745 |
| chlorprothixene | niclosamide | 0.633 | 0.684 |
| fluspirilene | prenylamine | 0.634 | 0.713 |
| amiodarone | meclozine | 0.634 | 0.733 |
| maprotiline | perphenazine | 0.634 | 0.740 |
| mebendazole | terfenadine | 0.635 | 0.715 |
| chlortetracycline | chlorzoxazone | 0.635 | 0.775 |
| emetine | suloctidil | 0.636 | 0.735 |
| chlorprothixene | metergoline | 0.636 | 0.729 |
| azacitidine | phenoxybenzamine | 0.636 | 0.771 |
| amitriptyline | trifluoperazine | 0.636 | 0.667 |
| mometasone | prenylamine | 0.636 | 0.763 |
| fluspirilene | pyrvinium | 0.636 | 0.773 |
| clotrimazole | pimozide | 0.636 | 0.769 |
| ketanserin | practolol | 0.637 | 0.684 |
| mometasone | terfenadine | 0.637 | 0.747 |
| fendiline | mefloquine | 0.637 | 0.762 |
| galantamine | trichlormethiazide | 0.637 | 0.723 |
| mefloquine | perhexiline | 0.638 | 0.743 |
| fluspirilene | suloctidil | 0.639 | 0.718 |
| metergoline | pyrvinium | 0.639 | 0.750 |
| phenoxybenzamine | trifluoperazine | 0.639 | 0.739 |
| metergoline | suloctidil | 0.640 | 0.689 |
| carbachol | doxazosin | 0.640 | 0.819 |
| fluspirilene | mefloquine | 0.641 | 0.742 |
| chlorprothixene | suloctidil | 0.641 | 0.760 |
| doxazosin | gliclazide | 0.641 | 0.692 |
| medrysone | tropicamide | 0.641 | 0.706 |
| econazole | trifluoperazine | 0.642 | 0.704 |

| | | | |
|---|---|---|---|
| gefitinib | pimozide | 0.642 | 0.744 |
| promethazine | sulconazole | 0.642 | 0.708 |
| disulfiram | niclosamide | 0.642 | 0.697 |
| hexetidine | mefloquine | 0.643 | 0.769 |
| hexetidine | niclosamide | 0.643 | 0.763 |
| fendiline | trifluoperazine | 0.643 | 0.742 |
| astemizole | menadione | 0.644 | 0.804 |
| syrosingopine | trifluoperazine | 0.644 | 0.776 |
| emetine | menadione | 0.644 | 0.795 |
| astemizole | fluphenazine | 0.644 | 0.729 |
| digoxin | terfenadine | 0.644 | 0.798 |
| betahistine | omeprazole | 0.645 | 0.703 |
| cetirizine | sulconazole | 0.645 | 0.705 |
| astemizole | flupentixol | 0.645 | 0.737 |
| benzethonium_chloride | fendiline | 0.646 | 0.712 |
| disulfiram | trifluoperazine | 0.646 | 0.744 |
| miconazole | terfenadine | 0.646 | 0.763 |
| colchicine | methylergometrine | 0.646 | 0.700 |
| proscillaridin | terfenadine | 0.647 | 0.754 |
| estriol | repaglinide | 0.647 | 0.763 |
| ciclopirox | etoposide | 0.648 | 0.797 |
| emetine | fendiline | 0.648 | 0.715 |
| chlorzoxazone | clopamide | 0.648 | 0.735 |
| chlorprothixene | mefloquine | 0.648 | 0.715 |
| econazole | terfenadine | 0.648 | 0.768 |
| astemizole | econazole | 0.648 | 0.731 |
| astemizole | benzethonium_chloric | 0.649 | 0.735 |
| disulfiram | mometasone | 0.649 | 0.724 |
| levomepromazine | maprotiline | 0.649 | 0.651 |
| fenbendazole | niclosamide | 0.649 | 0.672 |
| sulconazole | verteporfin | 0.649 | 0.791 |
| liothyronine | sulconazole | 0.650 | 0.735 |
| metyrapone | tyloxapol | 0.650 | 0.886 |
| estriol | milrinone | 0.650 | 0.680 |
| emetine | mefloquine | 0.650 | 0.763 |
| fendiline | pyrvinium | 0.650 | 0.770 |
| roxithromycin | scopolamine | 0.651 | 0.825 |
| ciclopirox | digoxin | 0.651 | 0.856 |
| pimozide | suloctidil | 0.651 | 0.728 |
| metergoline | nortriptyline | 0.651 | 0.731 |
| astemizole | perhexiline | 0.651 | 0.756 |
| metergoline | phenoxybenzamine | 0.651 | 0.730 |
| digoxin | suloctidil | 0.651 | 0.814 |
| benzethonium_chloride | loperamide | 0.651 | 0.721 |
| gliclazide | sulconazole | 0.652 | 0.739 |
| astemizole | desipramine | 0.652 | 0.741 |
| fenoprofen | sulfamethoxypyridazir | 0.652 | 0.682 |
| levonorgestrel | meclofenoxate | 0.652 | 0.747 |
| pipemidic_acid | promethazine | 0.653 | 0.713 |
| bisacodyl | menadione | 0.653 | 0.778 |

| | | | |
|---|---|---|---|
| flupentixol | terfenadine | 0.653 | 0.746 |
| phenoxybenzamine | sulconazole | 0.653 | 0.720 |
| chlorprothixene | clomifene | 0.653 | 0.720 |
| chlortetracycline | dirithromycin | 0.653 | 0.777 |
| clotrimazole | pyrvinium | 0.653 | 0.815 |
| emetine | proscillaridin | 0.654 | 0.763 |
| clomipramine | loperamide | 0.654 | 0.770 |
| perphenazine | protriptyline | 0.655 | 0.705 |
| loperamide | trimipramine | 0.655 | 0.765 |
| astemizole | chlorcyclizine | 0.655 | 0.734 |
| chlorprothixene | loperamide | 0.655 | 0.768 |
| ciclosporin | pizotifen | 0.656 | 0.897 |
| digoxin | phenoxybenzamine | 0.656 | 0.855 |
| loperamide | suloctidil | 0.657 | 0.715 |
| hydrocortisone | urapidil | 0.657 | 0.756 |
| ivermectin | niclosamide | 0.657 | 0.857 |
| niclosamide | perhexiline | 0.657 | 0.766 |
| bufexamac | rifabutin | 0.657 | 0.843 |
| medrysone | sulfamethoxypyridazir | 0.658 | 0.706 |
| bepridil | pyrvinium | 0.658 | 0.798 |
| perphenazine | suloctidil | 0.658 | 0.766 |
| etoposide | phenoxybenzamine | 0.658 | 0.784 |
| lanatoside_C | suloctidil | 0.658 | 0.850 |
| podophyllotoxin | trifluoperazine | 0.658 | 0.710 |
| prenylamine | pyrvinium | 0.658 | 0.764 |
| hydralazine | tropicamide | 0.659 | 0.731 |
| phenoxybenzamine | suloctidil | 0.659 | 0.724 |
| fluphenazine | terfenadine | 0.659 | 0.745 |
| fenbendazole | terfenadine | 0.659 | 0.725 |
| astemizole | ciclosporin | 0.659 | 0.850 |
| metamizole_sodium | sulconazole | 0.659 | 0.710 |
| mefloquine | mometasone | 0.659 | 0.733 |
| etoposide | trifluridine | 0.659 | 0.772 |
| bepridil | mefloquine | 0.660 | 0.756 |
| azacitidine | lanatoside_C | 0.660 | 0.865 |
| phenoxybenzamine | pyrvinium | 0.660 | 0.765 |
| astemizole | mebendazole | 0.660 | 0.750 |
| lomustine | niclosamide | 0.661 | 0.667 |
| pimozide | pyrvinium | 0.661 | 0.767 |
| menadione | suloctidil | 0.661 | 0.756 |
| rifabutin | valproic_acid | 0.661 | 0.876 |
| monobenzone | promethazine | 0.661 | 0.715 |
| miconazole | niclosamide | 0.662 | 0.708 |
| mometasone | perphenazine | 0.662 | 0.744 |
| doxylamine | phenazone | 0.662 | 0.711 |
| benzethonium_chloride | perphenazine | 0.662 | 0.775 |
| imipenem | meticrane | 0.662 | 0.705 |
| menadione | pyrvinium | 0.662 | 0.821 |
| sirolimus | trifluoperazine | 0.662 | 0.827 |
| sulconazole | trazodone | 0.662 | 0.704 |

| | | | |
|---|---|---|---|
| isoconazole | mefloquine | 0.662 | 0.742 |
| astemizole | syrosingopine | 0.662 | 0.756 |
| astemizole | emetine | 0.662 | 0.713 |
| bepridil | suloctidil | 0.662 | 0.761 |
| bepridil | loperamide | 0.662 | 0.729 |
| amoxapine | loperamide | 0.663 | 0.744 |
| benzethonium_chloride | pyrvinium | 0.663 | 0.799 |
| clozapine | trifluoperazine | 0.663 | 0.654 |
| perhexiline | pyrvinium | 0.663 | 0.818 |
| calcium_pantothenate | roxithromycin | 0.664 | 0.805 |
| capsaicin | sulconazole | 0.664 | 0.759 |
| dipyridamole | sulfafurazole | 0.664 | 0.773 |
| meticrane | roxithromycin | 0.664 | 0.849 |
| benzathine_benzylpenicillin | fluticasone | 0.664 | 0.723 |
| clotrimazole | niclosamide | 0.665 | 0.753 |
| milrinone | repaglinide | 0.665 | 0.776 |
| chlorcyclizine | chlorprothixene | 0.665 | 0.664 |
| albendazole | pimozide | 0.665 | 0.758 |
| etacrynic_acid | mepacrine | 0.665 | 0.717 |
| clomipramine | etacrynic_acid | 0.665 | 0.739 |
| disulfiram | menadione | 0.665 | 0.714 |
| terfenadine | zuclopenthixol | 0.665 | 0.724 |
| bromocriptine | etacrynic_acid | 0.666 | 0.771 |
| loperamide | pyrvinium | 0.666 | 0.787 |
| clotrimazole | gefitinib | 0.666 | 0.791 |
| mefloquine | nortriptyline | 0.666 | 0.749 |
| ajmaline | clindamycin | 0.666 | 0.715 |
| miconazole | trifluoperazine | 0.666 | 0.713 |
| loperamide | prenylamine | 0.666 | 0.686 |
| astemizole | pimozide | 0.666 | 0.696 |
| bepridil | prenylamine | 0.667 | 0.660 |
| phenazopyridine | terfenadine | 0.667 | 0.791 |
| miconazole | pyrvinium | 0.667 | 0.754 |
| buflomedil | primaquine | 0.667 | 0.656 |
| dacarbazine | sulconazole | 0.667 | 0.773 |
| proscillaridin | suloctidil | 0.667 | 0.771 |
| rimexolone | sulconazole | 0.668 | 0.760 |
| chlorcyclizine | loperamide | 0.668 | 0.731 |
| bromocriptine | mebendazole | 0.668 | 0.765 |
| fluspirilene | sulconazole | 0.668 | 0.737 |
| mefloquine | rescinnamine | 0.668 | 0.779 |
| nortriptyline | perphenazine | 0.668 | 0.651 |
| benzethonium_chloride | terfenadine | 0.668 | 0.707 |
| metergoline | phenazopyridine | 0.668 | 0.736 |
| decitabine | topiramate | 0.668 | 0.701 |
| doxazosin | etamsylate | 0.669 | 0.776 |
| benzethonium_chloride | prenylamine | 0.669 | 0.673 |
| deptropine | promethazine | 0.669 | 0.669 |
| fluphenazine | metergoline | 0.669 | 0.684 |
| etofenamate | oxamniquine | 0.669 | 0.708 |

| | | | |
|---|---|---|---|
| ketanserin | sulconazole | 0.669 | 0.746 |
| clemastine | trifluoperazine | 0.669 | 0.710 |
| doxazosin | sulfamethoxypyridazir | 0.669 | 0.724 |
| metaraminol | tiapride | 0.669 | 0.722 |
| trifluoperazine | vorinostat | 0.669 | 0.720 |
| nilutamide | promethazine | 0.669 | 0.721 |
| desipramine | metergoline | 0.669 | 0.716 |
| ticarcillin | trifluridine | 0.669 | 0.685 |
| levomepromazine | niclosamide | 0.670 | 0.685 |
| chlorcyclizine | terfenadine | 0.670 | 0.675 |
| sulconazole | tyloxapol | 0.670 | 0.861 |
| monobenzone | phenoxybenzamine | 0.670 | 0.715 |
| butoconazole | trifluoperazine | 0.670 | 0.717 |
| meptazinol | molindone | 0.670 | 0.734 |
| ketanserin | methapyrilene | 0.670 | 0.757 |
| chlorcyclizine | desipramine | 0.670 | 0.667 |
| maprotiline | terfenadine | 0.670 | 0.749 |
| azacitidine | emetine | 0.670 | 0.789 |
| benzethonium_chloride | ivermectin | 0.671 | 0.833 |
| omeprazole | sulconazole | 0.671 | 0.750 |
| selegiline | tobramycin | 0.671 | 0.839 |
| lovastatin | trifluoperazine | 0.671 | 0.726 |
| pizotifen | trifluoperazine | 0.671 | 0.714 |
| clioquinol | terfenadine | 0.671 | 0.815 |
| diltiazem | omeprazole | 0.671 | 0.710 |
| chlorzoxazone | danazol | 0.671 | 0.780 |
| desipramine | mefloquine | 0.671 | 0.740 |
| raloxifene | terfenadine | 0.672 | 0.743 |
| bromperidol | triflusal | 0.672 | 0.796 |
| flupentixol | metergoline | 0.672 | 0.708 |
| loperamide | miconazole | 0.672 | 0.746 |
| diltiazem | methylergometrine | 0.672 | 0.732 |
| chlorzoxazone | flufenamic_acid | 0.672 | 0.701 |
| haloperidol | perphenazine | 0.672 | 0.722 |
| meclofenoxate | medrysone | 0.672 | 0.743 |
| cyproterone | piperidolate | 0.672 | 0.745 |
| mycophenolic_acid | trifluridine | 0.672 | 0.680 |
| dipyridamole | naloxone | 0.673 | 0.770 |
| rimexolone | roxithromycin | 0.673 | 0.805 |
| bromocriptine | gefitinib | 0.673 | 0.739 |
| capsaicin | doxazosin | 0.673 | 0.694 |
| deptropine | rimexolone | 0.673 | 0.747 |
| mitoxantrone | promethazine | 0.673 | 0.709 |
| chlortetracycline | glibenclamide | 0.673 | 0.759 |
| hexetidine | suloctidil | 0.673 | 0.707 |
| metergoline | miconazole | 0.673 | 0.729 |
| oleandomycin | zimeldine | 0.673 | 0.835 |
| fluvoxamine | metergoline | 0.673 | 0.763 |
| fluspirilene | protriptyline | 0.674 | 0.754 |
| benzethonium_chloride | metergoline | 0.674 | 0.700 |

| | | | |
|---|---|---|---|
| sulconazole | sulfametoxydiazine | 0.674 | 0.733 |
| azacitidine | primaquine | 0.674 | 0.699 |
| loperamide | phenazopyridine | 0.674 | 0.765 |
| mebendazole | methylergometrine | 0.674 | 0.676 |
| fenoprofen | medrysone | 0.674 | 0.727 |
| meticrane | sulconazole | 0.674 | 0.773 |
| famotidine | gefitinib | 0.674 | 0.735 |
| bepridil | fluspirilene | 0.675 | 0.757 |
| fluspirilene | perphenazine | 0.675 | 0.713 |
| econazole | phenoxybenzamine | 0.675 | 0.697 |
| estriol | oxprenolol | 0.675 | 0.722 |
| amoxapine | trifluoperazine | 0.675 | 0.706 |
| fluocinonide | metamizole_sodium | 0.675 | 0.747 |
| tranylcypromine | xylometazoline | 0.675 | 0.685 |
| bromocriptine | latamoxef | 0.675 | 0.715 |
| norethisterone | sulconazole | 0.675 | 0.731 |
| fendiline | loperamide | 0.675 | 0.728 |
| lanatoside_C | phenoxybenzamine | 0.676 | 0.871 |
| loperamide | thioridazine | 0.676 | 0.752 |
| gliclazide | triflusal | 0.676 | 0.755 |
| mebendazole | phenoxybenzamine | 0.676 | 0.731 |
| digoxin | pyrvinium | 0.676 | 0.857 |
| metergoline | perhexiline | 0.676 | 0.755 |
| repaglinide | tyloxapol | 0.676 | 0.813 |
| sulconazole | triflusal | 0.676 | 0.782 |
| clomifene | trifluoperazine | 0.676 | 0.738 |
| etacrynic_acid | trifluoperazine | 0.676 | 0.681 |
| clotrimazole | loperamide | 0.677 | 0.705 |
| disulfiram | pyrvinium | 0.677 | 0.799 |
| ciclosporin | mometasone | 0.677 | 0.844 |
| clotrimazole | fendiline | 0.677 | 0.682 |
| clindamycin | clopamide | 0.677 | 0.677 |
| chlorprothixene | naftidrofuryl | 0.677 | 0.714 |
| amitriptyline | ciclosporin | 0.677 | 0.901 |
| desoxycortone | pipemidic_acid | 0.677 | 0.702 |
| menadione | prenylamine | 0.677 | 0.808 |
| piperidolate | repaglinide | 0.677 | 0.716 |
| repaglinide | triflusal | 0.677 | 0.742 |
| chlorcyclizine | niclosamide | 0.678 | 0.762 |
| maprotiline | niclosamide | 0.678 | 0.749 |
| doxazosin | repaglinide | 0.678 | 0.748 |
| fluphenazine | mefloquine | 0.678 | 0.708 |
| bromocriptine | pimozide | 0.678 | 0.708 |
| roxithromycin | sulconazole | 0.678 | 0.838 |
| perphenazine | trimipramine | 0.678 | 0.678 |
| mefloquine | pimozide | 0.678 | 0.780 |
| amoxapine | perphenazine | 0.678 | 0.733 |
| astemizole | clotrimazole | 0.678 | 0.773 |
| latamoxef | sirolimus | 0.678 | 0.793 |
| cyproterone | verteporfin | 0.678 | 0.774 |

| | | | |
|---|---|---|---|
| terfenadine | trimipramine | 0.678 | 0.764 |
| chlorprothixene | fendiline | 0.679 | 0.684 |
| diltiazem | promethazine | 0.679 | 0.723 |
| astemizole | dihydroergotamine | 0.679 | 0.728 |
| flufenamic_acid | sulconazole | 0.679 | 0.748 |
| ciclopirox | lanatoside_C | 0.679 | 0.876 |
| digoxin | prenylamine | 0.679 | 0.855 |
| aciclovir | lisuride | 0.679 | 0.724 |
| fendiline | phenoxybenzamine | 0.679 | 0.658 |
| omeprazole | roxithromycin | 0.679 | 0.826 |
| metergoline | prenylamine | 0.679 | 0.726 |
| cetirizine | fenoprofen | 0.679 | 0.730 |
| ivermectin | terfenadine | 0.679 | 0.807 |
| loperamide | mefloquine | 0.679 | 0.763 |
| sulconazole | sulfamethoxypyridazir | 0.679 | 0.746 |
| diltiazem | sulconazole | 0.679 | 0.734 |
| mefloquine | protriptyline | 0.680 | 0.754 |
| chlorcyclizine | perphenazine | 0.680 | 0.709 |
| latamoxef | sulconazole | 0.680 | 0.790 |
| astemizole | butoconazole | 0.680 | 0.748 |
| lanatoside_C | pyrvinium | 0.680 | 0.867 |
| doxazosin | omeprazole | 0.680 | 0.657 |
| bromocriptine | terfenadine | 0.680 | 0.751 |
| disulfiram | ivermectin | 0.680 | 0.857 |
| fendiline | pimozide | 0.681 | 0.732 |
| mometasone | phenoxybenzamine | 0.681 | 0.753 |
| miconazole | suloctidil | 0.681 | 0.735 |
| doxazosin | promethazine | 0.681 | 0.775 |
| sulconazole | trioxysalen | 0.681 | 0.756 |
| doxazosin | propylthiouracil | 0.681 | 0.710 |
| fluoxetine | trifluoperazine | 0.681 | 0.673 |
| repaglinide | verteporfin | 0.681 | 0.760 |
| etacrynic_acid | pimozide | 0.681 | 0.762 |
| doxazosin | verteporfin | 0.682 | 0.753 |
| cyproterone | sulconazole | 0.682 | 0.745 |
| ciclosporin | trifluoperazine | 0.682 | 0.861 |
| azacitidine | niclosamide | 0.682 | 0.724 |
| ambroxol | amikacin | 0.682 | 0.806 |
| cefsulodin | trimipramine | 0.682 | 0.811 |
| atovaquone | trichlormethiazide | 0.682 | 0.722 |
| perphenazine | prenylamine | 0.682 | 0.725 |
| doxorubicin | sulconazole | 0.682 | 0.747 |
| metixene | terfenadine | 0.682 | 0.738 |
| betahistine | doxazosin | 0.683 | 0.758 |
| astemizole | clomipramine | 0.683 | 0.741 |
| metixene | niclosamide | 0.683 | 0.732 |
| astemizole | maprotiline | 0.683 | 0.757 |
| clomipramine | sulconazole | 0.683 | 0.668 |
| acepromazine | sulconazole | 0.683 | 0.683 |
| fenoprofen | levonorgestrel | 0.683 | 0.715 |

| | | | |
|---|---|---|---|
| cinchocaine | doxazosin | 0.683 | 0.706 |
| doxorubicin | metamizole_sodium | 0.683 | 0.652 |
| levocabastine | talampicillin | 0.684 | 0.701 |
| clotrimazole | suloctidil | 0.684 | 0.752 |
| benzylpenicillin | verteporfin | 0.684 | 0.806 |
| amitriptyline | astemizole | 0.684 | 0.734 |
| meclozine | sulconazole | 0.684 | 0.709 |
| mitoxantrone | primaquine | 0.684 | 0.672 |
| fenoterol | methylergometrine | 0.684 | 0.696 |
| triflusal | tyloxapol | 0.684 | 0.885 |
| mefloquine | thioproperazine | 0.684 | 0.707 |
| benzethonium_chloride | bromocriptine | 0.684 | 0.773 |
| ketanserin | omeprazole | 0.684 | 0.673 |
| chloramphenicol | torasemide | 0.684 | 0.684 |
| proscillaridin | pyrvinium | 0.684 | 0.789 |
| fendiline | fluspirilene | 0.684 | 0.737 |
| etoposide | menadione | 0.684 | 0.809 |
| clomifene | prochlorperazine | 0.685 | 0.724 |
| lanatoside_C | trifluoperazine | 0.685 | 0.857 |
| deptropine | sulconazole | 0.685 | 0.680 |
| chlortetracycline | nilutamide | 0.685 | 0.721 |
| niclosamide | prochlorperazine | 0.685 | 0.743 |
| bepridil | metergoline | 0.685 | 0.729 |
| desipramine | niclosamide | 0.685 | 0.719 |
| liothyronine | roxithromycin | 0.685 | 0.811 |
| etofenamate | sulconazole | 0.685 | 0.745 |
| betahistine | liothyronine | 0.685 | 0.779 |
| succinylsulfathiazole | tolazoline | 0.686 | 0.753 |
| amoxicillin | dextromethorphan | 0.686 | 0.798 |
| gliclazide | imipenem | 0.686 | 0.730 |
| diltiazem | meptazinol | 0.686 | 0.754 |
| estriol | verteporfin | 0.686 | 0.809 |
| quinisocaine | trifluoperazine | 0.686 | 0.670 |
| danazol | glibenclamide | 0.686 | 0.778 |
| naloxone | sulconazole | 0.686 | 0.728 |
| nicotinic_acid | urapidil | 0.686 | 0.702 |
| flecainide | spiramycin | 0.686 | 0.802 |
| econazole | niclosamide | 0.686 | 0.740 |
| fluphenazine | loperamide | 0.686 | 0.711 |
| alfaxalone | remoxipride | 0.686 | 0.713 |
| fluspirilene | maprotiline | 0.686 | 0.746 |
| emetine | pyrvinium | 0.686 | 0.769 |
| gliclazide | repaglinide | 0.686 | 0.740 |
| etacrynic_acid | lomustine | 0.686 | 0.712 |
| diltiazem | monobenzone | 0.686 | 0.730 |
| ketanserin | monobenzone | 0.687 | 0.744 |
| buflomedil | deptropine | 0.687 | 0.745 |
| methylergometrine | terfenadine | 0.687 | 0.742 |
| benfotiamine | citiolone | 0.687 | 0.818 |
| hyoscyamine | pentetrazol | 0.687 | 0.783 |

| | | | |
|---|---|---|---|
| ronidazole | sulconazole | 0.687 | 0.772 |
| carbinoxamine | isopropamide_iodide | 0.687 | 0.691 |
| sulconazole | tropicamide | 0.687 | 0.709 |
| bisacodyl | sulconazole | 0.688 | 0.720 |
| cinchocaine | sulconazole | 0.688 | 0.759 |
| menadione | niclosamide | 0.688 | 0.675 |
| fluspirilene | phenoxybenzamine | 0.688 | 0.734 |
| astemizole | fluvoxamine | 0.688 | 0.765 |
| fenbendazole | trifluoperazine | 0.688 | 0.723 |
| daunorubicin | sulconazole | 0.688 | 0.743 |
| meclozine | pyrvinium | 0.688 | 0.765 |
| carbachol | ioversol | 0.688 | 0.846 |
| clomipramine | labetalol | 0.688 | 0.763 |
| methylergometrine | sirolimus | 0.689 | 0.826 |
| ipratropium_bromide | rimexolone | 0.689 | 0.693 |
| cefotaxime | nalbuphine | 0.689 | 0.722 |
| benfotiamine | sulfadimidine | 0.689 | 0.734 |
| clindamycin | nalbuphine | 0.689 | 0.708 |
| syrosingopine | terfenadine | 0.689 | 0.730 |
| gliclazide | propafenone | 0.689 | 0.680 |
| gefitinib | tolfenamic_acid | 0.690 | 0.716 |
| flupentixol | loperamide | 0.690 | 0.745 |
| metergoline | zuclopenthixol | 0.690 | 0.696 |
| pentoxyverine | sulconazole | 0.690 | 0.750 |
| fenoprofen | sulconazole | 0.690 | 0.710 |
| emetine | niclosamide | 0.690 | 0.740 |
| dipyridamole | sulconazole | 0.690 | 0.773 |
| dihydroergocristine | trifluoperazine | 0.690 | 0.740 |
| irinotecan | methotrexate | 0.690 | 0.725 |
| azacitidine | pyrvinium | 0.690 | 0.834 |
| flavoxate | proxymetacaine | 0.690 | 0.690 |
| dacarbazine | rimexolone | 0.690 | 0.775 |
| clotrimazole | trifluoperazine | 0.690 | 0.758 |
| etacrynic_acid | fendiline | 0.690 | 0.766 |
| chlorprothixene | prenylamine | 0.690 | 0.712 |
| astemizole | fenbendazole | 0.691 | 0.745 |
| metamizole_sodium | promazine | 0.691 | 0.690 |
| disulfiram | fendiline | 0.691 | 0.738 |
| rimexolone | talampicillin | 0.691 | 0.700 |
| metergoline | pimozide | 0.691 | 0.719 |
| doxylamine | pyrimethamine | 0.691 | 0.725 |
| etacrynic_acid | terfenadine | 0.691 | 0.747 |
| clonidine | labetalol | 0.691 | 0.769 |
| fluphenazine | haloperidol | 0.691 | 0.725 |
| piribedil | rimexolone | 0.691 | 0.724 |
| clofazimine | loperamide | 0.691 | 0.721 |
| betahistine | sulconazole | 0.691 | 0.769 |
| fenoprofen | mitoxantrone | 0.691 | 0.770 |
| carbinoxamine | ribostamycin | 0.692 | 0.788 |
| digoxin | fendiline | 0.692 | 0.859 |

| | | | |
|---|---|---|---|
| daunorubicin | doxazosin | 0.692 | 0.677 |
| repaglinide | rimexolone | 0.692 | 0.714 |
| medrysone | repaglinide | 0.692 | 0.705 |
| doxazosin | trioxysalen | 0.692 | 0.771 |
| clomipramine | menadione | 0.692 | 0.751 |
| ciclosporin | cyclobenzaprine | 0.692 | 0.900 |
| bromocriptine | dipyridamole | 0.692 | 0.776 |
| mitoxantrone | roxithromycin | 0.692 | 0.790 |
| fluphenazine | niclosamide | 0.692 | 0.713 |
| ciclosporin | perphenazine | 0.692 | 0.859 |
| etidronic_acid | liothyronine | 0.692 | 0.786 |
| etofenamate | fluvoxamine | 0.692 | 0.695 |
| iopanoic_acid | piribedil | 0.692 | 0.733 |
| tribenoside | trifluoperazine | 0.692 | 0.711 |
| estriol | sulconazole | 0.692 | 0.742 |
| lanatoside_C | mefloquine | 0.692 | 0.846 |
| piribedil | remoxipride | 0.692 | 0.678 |
| ciclosporin | desipramine | 0.693 | 0.899 |
| econazole | pyrvinium | 0.693 | 0.754 |
| haloperidol | metixene | 0.693 | 0.769 |
| citiolone | flunisolide | 0.693 | 0.808 |
| cefalexin | sulconazole | 0.693 | 0.754 |
| astemizole | raloxifene | 0.693 | 0.734 |
| propafenone | trimetazidine | 0.693 | 0.740 |
| mometasone | rescinnamine | 0.693 | 0.770 |
| galantamine | spectinomycin | 0.693 | 0.754 |
| nalidixic_acid | tiapride | 0.693 | 0.652 |
| bepridil | perphenazine | 0.694 | 0.726 |
| emetine | prenylamine | 0.694 | 0.745 |
| pyrazinamide | sulconazole | 0.694 | 0.811 |
| metergoline | trimipramine | 0.694 | 0.766 |
| dicoumarol | flecainide | 0.694 | 0.713 |
| bepridil | clomipramine | 0.694 | 0.669 |
| fendiline | lanatoside_C | 0.694 | 0.884 |
| etomidate | ioxaglic_acid | 0.694 | 0.830 |
| cinchocaine | ticlopidine | 0.694 | 0.742 |
| imipenem | sulconazole | 0.694 | 0.771 |
| omeprazole | pyrazinamide | 0.694 | 0.727 |
| albendazole | bromocriptine | 0.694 | 0.775 |
| benzethonium_chloride | clotrimazole | 0.694 | 0.710 |
| menadione | primaquine | 0.694 | 0.678 |
| isoconazole | ivermectin | 0.695 | 0.849 |
| chlorambucil | nialamide | 0.695 | 0.739 |
| promethazine | rimexolone | 0.695 | 0.757 |
| astemizole | erastin | 0.695 | 0.722 |
| hyoscyamine | pyrantel | 0.695 | 0.732 |
| doxazosin | sulfametoxydiazine | 0.695 | 0.699 |
| riluzole | trimethoprim | 0.695 | 0.652 |
| mitoxantrone | sulconazole | 0.695 | 0.768 |
| irinotecan | menadione | 0.695 | 0.760 |

| | | | |
|---|---|---|---|
| piribedil | sulconazole | 0.695 | 0.727 |
| etacrynic_acid | suloctidil | 0.695 | 0.738 |
| etoposide | terfenadine | 0.695 | 0.761 |
| nabumetone | remoxipride | 0.695 | 0.709 |
| roxithromycin | spectinomycin | 0.695 | 0.822 |
| chlorcyclizine | ciclosporin | 0.695 | 0.889 |
| bromocriptine | mefloquine | 0.696 | 0.761 |
| lanatoside_C | prenylamine | 0.696 | 0.873 |
| cefalotin | labetalol | 0.696 | 0.697 |
| mebendazole | pyrvinium | 0.696 | 0.740 |
| imipramine | moxisylyte | 0.696 | 0.712 |
| irinotecan | mycophenolic_acid | 0.696 | 0.758 |
| ipratropium_bromide | sulconazole | 0.696 | 0.708 |
| indapamide | phentolamine | 0.696 | 0.734 |
| metixene | prenylamine | 0.696 | 0.663 |
| acepromazine | dacarbazine | 0.696 | 0.698 |
| gliclazide | meticrane | 0.696 | 0.687 |
| butoconazole | pyrvinium | 0.696 | 0.765 |
| cetirizine | meptazinol | 0.696 | 0.751 |
| ketanserin | rimexolone | 0.696 | 0.726 |
| chlorprothixene | ciclosporin | 0.696 | 0.899 |
| bromocriptine | fenoterol | 0.696 | 0.743 |
| phenoxybenzamine | promethazine | 0.696 | 0.720 |
| etacrynic_acid | methylergometrine | 0.696 | 0.695 |
| pyrvinium | tribenoside | 0.696 | 0.784 |
| phenoxybenzamine | primaquine | 0.696 | 0.724 |
| gliclazide | rimexolone | 0.696 | 0.746 |
| benfotiamine | dicoumarol | 0.697 | 0.723 |
| repaglinide | talampicillin | 0.697 | 0.741 |
| metamizole_sodium | omeprazole | 0.697 | 0.696 |
| flupentixol | niclosamide | 0.697 | 0.703 |
| liothyronine | piromidic_acid | 0.697 | 0.701 |
| ivermectin | perphenazine | 0.697 | 0.820 |
| lisuride | perphenazine | 0.697 | 0.683 |
| desoxycortone | promethazine | 0.697 | 0.773 |
| rimexolone | tyloxapol | 0.697 | 0.860 |
| gliclazide | trioxysalen | 0.697 | 0.749 |
| etacrynic_acid | loperamide | 0.697 | 0.740 |
| etofenamate | molindone | 0.697 | 0.676 |
| niclosamide | protriptyline | 0.697 | 0.739 |
| flavoxate | piribedil | 0.697 | 0.713 |
| niclosamide | pimozide | 0.697 | 0.720 |
| etacrynic_acid | etoposide | 0.697 | 0.769 |
| desipramine | terfenadine | 0.697 | 0.781 |
| mefloquine | menadione | 0.697 | 0.738 |
| doxazosin | latamoxef | 0.697 | 0.718 |
| omeprazole | tobramycin | 0.697 | 0.744 |
| clotrimazole | phenoxybenzamine | 0.697 | 0.722 |
| methylergometrine | moroxydine | 0.697 | 0.720 |
| buflomedil | fenoprofen | 0.698 | 0.723 |

| | | | |
|---|---|---|---|
| astemizole | lomustine | 0.698 | 0.760 |
| betaxolol | chlorzoxazone | 0.698 | 0.735 |
| buflomedil | mitoxantrone | 0.698 | 0.682 |
| exemestane | rofecoxib | 0.698 | 0.694 |
| altizide | cefalotin | 0.698 | 0.661 |
| niclosamide | syrosingopine | 0.698 | 0.781 |
| monobenzone | sulconazole | 0.698 | 0.669 |
| colchicine | terfenadine | 0.698 | 0.759 |
| bepridil | chlorprothixene | 0.698 | 0.706 |
| flufenamic_acid | glibenclamide | 0.698 | 0.742 |
| calcium_folinate | glycopyrronium_brom | 0.698 | 0.795 |
| dacarbazine | omeprazole | 0.698 | 0.722 |
| clofazimine | metergoline | 0.698 | 0.710 |
| phenazopyridine | pimozide | 0.698 | 0.764 |
| meropenem | trifluridine | 0.698 | 0.706 |
| dopamine | fluphenazine | 0.699 | 0.763 |
| repaglinide | sulconazole | 0.699 | 0.746 |
| bacampicillin | cyanocobalamin | 0.699 | 0.849 |
| ciclopirox | irinotecan | 0.699 | 0.794 |
| cefalexin | meclozine | 0.699 | 0.736 |
| econazole | mefloquine | 0.699 | 0.747 |
| rifabutin | syrosingopine | 0.699 | 0.766 |
| pimozide | syrosingopine | 0.699 | 0.749 |
| desoxycortone | sulconazole | 0.699 | 0.748 |
| etomidate | talampicillin | 0.699 | 0.759 |
| doxazosin | estriol | 0.700 | 0.730 |
| menadione | sulconazole | 0.700 | 0.793 |
| astemizole | levomepromazine | 0.700 | 0.715 |
| hydralazine | medrysone | 0.700 | 0.780 |
| meticrane | sulfametoxydiazine | 0.700 | 0.702 |
| meropenem | verteporfin | 0.700 | 0.792 |
| mesoridazine | propafenone | 0.700 | 0.696 |
| mefloquine | prochlorperazine | 0.700 | 0.720 |
| buflomedil | sulconazole | 0.700 | 0.748 |
| doxazosin | meticrane | 0.700 | 0.771 |
| calcium_folinate | sulfadimethoxine | 0.700 | 0.754 |
| doxazosin | promazine | 0.700 | 0.755 |
| clioquinol | fluspirilene | 0.700 | 0.766 |
| hexetidine | metergoline | 0.700 | 0.750 |
| amiodarone | phenoxybenzamine | 0.700 | 0.751 |
| galantamine | reserpine | 0.700 | 0.779 |
| acetazolamide | benzonatate | 0.700 | 0.808 |
| mesoridazine | triflusal | 0.700 | 0.724 |
| astemizole | metixene | 0.700 | 0.739 |
| astemizole | bromocriptine | 0.700 | 0.725 |
| clomipramine | fluspirilene | 0.701 | 0.753 |
| deptropine | diltiazem | 0.701 | 0.735 |
| bisacodyl | tyloxapol | 0.701 | 0.844 |
| ciclopirox | trifluridine | 0.701 | 0.679 |
| fendiline | proscillaridin | 0.701 | 0.799 |

| | | | |
|---|---|---|---|
| imipenem | triflusal | 0.701 | 0.696 |
| clomifene | fluspirilene | 0.701 | 0.740 |
| fluphenazine | protriptyline | 0.701 | 0.733 |
| bepridil | menadione | 0.701 | 0.792 |
| etacrynic_acid | phenoxybenzamine | 0.701 | 0.745 |
| cyproheptadine | phenoxybenzamine | 0.701 | 0.716 |
| astemizole | verteporfin | 0.701 | 0.765 |
| gefitinib | syrosingopine | 0.701 | 0.769 |
| pyrimethamine | trifluridine | 0.701 | 0.690 |
| demecolcine | trifluoperazine | 0.701 | 0.695 |
| loperamide | phenoxybenzamine | 0.701 | 0.743 |
| dosulepin | phenindione | 0.701 | 0.714 |
| cyproheptadine | loperamide | 0.701 | 0.774 |
| rescinnamine | terconazole | 0.701 | 0.758 |
| cetirizine | doxazosin | 0.701 | 0.738 |
| propafenone | sulconazole | 0.701 | 0.700 |
| alfuzosin | practolol | 0.701 | 0.671 |
| astemizole | lanatoside_C | 0.701 | 0.836 |
| isoconazole | niclosamide | 0.701 | 0.738 |
| chlortetracycline | danazol | 0.701 | 0.749 |
| estriol | triflusal | 0.701 | 0.733 |
| bendroflumethiazide | fluocinonide | 0.702 | 0.715 |
| cefixime | deptropine | 0.702 | 0.798 |
| deptropine | mitoxantrone | 0.702 | 0.762 |
| benzethonium_chloride | fluphenazine | 0.702 | 0.786 |
| sulconazole | zimeldine | 0.702 | 0.674 |
| phenoxybenzamine | trifluridine | 0.702 | 0.775 |
| metergoline | metixene | 0.702 | 0.744 |
| etacrynic_acid | mefloquine | 0.702 | 0.686 |
| budesonide | propafenone | 0.702 | 0.755 |
| amantadine | benzocaine | 0.702 | 0.875 |
| emetine | phenoxybenzamine | 0.702 | 0.731 |
| mometasone | suloctidil | 0.702 | 0.775 |
| perphenazine | phenazopyridine | 0.702 | 0.738 |
| ciclopirox | terfenadine | 0.703 | 0.777 |
| disulfiram | suloctidil | 0.703 | 0.714 |
| loperamide | profenamine | 0.703 | 0.769 |
| proguanil | trifluoperazine | 0.703 | 0.708 |
| betaxolol | glibenclamide | 0.703 | 0.721 |
| imipramine | niclosamide | 0.703 | 0.729 |
| clotrimazole | ivermectin | 0.703 | 0.881 |
| proscillaridin | trifluoperazine | 0.703 | 0.790 |
| acetylsalicylic_acid | sulconazole | 0.703 | 0.782 |
| hexetidine | mometasone | 0.703 | 0.757 |
| minaprine | oxetacaine | 0.703 | 0.700 |
| niclosamide | ticlopidine | 0.703 | 0.704 |
| disulfiram | lanatoside_C | 0.703 | 0.864 |
| protriptyline | pyrvinium | 0.703 | 0.783 |
| chlorprothixene | phenoxybenzamine | 0.703 | 0.709 |
| cefalexin | medrysone | 0.703 | 0.696 |

| | | | |
|---|---|---|---|
| medrysone | piperidolate | 0.703 | 0.734 |
| podophyllotoxin | terfenadine | 0.703 | 0.766 |
| etofenamate | fenoprofen | 0.703 | 0.713 |
| amiodarone | sulconazole | 0.703 | 0.760 |
| methylergometrine | promethazine | 0.703 | 0.719 |
| ciclopirox | deferoxamine | 0.703 | 0.834 |
| griseofulvin | tiapride | 0.703 | 0.680 |
| betaxolol | sulconazole | 0.703 | 0.767 |
| amitriptyline | mometasone | 0.703 | 0.789 |
| fenoprofen | ketanserin | 0.703 | 0.731 |
| cyproheptadine | terfenadine | 0.704 | 0.766 |
| deptropine | omeprazole | 0.704 | 0.766 |
| doxazosin | talampicillin | 0.704 | 0.701 |
| perhexiline | suloctidil | 0.704 | 0.769 |
| trifluoperazine | troglitazone | 0.704 | 0.726 |
| medrysone | metamizole_sodium | 0.704 | 0.691 |
| clotrimazole | terfenadine | 0.704 | 0.732 |
| naftidrofuryl | tacrine | 0.704 | 0.734 |
| artemisinin | dosulepin | 0.704 | 0.754 |
| dicoumarol | spiramycin | 0.704 | 0.828 |
| ivermectin | metergoline | 0.704 | 0.825 |
| astemizole | phenazopyridine | 0.704 | 0.751 |
| cefazolin | mephentermine | 0.704 | 0.815 |
| fluspirilene | miconazole | 0.704 | 0.737 |
| bisacodyl | clomipramine | 0.704 | 0.722 |
| bepridil | proscillaridin | 0.704 | 0.815 |
| sulconazole | xylometazoline | 0.704 | 0.711 |
| rimexolone | tobramycin | 0.704 | 0.759 |
| mefloquine | oxetacaine | 0.704 | 0.714 |
| benzethonium_chloride | suloctidil | 0.704 | 0.703 |
| meptazinol | sulfathiazole | 0.704 | 0.695 |
| dacarbazine | meticrane | 0.704 | 0.663 |
| disulfiram | isoconazole | 0.704 | 0.743 |
| chlorprothixene | disulfiram | 0.704 | 0.762 |
| gliclazide | mesoridazine | 0.704 | 0.743 |
| clomipramine | niclosamide | 0.705 | 0.730 |
| mometasone | pyrvinium | 0.705 | 0.803 |
| buflomedil | succinylsulfathiazole | 0.705 | 0.721 |
| etamsylate | sulconazole | 0.705 | 0.818 |
| sulconazole | sulfamerazine | 0.705 | 0.748 |
| fluvoxamine | terfenadine | 0.705 | 0.769 |
| liothyronine | pyrazinamide | 0.705 | 0.790 |
| emetine | mebendazole | 0.705 | 0.742 |
| rimexolone | ronidazole | 0.705 | 0.784 |
| ceforanide | fluoxetine | 0.705 | 0.757 |
| doxorubicin | meticrane | 0.705 | 0.773 |
| cefalexin | dipyridamole | 0.705 | 0.759 |
| diltiazem | latamoxef | 0.705 | 0.752 |
| benperidol | tobramycin | 0.705 | 0.754 |
| prochlorperazine | terfenadine | 0.705 | 0.752 |

| | | | |
|---|---|---|---|
| chlorprothixene | pyrvinium | 0.705 | 0.769 |
| daunorubicin | medrysone | 0.705 | 0.730 |
| meticrane | scopolamine | 0.705 | 0.691 |
| gliclazide | trimetazidine | 0.705 | 0.725 |
| metergoline | rescinnamine | 0.705 | 0.706 |
| etofenamate | rimexolone | 0.706 | 0.736 |
| fenoprofen | flumetasone | 0.706 | 0.743 |
| acepromazine | deptropine | 0.706 | 0.689 |
| cetirizine | pentoxyverine | 0.706 | 0.698 |
| dopamine | haloperidol | 0.706 | 0.740 |
| procaine | rimexolone | 0.706 | 0.748 |
| clotrimazole | etacrynic_acid | 0.706 | 0.762 |
| dipyridamole | zimeldine | 0.706 | 0.753 |
| cefalexin | rimexolone | 0.706 | 0.686 |
| demecolcine | paclitaxel | 0.706 | 0.819 |
| metergoline | promazine | 0.706 | 0.738 |
| flumetasone | tropicamide | 0.706 | 0.750 |
| chlorcyclizine | metergoline | 0.706 | 0.759 |
| clomipramine | suloctidil | 0.706 | 0.752 |
| doxazosin | tyloxapol | 0.706 | 0.839 |
| bromperidol | sulconazole | 0.706 | 0.738 |
| ciclosporin | imipramine | 0.706 | 0.898 |
| phenoxybenzamine | roxithromycin | 0.706 | 0.853 |
| daunorubicin | scopolamine | 0.706 | 0.727 |
| doxazosin | meclozine | 0.706 | 0.763 |
| roxithromycin | verteporfin | 0.706 | 0.789 |
| ciclosporin | econazole | 0.706 | 0.880 |
| cyproheptadine | trifluoperazine | 0.706 | 0.689 |
| bromperidol | doxazosin | 0.706 | 0.670 |
| cefalexin | nilutamide | 0.706 | 0.692 |
| disulfiram | perphenazine | 0.706 | 0.769 |
| clomipramine | metergoline | 0.706 | 0.730 |
| buflomedil | tolazoline | 0.707 | 0.742 |
| fendiline | perphenazine | 0.707 | 0.716 |
| doxazosin | pivampicillin | 0.707 | 0.700 |
| ambroxol | primidone | 0.707 | 0.738 |
| chloramphenicol | etofylline | 0.707 | 0.676 |
| ciclopirox | phenoxybenzamine | 0.707 | 0.740 |
| astemizole | cyproheptadine | 0.707 | 0.739 |
| bromocriptine | fluspirilene | 0.707 | 0.738 |
| dacarbazine | medrysone | 0.707 | 0.761 |
| cyproheptadine | metergoline | 0.707 | 0.745 |
| cetirizine | procaine | 0.707 | 0.737 |
| etofenamate | piribedil | 0.707 | 0.707 |
| salbutamol | trimethoprim | 0.707 | 0.679 |
| meticrane | sulfaphenazole | 0.707 | 0.683 |
| mefloquine | tribenoside | 0.707 | 0.733 |
| naloxone | zimeldine | 0.707 | 0.733 |
| doxazosin | glibenclamide | 0.707 | 0.702 |
| liothyronine | omeprazole | 0.707 | 0.724 |

| | | | |
|---|---|---|---|
| diltiazem | pentoxyverine | 0.707 | 0.730 |
| acetylsalicylic_acid | tyloxapol | 0.707 | 0.897 |
| cyproheptadine | deptropine | 0.707 | 0.675 |
| emetine | propofol | 0.707 | 0.810 |
| estriol | tyloxapol | 0.707 | 0.861 |
| bromocriptine | sulconazole | 0.707 | 0.769 |
| bromocriptine | colchicine | 0.707 | 0.746 |
| astemizole | oxetacaine | 0.707 | 0.706 |
| dipyridamole | medrysone | 0.707 | 0.743 |
| doxazosin | tobramycin | 0.707 | 0.746 |
| ioversol | rolitetracycline | 0.707 | 0.755 |
| deptropine | latamoxef | 0.708 | 0.803 |
| mebendazole | suloctidil | 0.708 | 0.701 |
| estriol | flufenamic_acid | 0.708 | 0.664 |
| etomidate | repaglinide | 0.708 | 0.758 |
| felodipine | loperamide | 0.708 | 0.743 |
| clobetasol | sulconazole | 0.708 | 0.776 |
| azacitidine | irinotecan | 0.708 | 0.772 |
| meticrane | repaglinide | 0.708 | 0.772 |
| meropenem | piperidolate | 0.708 | 0.749 |
| cefalexin | fenoprofen | 0.708 | 0.700 |
| latamoxef | methylergometrine | 0.708 | 0.716 |
| benzylpenicillin | repaglinide | 0.708 | 0.710 |
| clomipramine | pyrvinium | 0.708 | 0.783 |
| astemizole | digoxin | 0.708 | 0.808 |
| propofol | sulconazole | 0.708 | 0.783 |
| piribedil | procaine | 0.708 | 0.657 |
| doxorubicin | medrysone | 0.708 | 0.746 |
| bromocriptine | suloctidil | 0.708 | 0.757 |
| phenazopyridine | trioxysalen | 0.708 | 0.656 |
| perphenazine | pizotifen | 0.708 | 0.704 |
| levomepromazine | loperamide | 0.708 | 0.765 |
| isosorbide | sulfamerazine | 0.708 | 0.716 |
| bromocriptine | perhexiline | 0.708 | 0.808 |
| aminophylline | sulconazole | 0.708 | 0.808 |
| amoxapine | astemizole | 0.708 | 0.756 |
| chlorprothixene | maprotiline | 0.709 | 0.672 |
| doxazosin | doxorubicin | 0.709 | 0.695 |
| flumetasone | meclofenoxate | 0.709 | 0.773 |
| bromperidol | rimexolone | 0.709 | 0.749 |
| astemizole | ciclopirox | 0.709 | 0.779 |
| fluphenazine | nortriptyline | 0.709 | 0.698 |
| propylthiouracil | selegiline | 0.709 | 0.730 |
| etamsylate | repaglinide | 0.709 | 0.773 |
| meptazinol | methylergometrine | 0.709 | 0.753 |
| bezafibrate | sulconazole | 0.709 | 0.740 |
| meropenem | oxprenolol | 0.709 | 0.753 |
| etoposide | mycophenolic_acid | 0.709 | 0.734 |
| deptropine | nilutamide | 0.709 | 0.783 |
| digoxin | mefloquine | 0.709 | 0.831 |

| | | | |
|---|---|---|---|
| loperamide | pimozide | 0.709 | 0.714 |
| desoxycortone | nilutamide | 0.709 | 0.749 |
| gefitinib | zimeldine | 0.709 | 0.759 |
| levonorgestrel | sulconazole | 0.709 | 0.733 |
| bezafibrate | doxazosin | 0.709 | 0.676 |
| oxyphenbutazone | semustine | 0.709 | 0.668 |
| econazole | nortriptyline | 0.709 | 0.701 |
| doxazosin | flufenamic_acid | 0.709 | 0.735 |
| metyrapone | sulconazole | 0.709 | 0.703 |
| cefalotin | oxprenolol | 0.709 | 0.732 |
| oxetacaine | trifluoperazine | 0.709 | 0.735 |
| bisoprolol | oxantel | 0.709 | 0.725 |
| ciclosporin | diethylstilbestrol | 0.709 | 0.899 |
| cetirizine | medrysone | 0.709 | 0.748 |
| clopamide | dirithromycin | 0.710 | 0.816 |
| deptropine | primaquine | 0.710 | 0.707 |
| doxazosin | pentoxyverine | 0.710 | 0.731 |
| doxazosin | monobenzone | 0.710 | 0.743 |
| chlorzoxazone | sulconazole | 0.710 | 0.766 |
| benfluorex | tribenoside | 0.710 | 0.717 |
| doxazosin | rimexolone | 0.710 | 0.756 |
| meptazinol | trifluoperazine | 0.710 | 0.761 |
| phenoxybenzamine | rescinnamine | 0.710 | 0.798 |
| rimexolone | triflusal | 0.710 | 0.785 |
| mitoxantrone | nilutamide | 0.710 | 0.707 |
| clomipramine | terfenadine | 0.710 | 0.762 |
| chlorzoxazone | triflusal | 0.710 | 0.701 |
| bromopride | sulfamethoxypyridazir | 0.710 | 0.653 |
| fenoprofen | omeprazole | 0.710 | 0.695 |
| doxazosin | rolitetracycline | 0.710 | 0.706 |
| gefitinib | methotrexate | 0.710 | 0.689 |
| theobromine | trichlormethiazide | 0.710 | 0.715 |
| doxazosin | ipratropium_bromide | 0.710 | 0.731 |
| lomustine | terfenadine | 0.711 | 0.781 |
| sulconazole | trimetazidine | 0.711 | 0.723 |
| aminohippuric_acid | tyloxapol | 0.711 | 0.878 |
| bromocriptine | cyproheptadine | 0.711 | 0.785 |
| molindone | tyloxapol | 0.711 | 0.871 |
| (-)-atenolol | urapidil | 0.711 | 0.731 |
| mefloquine | raloxifene | 0.711 | 0.744 |
| felbinac | xamoterol | 0.711 | 0.747 |
| biotin | felodipine | 0.711 | 0.762 |
| bepridil | tyloxapol | 0.711 | 0.854 |
| menadione | syrosingopine | 0.711 | 0.833 |
| cetirizine | dacarbazine | 0.711 | 0.773 |
| doxazosin | natamycin | 0.711 | 0.786 |
| medrysone | roxithromycin | 0.711 | 0.810 |
| benfluorex | pentoxyverine | 0.711 | 0.711 |
| rimexolone | sulfafurazole | 0.711 | 0.751 |
| imipramine | metergoline | 0.711 | 0.744 |

| | | | |
|---|---|---|---|
| medrysone | meticrane | 0.711 | 0.715 |
| astemizole | dosulepin | 0.711 | 0.733 |
| colchicine | tioguanine | 0.711 | 0.768 |
| cetirizine | rimexolone | 0.711 | 0.750 |
| chlorprothixene | fluspirilene | 0.711 | 0.774 |
| meclozine | pentoxyverine | 0.711 | 0.686 |
| amitriptyline | terfenadine | 0.712 | 0.771 |
| colchicine | niclosamide | 0.712 | 0.705 |
| doxazosin | methylergometrine | 0.712 | 0.687 |
| latamoxef | meclozine | 0.712 | 0.774 |
| cetirizine | ketanserin | 0.712 | 0.724 |
| butoconazole | niclosamide | 0.712 | 0.717 |
| bromperidol | propafenone | 0.712 | 0.692 |
| estriol | liothyronine | 0.712 | 0.711 |
| astemizole | thioproperazine | 0.712 | 0.744 |
| piperidolate | roxithromycin | 0.712 | 0.829 |
| doxazosin | liothyronine | 0.712 | 0.727 |
| clomipramine | pimozide | 0.712 | 0.787 |
| betaxolol | doxazosin | 0.712 | 0.726 |
| propantheline_bromide | roxithromycin | 0.712 | 0.813 |
| fluvastatin | urapidil | 0.712 | 0.742 |
| natamycin | piperidolate | 0.712 | 0.820 |
| fluspirilene | nortriptyline | 0.712 | 0.747 |
| piperidolate | verteporfin | 0.712 | 0.797 |
| digoxin | primaquine | 0.712 | 0.850 |
| gliclazide | verteporfin | 0.712 | 0.805 |
| ivermectin | pyrvinium | 0.712 | 0.864 |
| dipyridamole | verteporfin | 0.712 | 0.717 |
| clofazimine | terfenadine | 0.712 | 0.756 |
| chlortetracycline | clindamycin | 0.712 | 0.710 |
| doxazosin | levamisole | 0.712 | 0.760 |
| ambroxol | phenazone | 0.712 | 0.719 |
| cyproheptadine | fluspirilene | 0.712 | 0.753 |
| clomipramine | propafenone | 0.712 | 0.712 |
| amiodarone | dipyridamole | 0.712 | 0.744 |
| etamsylate | gliclazide | 0.712 | 0.667 |
| metergoline | syrosingopine | 0.712 | 0.741 |
| gliclazide | pentoxyverine | 0.712 | 0.654 |
| piromidic_acid | roxithromycin | 0.713 | 0.844 |
| ciclosporin | clotrimazole | 0.713 | 0.886 |
| butoconazole | metergoline | 0.713 | 0.732 |
| lymecycline | meticrane | 0.713 | 0.784 |
| bisacodyl | cefalotin | 0.713 | 0.746 |
| doxazosin | sulfaphenazole | 0.713 | 0.720 |
| clomifene | terfenadine | 0.713 | 0.723 |
| isoniazid | naltrexone | 0.713 | 0.747 |
| doxazosin | reserpine | 0.713 | 0.698 |
| mefloquine | proscillaridin | 0.713 | 0.773 |
| lomustine | trifluoperazine | 0.713 | 0.761 |
| rolitetracycline | sulconazole | 0.713 | 0.775 |

| | | | |
|---|---|---|---|
| bepridil | etacrynic_acid | 0.713 | 0.767 |
| fluvoxamine | molindone | 0.713 | 0.716 |
| daunorubicin | gliclazide | 0.713 | 0.733 |
| ipratropium_bromide | talampicillin | 0.713 | 0.701 |
| nitrofurantoin | sulconazole | 0.713 | 0.754 |
| liothyronine | triflusal | 0.713 | 0.768 |
| dinoprost | vigabatrin | 0.713 | 0.798 |
| doxorubicin | roxithromycin | 0.713 | 0.769 |
| benzethonium_chloride | pimozide | 0.713 | 0.760 |
| medrysone | omeprazole | 0.713 | 0.714 |
| sulconazole | talampicillin | 0.713 | 0.783 |
| buflomedil | cefalexin | 0.713 | 0.679 |
| meticrane | tyloxapol | 0.713 | 0.882 |
| cyproterone | ronidazole | 0.713 | 0.768 |
| doxylamine | oxetacaine | 0.714 | 0.763 |
| nitrendipine | paracetamol | 0.714 | 0.757 |
| etamsylate | piperidolate | 0.714 | 0.769 |
| medrysone | sulfafurazole | 0.714 | 0.715 |
| mitoxantrone | monobenzone | 0.714 | 0.759 |
| miconazole | perphenazine | 0.714 | 0.746 |
| fenoprofen | piribedil | 0.714 | 0.705 |
| dacarbazine | piperidolate | 0.714 | 0.771 |
| digoxin | metergoline | 0.714 | 0.815 |
| dacarbazine | doxazosin | 0.714 | 0.739 |
| cefalotin | rimexolone | 0.714 | 0.747 |
| doxazosin | metyrapone | 0.714 | 0.720 |
| repaglinide | sulfametoxydiazine | 0.714 | 0.751 |
| medrysone | pentoxyverine | 0.714 | 0.746 |
| ivermectin | protriptyline | 0.714 | 0.880 |
| astemizole | tribenoside | 0.714 | 0.720 |
| mebendazole | metergoline | 0.714 | 0.682 |
| etomidate | sulconazole | 0.714 | 0.705 |
| miconazole | prenylamine | 0.714 | 0.705 |
| doxazosin | piperidolate | 0.714 | 0.752 |
| medrysone | promethazine | 0.714 | 0.764 |
| meticrane | ronidazole | 0.714 | 0.672 |
| chlorprothixene | mometasone | 0.715 | 0.800 |
| latamoxef | trifluoperazine | 0.715 | 0.765 |
| fenoprofen | meticrane | 0.715 | 0.693 |
| artemisinin | bromperidol | 0.715 | 0.808 |
| imipenem | phenindione | 0.715 | 0.729 |
| diltiazem | doxazosin | 0.715 | 0.758 |
| pentoxyverine | rimexolone | 0.715 | 0.739 |
| flucloxacillin | rilmenidine | 0.715 | 0.788 |
| quinisocaine | terfenadine | 0.715 | 0.765 |
| doxorubicin | etidronic_acid | 0.715 | 0.825 |
| bisacodyl | daunorubicin | 0.715 | 0.742 |
| menadione | verteporfin | 0.715 | 0.858 |
| dexpanthenol | moxisylyte | 0.715 | 0.743 |
| budesonide | gliclazide | 0.715 | 0.726 |

| | | | |
|---|---|---|---|
| chlorcyclizine | mometasone | 0.715 | 0.788 |
| dipivefrine | tetryzoline | 0.715 | 0.773 |
| estriol | trioxysalen | 0.715 | 0.714 |
| deptropine | medrysone | 0.715 | 0.762 |
| sulfametoxydiazine | triflusal | 0.715 | 0.687 |
| cinchocaine | propylthiouracil | 0.715 | 0.710 |
| natamycin | sulconazole | 0.715 | 0.823 |
| milrinone | sulconazole | 0.715 | 0.756 |
| metamizole_sodium | roxithromycin | 0.715 | 0.829 |
| fendiline | fluphenazine | 0.715 | 0.752 |
| loperamide | menadione | 0.715 | 0.801 |
| loperamide | perhexiline | 0.715 | 0.767 |
| oxprenolol | verteporfin | 0.715 | 0.785 |
| hexetidine | lanatoside_C | 0.715 | 0.867 |
| disulfiram | emetine | 0.715 | 0.747 |
| piperidolate | rimexolone | 0.716 | 0.722 |
| quinisocaine | rescinnamine | 0.716 | 0.770 |
| bromocriptine | dipivefrine | 0.716 | 0.768 |
| disulfiram | oxyphenbutazone | 0.716 | 0.714 |
| buflomedil | cefixime | 0.716 | 0.720 |
| apomorphine | hydrocortisone | 0.716 | 0.782 |
| daunorubicin | mycophenolic_acid | 0.716 | 0.676 |
| fenoprofen | meptazinol | 0.716 | 0.721 |
| dacarbazine | gliclazide | 0.716 | 0.725 |
| doxorubicin | fenoprofen | 0.716 | 0.758 |
| alfuzosin | latamoxef | 0.716 | 0.711 |
| emetine | sirolimus | 0.716 | 0.817 |
| astemizole | imipramine | 0.716 | 0.718 |
| fenoprofen | rimexolone | 0.716 | 0.731 |
| fendiline | metergoline | 0.716 | 0.745 |
| doxorubicin | methotrexate | 0.716 | 0.710 |
| azacitidine | ciclopirox | 0.716 | 0.654 |
| diltiazem | fenoprofen | 0.716 | 0.729 |
| ajmaline | glibenclamide | 0.716 | 0.772 |
| oxybutynin | pimozide | 0.716 | 0.757 |
| clotrimazole | fluspirilene | 0.716 | 0.781 |
| flufenamic_acid | liothyronine | 0.716 | 0.722 |
| dosulepin | terfenadine | 0.716 | 0.764 |
| benfotiamine | flunisolide | 0.716 | 0.708 |
| flufenamic_acid | trioxysalen | 0.716 | 0.665 |
| oxetacaine | protriptyline | 0.716 | 0.759 |
| miconazole | ticlopidine | 0.716 | 0.691 |
| fenoterol | mebendazole | 0.716 | 0.692 |
| ipratropium_bromide | trioxysalen | 0.716 | 0.709 |
| cetirizine | roxithromycin | 0.716 | 0.816 |
| bisacodyl | repaglinide | 0.716 | 0.749 |
| griseofulvin | riluzole | 0.716 | 0.684 |
| valproic_acid | vorinostat | 0.717 | 0.737 |
| galantamine | quinisocaine | 0.717 | 0.661 |
| etamsylate | medrysone | 0.717 | 0.782 |

| | | | |
|---|---|---|---|
| dipyridamole | doxazosin | 0.717 | 0.699 |
| chlorcyclizine | phenoxybenzamine | 0.717 | 0.669 |
| estriol | meticrane | 0.717 | 0.716 |
| doxazosin | metamizole_sodium | 0.717 | 0.692 |
| zalcitabine | zuclopenthixol | 0.717 | 0.766 |
| natamycin | verteporfin | 0.717 | 0.743 |
| cefaclor | methoxamine | 0.717 | 0.719 |
| liothyronine | trioxysalen | 0.717 | 0.769 |
| chlorzoxazone | talampicillin | 0.717 | 0.790 |
| ajmaline | chlorzoxazone | 0.717 | 0.768 |
| doxorubicin | metyrapone | 0.717 | 0.757 |
| nortriptyline | prenylamine | 0.717 | 0.651 |
| bisacodyl | chlorzoxazone | 0.717 | 0.791 |
| chlorzoxazone | hyoscyamine | 0.717 | 0.752 |
| menadione | mometasone | 0.717 | 0.783 |
| hexetidine | perphenazine | 0.717 | 0.769 |
| bromocriptine | sirolimus | 0.717 | 0.779 |
| loperamide | reserpine | 0.717 | 0.730 |
| daunorubicin | phenoxybenzamine | 0.717 | 0.745 |
| sodium_phenylbutyrate | trifluoperazine | 0.717 | 0.719 |
| ketanserin | promethazine | 0.717 | 0.750 |
| doxazosin | tropicamide | 0.717 | 0.770 |
| cyproheptadine | suloctidil | 0.717 | 0.761 |
| nialamide | quinethazone | 0.717 | 0.742 |
| sulfametoxydiazine | trioxysalen | 0.717 | 0.732 |
| terfenadine | thioproperazine | 0.717 | 0.751 |
| cefalexin | doxazosin | 0.717 | 0.719 |
| metergoline | reserpine | 0.717 | 0.695 |
| loperamide | quinisocaine | 0.717 | 0.741 |
| felodipine | hydrocortisone | 0.717 | 0.761 |
| iopanoic_acid | proxymetacaine | 0.717 | 0.701 |
| amiodarone | cetirizine | 0.717 | 0.758 |
| estriol | piperidolate | 0.717 | 0.745 |
| mebendazole | trifluoperazine | 0.718 | 0.703 |
| loperamide | mebendazole | 0.718 | 0.706 |
| clomipramine | syrosingopine | 0.718 | 0.817 |
| fenoprofen | roxithromycin | 0.718 | 0.847 |
| mebendazole | syrosingopine | 0.718 | 0.775 |
| alfuzosin | ambroxol | 0.718 | 0.680 |
| buflomedil | medrysone | 0.718 | 0.703 |
| bromocriptine | zimeldine | 0.718 | 0.780 |
| carbachol | flufenamic_acid | 0.718 | 0.757 |
| dacarbazine | liothyronine | 0.718 | 0.778 |
| mephenesin | salbutamol | 0.718 | 0.672 |
| colchicine | fenbendazole | 0.718 | 0.734 |
| omeprazole | pentoxyverine | 0.718 | 0.705 |
| cefapirin | isopropamide_iodide | 0.718 | 0.775 |
| mefloquine | sirolimus | 0.718 | 0.833 |
| cetirizine | diltiazem | 0.718 | 0.727 |
| alimemazine | methazolamide | 0.718 | 0.706 |

| | | | |
|---|---|---|---|
| cefalotin | doxazosin | 0.718 | 0.679 |
| ivermectin | mometasone | 0.718 | 0.825 |
| estriol | natamycin | 0.718 | 0.810 |
| carbachol | rolitetracycline | 0.718 | 0.843 |
| ethotoin | sulconazole | 0.718 | 0.744 |
| fenbendazole | podophyllotoxin | 0.718 | 0.724 |
| sulconazole | sulfafurazole | 0.718 | 0.730 |
| bepridil | protriptyline | 0.718 | 0.686 |
| doxorubicin | etamsylate | 0.718 | 0.767 |
| colecalciferol | sulconazole | 0.718 | 0.735 |
| meropenem | natamycin | 0.718 | 0.779 |
| benfluorex | metergoline | 0.718 | 0.681 |
| latamoxef | meclofenoxate | 0.718 | 0.755 |
| bepridil | bisacodyl | 0.718 | 0.727 |
| niclosamide | thioproperazine | 0.718 | 0.694 |
| piromidic_acid | verteporfin | 0.718 | 0.783 |
| astemizole | quinisocaine | 0.718 | 0.716 |
| bemegride | doxazosin | 0.718 | 0.818 |
| prenylamine | proscillaridin | 0.719 | 0.795 |
| bromocriptine | diltiazem | 0.719 | 0.752 |
| budesonide | trimetazidine | 0.719 | 0.755 |
| aminophylline | doxazosin | 0.719 | 0.729 |
| ketanserin | metergoline | 0.719 | 0.692 |
| simvastatin | trichlormethiazide | 0.719 | 0.732 |
| amiodarone | pentoxyverine | 0.719 | 0.734 |
| gliclazide | zimeldine | 0.719 | 0.735 |
| artemisinin | phenindione | 0.719 | 0.764 |
| metergoline | raloxifene | 0.719 | 0.730 |
| benzethonium_chloride | desipramine | 0.719 | 0.716 |
| cyproterone | repaglinide | 0.719 | 0.744 |
| doxazosin | nitrofurantoin | 0.719 | 0.692 |
| medrysone | verteporfin | 0.719 | 0.796 |
| diltiazem | rimexolone | 0.719 | 0.733 |
| levothyroxine_sodium | metrizamide | 0.719 | 0.716 |
| terfenadine | tribenoside | 0.719 | 0.751 |
| clioquinol | lanatoside_C | 0.719 | 0.906 |
| nialamide | tinidazole | 0.719 | 0.714 |
| meticrane | norfloxacin | 0.719 | 0.672 |
| etacrynic_acid | gefitinib | 0.719 | 0.714 |
| doxazosin | phenazopyridine | 0.719 | 0.682 |
| flupentixol | suloctidil | 0.719 | 0.742 |
| cetirizine | deptropine | 0.719 | 0.707 |
| cetirizine | pyrazinamide | 0.719 | 0.793 |
| piperidolate | ronidazole | 0.719 | 0.762 |
| emetine | verteporfin | 0.719 | 0.769 |
| fluvoxamine | mefloquine | 0.719 | 0.694 |
| bromocriptine | trifluoperazine | 0.719 | 0.719 |
| doxazosin | menadione | 0.719 | 0.734 |
| etacrynic_acid | menadione | 0.720 | 0.697 |
| galantamine | probenecid | 0.720 | 0.715 |

| | | | |
|---|---|---|---|
| talampicillin | verteporfin | 0.720 | 0.772 |
| fendiline | mebendazole | 0.720 | 0.752 |
| prochlorperazine | protriptyline | 0.720 | 0.723 |
| clotrimazole | syrosingopine | 0.720 | 0.835 |
| cefalotin | trioxysalen | 0.720 | 0.749 |
| methylergometrine | sulconazole | 0.720 | 0.738 |
| astemizole | nicergoline | 0.720 | 0.723 |
| apomorphine | irinotecan | 0.720 | 0.757 |
| sulconazole | sulfaphenazole | 0.720 | 0.723 |
| flufenamic_acid | talampicillin | 0.720 | 0.723 |
| metergoline | rifabutin | 0.720 | 0.803 |
| fluspirilene | propafenone | 0.720 | 0.675 |
| pivampicillin | sulconazole | 0.720 | 0.796 |
| monobenzone | pipemidic_acid | 0.720 | 0.683 |
| deptropine | pentoxyverine | 0.720 | 0.721 |
| indoprofen | letrozole | 0.720 | 0.716 |
| terfenadine | thioridazine | 0.720 | 0.759 |
| carbachol | sulconazole | 0.720 | 0.734 |
| doxazosin | metacycline | 0.720 | 0.706 |
| deptropine | ketanserin | 0.720 | 0.757 |
| bromperidol | tyloxapol | 0.720 | 0.846 |
| metergoline | mometasone | 0.720 | 0.745 |
| sulconazole | tranylcypromine | 0.720 | 0.772 |
| desipramine | ivermectin | 0.720 | 0.881 |
| ethionamide | terconazole | 0.720 | 0.812 |
| ciclopirox | mometasone | 0.720 | 0.748 |
| estriol | etamsylate | 0.720 | 0.769 |
| phenoxybenzamine | syrosingopine | 0.720 | 0.813 |
| clindamycin | danazol | 0.720 | 0.765 |
| meticrane | omeprazole | 0.720 | 0.713 |
| cetirizine | gliclazide | 0.720 | 0.713 |
| amiodarone | ifenprodil | 0.720 | 0.743 |
| decitabine | nadolol | 0.720 | 0.729 |
| octopamine | torasemide | 0.720 | 0.733 |
| mefloquine | rifabutin | 0.720 | 0.802 |
| thiocolchicoside | trimetazidine | 0.720 | 0.753 |
| diltiazem | ketanserin | 0.720 | 0.735 |
| rimexolone | scopolamine | 0.720 | 0.670 |
| pyrvinium | ticlopidine | 0.721 | 0.762 |
| buflomedil | calcium_pantothenate | 0.721 | 0.792 |
| desoxycortone | tyloxapol | 0.721 | 0.859 |
| iopromide | pyrithyldione | 0.721 | 0.830 |
| griseofulvin | nalidixic_acid | 0.721 | 0.671 |
| benzethonium_chloride | perhexiline | 0.721 | 0.752 |
| bufexamac | vorinostat | 0.721 | 0.653 |
| metergoline | thioproperazine | 0.721 | 0.719 |
| astemizole | zuclopenthixol | 0.721 | 0.735 |
| ganciclovir | sulconazole | 0.721 | 0.766 |
| cefalexin | cetirizine | 0.721 | 0.724 |
| omeprazole | tyloxapol | 0.721 | 0.851 |

| | | | |
|---|---|---|---|
| glibenclamide | tyloxapol | 0.721 | 0.830 |
| meptazinol | promazine | 0.721 | 0.718 |
| doxazosin | meropenem | 0.721 | 0.728 |
| bepridil | perhexiline | 0.721 | 0.680 |
| isoconazole | prenylamine | 0.721 | 0.712 |
| etofenamate | meclofenoxate | 0.721 | 0.682 |
| flufenamic_acid | tyloxapol | 0.721 | 0.869 |
| propofol | terfenadine | 0.721 | 0.817 |
| pargyline | rimexolone | 0.721 | 0.807 |
| disulfiram | proscillaridin | 0.721 | 0.797 |
| betahistine | metamizole_sodium | 0.721 | 0.658 |
| deptropine | perhexiline | 0.721 | 0.692 |
| buflomedil | cetirizine | 0.721 | 0.753 |
| benperidol | probenecid | 0.721 | 0.732 |
| menadione | pimozide | 0.722 | 0.781 |
| ciclosporin | prochlorperazine | 0.722 | 0.861 |
| idoxuridine | isoxsuprine | 0.722 | 0.730 |
| bepridil | fendiline | 0.722 | 0.664 |
| terguride | urapidil | 0.722 | 0.756 |
| cetirizine | metyrapone | 0.722 | 0.761 |
| celecoxib | tacrolimus | 0.722 | 0.815 |
| enoxacin | probucol | 0.722 | 0.774 |
| cefalexin | zimeldine | 0.722 | 0.745 |
| metergoline | prochlorperazine | 0.722 | 0.714 |
| natamycin | repaglinide | 0.722 | 0.781 |
| desipramine | prochlorperazine | 0.722 | 0.675 |
| doxazosin | roxithromycin | 0.722 | 0.814 |
| clidinium_bromide | iopromide | 0.722 | 0.769 |
| albendazole | trifluoperazine | 0.722 | 0.715 |
| bromocriptine | naloxone | 0.722 | 0.786 |
| scopolamine | sulconazole | 0.722 | 0.745 |
| benzylpenicillin | cyproterone | 0.722 | 0.734 |
| liothyronine | tyloxapol | 0.722 | 0.838 |
| mefloquine | tamoxifen | 0.722 | 0.773 |
| piperidolate | propylthiouracil | 0.722 | 0.764 |
| disulfiram | metergoline | 0.722 | 0.745 |
| isoconazole | phenoxybenzamine | 0.722 | 0.719 |
| propafenone | zimeldine | 0.722 | 0.713 |
| fendiline | menadione | 0.722 | 0.797 |
| clioquinol | phenoxybenzamine | 0.722 | 0.730 |
| doxazosin | naloxone | 0.722 | 0.792 |
| disulfiram | prenylamine | 0.722 | 0.750 |
| flunisolide | sulconazole | 0.722 | 0.785 |
| clomipramine | clonidine | 0.722 | 0.716 |
| pyrantel | sulfaphenazole | 0.722 | 0.714 |
| doxazosin | fenoprofen | 0.722 | 0.749 |
| medrysone | sulfamerazine | 0.722 | 0.729 |
| daunorubicin | etamsylate | 0.722 | 0.757 |
| ciclopirox | primaquine | 0.722 | 0.667 |
| fenbufen | hydralazine | 0.722 | 0.672 |

| | | | |
|---|---|---|---|
| bromocriptine | syrosingopine | 0.722 | 0.729 |
| clomipramine | rimexolone | 0.722 | 0.770 |
| niclosamide | oxyphenbutazone | 0.722 | 0.702 |
| bepridil | bromocriptine | 0.723 | 0.766 |
| piperidolate | sertaconazole | 0.723 | 0.706 |
| clofazimine | maprotiline | 0.723 | 0.724 |
| cyproterone | estriol | 0.723 | 0.660 |
| phenoxybenzamine | proscillaridin | 0.723 | 0.797 |
| sulfametoxydiazine | tyloxapol | 0.723 | 0.869 |
| biotin | hydrocortisone | 0.723 | 0.712 |
| desoxycortone | metyrapone | 0.723 | 0.731 |
| ciclosporin | maprotiline | 0.723 | 0.895 |
| etoposide | loperamide | 0.723 | 0.742 |
| biotin | urapidil | 0.723 | 0.764 |
| digoxin | trifluoperazine | 0.723 | 0.828 |
| cetirizine | monobenzone | 0.723 | 0.745 |
| procaine | sulconazole | 0.723 | 0.735 |
| albendazole | fendiline | 0.723 | 0.756 |
| fluspirilene | fluvoxamine | 0.723 | 0.754 |
| acepromazine | doxazosin | 0.723 | 0.708 |
| talampicillin | trazodone | 0.723 | 0.703 |
| meticrane | proxyphylline | 0.723 | 0.658 |
| etacrynic_acid | semustine | 0.723 | 0.704 |
| procaine | propafenone | 0.723 | 0.698 |
| benzethonium_chloride | syrosingopine | 0.723 | 0.768 |
| buflomedil | latamoxef | 0.723 | 0.763 |
| dexpanthenol | triflupromazine | 0.723 | 0.754 |
| benzethonium_chloride | bepridil | 0.723 | 0.729 |
| repaglinide | trioxysalen | 0.723 | 0.760 |
| hydralazine | sulfamerazine | 0.723 | 0.688 |
| estriol | talampicillin | 0.723 | 0.766 |
| oxprenolol | piperidolate | 0.723 | 0.682 |
| bisacodyl | pyrvinium | 0.723 | 0.791 |
| promazine | sulconazole | 0.723 | 0.693 |
| prenylamine | trimipramine | 0.723 | 0.658 |
| colchicine | suloctidil | 0.723 | 0.757 |
| cyproterone | meticrane | 0.723 | 0.741 |
| piperidolate | promethazine | 0.723 | 0.707 |
| minaprine | sulfaguanidine | 0.723 | 0.736 |
| maprotiline | prochlorperazine | 0.724 | 0.735 |
| etofenamate | oxybuprocaine | 0.724 | 0.695 |
| cetirizine | promethazine | 0.724 | 0.726 |
| bisacodyl | cetirizine | 0.724 | 0.677 |
| piromidic_acid | sulconazole | 0.724 | 0.734 |
| fluvastatin | rolitetracycline | 0.724 | 0.730 |
| protriptyline | zuclopenthixol | 0.724 | 0.712 |
| desoxycortone | doxazosin | 0.724 | 0.728 |
| meropenem | repaglinide | 0.724 | 0.742 |
| loperamide | thioproperazine | 0.724 | 0.726 |
| alprostadil | podophyllotoxin | 0.724 | 0.751 |

| | | | |
|---|---|---|---|
| hyoscyamine | molindone | 0.724 | 0.708 |
| amikacin | meptazinol | 0.724 | 0.853 |
| atovaquone | reserpine | 0.724 | 0.726 |
| cefixime | cyanocobalamin | 0.724 | 0.845 |
| albendazole | terfenadine | 0.724 | 0.716 |
| oxybuprocaine | sulconazole | 0.724 | 0.751 |
| flufenamic_acid | verteporfin | 0.724 | 0.799 |
| natamycin | trifluridine | 0.724 | 0.805 |
| omeprazole | rimexolone | 0.724 | 0.738 |
| molindone | sulfaphenazole | 0.724 | 0.685 |
| ciclosporin | trimipramine | 0.724 | 0.896 |
| cefalotin | meticrane | 0.724 | 0.747 |
| bromocriptine | loperamide | 0.724 | 0.697 |
| syrosingopine | vorinostat | 0.724 | 0.767 |
| doxazosin | ioversol | 0.724 | 0.793 |
| gliclazide | oxprenolol | 0.724 | 0.682 |
| repaglinide | ronidazole | 0.724 | 0.732 |
| dienestrol | lynestrenol | 0.724 | 0.764 |
| bepridil | clomifene | 0.724 | 0.723 |
| mepacrine | methylergometrine | 0.725 | 0.671 |
| meticrane | natamycin | 0.725 | 0.812 |
| acepromazine | gefitinib | 0.725 | 0.700 |
| clomifene | metergoline | 0.725 | 0.687 |
| etofylline | levamisole | 0.725 | 0.671 |
| gliclazide | norethisterone | 0.725 | 0.791 |
| piperacillin | sulfadimidine | 0.725 | 0.745 |
| levomepromazine | metergoline | 0.725 | 0.743 |
| liothyronine | spectinomycin | 0.725 | 0.759 |
| roxithromycin | trioxysalen | 0.725 | 0.875 |
| doxorubicin | repaglinide | 0.725 | 0.745 |
| cefalotin | sulconazole | 0.725 | 0.779 |
| lomustine | mefloquine | 0.725 | 0.712 |
| proxymetacaine | remoxipride | 0.725 | 0.666 |
| primaquine | pyrvinium | 0.725 | 0.774 |
| benzylpenicillin | oxprenolol | 0.725 | 0.707 |
| omeprazole | promethazine | 0.725 | 0.747 |
| metyrapone | pipemidic_acid | 0.725 | 0.679 |
| dosulepin | metergoline | 0.725 | 0.733 |
| triflusal | trioxysalen | 0.725 | 0.708 |
| artemisinin | molsidomine | 0.725 | 0.768 |
| cyanocobalamin | fenoprofen | 0.725 | 0.889 |
| etacrynic_acid | pyrvinium | 0.725 | 0.770 |
| doxorubicin | sulfaphenazole | 0.725 | 0.715 |
| gliclazide | milrinone | 0.725 | 0.761 |
| atovaquone | spectinomycin | 0.725 | 0.751 |
| nimesulide | pargyline | 0.725 | 0.727 |
| doxazosin | triflusal | 0.725 | 0.777 |
| betahistine | molindone | 0.725 | 0.697 |
| moracizine | pivmecillinam | 0.725 | 0.738 |
| meclofenoxate | sulconazole | 0.725 | 0.725 |

| | | | |
|---|---|---|---|
| haloperidol | trifluoperazine | 0.725 | 0.722 |
| cycloserine | sulconazole | 0.725 | 0.833 |
| dicoumarol | piperacillin | 0.725 | 0.730 |
| cetirizine | tyloxapol | 0.725 | 0.826 |
| etofenamate | ketanserin | 0.725 | 0.726 |
| phenoxybenzamine | vorinostat | 0.725 | 0.726 |
| demecolcine | podophyllotoxin | 0.725 | 0.717 |
| sulfamethoxypyridazine | tyloxapol | 0.725 | 0.872 |
| betaxolol | tyloxapol | 0.725 | 0.861 |
| astemizole | terconazole | 0.725 | 0.730 |
| tamoxifen | trifluoperazine | 0.725 | 0.734 |
| trazodone | verteporfin | 0.725 | 0.795 |
| primaquine | terfenadine | 0.725 | 0.754 |
| metacycline | sulconazole | 0.725 | 0.740 |
| emetine | pimozide | 0.725 | 0.715 |
| levodopa | sulpiride | 0.725 | 0.728 |
| exemestane | verapamil | 0.725 | 0.756 |
| doxazosin | trichlormethiazide | 0.725 | 0.743 |
| piperidolate | sulfamethoxypyridazir | 0.726 | 0.703 |
| colecalciferol | doxazosin | 0.726 | 0.715 |
| ifenprodil | rimexolone | 0.726 | 0.706 |
| astemizole | thioridazine | 0.726 | 0.739 |
| amiodarone | doxazosin | 0.726 | 0.717 |
| metamizole_sodium | mitoxantrone | 0.726 | 0.708 |
| fluspirilene | oxetacaine | 0.726 | 0.743 |
| amodiaquine | clomipramine | 0.726 | 0.737 |
| cycloserine | etamsylate | 0.726 | 0.689 |
| medrysone | mitoxantrone | 0.726 | 0.739 |
| pyrvinium | syrosingopine | 0.726 | 0.792 |
| benfluorex | practolol | 0.726 | 0.706 |
| flufenamic_acid | triflusal | 0.726 | 0.675 |
| fluspirilene | phenazopyridine | 0.726 | 0.740 |
| gefitinib | gliclazide | 0.726 | 0.734 |
| doxylamine | sulfathiazole | 0.726 | 0.670 |
| bromopride | carbachol | 0.726 | 0.751 |
| chlorzoxazone | tyloxapol | 0.726 | 0.910 |
| acepromazine | rimexolone | 0.726 | 0.740 |
| ciclopirox | proscillaridin | 0.726 | 0.794 |
| propylthiouracil | verteporfin | 0.726 | 0.851 |
| ioxaglic_acid | risperidone | 0.726 | 0.764 |
| estriol | omeprazole | 0.726 | 0.742 |
| dacarbazine | tyloxapol | 0.726 | 0.895 |
| pivampicillin | torasemide | 0.726 | 0.739 |
| trazodone | tyloxapol | 0.726 | 0.850 |
| chlorprothixene | menadione | 0.726 | 0.705 |
| dipyridamole | gliclazide | 0.726 | 0.776 |
| fluticasone | fluvoxamine | 0.726 | 0.735 |
| methapyrilene | sulconazole | 0.726 | 0.696 |
| chlorcyclizine | levomepromazine | 0.726 | 0.687 |
| cycloserine | ronidazole | 0.726 | 0.740 |

| | | | |
|---|---|---|---|
| etamsylate | tyloxapol | 0.726 | 0.904 |
| etamsylate | verteporfin | 0.726 | 0.862 |
| bisacodyl | meclozine | 0.726 | 0.674 |
| meticrane | talampicillin | 0.726 | 0.749 |
| cycloserine | gliclazide | 0.726 | 0.850 |
| clemastine | nortriptyline | 0.726 | 0.679 |
| dosulepin | trifluoperazine | 0.726 | 0.670 |
| flufenamic_acid | phenoxybenzamine | 0.726 | 0.744 |
| clomipramine | prenylamine | 0.727 | 0.662 |
| griseofulvin | sulfamethoxazole | 0.727 | 0.675 |
| nafcillin | theophylline | 0.727 | 0.786 |
| astemizole | chlorphenamine | 0.727 | 0.730 |
| carbachol | triflusal | 0.727 | 0.697 |
| irinotecan | vorinostat | 0.727 | 0.753 |
| azacitidine | menadione | 0.727 | 0.685 |
| amoxapine | bromocriptine | 0.727 | 0.787 |
| ambroxol | mafenide | 0.727 | 0.733 |
| flunisolide | meticrane | 0.727 | 0.743 |
| deptropine | sulpiride | 0.727 | 0.756 |
| trioxysalen | tyloxapol | 0.727 | 0.894 |
| daunorubicin | roxithromycin | 0.727 | 0.771 |
| metacycline | verteporfin | 0.727 | 0.755 |
| chlorprothixene | digoxin | 0.727 | 0.878 |
| mometasone | protriptyline | 0.727 | 0.783 |
| sulfinpyrazone | trioxysalen | 0.727 | 0.738 |
| cefalexin | dacarbazine | 0.727 | 0.724 |
| clomifene | sulconazole | 0.727 | 0.724 |
| meticrane | naloxone | 0.727 | 0.731 |
| doxazosin | ethotoin | 0.727 | 0.752 |
| cetirizine | trazodone | 0.727 | 0.709 |
| isoconazole | menadione | 0.727 | 0.753 |
| methylergometrine | practolol | 0.727 | 0.703 |
| dacarbazine | ipratropium_bromide | 0.727 | 0.753 |
| ifenprodil | sulconazole | 0.727 | 0.725 |
| maprotiline | zuclopenthixol | 0.727 | 0.730 |
| latamoxef | phenoxybenzamine | 0.727 | 0.776 |
| oxamic_acid | zimeldine | 0.727 | 0.892 |
| hydrocortisone | nicotinic_acid | 0.727 | 0.835 |
| fluspirilene | mebendazole | 0.727 | 0.710 |
| gefitinib | trifluoperazine | 0.727 | 0.675 |
| acepromazine | chlorzoxazone | 0.727 | 0.727 |
| levamisole | repaglinide | 0.727 | 0.780 |
| nortriptyline | prochlorperazine | 0.727 | 0.707 |
| dipyridamole | meclozine | 0.727 | 0.800 |
| danazol | dirithromycin | 0.727 | 0.828 |
| repaglinide | ursodeoxycholic_acid | 0.727 | 0.708 |
| isoconazole | mometasone | 0.727 | 0.758 |
| dihydroergotamine | terfenadine | 0.728 | 0.748 |
| niclosamide | podophyllotoxin | 0.728 | 0.749 |
| bromocriptine | nortriptyline | 0.728 | 0.782 |

| | | | |
|---|---|---|---|
| etamsylate | trazodone | 0.728 | 0.758 |
| bromperidol | thiocolchicoside | 0.728 | 0.753 |
| dihydroergocristine | loperamide | 0.728 | 0.687 |
| bepridil | miconazole | 0.728 | 0.714 |
| etamsylate | rimexolone | 0.728 | 0.788 |
| benzethonium_chloride | sirolimus | 0.728 | 0.848 |
| dilazep | vorinostat | 0.728 | 0.790 |
| latamoxef | medrysone | 0.728 | 0.764 |
| doxorubicin | propylthiouracil | 0.728 | 0.748 |
| econazole | isotretinoin | 0.728 | 0.728 |
| daunorubicin | fenoprofen | 0.728 | 0.739 |
| betahistine | cetirizine | 0.728 | 0.757 |
| gabapentin | pridinol | 0.728 | 0.756 |
| fluocinonide | promazine | 0.728 | 0.797 |
| felodipine | profenamine | 0.728 | 0.701 |
| fluvoxamine | oxamniquine | 0.728 | 0.680 |
| clioquinol | prenylamine | 0.728 | 0.749 |
| metacycline | propylthiouracil | 0.728 | 0.738 |
| fenoprofen | metamizole_sodium | 0.728 | 0.662 |
| aminohippuric_acid | sulconazole | 0.728 | 0.720 |
| aminohippuric_acid | doxazosin | 0.728 | 0.691 |
| pentoxyverine | sulfafurazole | 0.728 | 0.715 |
| fluocinonide | sulconazole | 0.728 | 0.781 |
| estriol | mesalazine | 0.728 | 0.750 |
| astemizole | clioquinol | 0.728 | 0.775 |
| clofazimine | niclosamide | 0.728 | 0.675 |
| piperidolate | sulfafurazole | 0.728 | 0.697 |
| promethazine | tyloxapol | 0.728 | 0.873 |
| clotrimazole | mefloquine | 0.728 | 0.758 |
| medrysone | thiocolchicoside | 0.728 | 0.747 |
| bisacodyl | estriol | 0.728 | 0.750 |
| terguride | tyloxapol | 0.728 | 0.859 |
| bepridil | fluphenazine | 0.728 | 0.734 |
| azacitidine | terfenadine | 0.728 | 0.820 |
| mepacrine | metergoline | 0.728 | 0.713 |
| bepridil | lanatoside_C | 0.728 | 0.883 |
| mepacrine | terfenadine | 0.728 | 0.765 |
| prednisolone | thalidomide | 0.728 | 0.683 |
| bepridil | digoxin | 0.728 | 0.863 |
| fenbendazole | vinblastine | 0.728 | 0.814 |
| bepridil | phenoxybenzamine | 0.728 | 0.698 |
| cefalexin | sulfafurazole | 0.728 | 0.685 |
| bisacodyl | talampicillin | 0.728 | 0.736 |
| glibenclamide | liothyronine | 0.728 | 0.725 |
| rimexolone | trazodone | 0.728 | 0.766 |
| levocabastine | mecamylamine | 0.728 | 0.842 |
| ambroxol | doxylamine | 0.728 | 0.708 |
| procaine | roxithromycin | 0.728 | 0.853 |
| oxyphenbutazone | trifluoperazine | 0.728 | 0.667 |
| bepridil | colchicine | 0.729 | 0.755 |

| | | | |
|---|---|---|---|
| aminohippuric_acid | promethazine | 0.729 | 0.700 |
| diltiazem | gliclazide | 0.729 | 0.714 |
| medrysone | trioxysalen | 0.729 | 0.717 |
| remoxipride | sulconazole | 0.729 | 0.741 |
| chlorcyclizine | prenylamine | 0.729 | 0.675 |
| reserpine | spectinomycin | 0.729 | 0.776 |
| buflomedil | dacarbazine | 0.729 | 0.706 |
| rimexolone | trioxysalen | 0.729 | 0.760 |
| acetylsalicylic_acid | liothyronine | 0.729 | 0.768 |
| cefalexin | guanfacine | 0.729 | 0.719 |
| butoconazole | loperamide | 0.729 | 0.731 |
| amoxapine | cyproheptadine | 0.729 | 0.688 |
| atovaquone | galantamine | 0.729 | 0.772 |
| miconazole | protriptyline | 0.729 | 0.673 |
| meticrane | verteporfin | 0.729 | 0.827 |
| chloramphenicol | pivampicillin | 0.729 | 0.734 |
| ciclosporin | metergoline | 0.729 | 0.853 |
| fluvastatin | profenamine | 0.729 | 0.694 |
| estriol | ronidazole | 0.729 | 0.681 |
| primaquine | suloctidil | 0.729 | 0.727 |
| clotrimazole | erastin | 0.729 | 0.785 |
| albendazole | clonidine | 0.729 | 0.705 |
| dantrolene | ethisterone | 0.729 | 0.764 |
| mefloquine | mepacrine | 0.729 | 0.749 |
| aminohippuric_acid | terguride | 0.729 | 0.712 |
| gliclazide | natamycin | 0.729 | 0.809 |
| carbachol | repaglinide | 0.729 | 0.776 |
| aminohippuric_acid | urapidil | 0.729 | 0.721 |
| flupentixol | pyrvinium | 0.729 | 0.739 |
| cefsulodin | nafcillin | 0.729 | 0.686 |
| clomipramine | levamisole | 0.729 | 0.724 |
| hydralazine | meticrane | 0.729 | 0.668 |
| bromperidol | mesoridazine | 0.730 | 0.745 |
| amiodarone | bisacodyl | 0.730 | 0.721 |
| flufenamic_acid | norethisterone | 0.730 | 0.717 |
| betahistine | probenecid | 0.730 | 0.719 |
| etofenamate | omeprazole | 0.730 | 0.701 |
| remoxipride | rimexolone | 0.730 | 0.715 |
| metacycline | moxonidine | 0.730 | 0.706 |
| hydralazine | rimexolone | 0.730 | 0.778 |
| alfaxalone | nabumetone | 0.730 | 0.711 |
| sulconazole | tobramycin | 0.730 | 0.795 |
| mesalazine | oxprenolol | 0.730 | 0.719 |
| bepridil | pimozide | 0.730 | 0.765 |
| bepridil | mebendazole | 0.730 | 0.756 |
| glibenclamide | meticrane | 0.730 | 0.750 |
| tyloxapol | urapidil | 0.730 | 0.848 |
| flunisolide | promazine | 0.730 | 0.788 |
| amoxicillin | meticrane | 0.730 | 0.746 |
| econazole | prenylamine | 0.730 | 0.723 |

| | | | |
|---|---|---|---|
| benfluorex | promazine | 0.730 | 0.757 |
| alimemazine | vidarabine | 0.730 | 0.713 |
| metixene | zuclopenthixol | 0.730 | 0.682 |
| chlortetracycline | deptropine | 0.730 | 0.754 |
| dacarbazine | verteporfin | 0.730 | 0.844 |
| metamizole_sodium | meticrane | 0.730 | 0.691 |
| cetirizine | minaprine | 0.730 | 0.718 |
| talampicillin | triflusal | 0.730 | 0.770 |
| carbachol | doxorubicin | 0.730 | 0.842 |
| metyrapone | omeprazole | 0.730 | 0.679 |
| nafcillin | pralidoxime | 0.730 | 0.715 |
| lomefloxacin | tropicamide | 0.730 | 0.736 |
| piribedil | talampicillin | 0.730 | 0.723 |
| pyrazinamide | tyloxapol | 0.730 | 0.903 |
| benperidol | betahistine | 0.730 | 0.745 |
| niclosamide | nortriptyline | 0.730 | 0.701 |
| chloramphenicol | flufenamic_acid | 0.730 | 0.709 |
| pyrvinium | sulconazole | 0.730 | 0.745 |
| irinotecan | lomustine | 0.730 | 0.795 |
| bromocriptine | mepacrine | 0.730 | 0.713 |
| amrinone | sulfanilamide | 0.730 | 0.655 |
| clioquinol | suloctidil | 0.730 | 0.736 |
| ketanserin | methylergometrine | 0.731 | 0.679 |
| alimemazine | loperamide | 0.731 | 0.767 |
| ivermectin | prochlorperazine | 0.731 | 0.824 |
| etomidate | levamisole | 0.731 | 0.668 |
| propylthiouracil | sulconazole | 0.731 | 0.770 |
| doxazosin | fluspirilene | 0.731 | 0.697 |
| metaraminol | nalidixic_acid | 0.731 | 0.683 |
| bromperidol | trimetazidine | 0.731 | 0.758 |
| ambroxol | isoniazid | 0.731 | 0.759 |
| doxazosin | zomepirac | 0.731 | 0.705 |
| clozapine | loperamide | 0.731 | 0.773 |
| budesonide | sulconazole | 0.731 | 0.775 |
| doxazosin | propofol | 0.731 | 0.790 |
| felodipine | propofol | 0.731 | 0.761 |
| fluphenazine | prenylamine | 0.731 | 0.739 |
| cyproterone | doxazosin | 0.731 | 0.732 |
| aminocaproic_acid | proxyphylline | 0.731 | 0.682 |
| perphenazine | phenoxybenzamine | 0.731 | 0.750 |
| etidronic_acid | roxithromycin | 0.731 | 0.887 |
| suloctidil | zuclopenthixol | 0.731 | 0.762 |
| etofenamate | meticrane | 0.731 | 0.715 |
| estriol | glibenclamide | 0.731 | 0.767 |
| metacycline | triflusal | 0.731 | 0.751 |
| decitabine | isocarboxazid | 0.731 | 0.680 |
| erastin | loperamide | 0.731 | 0.690 |
| cyproterone | doxorubicin | 0.731 | 0.725 |
| desipramine | mometasone | 0.731 | 0.775 |
| diltiazem | piperidolate | 0.731 | 0.690 |

| | | | |
|---|---|---|---|
| propylthiouracil | repaglinide | 0.731 | 0.769 |
| benzethonium_chloride | rescinnamine | 0.731 | 0.749 |
| meptazinol | oxybuprocaine | 0.731 | 0.758 |
| medrysone | naloxone | 0.731 | 0.706 |
| cefsulodin | propylthiouracil | 0.731 | 0.797 |
| astemizole | oxyphenbutazone | 0.731 | 0.715 |
| fenoprofen | monobenzone | 0.731 | 0.657 |
| benfluorex | demecolcine | 0.731 | 0.731 |
| metergoline | proscillaridin | 0.731 | 0.756 |
| meticrane | phenindione | 0.731 | 0.721 |
| cyproterone | sertaconazole | 0.731 | 0.733 |
| ceforanide | ergocalciferol | 0.731 | 0.750 |
| levonorgestrel | roxithromycin | 0.731 | 0.830 |
| felbinac | nadolol | 0.732 | 0.713 |
| bromopride | verteporfin | 0.732 | 0.790 |
| deptropine | monobenzone | 0.732 | 0.688 |
| irinotecan | trifluridine | 0.732 | 0.766 |
| levomepromazine | terfenadine | 0.732 | 0.756 |
| astemizole | clomifene | 0.732 | 0.750 |
| tolfenamic_acid | tranylcypromine | 0.732 | 0.671 |
| fenbendazole | lomustine | 0.732 | 0.689 |
| clemastine | terfenadine | 0.732 | 0.729 |
| dacarbazine | dipyridamole | 0.732 | 0.775 |
| milrinone | trimetazidine | 0.732 | 0.718 |
| metixene | prochlorperazine | 0.732 | 0.691 |
| sulconazole | sulfamethoxazole | 0.732 | 0.750 |
| doxorubicin | rolitetracycline | 0.732 | 0.671 |
| deptropine | pyrvinium | 0.732 | 0.789 |
| gliclazide | tyloxapol | 0.732 | 0.863 |
| buflomedil | rimexolone | 0.732 | 0.750 |
| fipexide | vinpocetine | 0.732 | 0.764 |
| ethosuximide | pargyline | 0.732 | 0.747 |
| oxetacaine | sulconazole | 0.732 | 0.720 |
| mebendazole | salbutamol | 0.732 | 0.654 |
| dacarbazine | natamycin | 0.732 | 0.849 |
| medrysone | scopolamine | 0.732 | 0.667 |
| doxazosin | trimetazidine | 0.732 | 0.751 |
| meticrane | piribedil | 0.732 | 0.752 |
| procarbazine | trifluoperazine | 0.732 | 0.713 |
| carbachol | verteporfin | 0.732 | 0.867 |
| benzocaine | nystatin | 0.732 | 0.910 |
| ciclopirox | metixene | 0.732 | 0.701 |
| meropenem | ticarcillin | 0.732 | 0.708 |
| cetirizine | meticrane | 0.732 | 0.776 |
| digoxin | metixene | 0.732 | 0.868 |
| cetirizine | dydrogesterone | 0.732 | 0.756 |
| disulfiram | etacrynic_acid | 0.732 | 0.740 |
| methazolamide | rimexolone | 0.732 | 0.781 |
| perphenazine | quinisocaine | 0.732 | 0.679 |
| colchicine | trifluoperazine | 0.732 | 0.675 |

| | | | |
|---|---|---|---|
| cetirizine | etofenamate | 0.732 | 0.692 |
| liothyronine | promazine | 0.732 | 0.744 |
| dipyridamole | nitrofurantoin | 0.732 | 0.740 |
| benperidol | galantamine | 0.732 | 0.787 |
| gliclazide | talampicillin | 0.732 | 0.702 |
| erastin | trifluoperazine | 0.732 | 0.731 |
| ivermectin | miconazole | 0.733 | 0.847 |
| spectinomycin | trichlormethiazide | 0.733 | 0.700 |
| mebendazole | menadione | 0.733 | 0.664 |
| cetirizine | griseofulvin | 0.733 | 0.755 |
| capsaicin | metyrapone | 0.733 | 0.735 |
| benperidol | cetirizine | 0.733 | 0.714 |
| propylthiouracil | rimexolone | 0.733 | 0.754 |
| haloperidol | niclosamide | 0.733 | 0.696 |
| cefsulodin | doxazosin | 0.733 | 0.709 |
| ciclosporin | phenoxybenzamine | 0.733 | 0.894 |
| benfluorex | pyrvinium | 0.733 | 0.785 |
| doxazosin | oxetacaine | 0.733 | 0.704 |
| methotrexate | oxamic_acid | 0.733 | 0.851 |
| latamoxef | pyrvinium | 0.733 | 0.801 |
| econazole | fendiline | 0.733 | 0.716 |
| fluphenazine | pimozide | 0.733 | 0.742 |
| clotrimazole | lomustine | 0.733 | 0.767 |
| ketanserin | pentoxyverine | 0.733 | 0.738 |
| perhexiline | pimozide | 0.733 | 0.785 |
| fluphenazine | maprotiline | 0.733 | 0.739 |
| molsidomine | phenindione | 0.733 | 0.729 |
| flupentixol | mometasone | 0.733 | 0.729 |
| butoconazole | suloctidil | 0.733 | 0.745 |
| flufenamic_acid | piperidolate | 0.733 | 0.726 |
| chlorphenamine | mefloquine | 0.733 | 0.749 |
| beclometasone | piromidic_acid | 0.733 | 0.756 |
| chlorzoxazone | natamycin | 0.733 | 0.885 |
| mebendazole | pimozide | 0.733 | 0.719 |
| disulfiram | semustine | 0.733 | 0.671 |
| propafenone | triflusal | 0.733 | 0.747 |
| erastin | syrosingopine | 0.733 | 0.739 |
| isoconazole | nomifensine | 0.733 | 0.677 |
| liothyronine | molindone | 0.733 | 0.702 |
| imipramine | prochlorperazine | 0.733 | 0.667 |
| etodolac | oxolinic_acid | 0.733 | 0.654 |
| meptazinol | tyloxapol | 0.733 | 0.885 |
| rimexolone | sulfametoxydiazine | 0.733 | 0.750 |
| fluspirilene | reserpine | 0.733 | 0.746 |
| meclozine | naloxone | 0.733 | 0.775 |
| protriptyline | thioproperazine | 0.733 | 0.742 |
| etomidate | imipenem | 0.733 | 0.706 |
| astemizole | etacrynic_acid | 0.733 | 0.748 |
| ivermectin | nortriptyline | 0.733 | 0.879 |
| perphenazine | rescinnamine | 0.733 | 0.752 |

| | | | |
|---|---|---|---|
| econazole | perhexiline | 0.733 | 0.704 |
| mefloquine | methylergometrine | 0.734 | 0.706 |
| chlorprothixene | fluphenazine | 0.734 | 0.660 |
| desipramine | fluphenazine | 0.734 | 0.658 |
| ambroxol | nitrofural | 0.734 | 0.701 |
| demecolcine | promethazine | 0.734 | 0.725 |
| levomepromazine | protriptyline | 0.734 | 0.658 |
| albendazole | methylergometrine | 0.734 | 0.690 |
| propofol | verteporfin | 0.734 | 0.864 |
| celecoxib | imatinib | 0.734 | 0.760 |
| loxapine | niclosamide | 0.734 | 0.662 |
| oxyphenbutazone | podophyllotoxin | 0.734 | 0.734 |
| chlorzoxazone | pargyline | 0.734 | 0.692 |
| metixene | proscillaridin | 0.734 | 0.819 |
| fluvastatin | nicotinic_acid | 0.734 | 0.792 |
| cefsulodin | liothyronine | 0.734 | 0.741 |
| glibenclamide | sulconazole | 0.734 | 0.780 |
| nabumetone | proxymetacaine | 0.734 | 0.693 |
| dilazep | salbutamol | 0.734 | 0.792 |
| tobramycin | trichlormethiazide | 0.734 | 0.744 |
| azacitidine | sulconazole | 0.734 | 0.775 |
| ciclosporin | protriptyline | 0.734 | 0.901 |
| doxazosin | ganciclovir | 0.734 | 0.675 |
| nalbuphine | triamcinolone | 0.734 | 0.714 |
| astemizole | azacitidine | 0.734 | 0.794 |
| betaxolol | gefitinib | 0.734 | 0.741 |
| primaquine | proscillaridin | 0.734 | 0.795 |
| oxamniquine | piribedil | 0.734 | 0.695 |
| fendiline | ivermectin | 0.734 | 0.863 |
| meptazinol | tiapride | 0.734 | 0.754 |
| amiodarone | rimexolone | 0.734 | 0.769 |
| bepridil | syrosingopine | 0.734 | 0.802 |
| gliclazide | propylthiouracil | 0.734 | 0.763 |
| etamsylate | talampicillin | 0.734 | 0.782 |
| metoprolol | sulfapyridine | 0.734 | 0.665 |
| bromocriptine | tetryzoline | 0.734 | 0.805 |
| benperidol | calcium_pantothenate | 0.734 | 0.767 |
| triflusal | trimetazidine | 0.734 | 0.718 |
| meclozine | roxithromycin | 0.734 | 0.836 |
| bisacodyl | doxazosin | 0.735 | 0.760 |
| diltiazem | dydrogesterone | 0.735 | 0.758 |
| estriol | sulfametoxydiazine | 0.735 | 0.706 |
| isoconazole | trifluoperazine | 0.735 | 0.709 |
| omeprazole | repaglinide | 0.735 | 0.740 |
| gliclazide | trazodone | 0.735 | 0.680 |
| calcium_pantothenate | cefixime | 0.735 | 0.756 |
| daunorubicin | sulfametoxydiazine | 0.735 | 0.733 |
| etofenamate | metamizole_sodium | 0.735 | 0.656 |
| phenoxybenzamine | tyloxapol | 0.735 | 0.881 |
| clioquinol | etacrynic_acid | 0.735 | 0.686 |

| | | | |
|---|---|---|---|
| bisacodyl | doxorubicin | 0.735 | 0.760 |
| etacrynic_acid | fluspirilene | 0.735 | 0.730 |
| flupentixol | promethazine | 0.735 | 0.651 |
| piperidolate | sulfametoxydiazine | 0.735 | 0.707 |
| naloxone | oleandomycin | 0.735 | 0.810 |
| astemizole | demecolcine | 0.735 | 0.756 |
| meclofenoxate | meticrane | 0.735 | 0.707 |
| fenoterol | remoxipride | 0.735 | 0.693 |
| cinchocaine | gliclazide | 0.735 | 0.710 |
| bufexamac | doxepin | 0.735 | 0.743 |
| chlorzoxazone | methazolamide | 0.735 | 0.669 |
| medrysone | piromidic_acid | 0.735 | 0.697 |
| benfotiamine | spiramycin | 0.735 | 0.784 |
| flucytosine | urapidil | 0.735 | 0.710 |
| etacrynic_acid | perhexiline | 0.735 | 0.776 |
| albendazole | mefloquine | 0.735 | 0.726 |
| cetirizine | piribedil | 0.735 | 0.731 |
| reserpine | simvastatin | 0.735 | 0.756 |
| flurbiprofen | urapidil | 0.735 | 0.728 |
| piperidolate | talampicillin | 0.735 | 0.731 |
| prenylamine | quinisocaine | 0.735 | 0.694 |
| clomifene | miconazole | 0.735 | 0.744 |
| irinotecan | zimeldine | 0.735 | 0.780 |
| cefalotin | trazodone | 0.735 | 0.715 |
| cetirizine | repaglinide | 0.735 | 0.690 |
| lomustine | menadione | 0.735 | 0.674 |
| econazole | suloctidil | 0.735 | 0.728 |
| fenoprofen | sulfametoxydiazine | 0.735 | 0.693 |
| meticrane | tranylcypromine | 0.735 | 0.684 |
| gliclazide | roxithromycin | 0.735 | 0.826 |
| benzylpenicillin | doxazosin | 0.735 | 0.724 |
| chlorzoxazone | sulfametoxydiazine | 0.735 | 0.708 |
| amiodarone | cefalexin | 0.735 | 0.735 |
| carbenoxolone | tolmetin | 0.735 | 0.782 |
| pentoxyverine | promethazine | 0.735 | 0.762 |
| latamoxef | omeprazole | 0.735 | 0.721 |
| perhexiline | perphenazine | 0.735 | 0.751 |
| midecamycin | zimeldine | 0.735 | 0.891 |
| clomifene | suloctidil | 0.735 | 0.732 |
| amikacin | methylergometrine | 0.735 | 0.772 |
| lanatoside_C | metixene | 0.736 | 0.886 |
| flufenamic_acid | medrysone | 0.736 | 0.714 |
| alfaxalone | proxymetacaine | 0.736 | 0.729 |
| milrinone | vidarabine | 0.736 | 0.687 |
| benzylpenicillin | piperidolate | 0.736 | 0.711 |
| norethisterone | piribedil | 0.736 | 0.740 |
| aminohippuric_acid | nitrendipine | 0.736 | 0.732 |
| doxylamine | nalidixic_acid | 0.736 | 0.745 |
| fendiline | mepacrine | 0.736 | 0.748 |
| buflomedil | scopolamine | 0.736 | 0.697 |

| | | | |
|---|---|---|---|
| amoxapine | metergoline | 0.736 | 0.743 |
| piperidolate | tyloxapol | 0.736 | 0.859 |
| niclosamide | quinisocaine | 0.736 | 0.686 |
| benzethonium_chloride | chlorprothixene | 0.736 | 0.771 |
| gliclazide | ronidazole | 0.736 | 0.726 |
| methylergometrine | perhexiline | 0.736 | 0.755 |
| hexetidine | podophyllotoxin | 0.736 | 0.735 |
| gliclazide | menadione | 0.736 | 0.761 |
| loxapine | perphenazine | 0.736 | 0.737 |
| amoxapine | fluspirilene | 0.736 | 0.775 |
| cycloserine | medrysone | 0.736 | 0.929 |
| acetylsalicylic_acid | doxazosin | 0.736 | 0.793 |
| repaglinide | tropicamide | 0.736 | 0.753 |
| latamoxef | pentoxyverine | 0.736 | 0.741 |
| fluvastatin | sulconazole | 0.736 | 0.745 |
| doxorubicin | piromidic_acid | 0.736 | 0.698 |
| irinotecan | medrysone | 0.736 | 0.771 |
| cefalotin | tyloxapol | 0.736 | 0.846 |
| chlorprothixene | perhexiline | 0.736 | 0.706 |
| oxprenolol | trioxysalen | 0.736 | 0.720 |
| chloramphenicol | doxazosin | 0.736 | 0.746 |
| fenoprofen | piperidolate | 0.736 | 0.721 |
| promethazine | pyrvinium | 0.736 | 0.793 |
| promethazine | zimeldine | 0.736 | 0.652 |
| etamsylate | oxprenolol | 0.736 | 0.724 |
| ioxaglic_acid | spaglumic_acid | 0.736 | 0.808 |
| dacarbazine | demecolcine | 0.736 | 0.697 |
| iopanoic_acid | rimexolone | 0.736 | 0.742 |
| diltiazem | promazine | 0.736 | 0.709 |
| albendazole | mepacrine | 0.736 | 0.681 |
| etofenamate | sulfametoxydiazine | 0.736 | 0.716 |
| ipratropium_bromide | roxithromycin | 0.736 | 0.821 |
| loperamide | noretynodrel | 0.736 | 0.748 |
| chlorprothixene | rescinnamine | 0.736 | 0.788 |
| fluspirilene | syrosingopine | 0.736 | 0.767 |
| carbachol | liothyronine | 0.736 | 0.771 |
| cefixime | sulconazole | 0.736 | 0.769 |
| clotrimazole | meclozine | 0.736 | 0.698 |
| ciclopirox | felodipine | 0.737 | 0.738 |
| bromocriptine | meclozine | 0.737 | 0.741 |
| albendazole | mepyramine | 0.737 | 0.736 |
| ketotifen | streptomycin | 0.737 | 0.807 |
| desipramine | disulfiram | 0.737 | 0.755 |
| chlorzoxazone | verteporfin | 0.737 | 0.859 |
| piribedil | promethazine | 0.737 | 0.747 |
| glibenclamide | sulfametoxydiazine | 0.737 | 0.717 |
| metyrapone | triflusal | 0.737 | 0.701 |
| loperamide | progesterone | 0.737 | 0.760 |
| estriol | ipratropium_bromide | 0.737 | 0.728 |
| fenoprofen | latamoxef | 0.737 | 0.779 |

| | | | |
|---|---|---|---|
| desipramine | loxapine | 0.737 | 0.652 |
| bromocriptine | rescinnamine | 0.737 | 0.718 |
| bisacodyl | glibenclamide | 0.737 | 0.751 |
| deptropine | roxithromycin | 0.737 | 0.842 |
| biotin | profenamine | 0.737 | 0.780 |
| dacarbazine | mitoxantrone | 0.737 | 0.741 |
| bromopride | doxazosin | 0.737 | 0.695 |
| piribedil | pyrvinium | 0.737 | 0.728 |
| cetirizine | latamoxef | 0.737 | 0.750 |
| metamizole_sodium | verteporfin | 0.737 | 0.785 |
| astemizole | pizotifen | 0.737 | 0.771 |
| ciclopirox | fluspirilene | 0.737 | 0.754 |
| lomefloxacin | sulfamethoxypyridazir | 0.737 | 0.677 |
| clomifene | pyrvinium | 0.737 | 0.747 |
| clomifene | niclosamide | 0.737 | 0.734 |
| bepridil | propafenone | 0.737 | 0.714 |
| levonorgestrel | meticrane | 0.737 | 0.760 |
| acetazolamide | triflupromazine | 0.737 | 0.672 |
| cefsulodin | procyclidine | 0.737 | 0.801 |
| saquinavir | sertaconazole | 0.737 | 0.770 |
| felodipine | verteporfin | 0.737 | 0.823 |
| lymecycline | rimexolone | 0.737 | 0.778 |
| capsaicin | liothyronine | 0.737 | 0.754 |
| bisacodyl | flufenamic_acid | 0.737 | 0.730 |
| meclofenoxate | mitoxantrone | 0.737 | 0.751 |
| ciclopirox | protriptyline | 0.737 | 0.702 |
| amiodarone | omeprazole | 0.737 | 0.734 |
| desoxycortone | diltiazem | 0.737 | 0.757 |
| etomidate | triflusal | 0.737 | 0.689 |
| cetirizine | demecolcine | 0.737 | 0.763 |
| benfluorex | deptropine | 0.737 | 0.765 |
| methapyrilene | omeprazole | 0.737 | 0.731 |
| irinotecan | primaquine | 0.737 | 0.703 |
| rimexolone | xylometazoline | 0.737 | 0.752 |
| benzethonium_chloride | thioridazine | 0.737 | 0.777 |
| bisacodyl | medrysone | 0.737 | 0.746 |
| iocetamic_acid | paromomycin | 0.737 | 0.812 |
| pyrazinamide | trioxysalen | 0.737 | 0.724 |
| benzethonium_chloride | methylergometrine | 0.737 | 0.762 |
| meptazinol | roxithromycin | 0.738 | 0.872 |
| ciclosporin | estrone | 0.738 | 0.890 |
| mesoridazine | talampicillin | 0.738 | 0.756 |
| disulfiram | hexetidine | 0.738 | 0.750 |
| cyproheptadine | miconazole | 0.738 | 0.703 |
| cefalotin | etamsylate | 0.738 | 0.762 |
| norethisterone | repaglinide | 0.738 | 0.753 |
| monobenzone | practolol | 0.738 | 0.712 |
| piribedil | tribenoside | 0.738 | 0.744 |
| nitrofurantoin | verteporfin | 0.738 | 0.812 |
| cetirizine | triflusal | 0.738 | 0.785 |

| | | | |
|---|---|---|---|
| betaxolol | oxamic_acid | 0.738 | 0.864 |
| triflusal | verteporfin | 0.738 | 0.843 |
| flumetasone | levonorgestrel | 0.738 | 0.673 |
| benfluorex | nilutamide | 0.738 | 0.736 |
| rimexolone | zimeldine | 0.738 | 0.741 |
| dexibuprofen | methylergometrine | 0.738 | 0.718 |
| buflomedil | pentoxyverine | 0.738 | 0.705 |
| artemisinin | chloroquine | 0.738 | 0.776 |
| bromperidol | medrysone | 0.738 | 0.742 |
| menadione | vorinostat | 0.738 | 0.704 |
| benzonatate | imipramine | 0.738 | 0.832 |
| etacrynic_acid | phenazopyridine | 0.738 | 0.697 |
| etamsylate | ronidazole | 0.738 | 0.661 |
| chlorprothixene | hexetidine | 0.738 | 0.759 |
| meclofenoxate | tropicamide | 0.738 | 0.685 |
| dacarbazine | fenoprofen | 0.738 | 0.698 |
| doxazosin | etofenamate | 0.738 | 0.724 |
| chlorprothixene | lanatoside_C | 0.738 | 0.896 |
| cyproterone | roxithromycin | 0.738 | 0.817 |
| isoconazole | pyrvinium | 0.738 | 0.765 |
| monobenzone | omeprazole | 0.738 | 0.718 |
| doxorubicin | irinotecan | 0.738 | 0.705 |
| capsaicin | tyloxapol | 0.738 | 0.864 |
| glibenclamide | meptazinol | 0.738 | 0.815 |
| astemizole | proscillaridin | 0.738 | 0.762 |
| bromocriptine | perphenazine | 0.738 | 0.697 |
| bromperidol | dirithromycin | 0.738 | 0.803 |
| metamizole_sodium | piperidolate | 0.738 | 0.674 |
| butoconazole | terfenadine | 0.738 | 0.756 |
| doxorubicin | scopolamine | 0.738 | 0.747 |
| astemizole | pimethixene | 0.738 | 0.752 |
| fluorometholone | sulconazole | 0.738 | 0.767 |
| ambroxol | enoxacin | 0.738 | 0.655 |
| bromperidol | sulfametoxydiazine | 0.738 | 0.691 |
| niclosamide | zuclopenthixol | 0.738 | 0.680 |
| benzylpenicillin | trioxysalen | 0.738 | 0.747 |
| cetirizine | sulfamethoxypyridazir | 0.738 | 0.726 |
| metamizole_sodium | tobramycin | 0.738 | 0.756 |
| cinchocaine | diltiazem | 0.738 | 0.712 |
| fluspirilene | perhexiline | 0.738 | 0.740 |
| chlorprothixene | miconazole | 0.738 | 0.720 |
| atovaquone | doxazosin | 0.738 | 0.660 |
| cyanocobalamin | sparteine | 0.738 | 0.919 |
| profenamine | propofol | 0.738 | 0.738 |
| clioquinol | digoxin | 0.739 | 0.883 |
| carbachol | talampicillin | 0.739 | 0.812 |
| etacrynic_acid | nortriptyline | 0.739 | 0.712 |
| fluphenazine | fluspirilene | 0.739 | 0.714 |
| metergoline | oxetacaine | 0.739 | 0.691 |
| chlorprothixene | isoconazole | 0.739 | 0.660 |

| | | | |
|---|---|---|---|
| isosorbide | pramocaine | 0.739 | 0.792 |
| clomifene | loperamide | 0.739 | 0.742 |
| astemizole | tamoxifen | 0.739 | 0.753 |
| cefalotin | estriol | 0.739 | 0.756 |
| altretamine | tyloxapol | 0.739 | 0.886 |
| etomidate | rimexolone | 0.739 | 0.754 |
| acepromazine | desoxycortone | 0.739 | 0.727 |
| mepyramine | oxytetracycline | 0.739 | 0.747 |
| etoposide | mepacrine | 0.739 | 0.738 |
| cefalotin | repaglinide | 0.739 | 0.733 |
| acepromazine | verteporfin | 0.739 | 0.750 |
| promazine | verteporfin | 0.739 | 0.777 |
| felodipine | suloctidil | 0.739 | 0.749 |
| clindamycin | meptazinol | 0.739 | 0.790 |
| decitabine | triamcinolone | 0.739 | 0.737 |
| disulfiram | mebendazole | 0.739 | 0.703 |
| felodipine | terfenadine | 0.739 | 0.763 |
| doxorubicin | nitrofurantoin | 0.739 | 0.730 |
| nicergoline | spironolactone | 0.739 | 0.765 |
| protriptyline | syrosingopine | 0.739 | 0.814 |
| bisacodyl | niclosamide | 0.739 | 0.748 |
| latamoxef | tyloxapol | 0.739 | 0.824 |
| clioquinol | loperamide | 0.739 | 0.780 |
| nadolol | trihexyphenidyl | 0.739 | 0.734 |
| clomipramine | perhexiline | 0.739 | 0.669 |
| diazoxide | nalbuphine | 0.739 | 0.757 |
| meticrane | trazodone | 0.739 | 0.781 |
| diltiazem | dipyridamole | 0.739 | 0.766 |
| deptropine | doxazosin | 0.739 | 0.812 |
| etofylline | flufenamic_acid | 0.739 | 0.653 |
| acepromazine | latamoxef | 0.739 | 0.790 |
| dextromethorphan | sulconazole | 0.739 | 0.732 |
| benfluorex | suloctidil | 0.739 | 0.703 |
| fluspirilene | gliclazide | 0.739 | 0.686 |
| colchicine | pyrvinium | 0.739 | 0.773 |
| cinchocaine | zimeldine | 0.739 | 0.710 |
| cefalexin | diltiazem | 0.739 | 0.714 |
| trioxysalen | verteporfin | 0.739 | 0.839 |
| sulfametoxydiazine | trazodone | 0.739 | 0.690 |
| cefalexin | xylometazoline | 0.739 | 0.762 |
| sertaconazole | verteporfin | 0.739 | 0.782 |
| meticrane | promethazine | 0.739 | 0.724 |
| pyrazinamide | sulfametoxydiazine | 0.739 | 0.677 |
| iopanoic_acid | talampicillin | 0.739 | 0.784 |
| flucytosine | hydrocortisone | 0.739 | 0.841 |
| astemizole | reserpine | 0.739 | 0.726 |
| cycloserine | repaglinide | 0.739 | 0.908 |
| doxorubicin | trioxysalen | 0.739 | 0.782 |
| fenoterol | flavoxate | 0.739 | 0.700 |
| doxazosin | galantamine | 0.739 | 0.770 |

| | | | |
|---|---|---|---|
| cefotaxime | triamcinolone | 0.739 | 0.716 |
| ioversol | sulconazole | 0.739 | 0.795 |
| natamycin | triflusal | 0.739 | 0.842 |
| beclometasone | terconazole | 0.740 | 0.753 |
| fendiline | gefitinib | 0.740 | 0.777 |
| roxithromycin | zimeldine | 0.740 | 0.854 |
| mesalazine | repaglinide | 0.740 | 0.725 |
| chlorzoxazone | doxazosin | 0.740 | 0.774 |
| nafcillin | suprofen | 0.740 | 0.679 |
| cefalexin | minaprine | 0.740 | 0.704 |
| mefloquine | naltrexone | 0.740 | 0.701 |
| perphenazine | pimozide | 0.740 | 0.715 |
| bromperidol | piracetam | 0.740 | 0.772 |
| etofenamate | medrysone | 0.740 | 0.734 |
| cetirizine | metamizole_sodium | 0.740 | 0.721 |
| irinotecan | terfenadine | 0.740 | 0.751 |
| benzethonium_chloride | etacrynic_acid | 0.740 | 0.761 |
| protriptyline | proxymetacaine | 0.740 | 0.773 |
| cefixime | primaquine | 0.740 | 0.697 |
| fenspiride | meticrane | 0.740 | 0.751 |
| medrysone | trazodone | 0.740 | 0.753 |
| clemastine | metergoline | 0.740 | 0.738 |
| ambroxol | cefotiam | 0.740 | 0.775 |
| nalidixic_acid | sulfamethoxazole | 0.740 | 0.678 |
| cefalexin | promethazine | 0.740 | 0.762 |
| doxazosin | nalidixic_acid | 0.740 | 0.713 |
| etofenamate | ronidazole | 0.740 | 0.712 |
| mebendazole | perhexiline | 0.740 | 0.783 |
| flufenamic_acid | meclozine | 0.740 | 0.782 |
| etofenamate | tyloxapol | 0.740 | 0.821 |
| astemizole | methylergometrine | 0.740 | 0.743 |
| liothyronine | sulfamethoxypyridazir | 0.740 | 0.711 |
| neomycin | pirenzepine | 0.740 | 0.805 |
| betaxolol | capsaicin | 0.740 | 0.706 |
| dihydroergocristine | podophyllotoxin | 0.740 | 0.752 |
| meticrane | tropicamide | 0.740 | 0.720 |
| meticrane | trioxysalen | 0.740 | 0.669 |
| latamoxef | triflusal | 0.740 | 0.800 |
| ciclopirox | trifluoperazine | 0.740 | 0.745 |
| medrysone | propylthiouracil | 0.740 | 0.730 |
| bromocriptine | phenoxybenzamine | 0.740 | 0.783 |
| sulfaphenazole | triflusal | 0.740 | 0.726 |
| cefalexin | pipemidic_acid | 0.740 | 0.666 |
| meclozine | phenoxybenzamine | 0.740 | 0.681 |
| roxithromycin | sulfamethoxypyridazir | 0.740 | 0.841 |
| (-)-atenolol | ioversol | 0.740 | 0.788 |
| rimexolone | sulfamethoxypyridazir | 0.740 | 0.726 |
| piribedil | ronidazole | 0.740 | 0.704 |
| doxazosin | mitoxantrone | 0.740 | 0.704 |
| fenoprofen | sulpiride | 0.740 | 0.700 |

| | | | |
|---|---|---|---|
| ivermectin | phenoxybenzamine | 0.740 | 0.861 |
| podophyllotoxin | vinblastine | 0.740 | 0.792 |
| bromocriptine | phenazopyridine | 0.740 | 0.784 |
| roxithromycin | trazodone | 0.740 | 0.837 |
| monobenzone | vidarabine | 0.740 | 0.719 |
| econazole | mometasone | 0.741 | 0.770 |
| aciclovir | timolol | 0.741 | 0.661 |
| etoposide | perphenazine | 0.741 | 0.731 |
| meclozine | medrysone | 0.741 | 0.761 |
| budesonide | clomipramine | 0.741 | 0.799 |
| benfluorex | ketanserin | 0.741 | 0.655 |
| astemizole | colchicine | 0.741 | 0.758 |
| etidronic_acid | piromidic_acid | 0.741 | 0.785 |
| dipivefrine | ribostamycin | 0.741 | 0.728 |
| gliclazide | reserpine | 0.741 | 0.724 |
| loperamide | propranolol | 0.741 | 0.733 |
| procyclidine | theophylline | 0.741 | 0.780 |
| ketanserin | xylometazoline | 0.741 | 0.768 |
| chlorzoxazone | trimetazidine | 0.741 | 0.727 |
| cetirizine | sulfametoxydiazine | 0.741 | 0.737 |
| roxithromycin | tyloxapol | 0.741 | 0.796 |
| etamsylate | sulfametoxydiazine | 0.741 | 0.659 |
| diltiazem | liothyronine | 0.741 | 0.712 |
| norfloxacin | sulconazole | 0.741 | 0.757 |
| doxylamine | meptazinol | 0.741 | 0.690 |
| (-)-catechin | chlorzoxazone | 0.741 | 0.722 |
| meticrane | procaine | 0.741 | 0.733 |
| bromperidol | phenindione | 0.741 | 0.720 |
| piromidic_acid | triflusal | 0.741 | 0.678 |
| chlortetracycline | diltiazem | 0.741 | 0.722 |
| bepridil | prochlorperazine | 0.741 | 0.716 |
| latamoxef | promethazine | 0.741 | 0.810 |
| fendiline | hexetidine | 0.741 | 0.707 |
| nifuroxazide | pyrvinium | 0.741 | 0.780 |
| promethazine | roxithromycin | 0.741 | 0.855 |
| cyproterone | triflusal | 0.741 | 0.771 |
| clioquinol | pyrvinium | 0.741 | 0.785 |
| chlorzoxazone | methylergometrine | 0.741 | 0.762 |
| dipyridamole | pentoxyverine | 0.741 | 0.781 |
| demecolcine | fenoprofen | 0.741 | 0.692 |
| citalopram | guaifenesin | 0.741 | 0.724 |
| chlorcyclizine | prochlorperazine | 0.741 | 0.667 |
| moxonidine | tobramycin | 0.741 | 0.803 |
| etacrynic_acid | perphenazine | 0.741 | 0.720 |
| clofazimine | trifluoperazine | 0.741 | 0.686 |
| demecolcine | xylometazoline | 0.741 | 0.748 |
| alfuzosin | meptazinol | 0.741 | 0.761 |
| trazodone | trioxysalen | 0.741 | 0.778 |
| ipratropium_bromide | triflusal | 0.741 | 0.754 |
| clomipramine | oxytetracycline | 0.741 | 0.782 |

| | | | |
|---|---|---|---|
| trifluoperazine | verapamil | 0.741 | 0.760 |
| fluvoxamine | loperamide | 0.741 | 0.763 |
| liothyronine | theobromine | 0.741 | 0.774 |
| perphenazine | raloxifene | 0.741 | 0.703 |
| sulfapyridine | tetryzoline | 0.741 | 0.698 |
| methazolamide | repaglinide | 0.741 | 0.763 |
| doxazosin | progesterone | 0.741 | 0.740 |
| deptropine | fenoprofen | 0.741 | 0.690 |
| ciclosporin | fenbendazole | 0.741 | 0.880 |
| cefalexin | naloxone | 0.741 | 0.747 |
| doxorubicin | estriol | 0.741 | 0.729 |
| clindamycin | molindone | 0.741 | 0.724 |
| phentolamine | promazine | 0.741 | 0.677 |
| ambroxol | tiapride | 0.741 | 0.702 |
| methoxamine | pyrazinamide | 0.741 | 0.652 |
| lomustine | loperamide | 0.741 | 0.747 |
| dipyridamole | repaglinide | 0.741 | 0.756 |
| betaxolol | ronidazole | 0.741 | 0.708 |
| ivermectin | suloctidil | 0.741 | 0.842 |
| chlorzoxazone | oxprenolol | 0.741 | 0.727 |
| clindamycin | talampicillin | 0.741 | 0.710 |
| cyproheptadine | perphenazine | 0.741 | 0.691 |
| clofazimine | gefitinib | 0.741 | 0.702 |
| doxepin | trifluoperazine | 0.741 | 0.667 |
| cefalexin | meclofenoxate | 0.741 | 0.662 |
| rifabutin | terfenadine | 0.741 | 0.788 |
| loperamide | syrosingopine | 0.741 | 0.763 |
| ronidazole | tyloxapol | 0.741 | 0.881 |
| liothyronine | scopolamine | 0.741 | 0.713 |
| pentoxyverine | sulfamethoxypyridazir | 0.741 | 0.715 |
| perhexiline | promazine | 0.741 | 0.684 |
| chloramphenicol | octopamine | 0.741 | 0.676 |
| glibenclamide | repaglinide | 0.741 | 0.728 |
| cefalotin | proscillaridin | 0.741 | 0.753 |
| acebutolol | gefitinib | 0.741 | 0.667 |
| clotrimazole | prenylamine | 0.741 | 0.669 |
| flupentixol | mefloquine | 0.741 | 0.691 |
| ketanserin | metamizole_sodium | 0.741 | 0.706 |
| fenoprofen | oxybuprocaine | 0.741 | 0.701 |
| bisacodyl | rimexolone | 0.741 | 0.746 |
| griseofulvin | nitrofurantoin | 0.742 | 0.706 |
| niclosamide | raloxifene | 0.742 | 0.741 |
| clomipramine | phenoxybenzamine | 0.742 | 0.677 |
| dydrogesterone | promethazine | 0.742 | 0.768 |
| fluocinonide | ketanserin | 0.742 | 0.734 |
| propofol | suloctidil | 0.742 | 0.787 |
| bisacodyl | propafenone | 0.742 | 0.740 |
| cinchocaine | repaglinide | 0.742 | 0.724 |
| sulfamethoxypyridazine | tropicamide | 0.742 | 0.660 |
| mephenytoin | tropine | 0.742 | 0.747 |

| | | | |
|---|---|---|---|
| imipenem | lymecycline | 0.742 | 0.779 |
| econazole | metergoline | 0.742 | 0.738 |
| metergoline | thioridazine | 0.742 | 0.718 |
| chlorphenesin | gefitinib | 0.742 | 0.736 |
| mitoxantrone | xylometazoline | 0.742 | 0.778 |
| calcium_pantothenate | propantheline_bromic | 0.742 | 0.791 |
| metaraminol | ofloxacin | 0.742 | 0.744 |
| levodopa | oxybuprocaine | 0.742 | 0.707 |
| amikacin | doxylamine | 0.742 | 0.825 |
| betamethasone | zidovudine | 0.742 | 0.720 |
| albendazole | labetalol | 0.742 | 0.703 |
| piperidolate | piromidic_acid | 0.742 | 0.732 |
| piperidolate | ramipril | 0.742 | 0.729 |
| sulfamethoxazole | tiapride | 0.742 | 0.693 |
| nitrofurantoin | talampicillin | 0.742 | 0.767 |
| artemisinin | piracetam | 0.742 | 0.736 |
| flufenamic_acid | trimetazidine | 0.742 | 0.664 |
| liothyronine | trimipramine | 0.742 | 0.740 |
| gliclazide | piracetam | 0.742 | 0.765 |
| dacarbazine | doxorubicin | 0.742 | 0.755 |
| etoposide | maprotiline | 0.742 | 0.796 |
| prochlorperazine | rescinnamine | 0.742 | 0.789 |
| propylthiouracil | sulfamethoxypyridazir | 0.742 | 0.692 |
| scopolamine | thiocolchicoside | 0.742 | 0.766 |
| triflusal | tropicamide | 0.742 | 0.725 |
| amitriptyline | rescinnamine | 0.742 | 0.812 |
| dihydroergotamine | mebendazole | 0.742 | 0.763 |
| maprotiline | mefloquine | 0.742 | 0.737 |
| budesonide | triflusal | 0.742 | 0.776 |
| liothyronine | repaglinide | 0.742 | 0.697 |
| tropicamide | verteporfin | 0.742 | 0.822 |
| metrifonate | sulfaphenazole | 0.742 | 0.736 |
| estriol | rimexolone | 0.742 | 0.711 |
| nitrofurantoin | repaglinide | 0.742 | 0.756 |
| apomorphine | roxithromycin | 0.742 | 0.871 |
| flufenamic_acid | repaglinide | 0.742 | 0.751 |
| indapamide | nalidixic_acid | 0.742 | 0.707 |
| bisacodyl | cinchocaine | 0.742 | 0.698 |
| estriol | gliclazide | 0.742 | 0.765 |
| levomepromazine | podophyllotoxin | 0.742 | 0.710 |
| molindone | pivampicillin | 0.742 | 0.763 |
| terfenadine | vorinostat | 0.742 | 0.747 |
| bepridil | cyproheptadine | 0.742 | 0.708 |
| budesonide | mesoridazine | 0.742 | 0.768 |
| metamizole_sodium | piribedil | 0.742 | 0.668 |
| carbachol | tyloxapol | 0.742 | 0.915 |
| oxyphenbutazone | terfenadine | 0.742 | 0.767 |
| ajmaline | chlortetracycline | 0.742 | 0.695 |
| glibenclamide | oxprenolol | 0.742 | 0.749 |
| halcinonide | sulconazole | 0.742 | 0.782 |

| | | | |
|---|---|---|---|
| amiodarone | latamoxef | 0.742 | 0.771 |
| galantamine | sulfathiazole | 0.742 | 0.695 |
| lymecycline | moracizine | 0.743 | 0.755 |
| prochlorperazine | suloctidil | 0.743 | 0.759 |
| fenoprofen | ronidazole | 0.743 | 0.694 |
| cefixime | omeprazole | 0.743 | 0.673 |
| nitrofurantoin | salbutamol | 0.743 | 0.677 |
| budesonide | repaglinide | 0.743 | 0.716 |
| hydralazine | levonorgestrel | 0.743 | 0.755 |
| clotrimazole | mifepristone | 0.743 | 0.733 |
| carbachol | propofol | 0.743 | 0.743 |
| mepacrine | rifabutin | 0.743 | 0.788 |
| chlorzoxazone | exemestane | 0.743 | 0.780 |
| sulfaphenazole | talampicillin | 0.743 | 0.737 |
| benzonatate | metrifonate | 0.743 | 0.872 |
| hydralazine | omeprazole | 0.743 | 0.723 |
| levamisole | ranitidine | 0.743 | 0.760 |
| bisacodyl | carbachol | 0.743 | 0.782 |
| amoxapine | terfenadine | 0.743 | 0.735 |
| gliclazide | piperidolate | 0.743 | 0.676 |
| cyanocobalamin | meticrane | 0.743 | 0.885 |
| bufexamac | syrosingopine | 0.743 | 0.806 |
| methylergometrine | rifabutin | 0.743 | 0.786 |
| bepridil | mometasone | 0.743 | 0.773 |
| amrinone | zomepirac | 0.743 | 0.686 |
| latamoxef | menadione | 0.743 | 0.826 |
| fenoprofen | triflusal | 0.743 | 0.692 |
| carbinoxamine | cefapirin | 0.743 | 0.782 |
| erastin | metergoline | 0.743 | 0.720 |
| cetirizine | propylthiouracil | 0.743 | 0.794 |
| amikacin | primidone | 0.743 | 0.847 |
| calcium_pantothenate | rimexolone | 0.743 | 0.776 |
| doxazosin | propafenone | 0.743 | 0.722 |
| scopolamine | succinylsulfathiazole | 0.743 | 0.718 |
| butoconazole | mefloquine | 0.743 | 0.752 |
| betahistine | medrysone | 0.743 | 0.776 |
| mometasone | nortriptyline | 0.743 | 0.779 |
| etoposide | lanatoside_C | 0.743 | 0.813 |
| dipyridamole | nabumetone | 0.743 | 0.725 |
| rimexolone | trimetazidine | 0.743 | 0.743 |
| methazolamide | sulconazole | 0.743 | 0.785 |
| reserpine | sulconazole | 0.743 | 0.782 |
| etamsylate | milrinone | 0.743 | 0.701 |
| levamisole | triflusal | 0.743 | 0.753 |
| mefloquine | syrosingopine | 0.743 | 0.782 |
| oxybutynin | vinpocetine | 0.743 | 0.719 |
| flunisolide | repaglinide | 0.743 | 0.722 |
| etofenamate | meptazinol | 0.743 | 0.756 |
| ketanserin | tribenoside | 0.743 | 0.738 |
| mepacrine | sirolimus | 0.743 | 0.840 |

| | | | |
|---|---|---|---|
| naftidrofuryl | profenamine | 0.743 | 0.732 |
| biotin | metixene | 0.743 | 0.769 |
| fendiline | maprotiline | 0.743 | 0.656 |
| butoconazole | ivermectin | 0.743 | 0.847 |
| liothyronine | sulfametoxydiazine | 0.743 | 0.711 |
| colchicine | promethazine | 0.743 | 0.700 |
| digoxin | fluspirilene | 0.743 | 0.812 |
| fenoprofen | metyrapone | 0.743 | 0.665 |
| dosulepin | mometasone | 0.743 | 0.777 |
| irinotecan | syrosingopine | 0.743 | 0.743 |
| gemfibrozil | ornidazole | 0.743 | 0.678 |
| clioquinol | miconazole | 0.743 | 0.727 |
| cyproheptadine | promethazine | 0.743 | 0.660 |
| chlorprothixene | ivermectin | 0.743 | 0.871 |
| disulfiram | prochlorperazine | 0.743 | 0.753 |
| prenylamine | propofol | 0.743 | 0.757 |
| metaraminol | trimethoprim | 0.744 | 0.727 |
| atovaquone | probenecid | 0.744 | 0.717 |
| cinchocaine | etamsylate | 0.744 | 0.744 |
| bisacodyl | zimeldine | 0.744 | 0.710 |
| prochlorperazine | proguanil | 0.744 | 0.740 |
| ciclosporin | erastin | 0.744 | 0.827 |
| hyoscyamine | tyloxapol | 0.744 | 0.871 |
| acetylsalicylic_acid | trazodone | 0.744 | 0.761 |
| metamizole_sodium | propylthiouracil | 0.744 | 0.719 |
| promethazine | trazodone | 0.744 | 0.770 |
| iopanoic_acid | pivampicillin | 0.744 | 0.784 |
| perphenazine | pyrvinium | 0.744 | 0.747 |
| ciclosporin | hexetidine | 0.744 | 0.870 |
| sulconazole | thiocolchicoside | 0.744 | 0.796 |
| (-)-catechin | glibenclamide | 0.744 | 0.753 |
| levamisole | norfloxacin | 0.744 | 0.719 |
| meptazinol | promethazine | 0.744 | 0.697 |
| hydrocortisone | scopolamine | 0.744 | 0.698 |
| amitriptyline | metergoline | 0.744 | 0.711 |
| prochlorperazine | rifabutin | 0.744 | 0.799 |
| erastin | terfenadine | 0.744 | 0.709 |
| propafenone | protriptyline | 0.744 | 0.730 |
| etofenamate | phenacetin | 0.744 | 0.730 |
| albendazole | colchicine | 0.744 | 0.699 |
| exemestane | loperamide | 0.744 | 0.776 |
| ambroxol | vigabatrin | 0.744 | 0.736 |
| cinchocaine | minaprine | 0.744 | 0.681 |
| dacarbazine | remoxipride | 0.744 | 0.734 |
| phenazopyridine | sulconazole | 0.744 | 0.695 |
| felodipine | prenylamine | 0.744 | 0.739 |
| hexetidine | levomepromazine | 0.744 | 0.748 |
| mebendazole | proscillaridin | 0.744 | 0.756 |
| buflomedil | cyanocobalamin | 0.744 | 0.872 |
| fluticasone | oxamniquine | 0.744 | 0.754 |

| | | | |
|---|---|---|---|
| betaxolol | rimexolone | 0.744 | 0.752 |
| piracetam | trimetazidine | 0.744 | 0.715 |
| menadione | repaglinide | 0.744 | 0.773 |
| cefalotin | progesterone | 0.744 | 0.751 |
| fluticasone | memantine | 0.744 | 0.806 |
| etacrynic_acid | fluphenazine | 0.744 | 0.710 |
| piromidic_acid | reserpine | 0.744 | 0.766 |
| aminohippuric_acid | amoxicillin | 0.744 | 0.705 |
| cyproheptadine | niclosamide | 0.744 | 0.735 |
| betahistine | chloramphenicol | 0.744 | 0.734 |
| acepromazine | ipratropium_bromide | 0.744 | 0.721 |
| moroxydine | promethazine | 0.744 | 0.737 |
| rimexolone | verteporfin | 0.744 | 0.805 |
| apomorphine | monobenzone | 0.744 | 0.661 |
| bumetanide | dyclonine | 0.744 | 0.747 |
| lanatoside_C | mometasone | 0.744 | 0.796 |
| metyrapone | promethazine | 0.744 | 0.707 |
| meclozine | sulfafurazole | 0.744 | 0.755 |
| fenbendazole | loperamide | 0.744 | 0.717 |
| diltiazem | minaprine | 0.744 | 0.731 |
| guanfacine | testosterone | 0.744 | 0.746 |
| piribedil | practolol | 0.744 | 0.693 |
| fenbendazole | semustine | 0.744 | 0.664 |
| monobenzone | promazine | 0.744 | 0.709 |
| fluspirilene | lanatoside_C | 0.744 | 0.844 |
| imipenem | rimexolone | 0.744 | 0.742 |
| fendiline | nortriptyline | 0.744 | 0.679 |
| diltiazem | phenoxybenzamine | 0.744 | 0.745 |
| carbachol | gliclazide | 0.745 | 0.767 |
| oxolamine | theobromine | 0.745 | 0.698 |
| dobutamine | practolol | 0.745 | 0.698 |
| bepridil | clioquinol | 0.745 | 0.768 |
| levomepromazine | mefloquine | 0.745 | 0.702 |
| fluphenazine | mometasone | 0.745 | 0.743 |
| benperidol | pyrazinamide | 0.745 | 0.757 |
| phenoxybenzamine | sulfametoxydiazine | 0.745 | 0.705 |
| meptazinol | zalcitabine | 0.745 | 0.709 |
| ronidazole | roxithromycin | 0.745 | 0.870 |
| acepromazine | trazodone | 0.745 | 0.742 |
| astemizole | noretynodrel | 0.745 | 0.766 |
| flupentixol | fluspirilene | 0.745 | 0.723 |
| bromperidol | promethazine | 0.745 | 0.780 |
| meclofenoxate | piperidolate | 0.745 | 0.652 |
| ambroxol | meptazinol | 0.745 | 0.739 |
| cefalexin | ketanserin | 0.745 | 0.686 |
| lymecycline | verteporfin | 0.745 | 0.782 |
| metergoline | vorinostat | 0.745 | 0.712 |
| deptropine | metergoline | 0.745 | 0.752 |
| cyclopenthiazide | primaquine | 0.745 | 0.729 |
| benzylpenicillin | estriol | 0.745 | 0.715 |

| | | | |
|---|---|---|---|
| natamycin | propylthiouracil | 0.745 | 0.856 |
| gliclazide | irinotecan | 0.745 | 0.761 |
| demecolcine | pyrvinium | 0.745 | 0.763 |
| repaglinide | trimetazidine | 0.745 | 0.757 |
| gliclazide | tropicamide | 0.745 | 0.719 |
| milrinone | oxprenolol | 0.745 | 0.734 |
| etamsylate | natamycin | 0.745 | 0.866 |
| imipenem | verteporfin | 0.745 | 0.783 |
| doxorubicin | gliclazide | 0.745 | 0.735 |
| perhexiline | trazodone | 0.745 | 0.788 |
| menadione | trifluoperazine | 0.745 | 0.729 |
| hyoscyamine | meptazinol | 0.745 | 0.742 |
| bromocriptine | rifabutin | 0.745 | 0.770 |
| carbamazepine | decitabine | 0.745 | 0.735 |
| meticrane | rimexolone | 0.745 | 0.713 |
| dipyridamole | phenoxybenzamine | 0.745 | 0.802 |
| riluzole | tiapride | 0.745 | 0.704 |
| etamsylate | sulfamethoxypyridazir | 0.745 | 0.659 |
| benfluorex | piribedil | 0.745 | 0.685 |
| cefalotin | triflusal | 0.745 | 0.749 |
| nabumetone | piribedil | 0.745 | 0.689 |
| fusidic_acid | prochlorperazine | 0.745 | 0.775 |
| bepridil | betaxolol | 0.745 | 0.757 |
| azacitidine | methotrexate | 0.745 | 0.726 |
| pentoxyverine | sulpiride | 0.745 | 0.718 |
| betahistine | promethazine | 0.745 | 0.699 |
| albendazole | clomipramine | 0.745 | 0.748 |
| bromopride | citiolone | 0.745 | 0.760 |
| etofenamate | piperidolate | 0.745 | 0.719 |
| chlorzoxazone | rimexolone | 0.745 | 0.793 |
| labetalol | terbutaline | 0.745 | 0.676 |
| pargyline | sulconazole | 0.745 | 0.725 |
| econazole | lomustine | 0.745 | 0.753 |
| pyrazinamide | tobramycin | 0.745 | 0.864 |
| desipramine | loperamide | 0.745 | 0.779 |
| mesoridazine | trimetazidine | 0.745 | 0.708 |
| piperidolate | saquinavir | 0.745 | 0.788 |
| albendazole | pyrvinium | 0.745 | 0.780 |
| cinchocaine | dipyridamole | 0.745 | 0.739 |
| benzylpenicillin | carbachol | 0.745 | 0.772 |
| levamisole | trazodone | 0.745 | 0.749 |
| chlorzoxazone | etomidate | 0.745 | 0.698 |
| fluspirilene | ticlopidine | 0.746 | 0.744 |
| albendazole | suloctidil | 0.746 | 0.719 |
| cycloserine | meticrane | 0.746 | 0.916 |
| isoxsuprine | sulfathiazole | 0.746 | 0.661 |
| medrysone | sulfametoxydiazine | 0.746 | 0.728 |
| fluoxetine | mefloquine | 0.746 | 0.678 |
| loperamide | oxamic_acid | 0.746 | 0.906 |
| clomipramine | verteporfin | 0.746 | 0.785 |

| | | | |
|---|---|---|---|
| doxazosin | imipenem | 0.746 | 0.726 |
| ipratropium_bromide | medrysone | 0.746 | 0.706 |
| ifenprodil | trazodone | 0.746 | 0.700 |
| menadione | metergoline | 0.746 | 0.775 |
| flupentixol | nortriptyline | 0.746 | 0.671 |
| estriol | medrysone | 0.746 | 0.692 |
| loperamide | saquinavir | 0.746 | 0.727 |
| benfluorex | clebopride | 0.746 | 0.680 |
| gliclazide | tobramycin | 0.746 | 0.749 |
| medrysone | trimetazidine | 0.746 | 0.731 |
| deptropine | gliclazide | 0.746 | 0.785 |
| benzethonium_chloride | phenazopyridine | 0.746 | 0.768 |
| neomycin | nomifensine | 0.746 | 0.855 |
| exemestane | verteporfin | 0.746 | 0.802 |
| metacycline | meticrane | 0.746 | 0.706 |
| cefalexin | ronidazole | 0.746 | 0.708 |
| hydralazine | lomefloxacin | 0.746 | 0.687 |
| benperidol | roxithromycin | 0.746 | 0.825 |
| niclosamide | thioridazine | 0.746 | 0.688 |
| decitabine | felbinac | 0.746 | 0.708 |
| hexetidine | raloxifene | 0.746 | 0.768 |
| aciclovir | podophyllotoxin | 0.746 | 0.729 |
| niclosamide | rescinnamine | 0.746 | 0.775 |
| ipratropium_bromide | tyloxapol | 0.746 | 0.854 |
| flunarizine | sulconazole | 0.746 | 0.724 |
| chlorcyclizine | suloctidil | 0.746 | 0.769 |
| carbachol | clomipramine | 0.746 | 0.793 |
| bromopride | triflusal | 0.746 | 0.738 |
| omeprazole | trioxysalen | 0.746 | 0.729 |
| enoxacin | meptazinol | 0.746 | 0.744 |
| cyproheptadine | diltiazem | 0.746 | 0.748 |
| idoxuridine | sulfathiazole | 0.746 | 0.675 |
| buflomedil | diltiazem | 0.746 | 0.721 |
| carbachol | oxantel | 0.746 | 0.733 |
| nortriptyline | oxetacaine | 0.746 | 0.778 |
| pentoxyverine | zimeldine | 0.746 | 0.735 |
| cetirizine | ipratropium_bromide | 0.746 | 0.741 |
| aminoglutethimide | doxepin | 0.746 | 0.736 |
| bepridil | clotrimazole | 0.746 | 0.666 |
| estriol | trazodone | 0.746 | 0.741 |
| cefalotin | menadione | 0.746 | 0.752 |
| repaglinide | sulfamethoxypyridazir | 0.746 | 0.748 |
| doxorubicin | monobenzone | 0.746 | 0.768 |
| glibenclamide | milrinone | 0.746 | 0.789 |
| meticrane | metyrapone | 0.746 | 0.703 |
| chlorprothixene | econazole | 0.746 | 0.691 |
| fipexide | gliclazide | 0.746 | 0.674 |
| mefloquine | metixene | 0.746 | 0.733 |
| astemizole | propofol | 0.746 | 0.811 |
| dacarbazine | piribedil | 0.746 | 0.721 |

| | | | |
|---|---|---|---|
| amoxicillin | sulconazole | 0.746 | 0.785 |
| amphotericin_B | butoconazole | 0.746 | 0.862 |
| miconazole | phenoxybenzamine | 0.746 | 0.718 |
| propylthiouracil | tropicamide | 0.746 | 0.710 |
| etofenamate | fluticasone | 0.746 | 0.755 |
| clomifene | procainamide | 0.746 | 0.743 |
| medrysone | propofol | 0.746 | 0.777 |
| dacarbazine | deptropine | 0.746 | 0.771 |
| pyrvinium | thioridazine | 0.746 | 0.757 |
| flunarizine | glafenine | 0.746 | 0.754 |
| chlorprothixene | loxapine | 0.746 | 0.656 |
| acetylsalicylic_acid | ursodeoxycholic_acid | 0.746 | 0.808 |
| diltiazem | triflusal | 0.746 | 0.756 |
| fendiline | miconazole | 0.746 | 0.714 |
| bisacodyl | dipyridamole | 0.746 | 0.742 |
| cycloserine | rimexolone | 0.746 | 0.870 |
| iopanoic_acid | remoxipride | 0.746 | 0.703 |
| dacarbazine | xylometazoline | 0.746 | 0.741 |
| fluvoxamine | meptazinol | 0.747 | 0.768 |
| ajmaline | clopamide | 0.747 | 0.715 |
| clioquinol | felodipine | 0.747 | 0.742 |
| gefitinib | trimetazidine | 0.747 | 0.744 |
| benzylpenicillin | triflusal | 0.747 | 0.736 |
| diltiazem | moroxydine | 0.747 | 0.778 |
| droperidol | prochlorperazine | 0.747 | 0.756 |
| tyloxapol | verteporfin | 0.747 | 0.796 |
| propafenone | rimexolone | 0.747 | 0.732 |
| dihydroergotamine | perphenazine | 0.747 | 0.735 |
| fluphenazine | labetalol | 0.747 | 0.726 |
| niclosamide | trimipramine | 0.747 | 0.749 |
| flufenamic_acid | roxithromycin | 0.747 | 0.840 |
| butoconazole | prenylamine | 0.747 | 0.713 |
| meticrane | nitrofurantoin | 0.747 | 0.727 |
| cinchocaine | tyloxapol | 0.747 | 0.851 |
| azacitidine | etofenamate | 0.747 | 0.722 |
| bisacodyl | metyrapone | 0.747 | 0.724 |
| dipyridamole | rimexolone | 0.747 | 0.748 |
| doxorubicin | levonorgestrel | 0.747 | 0.765 |
| carbachol | chloramphenicol | 0.747 | 0.762 |
| latamoxef | perhexiline | 0.747 | 0.812 |
| meptazinol | pentetrazol | 0.747 | 0.730 |
| medrysone | triflusal | 0.747 | 0.776 |
| medrysone | natamycin | 0.747 | 0.800 |
| cetirizine | verteporfin | 0.747 | 0.763 |
| sulconazole | vidarabine | 0.747 | 0.769 |
| bisacodyl | omeprazole | 0.747 | 0.739 |
| levamisole | tyloxapol | 0.747 | 0.902 |
| diazoxide | isoniazid | 0.747 | 0.694 |
| etacrynic_acid | quinisocaine | 0.747 | 0.692 |
| repaglinide | scopolamine | 0.747 | 0.735 |

| | | | |
|---|---|---|---|
| scopolamine | tolfenamic_acid | 0.747 | 0.693 |
| cycloserine | estriol | 0.747 | 1.000 |
| glibenclamide | molindone | 0.747 | 0.748 |
| rimexolone | rolitetracycline | 0.747 | 0.752 |
| lanatoside_C | podophyllotoxin | 0.747 | 0.854 |
| fenoprofen | tropicamide | 0.747 | 0.711 |
| acetylsalicylic_acid | norethisterone | 0.747 | 0.805 |
| meptazinol | metyrapone | 0.747 | 0.674 |
| propafenone | thiocolchicoside | 0.747 | 0.757 |
| econazole | fluspirilene | 0.747 | 0.755 |
| disulfiram | podophyllotoxin | 0.747 | 0.739 |
| etoposide | trifluoperazine | 0.747 | 0.744 |
| metyrapone | trioxysalen | 0.747 | 0.687 |
| calcium_pantothenate | sulconazole | 0.747 | 0.825 |
| meclozine | verteporfin | 0.747 | 0.810 |
| fendiline | mometasone | 0.747 | 0.764 |
| etacrynic_acid | raloxifene | 0.747 | 0.734 |
| tyloxapol | zomepirac | 0.747 | 0.857 |
| nilutamide | practolol | 0.747 | 0.678 |
| cefalexin | deptropine | 0.747 | 0.758 |
| doxylamine | idoxuridine | 0.747 | 0.730 |
| cycloserine | flufenamic_acid | 0.747 | 0.814 |
| colchicine | mefloquine | 0.747 | 0.709 |
| fluspirilene | ketanserin | 0.747 | 0.692 |
| roxithromycin | xylometazoline | 0.747 | 0.864 |
| quinisocaine | reserpine | 0.747 | 0.760 |
| clomipramine | trimetazidine | 0.747 | 0.699 |
| betahistine | diltiazem | 0.747 | 0.750 |
| flufenamic_acid | proxyphylline | 0.747 | 0.656 |
| disulfiram | methylergometrine | 0.747 | 0.708 |
| apomorphine | fluvastatin | 0.747 | 0.688 |
| famotidine | fluspirilene | 0.747 | 0.714 |
| reserpine | tobramycin | 0.747 | 0.764 |
| cyproheptadine | naloxone | 0.747 | 0.782 |
| calcium_folinate | isocarboxazid | 0.747 | 0.715 |
| mefloquine | miconazole | 0.747 | 0.746 |
| lansoprazole | lymecycline | 0.747 | 0.726 |
| hydrocortisone | meticrane | 0.747 | 0.716 |
| naloxone | tyloxapol | 0.747 | 0.860 |
| glibenclamide | piperidolate | 0.747 | 0.758 |
| dilazep | terfenadine | 0.748 | 0.771 |
| fenoprofen | tetryzoline | 0.748 | 0.690 |
| cinchocaine | medrysone | 0.748 | 0.736 |
| bisacodyl | suloctidil | 0.748 | 0.770 |
| nafcillin | tiapride | 0.748 | 0.686 |
| fipexide | sulfinpyrazone | 0.748 | 0.703 |
| glipizide | nilutamide | 0.748 | 0.754 |
| bisacodyl | miconazole | 0.748 | 0.726 |
| urapidil | verteporfin | 0.748 | 0.773 |
| mometasone | prochlorperazine | 0.748 | 0.755 |

| | | | |
|---|---|---|---|
| sulfamethoxypyridazine | trazodone | 0.748 | 0.712 |
| daunorubicin | menadione | 0.748 | 0.716 |
| methylergometrine | phenoxybenzamine | 0.748 | 0.709 |
| norethisterone | propylthiouracil | 0.748 | 0.746 |
| betahistine | meticrane | 0.748 | 0.691 |
| bromperidol | budesonide | 0.748 | 0.739 |
| flufenamic_acid | trazodone | 0.748 | 0.737 |
| haloperidol | terfenadine | 0.748 | 0.684 |
| fendiline | isoconazole | 0.748 | 0.692 |
| bromopride | repaglinide | 0.748 | 0.737 |
| norethisterone | trazodone | 0.748 | 0.783 |
| clomifene | ivermectin | 0.748 | 0.843 |
| aminohippuric_acid | meticrane | 0.748 | 0.694 |
| citiolone | sulconazole | 0.748 | 0.821 |
| betaxolol | zimeldine | 0.748 | 0.733 |
| astemizole | labetalol | 0.748 | 0.703 |
| daunorubicin | meticrane | 0.748 | 0.763 |
| buflomedil | gliclazide | 0.748 | 0.676 |
| fenoprofen | natamycin | 0.748 | 0.823 |
| galantamine | sulfamethoxazole | 0.748 | 0.716 |
| repaglinide | roxithromycin | 0.748 | 0.793 |
| calcium_pantothenate | scopolamine | 0.748 | 0.784 |
| exemestane | gefitinib | 0.748 | 0.735 |
| amiodarone | promethazine | 0.748 | 0.753 |
| liothyronine | metamizole_sodium | 0.748 | 0.664 |
| pentetrazol | pyrantel | 0.748 | 0.775 |
| cinoxacin | doxazosin | 0.748 | 0.688 |
| flufenamic_acid | rimexolone | 0.748 | 0.723 |
| promazine | protriptyline | 0.748 | 0.662 |
| amoxapine | sulconazole | 0.748 | 0.710 |
| doxorubicin | physostigmine | 0.748 | 0.783 |
| methapyrilene | practolol | 0.748 | 0.740 |
| chloramphenicol | flunarizine | 0.748 | 0.785 |
| protriptyline | suloctidil | 0.748 | 0.735 |
| hydrocortisone | repaglinide | 0.748 | 0.727 |
| doxylamine | tiapride | 0.748 | 0.741 |
| amikacin | moroxydine | 0.748 | 0.854 |
| doxorubicin | vidarabine | 0.748 | 0.684 |
| remoxipride | tobramycin | 0.748 | 0.763 |
| bromperidol | meticrane | 0.748 | 0.770 |
| adipiodone | rilmenidine | 0.748 | 0.839 |
| (-)-catechin | meticrane | 0.748 | 0.717 |
| astemizole | clemastine | 0.748 | 0.744 |
| cyproheptadine | prenylamine | 0.748 | 0.683 |
| desoxycortone | promazine | 0.748 | 0.764 |
| etamsylate | omeprazole | 0.748 | 0.719 |
| pentetrazol | talampicillin | 0.748 | 0.819 |
| sulfinpyrazone | terbutaline | 0.748 | 0.717 |
| dapsone | homatropine | 0.748 | 0.663 |
| apomorphine | gliclazide | 0.748 | 0.786 |

| | | | |
|---|---|---|---|
| nitrofurantoin | oxprenolol | 0.748 | 0.706 |
| doxazosin | protriptyline | 0.748 | 0.793 |
| griseofulvin | rimexolone | 0.748 | 0.724 |
| phenacetin | tolazamide | 0.748 | 0.715 |
| latamoxef | meptazinol | 0.748 | 0.799 |
| diazoxide | naltrexone | 0.748 | 0.745 |
| chloramphenicol | trichlormethiazide | 0.748 | 0.691 |
| benfluorex | monobenzone | 0.748 | 0.760 |
| fluocinonide | monobenzone | 0.748 | 0.812 |
| fenoprofen | meclozine | 0.748 | 0.755 |
| clonidine | liothyronine | 0.748 | 0.705 |
| naloxone | pyrazinamide | 0.748 | 0.769 |
| mometasone | raloxifene | 0.748 | 0.757 |
| propafenone | repaglinide | 0.748 | 0.741 |
| methylprednisolone | oxybutynin | 0.748 | 0.746 |
| ipratropium_bromide | liothyronine | 0.748 | 0.721 |
| methylergometrine | monobenzone | 0.748 | 0.719 |
| etomidate | ipratropium_bromide | 0.748 | 0.663 |
| desipramine | suloctidil | 0.749 | 0.739 |
| loperamide | pimethixene | 0.749 | 0.781 |
| phenoxybenzamine | verteporfin | 0.749 | 0.819 |
| cetirizine | meclofenoxate | 0.749 | 0.707 |
| glibenclamide | mepyramine | 0.749 | 0.786 |
| liothyronine | trichlormethiazide | 0.749 | 0.725 |
| estriol | procaine | 0.749 | 0.715 |
| pentetrazol | triflusal | 0.749 | 0.735 |
| prilocaine | tropicamide | 0.749 | 0.706 |
| lomustine | metergoline | 0.749 | 0.705 |
| etacrynic_acid | metergoline | 0.749 | 0.708 |
| flufenamic_acid | oxprenolol | 0.749 | 0.658 |
| chlortetracycline | promethazine | 0.749 | 0.746 |
| carbachol | daunorubicin | 0.749 | 0.841 |
| roxithromycin | triflusal | 0.749 | 0.857 |
| etamsylate | trioxysalen | 0.749 | 0.690 |
| bisacodyl | verteporfin | 0.749 | 0.784 |
| colchicine | phenoxybenzamine | 0.749 | 0.763 |
| ciclosporin | niclosamide | 0.749 | 0.882 |
| fluphenazine | metixene | 0.749 | 0.734 |
| dacarbazine | phenoxybenzamine | 0.749 | 0.790 |
| betaxolol | omeprazole | 0.749 | 0.681 |
| galantamine | tobramycin | 0.749 | 0.788 |
| gefitinib | ketanserin | 0.749 | 0.697 |
| meclozine | tyloxapol | 0.749 | 0.855 |
| cefalotin | milrinone | 0.749 | 0.750 |
| phenoxybenzamine | pimozide | 0.749 | 0.755 |
| pargyline | pentetrazol | 0.749 | 0.740 |
| flupentixol | syrosingopine | 0.749 | 0.763 |
| fluoxetine | metergoline | 0.749 | 0.721 |
| oxyphenbutazone | phenoxybenzamine | 0.749 | 0.737 |
| scopolamine | verteporfin | 0.749 | 0.820 |

| | | | |
|---|---|---|---|
| aminohippuric_acid | piribedil | 0.749 | 0.687 |
| dihydroergotamine | trifluoperazine | 0.749 | 0.736 |
| betahistine | trioxysalen | 0.749 | 0.680 |
| deptropine | propantheline_bromic | 0.749 | 0.687 |
| oxetacaine | rifabutin | 0.749 | 0.779 |
| metyrapone | terguride | 0.749 | 0.703 |
| benzethonium_chloride | cefmetazole | 0.749 | 0.778 |
| meticrane | pentoxyverine | 0.749 | 0.737 |
| sulfamethoxypyridazine | trioxysalen | 0.749 | 0.732 |
| cyproheptadine | mefloquine | 0.749 | 0.747 |
| meticrane | sulfafurazole | 0.749 | 0.694 |
| chlorcyclizine | maprotiline | 0.749 | 0.668 |
| meptazinol | metacycline | 0.749 | 0.750 |
| propylthiouracil | tyloxapol | 0.749 | 0.897 |
| glibenclamide | triflusal | 0.749 | 0.785 |
| ethisterone | vincamine | 0.749 | 0.753 |
| betaxolol | methapyrilene | 0.749 | 0.730 |
| mepacrine | vorinostat | 0.749 | 0.742 |
| estriol | roxithromycin | 0.749 | 0.846 |
| doxorubicin | sulfametoxydiazine | 0.749 | 0.728 |
| acepromazine | diltiazem | 0.749 | 0.721 |
| halcinonide | nitrofurantoin | 0.749 | 0.752 |
| sulfamethoxazole | trimethoprim | 0.749 | 0.690 |
| dacarbazine | tropicamide | 0.749 | 0.750 |
| chlorzoxazone | estriol | 0.749 | 0.723 |
| niclosamide | promazine | 0.749 | 0.702 |
| clotrimazole | nifuroxazide | 0.749 | 0.821 |
| levonorgestrel | tropicamide | 0.749 | 0.726 |
| alfaxalone | piribedil | 0.749 | 0.727 |
| nilutamide | pentoxyverine | 0.749 | 0.754 |
| phenazone | tiapride | 0.749 | 0.723 |
| bisacodyl | promazine | 0.749 | 0.704 |
| minoxidil | sulfaguanidine | 0.749 | 0.651 |
| phenazopyridine | syrosingopine | 0.749 | 0.816 |
| cinchocaine | naloxone | 0.749 | 0.746 |
| liothyronine | meticrane | 0.749 | 0.739 |
| latamoxef | monobenzone | 0.749 | 0.809 |
| demecolcine | ketanserin | 0.749 | 0.726 |
| meticrane | molindone | 0.749 | 0.685 |
| norethisterone | propafenone | 0.749 | 0.767 |
| letrozole | tocainide | 0.749 | 0.714 |
| sulfamethoxypyridazine | triflusal | 0.749 | 0.701 |
| pergolide | podophyllotoxin | 0.749 | 0.751 |
| cefalotin | talampicillin | 0.749 | 0.678 |
| cyclizine | thiocolchicoside | 0.749 | 0.789 |
| prenylamine | propafenone | 0.749 | 0.703 |
| demecolcine | dipivefrine | 0.750 | 0.729 |
| cyproheptadine | pyrvinium | 0.750 | 0.786 |
| liothyronine | metyrapone | 0.750 | 0.742 |
| hydralazine | tobramycin | 0.750 | 0.831 |

| | | | |
|---|---|---|---|
| levamisole | medrysone | 0.750 | 0.761 |
| pargyline | xylometazoline | 0.750 | 0.653 |
| paroxetine | quinethazone | 0.750 | 0.716 |
| etofenamate | monobenzone | 0.750 | 0.730 |
| doxazosin | ticlopidine | 0.750 | 0.770 |
| imipramine | mometasone | 0.750 | 0.781 |
| ipratropium_bromide | trazodone | 0.750 | 0.703 |
| benzethonium_chloride | protriptyline | 0.750 | 0.721 |
| chlorprothixene | emetine | 0.750 | 0.760 |
| cisapride | nitrofural | 0.750 | 0.774 |
| disulfiram | rifabutin | 0.750 | 0.811 |
| calcium_pantothenate | deptropine | 0.750 | 0.825 |
| astemizole | rifabutin | 0.750 | 0.804 |
| dacarbazine | trazodone | 0.750 | 0.764 |
| chlorzoxazone | latamoxef | 0.750 | 0.826 |
| chlorzoxazone | repaglinide | 0.750 | 0.800 |
| mefloquine | phenazopyridine | 0.750 | 0.727 |
| bisacodyl | dacarbazine | 0.750 | 0.784 |
| colchicine | practolol | 0.750 | 0.741 |
| ciclosporin | saquinavir | 0.750 | 0.789 |
| cetirizine | dipyridamole | 0.750 | 0.780 |
| glipizide | mitoxantrone | 0.750 | 0.734 |
| carbachol | progesterone | 0.750 | 0.759 |
| fluphenazine | suloctidil | 0.750 | 0.749 |
| azacitidine | benfluorex | 0.750 | 0.750 |
| alprostadil | etilefrine | 0.750 | 0.776 |
| cetirizine | clobetasol | 0.750 | 0.726 |
| cefalotin | chlorzoxazone | 0.750 | 0.797 |
| maprotiline | prenylamine | 0.750 | 0.670 |
| rolitetracycline | tobramycin | 0.750 | 0.747 |
| demecolcine | terfenadine | 0.750 | 0.764 |
| ethosuximide | ipratropium_bromide | 0.750 | 0.776 |
| phenazopyridine | suloctidil | 0.750 | 0.739 |
| repaglinide | zomepirac | 0.750 | 0.718 |
| mometasone | oxetacaine | 0.750 | 0.726 |
| oxybuprocaine | rimexolone | 0.750 | 0.738 |
| dipyridamole | midecamycin | 0.750 | 0.794 |
| sulconazole | zomepirac | 0.750 | 0.696 |
| metamizole_sodium | natamycin | 0.750 | 0.790 |
| sulconazole | ticlopidine | 0.750 | 0.699 |
| deptropine | practolol | 0.750 | 0.773 |
| fenoprofen | gliclazide | 0.750 | 0.715 |
| doxazosin | phenoxybenzamine | 0.750 | 0.744 |
| repaglinide | vidarabine | 0.750 | 0.768 |
| trioxysalen | vidarabine | 0.750 | 0.694 |
| meclozine | menadione | 0.750 | 0.812 |
| methylergometrine | nortriptyline | 0.750 | 0.699 |
| calcium_pantothenate | meptazinol | 0.750 | 0.834 |
| liothyronine | meptazinol | 0.750 | 0.756 |
| menadione | propafenone | 0.750 | 0.717 |

| | | | |
|---|---|---|---|
| simvastatin | sulconazole | 0.750 | 0.742 |
| amitriptyline | prochlorperazine | 0.750 | 0.672 |
| pimozide | promethazine | 0.750 | 0.786 |
| doxorubicin | tobramycin | 0.750 | 0.715 |
| tolnaftate | zalcitabine | 0.750 | 0.733 |
| loperamide | propafenone | 0.750 | 0.711 |
| phenoxybenzamine | protriptyline | 0.750 | 0.702 |
| disulfiram | rescinnamine | 0.750 | 0.784 |
| doxorubicin | tropicamide | 0.750 | 0.756 |
| lanatoside_C | primaquine | 0.750 | 0.874 |
| latamoxef | meticrane | 0.750 | 0.786 |
| daunorubicin | dipyridamole | 0.751 | 0.715 |
| clomipramine | propylthiouracil | 0.751 | 0.761 |
| betaxolol | repaglinide | 0.751 | 0.766 |
| chlorzoxazone | rofecoxib | 0.751 | 0.755 |
| levamisole | propylthiouracil | 0.751 | 0.673 |
| phensuximide | rimexolone | 0.751 | 0.785 |
| acetylsalicylic_acid | talampicillin | 0.751 | 0.760 |
| cinchocaine | trioxysalen | 0.751 | 0.747 |
| trifluoperazine | trihexyphenidyl | 0.751 | 0.728 |
| albendazole | benzydamine | 0.751 | 0.674 |
| mitoxantrone | scopolamine | 0.751 | 0.784 |
| ethambutol | nafcillin | 0.751 | 0.732 |
| daunorubicin | glibenclamide | 0.751 | 0.716 |
| prenylamine | zuclopenthixol | 0.751 | 0.737 |
| digoxin | disulfiram | 0.751 | 0.836 |
| rolitetracycline | triflusal | 0.751 | 0.771 |
| bepridil | nortriptyline | 0.751 | 0.689 |
| flufenamic_acid | propafenone | 0.751 | 0.682 |
| bromperidol | omeprazole | 0.751 | 0.692 |
| cefsulodin | reserpine | 0.751 | 0.728 |
| metyrapone | piperidolate | 0.751 | 0.668 |
| bisacodyl | colecalciferol | 0.751 | 0.753 |
| glipizide | oleandomycin | 0.751 | 0.803 |
| azacitidine | meticrane | 0.751 | 0.701 |
| pargyline | tiratricol | 0.751 | 0.761 |
| betahistine | flufenamic_acid | 0.751 | 0.662 |
| doxazosin | dydrogesterone | 0.751 | 0.750 |
| benfluorex | promethazine | 0.751 | 0.745 |
| cetirizine | oxybuprocaine | 0.751 | 0.760 |
| bretylium_tosilate | diloxanide | 0.751 | 0.765 |
| methylergometrine | pyrvinium | 0.751 | 0.760 |
| chlorcyclizine | mefloquine | 0.751 | 0.749 |
| cetirizine | levonorgestrel | 0.751 | 0.773 |
| ifosfamide | lidoflazine | 0.751 | 0.794 |
| metamizole_sodium | sulfaphenazole | 0.751 | 0.674 |
| metamizole_sodium | sulfamethoxypyridazir | 0.751 | 0.688 |
| roxithromycin | tobramycin | 0.751 | 0.784 |
| chlorprothixene | felodipine | 0.751 | 0.712 |
| bromopride | doxorubicin | 0.751 | 0.698 |

| | | | |
|---|---|---|---|
| pyrazinamide | roxithromycin | 0.751 | 0.906 |
| dacarbazine | roxithromycin | 0.751 | 0.873 |
| clomipramine | felodipine | 0.751 | 0.742 |
| latamoxef | oxybuprocaine | 0.751 | 0.761 |
| azacitidine | meclozine | 0.751 | 0.806 |
| piromidic_acid | tyloxapol | 0.751 | 0.862 |
| procyclidine | theobromine | 0.751 | 0.786 |
| flufenamic_acid | pivampicillin | 0.751 | 0.723 |
| chlorprothixene | proscillaridin | 0.751 | 0.827 |
| etamsylate | etomidate | 0.751 | 0.713 |
| etacrynic_acid | protriptyline | 0.751 | 0.722 |
| cefalotin | piperidolate | 0.751 | 0.727 |
| estriol | zomepirac | 0.751 | 0.663 |
| niclosamide | pizotifen | 0.751 | 0.724 |
| diltiazem | naloxone | 0.751 | 0.725 |
| doxorubicin | sulfamethoxazole | 0.751 | 0.757 |
| cetirizine | liothyronine | 0.751 | 0.693 |
| bisacodyl | progesterone | 0.751 | 0.720 |
| clomipramine | dacarbazine | 0.751 | 0.762 |
| ketanserin | promazine | 0.751 | 0.734 |
| pentamidine | sulconazole | 0.751 | 0.766 |
| alprenolol | chlortalidone | 0.751 | 0.690 |
| amoxicillin | rimexolone | 0.751 | 0.721 |
| amiodarone | medrysone | 0.751 | 0.746 |
| meropenem | talampicillin | 0.751 | 0.713 |
| mepacrine | mepyramine | 0.751 | 0.729 |
| flufenamic_acid | hydrocortisone | 0.751 | 0.731 |
| hyoscyamine | liothyronine | 0.751 | 0.750 |
| ketoprofen | lomefloxacin | 0.751 | 0.670 |
| mefloquine | thioridazine | 0.751 | 0.729 |
| isoetarine | methylergometrine | 0.751 | 0.736 |
| hydrocortisone | profenamine | 0.751 | 0.790 |
| doxorubicin | meclofenoxate | 0.751 | 0.715 |
| etidronic_acid | meticrane | 0.751 | 0.735 |
| oxamic_acid | thioridazine | 0.751 | 0.900 |
| mifepristone | pimozide | 0.751 | 0.765 |
| cortisone | trazodone | 0.751 | 0.755 |
| niclosamide | tamoxifen | 0.751 | 0.747 |
| aminohippuric_acid | repaglinide | 0.751 | 0.768 |
| gefitinib | vinpocetine | 0.751 | 0.762 |
| benzylpenicillin | doxorubicin | 0.751 | 0.709 |
| etodolac | xylometazoline | 0.751 | 0.688 |
| biotin | phenylpropanolamine | 0.751 | 0.736 |
| miconazole | syrosingopine | 0.751 | 0.809 |
| cyproheptadine | gefitinib | 0.751 | 0.760 |
| deptropine | promazine | 0.751 | 0.680 |
| fludroxycortide | tiapride | 0.751 | 0.750 |
| danazol | loperamide | 0.751 | 0.761 |
| econazole | perphenazine | 0.751 | 0.727 |
| cortisone | sulconazole | 0.752 | 0.761 |

| | | | |
|---|---|---|---|
| flucytosine | tyloxapol | 0.752 | 0.920 |
| benperidol | trichlormethiazide | 0.752 | 0.757 |
| cyproterone | tyloxapol | 0.752 | 0.856 |
| cetirizine | phenoxybenzamine | 0.752 | 0.752 |
| doxazosin | sulfafurazole | 0.752 | 0.750 |
| bisacodyl | metixene | 0.752 | 0.699 |
| methotrexate | tioguanine | 0.752 | 0.718 |
| dobutamine | mebendazole | 0.752 | 0.714 |
| betazole | imipramine | 0.752 | 0.800 |
| promazine | sulfametoxydiazine | 0.752 | 0.725 |
| acetylsalicylic_acid | piromidic_acid | 0.752 | 0.704 |
| beclometasone | letrozole | 0.752 | 0.767 |
| hydroquinine | lanatoside_C | 0.752 | 0.864 |
| scopolamine | triflusal | 0.752 | 0.738 |
| astemizole | haloperidol | 0.752 | 0.705 |
| bromperidol | etomidate | 0.752 | 0.734 |
| benfluorex | emetine | 0.752 | 0.729 |
| doxylamine | trifluoperazine | 0.752 | 0.710 |
| bromperidol | trioxysalen | 0.752 | 0.770 |
| cefsulodin | theobromine | 0.752 | 0.808 |
| daunorubicin | latamoxef | 0.752 | 0.714 |
| clonidine | dacarbazine | 0.752 | 0.667 |
| fluoxetine | loperamide | 0.752 | 0.718 |
| meticrane | piromidic_acid | 0.752 | 0.688 |
| betahistine | triflusal | 0.752 | 0.729 |
| lymecycline | risperidone | 0.752 | 0.769 |
| sulfamethoxypyridazine | verteporfin | 0.752 | 0.814 |
| methylergometrine | orciprenaline | 0.752 | 0.732 |
| ifenprodil | roxithromycin | 0.752 | 0.818 |
| clindamycin | diazoxide | 0.752 | 0.773 |
| mesoridazine | rimexolone | 0.752 | 0.749 |
| benzethonium_chloride | chlorcyclizine | 0.752 | 0.739 |
| etoposide | vorinostat | 0.752 | 0.762 |
| buflomedil | roxithromycin | 0.752 | 0.829 |
| betaxolol | zidovudine | 0.752 | 0.729 |
| mebendazole | protriptyline | 0.752 | 0.732 |
| liothyronine | tropicamide | 0.752 | 0.735 |
| chloramphenicol | molindone | 0.752 | 0.712 |
| estriol | metacycline | 0.752 | 0.700 |
| gliclazide | levamisole | 0.752 | 0.744 |
| terguride | triflusal | 0.752 | 0.775 |
| nicotinic_acid | profenamine | 0.752 | 0.745 |
| bromocriptine | clomipramine | 0.752 | 0.781 |
| desoxycortone | phenoxybenzamine | 0.752 | 0.760 |
| tropicamide | tyloxapol | 0.752 | 0.866 |
| carmustine | phenoxybenzamine | 0.752 | 0.740 |
| deferoxamine | medrysone | 0.752 | 0.832 |
| liothyronine | verteporfin | 0.752 | 0.775 |
| bromopride | etamsylate | 0.752 | 0.707 |
| doxazosin | milrinone | 0.752 | 0.747 |

| | | | |
|---|---|---|---|
| podophyllotoxin | prochlorperazine | 0.752 | 0.739 |
| estriol | meropenem | 0.752 | 0.708 |
| meglumine | naftidrofuryl | 0.752 | 0.773 |
| betahistine | etofenamate | 0.752 | 0.722 |
| acetylsalicylic_acid | benzylpenicillin | 0.752 | 0.742 |
| dextromethorphan | rimexolone | 0.752 | 0.777 |
| procaine | repaglinide | 0.752 | 0.749 |
| dacarbazine | zomepirac | 0.752 | 0.713 |
| bepridil | cinchocaine | 0.752 | 0.745 |
| norethisterone | tropicamide | 0.752 | 0.725 |
| moxisylyte | sulfadiazine | 0.752 | 0.742 |
| imipenem | propafenone | 0.752 | 0.738 |
| ifenprodil | latamoxef | 0.752 | 0.737 |
| tolfenamic_acid | trimetazidine | 0.752 | 0.680 |
| benzylpenicillin | meropenem | 0.752 | 0.684 |
| maprotiline | profenamine | 0.752 | 0.682 |
| idoxuridine | rifabutin | 0.752 | 0.808 |
| piperacillin | spiramycin | 0.752 | 0.771 |
| fluspirilene | trimipramine | 0.752 | 0.762 |
| repaglinide | urapidil | 0.752 | 0.726 |
| digoxin | mepacrine | 0.752 | 0.843 |
| levonorgestrel | sulfamerazine | 0.752 | 0.741 |
| nialamide | sulconazole | 0.752 | 0.763 |
| amoxapine | fluphenazine | 0.752 | 0.722 |
| piracetam | thiocolchicoside | 0.752 | 0.828 |
| trimetazidine | tyloxapol | 0.752 | 0.871 |
| levomepromazine | lovastatin | 0.752 | 0.766 |
| ethotoin | pentoxyverine | 0.752 | 0.710 |
| bepridil | methylergometrine | 0.752 | 0.729 |
| talampicillin | tyloxapol | 0.752 | 0.822 |
| diltiazem | zimeldine | 0.752 | 0.715 |
| aminohippuric_acid | desoxycortone | 0.752 | 0.752 |
| promethazine | tropicamide | 0.752 | 0.715 |
| oxolinic_acid | pentetrazol | 0.752 | 0.662 |
| piribedil | propafenone | 0.753 | 0.704 |
| chlorcyclizine | haloperidol | 0.753 | 0.756 |
| fluphenazine | sirolimus | 0.753 | 0.817 |
| fenoprofen | pipemidic_acid | 0.753 | 0.694 |
| dacarbazine | naloxone | 0.753 | 0.726 |
| hydrocortisone | phenylpropanolamine | 0.753 | 0.773 |
| amiodarone | cefalotin | 0.753 | 0.749 |
| flufenamic_acid | propylthiouracil | 0.753 | 0.665 |
| metamizole_sodium | rimexolone | 0.753 | 0.702 |
| chlorzoxazone | molindone | 0.753 | 0.710 |
| fluvoxamine | niclosamide | 0.753 | 0.658 |
| propafenone | propylthiouracil | 0.753 | 0.758 |
| piribedil | xylometazoline | 0.753 | 0.732 |
| dienestrol | phenoxybenzamine | 0.753 | 0.764 |
| mesoridazine | tyloxapol | 0.753 | 0.844 |
| fluspirilene | mometasone | 0.753 | 0.690 |

| | | | |
|---|---|---|---|
| betahistine | tyloxapol | 0.753 | 0.909 |
| fenoprofen | sulfaphenazole | 0.753 | 0.671 |
| cetirizine | sulfafurazole | 0.753 | 0.751 |
| apomorphine | felodipine | 0.753 | 0.736 |
| ivermectin | rescinnamine | 0.753 | 0.802 |
| metyrapone | rimexolone | 0.753 | 0.692 |
| profenamine | terfenadine | 0.753 | 0.783 |
| procaine | scopolamine | 0.753 | 0.680 |
| chlorambucil | quinethazone | 0.753 | 0.718 |
| ipratropium_bromide | trimetazidine | 0.753 | 0.716 |
| desoxycortone | triflusal | 0.753 | 0.794 |
| irinotecan | sulconazole | 0.753 | 0.800 |
| dacarbazine | sulfametoxydiazine | 0.753 | 0.699 |
| alimemazine | astemizole | 0.753 | 0.745 |
| oxytetracycline | vinpocetine | 0.753 | 0.716 |
| cefazolin | isocarboxazid | 0.753 | 0.727 |
| hexetidine | proscillaridin | 0.753 | 0.780 |
| latamoxef | naloxone | 0.753 | 0.775 |
| cinchocaine | sulfafurazole | 0.753 | 0.730 |
| amoxicillin | doxorubicin | 0.753 | 0.739 |
| chlortetracycline | ketanserin | 0.753 | 0.729 |
| metergoline | profenamine | 0.753 | 0.745 |
| procainamide | triflusal | 0.753 | 0.731 |
| oxybuprocaine | piperidolate | 0.753 | 0.698 |
| hydrocortisone | imipenem | 0.753 | 0.698 |
| bendroflumethiazide | promazine | 0.753 | 0.754 |
| etamsylate | progesterone | 0.753 | 0.801 |
| rimexolone | tolfenamic_acid | 0.753 | 0.738 |
| fenspiride | pargyline | 0.753 | 0.688 |
| chlorcyclizine | miconazole | 0.753 | 0.679 |
| trimetazidine | trioxysalen | 0.753 | 0.682 |
| perphenazine | rifabutin | 0.753 | 0.780 |
| diltiazem | practolol | 0.753 | 0.758 |
| dexibuprofen | promethazine | 0.753 | 0.731 |
| etofenamate | promazine | 0.753 | 0.713 |
| chloroquine | mesoridazine | 0.753 | 0.688 |
| dacarbazine | meclofenoxate | 0.753 | 0.715 |
| haloperidol | maprotiline | 0.753 | 0.753 |
| medrysone | oxprenolol | 0.753 | 0.729 |
| benzydamine | cinnarizine | 0.753 | 0.712 |
| gliclazide | promethazine | 0.753 | 0.769 |
| clomipramine | tolfenamic_acid | 0.753 | 0.700 |
| carbimazole | dinoprost | 0.753 | 0.811 |
| deferoxamine | sulconazole | 0.753 | 0.846 |
| apomorphine | danazol | 0.753 | 0.723 |
| ifenprodil | liothyronine | 0.753 | 0.718 |
| sulfametoxydiazine | verteporfin | 0.753 | 0.820 |
| metacycline | sulfametoxydiazine | 0.753 | 0.703 |
| cyclobenzaprine | hexetidine | 0.753 | 0.728 |
| ipratropium_bromide | levamisole | 0.753 | 0.722 |

| | | | |
|---|---|---|---|
| galantamine | molindone | 0.753 | 0.724 |
| etamivan | mesoridazine | 0.753 | 0.731 |
| cyproheptadine | zuclopenthixol | 0.753 | 0.664 |
| sulfanilamide | zomepirac | 0.753 | 0.683 |
| loperamide | prochlorperazine | 0.753 | 0.690 |
| rifabutin | thioproperazine | 0.753 | 0.774 |
| vidarabine | zomepirac | 0.753 | 0.708 |
| capsaicin | monobenzone | 0.753 | 0.701 |
| propafenone | talampicillin | 0.753 | 0.736 |
| etamsylate | metacycline | 0.753 | 0.751 |
| dihydroergocristine | terfenadine | 0.753 | 0.759 |
| liothyronine | rimexolone | 0.753 | 0.724 |
| natamycin | rimexolone | 0.753 | 0.801 |
| fenoprofen | repaglinide | 0.753 | 0.740 |
| mefloquine | trimipramine | 0.753 | 0.738 |
| cycloserine | piperidolate | 0.753 | 0.908 |
| dextromethorphan | milrinone | 0.754 | 0.747 |
| cycloserine | verteporfin | 0.754 | 0.912 |
| clemastine | perphenazine | 0.754 | 0.731 |
| deptropine | trifluoperazine | 0.754 | 0.692 |
| chloramphenicol | trioxysalen | 0.754 | 0.719 |
| idoxuridine | pyrimethamine | 0.754 | 0.664 |
| beclometasone | doxazosin | 0.754 | 0.753 |
| gefitinib | nimesulide | 0.754 | 0.727 |
| deptropine | phenoxybenzamine | 0.754 | 0.718 |
| fendiline | trimipramine | 0.754 | 0.669 |
| lomustine | rescinnamine | 0.754 | 0.805 |
| metixene | terconazole | 0.754 | 0.766 |
| gefitinib | mebendazole | 0.754 | 0.680 |
| procaine | tyloxapol | 0.754 | 0.881 |
| nortriptyline | rescinnamine | 0.754 | 0.799 |
| ipratropium_bromide | sulfafurazole | 0.754 | 0.681 |
| loperamide | propofol | 0.754 | 0.816 |
| cefalotin | natamycin | 0.754 | 0.787 |
| dextromethorphan | triflusal | 0.754 | 0.759 |
| doxazosin | hyoscyamine | 0.754 | 0.744 |
| levamisole | tiratricol | 0.754 | 0.752 |
| omeprazole | ronidazole | 0.754 | 0.703 |
| clotrimazole | menadione | 0.754 | 0.770 |
| progesterone | sulconazole | 0.754 | 0.705 |
| nitrofurantoin | tiapride | 0.754 | 0.701 |
| mometasone | trimipramine | 0.754 | 0.781 |
| butoconazole | phenoxybenzamine | 0.754 | 0.711 |
| nabumetone | protriptyline | 0.754 | 0.711 |
| capsaicin | ioversol | 0.754 | 0.810 |
| ciclopirox | perphenazine | 0.754 | 0.763 |
| terfenadine | ticlopidine | 0.754 | 0.776 |
| clomipramine | gliclazide | 0.754 | 0.776 |
| diphenhydramine | heptaminol | 0.754 | 0.747 |
| acetylsalicylic_acid | sulfametoxydiazine | 0.754 | 0.673 |

| | | | |
|---|---|---|---|
| lymecycline | talampicillin | 0.754 | 0.714 |
| ornidazole | pivampicillin | 0.754 | 0.780 |
| doxazosin | thiocolchicoside | 0.754 | 0.711 |
| etamsylate | piromidic_acid | 0.754 | 0.668 |
| betamethasone | carbachol | 0.754 | 0.801 |
| benzylpenicillin | meticrane | 0.754 | 0.715 |
| chloramphenicol | clindamycin | 0.754 | 0.725 |
| cinchocaine | sulfamethoxypyridazir | 0.754 | 0.697 |
| medrysone | propafenone | 0.754 | 0.738 |
| etacrynic_acid | prenylamine | 0.754 | 0.774 |
| cetirizine | xylometazoline | 0.754 | 0.760 |
| famotidine | oxamic_acid | 0.754 | 0.823 |
| propylthiouracil | zimeldine | 0.754 | 0.691 |
| aminohippuric_acid | roxithromycin | 0.754 | 0.872 |
| phenoxybenzamine | promazine | 0.754 | 0.700 |
| dihydroergocristine | metixene | 0.754 | 0.783 |
| hexetidine | pyrvinium | 0.754 | 0.782 |
| cycloserine | roxithromycin | 0.754 | 0.912 |
| chlorzoxazone | clomipramine | 0.754 | 0.761 |
| digoxin | loperamide | 0.754 | 0.819 |
| clotrimazole | exemestane | 0.754 | 0.736 |
| meclofenoxate | metamizole_sodium | 0.754 | 0.671 |
| colchicine | gefitinib | 0.754 | 0.711 |
| irinotecan | pyrvinium | 0.754 | 0.778 |
| fipexide | tolfenamic_acid | 0.754 | 0.703 |
| mepyramine | oxybutynin | 0.754 | 0.710 |
| clioquinol | tyloxapol | 0.754 | 0.907 |
| propafenone | tyloxapol | 0.754 | 0.843 |
| liothyronine | medrysone | 0.754 | 0.716 |
| tiaprofenic_acid | trimethadione | 0.754 | 0.743 |
| carisoprodol | moxisylyte | 0.754 | 0.714 |
| cinchocaine | oxetacaine | 0.754 | 0.734 |
| phenacetin | tobramycin | 0.754 | 0.826 |
| oxyphenbutazone | perphenazine | 0.754 | 0.714 |
| isradipine | protriptyline | 0.754 | 0.711 |
| procaine | rolitetracycline | 0.754 | 0.760 |
| flumetasone | hydralazine | 0.754 | 0.799 |
| hydroquinine | proscillaridin | 0.754 | 0.783 |
| carbachol | phenoxybenzamine | 0.754 | 0.790 |
| bezafibrate | clomifene | 0.754 | 0.738 |
| digoxin | irinotecan | 0.754 | 0.748 |
| bromocriptine | oxetacaine | 0.754 | 0.717 |
| dacarbazine | ketanserin | 0.754 | 0.735 |
| ioversol | rimexolone | 0.754 | 0.764 |
| dextromethorphan | doxorubicin | 0.754 | 0.776 |
| bisacodyl | oxprenolol | 0.754 | 0.730 |
| deptropine | ipratropium_bromide | 0.754 | 0.724 |
| acetylsalicylic_acid | meclozine | 0.754 | 0.801 |
| bufexamac | liothyronine | 0.754 | 0.674 |
| amoxapine | chlorprothixene | 0.754 | 0.678 |

| | | | |
|---|---|---|---|
| etofenamate | liothyronine | 0.755 | 0.713 |
| cinchocaine | trazodone | 0.755 | 0.706 |
| pimozide | prenylamine | 0.755 | 0.716 |
| monobenzone | xylometazoline | 0.755 | 0.714 |
| ethambutol | rifampicin | 0.755 | 0.857 |
| amiodarone | roxithromycin | 0.755 | 0.808 |
| ivermectin | syrosingopine | 0.755 | 0.785 |
| carisoprodol | liothyronine | 0.755 | 0.745 |
| flupentixol | protriptyline | 0.755 | 0.722 |
| azacitidine | metamizole_sodium | 0.755 | 0.703 |
| latamoxef | scopolamine | 0.755 | 0.735 |
| betahistine | pivampicillin | 0.755 | 0.773 |
| dacarbazine | pentoxyverine | 0.755 | 0.765 |
| acepromazine | gliclazide | 0.755 | 0.748 |
| ciclopirox | prenylamine | 0.755 | 0.741 |
| bromopride | roxithromycin | 0.755 | 0.845 |
| cefalotin | zomepirac | 0.755 | 0.705 |
| cefalotin | gliclazide | 0.755 | 0.680 |
| iopanoic_acid | lymecycline | 0.755 | 0.797 |
| imipramine | metrifonate | 0.755 | 0.765 |
| fluticasone | zuclopenthixol | 0.755 | 0.759 |
| estriol | vidarabine | 0.755 | 0.675 |
| pyrvinium | rimexolone | 0.755 | 0.785 |
| cyproterone | natamycin | 0.755 | 0.791 |
| menadione | perphenazine | 0.755 | 0.762 |
| bromopride | gliclazide | 0.755 | 0.655 |
| benzethonium_chloride | phenoxybenzamine | 0.755 | 0.713 |
| acepromazine | etamsylate | 0.755 | 0.755 |
| methoxsalen | progesterone | 0.755 | 0.732 |
| clomifene | mefloquine | 0.755 | 0.788 |
| fendiline | quinisocaine | 0.755 | 0.697 |
| gefitinib | midecamycin | 0.755 | 0.808 |
| dacarbazine | ifenprodil | 0.755 | 0.760 |
| acepromazine | tyloxapol | 0.755 | 0.858 |
| amiodarone | ronidazole | 0.755 | 0.762 |
| milrinone | tyloxapol | 0.755 | 0.888 |
| citiolone | metamizole_sodium | 0.755 | 0.755 |
| cefalotin | fluspirilene | 0.755 | 0.707 |
| fenoprofen | scopolamine | 0.755 | 0.694 |
| acetylsalicylic_acid | medrysone | 0.755 | 0.742 |
| clenbuterol | testosterone | 0.755 | 0.752 |
| butoconazole | fluspirilene | 0.755 | 0.743 |
| mephenesin | trimethoprim | 0.755 | 0.673 |
| doxazosin | oxprenolol | 0.755 | 0.720 |
| meticrane | sulfamethoxypyridazir | 0.755 | 0.686 |
| daunorubicin | repaglinide | 0.755 | 0.742 |
| simvastatin | tobramycin | 0.755 | 0.748 |
| clioquinol | sulconazole | 0.755 | 0.730 |
| prochlorperazine | topiramate | 0.755 | 0.741 |
| bromocriptine | orciprenaline | 0.755 | 0.795 |

| | | | |
|---|---|---|---|
| clindamycin | estriol | 0.755 | 0.759 |
| doxazosin | iopanoic_acid | 0.755 | 0.742 |
| emetine | etoposide | 0.755 | 0.742 |
| colchicine | podophyllotoxin | 0.755 | 0.686 |
| dipyridamole | monobenzone | 0.755 | 0.798 |
| antazoline | oxybutynin | 0.755 | 0.717 |
| chlorcyclizine | ivermectin | 0.755 | 0.863 |
| fluphenazine | troglitazone | 0.755 | 0.704 |
| atovaquone | triflusal | 0.755 | 0.744 |
| clomipramine | propofol | 0.755 | 0.747 |
| bumetanide | chenodeoxycholic_aci | 0.755 | 0.767 |
| phenylpropanolamine | urapidil | 0.755 | 0.787 |
| hydralazine | meclofenoxate | 0.755 | 0.691 |
| meticrane | remoxipride | 0.755 | 0.699 |
| ivermectin | naltrexone | 0.755 | 0.851 |
| molindone | sulfametoxydiazine | 0.755 | 0.654 |
| amoxicillin | roxithromycin | 0.755 | 0.811 |
| doxazosin | meclofenoxate | 0.755 | 0.711 |
| ambroxol | ceforanide | 0.755 | 0.783 |
| benfluorex | phenoxybenzamine | 0.755 | 0.719 |
| bezafibrate | flufenamic_acid | 0.755 | 0.710 |
| deptropine | pipemidic_acid | 0.755 | 0.764 |
| bepridil | procainamide | 0.755 | 0.742 |
| demecolcine | proscillaridin | 0.755 | 0.800 |
| prenylamine | prochlorperazine | 0.755 | 0.712 |
| captopril | prednisone | 0.755 | 0.719 |
| maprotiline | phenazopyridine | 0.755 | 0.743 |
| niclosamide | semustine | 0.755 | 0.678 |
| benperidol | omeprazole | 0.755 | 0.695 |
| letrozole | trichlormethiazide | 0.755 | 0.740 |
| liothyronine | reserpine | 0.755 | 0.751 |
| articaine | ursodeoxycholic_acid | 0.755 | 0.772 |
| medrysone | phenoxybenzamine | 0.755 | 0.762 |
| proxyphylline | sulconazole | 0.755 | 0.770 |
| estrone | fluspirilene | 0.756 | 0.684 |
| promethazine | remoxipride | 0.756 | 0.712 |
| betaxolol | exemestane | 0.756 | 0.755 |
| diclofenac | estriol | 0.756 | 0.665 |
| colchicine | metergoline | 0.756 | 0.735 |
| altretamine | terguride | 0.756 | 0.770 |
| benfluorex | etofenamate | 0.756 | 0.654 |
| probucol | sulfafurazole | 0.756 | 0.781 |
| lomustine | mometasone | 0.756 | 0.736 |
| procyclidine | simvastatin | 0.756 | 0.727 |
| pyrazinamide | rimexolone | 0.756 | 0.824 |
| metixene | progesterone | 0.756 | 0.766 |
| pimethixene | trazodone | 0.756 | 0.761 |
| bisacodyl | latamoxef | 0.756 | 0.766 |
| hexetidine | primaquine | 0.756 | 0.753 |
| ondansetron | saquinavir | 0.756 | 0.759 |

| | | | |
|---|---|---|---|
| procyclidine | roxithromycin | 0.756 | 0.831 |
| oxprenolol | sulconazole | 0.756 | 0.743 |
| dipyridamole | meticrane | 0.756 | 0.757 |
| (-)-atenolol | clioquinol | 0.756 | 0.749 |
| dacarbazine | daunorubicin | 0.756 | 0.739 |
| liothyronine | naloxone | 0.756 | 0.748 |
| bromocriptine | cinchocaine | 0.756 | 0.749 |
| chloramphenicol | norethisterone | 0.756 | 0.731 |
| ipratropium_bromide | omeprazole | 0.756 | 0.655 |
| diflorasone | selegiline | 0.756 | 0.783 |
| carbachol | profenamine | 0.756 | 0.796 |
| cisapride | doxazosin | 0.756 | 0.684 |
| lanatoside_C | metergoline | 0.756 | 0.844 |
| domperidone | ritodrine | 0.756 | 0.697 |
| nicergoline | niclosamide | 0.756 | 0.682 |
| cycloserine | scopolamine | 0.756 | 0.813 |
| thioridazine | tioguanine | 0.756 | 0.757 |
| etoposide | fluspirilene | 0.756 | 0.734 |
| mepyramine | milrinone | 0.756 | 0.740 |
| econazole | loperamide | 0.756 | 0.732 |
| loperamide | raloxifene | 0.756 | 0.738 |
| desoxycortone | mitoxantrone | 0.756 | 0.748 |
| bisacodyl | roxithromycin | 0.756 | 0.805 |
| carbachol | reserpine | 0.756 | 0.835 |
| ipratropium_bromide | norethisterone | 0.756 | 0.710 |
| bromperidol | pyrvinium | 0.756 | 0.765 |
| medrysone | ronidazole | 0.756 | 0.769 |
| molindone | roxithromycin | 0.756 | 0.846 |
| clozapine | terfenadine | 0.756 | 0.783 |
| perhexiline | practolol | 0.756 | 0.777 |
| enalapril | triprolidine | 0.756 | 0.731 |
| guanethidine | vinpocetine | 0.756 | 0.745 |
| hydralazine | hydrocortisone | 0.756 | 0.798 |
| amiodarone | bromopride | 0.756 | 0.693 |
| acetazolamide | metrifonate | 0.756 | 0.717 |
| cefalotin | daunorubicin | 0.756 | 0.722 |
| sulconazole | ursodeoxycholic_acid | 0.756 | 0.758 |
| alfaxalone | sulconazole | 0.756 | 0.764 |
| astemizole | primaquine | 0.756 | 0.724 |
| doxazosin | norethisterone | 0.756 | 0.770 |
| bisacodyl | etamsylate | 0.756 | 0.786 |
| ketanserin | propantheline_bromic | 0.756 | 0.715 |
| cefazolin | mephenesin | 0.756 | 0.788 |
| sulfafurazole | testosterone | 0.756 | 0.762 |
| acetylsalicylic_acid | phenoxybenzamine | 0.756 | 0.784 |
| piperidolate | trimetazidine | 0.756 | 0.746 |
| latamoxef | trazodone | 0.756 | 0.716 |
| colecalciferol | tyloxapol | 0.756 | 0.837 |
| roxithromycin | trimetazidine | 0.756 | 0.848 |
| etoposide | gefitinib | 0.756 | 0.746 |

| | | | |
|---|---|---|---|
| theophylline | trichlormethiazide | 0.756 | 0.732 |
| dacarbazine | latamoxef | 0.756 | 0.772 |
| betamethasone | bisoprolol | 0.756 | 0.761 |
| latamoxef | rimexolone | 0.756 | 0.753 |
| amoxapine | zimeldine | 0.756 | 0.695 |
| latamoxef | methazolamide | 0.756 | 0.753 |
| liothyronine | piribedil | 0.756 | 0.668 |
| betaxolol | meticrane | 0.756 | 0.744 |
| oxaprozin | quinethazone | 0.756 | 0.717 |
| cyproterone | liothyronine | 0.756 | 0.692 |
| acetylsalicylic_acid | trioxysalen | 0.756 | 0.672 |
| phenoxybenzamine | ticlopidine | 0.756 | 0.654 |
| labetalol | mefloquine | 0.756 | 0.708 |
| menadione | talampicillin | 0.756 | 0.772 |
| piromidic_acid | sulfamethoxypyridazir | 0.756 | 0.678 |
| flufenamic_acid | pargyline | 0.756 | 0.694 |
| podophyllotoxin | topiramate | 0.756 | 0.739 |
| astemizole | promethazine | 0.756 | 0.717 |
| (-)-catechin | repaglinide | 0.756 | 0.758 |
| ifenprodil | perhexiline | 0.756 | 0.783 |
| halcinonide | sulfamerazine | 0.756 | 0.750 |
| doxazosin | pyrazinamide | 0.756 | 0.757 |
| bisacodyl | trioxysalen | 0.756 | 0.779 |
| proxyphylline | talampicillin | 0.756 | 0.777 |
| etacrynic_acid | oxytetracycline | 0.756 | 0.750 |
| bepridil | rimexolone | 0.756 | 0.761 |
| flufenamic_acid | ornidazole | 0.757 | 0.665 |
| rimexolone | tropicamide | 0.757 | 0.737 |
| niclosamide | tribenoside | 0.757 | 0.752 |
| isopropamide_iodide | ribostamycin | 0.757 | 0.782 |
| carbachol | norethisterone | 0.757 | 0.745 |
| lanatoside_C | loperamide | 0.757 | 0.842 |
| levonorgestrel | monobenzone | 0.757 | 0.702 |
| miconazole | rimexolone | 0.757 | 0.748 |
| butoconazole | oxetacaine | 0.757 | 0.714 |
| dacarbazine | desoxycortone | 0.757 | 0.766 |
| clomipramine | progesterone | 0.757 | 0.743 |
| clioquinol | clomipramine | 0.757 | 0.720 |
| cefalexin | piperidolate | 0.757 | 0.686 |
| doxazosin | methapyrilene | 0.757 | 0.784 |
| isoxsuprine | trichlormethiazide | 0.757 | 0.727 |
| alimemazine | clidinium_bromide | 0.757 | 0.713 |
| chlortetracycline | monobenzone | 0.757 | 0.758 |
| gliclazide | piromidic_acid | 0.757 | 0.733 |
| fluvoxamine | proscillaridin | 0.757 | 0.777 |
| cefalexin | cinchocaine | 0.757 | 0.705 |
| digoxin | etoposide | 0.757 | 0.786 |
| isocarboxazid | nalidixic_acid | 0.757 | 0.670 |
| diltiazem | repaglinide | 0.757 | 0.736 |
| bepridil | sulconazole | 0.757 | 0.718 |

| | | | |
|---|---|---|---|
| fludroxycortide | sulconazole | 0.757 | 0.784 |
| dacarbazine | sulfamerazine | 0.757 | 0.691 |
| mefloquine | promazine | 0.757 | 0.714 |
| hydrocortisone | meglumine | 0.757 | 0.749 |
| cefalotin | verteporfin | 0.757 | 0.787 |
| acebutolol | meclocycline | 0.757 | 0.709 |
| etomidate | spaglumic_acid | 0.757 | 0.742 |
| methazolamide | sulfametoxydiazine | 0.757 | 0.664 |
| ivermectin | loperamide | 0.757 | 0.818 |
| miconazole | progesterone | 0.757 | 0.726 |
| clioquinol | metergoline | 0.757 | 0.761 |
| latamoxef | mitoxantrone | 0.757 | 0.766 |
| fenoprofen | hydralazine | 0.757 | 0.684 |
| demecolcine | disulfiram | 0.757 | 0.726 |
| benfotiamine | piperacillin | 0.757 | 0.720 |
| dipyridamole | trioxysalen | 0.757 | 0.775 |
| bromopride | trazodone | 0.757 | 0.682 |
| etamivan | propafenone | 0.757 | 0.734 |
| emetine | primaquine | 0.757 | 0.730 |
| piribedil | sulfaphenazole | 0.757 | 0.676 |
| acenocoumarol | isoconazole | 0.757 | 0.701 |
| moroxydine | tiapride | 0.757 | 0.708 |
| omeprazole | probenecid | 0.757 | 0.687 |
| astemizole | profenamine | 0.757 | 0.736 |
| dilazep | sulfamethoxazole | 0.757 | 0.803 |
| amodiaquine | etacrynic_acid | 0.757 | 0.656 |
| omeprazole | promazine | 0.757 | 0.743 |
| benfluorex | cefalexin | 0.757 | 0.653 |
| meropenem | propylthiouracil | 0.757 | 0.747 |
| amoxapine | niclosamide | 0.757 | 0.686 |
| sulfafurazole | tobramycin | 0.757 | 0.779 |
| flupentixol | prochlorperazine | 0.757 | 0.661 |
| rifabutin | sodium_phenylbutyra | 0.757 | 0.868 |
| betaxolol | roxithromycin | 0.757 | 0.832 |
| amiodarone | chlorzoxazone | 0.757 | 0.794 |
| fenoprofen | phenoxybenzamine | 0.757 | 0.718 |
| miconazole | trimipramine | 0.757 | 0.665 |
| meticrane | trimetazidine | 0.757 | 0.702 |
| clomipramine | mefloquine | 0.757 | 0.743 |
| dacarbazine | sulfafurazole | 0.757 | 0.713 |
| cefoxitin | pyrithyldione | 0.757 | 0.799 |
| trimetazidine | zimeldine | 0.757 | 0.701 |
| ipratropium_bromide | ronidazole | 0.757 | 0.749 |
| dilazep | trimethoprim | 0.757 | 0.793 |
| amiodarone | flufenamic_acid | 0.757 | 0.744 |
| etofenamate | levonorgestrel | 0.757 | 0.747 |
| (-)-catechin | acetylsalicylic_acid | 0.757 | 0.714 |
| diltiazem | medrysone | 0.757 | 0.742 |
| benfluorex | iohexol | 0.757 | 0.783 |
| hydroflumethiazide | levocabastine | 0.757 | 0.779 |

| | | | |
|---|---|---|---|
| norethisterone | pivampicillin | 0.757 | 0.769 |
| pentoxyverine | roxithromycin | 0.757 | 0.817 |
| promazine | triflusal | 0.757 | 0.747 |
| latamoxef | promazine | 0.757 | 0.810 |
| sulfametoxydiazine | tropicamide | 0.757 | 0.680 |
| dipyridamole | ipratropium_bromide | 0.757 | 0.770 |
| spironolactone | vorinostat | 0.757 | 0.749 |
| benfluorex | perhexiline | 0.757 | 0.776 |
| fenoprofen | practolol | 0.758 | 0.715 |
| gliclazide | latamoxef | 0.758 | 0.710 |
| moxonidine | urapidil | 0.758 | 0.704 |
| captopril | levodopa | 0.758 | 0.684 |
| dipivefrine | ketanserin | 0.758 | 0.759 |
| clomifene | fendiline | 0.758 | 0.717 |
| lymecycline | sulfaphenazole | 0.758 | 0.771 |
| chloramphenicol | trimetazidine | 0.758 | 0.707 |
| cetirizine | pyrvinium | 0.758 | 0.779 |
| chlorzoxazone | propafenone | 0.758 | 0.764 |
| lanatoside_C | mebendazole | 0.758 | 0.858 |
| buflomedil | sulfaguanidine | 0.758 | 0.676 |
| chlorcyclizine | fenbendazole | 0.758 | 0.739 |
| promazine | roxithromycin | 0.758 | 0.859 |
| buflomedil | piperidolate | 0.758 | 0.658 |
| cefalexin | phenoxybenzamine | 0.758 | 0.719 |
| digoxin | mebendazole | 0.758 | 0.830 |
| gliclazide | rolitetracycline | 0.758 | 0.709 |
| etofenamate | roxithromycin | 0.758 | 0.813 |
| ciclosporin | dosulepin | 0.758 | 0.896 |
| clioquinol | rescinnamine | 0.758 | 0.803 |
| lymecycline | proxyphylline | 0.758 | 0.789 |
| fenoprofen | promethazine | 0.758 | 0.714 |
| cinchocaine | daunorubicin | 0.758 | 0.708 |
| ketorolac | meticrane | 0.758 | 0.694 |
| demecolcine | mefloquine | 0.758 | 0.720 |
| clofazimine | clomipramine | 0.758 | 0.716 |
| benzylpenicillin | gliclazide | 0.758 | 0.665 |
| bezafibrate | estriol | 0.758 | 0.736 |
| loperamide | verapamil | 0.758 | 0.726 |
| alfaxalone | promethazine | 0.758 | 0.766 |
| clotrimazole | tioguanine | 0.758 | 0.764 |
| econazole | ivermectin | 0.758 | 0.849 |
| mebendazole | mefloquine | 0.758 | 0.706 |
| cetirizine | mitoxantrone | 0.758 | 0.729 |
| practolol | xylometazoline | 0.758 | 0.743 |
| fluphenazine | perhexiline | 0.758 | 0.767 |
| etamsylate | fluorometholone | 0.758 | 0.783 |
| carbachol | milrinone | 0.758 | 0.717 |
| miconazole | propofol | 0.758 | 0.777 |
| mycophenolic_acid | rifampicin | 0.758 | 0.797 |
| piromidic_acid | simvastatin | 0.758 | 0.745 |

| | | | |
|---|---|---|---|
| talampicillin | zomepirac | 0.758 | 0.726 |
| promazine | pyrvinium | 0.758 | 0.767 |
| bromocriptine | oxamic_acid | 0.758 | 0.896 |
| azacitidine | deptropine | 0.758 | 0.773 |
| pentoxyverine | sulfametoxydiazine | 0.758 | 0.713 |
| cyproheptadine | perhexiline | 0.758 | 0.664 |
| doxorubicin | sulfamerazine | 0.758 | 0.746 |
| ciclosporin | terfenadine | 0.758 | 0.839 |
| acepromazine | omeprazole | 0.758 | 0.730 |
| saquinavir | tropine | 0.758 | 0.877 |
| clomipramine | tyloxapol | 0.758 | 0.868 |
| diclofenac | glibenclamide | 0.758 | 0.774 |
| fludroxycortide | griseofulvin | 0.758 | 0.727 |
| flufenamic_acid | gliclazide | 0.758 | 0.680 |
| sulfametoxydiazine | sulfinpyrazone | 0.758 | 0.696 |
| bisacodyl | natamycin | 0.758 | 0.801 |
| protriptyline | thioridazine | 0.758 | 0.685 |
| chlorhexidine | clopamide | 0.758 | 0.787 |
| ethosuximide | sulconazole | 0.758 | 0.798 |
| ciclopirox | clofazimine | 0.758 | 0.730 |
| ketanserin | pipemidic_acid | 0.758 | 0.680 |
| clomipramine | reserpine | 0.758 | 0.779 |
| ethosuximide | oxymetazoline | 0.758 | 0.785 |
| trioxysalen | urapidil | 0.758 | 0.769 |
| acetylsalicylic_acid | gliclazide | 0.758 | 0.729 |
| altretamine | urapidil | 0.758 | 0.760 |
| daunorubicin | levonorgestrel | 0.758 | 0.762 |
| phenazopyridine | prenylamine | 0.758 | 0.751 |
| alprenolol | mebeverine | 0.758 | 0.735 |
| clomipramine | trazodone | 0.758 | 0.766 |
| pentoxyverine | piribedil | 0.758 | 0.729 |
| pyrvinium | raloxifene | 0.758 | 0.772 |
| calcium_folinate | cefamandole | 0.758 | 0.691 |
| bepridil | verteporfin | 0.758 | 0.811 |
| dipivefrine | metergoline | 0.758 | 0.755 |
| dextromethorphan | doxazosin | 0.758 | 0.796 |
| lanatoside_C | mepacrine | 0.758 | 0.865 |
| methylergometrine | pipemidic_acid | 0.758 | 0.681 |
| molindone | moxonidine | 0.758 | 0.657 |
| acepromazine | cefalotin | 0.758 | 0.744 |
| cefalotin | procaine | 0.758 | 0.716 |
| azacitidine | suloctidil | 0.758 | 0.775 |
| monobenzone | ronidazole | 0.758 | 0.676 |
| medrysone | menadione | 0.758 | 0.767 |
| ambroxol | fludrocortisone | 0.758 | 0.726 |
| mycophenolic_acid | vorinostat | 0.758 | 0.695 |
| oxedrine | ramipril | 0.758 | 0.786 |
| loperamide | tioguanine | 0.758 | 0.834 |
| mitoxantrone | rimexolone | 0.758 | 0.762 |
| ketoconazole | suloctidil | 0.758 | 0.767 |

| | | | |
|---|---|---|---|
| etamsylate | roxithromycin | 0.759 | 0.879 |
| bromocriptine | deptropine | 0.759 | 0.778 |
| amiodarone | tyloxapol | 0.759 | 0.835 |
| cinchocaine | triflusal | 0.759 | 0.739 |
| colchicine | menadione | 0.759 | 0.731 |
| mercaptopurine | methotrexate | 0.759 | 0.742 |
| bepridil | doxazosin | 0.759 | 0.778 |
| perhexiline | sirolimus | 0.759 | 0.874 |
| meptazinol | nafcillin | 0.759 | 0.772 |
| dacarbazine | tranylcypromine | 0.759 | 0.700 |
| chlorzoxazone | levamisole | 0.759 | 0.652 |
| doxorubicin | sulfamethoxypyridazir | 0.759 | 0.750 |
| medrysone | zimeldine | 0.759 | 0.742 |
| benzylpenicillin | sulconazole | 0.759 | 0.748 |
| chlorprothixene | vorinostat | 0.759 | 0.754 |
| acetylsalicylic_acid | doxorubicin | 0.759 | 0.767 |
| chlorzoxazone | piperidolate | 0.759 | 0.720 |
| piromidic_acid | trazodone | 0.759 | 0.717 |
| bumetanide | enalapril | 0.759 | 0.703 |
| desoxycortone | piperidolate | 0.759 | 0.763 |
| albendazole | fluphenazine | 0.759 | 0.735 |
| tobramycin | trimethadione | 0.759 | 0.860 |
| repaglinide | tobramycin | 0.759 | 0.759 |
| benfotiamine | meticrane | 0.759 | 0.770 |
| metamizole_sodium | procaine | 0.759 | 0.657 |
| albendazole | gefitinib | 0.759 | 0.690 |
| deferoxamine | metamizole_sodium | 0.759 | 0.817 |
| etomidate | trioxysalen | 0.759 | 0.701 |
| mepyramine | vinpocetine | 0.759 | 0.723 |
| daunorubicin | levamisole | 0.759 | 0.787 |
| etomidate | trimetazidine | 0.759 | 0.704 |
| flunarizine | talampicillin | 0.759 | 0.745 |
| fluvoxamine | syrosingopine | 0.759 | 0.786 |
| medrysone | pyrazinamide | 0.759 | 0.803 |
| mifepristone | rescinnamine | 0.759 | 0.782 |
| benperidol | promethazine | 0.759 | 0.753 |
| milrinone | pridinol | 0.759 | 0.750 |
| ketanserin | nilutamide | 0.759 | 0.699 |
| mepacrine | proscillaridin | 0.759 | 0.791 |
| nortriptyline | thioproperazine | 0.759 | 0.694 |
| salbutamol | trichlormethiazide | 0.759 | 0.658 |
| liothyronine | vidarabine | 0.759 | 0.726 |
| hydralazine | sulfamethoxazole | 0.759 | 0.671 |
| propafenone | reserpine | 0.759 | 0.717 |
| glibenclamide | vidarabine | 0.759 | 0.763 |
| sirolimus | troglitazone | 0.759 | 0.825 |
| doxorubicin | zimeldine | 0.759 | 0.743 |
| metyrapone | phenoxybenzamine | 0.759 | 0.717 |
| primaquine | tolazoline | 0.759 | 0.709 |
| cefalotin | dacarbazine | 0.759 | 0.742 |

| | | | |
|---|---|---|---|
| diltiazem | oxetacaine | 0.759 | 0.731 |
| flufenamic_acid | fluorometholone | 0.759 | 0.701 |
| cyanocobalamin | sulfaphenazole | 0.759 | 0.877 |
| bromocriptine | pyrvinium | 0.759 | 0.763 |
| cyproterone | fenoprofen | 0.759 | 0.718 |
| doxazosin | remoxipride | 0.759 | 0.734 |
| chlorzoxazone | liothyronine | 0.759 | 0.780 |
| calcium_pantothenate | dacarbazine | 0.759 | 0.825 |
| chlorambucil | flutamide | 0.759 | 0.722 |
| metergoline | tribenoside | 0.759 | 0.718 |
| flavoxate | remoxipride | 0.759 | 0.694 |
| benfotiamine | doxazosin | 0.759 | 0.716 |
| (-)-atenolol | tyloxapol | 0.759 | 0.866 |
| milrinone | sulfametoxydiazine | 0.759 | 0.690 |
| bepridil | labetalol | 0.759 | 0.763 |
| etoposide | methotrexate | 0.759 | 0.723 |
| dorzolamide | fenoprofen | 0.759 | 0.726 |
| fluphenazine | raloxifene | 0.759 | 0.698 |
| acepromazine | repaglinide | 0.759 | 0.753 |
| doxylamine | minaprine | 0.759 | 0.707 |
| nalidixic_acid | trimethoprim | 0.759 | 0.664 |
| cefsulodin | tobramycin | 0.759 | 0.732 |
| glipizide | naloxone | 0.759 | 0.798 |
| metrifonate | moxisylyte | 0.759 | 0.784 |
| terconazole | trifluoperazine | 0.759 | 0.754 |
| astemizole | mepacrine | 0.759 | 0.723 |
| latamoxef | norfloxacin | 0.759 | 0.757 |
| meptazinol | phenazone | 0.759 | 0.723 |
| clobetasol | dacarbazine | 0.759 | 0.766 |
| metyrapone | roxithromycin | 0.759 | 0.853 |
| desoxycortone | verteporfin | 0.759 | 0.801 |
| meticrane | ursodeoxycholic_acid | 0.759 | 0.758 |
| perphenazine | vorinostat | 0.759 | 0.703 |
| piromidic_acid | spectinomycin | 0.759 | 0.725 |
| iocetamic_acid | iodixanol | 0.759 | 0.821 |
| nortriptyline | suloctidil | 0.759 | 0.764 |
| bromperidol | imipenem | 0.759 | 0.746 |
| bisacodyl | methazolamide | 0.759 | 0.758 |
| galantamine | meptazinol | 0.759 | 0.694 |
| clobetasol | zimeldine | 0.759 | 0.761 |
| ipratropium_bromide | natamycin | 0.759 | 0.811 |
| meclofenoxate | rimexolone | 0.759 | 0.763 |
| milrinone | procaine | 0.759 | 0.696 |
| clofazimine | profenamine | 0.759 | 0.682 |
| flufenamic_acid | fluspirilene | 0.759 | 0.728 |
| buspirone | carbachol | 0.759 | 0.824 |
| desoxycortone | monobenzone | 0.759 | 0.717 |
| flumetasone | sulfamerazine | 0.759 | 0.729 |
| capsaicin | milrinone | 0.759 | 0.686 |
| oxetacaine | promazine | 0.760 | 0.796 |

| | | | |
|---|---|---|---|
| clomipramine | terbutaline | 0.760 | 0.755 |
| clomipramine | zimeldine | 0.760 | 0.655 |
| metacycline | tyloxapol | 0.760 | 0.839 |
| indapamide | mexiletine | 0.760 | 0.714 |
| fluvoxamine | oxybuprocaine | 0.760 | 0.736 |
| cefixime | medrysone | 0.760 | 0.722 |
| amantadine | tolnaftate | 0.760 | 0.798 |
| phenoxybenzamine | rifabutin | 0.760 | 0.837 |
| bromperidol | fenoprofen | 0.760 | 0.736 |
| buflomedil | sulfamerazine | 0.760 | 0.662 |
| glibenclamide | omeprazole | 0.760 | 0.717 |
| tocainide | zuclopenthixol | 0.760 | 0.748 |
| gliclazide | nitrofurantoin | 0.760 | 0.729 |
| mefloquine | nomifensine | 0.760 | 0.715 |
| (-)-catechin | cefalotin | 0.760 | 0.708 |
| piperidolate | rolitetracycline | 0.760 | 0.758 |
| bisacodyl | mesoridazine | 0.760 | 0.719 |
| desoxycortone | repaglinide | 0.760 | 0.727 |
| liothyronine | milrinone | 0.760 | 0.736 |
| meptazinol | methapyrilene | 0.760 | 0.676 |
| cetirizine | desoxycortone | 0.760 | 0.747 |
| astemizole | cyclobenzaprine | 0.760 | 0.731 |
| sulfaphenazole | ursodeoxycholic_acid | 0.760 | 0.752 |
| benzethonium_chloride | fluspirilene | 0.760 | 0.750 |
| miconazole | oxetacaine | 0.760 | 0.713 |
| etofenamate | pipemidic_acid | 0.760 | 0.662 |
| paracetamol | sulconazole | 0.760 | 0.779 |
| budesonide | flufenamic_acid | 0.760 | 0.736 |
| cyproterone | omeprazole | 0.760 | 0.754 |
| meclofenoxate | natamycin | 0.760 | 0.802 |
| calcium_pantothenate | fenoprofen | 0.760 | 0.802 |
| omeprazole | talampicillin | 0.760 | 0.712 |
| omeprazole | tranylcypromine | 0.760 | 0.726 |
| etoposide | lomustine | 0.760 | 0.807 |
| clomipramine | ketanserin | 0.760 | 0.777 |
| isoconazole | perphenazine | 0.760 | 0.729 |
| betahistine | tiapride | 0.760 | 0.730 |
| mesoridazine | phenindione | 0.760 | 0.744 |
| etofenamate | sulfaphenazole | 0.760 | 0.728 |
| flupentixol | sulconazole | 0.760 | 0.731 |
| diazoxide | topiramate | 0.760 | 0.758 |
| benzethonium_chloride | emetine | 0.760 | 0.762 |
| disulfiram | raloxifene | 0.760 | 0.786 |
| isoconazole | pyrantel | 0.760 | 0.713 |
| cefazolin | nalidixic_acid | 0.760 | 0.764 |
| benfotiamine | sulconazole | 0.760 | 0.736 |
| nabumetone | procaine | 0.760 | 0.670 |
| norfloxacin | ranitidine | 0.760 | 0.706 |
| clotrimazole | perphenazine | 0.760 | 0.747 |
| bepridil | talampicillin | 0.760 | 0.787 |

| | | | |
|---|---|---|---|
| gliclazide | sulfaphenazole | 0.760 | 0.671 |
| cetirizine | ethotoin | 0.760 | 0.758 |
| fluspirilene | proscillaridin | 0.760 | 0.736 |
| levamisole | piroxicam | 0.760 | 0.702 |
| mitoxantrone | sulfamethoxazole | 0.760 | 0.770 |
| amphotericin_B | trifluoperazine | 0.760 | 0.830 |
| acepromazine | meclozine | 0.760 | 0.736 |
| desoxycortone | sulfametoxydiazine | 0.760 | 0.755 |
| bumetanide | bupivacaine | 0.760 | 0.734 |
| (-)-catechin | betaxolol | 0.760 | 0.721 |
| deferoxamine | fluocinonide | 0.760 | 0.810 |
| aminoglutethimide | fenofibrate | 0.760 | 0.762 |
| nitrofural | primidone | 0.760 | 0.737 |
| griseofulvin | proscillaridin | 0.760 | 0.790 |
| cyproheptadine | lynestrenol | 0.760 | 0.723 |
| clotrimazole | oxyphenbutazone | 0.760 | 0.720 |
| ketanserin | perhexiline | 0.760 | 0.788 |
| tobramycin | zimeldine | 0.760 | 0.803 |
| decitabine | naltrexone | 0.760 | 0.724 |
| atovaquone | betahistine | 0.760 | 0.747 |
| bufexamac | sulconazole | 0.760 | 0.746 |
| perhexiline | syrosingopine | 0.760 | 0.842 |
| chlorzoxazone | methapyrilene | 0.760 | 0.738 |
| cetirizine | ronidazole | 0.760 | 0.734 |
| carbachol | estriol | 0.760 | 0.752 |
| doxazosin | procaine | 0.760 | 0.690 |
| meclofenoxate | piribedil | 0.760 | 0.666 |
| alfuzosin | promazine | 0.760 | 0.721 |
| betahistine | galantamine | 0.760 | 0.717 |
| ketoprofen | medrysone | 0.760 | 0.690 |
| cyproterone | sulfaphenazole | 0.760 | 0.746 |
| bethanechol | risperidone | 0.760 | 0.820 |
| molindone | sotalol | 0.760 | 0.716 |
| albendazole | oxytetracycline | 0.760 | 0.758 |
| levamisole | sulconazole | 0.760 | 0.734 |
| gefitinib | metergoline | 0.760 | 0.717 |
| monobenzone | norethisterone | 0.760 | 0.696 |
| clomipramine | procaine | 0.760 | 0.736 |
| cetirizine | sulfaguanidine | 0.760 | 0.762 |
| estriol | urapidil | 0.760 | 0.743 |
| albendazole | cinnarizine | 0.760 | 0.758 |
| fluspirilene | progesterone | 0.760 | 0.696 |
| fendiline | zuclopenthixol | 0.760 | 0.739 |
| miconazole | propafenone | 0.760 | 0.733 |
| deptropine | terfenadine | 0.760 | 0.753 |
| lansoprazole | moracizine | 0.760 | 0.712 |
| buflomedil | dipyridamole | 0.760 | 0.736 |
| mesoridazine | thiocolchicoside | 0.760 | 0.734 |
| betahistine | trazodone | 0.760 | 0.718 |
| nafcillin | theobromine | 0.760 | 0.743 |

| | | | |
|---|---|---|---|
| dipyridamole | roxithromycin | 0.760 | 0.789 |
| demecolcine | metergoline | 0.760 | 0.694 |
| bromopride | propylthiouracil | 0.760 | 0.698 |
| sulfafurazole | thiocolchicoside | 0.760 | 0.783 |
| meclozine | miconazole | 0.760 | 0.708 |
| glibenclamide | phenoxybenzamine | 0.760 | 0.781 |
| etofenamate | verteporfin | 0.760 | 0.721 |
| dipyridamole | syrosingopine | 0.760 | 0.775 |
| chlortetracycline | practolol | 0.760 | 0.752 |
| enoxacin | mefloquine | 0.760 | 0.691 |
| amitriptyline | niclosamide | 0.760 | 0.727 |
| amiodarone | bromocriptine | 0.760 | 0.746 |
| milrinone | roxithromycin | 0.760 | 0.864 |
| haloperidol | pyrvinium | 0.760 | 0.773 |
| fluoxetine | nilutamide | 0.760 | 0.689 |
| metacycline | omeprazole | 0.761 | 0.685 |
| oxprenolol | talampicillin | 0.761 | 0.740 |
| daunorubicin | vidarabine | 0.761 | 0.661 |
| clindamycin | liothyronine | 0.761 | 0.706 |
| bisacodyl | piribedil | 0.761 | 0.705 |
| semustine | trifluoperazine | 0.761 | 0.749 |
| alfaxalone | flavoxate | 0.761 | 0.752 |
| (-)-catechin | disulfiram | 0.761 | 0.677 |
| chlorcyclizine | quinisocaine | 0.761 | 0.654 |
| doxazosin | mesalazine | 0.761 | 0.742 |
| (-)-catechin | sulconazole | 0.761 | 0.744 |
| benfotiamine | metamizole_sodium | 0.761 | 0.686 |
| oxybuprocaine | promethazine | 0.761 | 0.746 |
| capsaicin | dacarbazine | 0.761 | 0.709 |
| betaxolol | bisacodyl | 0.761 | 0.739 |
| flufenamic_acid | omeprazole | 0.761 | 0.699 |
| benzethonium_chloride | disulfiram | 0.761 | 0.790 |
| chlorambucil | monobenzone | 0.761 | 0.706 |
| cetirizine | methapyrilene | 0.761 | 0.704 |
| nortriptyline | phenazopyridine | 0.761 | 0.724 |
| pentoxyverine | repaglinide | 0.761 | 0.728 |
| dacarbazine | tobramycin | 0.761 | 0.805 |
| phensuximide | piribedil | 0.761 | 0.714 |
| diltiazem | mitoxantrone | 0.761 | 0.737 |
| dinoprost | melatonin | 0.761 | 0.759 |
| doxorubicin | tyloxapol | 0.761 | 0.807 |
| acetohexamide | cinchocaine | 0.761 | 0.721 |
| dorzolamide | piperidolate | 0.761 | 0.722 |
| guanfacine | sulfafurazole | 0.761 | 0.675 |
| betaxolol | medrysone | 0.761 | 0.763 |
| carbachol | meropenem | 0.761 | 0.804 |
| acepromazine | zomepirac | 0.761 | 0.655 |
| proxyphylline | roxithromycin | 0.761 | 0.848 |
| dienestrol | lomustine | 0.761 | 0.759 |
| sulfaphenazole | verteporfin | 0.761 | 0.800 |

| | | | |
|---|---|---|---|
| bisacodyl | diltiazem | 0.761 | 0.729 |
| promazine | tyloxapol | 0.761 | 0.873 |
| mefloquine | ticlopidine | 0.761 | 0.734 |
| metoprolol | succinylsulfathiazole | 0.761 | 0.707 |
| bendroflumethiazide | doxazosin | 0.761 | 0.690 |
| promazine | trazodone | 0.761 | 0.741 |
| betaxolol | chlortetracycline | 0.761 | 0.761 |
| flecainide | piperacillin | 0.761 | 0.723 |
| pivampicillin | tridihexethyl | 0.761 | 0.776 |
| lomustine | pyrvinium | 0.761 | 0.818 |
| carbachol | rimexolone | 0.761 | 0.766 |
| digoxin | griseofulvin | 0.761 | 0.846 |
| oxprenolol | trimetazidine | 0.761 | 0.710 |
| colecalciferol | progesterone | 0.761 | 0.709 |
| miconazole | reserpine | 0.761 | 0.770 |
| benperidol | chlorzoxazone | 0.761 | 0.753 |
| sulfafurazole | trazodone | 0.761 | 0.716 |
| demecolcine | nilutamide | 0.761 | 0.710 |
| talampicillin | trioxysalen | 0.761 | 0.812 |
| rimexolone | sulpiride | 0.761 | 0.734 |
| atovaquone | sulconazole | 0.761 | 0.723 |
| dydrogesterone | methylergometrine | 0.761 | 0.699 |
| ifenprodil | ipratropium_bromide | 0.761 | 0.695 |
| daunorubicin | monobenzone | 0.761 | 0.770 |
| metacycline | repaglinide | 0.761 | 0.722 |
| disulfiram | mepacrine | 0.761 | 0.750 |
| buspirone | oxantel | 0.761 | 0.764 |
| benzethonium_chloride | mometasone | 0.761 | 0.796 |
| doxazosin | lymecycline | 0.761 | 0.753 |
| etacrynic_acid | oxyphenbutazone | 0.761 | 0.676 |
| cetirizine | procyclidine | 0.761 | 0.669 |
| capsaicin | metamizole_sodium | 0.761 | 0.733 |
| maprotiline | suloctidil | 0.761 | 0.748 |
| bisacodyl | meticrane | 0.761 | 0.770 |
| gliclazide | tolfenamic_acid | 0.761 | 0.714 |
| diltiazem | pipemidic_acid | 0.761 | 0.748 |
| benfluorex | phensuximide | 0.761 | 0.761 |
| diltiazem | estriol | 0.761 | 0.747 |
| acepromazine | medrysone | 0.761 | 0.711 |
| oxolinic_acid | rimexolone | 0.761 | 0.725 |
| daunorubicin | propylthiouracil | 0.761 | 0.750 |
| (-)-catechin | rimexolone | 0.761 | 0.745 |
| natamycin | sulfametoxydiazine | 0.761 | 0.813 |
| econazole | flupentixol | 0.761 | 0.731 |
| acetylsalicylic_acid | estriol | 0.761 | 0.758 |
| diltiazem | sulfametoxydiazine | 0.761 | 0.704 |
| cefalexin | pyrazinamide | 0.761 | 0.750 |
| norethisterone | pentoxyverine | 0.761 | 0.775 |
| chloramphenicol | tobramycin | 0.761 | 0.750 |
| cefalotin | latamoxef | 0.761 | 0.669 |

| | | | |
|---|---|---|---|
| mefloquine | quinisocaine | 0.761 | 0.700 |
| pivampicillin | promazine | 0.761 | 0.802 |
| riluzole | salbutamol | 0.761 | 0.671 |
| cefalexin | griseofulvin | 0.761 | 0.715 |
| natamycin | sertaconazole | 0.761 | 0.812 |
| phenoxybenzamine | sulfamethoxypyridazir | 0.761 | 0.717 |
| fluphenazine | ivermectin | 0.761 | 0.809 |
| bromocriptine | ketanserin | 0.761 | 0.706 |
| bisacodyl | terguride | 0.762 | 0.738 |
| acepromazine | cefalexin | 0.762 | 0.749 |
| cetirizine | pentamidine | 0.762 | 0.743 |
| exemestane | glibenclamide | 0.762 | 0.775 |
| perphenazine | syrosingopine | 0.762 | 0.756 |
| niridazole | tyloxapol | 0.762 | 0.892 |
| bromocriptine | chlortetracycline | 0.762 | 0.716 |
| promethazine | sulfametoxydiazine | 0.762 | 0.742 |
| mebendazole | podophyllotoxin | 0.762 | 0.723 |
| amiodarone | niridazole | 0.762 | 0.774 |
| capsaicin | sulfaphenazole | 0.762 | 0.727 |
| noretynodrel | terfenadine | 0.762 | 0.752 |
| carbachol | digoxin | 0.762 | 0.871 |
| procarbazine | proguanil | 0.762 | 0.667 |
| isocarboxazid | spiramycin | 0.762 | 0.859 |
| chloramphenicol | sulconazole | 0.762 | 0.751 |
| griseofulvin | metaraminol | 0.762 | 0.738 |
| betaxolol | ketanserin | 0.762 | 0.732 |
| doxazosin | ursodeoxycholic_acid | 0.762 | 0.713 |
| doxorubicin | hydralazine | 0.762 | 0.761 |
| cetirizine | pipemidic_acid | 0.762 | 0.753 |
| pargyline | talampicillin | 0.762 | 0.789 |
| bendroflumethiazide | sulconazole | 0.762 | 0.739 |
| cefalotin | sulfametoxydiazine | 0.762 | 0.709 |
| propylthiouracil | reserpine | 0.762 | 0.798 |
| hexetidine | phenoxybenzamine | 0.762 | 0.718 |
| meropenem | sulconazole | 0.762 | 0.758 |
| fluspirilene | terguride | 0.762 | 0.709 |
| adipiodone | flucloxacillin | 0.762 | 0.731 |
| pivampicillin | rimexolone | 0.762 | 0.734 |
| hydralazine | tyloxapol | 0.762 | 0.897 |
| amiodarone | zimeldine | 0.762 | 0.743 |
| norethisterone | trioxysalen | 0.762 | 0.698 |
| molindone | sulconazole | 0.762 | 0.718 |
| propafenone | trioxysalen | 0.762 | 0.726 |
| estriol | etomidate | 0.762 | 0.707 |
| flavoxate | flunarizine | 0.762 | 0.728 |
| metamizole_sodium | metyrapone | 0.762 | 0.664 |
| cefalotin | metacycline | 0.762 | 0.713 |
| clotrimazole | propofol | 0.762 | 0.719 |
| bromperidol | pyrazinamide | 0.762 | 0.749 |
| clomipramine | guanethidine | 0.762 | 0.742 |

| | | | |
|---|---|---|---|
| etacrynic_acid | fenoterol | 0.762 | 0.719 |
| dipyridamole | menadione | 0.762 | 0.799 |
| imipramine | loperamide | 0.762 | 0.765 |
| methotrexate | trifluridine | 0.762 | 0.755 |
| pyrvinium | talampicillin | 0.762 | 0.790 |
| acepromazine | piperidolate | 0.762 | 0.703 |
| hydrocortisone | mesoridazine | 0.762 | 0.769 |
| buflomedil | omeprazole | 0.762 | 0.660 |
| doxazosin | tranylcypromine | 0.762 | 0.799 |
| hexetidine | loperamide | 0.762 | 0.735 |
| carbachol | hydrocortisone | 0.762 | 0.795 |
| doxazosin | ronidazole | 0.762 | 0.718 |
| fluspirilene | menadione | 0.762 | 0.791 |
| triflusal | vidarabine | 0.762 | 0.653 |
| piperidolate | scopolamine | 0.762 | 0.651 |
| estriol | pyrazinamide | 0.762 | 0.720 |
| molsidomine | piracetam | 0.762 | 0.722 |
| amoxicillin | paracetamol | 0.762 | 0.732 |
| bezafibrate | metamizole_sodium | 0.762 | 0.689 |
| milrinone | verteporfin | 0.762 | 0.824 |
| (-)-catechin | estriol | 0.762 | 0.665 |
| piperidolate | piribedil | 0.762 | 0.716 |
| clotrimazole | fluphenazine | 0.762 | 0.763 |
| medrysone | simvastatin | 0.762 | 0.733 |
| natamycin | oxprenolol | 0.762 | 0.824 |
| norethisterone | trimetazidine | 0.762 | 0.717 |
| perhexiline | sulconazole | 0.762 | 0.724 |
| butoconazole | syrosingopine | 0.762 | 0.779 |
| sulfametoxydiazine | talampicillin | 0.762 | 0.745 |
| trazodone | tropicamide | 0.762 | 0.753 |
| doxylamine | procainamide | 0.762 | 0.673 |
| etomidate | tolfenamic_acid | 0.762 | 0.675 |
| acetylsalicylic_acid | benperidol | 0.762 | 0.735 |
| fluvoxamine | rescinnamine | 0.762 | 0.794 |
| pentetrazol | tiratricol | 0.762 | 0.791 |
| dexibuprofen | pipemidic_acid | 0.762 | 0.692 |
| bisacodyl | liothyronine | 0.762 | 0.712 |
| sisomicin | tolazamide | 0.762 | 0.724 |
| estriol | propylthiouracil | 0.762 | 0.703 |
| cefepime | thiethylperazine | 0.762 | 0.774 |
| chlorphenamine | ivermectin | 0.762 | 0.868 |
| chlorzoxazone | trazodone | 0.762 | 0.728 |
| dipyridamole | tropicamide | 0.762 | 0.761 |
| nitrofurantoin | omeprazole | 0.762 | 0.681 |
| diltiazem | meclofenoxate | 0.762 | 0.717 |
| ciprofibrate | piperidolate | 0.762 | 0.698 |
| dipivefrine | phenoxybenzamine | 0.762 | 0.766 |
| flufenamic_acid | flunisolide | 0.762 | 0.727 |
| betaxolol | mitoxantrone | 0.762 | 0.727 |
| ivermectin | nomifensine | 0.762 | 0.885 |

| | | | |
|---|---|---|---|
| etomidate | meticrane | 0.762 | 0.721 |
| digoxin | felodipine | 0.762 | 0.853 |
| digoxin | hexetidine | 0.762 | 0.846 |
| azlocillin | methazolamide | 0.762 | 0.753 |
| glibenclamide | mitoxantrone | 0.762 | 0.738 |
| levamisole | talampicillin | 0.762 | 0.782 |
| etidronic_acid | tyloxapol | 0.762 | 0.913 |
| galantamine | tyloxapol | 0.762 | 0.874 |
| carteolol | cefadroxil | 0.762 | 0.688 |
| milrinone | propafenone | 0.762 | 0.748 |
| milrinone | natamycin | 0.762 | 0.856 |
| cycloserine | cyproterone | 0.762 | 0.836 |
| chlorphenesin | oxymetazoline | 0.762 | 0.704 |
| gliclazide | meropenem | 0.762 | 0.712 |
| bromocriptine | clobetasol | 0.762 | 0.755 |
| molindone | piromidic_acid | 0.762 | 0.665 |
| natamycin | sulfamethoxypyridazir | 0.762 | 0.812 |
| cefazolin | glycopyrronium_brom | 0.762 | 0.766 |
| (-)-atenolol | hydrocortisone | 0.762 | 0.688 |
| carbachol | miconazole | 0.762 | 0.736 |
| amoxapine | benzethonium_chloric | 0.763 | 0.797 |
| levamisole | piperidolate | 0.763 | 0.684 |
| cinchocaine | menadione | 0.763 | 0.691 |
| bufexamac | trifluoperazine | 0.763 | 0.735 |
| chloramphenicol | mephenesin | 0.763 | 0.702 |
| benzylpenicillin | etamsylate | 0.763 | 0.759 |
| fenoterol | syrosingopine | 0.763 | 0.765 |
| betaxolol | menadione | 0.763 | 0.759 |
| clotrimazole | cyproheptadine | 0.763 | 0.661 |
| bepridil | phenazopyridine | 0.763 | 0.761 |
| cetirizine | promazine | 0.763 | 0.733 |
| griseofulvin | practolol | 0.763 | 0.688 |
| betahistine | methylergometrine | 0.763 | 0.729 |
| ambroxol | fusidic_acid | 0.763 | 0.765 |
| prochlorperazine | trimipramine | 0.763 | 0.693 |
| moracizine | risperidone | 0.763 | 0.729 |
| cefuroxime | fluorometholone | 0.763 | 0.725 |
| metergoline | saquinavir | 0.763 | 0.748 |
| atovaquone | trioxysalen | 0.763 | 0.733 |
| imipramine | sulfacetamide | 0.763 | 0.743 |
| benzathine_benzylpenicillin | etilefrine | 0.763 | 0.728 |
| clotrimazole | semustine | 0.763 | 0.757 |
| apomorphine | zimeldine | 0.763 | 0.716 |
| clemastine | levomepromazine | 0.763 | 0.670 |
| metamizole_sodium | phenoxybenzamine | 0.763 | 0.715 |
| meclofenoxate | metyrapone | 0.763 | 0.699 |
| fipexide | gefitinib | 0.763 | 0.734 |
| beclometasone | paromomycin | 0.763 | 0.757 |
| phenoxybenzamine | prochlorperazine | 0.763 | 0.738 |
| mefloquine | pizotifen | 0.763 | 0.748 |

| | | | |
|---|---|---|---|
| bromperidol | phenoxybenzamine | 0.763 | 0.740 |
| mebendazole | prenylamine | 0.763 | 0.726 |
| diltiazem | tyloxapol | 0.763 | 0.842 |
| dacarbazine | etofenamate | 0.763 | 0.691 |
| dextromethorphan | tyloxapol | 0.763 | 0.888 |
| liothyronine | promethazine | 0.763 | 0.747 |
| amiodarone | pyrazinamide | 0.763 | 0.776 |
| astemizole | irinotecan | 0.763 | 0.757 |
| ronidazole | sulfamethoxypyridazir | 0.763 | 0.681 |
| dextromethorphan | paracetamol | 0.763 | 0.710 |
| amphotericin_B | dinoprost | 0.763 | 0.827 |
| gliclazide | naloxone | 0.763 | 0.754 |
| metergoline | pimethixene | 0.763 | 0.753 |
| oxprenolol | urapidil | 0.763 | 0.752 |
| amiodarone | cisapride | 0.763 | 0.739 |
| azacitidine | latamoxef | 0.763 | 0.752 |
| acepromazine | triflusal | 0.763 | 0.720 |
| fluorometholone | repaglinide | 0.763 | 0.711 |
| daunorubicin | dextromethorphan | 0.763 | 0.777 |
| meclofenamic_acid | pentoxifylline | 0.763 | 0.690 |
| sulconazole | sulfinpyrazone | 0.763 | 0.736 |
| doxazosin | scopolamine | 0.763 | 0.743 |
| benzathine_benzylpenicillin | memantine | 0.763 | 0.747 |
| bepridil | promethazine | 0.763 | 0.699 |
| dextromethorphan | meticrane | 0.763 | 0.740 |
| haloperidol | terconazole | 0.763 | 0.743 |
| cefotaxime | clindamycin | 0.763 | 0.701 |
| cyproheptadine | econazole | 0.763 | 0.689 |
| meptazinol | oxolinic_acid | 0.763 | 0.712 |
| bromperidol | gefitinib | 0.763 | 0.727 |
| flupentixol | phenoxybenzamine | 0.763 | 0.752 |
| metamizole_sodium | ronidazole | 0.763 | 0.678 |
| gliclazide | hydrocortisone | 0.763 | 0.725 |
| menadione | ticlopidine | 0.763 | 0.734 |
| dilazep | riluzole | 0.763 | 0.802 |
| fluspirilene | liothyronine | 0.763 | 0.697 |
| aminophenazone | pirenzepine | 0.763 | 0.680 |
| benfotiamine | doxorubicin | 0.763 | 0.677 |
| chlortalidone | mebeverine | 0.763 | 0.773 |
| doxazosin | fluocinonide | 0.763 | 0.751 |
| natamycin | roxithromycin | 0.763 | 0.756 |
| fenspiride | tranylcypromine | 0.763 | 0.705 |
| protriptyline | rifabutin | 0.763 | 0.847 |
| dipyridamole | latamoxef | 0.763 | 0.765 |
| isotretinoin | phenoxybenzamine | 0.763 | 0.766 |
| oxaprozin | verteporfin | 0.763 | 0.769 |
| budesonide | ronidazole | 0.763 | 0.774 |
| remoxipride | roxithromycin | 0.763 | 0.821 |
| glibenclamide | trioxysalen | 0.763 | 0.806 |
| nicergoline | trifluoperazine | 0.763 | 0.714 |

| | | | |
|---|---|---|---|
| guanfacine | pentoxyverine | 0.763 | 0.703 |
| chloramphenicol | tyloxapol | 0.763 | 0.867 |
| paroxetine | tinidazole | 0.763 | 0.720 |
| simvastatin | trimipramine | 0.763 | 0.750 |
| promazine | rimexolone | 0.763 | 0.766 |
| desoxycortone | latamoxef | 0.763 | 0.758 |
| imipenem | thiocolchicoside | 0.763 | 0.755 |
| fenoprofen | procaine | 0.763 | 0.672 |
| mebendazole | tribenoside | 0.763 | 0.741 |
| bisacodyl | ipratropium_bromide | 0.763 | 0.748 |
| flupentixol | fluvoxamine | 0.763 | 0.697 |
| emetine | methylergometrine | 0.763 | 0.727 |
| meptazinol | naltrexone | 0.763 | 0.659 |
| bisacodyl | milrinone | 0.763 | 0.756 |
| chlorzoxazone | ethosuximide | 0.763 | 0.654 |
| dihydroergocristine | perphenazine | 0.763 | 0.741 |
| meticrane | rolitetracycline | 0.763 | 0.755 |
| ciclopirox | zuclopenthixol | 0.763 | 0.740 |
| cefalotin | propylthiouracil | 0.763 | 0.737 |
| cimetidine | sulfathiazole | 0.763 | 0.702 |
| hydrocortisone | trioxysalen | 0.763 | 0.754 |
| sulfafurazole | tyloxapol | 0.763 | 0.881 |
| chlorcyclizine | lomustine | 0.763 | 0.753 |
| cinchocaine | oxybuprocaine | 0.763 | 0.671 |
| clomipramine | flufenamic_acid | 0.763 | 0.713 |
| alfuzosin | methapyrilene | 0.763 | 0.741 |
| flavoxate | nabumetone | 0.763 | 0.719 |
| cinchocaine | ipratropium_bromide | 0.763 | 0.716 |
| medrysone | metyrapone | 0.764 | 0.705 |
| omeprazole | propylthiouracil | 0.764 | 0.719 |
| naloxone | rimexolone | 0.764 | 0.712 |
| benzylpenicillin | flufenamic_acid | 0.764 | 0.685 |
| bepridil | thioproperazine | 0.764 | 0.714 |
| molindone | pentetrazol | 0.764 | 0.731 |
| bufexamac | repaglinide | 0.764 | 0.736 |
| mephenesin | trichlormethiazide | 0.764 | 0.688 |
| amiodarone | milrinone | 0.764 | 0.760 |
| benzocaine | tolnaftate | 0.764 | 0.720 |
| simvastatin | theophylline | 0.764 | 0.818 |
| meclofenoxate | triflusal | 0.764 | 0.732 |
| liothyronine | phenoxybenzamine | 0.764 | 0.748 |
| capsaicin | omeprazole | 0.764 | 0.678 |
| bisacodyl | sulfaphenazole | 0.764 | 0.738 |
| ifenprodil | sulfametoxydiazine | 0.764 | 0.707 |
| clomipramine | glibenclamide | 0.764 | 0.784 |
| nizatidine | paracetamol | 0.764 | 0.779 |
| cyproterone | milrinone | 0.764 | 0.740 |
| etamsylate | proscillaridin | 0.764 | 0.836 |
| cinchocaine | piperidolate | 0.764 | 0.693 |
| ifenprodil | nortriptyline | 0.764 | 0.763 |

| | | | |
|---|---|---|---|
| gefitinib | oxybutynin | 0.764 | 0.765 |
| levonorgestrel | promethazine | 0.764 | 0.756 |
| quinisocaine | trichlormethiazide | 0.764 | 0.729 |
| rolitetracycline | trazodone | 0.764 | 0.711 |
| lanatoside_C | lomustine | 0.764 | 0.876 |
| amoxapine | clotrimazole | 0.764 | 0.694 |
| ronidazole | sulfametoxydiazine | 0.764 | 0.656 |
| ioversol | talampicillin | 0.764 | 0.762 |
| capsaicin | doxorubicin | 0.764 | 0.754 |
| ivermectin | trimipramine | 0.764 | 0.873 |
| flufenamic_acid | levamisole | 0.764 | 0.662 |
| acetazolamide | moxisylyte | 0.764 | 0.734 |
| cetirizine | fluocinonide | 0.764 | 0.761 |
| betahistine | promazine | 0.764 | 0.697 |
| amantadine | scopolamine | 0.764 | 0.772 |
| piromidic_acid | tobramycin | 0.764 | 0.760 |
| carbachol | sulfametoxydiazine | 0.764 | 0.744 |
| clomifene | labetalol | 0.764 | 0.743 |
| meticrane | piperidolate | 0.764 | 0.744 |
| ofloxacin | sulfamethoxazole | 0.764 | 0.710 |
| levamisole | procaine | 0.764 | 0.659 |
| phenindione | triflusal | 0.764 | 0.708 |
| diltiazem | talampicillin | 0.764 | 0.748 |
| fluphenazine | pyrvinium | 0.764 | 0.760 |
| cetirizine | pargyline | 0.764 | 0.757 |
| diflunisal | pheniramine | 0.764 | 0.733 |
| pimozide | propafenone | 0.764 | 0.720 |
| etoposide | fendiline | 0.764 | 0.788 |
| mitoxantrone | oxybuprocaine | 0.764 | 0.700 |
| daunorubicin | primaquine | 0.764 | 0.675 |
| daunorubicin | trimetazidine | 0.764 | 0.749 |
| clomipramine | methazolamide | 0.764 | 0.771 |
| mepyramine | sulconazole | 0.764 | 0.692 |
| amiodarone | etacrynic_acid | 0.764 | 0.704 |
| loperamide | vorinostat | 0.764 | 0.724 |
| etomidate | sulfafurazole | 0.764 | 0.679 |
| bendroflumethiazide | spiramycin | 0.764 | 0.803 |
| mesoridazine | piracetam | 0.764 | 0.792 |
| etoposide | suloctidil | 0.764 | 0.782 |
| nitrofurantoin | piperidolate | 0.764 | 0.772 |
| dacarbazine | sulfamethoxypyridazir | 0.764 | 0.705 |
| prenylamine | verteporfin | 0.764 | 0.811 |
| cetirizine | talampicillin | 0.764 | 0.738 |
| talampicillin | torasemide | 0.764 | 0.725 |
| cetirizine | zimeldine | 0.764 | 0.695 |
| nafcillin | trichlormethiazide | 0.764 | 0.691 |
| benzathine_benzylpenicillin | zalcitabine | 0.764 | 0.735 |
| demecolcine | rimexolone | 0.764 | 0.721 |
| dipivefrine | pimozide | 0.764 | 0.774 |
| betaxolol | gliclazide | 0.764 | 0.689 |

| | | | |
|---|---|---|---|
| ciclosporin | cyproheptadine | 0.764 | 0.904 |
| dacarbazine | metamizole_sodium | 0.764 | 0.707 |
| gefitinib | perphenazine | 0.764 | 0.673 |
| cyproterone | oxprenolol | 0.764 | 0.723 |
| molindone | trichlormethiazide | 0.764 | 0.704 |
| trazodone | trimetazidine | 0.764 | 0.758 |
| benzethonium_chloride | prochlorperazine | 0.764 | 0.758 |
| pyrvinium | zuclopenthixol | 0.764 | 0.731 |
| daunorubicin | metyrapone | 0.764 | 0.751 |
| protriptyline | reserpine | 0.764 | 0.790 |
| daunorubicin | rimexolone | 0.764 | 0.756 |
| pyrimethamine | sulfathiazole | 0.764 | 0.689 |
| talampicillin | trimetazidine | 0.764 | 0.776 |
| clomifene | progesterone | 0.764 | 0.757 |
| estriol | meclozine | 0.764 | 0.783 |
| econazole | hexetidine | 0.764 | 0.693 |
| antazoline | etacrynic_acid | 0.764 | 0.721 |
| hydralazine | trimethoprim | 0.764 | 0.710 |
| levomepromazine | mometasone | 0.764 | 0.775 |
| menadione | terbutaline | 0.764 | 0.659 |
| astemizole | vorinostat | 0.764 | 0.748 |
| oxamic_acid | propafenone | 0.764 | 0.878 |
| cinchocaine | promethazine | 0.764 | 0.714 |
| latamoxef | piperidolate | 0.764 | 0.780 |
| carbachol | trazodone | 0.764 | 0.780 |
| butoconazole | progesterone | 0.764 | 0.737 |
| imipenem | sulfametoxydiazine | 0.764 | 0.704 |
| nitrofurantoin | tyloxapol | 0.764 | 0.882 |
| metyrapone | mitoxantrone | 0.764 | 0.747 |
| benzydamine | ketotifen | 0.764 | 0.730 |
| meptazinol | procainamide | 0.764 | 0.715 |
| doxazosin | pridinol | 0.764 | 0.766 |
| amiodarone | repaglinide | 0.764 | 0.745 |
| propafenone | xylometazoline | 0.764 | 0.736 |
| disulfiram | econazole | 0.764 | 0.750 |
| lomustine | primaquine | 0.764 | 0.670 |
| metamizole_sodium | pivampicillin | 0.764 | 0.725 |
| amiodarone | sulfamethoxypyridazir | 0.764 | 0.739 |
| molindone | omeprazole | 0.764 | 0.695 |
| etofenamate | promethazine | 0.764 | 0.723 |
| trioxysalen | tropicamide | 0.764 | 0.733 |
| rolitetracycline | tyloxapol | 0.764 | 0.825 |
| gefitinib | xylometazoline | 0.764 | 0.774 |
| methylergometrine | omeprazole | 0.764 | 0.673 |
| capsaicin | estriol | 0.764 | 0.722 |
| bepridil | pimethixene | 0.764 | 0.706 |
| glibenclamide | rimexolone | 0.764 | 0.751 |
| betahistine | sulfametoxydiazine | 0.764 | 0.671 |
| cyanocobalamin | mitoxantrone | 0.764 | 0.845 |
| fluspirilene | thioridazine | 0.764 | 0.743 |

| | | | |
|---|---|---|---|
| pentoxyverine | trazodone | 0.764 | 0.705 |
| deptropine | loperamide | 0.764 | 0.762 |
| astemizole | semustine | 0.764 | 0.748 |
| dilazep | mephenesin | 0.764 | 0.815 |
| fluocinonide | tropicamide | 0.765 | 0.764 |
| nialamide | phenelzine | 0.765 | 0.704 |
| fluvastatin | rimexolone | 0.765 | 0.758 |
| calcium_pantothenate | cetirizine | 0.765 | 0.793 |
| aciclovir | thiamphenicol | 0.765 | 0.705 |
| rimexolone | sulfamerazine | 0.765 | 0.755 |
| meropenem | sertaconazole | 0.765 | 0.752 |
| pivampicillin | sulfathiazole | 0.765 | 0.753 |
| clomipramine | dextromethorphan | 0.765 | 0.716 |
| amiodarone | diltiazem | 0.765 | 0.750 |
| bromocriptine | bromperidol | 0.765 | 0.734 |
| fenspiride | omeprazole | 0.765 | 0.662 |
| bromopride | cetirizine | 0.765 | 0.740 |
| meticrane | terguride | 0.765 | 0.768 |
| latamoxef | sulfametoxydiazine | 0.765 | 0.744 |
| carbachol | oxprenolol | 0.765 | 0.737 |
| mitoxantrone | ronidazole | 0.765 | 0.708 |
| fluvastatin | metergoline | 0.765 | 0.665 |
| cyproterone | promethazine | 0.765 | 0.748 |
| oxamniquine | roxithromycin | 0.765 | 0.835 |
| latamoxef | pargyline | 0.765 | 0.829 |
| isoconazole | metergoline | 0.765 | 0.744 |
| amiodarone | piperidolate | 0.765 | 0.710 |
| dipyridamole | flufenamic_acid | 0.765 | 0.740 |
| doxorubicin | flufenamic_acid | 0.765 | 0.711 |
| mephentermine | nitrofurantoin | 0.765 | 0.742 |
| diltiazem | methazolamide | 0.765 | 0.753 |
| clomipramine | fipexide | 0.765 | 0.790 |
| methylergometrine | tyloxapol | 0.765 | 0.842 |
| medrysone | milrinone | 0.765 | 0.734 |
| fluvoxamine | sulconazole | 0.765 | 0.747 |
| flupentixol | propofol | 0.765 | 0.787 |
| cefalotin | fluorometholone | 0.765 | 0.718 |
| indoprofen | simvastatin | 0.765 | 0.747 |
| diltiazem | trazodone | 0.765 | 0.755 |
| cycloserine | ramipril | 0.765 | 0.871 |
| budesonide | doxazosin | 0.765 | 0.743 |
| flufenamic_acid | sulfametoxydiazine | 0.765 | 0.677 |
| galantamine | piperidolate | 0.765 | 0.738 |
| benfotiamine | flecainide | 0.765 | 0.716 |
| dacarbazine | zimeldine | 0.765 | 0.746 |
| fluvoxamine | pyrvinium | 0.765 | 0.800 |
| norethisterone | torasemide | 0.765 | 0.741 |
| desipramine | etacrynic_acid | 0.765 | 0.709 |
| ketanserin | oxamic_acid | 0.765 | 0.855 |
| cefalexin | practolol | 0.765 | 0.716 |

| | | | |
|---|---|---|---|
| clindamycin | pentetrazol | 0.765 | 0.816 |
| doxazosin | etomidate | 0.765 | 0.751 |
| clozapine | perphenazine | 0.765 | 0.651 |
| citiolone | doxazosin | 0.765 | 0.819 |
| glibenclamide | metacycline | 0.765 | 0.747 |
| clomipramine | oxamic_acid | 0.765 | 0.894 |
| oxybutynin | oxytetracycline | 0.765 | 0.745 |
| deptropine | protriptyline | 0.765 | 0.663 |
| doxorubicin | glibenclamide | 0.765 | 0.726 |
| cefsulodin | metacycline | 0.765 | 0.735 |
| butoconazole | fluphenazine | 0.765 | 0.746 |
| clonidine | rimexolone | 0.765 | 0.795 |
| meptazinol | tribenoside | 0.765 | 0.802 |
| betaxolol | doxorubicin | 0.765 | 0.765 |
| pentamidine | terconazole | 0.765 | 0.746 |
| econazole | menadione | 0.765 | 0.751 |
| clenbuterol | clofazimine | 0.765 | 0.734 |
| phenazopyridine | triflusal | 0.765 | 0.714 |
| butoconazole | cisapride | 0.765 | 0.767 |
| acepromazine | milrinone | 0.765 | 0.659 |
| fenoprofen | procyclidine | 0.765 | 0.720 |
| cefalexin | monobenzone | 0.765 | 0.738 |
| cefalotin | hydrocortisone | 0.765 | 0.702 |
| bromopride | medrysone | 0.765 | 0.712 |
| ipratropium_bromide | verteporfin | 0.765 | 0.802 |
| clobetasol | phenazopyridine | 0.765 | 0.762 |
| clotrimazole | ticlopidine | 0.765 | 0.731 |
| clomipramine | pargyline | 0.765 | 0.670 |
| omeprazole | tropicamide | 0.765 | 0.693 |
| natamycin | tyloxapol | 0.765 | 0.800 |
| dipyridamole | sulfametoxydiazine | 0.765 | 0.768 |
| ipratropium_bromide | sulfametoxydiazine | 0.765 | 0.681 |
| benzylpenicillin | propofol | 0.765 | 0.778 |
| ethotoin | fenoprofen | 0.765 | 0.658 |
| acetylsalicylic_acid | proscillaridin | 0.765 | 0.832 |
| budesonide | phenindione | 0.765 | 0.773 |
| imipenem | milrinone | 0.765 | 0.684 |
| fenoprofen | liothyronine | 0.765 | 0.690 |
| paroxetine | pramocaine | 0.765 | 0.739 |
| proxymetacaine | sulconazole | 0.765 | 0.759 |
| cinchocaine | rimexolone | 0.765 | 0.765 |
| capsaicin | naloxone | 0.765 | 0.780 |
| propylthiouracil | ticlopidine | 0.765 | 0.740 |
| clozapine | fluphenazine | 0.765 | 0.651 |
| bupropion | clomipramine | 0.765 | 0.710 |
| mafenide | meptazinol | 0.765 | 0.742 |
| clofazimine | prenylamine | 0.765 | 0.692 |
| aciclovir | nadolol | 0.765 | 0.705 |
| piperidolate | promazine | 0.765 | 0.700 |
| amikacin | dexibuprofen | 0.765 | 0.845 |

| | | | |
|---|---|---|---|
| butoconazole | chlorprothixene | 0.765 | 0.712 |
| flumequine | gliclazide | 0.765 | 0.723 |
| artemisinin | imipenem | 0.765 | 0.742 |
| cefamandole | metaraminol | 0.765 | 0.787 |
| doxorubicin | ethotoin | 0.765 | 0.778 |
| demecolcine | pentoxyverine | 0.765 | 0.765 |
| bepridil | metyrapone | 0.765 | 0.713 |
| ethotoin | omeprazole | 0.765 | 0.700 |
| norethisterone | tobramycin | 0.765 | 0.781 |
| dobutamine | ketanserin | 0.765 | 0.666 |
| niflumic_acid | trazodone | 0.765 | 0.707 |
| bepridil | etamsylate | 0.765 | 0.816 |
| azacitidine | digoxin | 0.766 | 0.836 |
| calcium_pantothenate | tyloxapol | 0.766 | 0.843 |
| niclosamide | trihexyphenidyl | 0.766 | 0.733 |
| ketanserin | meptazinol | 0.766 | 0.784 |
| cyproterone | trioxysalen | 0.766 | 0.727 |
| levodopa | prednisone | 0.766 | 0.729 |
| dipivefrine | xylometazoline | 0.766 | 0.755 |
| clofazimine | zuclopenthixol | 0.766 | 0.706 |
| progesterone | propafenone | 0.766 | 0.760 |
| bezafibrate | bisoprolol | 0.766 | 0.684 |
| doxorubicin | rimexolone | 0.766 | 0.764 |
| reserpine | roxithromycin | 0.766 | 0.778 |
| chlorprothixene | mebendazole | 0.766 | 0.707 |
| deptropine | ethisterone | 0.766 | 0.759 |
| amiodarone | fenoprofen | 0.766 | 0.752 |
| cinchocaine | metacycline | 0.766 | 0.712 |
| cyclobenzaprine | metergoline | 0.766 | 0.740 |
| alfaxalone | ipratropium_bromide | 0.766 | 0.703 |
| aceclofenac | glafenine | 0.766 | 0.652 |
| indoprofen | trichlormethiazide | 0.766 | 0.667 |
| gefitinib | guanethidine | 0.766 | 0.771 |
| ketanserin | metyrapone | 0.766 | 0.727 |
| diltiazem | metyrapone | 0.766 | 0.700 |
| astemizole | deptropine | 0.766 | 0.764 |
| clioquinol | urapidil | 0.766 | 0.727 |
| gliclazide | phenoxybenzamine | 0.766 | 0.725 |
| dyclonine | triprolidine | 0.766 | 0.736 |
| mefloquine | vorinostat | 0.766 | 0.744 |
| omeprazole | trazodone | 0.766 | 0.655 |
| mafenide | phenazone | 0.766 | 0.710 |
| piperidolate | remoxipride | 0.766 | 0.652 |
| tolfenamic_acid | xylometazoline | 0.766 | 0.718 |
| metixene | nortriptyline | 0.766 | 0.662 |
| atovaquone | talampicillin | 0.766 | 0.703 |
| fenbendazole | pyrvinium | 0.766 | 0.730 |
| (-)-atenolol | liothyronine | 0.766 | 0.702 |
| haloperidol | mometasone | 0.766 | 0.725 |
| latamoxef | sulfamethoxypyridazir | 0.766 | 0.729 |

| | | | |
|---|---|---|---|
| aminophylline | nizatidine | 0.766 | 0.785 |
| mifepristone | trifluoperazine | 0.766 | 0.711 |
| fenoprofen | labetalol | 0.766 | 0.708 |
| bromopride | cefalotin | 0.766 | 0.714 |
| gliclazide | metacycline | 0.766 | 0.713 |
| carbachol | trioxysalen | 0.766 | 0.651 |
| clonidine | etacrynic_acid | 0.766 | 0.677 |
| domperidone | ketoconazole | 0.766 | 0.732 |
| butoconazole | ciclopirox | 0.766 | 0.719 |
| pyrvinium | quinisocaine | 0.766 | 0.774 |
| fluspirilene | promethazine | 0.766 | 0.767 |
| cetirizine | nifuroxazide | 0.766 | 0.743 |
| flunarizine | triprolidine | 0.766 | 0.715 |
| betaxolol | metyrapone | 0.766 | 0.733 |
| clofazimine | metixene | 0.766 | 0.729 |
| bromperidol | buflomedil | 0.766 | 0.693 |
| promazine | syrosingopine | 0.766 | 0.796 |
| fenoterol | propafenone | 0.766 | 0.705 |
| pentoxyverine | procaine | 0.766 | 0.707 |
| deptropine | meptazinol | 0.766 | 0.747 |
| cloxacillin | pentoxifylline | 0.766 | 0.667 |
| bisacodyl | phenoxybenzamine | 0.766 | 0.741 |
| aminohippuric_acid | etofenamate | 0.766 | 0.658 |
| buflomedil | nilutamide | 0.766 | 0.693 |
| clomifene | syrosingopine | 0.766 | 0.780 |
| pimozide | protriptyline | 0.766 | 0.767 |
| cefixime | dacarbazine | 0.766 | 0.720 |
| betahistine | bromperidol | 0.766 | 0.756 |
| mianserin | thioproperazine | 0.766 | 0.760 |
| mexiletine | phentolamine | 0.766 | 0.703 |
| cyclizine | gliclazide | 0.766 | 0.752 |
| econazole | rescinnamine | 0.766 | 0.789 |
| physostigmine | sulfametoxydiazine | 0.766 | 0.718 |
| deptropine | remoxipride | 0.766 | 0.714 |
| etoposide | niclosamide | 0.766 | 0.761 |
| bromopride | oxantel | 0.766 | 0.674 |
| letrozole | simvastatin | 0.766 | 0.741 |
| dosulepin | mefloquine | 0.766 | 0.719 |
| bromperidol | cetirizine | 0.766 | 0.709 |
| haloperidol | levomepromazine | 0.766 | 0.760 |
| terconazole | tiabendazole | 0.766 | 0.782 |
| tropicamide | zimeldine | 0.766 | 0.686 |
| aminohippuric_acid | rimexolone | 0.766 | 0.759 |
| meptazinol | omeprazole | 0.766 | 0.765 |
| bisacodyl | triflusal | 0.766 | 0.784 |
| cyproterone | etamsylate | 0.766 | 0.800 |
| bepridil | cefalotin | 0.766 | 0.773 |
| meticrane | sulfamerazine | 0.766 | 0.690 |
| alprostadil | dihydroergocristine | 0.766 | 0.747 |
| flufenamic_acid | piroxicam | 0.766 | 0.674 |

| | | | |
|---|---|---|---|
| maprotiline | penbutolol | 0.766 | 0.658 |
| ronidazole | verteporfin | 0.766 | 0.836 |
| ambroxol | nafcillin | 0.766 | 0.655 |
| acetazolamide | imipramine | 0.766 | 0.785 |
| cetirizine | natamycin | 0.766 | 0.805 |
| capsaicin | piperidolate | 0.766 | 0.733 |
| bromocriptine | propafenone | 0.766 | 0.731 |
| carbachol | quinisocaine | 0.766 | 0.767 |
| bromopride | rimexolone | 0.766 | 0.747 |
| metamizole_sodium | sulfametoxydiazine | 0.766 | 0.679 |
| bisacodyl | sulfamethoxypyridazir | 0.766 | 0.705 |
| ethotoin | quinethazone | 0.766 | 0.669 |
| fluspirilene | metixene | 0.766 | 0.758 |
| desoxycortone | molindone | 0.766 | 0.718 |
| (-)-catechin | trioxysalen | 0.766 | 0.682 |
| capsaicin | pyrazinamide | 0.766 | 0.745 |
| calcium_pantothenate | piperidolate | 0.766 | 0.797 |
| talampicillin | ursodeoxycholic_acid | 0.766 | 0.727 |
| nitrofurantoin | pivampicillin | 0.766 | 0.767 |
| oxetacaine | terfenadine | 0.766 | 0.697 |
| dosulepin | prenylamine | 0.766 | 0.696 |
| pergolide | trifluoperazine | 0.766 | 0.681 |
| diazoxide | triamcinolone | 0.766 | 0.766 |
| etamsylate | sulpiride | 0.766 | 0.690 |
| acetylsalicylic_acid | rimexolone | 0.766 | 0.766 |
| clindamycin | flufenamic_acid | 0.766 | 0.744 |
| labetalol | trifluoperazine | 0.766 | 0.712 |
| levamisole | sulfamethoxypyridazir | 0.766 | 0.697 |
| imipenem | proxyphylline | 0.766 | 0.653 |
| etodolac | glafenine | 0.766 | 0.682 |
| etamsylate | ketoprofen | 0.766 | 0.702 |
| doxepin | dyclonine | 0.766 | 0.743 |
| meticrane | oxprenolol | 0.766 | 0.693 |
| dipivefrine | practolol | 0.766 | 0.730 |
| econazole | pimozide | 0.766 | 0.763 |
| cisapride | sulconazole | 0.766 | 0.771 |
| methazolamide | papaverine | 0.766 | 0.705 |
| meropenem | triflusal | 0.766 | 0.761 |
| hyoscyamine | pargyline | 0.766 | 0.727 |
| homatropine | loracarbef | 0.766 | 0.675 |
| dexibuprofen | primidone | 0.766 | 0.693 |
| flufenamic_acid | naloxone | 0.766 | 0.703 |
| etofylline | pargyline | 0.766 | 0.723 |
| oxaprozin | triflusal | 0.767 | 0.714 |
| benperidol | sulconazole | 0.767 | 0.738 |
| dicoumarol | meticrane | 0.767 | 0.718 |
| mefenamic_acid | sulfadiazine | 0.767 | 0.678 |
| meclozine | promethazine | 0.767 | 0.708 |
| deferoxamine | promazine | 0.767 | 0.847 |
| piromidic_acid | rimexolone | 0.767 | 0.732 |

| | | | |
|---|---|---|---|
| liothyronine | meclozine | 0.767 | 0.754 |
| ciclosporin | suloctidil | 0.767 | 0.872 |
| pargyline | ranitidine | 0.767 | 0.768 |
| griseofulvin | roxithromycin | 0.767 | 0.833 |
| budesonide | oxamic_acid | 0.767 | 0.872 |
| dipivefrine | emetine | 0.767 | 0.746 |
| desoxycortone | gliclazide | 0.767 | 0.768 |
| fenoprofen | xylometazoline | 0.767 | 0.707 |
| clotrimazole | sertaconazole | 0.767 | 0.666 |
| dipivefrine | sulfadimidine | 0.767 | 0.730 |
| cetirizine | milrinone | 0.767 | 0.779 |
| raloxifene | suloctidil | 0.767 | 0.748 |
| betaxolol | verteporfin | 0.767 | 0.818 |
| lanatoside_C | vorinostat | 0.767 | 0.856 |
| medrysone | procaine | 0.767 | 0.688 |
| meticrane | pivampicillin | 0.767 | 0.754 |
| spectinomycin | theobromine | 0.767 | 0.771 |
| tranylcypromine | zimeldine | 0.767 | 0.689 |
| doxylamine | methylergometrine | 0.767 | 0.722 |
| meclofenoxate | menadione | 0.767 | 0.709 |
| oxetacaine | rescinnamine | 0.767 | 0.712 |
| liothyronine | paracetamol | 0.767 | 0.752 |
| ketorolac | lymecycline | 0.767 | 0.777 |
| etoposide | pyrvinium | 0.767 | 0.795 |
| nortriptyline | syrosingopine | 0.767 | 0.818 |
| propylthiouracil | triflusal | 0.767 | 0.654 |
| oxybutynin | rimexolone | 0.767 | 0.731 |
| flunarizine | nitrofurantoin | 0.767 | 0.786 |
| ciclosporin | metixene | 0.767 | 0.892 |
| procyclidine | sulconazole | 0.767 | 0.709 |
| butoconazole | perphenazine | 0.767 | 0.741 |
| aminohippuric_acid | dextromethorphan | 0.767 | 0.737 |
| bepridil | flupentixol | 0.767 | 0.735 |
| menadione | sulfamethoxypyridazir | 0.767 | 0.722 |
| betahistine | ketanserin | 0.767 | 0.742 |
| exemestane | idoxuridine | 0.767 | 0.699 |
| cefalotin | sulfamethoxypyridazir | 0.767 | 0.710 |
| colchicine | etacrynic_acid | 0.767 | 0.705 |
| ethambutol | suprofen | 0.767 | 0.682 |
| semustine | trifluridine | 0.767 | 0.702 |
| metamizole_sodium | monobenzone | 0.767 | 0.656 |
| mepyramine | repaglinide | 0.767 | 0.752 |
| cetirizine | methazolamide | 0.767 | 0.775 |
| azlocillin | gabapentin | 0.767 | 0.797 |
| butoconazole | meclozine | 0.767 | 0.720 |
| azacitidine | etidronic_acid | 0.767 | 0.713 |
| fluvastatin | hydrocortisone | 0.767 | 0.721 |
| molindone | repaglinide | 0.767 | 0.740 |
| clotrimazole | fipexide | 0.767 | 0.767 |
| metaraminol | nitrofurantoin | 0.767 | 0.734 |

| | | | |
|---|---|---|---|
| metacycline | ursodeoxycholic_acid | 0.767 | 0.727 |
| gliclazide | labetalol | 0.767 | 0.668 |
| lymecycline | trazodone | 0.767 | 0.762 |
| meticrane | promazine | 0.767 | 0.742 |
| phenazopyridine | pyrvinium | 0.767 | 0.775 |
| fluvoxamine | memantine | 0.767 | 0.787 |
| liothyronine | talampicillin | 0.767 | 0.724 |
| fenoprofen | pentoxyverine | 0.767 | 0.733 |
| deptropine | zimeldine | 0.767 | 0.722 |
| azacitidine | syrosingopine | 0.767 | 0.814 |
| daunorubicin | deptropine | 0.767 | 0.743 |
| betaxolol | piperidolate | 0.767 | 0.727 |
| omeprazole | rolitetracycline | 0.767 | 0.723 |
| ronidazole | sulfafurazole | 0.767 | 0.676 |
| isotretinoin | lomustine | 0.767 | 0.777 |
| bromopride | hydrocortisone | 0.767 | 0.760 |
| phenoxybenzamine | quinisocaine | 0.767 | 0.693 |
| clobetasol | naloxone | 0.767 | 0.738 |
| folic_acid | racecadotril | 0.767 | 0.736 |
| fenbendazole | perphenazine | 0.767 | 0.682 |
| fenoprofen | trazodone | 0.767 | 0.728 |
| biotin | flucytosine | 0.767 | 0.735 |
| raloxifene | rifabutin | 0.767 | 0.779 |
| clomipramine | hydrocortisone | 0.767 | 0.794 |
| promazine | proscillaridin | 0.767 | 0.823 |
| bepridil | quinisocaine | 0.767 | 0.700 |
| daunorubicin | sulfamerazine | 0.767 | 0.741 |
| bromperidol | ronidazole | 0.767 | 0.745 |
| amiodarone | liothyronine | 0.767 | 0.718 |
| ciclosporin | fluspirilene | 0.767 | 0.869 |
| omeprazole | procainamide | 0.767 | 0.661 |
| etidronic_acid | sulconazole | 0.767 | 0.836 |
| labetalol | terfenadine | 0.767 | 0.723 |
| piribedil | proxymetacaine | 0.767 | 0.673 |
| bepridil | ciclopirox | 0.767 | 0.767 |
| bromperidol | spectinomycin | 0.767 | 0.771 |
| sulfametoxydiazine | zomepirac | 0.767 | 0.665 |
| noretynodrel | perphenazine | 0.767 | 0.749 |
| dacarbazine | promethazine | 0.767 | 0.732 |
| methylprednisolone | pimozide | 0.767 | 0.759 |
| etacrynic_acid | mifepristone | 0.767 | 0.738 |
| cefalotin | omeprazole | 0.767 | 0.683 |
| phenazopyridine | protriptyline | 0.767 | 0.747 |
| ioversol | nicotinic_acid | 0.767 | 0.853 |
| levonorgestrel | triflusal | 0.767 | 0.787 |
| dacarbazine | pargyline | 0.767 | 0.702 |
| metamizole_sodium | triflusal | 0.767 | 0.716 |
| clomifene | protriptyline | 0.767 | 0.696 |
| tolazoline | tolnaftate | 0.767 | 0.685 |
| chlorphenamine | nomifensine | 0.767 | 0.652 |

| | | | |
|---|---|---|---|
| bepridil | chlorcyclizine | 0.767 | 0.652 |
| miconazole | thioridazine | 0.767 | 0.701 |
| acepromazine | fluspirilene | 0.767 | 0.752 |
| amiloride | dicycloverine | 0.767 | 0.769 |
| mitoxantrone | pentoxyverine | 0.767 | 0.785 |
| suloctidil | ticlopidine | 0.767 | 0.756 |
| meticrane | mitoxantrone | 0.767 | 0.747 |
| cycloserine | zimeldine | 0.767 | 0.868 |
| nabumetone | sulconazole | 0.768 | 0.689 |
| ketanserin | mebendazole | 0.768 | 0.664 |
| hydralazine | sulconazole | 0.768 | 0.784 |
| ketanserin | remoxipride | 0.768 | 0.704 |
| daunorubicin | etoposide | 0.768 | 0.692 |
| felodipine | fendiline | 0.768 | 0.735 |
| molindone | talampicillin | 0.768 | 0.756 |
| etomidate | risperidone | 0.768 | 0.740 |
| alfuzosin | trifluoperazine | 0.768 | 0.672 |
| (-)-isoprenaline | methylergometrine | 0.768 | 0.728 |
| pentamidine | piromidic_acid | 0.768 | 0.701 |
| ipratropium_bromide | pargyline | 0.768 | 0.736 |
| desipramine | phenoxybenzamine | 0.768 | 0.708 |
| oxamniquine | promethazine | 0.768 | 0.744 |
| cefalotin | levamisole | 0.768 | 0.741 |
| doxorubicin | meclozine | 0.768 | 0.779 |
| piperidolate | tetryzoline | 0.768 | 0.661 |
| amiodarone | naloxone | 0.768 | 0.759 |
| acepromazine | syrosingopine | 0.768 | 0.798 |
| ketanserin | sulfafurazole | 0.768 | 0.705 |
| bisacodyl | felodipine | 0.768 | 0.720 |
| dipyridamole | ronidazole | 0.768 | 0.742 |
| estriol | molindone | 0.768 | 0.666 |
| (-)-isoprenaline | bromocriptine | 0.768 | 0.796 |
| metyrapone | molindone | 0.768 | 0.664 |
| azacitidine | levonorgestrel | 0.768 | 0.767 |
| galantamine | sulconazole | 0.768 | 0.751 |
| tobramycin | trazodone | 0.768 | 0.774 |
| droperidol | ritodrine | 0.768 | 0.668 |
| clonidine | triflusal | 0.768 | 0.722 |
| meptazinol | minaprine | 0.768 | 0.745 |
| bisacodyl | butoconazole | 0.768 | 0.749 |
| cinchocaine | miconazole | 0.768 | 0.758 |
| oxybuprocaine | tyloxapol | 0.768 | 0.860 |
| clonidine | fenoprofen | 0.768 | 0.688 |
| flufenamic_acid | oxamic_acid | 0.768 | 0.803 |
| bisacodyl | gliclazide | 0.768 | 0.743 |
| buflomedil | meclofenoxate | 0.768 | 0.651 |
| nitrofurantoin | triflusal | 0.768 | 0.712 |
| (-)-catechin | oxetacaine | 0.768 | 0.782 |
| cefalexin | roxithromycin | 0.768 | 0.801 |
| chloramphenicol | talampicillin | 0.768 | 0.746 |

| | | | |
|---|---|---|---|
| terfenadine | trazodone | 0.768 | 0.698 |
| clomipramine | digoxin | 0.768 | 0.869 |
| bromocriptine | tribenoside | 0.768 | 0.729 |
| etomidate | propylthiouracil | 0.768 | 0.658 |
| benperidol | piribedil | 0.768 | 0.686 |
| diltiazem | pargyline | 0.768 | 0.772 |
| daunorubicin | nitrofurantoin | 0.768 | 0.728 |
| demecolcine | loperamide | 0.768 | 0.761 |
| progesterone | trioxysalen | 0.768 | 0.724 |
| cyproterone | piromidic_acid | 0.768 | 0.726 |
| benzylpenicillin | urapidil | 0.768 | 0.735 |
| etofenamate | metyrapone | 0.768 | 0.693 |
| cyproheptadine | fenbendazole | 0.768 | 0.751 |
| doxorubicin | lymecycline | 0.768 | 0.683 |
| apomorphine | rimexolone | 0.768 | 0.752 |
| levamisole | rimexolone | 0.768 | 0.764 |
| dopamine | iloprost | 0.768 | 0.804 |
| cimetidine | clindamycin | 0.768 | 0.734 |
| bendroflumethiazide | flunisolide | 0.768 | 0.706 |
| dipyridamole | testosterone | 0.768 | 0.770 |
| protriptyline | vorinostat | 0.768 | 0.777 |
| doxylamine | enoxacin | 0.768 | 0.758 |
| cefalexin | testosterone | 0.768 | 0.739 |
| cefalotin | ronidazole | 0.768 | 0.721 |
| doxazosin | etidronic_acid | 0.768 | 0.829 |
| etidronic_acid | talampicillin | 0.768 | 0.819 |
| (-)-atenolol | carbachol | 0.768 | 0.751 |
| sirolimus | terfenadine | 0.768 | 0.823 |
| bisoprolol | droperidol | 0.768 | 0.709 |
| cefotaxime | decitabine | 0.768 | 0.713 |
| moxonidine | sotalol | 0.768 | 0.692 |
| beclometasone | verteporfin | 0.768 | 0.773 |
| mesoridazine | sulconazole | 0.768 | 0.716 |
| carbachol | menadione | 0.768 | 0.712 |
| mitoxantrone | rolitetracycline | 0.768 | 0.767 |
| protriptyline | talampicillin | 0.768 | 0.786 |
| glibenclamide | talampicillin | 0.768 | 0.717 |
| clomifene | flupentixol | 0.768 | 0.732 |
| cyproterone | hydralazine | 0.768 | 0.779 |
| flufenamic_acid | ronidazole | 0.768 | 0.677 |
| hydroquinine | metixene | 0.768 | 0.684 |
| ambroxol | iopamidol | 0.768 | 0.757 |
| doxazosin | ifenprodil | 0.768 | 0.725 |
| etidronic_acid | trimipramine | 0.768 | 0.839 |
| acetylsalicylic_acid | flunisolide | 0.768 | 0.761 |
| etomidate | gliclazide | 0.768 | 0.697 |
| isradipine | verteporfin | 0.768 | 0.818 |
| ethambutol | galantamine | 0.768 | 0.733 |
| menadione | pimethixene | 0.768 | 0.680 |
| etamsylate | levamisole | 0.768 | 0.701 |

| | | | |
|---|---|---|---|
| azacitidine | promazine | 0.768 | 0.744 |
| deptropine | piperidolate | 0.768 | 0.675 |
| bepridil | chlorzoxazone | 0.768 | 0.795 |
| ketanserin | roxithromycin | 0.768 | 0.813 |
| milrinone | remoxipride | 0.768 | 0.750 |
| (-)-catechin | imipenem | 0.768 | 0.691 |
| meticrane | vidarabine | 0.768 | 0.676 |
| azacitidine | roxithromycin | 0.768 | 0.847 |
| benfluorex | tetryzoline | 0.768 | 0.758 |
| fenoterol | tolfenamic_acid | 0.768 | 0.699 |
| haloperidol | suloctidil | 0.768 | 0.685 |
| fipexide | glibenclamide | 0.768 | 0.711 |
| menadione | miconazole | 0.768 | 0.764 |
| amitriptyline | ivermectin | 0.768 | 0.877 |
| disulfiram | naltrexone | 0.768 | 0.722 |
| procyclidine | trimipramine | 0.768 | 0.685 |
| deptropine | piribedil | 0.768 | 0.766 |
| cyclobenzaprine | fenbendazole | 0.768 | 0.726 |
| repaglinide | vinpocetine | 0.768 | 0.748 |
| etidronic_acid | rimexolone | 0.768 | 0.824 |
| budesonide | carbachol | 0.768 | 0.808 |
| omeprazole | pargyline | 0.768 | 0.746 |
| calcium_pantothenate | triflusal | 0.768 | 0.810 |
| bisacodyl | irinotecan | 0.768 | 0.778 |
| clomipramine | latamoxef | 0.768 | 0.819 |
| dihydroergotamine | disulfiram | 0.768 | 0.779 |
| glibenclamide | mesalazine | 0.768 | 0.760 |
| paroxetine | phenelzine | 0.768 | 0.754 |
| monobenzone | piribedil | 0.768 | 0.702 |
| apomorphine | naloxone | 0.768 | 0.753 |
| dipivefrine | etilefrine | 0.768 | 0.686 |
| clozapine | metergoline | 0.768 | 0.721 |
| phenoxybenzamine | propofol | 0.768 | 0.730 |
| lomefloxacin | propylthiouracil | 0.768 | 0.658 |
| griseofulvin | liothyronine | 0.768 | 0.715 |
| alimemazine | labetalol | 0.768 | 0.755 |
| imipramine | pyrvinium | 0.768 | 0.785 |
| oxetacaine | pimozide | 0.768 | 0.712 |
| cinchocaine | desoxycortone | 0.768 | 0.769 |
| gefitinib | tetracycline | 0.768 | 0.739 |
| betaxolol | milrinone | 0.769 | 0.756 |
| apomorphine | clomipramine | 0.769 | 0.690 |
| ciclosporin | dienestrol | 0.769 | 0.897 |
| hydralazine | roxithromycin | 0.769 | 0.889 |
| doxorubicin | ganciclovir | 0.769 | 0.670 |
| doxazosin | procyclidine | 0.769 | 0.781 |
| bemegride | sulconazole | 0.769 | 0.784 |
| cefoxitin | ribostamycin | 0.769 | 0.738 |
| chloramphenicol | triflusal | 0.769 | 0.680 |
| metergoline | quinisocaine | 0.769 | 0.710 |

| | | | |
|---|---|---|---|
| mianserin | nalbuphine | 0.769 | 0.782 |
| menadione | metixene | 0.769 | 0.750 |
| flumetasone | sulfamethoxypyridazir | 0.769 | 0.737 |
| metamizole_sodium | sulfamethoxazole | 0.769 | 0.690 |
| benzethonium_chloride | thioproperazine | 0.769 | 0.775 |
| ketanserin | latamoxef | 0.769 | 0.712 |
| mycophenolic_acid | ribavirin | 0.769 | 0.685 |
| fluspirilene | sulfamethoxypyridazir | 0.769 | 0.706 |
| imipenem | medrysone | 0.769 | 0.729 |
| ciclopirox | metergoline | 0.769 | 0.761 |
| etacrynic_acid | sirolimus | 0.769 | 0.858 |
| cefamandole | mephenesin | 0.769 | 0.762 |
| meclofenoxate | omeprazole | 0.769 | 0.653 |
| ivermectin | ticlopidine | 0.769 | 0.876 |
| oxybuprocaine | roxithromycin | 0.769 | 0.835 |
| disulfiram | pimozide | 0.769 | 0.773 |
| cefotiam | nitrofural | 0.769 | 0.764 |
| nitrofurantoin | tropicamide | 0.769 | 0.723 |
| oxymetazoline | tranylcypromine | 0.769 | 0.731 |
| propylthiouracil | talampicillin | 0.769 | 0.789 |
| gliclazide | propofol | 0.769 | 0.795 |
| methazolamide | tyloxapol | 0.769 | 0.887 |
| biotin | oxantel | 0.769 | 0.731 |
| benzocaine | biperiden | 0.769 | 0.701 |
| astemizole | promazine | 0.769 | 0.734 |
| dihydroergotamine | loperamide | 0.769 | 0.686 |
| oxedrine | verteporfin | 0.769 | 0.856 |
| fipexide | repaglinide | 0.769 | 0.738 |
| estriol | sulfamethoxypyridazir | 0.769 | 0.717 |
| flavoxate | trazodone | 0.769 | 0.694 |
| bumetanide | dropropizine | 0.769 | 0.716 |
| ivermectin | mifepristone | 0.769 | 0.837 |
| metamizole_sodium | trioxysalen | 0.769 | 0.671 |
| probenecid | trichlormethiazide | 0.769 | 0.669 |
| diltiazem | ifenprodil | 0.769 | 0.755 |
| bezafibrate | talampicillin | 0.769 | 0.686 |
| betaxolol | bromperidol | 0.769 | 0.711 |
| colchicine | proscillaridin | 0.769 | 0.800 |
| betahistine | meclozine | 0.769 | 0.726 |
| cefotiam | fluoxetine | 0.769 | 0.770 |
| etofenamate | proscillaridin | 0.769 | 0.763 |
| demecolcine | piribedil | 0.769 | 0.730 |
| etidronic_acid | verteporfin | 0.769 | 0.875 |
| fluphenazine | miconazole | 0.769 | 0.745 |
| methapyrilene | pentoxyverine | 0.769 | 0.703 |
| amodiaquine | guanethidine | 0.769 | 0.740 |
| (-)-catechin | perphenazine | 0.769 | 0.739 |
| oxetacaine | vorinostat | 0.769 | 0.721 |
| meclofenoxate | verteporfin | 0.769 | 0.803 |
| pentamidine | pyrazinamide | 0.769 | 0.722 |

| | | | |
|---|---|---|---|
| bromopride | estriol | 0.769 | 0.700 |
| prochlorperazine | terconazole | 0.769 | 0.750 |
| doxorubicin | moxonidine | 0.769 | 0.725 |
| lymecycline | repaglinide | 0.769 | 0.777 |
| rolitetracycline | roxithromycin | 0.769 | 0.770 |
| dextromethorphan | omeprazole | 0.769 | 0.762 |
| acetylsalicylic_acid | urapidil | 0.769 | 0.766 |
| astemizole | levonorgestrel | 0.769 | 0.772 |
| flufenamic_acid | torasemide | 0.769 | 0.660 |
| cefixime | pentoxyverine | 0.769 | 0.747 |
| griseofulvin | sulconazole | 0.769 | 0.735 |
| ifenprodil | pentoxyverine | 0.769 | 0.726 |
| promazine | talampicillin | 0.769 | 0.783 |
| letrozole | metaraminol | 0.769 | 0.768 |
| carbachol | fluvastatin | 0.769 | 0.805 |
| prenylamine | rescinnamine | 0.769 | 0.783 |
| nystatin | zuclopenthixol | 0.769 | 0.846 |
| roxithromycin | sulfapyridine | 0.769 | 0.857 |
| dydrogesterone | galantamine | 0.769 | 0.746 |
| meglumine | nicotinic_acid | 0.769 | 0.730 |
| amoxicillin | bepridil | 0.769 | 0.784 |
| metergoline | procarbazine | 0.769 | 0.736 |
| ciclosporin | levomepromazine | 0.769 | 0.886 |
| diflunisal | doxylamine | 0.769 | 0.754 |
| chlorcyclizine | isosorbide | 0.769 | 0.773 |
| pivampicillin | tyloxapol | 0.769 | 0.836 |
| ethotoin | oxamniquine | 0.769 | 0.728 |
| fenbendazole | maprotiline | 0.769 | 0.732 |
| azacitidine | bisacodyl | 0.769 | 0.763 |
| cefalexin | piribedil | 0.769 | 0.663 |
| gefitinib | thiocolchicoside | 0.769 | 0.723 |
| promethazine | triflusal | 0.769 | 0.744 |
| labetalol | sulconazole | 0.769 | 0.746 |
| aminohippuric_acid | medrysone | 0.769 | 0.735 |
| hyoscyamine | triflusal | 0.769 | 0.727 |
| diltiazem | pyrvinium | 0.769 | 0.793 |
| fluspirilene | thioproperazine | 0.769 | 0.745 |
| fluphenazine | rifabutin | 0.769 | 0.760 |
| menadione | sulfaphenazole | 0.769 | 0.683 |
| fenbufen | tropicamide | 0.769 | 0.712 |
| demecolcine | vinblastine | 0.769 | 0.812 |
| demecolcine | deptropine | 0.769 | 0.749 |
| proxyphylline | repaglinide | 0.769 | 0.725 |
| isocarboxazid | levomepromazine | 0.769 | 0.730 |
| benzonatate | dexpanthenol | 0.769 | 0.854 |
| ciclopirox | loperamide | 0.769 | 0.794 |
| isradipine | proxymetacaine | 0.769 | 0.748 |
| deptropine | ethotoin | 0.769 | 0.742 |
| etoposide | proscillaridin | 0.769 | 0.754 |
| chlortetracycline | midecamycin | 0.769 | 0.789 |

| | | | |
|---|---|---|---|
| acetylsalicylic_acid | dipyridamole | 0.769 | 0.803 |
| acepromazine | labetalol | 0.769 | 0.720 |
| emetine | fluspirilene | 0.769 | 0.715 |
| clotrimazole | ketoconazole | 0.769 | 0.754 |
| cycloserine | doxorubicin | 0.769 | 0.879 |
| cinchocaine | estriol | 0.769 | 0.764 |
| acepromazine | phenoxybenzamine | 0.769 | 0.743 |
| cefalotin | cycloserine | 0.769 | 0.857 |
| alfuzosin | betaxolol | 0.769 | 0.714 |
| benzonatate | betazole | 0.769 | 0.854 |
| liothyronine | oxaprozin | 0.770 | 0.674 |
| flunarizine | propafenone | 0.770 | 0.754 |
| aminohippuric_acid | cinchocaine | 0.770 | 0.684 |
| meptazinol | piribedil | 0.770 | 0.746 |
| norethisterone | tyloxapol | 0.770 | 0.874 |
| propafenone | tropicamide | 0.770 | 0.723 |
| mecamylamine | moxisylyte | 0.770 | 0.795 |
| diltiazem | mefloquine | 0.770 | 0.730 |
| flucytosine | progesterone | 0.770 | 0.851 |
| scopolamine | sulfamerazine | 0.770 | 0.686 |
| ganciclovir | nitrendipine | 0.770 | 0.712 |
| ganciclovir | paracetamol | 0.770 | 0.661 |
| bromocriptine | tyloxapol | 0.770 | 0.816 |
| propylthiouracil | tiaprofenic_acid | 0.770 | 0.681 |
| amiodarone | triflusal | 0.770 | 0.803 |
| fluorometholone | verteporfin | 0.770 | 0.784 |
| ipratropium_bromide | propafenone | 0.770 | 0.701 |
| betahistine | fenoprofen | 0.770 | 0.694 |
| amiodarone | dacarbazine | 0.770 | 0.774 |
| cinchocaine | propafenone | 0.770 | 0.671 |
| metacycline | phenoxybenzamine | 0.770 | 0.719 |
| (-)-catechin | dacarbazine | 0.770 | 0.652 |
| monobenzone | trazodone | 0.770 | 0.753 |
| propafenone | tolfenamic_acid | 0.770 | 0.664 |
| econazole | zuclopenthixol | 0.770 | 0.730 |
| levonorgestrel | tyloxapol | 0.770 | 0.873 |
| cyanocobalamin | flupentixol | 0.770 | 0.849 |
| bromocriptine | metergoline | 0.770 | 0.683 |
| fenoterol | nabumetone | 0.770 | 0.669 |
| bepridil | emetine | 0.770 | 0.748 |
| labetalol | latamoxef | 0.770 | 0.725 |
| oxamniquine | oxybuprocaine | 0.770 | 0.711 |
| mitoxantrone | sulfametoxydiazine | 0.770 | 0.745 |
| clindamycin | nitrendipine | 0.770 | 0.738 |
| chlorcyclizine | isoconazole | 0.770 | 0.684 |
| cefalotin | clomipramine | 0.770 | 0.787 |
| mometasone | quinisocaine | 0.770 | 0.765 |
| cefalexin | trimetazidine | 0.770 | 0.729 |
| famotidine | oleandomycin | 0.770 | 0.815 |
| maprotiline | thioridazine | 0.770 | 0.697 |

| | | | |
|---|---|---|---|
| bezafibrate | tyloxapol | 0.770 | 0.853 |
| benzethonium_chloride | haloperidol | 0.770 | 0.711 |
| flupentixol | rescinnamine | 0.770 | 0.738 |
| bisacodyl | niridazole | 0.770 | 0.769 |
| azacitidine | proscillaridin | 0.770 | 0.805 |
| octopamine | tobramycin | 0.770 | 0.814 |
| ketoprofen | triflusal | 0.770 | 0.700 |
| meclozine | piribedil | 0.770 | 0.695 |
| bromopride | tyloxapol | 0.770 | 0.865 |
| pentoxyverine | phenoxybenzamine | 0.770 | 0.734 |
| mefloquine | meptazinol | 0.770 | 0.727 |
| promethazine | terfenadine | 0.770 | 0.784 |
| carbachol | flunisolide | 0.770 | 0.812 |
| disopyramide | glafenine | 0.770 | 0.750 |
| gliclazide | phenindione | 0.770 | 0.713 |
| emetine | etacrynic_acid | 0.770 | 0.764 |
| alimemazine | terfenadine | 0.770 | 0.770 |
| metixene | pyrvinium | 0.770 | 0.782 |
| latamoxef | practolol | 0.770 | 0.716 |
| colecalciferol | methoxsalen | 0.770 | 0.738 |
| meglumine | urapidil | 0.770 | 0.796 |
| reserpine | trioxysalen | 0.770 | 0.818 |
| betahistine | theobromine | 0.770 | 0.676 |
| astemizole | dienestrol | 0.770 | 0.735 |
| flufenamic_acid | ursodeoxycholic_acid | 0.770 | 0.725 |
| labetalol | metergoline | 0.770 | 0.720 |
| alprenolol | dipivefrine | 0.770 | 0.723 |
| tolmetin | trimipramine | 0.770 | 0.739 |
| (-)-catechin | propafenone | 0.770 | 0.710 |
| flunisolide | spiramycin | 0.770 | 0.809 |
| benzylpenicillin | zomepirac | 0.770 | 0.674 |
| omeprazole | selegiline | 0.770 | 0.735 |
| suloctidil | syrosingopine | 0.770 | 0.766 |
| clotrimazole | verteporfin | 0.770 | 0.832 |
| fenoterol | meticrane | 0.770 | 0.743 |
| hydrocortisone | talampicillin | 0.770 | 0.696 |
| dipyridamole | fenoterol | 0.770 | 0.772 |
| reserpine | terfenadine | 0.770 | 0.737 |
| promethazine | verteporfin | 0.770 | 0.802 |
| acepromazine | fenoprofen | 0.770 | 0.700 |
| gentamicin | midodrine | 0.770 | 0.757 |
| papaverine | talampicillin | 0.770 | 0.761 |
| phenazone | sulfathiazole | 0.770 | 0.691 |
| acepromazine | meticrane | 0.770 | 0.711 |
| hyoscyamine | talampicillin | 0.770 | 0.709 |
| ronidazole | trimetazidine | 0.770 | 0.662 |
| decitabine | mycophenolic_acid | 0.770 | 0.702 |
| fluocinonide | roxithromycin | 0.770 | 0.771 |
| guanethidine | sulfinpyrazone | 0.770 | 0.765 |
| protriptyline | rescinnamine | 0.770 | 0.797 |

| | | | |
|---|---|---|---|
| piperidolate | sulfaphenazole | 0.770 | 0.702 |
| oxetacaine | reserpine | 0.770 | 0.715 |
| diltiazem | ipratropium_bromide | 0.770 | 0.719 |
| lovastatin | methoxamine | 0.770 | 0.774 |
| azacitidine | sulfaphenazole | 0.770 | 0.677 |
| sulfamethoxazole | tropicamide | 0.770 | 0.683 |
| ronidazole | xylometazoline | 0.770 | 0.703 |
| clobetasol | promethazine | 0.770 | 0.782 |
| dihydroergotamine | mefloquine | 0.770 | 0.746 |
| doxorubicin | latamoxef | 0.770 | 0.715 |
| methylergometrine | pimozide | 0.770 | 0.735 |
| acepromazine | benzylpenicillin | 0.770 | 0.748 |
| mesalazine | trazodone | 0.770 | 0.710 |
| sulfamerazine | tropicamide | 0.770 | 0.673 |
| calcium_pantothenate | etofenamate | 0.770 | 0.775 |
| benperidol | doxazosin | 0.770 | 0.672 |
| pyrvinium | tyloxapol | 0.770 | 0.864 |
| pargyline | repaglinide | 0.770 | 0.795 |
| dipivefrine | suloctidil | 0.770 | 0.732 |
| bromopride | sulconazole | 0.770 | 0.746 |
| ethotoin | tyloxapol | 0.770 | 0.888 |
| felodipine | miconazole | 0.770 | 0.715 |
| acetylsalicylic_acid | bisacodyl | 0.770 | 0.760 |
| mephenytoin | saquinavir | 0.770 | 0.812 |
| fenoterol | nortriptyline | 0.770 | 0.758 |
| aminohippuric_acid | diltiazem | 0.770 | 0.743 |
| tyloxapol | zimeldine | 0.770 | 0.876 |
| roxithromycin | simvastatin | 0.770 | 0.785 |
| loperamide | oxyphenbutazone | 0.770 | 0.746 |
| (-)-catechin | clindamycin | 0.770 | 0.737 |
| dicycloverine | terfenadine | 0.770 | 0.712 |
| dipyridamole | estriol | 0.770 | 0.746 |
| amiodarone | clomipramine | 0.770 | 0.748 |
| cetirizine | daunorubicin | 0.770 | 0.745 |
| haloperidol | prenylamine | 0.770 | 0.714 |
| acetylsalicylic_acid | glibenclamide | 0.770 | 0.795 |
| phentolamine | proglumide | 0.770 | 0.689 |
| mitoxantrone | tropicamide | 0.770 | 0.767 |
| pimethixene | terfenadine | 0.770 | 0.795 |
| benzylpenicillin | dacarbazine | 0.770 | 0.743 |
| acetylsalicylic_acid | capsaicin | 0.770 | 0.758 |
| nicotinic_acid | scopolamine | 0.770 | 0.724 |
| benperidol | flufenamic_acid | 0.770 | 0.691 |
| betaxolol | trazodone | 0.770 | 0.699 |
| cimetidine | dirithromycin | 0.770 | 0.842 |
| azacitidine | felodipine | 0.770 | 0.745 |
| droperidol | fusidic_acid | 0.770 | 0.704 |
| estriol | sulfaphenazole | 0.771 | 0.717 |
| clomifene | estrone | 0.771 | 0.755 |
| aciclovir | felbinac | 0.771 | 0.663 |

| | | | |
|---|---|---|---|
| mephenesin | tiapride | 0.771 | 0.694 |
| flucytosine | terguride | 0.771 | 0.810 |
| guanethidine | terbutaline | 0.771 | 0.668 |
| clemastine | loperamide | 0.771 | 0.728 |
| cefamandole | mephentermine | 0.771 | 0.810 |
| cyproheptadine | menadione | 0.771 | 0.743 |
| methapyrilene | zidovudine | 0.771 | 0.703 |
| irinotecan | semustine | 0.771 | 0.787 |
| mesalazine | verteporfin | 0.771 | 0.858 |
| rifabutin | trifluridine | 0.771 | 0.815 |
| meclozine | sulfamethoxypyridazir | 0.771 | 0.740 |
| bromperidol | etamivan | 0.771 | 0.688 |
| sulfamethoxypyridazine | tolfenamic_acid | 0.771 | 0.659 |
| benfotiamine | roxithromycin | 0.771 | 0.777 |
| bromperidol | metyrapone | 0.771 | 0.733 |
| diltiazem | paclitaxel | 0.771 | 0.796 |
| lynestrenol | phenoxybenzamine | 0.771 | 0.745 |
| methylergometrine | primidone | 0.771 | 0.756 |
| phenoxybenzamine | testosterone | 0.771 | 0.768 |
| etacrynic_acid | labetalol | 0.771 | 0.724 |
| oxprenolol | vidarabine | 0.771 | 0.697 |
| altretamine | metyrapone | 0.771 | 0.728 |
| phenoxybenzamine | trimipramine | 0.771 | 0.691 |
| acepromazine | ketanserin | 0.771 | 0.735 |
| piribedil | repaglinide | 0.771 | 0.728 |
| benperidol | selegiline | 0.771 | 0.734 |
| benzethonium_chloride | nortriptyline | 0.771 | 0.745 |
| rolitetracycline | ronidazole | 0.771 | 0.773 |
| bromocriptine | practolol | 0.771 | 0.776 |
| gliclazide | metyrapone | 0.771 | 0.733 |
| lomefloxacin | thiocolchicoside | 0.771 | 0.700 |
| dacarbazine | fluocinonide | 0.771 | 0.806 |
| atovaquone | etamsylate | 0.771 | 0.719 |
| etofylline | norethisterone | 0.771 | 0.727 |
| meticrane | proscillaridin | 0.771 | 0.809 |
| amiodarone | deptropine | 0.771 | 0.763 |
| ethotoin | meticrane | 0.771 | 0.723 |
| mesoridazine | meticrane | 0.771 | 0.716 |
| beclometasone | carbachol | 0.771 | 0.827 |
| chlorzoxazone | sulfathiazole | 0.771 | 0.718 |
| chlorprothixene | haloperidol | 0.771 | 0.762 |
| cefalexin | demecolcine | 0.771 | 0.730 |
| colchicine | disulfiram | 0.771 | 0.751 |
| dienestrol | semustine | 0.771 | 0.751 |
| doxazosin | fenoterol | 0.771 | 0.699 |
| betaxolol | phenoxybenzamine | 0.771 | 0.736 |
| chlorcyclizine | pramocaine | 0.771 | 0.758 |
| (-)-catechin | diltiazem | 0.771 | 0.701 |
| lymecycline | piperidolate | 0.771 | 0.787 |
| benzethonium_chloride | miconazole | 0.771 | 0.744 |

| | | | |
|---|---|---|---|
| doxorubicin | remoxipride | 0.771 | 0.697 |
| selegiline | theophylline | 0.771 | 0.756 |
| amoxapine | prenylamine | 0.771 | 0.741 |
| trazodone | triflusal | 0.771 | 0.777 |
| metaraminol | trichlormethiazide | 0.771 | 0.715 |
| proxyphylline | rimexolone | 0.771 | 0.758 |
| irinotecan | naloxone | 0.771 | 0.812 |
| bethanechol | prednicarbate | 0.771 | 0.812 |
| carbachol | ketoprofen | 0.771 | 0.722 |
| budesonide | sulfasalazine | 0.771 | 0.737 |
| clomipramine | medrysone | 0.771 | 0.776 |
| hydrocortisone | sulconazole | 0.771 | 0.771 |
| propylthiouracil | thiocolchicoside | 0.771 | 0.777 |
| selegiline | trichlormethiazide | 0.771 | 0.761 |
| diltiazem | ronidazole | 0.771 | 0.753 |
| capsaicin | etofylline | 0.771 | 0.691 |
| bepridil | droperidol | 0.771 | 0.769 |
| mebendazole | oxedrine | 0.771 | 0.670 |
| oxedrine | piperidolate | 0.771 | 0.726 |
| repaglinide | trazodone | 0.771 | 0.733 |
| meclozine | omeprazole | 0.771 | 0.728 |
| demecolcine | mebendazole | 0.771 | 0.672 |
| piribedil | tyloxapol | 0.771 | 0.870 |
| phentolamine | trifluoperazine | 0.771 | 0.707 |
| flupentixol | pimozide | 0.771 | 0.715 |
| alfuzosin | mefloquine | 0.771 | 0.735 |
| dicoumarol | hydrocortisone | 0.771 | 0.722 |
| perhexiline | tribenoside | 0.771 | 0.757 |
| diclofenamide | tacrine | 0.771 | 0.684 |
| dacarbazine | rolitetracycline | 0.771 | 0.797 |
| benzethonium_chloride | butoconazole | 0.771 | 0.744 |
| cycloserine | sulfametoxydiazine | 0.771 | 0.810 |
| piracetam | propafenone | 0.771 | 0.764 |
| propylthiouracil | torasemide | 0.771 | 0.727 |
| desoxycortone | talampicillin | 0.771 | 0.735 |
| bromocriptine | vorinostat | 0.771 | 0.760 |
| chlorphenesin | pargyline | 0.771 | 0.659 |
| clomifene | pimozide | 0.771 | 0.739 |
| aminohippuric_acid | ipratropium_bromide | 0.771 | 0.704 |
| cefsulodin | chloropyramine | 0.771 | 0.790 |
| propafenone | remoxipride | 0.771 | 0.682 |
| celecoxib | decitabine | 0.771 | 0.735 |
| cefalotin | phenazopyridine | 0.771 | 0.718 |
| ethisterone | metamizole_sodium | 0.771 | 0.723 |
| tranylcypromine | trioxysalen | 0.771 | 0.674 |
| ipratropium_bromide | meticrane | 0.771 | 0.692 |
| acepromazine | liothyronine | 0.771 | 0.742 |
| clotrimazole | flupentixol | 0.771 | 0.761 |
| cimetidine | glibenclamide | 0.771 | 0.739 |
| benfluorex | dacarbazine | 0.771 | 0.749 |

| | | | |
|---|---|---|---|
| cefalotin | sulfaphenazole | 0.771 | 0.722 |
| diltiazem | midecamycin | 0.771 | 0.803 |
| iopanoic_acid | repaglinide | 0.771 | 0.733 |
| atovaquone | medrysone | 0.771 | 0.743 |
| procaine | propylthiouracil | 0.771 | 0.669 |
| cyanocobalamin | metaraminol | 0.771 | 0.903 |
| diltiazem | milrinone | 0.771 | 0.779 |
| promethazine | ronidazole | 0.771 | 0.704 |
| flunisolide | metamizole_sodium | 0.771 | 0.699 |
| betahistine | meptazinol | 0.771 | 0.659 |
| fludroxycortide | latamoxef | 0.771 | 0.734 |
| ipratropium_bromide | meropenem | 0.771 | 0.723 |
| acetylsalicylic_acid | etidronic_acid | 0.771 | 0.703 |
| imipramine | ivermectin | 0.771 | 0.876 |
| metamizole_sodium | metaraminol | 0.771 | 0.693 |
| chlorphenesin | nimesulide | 0.771 | 0.661 |
| protriptyline | troglitazone | 0.771 | 0.775 |
| chloroquine | oxamic_acid | 0.771 | 0.865 |
| naltrexone | promazine | 0.771 | 0.735 |
| calcium_pantothenate | metamizole_sodium | 0.771 | 0.797 |
| metamizole_sodium | oxybuprocaine | 0.771 | 0.707 |
| norethisterone | promethazine | 0.771 | 0.771 |
| flavoxate | iopanoic_acid | 0.771 | 0.717 |
| flurbiprofen | terguride | 0.771 | 0.703 |
| ticlopidine | verteporfin | 0.771 | 0.832 |
| liothyronine | procyclidine | 0.771 | 0.743 |
| fipexide | propylthiouracil | 0.771 | 0.750 |
| buflomedil | ketanserin | 0.771 | 0.697 |
| reserpine | zimeldine | 0.771 | 0.756 |
| emetine | mepacrine | 0.771 | 0.730 |
| mebendazole | rescinnamine | 0.771 | 0.759 |
| fenoprofen | sulfamerazine | 0.771 | 0.670 |
| chloramphenicol | metaraminol | 0.771 | 0.660 |
| piribedil | roxithromycin | 0.771 | 0.840 |
| astemizole | triflupromazine | 0.771 | 0.736 |
| budesonide | procaine | 0.771 | 0.743 |
| carbachol | cefalotin | 0.771 | 0.806 |
| clofazimine | perphenazine | 0.771 | 0.727 |
| chloramphenicol | estriol | 0.771 | 0.722 |
| clonidine | sirolimus | 0.771 | 0.893 |
| nialamide | pramocaine | 0.771 | 0.701 |
| flufenamic_acid | tobramycin | 0.771 | 0.772 |
| clomifene | nortriptyline | 0.771 | 0.708 |
| artemisinin | mesoridazine | 0.771 | 0.762 |
| flupentixol | trazodone | 0.771 | 0.715 |
| doxorubicin | meropenem | 0.771 | 0.718 |
| cefixime | cetirizine | 0.771 | 0.730 |
| ifenprodil | lisinopril | 0.771 | 0.731 |
| ceftazidime | etofylline | 0.771 | 0.744 |
| hydrocortisone | tobramycin | 0.771 | 0.709 |

| | | | |
|---|---|---|---|
| piribedil | promazine | 0.771 | 0.725 |
| bromopride | deferoxamine | 0.771 | 0.825 |
| roxithromycin | tropicamide | 0.772 | 0.827 |
| piromidic_acid | promethazine | 0.772 | 0.706 |
| deptropine | tolazoline | 0.772 | 0.753 |
| heptaminol | thiamphenicol | 0.772 | 0.739 |
| etacrynic_acid | syrosingopine | 0.772 | 0.808 |
| imipramine | oxetacaine | 0.772 | 0.782 |
| naloxone | sulfafurazole | 0.772 | 0.722 |
| sulfamerazine | tyloxapol | 0.772 | 0.876 |
| chlorzoxazone | meticrane | 0.772 | 0.697 |
| dyclonine | enalapril | 0.772 | 0.738 |
| chloroquine | etamivan | 0.772 | 0.675 |
| medrysone | vidarabine | 0.772 | 0.733 |
| chloramphenicol | chlorzoxazone | 0.772 | 0.765 |
| cinchocaine | phenazopyridine | 0.772 | 0.677 |
| betahistine | propafenone | 0.772 | 0.728 |
| meticrane | propylthiouracil | 0.772 | 0.683 |
| verteporfin | zimeldine | 0.772 | 0.802 |
| doxazosin | pargyline | 0.772 | 0.790 |
| cefalotin | ipratropium_bromide | 0.772 | 0.708 |
| acetylsalicylic_acid | omeprazole | 0.772 | 0.722 |
| aminohippuric_acid | estriol | 0.772 | 0.657 |
| flufenamic_acid | ioversol | 0.772 | 0.774 |
| nortriptyline | trazodone | 0.772 | 0.757 |
| amoxicillin | triflusal | 0.772 | 0.746 |
| pentoxyverine | piperidolate | 0.772 | 0.653 |
| benfluorex | chlorprothixene | 0.772 | 0.754 |
| trazodone | zimeldine | 0.772 | 0.728 |
| natamycin | talampicillin | 0.772 | 0.768 |
| fenbendazole | prenylamine | 0.772 | 0.726 |
| ifenprodil | ketanserin | 0.772 | 0.682 |
| liothyronine | metacycline | 0.772 | 0.713 |
| liothyronine | miconazole | 0.772 | 0.739 |
| reserpine | trimipramine | 0.772 | 0.805 |
| proscillaridin | vorinostat | 0.772 | 0.734 |
| penbutolol | terfenadine | 0.772 | 0.735 |
| bumetanide | prednisolone | 0.772 | 0.741 |
| desoxycortone | pentoxyverine | 0.772 | 0.763 |
| cefixime | gliclazide | 0.772 | 0.689 |
| clomifene | prenylamine | 0.772 | 0.705 |
| bemegride | colecalciferol | 0.772 | 0.775 |
| haloperidol | zuclopenthixol | 0.772 | 0.701 |
| carbachol | famotidine | 0.772 | 0.785 |
| dextromethorphan | ganciclovir | 0.772 | 0.741 |
| ipratropium_bromide | remoxipride | 0.772 | 0.690 |
| clemastine | niclosamide | 0.772 | 0.758 |
| acepromazine | pentoxyverine | 0.772 | 0.756 |
| cefixime | diltiazem | 0.772 | 0.729 |
| etidronic_acid | metyrapone | 0.772 | 0.759 |

| | | | |
|---|---|---|---|
| desoxycortone | oxprenolol | 0.772 | 0.757 |
| etofylline | talampicillin | 0.772 | 0.767 |
| fluticasone | zalcitabine | 0.772 | 0.782 |
| procaine | talampicillin | 0.772 | 0.744 |
| bromperidol | fluspirilene | 0.772 | 0.671 |
| fluphenazine | syrosingopine | 0.772 | 0.745 |
| amiodarone | pyrvinium | 0.772 | 0.750 |
| roxithromycin | sulfametoxydiazine | 0.772 | 0.849 |
| bisacodyl | procaine | 0.772 | 0.749 |
| benzethonium_chloride | mifepristone | 0.772 | 0.797 |
| oxprenolol | sertaconazole | 0.772 | 0.730 |
| bumetanide | fenofibrate | 0.772 | 0.725 |
| etofenamate | nafcillin | 0.772 | 0.677 |
| chlortalidone | pyrithyldione | 0.772 | 0.756 |
| haloperidol | protriptyline | 0.772 | 0.768 |
| pentetrazol | sulfametoxydiazine | 0.772 | 0.742 |
| miconazole | tyloxapol | 0.772 | 0.859 |
| doxorubicin | piribedil | 0.772 | 0.686 |
| benzethonium_chloride | levomepromazine | 0.772 | 0.777 |
| metergoline | promethazine | 0.772 | 0.749 |
| altizide | zomepirac | 0.772 | 0.654 |
| bromperidol | xylometazoline | 0.772 | 0.780 |
| imipenem | oxprenolol | 0.772 | 0.729 |
| doxazosin | oxybuprocaine | 0.772 | 0.711 |
| simvastatin | sulfafurazole | 0.772 | 0.777 |
| (-)-catechin | hyoscyamine | 0.772 | 0.672 |
| bendroflumethiazide | verteporfin | 0.772 | 0.769 |
| flufenamic_acid | gefitinib | 0.772 | 0.717 |
| clomipramine | famotidine | 0.772 | 0.802 |
| betaxolol | estriol | 0.772 | 0.717 |
| galantamine | nafcillin | 0.772 | 0.752 |
| azlocillin | ifosfamide | 0.772 | 0.759 |
| aminophylline | carbachol | 0.772 | 0.732 |
| carbachol | fluspirilene | 0.772 | 0.790 |
| rescinnamine | sirolimus | 0.772 | 0.801 |
| amoxapine | phenoxybenzamine | 0.772 | 0.734 |
| isocarboxazid | mephentermine | 0.772 | 0.660 |
| suloctidil | tribenoside | 0.772 | 0.741 |
| medrysone | remoxipride | 0.772 | 0.723 |
| mepacrine | oxytetracycline | 0.772 | 0.754 |
| clomipramine | quinisocaine | 0.772 | 0.656 |
| gliclazide | ketanserin | 0.772 | 0.675 |
| atovaquone | tobramycin | 0.772 | 0.758 |
| meclofenoxate | promethazine | 0.772 | 0.738 |
| acenocoumarol | neomycin | 0.772 | 0.790 |
| halcinonide | talampicillin | 0.772 | 0.714 |
| glafenine | mebendazole | 0.772 | 0.657 |
| mebendazole | quinisocaine | 0.772 | 0.680 |
| demecolcine | diltiazem | 0.772 | 0.739 |
| etofenamate | flavoxate | 0.772 | 0.665 |

| | | | |
|---|---|---|---|
| artemisinin | gefitinib | 0.772 | 0.813 |
| fenbendazole | lanatoside_C | 0.772 | 0.865 |
| deptropine | pargyline | 0.772 | 0.685 |
| acetylsalicylic_acid | repaglinide | 0.772 | 0.721 |
| amitriptyline | haloperidol | 0.772 | 0.766 |
| fluspirilene | prasterone | 0.772 | 0.737 |
| demecolcine | lomustine | 0.772 | 0.737 |
| dextromethorphan | dobutamine | 0.772 | 0.764 |
| pimethixene | pimozide | 0.772 | 0.782 |
| astemizole | penbutolol | 0.772 | 0.740 |
| carbachol | halcinonide | 0.772 | 0.801 |
| amoxicillin | dobutamine | 0.772 | 0.721 |
| aminophylline | betahistine | 0.772 | 0.665 |
| doxazosin | urapidil | 0.772 | 0.669 |
| orciprenaline | sirolimus | 0.772 | 0.871 |
| bromocriptine | salbutamol | 0.772 | 0.789 |
| ceftazidime | gliclazide | 0.772 | 0.733 |
| desoxycortone | etofenamate | 0.772 | 0.723 |
| pivampicillin | sulfaphenazole | 0.772 | 0.719 |
| cinchocaine | methylergometrine | 0.772 | 0.679 |
| monobenzone | triflusal | 0.772 | 0.750 |
| propylthiouracil | roxithromycin | 0.772 | 0.881 |
| colchicine | perhexiline | 0.772 | 0.753 |
| clotrimazole | felodipine | 0.772 | 0.701 |
| cefalexin | procainamide | 0.772 | 0.687 |
| chlorambucil | tinidazole | 0.772 | 0.738 |
| cycloserine | dipyridamole | 0.772 | 0.894 |
| medrysone | sulfamethoxazole | 0.772 | 0.733 |
| doxazosin | fluorometholone | 0.772 | 0.770 |
| clotrimazole | tribenoside | 0.772 | 0.772 |
| nadolol | tolnaftate | 0.772 | 0.722 |
| aminohippuric_acid | cetirizine | 0.772 | 0.730 |
| mycophenolic_acid | phenoxybenzamine | 0.772 | 0.746 |
| flunarizine | meticrane | 0.772 | 0.802 |
| amoxicillin | promethazine | 0.772 | 0.794 |
| fluocinonide | piribedil | 0.772 | 0.744 |
| propafenone | trazodone | 0.772 | 0.665 |
| flufenamic_acid | protriptyline | 0.772 | 0.707 |
| naftidrofuryl | oxantel | 0.772 | 0.756 |
| amiodarone | betahistine | 0.773 | 0.769 |
| disopyramide | nilutamide | 0.773 | 0.741 |
| pargyline | pentoxyverine | 0.773 | 0.737 |
| flunisolide | piromidic_acid | 0.773 | 0.739 |
| promethazine | pyrazinamide | 0.773 | 0.766 |
| tamoxifen | terfenadine | 0.773 | 0.715 |
| apomorphine | dipyridamole | 0.773 | 0.728 |
| clindamycin | rimexolone | 0.773 | 0.722 |
| fenoprofen | verteporfin | 0.773 | 0.819 |
| meptazinol | practolol | 0.773 | 0.729 |
| primaquine | succinylsulfathiazole | 0.773 | 0.718 |

| | | | |
|---|---|---|---|
| repaglinide | thiocolchicoside | 0.773 | 0.740 |
| budesonide | piracetam | 0.773 | 0.826 |
| benzylpenicillin | bisacodyl | 0.773 | 0.731 |
| dipyridamole | vidarabine | 0.773 | 0.726 |
| atovaquone | daunorubicin | 0.773 | 0.686 |
| metamizole_sodium | promethazine | 0.773 | 0.691 |
| remoxipride | tetryzoline | 0.773 | 0.704 |
| dihydroergocristine | dinoprost | 0.773 | 0.738 |
| hyoscyamine | oxprenolol | 0.773 | 0.714 |
| amoxapine | naloxone | 0.773 | 0.692 |
| benzylpenicillin | chlorzoxazone | 0.773 | 0.773 |
| monobenzone | pentoxyverine | 0.773 | 0.740 |
| trazodone | vidarabine | 0.773 | 0.740 |
| bepridil | medrysone | 0.773 | 0.779 |
| nifuroxazide | phenoxybenzamine | 0.773 | 0.705 |
| bromopride | flunisolide | 0.773 | 0.738 |
| meclofenoxate | repaglinide | 0.773 | 0.727 |
| rimexolone | thiocolchicoside | 0.773 | 0.762 |
| cefixime | ketanserin | 0.773 | 0.668 |
| acepromazine | bepridil | 0.773 | 0.727 |
| ganciclovir | rolitetracycline | 0.773 | 0.746 |
| tobramycin | tyloxapol | 0.773 | 0.833 |
| simvastatin | zimeldine | 0.773 | 0.746 |
| meptazinol | monobenzone | 0.773 | 0.685 |
| molindone | nitrofurantoin | 0.773 | 0.661 |
| bepridil | deptropine | 0.773 | 0.720 |
| penbutolol | risperidone | 0.773 | 0.751 |
| etamivan | trimetazidine | 0.773 | 0.674 |
| chloroquine | demecolcine | 0.773 | 0.685 |
| cyproterone | metyrapone | 0.773 | 0.727 |
| dipyridamole | etamsylate | 0.773 | 0.796 |
| flufenamic_acid | metacycline | 0.773 | 0.682 |
| clemastine | maprotiline | 0.773 | 0.653 |
| fludroxycortide | pyrazinamide | 0.773 | 0.827 |
| papaverine | syrosingopine | 0.773 | 0.775 |
| amikacin | enoxacin | 0.773 | 0.780 |
| syrosingopine | thioridazine | 0.773 | 0.797 |
| cefotaxime | topiramate | 0.773 | 0.777 |
| meticrane | monobenzone | 0.773 | 0.753 |
| pyrvinium | verteporfin | 0.773 | 0.799 |
| cetirizine | norethisterone | 0.773 | 0.771 |
| estriol | trimetazidine | 0.773 | 0.683 |
| tranylcypromine | tyloxapol | 0.773 | 0.903 |
| bisacodyl | ticlopidine | 0.773 | 0.708 |
| carisoprodol | pyrazinamide | 0.773 | 0.748 |
| azacitidine | ivermectin | 0.773 | 0.866 |
| memantine | tocainide | 0.773 | 0.737 |
| cefalexin | clebopride | 0.773 | 0.691 |
| amiodarone | progesterone | 0.773 | 0.761 |
| daunorubicin | trioxysalen | 0.773 | 0.779 |

| | | | |
|---|---|---|---|
| protriptyline | tribenoside | 0.773 | 0.772 |
| nafcillin | procyclidine | 0.773 | 0.718 |
| cefalexin | clomifene | 0.773 | 0.770 |
| citiolone | sulfaphenazole | 0.773 | 0.753 |
| flunarizine | sulfaphenazole | 0.773 | 0.733 |
| clomipramine | fluphenazine | 0.773 | 0.680 |
| mitoxantrone | omeprazole | 0.773 | 0.745 |
| cetirizine | fluvoxamine | 0.773 | 0.748 |
| cefalotin | sulfanilamide | 0.773 | 0.774 |
| benfluorex | carteolol | 0.773 | 0.674 |
| mesalazine | piperidolate | 0.773 | 0.756 |
| cortisone | pimethixene | 0.773 | 0.782 |
| liothyronine | urapidil | 0.773 | 0.725 |
| doxorubicin | procaine | 0.773 | 0.699 |
| nafcillin | ofloxacin | 0.773 | 0.690 |
| cefotaxime | isoniazid | 0.773 | 0.765 |
| bepridil | zimeldine | 0.773 | 0.705 |
| tobramycin | tolazamide | 0.773 | 0.757 |
| amitriptyline | fenbendazole | 0.773 | 0.741 |
| carbachol | cycloserine | 0.773 | 0.772 |
| clomipramine | vinpocetine | 0.773 | 0.691 |
| suloctidil | thioridazine | 0.773 | 0.737 |
| clofazimine | pyrvinium | 0.773 | 0.752 |
| bisacodyl | sulfametoxydiazine | 0.773 | 0.726 |
| betahistine | tobramycin | 0.773 | 0.856 |
| clomipramine | scopolamine | 0.773 | 0.755 |
| doxepin | loperamide | 0.773 | 0.754 |
| clioquinol | mefloquine | 0.773 | 0.735 |
| betaxolol | kanamycin | 0.773 | 0.795 |
| amiodarone | butoconazole | 0.773 | 0.743 |
| doxorubicin | levamisole | 0.773 | 0.778 |
| propofol | pyrvinium | 0.773 | 0.818 |
| primaquine | sulfapyridine | 0.773 | 0.670 |
| metamizole_sodium | tyloxapol | 0.773 | 0.856 |
| glibenclamide | gliclazide | 0.773 | 0.661 |
| bromopride | cortisone | 0.773 | 0.737 |
| deptropine | xylometazoline | 0.773 | 0.729 |
| doxorubicin | sertaconazole | 0.773 | 0.717 |
| hyoscyamine | rimexolone | 0.773 | 0.716 |
| doxazosin | prilocaine | 0.773 | 0.710 |
| betahistine | estriol | 0.773 | 0.715 |
| dydrogesterone | sulconazole | 0.773 | 0.718 |
| ethisterone | primaquine | 0.773 | 0.733 |
| betaxolol | sulfametoxydiazine | 0.773 | 0.684 |
| griseofulvin | pentoxyverine | 0.773 | 0.751 |
| metamizole_sodium | remoxipride | 0.773 | 0.653 |
| moroxydine | phenazone | 0.773 | 0.664 |
| clomifene | imipramine | 0.773 | 0.718 |
| amiodarone | pargyline | 0.773 | 0.784 |
| amiodarone | estriol | 0.773 | 0.760 |

| | | | |
|---|---|---|---|
| bisacodyl | propylthiouracil | 0.773 | 0.780 |
| trimethoprim | tropicamide | 0.773 | 0.690 |
| betaxolol | cefalexin | 0.773 | 0.724 |
| hydralazine | piperidolate | 0.773 | 0.734 |
| griseofulvin | naloxone | 0.773 | 0.716 |
| butoconazole | mometasone | 0.773 | 0.761 |
| isradipine | nabumetone | 0.773 | 0.747 |
| clioquinol | terguride | 0.773 | 0.734 |
| bromopride | halcinonide | 0.773 | 0.758 |
| dacarbazine | trimetazidine | 0.773 | 0.681 |
| etodolac | levocabastine | 0.773 | 0.757 |
| mefenamic_acid | moxisylyte | 0.773 | 0.690 |
| melatonin | pralidoxime | 0.773 | 0.682 |
| chlorprothixene | pimozide | 0.773 | 0.784 |
| cetirizine | propantheline_bromic | 0.773 | 0.748 |
| fluvastatin | terfenadine | 0.773 | 0.748 |
| atovaquone | verteporfin | 0.773 | 0.792 |
| chlortetracycline | omeprazole | 0.773 | 0.714 |
| cyproheptadine | pimozide | 0.773 | 0.785 |
| promazine | pyrazinamide | 0.773 | 0.735 |
| cefalotin | cetirizine | 0.773 | 0.740 |
| clindamycin | oxprenolol | 0.773 | 0.720 |
| nafcillin | naltrexone | 0.773 | 0.721 |
| fludroxycortide | ofloxacin | 0.773 | 0.718 |
| chlorzoxazone | phenoxybenzamine | 0.773 | 0.748 |
| citalopram | paroxetine | 0.773 | 0.715 |
| atovaquone | progesterone | 0.773 | 0.743 |
| promazine | terfenadine | 0.773 | 0.773 |
| clomifene | pimethixene | 0.773 | 0.741 |
| metyrapone | verteporfin | 0.773 | 0.831 |
| molindone | sulfathiazole | 0.773 | 0.685 |
| dyclonine | trifluoperazine | 0.773 | 0.745 |
| fluspirilene | talampicillin | 0.773 | 0.735 |
| acetylsalicylic_acid | ifenprodil | 0.773 | 0.765 |
| felodipine | urapidil | 0.773 | 0.767 |
| pralidoxime | sulfathiazole | 0.773 | 0.675 |
| chlorprothixene | fenbendazole | 0.773 | 0.723 |
| felodipine | ketoprofen | 0.773 | 0.717 |
| cefalotin | methazolamide | 0.773 | 0.745 |
| bisacodyl | terfenadine | 0.773 | 0.726 |
| levamisole | menadione | 0.773 | 0.681 |
| ambroxol | sulfathiazole | 0.773 | 0.690 |
| bisacodyl | ciclopirox | 0.773 | 0.764 |
| isocarboxazid | terconazole | 0.773 | 0.768 |
| budesonide | imipenem | 0.773 | 0.732 |
| etamsylate | flufenamic_acid | 0.773 | 0.671 |
| daunorubicin | rolitetracycline | 0.773 | 0.662 |
| fluspirilene | trazodone | 0.773 | 0.684 |
| benperidol | trioxysalen | 0.774 | 0.766 |
| imipenem | mesoridazine | 0.774 | 0.737 |

| | | | |
|---|---|---|---|
| cycloserine | sulfaphenazole | 0.774 | 0.821 |
| guaifenesin | propofol | 0.774 | 0.678 |
| methylergometrine | nalidixic_acid | 0.774 | 0.698 |
| carbachol | medrysone | 0.774 | 0.791 |
| nicergoline | protriptyline | 0.774 | 0.745 |
| cyproheptadine | ketanserin | 0.774 | 0.769 |
| idoxuridine | meptazinol | 0.774 | 0.706 |
| bisacodyl | rolitetracycline | 0.774 | 0.745 |
| ketanserin | medrysone | 0.774 | 0.721 |
| monobenzone | pyrazinamide | 0.774 | 0.707 |
| fenoterol | trifluoperazine | 0.774 | 0.733 |
| betahistine | milrinone | 0.774 | 0.709 |
| bupivacaine | lisinopril | 0.774 | 0.738 |
| nicergoline | terconazole | 0.774 | 0.736 |
| meropenem | urapidil | 0.774 | 0.722 |
| metamizole_sodium | phenindione | 0.774 | 0.698 |
| bisacodyl | ethotoin | 0.774 | 0.742 |
| oxprenolol | tyloxapol | 0.774 | 0.867 |
| norethisterone | ornidazole | 0.774 | 0.758 |
| perhexiline | thioridazine | 0.774 | 0.687 |
| nitrofurantoin | trazodone | 0.774 | 0.710 |
| dacarbazine | lymecycline | 0.774 | 0.815 |
| loperamide | thiethylperazine | 0.774 | 0.705 |
| cefalexin | sulfamethoxypyridazir | 0.774 | 0.661 |
| benzonatate | moxisylyte | 0.774 | 0.809 |
| calcium_pantothenate | ketanserin | 0.774 | 0.740 |
| fluspirilene | quinisocaine | 0.774 | 0.738 |
| liothyronine | ursodeoxycholic_acid | 0.774 | 0.701 |
| doxazosin | ornidazole | 0.774 | 0.736 |
| cetirizine | estriol | 0.774 | 0.766 |
| cimetidine | doxylamine | 0.774 | 0.747 |
| fluvoxamine | nortriptyline | 0.774 | 0.736 |
| phenoxybenzamine | tribenoside | 0.774 | 0.749 |
| demecolcine | practolol | 0.774 | 0.722 |
| erastin | niclosamide | 0.774 | 0.757 |
| levamisole | meticrane | 0.774 | 0.716 |
| doxazosin | flunisolide | 0.774 | 0.742 |
| galantamine | simvastatin | 0.774 | 0.747 |
| meptazinol | pivampicillin | 0.774 | 0.795 |
| phenindione | thiocolchicoside | 0.774 | 0.795 |
| fludroxycortide | liothyronine | 0.774 | 0.728 |
| meropenem | reserpine | 0.774 | 0.740 |
| irinotecan | monobenzone | 0.774 | 0.793 |
| bromocriptine | etoposide | 0.774 | 0.743 |
| albendazole | loperamide | 0.774 | 0.729 |
| budesonide | milrinone | 0.774 | 0.782 |
| erastin | perphenazine | 0.774 | 0.723 |
| aminocaproic_acid | rimexolone | 0.774 | 0.779 |
| disulfiram | tamoxifen | 0.774 | 0.778 |
| doxorubicin | nabumetone | 0.774 | 0.736 |

| | | | |
|---|---|---|---|
| mebendazole | miconazole | 0.774 | 0.724 |
| capsaicin | diltiazem | 0.774 | 0.764 |
| medrysone | metacycline | 0.774 | 0.711 |
| azacitidine | doxazosin | 0.774 | 0.750 |
| capsaicin | sulfametoxydiazine | 0.774 | 0.693 |
| omeprazole | piromidic_acid | 0.774 | 0.668 |
| benperidol | clindamycin | 0.774 | 0.737 |
| piribedil | sulfamethoxazole | 0.774 | 0.677 |
| cefalotin | famotidine | 0.774 | 0.714 |
| bepridil | dextromethorphan | 0.774 | 0.730 |
| biotin | nicotinic_acid | 0.774 | 0.764 |
| griseofulvin | tyloxapol | 0.774 | 0.852 |
| acetohexamide | meticrane | 0.774 | 0.703 |
| torasemide | trimetazidine | 0.774 | 0.708 |
| sulfaphenazole | tyloxapol | 0.774 | 0.868 |
| methylprednisolone | sulconazole | 0.774 | 0.762 |
| imipramine | noretynodrel | 0.774 | 0.746 |
| pentoxyverine | pipemidic_acid | 0.774 | 0.762 |
| diltiazem | perhexiline | 0.774 | 0.732 |
| levamisole | verteporfin | 0.774 | 0.842 |
| isoconazole | pirenzepine | 0.774 | 0.720 |
| oxprenolol | terguride | 0.774 | 0.707 |
| gliclazide | pargyline | 0.774 | 0.753 |
| altizide | metoprolol | 0.774 | 0.695 |
| perhexiline | phenoxybenzamine | 0.774 | 0.705 |
| flupentixol | sulfaphenazole | 0.774 | 0.723 |
| acetylsalicylic_acid | daunorubicin | 0.774 | 0.762 |
| (-)-atenolol | etomidate | 0.774 | 0.736 |
| thiocolchicoside | tropicamide | 0.774 | 0.772 |
| meclozine | metyrapone | 0.774 | 0.749 |
| clidinium_bromide | pyrithyldione | 0.774 | 0.750 |
| betaxolol | trioxysalen | 0.774 | 0.750 |
| latamoxef | zimeldine | 0.774 | 0.792 |
| desipramine | pyrvinium | 0.774 | 0.791 |
| etomidate | sulfamerazine | 0.774 | 0.679 |
| acetylsalicylic_acid | ipratropium_bromide | 0.774 | 0.699 |
| pimozide | rescinnamine | 0.774 | 0.750 |
| etamsylate | zimeldine | 0.774 | 0.768 |
| clindamycin | trifluridine | 0.774 | 0.739 |
| cetirizine | naloxone | 0.774 | 0.768 |
| phenoxybenzamine | sulfafurazole | 0.774 | 0.722 |
| betahistine | doxylamine | 0.774 | 0.662 |
| nitrofurantoin | phenoxybenzamine | 0.774 | 0.757 |
| astemizole | mianserin | 0.774 | 0.763 |
| capsaicin | flufenamic_acid | 0.774 | 0.712 |
| oxaprozin | tyloxapol | 0.774 | 0.855 |
| liothyronine | oxolinic_acid | 0.774 | 0.696 |
| benperidol | sulfathiazole | 0.774 | 0.743 |
| etamsylate | ifenprodil | 0.774 | 0.790 |
| aminohippuric_acid | ketanserin | 0.774 | 0.697 |

| | | | |
|---|---|---|---|
| chlorzoxazone | hydrocortisone | 0.774 | 0.823 |
| biotin | progesterone | 0.774 | 0.682 |
| chlorambucil | sulconazole | 0.774 | 0.697 |
| gliclazide | oxamic_acid | 0.774 | 0.909 |
| ioversol | propranolol | 0.774 | 0.788 |
| chlorzoxazone | mepyramine | 0.774 | 0.763 |
| daunorubicin | tropicamide | 0.774 | 0.746 |
| demecolcine | sulfametoxydiazine | 0.774 | 0.741 |
| guaifenesin | paroxetine | 0.774 | 0.701 |
| cefalotin | ketoprofen | 0.774 | 0.715 |
| griseofulvin | promethazine | 0.774 | 0.698 |
| cycloserine | trimetazidine | 0.774 | 0.808 |
| gliclazide | miconazole | 0.774 | 0.756 |
| demecolcine | digoxin | 0.774 | 0.846 |
| cefixime | fenoprofen | 0.774 | 0.768 |
| bezafibrate | metacycline | 0.774 | 0.736 |
| dyclonine | pergolide | 0.774 | 0.731 |
| aztreonam | ethosuximide | 0.774 | 0.812 |
| capsaicin | rimexolone | 0.774 | 0.752 |
| cycloserine | trioxysalen | 0.774 | 0.914 |
| dacarbazine | meclozine | 0.774 | 0.813 |
| (-)-atenolol | sulfamethoxypyridazir | 0.774 | 0.692 |
| altizide | verteporfin | 0.774 | 0.778 |
| benfluorex | etodolac | 0.774 | 0.725 |
| pentamidine | tyloxapol | 0.774 | 0.863 |
| dipyridamole | guanfacine | 0.774 | 0.783 |
| metamizole_sodium | sulfamerazine | 0.774 | 0.669 |
| dipivefrine | syrosingopine | 0.774 | 0.782 |
| deferoxamine | trifluridine | 0.774 | 0.822 |
| benperidol | guanfacine | 0.774 | 0.754 |
| imipenem | zimeldine | 0.774 | 0.733 |
| diclofenac | repaglinide | 0.774 | 0.710 |
| metixene | mometasone | 0.774 | 0.762 |
| clebopride | pentoxyverine | 0.774 | 0.692 |
| fludroxycortide | nalidixic_acid | 0.774 | 0.759 |
| molindone | trioxysalen | 0.774 | 0.697 |
| ipratropium_bromide | repaglinide | 0.774 | 0.722 |
| carbachol | proscillaridin | 0.774 | 0.839 |
| capsaicin | cefalexin | 0.774 | 0.732 |
| labetalol | pimozide | 0.774 | 0.696 |
| meclozine | sulfametoxydiazine | 0.774 | 0.730 |
| oxprenolol | ronidazole | 0.774 | 0.697 |
| dirithromycin | rimexolone | 0.774 | 0.795 |
| antazoline | vinpocetine | 0.774 | 0.712 |
| galantamine | triflusal | 0.774 | 0.712 |
| griseofulvin | piribedil | 0.774 | 0.711 |
| chlorzoxazone | gliclazide | 0.774 | 0.749 |
| ipratropium_bromide | methazolamide | 0.774 | 0.745 |
| bisacodyl | prenylamine | 0.774 | 0.709 |
| flunarizine | levocabastine | 0.774 | 0.723 |

| | | | |
|---|---|---|---|
| estrone | pentetrazol | 0.774 | 0.762 |
| enalapril | topiramate | 0.774 | 0.733 |
| proscillaridin | tyloxapol | 0.774 | 0.815 |
| azacitidine | fenoprofen | 0.774 | 0.714 |
| betahistine | gliclazide | 0.774 | 0.710 |
| fenoterol | iopanoic_acid | 0.774 | 0.724 |
| clomipramine | mebendazole | 0.774 | 0.758 |
| dacarbazine | repaglinide | 0.774 | 0.770 |
| levonorgestrel | verteporfin | 0.774 | 0.820 |
| deptropine | ifenprodil | 0.775 | 0.754 |
| torasemide | triflusal | 0.775 | 0.730 |
| bromocriptine | clomifene | 0.775 | 0.752 |
| cinchocaine | meticrane | 0.775 | 0.718 |
| betahistine | piperidolate | 0.775 | 0.704 |
| melatonin | vigabatrin | 0.775 | 0.726 |
| latamoxef | verteporfin | 0.775 | 0.783 |
| pivampicillin | promethazine | 0.775 | 0.807 |
| daunorubicin | physostigmine | 0.775 | 0.778 |
| progesterone | propranolol | 0.775 | 0.755 |
| etamsylate | procaine | 0.775 | 0.677 |
| ronidazole | sulfaphenazole | 0.775 | 0.701 |
| cyproterone | daunorubicin | 0.775 | 0.728 |
| chlorprothixene | clotrimazole | 0.775 | 0.725 |
| benzathine_benzylpenicillin | decitabine | 0.775 | 0.714 |
| gefitinib | zidovudine | 0.775 | 0.714 |
| oxprenolol | ramipril | 0.775 | 0.727 |
| levonorgestrel | liothyronine | 0.775 | 0.725 |
| acetazolamide | noretynodrel | 0.775 | 0.761 |
| amiodarone | benperidol | 0.775 | 0.711 |
| ifenprodil | ronidazole | 0.775 | 0.760 |
| mebendazole | sirolimus | 0.775 | 0.851 |
| benfluorex | cetirizine | 0.775 | 0.724 |
| cefaclor | lovastatin | 0.775 | 0.726 |
| altizide | meropenem | 0.775 | 0.694 |
| diltiazem | norfloxacin | 0.775 | 0.754 |
| fluphenazine | latamoxef | 0.775 | 0.751 |
| loracarbef | tridihexethyl | 0.775 | 0.743 |
| albendazole | terbutaline | 0.775 | 0.688 |
| phenindione | trimetazidine | 0.775 | 0.721 |
| levamisole | trioxysalen | 0.775 | 0.696 |
| guanethidine | sulconazole | 0.775 | 0.736 |
| astemizole | dilazep | 0.775 | 0.777 |
| piperidolate | tobramycin | 0.775 | 0.784 |
| maprotiline | mometasone | 0.775 | 0.770 |
| doxazosin | tiabendazole | 0.775 | 0.691 |
| carisoprodol | levothyroxine_sodium | 0.775 | 0.750 |
| metyrapone | sulfaphenazole | 0.775 | 0.659 |
| tobramycin | trimetazidine | 0.775 | 0.786 |
| flumequine | oxymetazoline | 0.775 | 0.681 |
| clonidine | perhexiline | 0.775 | 0.723 |

| | | | |
|---|---|---|---|
| dobutamine | nicardipine | 0.775 | 0.750 |
| desipramine | enoxacin | 0.775 | 0.720 |
| amiodarone | piribedil | 0.775 | 0.738 |
| cefalexin | tropicamide | 0.775 | 0.682 |
| cefalexin | estriol | 0.775 | 0.742 |
| flutamide | nialamide | 0.775 | 0.716 |
| cyproheptadine | mebendazole | 0.775 | 0.725 |
| mepacrine | pimozide | 0.775 | 0.745 |
| minaprine | tiapride | 0.775 | 0.680 |
| labetalol | triflusal | 0.775 | 0.727 |
| etidronic_acid | sulfametoxydiazine | 0.775 | 0.742 |
| fluspirilene | promazine | 0.775 | 0.758 |
| cefixime | mitoxantrone | 0.775 | 0.704 |
| liothyronine | tobramycin | 0.775 | 0.747 |
| bromocriptine | oleandomycin | 0.775 | 0.761 |
| acetylsalicylic_acid | metyrapone | 0.775 | 0.680 |
| norfloxacin | pentetrazol | 0.775 | 0.737 |
| procarbazine | terfenadine | 0.775 | 0.746 |
| ethotoin | medrysone | 0.775 | 0.772 |
| flunisolide | isosorbide | 0.775 | 0.811 |
| felodipine | rolitetracycline | 0.775 | 0.762 |
| deptropine | ronidazole | 0.775 | 0.759 |
| amoxapine | haloperidol | 0.775 | 0.758 |
| bisacodyl | capsaicin | 0.775 | 0.772 |
| acepromazine | pargyline | 0.775 | 0.700 |
| azacitidine | medrysone | 0.775 | 0.747 |
| bufexamac | trazodone | 0.775 | 0.715 |
| cefadroxil | levothyroxine_sodium | 0.775 | 0.730 |
| clindamycin | sulfametoxydiazine | 0.775 | 0.722 |
| ethotoin | trazodone | 0.775 | 0.691 |
| atovaquone | roxithromycin | 0.775 | 0.834 |
| benzathine_benzylpenicillin | levobunolol | 0.775 | 0.707 |
| amiodarone | protriptyline | 0.775 | 0.767 |
| omeprazole | scopolamine | 0.775 | 0.684 |
| levomepromazine | suloctidil | 0.775 | 0.743 |
| cinchocaine | talampicillin | 0.775 | 0.737 |
| menadione | perhexiline | 0.775 | 0.795 |
| gliclazide | mitoxantrone | 0.775 | 0.752 |
| altizide | dacarbazine | 0.775 | 0.726 |
| azlocillin | pridinol | 0.775 | 0.710 |
| clomifene | oxetacaine | 0.775 | 0.762 |
| ethotoin | rimexolone | 0.775 | 0.770 |
| bromocriptine | fendiline | 0.775 | 0.767 |
| pimethixene | protriptyline | 0.775 | 0.668 |
| gliclazide | iopanoic_acid | 0.775 | 0.718 |
| acepromazine | talampicillin | 0.775 | 0.765 |
| amoxapine | doxazosin | 0.775 | 0.769 |
| flupentixol | oxetacaine | 0.775 | 0.745 |
| citiolone | sulfamerazine | 0.775 | 0.734 |
| buflomedil | meticrane | 0.775 | 0.721 |

| | | | |
|---|---|---|---|
| roxithromycin | thiocolchicoside | 0.775 | 0.782 |
| bisacodyl | bromopride | 0.775 | 0.740 |
| carbachol | metacycline | 0.775 | 0.811 |
| betahistine | verteporfin | 0.775 | 0.854 |
| menadione | tyloxapol | 0.775 | 0.905 |
| levomepromazine | rifabutin | 0.775 | 0.816 |
| fenoterol | piribedil | 0.775 | 0.657 |
| cefoxitin | clidinium_bromide | 0.775 | 0.739 |
| daunorubicin | flufenamic_acid | 0.775 | 0.711 |
| amantadine | nystatin | 0.775 | 0.922 |
| benzylpenicillin | cycloserine | 0.775 | 0.809 |
| fipexide | oxytetracycline | 0.775 | 0.753 |
| flufenamic_acid | imipenem | 0.775 | 0.692 |
| menadione | methylergometrine | 0.775 | 0.737 |
| doxazosin | nabumetone | 0.775 | 0.705 |
| cinchocaine | oxprenolol | 0.775 | 0.650 |
| flufenamic_acid | phenacetin | 0.775 | 0.687 |
| miconazole | mometasone | 0.775 | 0.762 |
| sulfaphenazole | trioxysalen | 0.775 | 0.739 |
| oxprenolol | sulfamethoxypyridazir | 0.775 | 0.669 |
| ifenprodil | meticrane | 0.775 | 0.771 |
| liothyronine | nitrofurantoin | 0.775 | 0.724 |
| digoxin | hydroquinine | 0.775 | 0.832 |
| protriptyline | ursodeoxycholic_acid | 0.775 | 0.791 |
| reserpine | verteporfin | 0.775 | 0.762 |
| progesterone | reserpine | 0.775 | 0.784 |
| niclosamide | terconazole | 0.775 | 0.763 |
| mebendazole | mephenesin | 0.775 | 0.664 |
| gefitinib | idoxuridine | 0.775 | 0.723 |
| capsaicin | talampicillin | 0.775 | 0.727 |
| ethotoin | mitoxantrone | 0.775 | 0.756 |
| alprenolol | oxybutynin | 0.775 | 0.717 |
| diltiazem | oxybuprocaine | 0.775 | 0.748 |
| molindone | oxolinic_acid | 0.775 | 0.669 |
| benzethonium_chloride | erastin | 0.775 | 0.759 |
| cinnarizine | mifepristone | 0.775 | 0.790 |
| benzylpenicillin | nitrofurantoin | 0.775 | 0.710 |
| cefsulodin | paromomycin | 0.775 | 0.733 |
| guaifenesin | sulfamethoxypyridazir | 0.775 | 0.671 |
| clioquinol | clomifene | 0.775 | 0.770 |
| cyproterone | saquinavir | 0.775 | 0.803 |
| dobutamine | remoxipride | 0.775 | 0.729 |
| nialamide | rifabutin | 0.775 | 0.805 |
| (-)-catechin | clonidine | 0.775 | 0.729 |
| latamoxef | tribenoside | 0.775 | 0.734 |
| trimipramine | verteporfin | 0.775 | 0.801 |
| repaglinide | rolitetracycline | 0.775 | 0.737 |
| piperidolate | trioxysalen | 0.775 | 0.769 |
| ketanserin | tropicamide | 0.775 | 0.721 |
| octopamine | reserpine | 0.775 | 0.806 |

| | | | |
|---|---|---|---|
| enoxacin | nafcillin | 0.775 | 0.702 |
| bepridil | meticrane | 0.775 | 0.792 |
| bisacodyl | tropicamide | 0.775 | 0.741 |
| ambroxol | tranexamic_acid | 0.775 | 0.740 |
| clotrimazole | reserpine | 0.775 | 0.809 |
| oxytetracycline | sulconazole | 0.775 | 0.764 |
| bisacodyl | imipenem | 0.775 | 0.730 |
| piperidolate | pivampicillin | 0.775 | 0.749 |
| methylergometrine | trifluridine | 0.775 | 0.740 |
| carisoprodol | meclocycline | 0.775 | 0.728 |
| clonidine | fenoterol | 0.775 | 0.753 |
| milrinone | talampicillin | 0.775 | 0.796 |
| pridinol | zomepirac | 0.775 | 0.715 |
| dipyridamole | meclofenoxate | 0.775 | 0.767 |
| dacarbazine | hydrocortisone | 0.776 | 0.791 |
| flunisolide | gliclazide | 0.776 | 0.745 |
| ipratropium_bromide | milrinone | 0.776 | 0.752 |
| astemizole | ticlopidine | 0.776 | 0.761 |
| etomidate | procaine | 0.776 | 0.695 |
| propafenone | sulfasalazine | 0.776 | 0.725 |
| econazole | semustine | 0.776 | 0.722 |
| hydroflumethiazide | metrifonate | 0.776 | 0.739 |
| piribedil | tobramycin | 0.776 | 0.767 |
| atovaquone | dipyridamole | 0.776 | 0.760 |
| amikacin | promethazine | 0.776 | 0.829 |
| benperidol | simvastatin | 0.776 | 0.753 |
| diltiazem | meclozine | 0.776 | 0.723 |
| carbamazepine | labetalol | 0.776 | 0.731 |
| meclozine | methylergometrine | 0.776 | 0.725 |
| norethisterone | octopamine | 0.776 | 0.765 |
| rimexolone | sulfinpyrazone | 0.776 | 0.731 |
| bufexamac | nortriptyline | 0.776 | 0.737 |
| loxapine | trifluoperazine | 0.776 | 0.707 |
| benperidol | trimetazidine | 0.776 | 0.760 |
| bromocriptine | sodium_phenylbutyra | 0.776 | 0.794 |
| nortriptyline | pyrvinium | 0.776 | 0.781 |
| aztreonam | flumequine | 0.776 | 0.737 |
| ipratropium_bromide | proxyphylline | 0.776 | 0.752 |
| dipyridamole | felodipine | 0.776 | 0.763 |
| metamizole_sodium | pipemidic_acid | 0.776 | 0.653 |
| dipyridamole | tyloxapol | 0.776 | 0.818 |
| etoposide | prenylamine | 0.776 | 0.772 |
| altizide | piperidolate | 0.776 | 0.741 |
| fluspirilene | labetalol | 0.776 | 0.697 |
| bromocriptine | oxytetracycline | 0.776 | 0.740 |
| levomepromazine | phenazopyridine | 0.776 | 0.663 |
| ipratropium_bromide | pentoxyverine | 0.776 | 0.692 |
| chlorambucil | tolbutamide | 0.776 | 0.725 |
| (-)-isoprenaline | benzylpenicillin | 0.776 | 0.730 |
| ipratropium_bromide | zimeldine | 0.776 | 0.707 |

| | | | |
|---|---|---|---|
| scopolamine | zimeldine | 0.776 | 0.701 |
| colchicine | loperamide | 0.776 | 0.761 |
| dipyridamole | metamizole_sodium | 0.776 | 0.729 |
| trifluoperazine | zalcitabine | 0.776 | 0.723 |
| clioquinol | pimozide | 0.776 | 0.762 |
| levamisole | sulfametoxydiazine | 0.776 | 0.696 |
| nifuroxazide | suloctidil | 0.776 | 0.694 |
| dipivefrine | ifenprodil | 0.776 | 0.747 |
| fipexide | trioxysalen | 0.776 | 0.767 |
| doxazosin | trimipramine | 0.776 | 0.801 |
| oxprenolol | rimexolone | 0.776 | 0.749 |
| etomidate | roxithromycin | 0.776 | 0.855 |
| pentoxyverine | trimetazidine | 0.776 | 0.727 |
| levonorgestrel | natamycin | 0.776 | 0.815 |
| deptropine | dorzolamide | 0.776 | 0.726 |
| sulconazole | vinpocetine | 0.776 | 0.732 |
| dicycloverine | loperamide | 0.776 | 0.713 |
| prenylamine | primaquine | 0.776 | 0.754 |
| dacarbazine | norethisterone | 0.776 | 0.783 |
| doxazosin | flucytosine | 0.776 | 0.765 |
| levobunolol | rifampicin | 0.776 | 0.823 |
| rolitetracycline | verteporfin | 0.776 | 0.775 |
| diltiazem | natamycin | 0.776 | 0.787 |
| fludroxycortide | tyloxapol | 0.776 | 0.835 |
| disulfiram | thioproperazine | 0.776 | 0.757 |
| doxazosin | mebendazole | 0.776 | 0.662 |
| famotidine | oxantel | 0.776 | 0.745 |
| ciclopirox | suloctidil | 0.776 | 0.730 |
| pyrvinium | rescinnamine | 0.776 | 0.787 |
| cycloserine | levamisole | 0.776 | 0.795 |
| propantheline_bromide | rimexolone | 0.776 | 0.720 |
| aminocaproic_acid | zimeldine | 0.776 | 0.760 |
| amiloride | cinchocaine | 0.776 | 0.672 |
| chloramphenicol | lymecycline | 0.776 | 0.778 |
| flunisolide | pyrazinamide | 0.776 | 0.804 |
| budesonide | piribedil | 0.776 | 0.728 |
| clomifene | oxantel | 0.776 | 0.752 |
| apomorphine | cyproheptadine | 0.776 | 0.673 |
| bepridil | etofenamate | 0.776 | 0.764 |
| fipexide | mepyramine | 0.776 | 0.739 |
| butoconazole | latamoxef | 0.776 | 0.787 |
| ketanserin | liothyronine | 0.776 | 0.692 |
| bezafibrate | medrysone | 0.776 | 0.747 |
| bisacodyl | fipexide | 0.776 | 0.721 |
| dacarbazine | talampicillin | 0.776 | 0.772 |
| procaine | tolfenamic_acid | 0.776 | 0.653 |
| clotrimazole | oxamic_acid | 0.776 | 0.903 |
| minaprine | naloxone | 0.776 | 0.759 |
| glipizide | metolazone | 0.776 | 0.716 |
| galantamine | talampicillin | 0.776 | 0.777 |

| | | | |
|---|---|---|---|
| (-)-catechin | chloramphenicol | 0.776 | 0.693 |
| bromperidol | repaglinide | 0.776 | 0.751 |
| diltiazem | flufenamic_acid | 0.776 | 0.710 |
| meticrane | pyrantel | 0.776 | 0.700 |
| trichlormethiazide | trioxysalen | 0.776 | 0.696 |
| dextromethorphan | loperamide | 0.776 | 0.768 |
| proguanil | topiramate | 0.776 | 0.772 |
| sulfamethoxazole | tobramycin | 0.776 | 0.781 |
| debrisoquine | diclofenamide | 0.776 | 0.662 |
| deptropine | metyrapone | 0.776 | 0.724 |
| nicotinic_acid | remoxipride | 0.776 | 0.713 |
| metamizole_sodium | milrinone | 0.776 | 0.697 |
| ronidazole | zimeldine | 0.776 | 0.727 |
| propylthiouracil | sulfametoxydiazine | 0.776 | 0.702 |
| cefazolin | metaraminol | 0.776 | 0.806 |
| natamycin | ticarcillin | 0.776 | 0.769 |
| betahistine | phenoxybenzamine | 0.776 | 0.699 |
| bepridil | promazine | 0.776 | 0.686 |
| bromocriptine | dilazep | 0.776 | 0.760 |
| liothyronine | propylthiouracil | 0.776 | 0.754 |
| amoxapine | mefloquine | 0.776 | 0.698 |
| atovaquone | flufenamic_acid | 0.776 | 0.703 |
| ranitidine | sulconazole | 0.776 | 0.737 |
| chlorcyclizine | hexetidine | 0.776 | 0.721 |
| aminophenazone | spironolactone | 0.776 | 0.800 |
| gliclazide | remoxipride | 0.776 | 0.683 |
| beclometasone | trichlormethiazide | 0.776 | 0.732 |
| pyrantel | repaglinide | 0.776 | 0.797 |
| etamsylate | propofol | 0.776 | 0.695 |
| aminocaproic_acid | ursodeoxycholic_acid | 0.776 | 0.770 |
| ronidazole | scopolamine | 0.776 | 0.728 |
| propylthiouracil | trazodone | 0.776 | 0.689 |
| diltiazem | pyrazinamide | 0.776 | 0.789 |
| proxyphylline | tyloxapol | 0.776 | 0.881 |
| bepridil | oxetacaine | 0.776 | 0.749 |
| miconazole | rescinnamine | 0.776 | 0.778 |
| spectinomycin | theophylline | 0.776 | 0.763 |
| astemizole | fenoterol | 0.776 | 0.740 |
| iopanoic_acid | sulconazole | 0.776 | 0.694 |
| milrinone | progesterone | 0.776 | 0.710 |
| cyproterone | molindone | 0.776 | 0.718 |
| podophyllotoxin | terazosin | 0.776 | 0.733 |
| clomipramine | trioxysalen | 0.776 | 0.722 |
| iopanoic_acid | pentetrazol | 0.776 | 0.686 |
| metergoline | sertaconazole | 0.776 | 0.744 |
| menadione | piribedil | 0.776 | 0.739 |
| clobetasol | deptropine | 0.776 | 0.758 |
| flufenamic_acid | levonorgestrel | 0.776 | 0.726 |
| imipenem | tranylcypromine | 0.776 | 0.736 |
| metacycline | pyrazinamide | 0.776 | 0.738 |

| | | | |
|---|---|---|---|
| fluvoxamine | protriptyline | 0.776 | 0.755 |
| syrosingopine | zuclopenthixol | 0.776 | 0.766 |
| liothyronine | trazodone | 0.776 | 0.708 |
| rimexolone | sulfaphenazole | 0.776 | 0.746 |
| procainamide | tyloxapol | 0.776 | 0.872 |
| deptropine | oxamniquine | 0.776 | 0.779 |
| pentoxyverine | sulfamethoxazole | 0.776 | 0.704 |
| propafenone | trifluoperazine | 0.776 | 0.694 |
| medrysone | nitrofurantoin | 0.776 | 0.714 |
| ioversol | zidovudine | 0.776 | 0.772 |
| ketoprofen | sulconazole | 0.776 | 0.729 |
| nortriptyline | pimozide | 0.776 | 0.778 |
| dipyridamole | reserpine | 0.776 | 0.763 |
| diltiazem | nialamide | 0.776 | 0.749 |
| chlortetracycline | gefitinib | 0.776 | 0.756 |
| (-)-catechin | tyloxapol | 0.776 | 0.868 |
| budesonide | ketanserin | 0.776 | 0.739 |
| glibenclamide | progesterone | 0.776 | 0.783 |
| ribostamycin | tetryzoline | 0.776 | 0.816 |
| levodopa | piribedil | 0.776 | 0.696 |
| protriptyline | sulconazole | 0.776 | 0.659 |
| dydrogesterone | ketanserin | 0.776 | 0.741 |
| meclozine | oxybuprocaine | 0.776 | 0.721 |
| phenoxybenzamine | propafenone | 0.776 | 0.730 |
| diltiazem | tribenoside | 0.776 | 0.722 |
| clotrimazole | disulfiram | 0.776 | 0.736 |
| diltiazem | sulfaguanidine | 0.776 | 0.739 |
| gliclazide | liothyronine | 0.777 | 0.730 |
| cetirizine | trioxysalen | 0.777 | 0.799 |
| latamoxef | metoprolol | 0.777 | 0.771 |
| benfluorex | terfenadine | 0.777 | 0.678 |
| dihydroergotamine | spironolactone | 0.777 | 0.784 |
| roxithromycin | urapidil | 0.777 | 0.824 |
| chloramphenicol | rolitetracycline | 0.777 | 0.740 |
| chlorcyclizine | fluvoxamine | 0.777 | 0.759 |
| benperidol | propylthiouracil | 0.777 | 0.744 |
| lomustine | trimipramine | 0.777 | 0.772 |
| acepromazine | menadione | 0.777 | 0.717 |
| probenecid | tobramycin | 0.777 | 0.779 |
| etofenamate | ifenprodil | 0.777 | 0.711 |
| cefsulodin | theophylline | 0.777 | 0.823 |
| nabumetone | tribenoside | 0.777 | 0.770 |
| fluvoxamine | promazine | 0.777 | 0.715 |
| fipexide | reserpine | 0.777 | 0.714 |
| etidronic_acid | trazodone | 0.777 | 0.816 |
| alprenolol | piribedil | 0.777 | 0.697 |
| doxazosin | ketoprofen | 0.777 | 0.721 |
| ciclosporin | zuclopenthixol | 0.777 | 0.849 |
| clomipramine | dipyridamole | 0.777 | 0.756 |
| dosulepin | molsidomine | 0.777 | 0.772 |

| | | | |
|---|---|---|---|
| dilazep | trifluoperazine | 0.777 | 0.781 |
| meticrane | oxaprozin | 0.777 | 0.722 |
| latamoxef | mefloquine | 0.777 | 0.749 |
| chlorprothixene | fluvoxamine | 0.777 | 0.723 |
| promazine | verapamil | 0.777 | 0.777 |
| pyrvinium | sulfametoxydiazine | 0.777 | 0.781 |
| benzocaine | mebhydrolin | 0.777 | 0.739 |
| papaverine | propylthiouracil | 0.777 | 0.723 |
| clonidine | sulconazole | 0.777 | 0.745 |
| deferoxamine | gliclazide | 0.777 | 0.810 |
| cycloserine | meropenem | 0.777 | 0.854 |
| biotin | bromopride | 0.777 | 0.746 |
| carbachol | zidovudine | 0.777 | 0.732 |
| bepridil | fluvoxamine | 0.777 | 0.774 |
| dilazep | mebendazole | 0.777 | 0.773 |
| acepromazine | imipenem | 0.777 | 0.737 |
| betaxolol | natamycin | 0.777 | 0.798 |
| carbachol | levamisole | 0.777 | 0.701 |
| ambroxol | topiramate | 0.777 | 0.769 |
| doxorubicin | zomepirac | 0.777 | 0.692 |
| isradipine | remoxipride | 0.777 | 0.723 |
| ronidazole | tropicamide | 0.777 | 0.706 |
| dipyridamole | triflusal | 0.777 | 0.790 |
| carbachol | urapidil | 0.777 | 0.790 |
| oxprenolol | propylthiouracil | 0.777 | 0.717 |
| dextromethorphan | roxithromycin | 0.777 | 0.864 |
| felodipine | phenylpropanolamine | 0.777 | 0.741 |
| betazole | procaine | 0.777 | 0.713 |
| omeprazole | pipemidic_acid | 0.777 | 0.660 |
| bromperidol | terfenadine | 0.777 | 0.682 |
| sulfathiazole | tiapride | 0.777 | 0.706 |
| budesonide | cycloserine | 0.777 | 0.858 |
| bromocriptine | niclosamide | 0.777 | 0.743 |
| bromocriptine | exemestane | 0.777 | 0.767 |
| dextromethorphan | tranylcypromine | 0.777 | 0.692 |
| (-)-atenolol | flucytosine | 0.777 | 0.761 |
| lymecycline | piribedil | 0.777 | 0.763 |
| cefsulodin | trichlormethiazide | 0.777 | 0.744 |
| flufenamic_acid | oxetacaine | 0.777 | 0.768 |
| clobetasol | trioxysalen | 0.777 | 0.770 |
| liothyronine | proscillaridin | 0.777 | 0.747 |
| ethotoin | phenoxybenzamine | 0.777 | 0.737 |
| acetylsalicylic_acid | cyproterone | 0.777 | 0.763 |
| meticrane | pyrazinamide | 0.777 | 0.700 |
| fasudil | sodium_phenylbutyrat | 0.777 | 0.674 |
| pivampicillin | sulfamethoxazole | 0.777 | 0.739 |
| piribedil | pivampicillin | 0.777 | 0.717 |
| imipramine | mefloquine | 0.777 | 0.724 |
| flurbiprofen | nicotinic_acid | 0.777 | 0.679 |
| clioquinol | mometasone | 0.777 | 0.790 |

| | | | |
|---|---|---|---|
| astemizole | lynestrenol | 0.777 | 0.781 |
| cycloserine | fenoprofen | 0.777 | 0.789 |
| pyrazinamide | repaglinide | 0.777 | 0.761 |
| daunorubicin | etidronic_acid | 0.777 | 0.825 |
| etamsylate | hydrocortisone | 0.777 | 0.798 |
| fusidic_acid | topiramate | 0.777 | 0.769 |
| metacycline | sotalol | 0.777 | 0.737 |
| biperiden | nitrofural | 0.777 | 0.783 |
| etidronic_acid | sparteine | 0.777 | 0.791 |
| budesonide | simvastatin | 0.777 | 0.723 |
| ethotoin | paroxetine | 0.777 | 0.746 |
| ioxaglic_acid | talampicillin | 0.777 | 0.759 |
| chlorprothixene | propofol | 0.777 | 0.730 |
| doxazosin | fipexide | 0.777 | 0.671 |
| mephentermine | nalidixic_acid | 0.777 | 0.718 |
| etomidate | vidarabine | 0.777 | 0.683 |
| lomustine | trifluridine | 0.777 | 0.694 |
| estriol | sertaconazole | 0.777 | 0.740 |
| fendiline | lomustine | 0.777 | 0.740 |
| flumetasone | roxithromycin | 0.777 | 0.796 |
| milrinone | piromidic_acid | 0.777 | 0.651 |
| cetirizine | tropicamide | 0.777 | 0.731 |
| cetirizine | methylergometrine | 0.777 | 0.706 |
| pargyline | piperidolate | 0.777 | 0.704 |
| azacitidine | perhexiline | 0.777 | 0.782 |
| mepyramine | mifepristone | 0.777 | 0.732 |
| galantamine | isosorbide | 0.777 | 0.741 |
| cefalotin | metoprolol | 0.777 | 0.709 |
| mesoridazine | oxprenolol | 0.777 | 0.693 |
| chloramphenicol | theobromine | 0.777 | 0.736 |
| bisacodyl | bromperidol | 0.777 | 0.752 |
| clomifene | trimipramine | 0.777 | 0.722 |
| betahistine | roxithromycin | 0.777 | 0.898 |
| dicycloverine | metergoline | 0.777 | 0.711 |
| prilocaine | sulconazole | 0.777 | 0.732 |
| alprostadil | zuclopenthixol | 0.777 | 0.753 |
| nitrofurantoin | zomepirac | 0.777 | 0.669 |
| diltiazem | miconazole | 0.777 | 0.739 |
| carbachol | etomidate | 0.777 | 0.724 |
| cetirizine | ifenprodil | 0.777 | 0.676 |
| diltiazem | trioxysalen | 0.777 | 0.749 |
| loperamide | loxapine | 0.777 | 0.734 |
| bisacodyl | nitrofurantoin | 0.777 | 0.743 |
| clomifene | quinisocaine | 0.777 | 0.714 |
| natamycin | trioxysalen | 0.777 | 0.858 |
| metamizole_sodium | rolitetracycline | 0.777 | 0.727 |
| cefalexin | ethosuximide | 0.777 | 0.784 |
| cefalexin | metyrapone | 0.777 | 0.687 |
| gliclazide | ursodeoxycholic_acid | 0.777 | 0.739 |
| cycloserine | trazodone | 0.777 | 0.813 |

| | | | |
|---|---|---|---|
| benfotiamine | hydralazine | 0.777 | 0.781 |
| azlocillin | zomepirac | 0.777 | 0.732 |
| apomorphine | miconazole | 0.777 | 0.754 |
| suloctidil | tamoxifen | 0.777 | 0.744 |
| hydroxyzine | perphenazine | 0.777 | 0.653 |
| aztreonam | hyoscyamine | 0.777 | 0.739 |
| metyrapone | vidarabine | 0.777 | 0.686 |
| etoposide | metixene | 0.777 | 0.782 |
| irinotecan | mercaptopurine | 0.777 | 0.754 |
| mitoxantrone | triflusal | 0.777 | 0.770 |
| bisacodyl | remoxipride | 0.777 | 0.755 |
| remoxipride | tolfenamic_acid | 0.777 | 0.676 |
| cinchocaine | milrinone | 0.777 | 0.736 |
| calcium_pantothenate | estriol | 0.777 | 0.800 |
| norfloxacin | tranylcypromine | 0.777 | 0.736 |
| sulconazole | troleandomycin | 0.777 | 0.830 |
| ambroxol | nalidixic_acid | 0.777 | 0.687 |
| calcium_pantothenate | omeprazole | 0.777 | 0.760 |
| phenazopyridine | trazodone | 0.777 | 0.689 |
| nicotinic_acid | pentetic_acid | 0.777 | 0.825 |
| cetirizine | ranitidine | 0.777 | 0.743 |
| scopolamine | sulfamethoxypyridazir | 0.777 | 0.657 |
| cetirizine | phenacetin | 0.777 | 0.770 |
| acetylsalicylic_acid | hydrocortisone | 0.777 | 0.773 |
| dienestrol | econazole | 0.777 | 0.722 |
| scopolamine | trazodone | 0.777 | 0.727 |
| benzylpenicillin | bepridil | 0.777 | 0.750 |
| clomifene | clotrimazole | 0.777 | 0.745 |
| flunisolide | liothyronine | 0.777 | 0.722 |
| flufenamic_acid | halcinonide | 0.777 | 0.750 |
| cinchocaine | tolfenamic_acid | 0.777 | 0.688 |
| fluorometholone | talampicillin | 0.777 | 0.696 |
| meclofenoxate | phenoxybenzamine | 0.777 | 0.693 |
| estriol | menadione | 0.777 | 0.750 |
| deptropine | scopolamine | 0.777 | 0.746 |
| practolol | promethazine | 0.777 | 0.751 |
| ifenprodil | promethazine | 0.777 | 0.774 |
| cycloserine | urapidil | 0.777 | 0.849 |
| cefixime | sulfafurazole | 0.777 | 0.723 |
| pentoxyverine | ronidazole | 0.777 | 0.767 |
| dacarbazine | pimozide | 0.777 | 0.784 |
| clomipramine | daunorubicin | 0.777 | 0.762 |
| cetirizine | doxorubicin | 0.777 | 0.762 |
| medrysone | tyloxapol | 0.777 | 0.854 |
| meticrane | propafenone | 0.777 | 0.714 |
| bisacodyl | sulfafurazole | 0.777 | 0.741 |
| griseofulvin | salbutamol | 0.777 | 0.714 |
| hexetidine | ivermectin | 0.777 | 0.836 |
| estrone | lynestrenol | 0.777 | 0.680 |
| levonorgestrel | sulfametoxydiazine | 0.777 | 0.752 |

| | | | |
|---|---|---|---|
| bepridil | ticlopidine | 0.777 | 0.707 |
| iopanoic_acid | promazine | 0.777 | 0.684 |
| terguride | verteporfin | 0.777 | 0.801 |
| benfluorex | meptazinol | 0.778 | 0.755 |
| nomifensine | triflusal | 0.778 | 0.742 |
| meptazinol | sulconazole | 0.778 | 0.729 |
| amiodarone | fluocinonide | 0.778 | 0.771 |
| papaverine | triflusal | 0.778 | 0.762 |
| dipyridamole | promethazine | 0.778 | 0.746 |
| sulfafurazole | triflusal | 0.778 | 0.704 |
| benzylpenicillin | mesalazine | 0.778 | 0.725 |
| amoxicillin | sulfametoxydiazine | 0.778 | 0.651 |
| cetirizine | oxamniquine | 0.778 | 0.738 |
| acetylsalicylic_acid | sulfaphenazole | 0.778 | 0.697 |
| cetirizine | metacycline | 0.778 | 0.757 |
| pentoxyverine | xylometazoline | 0.778 | 0.751 |
| dextromethorphan | metamizole_sodium | 0.778 | 0.752 |
| cefalotin | scopolamine | 0.778 | 0.719 |
| benzathine_benzylpenicillin | zuclopenthixol | 0.778 | 0.755 |
| mefloquine | nalbuphine | 0.778 | 0.704 |
| cyproterone | sulfametoxydiazine | 0.778 | 0.746 |
| (-)-atenolol | bromopride | 0.778 | 0.691 |
| cinchocaine | natamycin | 0.778 | 0.809 |
| demecolcine | omeprazole | 0.778 | 0.740 |
| dacarbazine | procaine | 0.778 | 0.658 |
| isoetarine | pridinol | 0.778 | 0.669 |
| menadione | pivampicillin | 0.778 | 0.801 |
| medrysone | mesoridazine | 0.778 | 0.734 |
| acepromazine | daunorubicin | 0.778 | 0.688 |
| glibenclamide | trazodone | 0.778 | 0.694 |
| risperidone | talampicillin | 0.778 | 0.721 |
| pentoxyverine | testosterone | 0.778 | 0.782 |
| roxithromycin | sulpiride | 0.778 | 0.809 |
| letrozole | spiramycin | 0.778 | 0.836 |
| galantamine | pyrazinamide | 0.778 | 0.760 |
| bisacodyl | urapidil | 0.778 | 0.762 |
| clozapine | haloperidol | 0.778 | 0.752 |
| tetryzoline | tribenoside | 0.778 | 0.798 |
| estriol | vinpocetine | 0.778 | 0.737 |
| benzylpenicillin | sulfametoxydiazine | 0.778 | 0.684 |
| hexetidine | prenylamine | 0.778 | 0.717 |
| apomorphine | levonorgestrel | 0.778 | 0.696 |
| hyoscyamine | meticrane | 0.778 | 0.697 |
| chlorphenesin | ethosuximide | 0.778 | 0.727 |
| mefloquine | promethazine | 0.778 | 0.704 |
| pentoxyverine | tropicamide | 0.778 | 0.727 |
| ioversol | trazodone | 0.778 | 0.800 |
| dextromethorphan | gliclazide | 0.778 | 0.781 |
| ondansetron | tropine | 0.778 | 0.781 |
| etofylline | tyloxapol | 0.778 | 0.883 |

| | | | |
|---|---|---|---|
| capsaicin | repaglinide | 0.778 | 0.726 |
| meptazinol | rifabutin | 0.778 | 0.854 |
| naloxone | omeprazole | 0.778 | 0.753 |
| acetylsalicylic_acid | piperidolate | 0.778 | 0.733 |
| disulfiram | fenbendazole | 0.778 | 0.718 |
| buflomedil | promethazine | 0.778 | 0.719 |
| mebendazole | tetryzoline | 0.778 | 0.724 |
| benzylpenicillin | daunorubicin | 0.778 | 0.694 |
| monobenzone | piperidolate | 0.778 | 0.720 |
| fendiline | thioridazine | 0.778 | 0.723 |
| progesterone | repaglinide | 0.778 | 0.738 |
| metergoline | semustine | 0.778 | 0.725 |
| lomefloxacin | phenformin | 0.778 | 0.658 |
| atovaquone | cinchocaine | 0.778 | 0.669 |
| deptropine | dydrogesterone | 0.778 | 0.761 |
| perhexiline | zuclopenthixol | 0.778 | 0.743 |
| diltiazem | meticrane | 0.778 | 0.746 |
| hydralazine | sulfamethoxypyridazir | 0.778 | 0.701 |
| amiodarone | fluspirilene | 0.778 | 0.749 |
| mesoridazine | metacycline | 0.778 | 0.704 |
| clonidine | latamoxef | 0.778 | 0.813 |
| econazole | erastin | 0.778 | 0.767 |
| carbachol | piperidolate | 0.778 | 0.761 |
| pyrantel | sulconazole | 0.778 | 0.734 |
| betahistine | carbachol | 0.778 | 0.674 |
| haloperidol | thioridazine | 0.778 | 0.746 |
| fenoterol | gliclazide | 0.778 | 0.674 |
| galantamine | selegiline | 0.778 | 0.751 |
| dacarbazine | thiocolchicoside | 0.778 | 0.788 |
| piroxicam | ranitidine | 0.778 | 0.738 |
| diclofenamide | naftidrofuryl | 0.778 | 0.746 |
| imipenem | trimetazidine | 0.778 | 0.715 |
| diclofenac | oxprenolol | 0.778 | 0.677 |
| clomipramine | doxazosin | 0.778 | 0.789 |
| mebendazole | sulfamethoxazole | 0.778 | 0.682 |
| dydrogesterone | verteporfin | 0.778 | 0.814 |
| estrone | oxetacaine | 0.778 | 0.786 |
| clindamycin | hyoscyamine | 0.778 | 0.725 |
| aminohippuric_acid | pargyline | 0.778 | 0.673 |
| atovaquone | repaglinide | 0.778 | 0.739 |
| alprenolol | xylometazoline | 0.778 | 0.704 |
| glafenine | triprolidine | 0.778 | 0.710 |
| prochlorperazine | trihexyphenidyl | 0.778 | 0.683 |
| labetalol | mepacrine | 0.778 | 0.718 |
| bupropion | papaverine | 0.778 | 0.700 |
| buflomedil | xylometazoline | 0.778 | 0.722 |
| dipyridamole | simvastatin | 0.778 | 0.733 |
| methylergometrine | pivampicillin | 0.778 | 0.738 |
| cycloserine | mitoxantrone | 0.778 | 0.886 |
| cetirizine | molindone | 0.778 | 0.732 |

| | | | |
|---|---|---|---|
| clindamycin | trazodone | 0.778 | 0.721 |
| chlorcyclizine | econazole | 0.778 | 0.679 |
| diflorasone | rescinnamine | 0.778 | 0.765 |
| benzathine_benzylpenicillin | nadolol | 0.778 | 0.689 |
| oxybuprocaine | sulfametoxydiazine | 0.778 | 0.679 |
| griseofulvin | meticrane | 0.778 | 0.712 |
| meticrane | propofol | 0.778 | 0.682 |
| methapyrilene | natamycin | 0.778 | 0.840 |
| sulfafurazole | tropicamide | 0.778 | 0.676 |
| clemastine | pimethixene | 0.778 | 0.692 |
| diclofenac | tyloxapol | 0.778 | 0.867 |
| levamisole | vinpocetine | 0.778 | 0.707 |
| ethotoin | verteporfin | 0.778 | 0.841 |
| daunorubicin | zimeldine | 0.778 | 0.738 |
| perphenazine | terconazole | 0.778 | 0.745 |
| iopanoic_acid | liothyronine | 0.778 | 0.683 |
| etomidate | propafenone | 0.778 | 0.725 |
| monobenzone | moroxydine | 0.778 | 0.705 |
| dicoumarol | pyrantel | 0.778 | 0.722 |
| isoconazole | syrosingopine | 0.778 | 0.800 |
| spironolactone | thioproperazine | 0.778 | 0.733 |
| diltiazem | roxithromycin | 0.778 | 0.798 |
| amiodarone | trazodone | 0.778 | 0.718 |
| benzethonium_chloride | tribenoside | 0.778 | 0.752 |
| glibenclamide | medrysone | 0.778 | 0.772 |
| azapropazone | disopyramide | 0.778 | 0.718 |
| flurbiprofen | tyloxapol | 0.778 | 0.871 |
| flucytosine | rolitetracycline | 0.778 | 0.830 |
| labetalol | methylprednisolone | 0.778 | 0.733 |
| dextromethorphan | nitrendipine | 0.778 | 0.728 |
| cyproterone | trazodone | 0.778 | 0.762 |
| bromperidol | cinchocaine | 0.778 | 0.739 |
| ketoconazole | procainamide | 0.778 | 0.764 |
| flufenamic_acid | natamycin | 0.778 | 0.815 |
| glibenclamide | verteporfin | 0.778 | 0.772 |
| chlorprothixene | dienestrol | 0.778 | 0.731 |
| clomipramine | pentoxyverine | 0.778 | 0.725 |
| acepromazine | glibenclamide | 0.778 | 0.741 |
| daunorubicin | sulfamethoxypyridazir | 0.778 | 0.727 |
| hydroflumethiazide | moxisylyte | 0.778 | 0.714 |
| liothyronine | sulfaphenazole | 0.778 | 0.687 |
| ketanserin | mitoxantrone | 0.778 | 0.704 |
| chlorcyclizine | fluspirilene | 0.778 | 0.736 |
| mercaptopurine | syrosingopine | 0.778 | 0.816 |
| rolitetracycline | talampicillin | 0.778 | 0.679 |
| chloramphenicol | reserpine | 0.778 | 0.767 |
| acetylsalicylic_acid | molindone | 0.778 | 0.738 |
| carbenoxolone | trimipramine | 0.778 | 0.822 |
| ethosuximide | trimetazidine | 0.778 | 0.782 |
| methazolamide | monobenzone | 0.778 | 0.717 |

| | | | |
|---|---|---|---|
| amikacin | monobenzone | 0.778 | 0.856 |
| suloctidil | verteporfin | 0.778 | 0.812 |
| miconazole | pimozide | 0.778 | 0.744 |
| daunorubicin | mesoridazine | 0.778 | 0.663 |
| acepromazine | dydrogesterone | 0.778 | 0.722 |
| dicoumarol | flunisolide | 0.778 | 0.703 |
| bromocriptine | raloxifene | 0.778 | 0.749 |
| hyoscyamine | natamycin | 0.778 | 0.817 |
| sulfamethoxypyridazine | ursodeoxycholic_acid | 0.778 | 0.746 |
| oxybuprocaine | piribedil | 0.778 | 0.708 |
| levonorgestrel | pyrvinium | 0.778 | 0.793 |
| oxolinic_acid | pargyline | 0.778 | 0.745 |
| fenbendazole | paclitaxel | 0.778 | 0.826 |
| famotidine | progesterone | 0.778 | 0.739 |
| liothyronine | lymecycline | 0.778 | 0.767 |
| capsaicin | cetirizine | 0.778 | 0.741 |
| hydralazine | metamizole_sodium | 0.778 | 0.689 |
| ornidazole | pargyline | 0.778 | 0.680 |
| meticrane | urapidil | 0.778 | 0.774 |
| bromocriptine | protriptyline | 0.779 | 0.777 |
| clobetasol | doxazosin | 0.779 | 0.757 |
| ketoprofen | repaglinide | 0.779 | 0.736 |
| pivampicillin | repaglinide | 0.779 | 0.745 |
| estriol | proxyphylline | 0.779 | 0.680 |
| azacitidine | glibenclamide | 0.779 | 0.794 |
| fendiline | oxyphenbutazone | 0.779 | 0.730 |
| deptropine | fluspirilene | 0.779 | 0.758 |
| naloxone | promethazine | 0.779 | 0.707 |
| levamisole | terguride | 0.779 | 0.739 |
| nifurtimox | sulconazole | 0.779 | 0.739 |
| clindamycin | ivermectin | 0.779 | 0.805 |
| flufenamic_acid | hyoscyamine | 0.779 | 0.659 |
| milrinone | piperidolate | 0.779 | 0.771 |
| amoxicillin | ganciclovir | 0.779 | 0.702 |
| atovaquone | azacitidine | 0.779 | 0.724 |
| doxorubicin | ioversol | 0.779 | 0.708 |
| cisapride | meclozine | 0.779 | 0.752 |
| clidinium_bromide | metoprolol | 0.779 | 0.713 |
| budesonide | prenylamine | 0.779 | 0.778 |
| bisacodyl | fluspirilene | 0.779 | 0.731 |
| clioquinol | fenbendazole | 0.779 | 0.700 |
| perphenazine | podophyllotoxin | 0.779 | 0.719 |
| albendazole | demecolcine | 0.779 | 0.700 |
| bepridil | flufenamic_acid | 0.779 | 0.758 |
| cinchocaine | liothyronine | 0.779 | 0.718 |
| etamsylate | tolfenamic_acid | 0.779 | 0.674 |
| ketanserin | tetryzoline | 0.779 | 0.766 |
| roxithromycin | trichlormethiazide | 0.779 | 0.832 |
| dacarbazine | sulfadimidine | 0.779 | 0.688 |
| etamsylate | imipenem | 0.779 | 0.726 |

| | | | |
|---|---|---|---|
| cefsulodin | doxorubicin | 0.779 | 0.727 |
| bepridil | nicergoline | 0.779 | 0.739 |
| doxazosin | nialamide | 0.779 | 0.659 |
| propafenone | rolitetracycline | 0.779 | 0.740 |
| pivampicillin | trazodone | 0.779 | 0.704 |
| captopril | cimetidine | 0.779 | 0.721 |
| sulfadimidine | tobramycin | 0.779 | 0.785 |
| nitrendipine | tyloxapol | 0.779 | 0.851 |
| liothyronine | ronidazole | 0.779 | 0.713 |
| flunarizine | galantamine | 0.779 | 0.741 |
| alfaxalone | propafenone | 0.779 | 0.740 |
| apomorphine | mitoxantrone | 0.779 | 0.696 |
| etamsylate | ipratropium_bromide | 0.779 | 0.709 |
| betaxolol | meclozine | 0.779 | 0.753 |
| quinethazone | tyloxapol | 0.779 | 0.871 |
| amoxapine | apomorphine | 0.779 | 0.710 |
| chlorcyclizine | zuclopenthixol | 0.779 | 0.704 |
| cycloserine | dextromethorphan | 0.779 | 0.882 |
| hydrocortisone | procaine | 0.779 | 0.747 |
| flupentixol | mebendazole | 0.779 | 0.689 |
| cefixime | rimexolone | 0.779 | 0.749 |
| cyproheptadine | oxamic_acid | 0.779 | 0.895 |
| maprotiline | pimozide | 0.779 | 0.763 |
| bisacodyl | labetalol | 0.779 | 0.745 |
| ganciclovir | trichlormethiazide | 0.779 | 0.676 |
| azacitidine | rimexolone | 0.779 | 0.770 |
| benfluorex | buflomedil | 0.779 | 0.697 |
| hydrocortisone | roxithromycin | 0.779 | 0.791 |
| etamsylate | fipexide | 0.779 | 0.788 |
| ketanserin | loperamide | 0.779 | 0.680 |
| bromocriptine | medrysone | 0.779 | 0.764 |
| calcium_pantothenate | probenecid | 0.779 | 0.794 |
| betaxolol | clindamycin | 0.779 | 0.758 |
| mercaptopurine | mycophenolic_acid | 0.779 | 0.675 |
| pyrantel | rimexolone | 0.779 | 0.781 |
| deptropine | levonorgestrel | 0.779 | 0.751 |
| (-)-atenolol | sulconazole | 0.779 | 0.781 |
| ioversol | mitoxantrone | 0.779 | 0.727 |
| meropenem | terguride | 0.779 | 0.754 |
| nicergoline | perphenazine | 0.779 | 0.704 |
| bromperidol | tranylcypromine | 0.779 | 0.748 |
| flunisolide | ursodeoxycholic_acid | 0.779 | 0.704 |
| diltiazem | levonorgestrel | 0.779 | 0.752 |
| imipramine | mebendazole | 0.779 | 0.737 |
| methoxamine | practolol | 0.779 | 0.706 |
| aminohippuric_acid | cefalotin | 0.779 | 0.740 |
| amikacin | phenazone | 0.779 | 0.866 |
| alimemazine | milrinone | 0.779 | 0.705 |
| flupentixol | prenylamine | 0.779 | 0.752 |
| betahistine | nilutamide | 0.779 | 0.694 |

| | | | |
|---|---|---|---|
| daunorubicin | estriol | 0.779 | 0.724 |
| methylergometrine | trifluoperazine | 0.779 | 0.657 |
| piromidic_acid | sulfametoxydiazine | 0.779 | 0.671 |
| digoxin | thioridazine | 0.779 | 0.858 |
| amikacin | diltiazem | 0.779 | 0.779 |
| disulfiram | loperamide | 0.779 | 0.754 |
| bezafibrate | nitrofurantoin | 0.779 | 0.708 |
| digoxin | mometasone | 0.779 | 0.781 |
| bromperidol | sulfamethoxypyridazir | 0.779 | 0.706 |
| lynestrenol | naloxone | 0.779 | 0.728 |
| chloramphenicol | repaglinide | 0.779 | 0.719 |
| thioproperazine | vorinostat | 0.779 | 0.746 |
| ethotoin | nialamide | 0.779 | 0.700 |
| acepromazine | exemestane | 0.779 | 0.689 |
| etilefrine | molindone | 0.779 | 0.687 |
| fipexide | guanethidine | 0.779 | 0.733 |
| meclozine | metacycline | 0.779 | 0.754 |
| probenecid | reserpine | 0.779 | 0.760 |
| perhexiline | primaquine | 0.779 | 0.733 |
| ivermectin | nicergoline | 0.779 | 0.807 |
| metamizole_sodium | pentoxyverine | 0.779 | 0.705 |
| fluspirilene | rescinnamine | 0.779 | 0.764 |
| ketanserin | meclofenoxate | 0.779 | 0.690 |
| flufenamic_acid | methazolamide | 0.779 | 0.668 |
| daunorubicin | triflusal | 0.779 | 0.766 |
| profenamine | urapidil | 0.779 | 0.756 |
| famotidine | tioguanine | 0.779 | 0.734 |
| chlorambucil | meticrane | 0.779 | 0.747 |
| cefsulodin | selegiline | 0.779 | 0.788 |
| bromopride | phenformin | 0.779 | 0.684 |
| gliclazide | spectinomycin | 0.779 | 0.712 |
| hydralazine | scopolamine | 0.779 | 0.717 |
| rifampicin | suprofen | 0.779 | 0.828 |
| latamoxef | metyrapone | 0.779 | 0.769 |
| clioquinol | etoposide | 0.779 | 0.799 |
| isoxsuprine | reserpine | 0.779 | 0.736 |
| naloxone | tropicamide | 0.779 | 0.686 |
| mexiletine | proglumide | 0.779 | 0.719 |
| capsaicin | gliclazide | 0.779 | 0.698 |
| imipenem | procaine | 0.779 | 0.694 |
| amoxapine | fendiline | 0.779 | 0.729 |
| benzonatate | sulfacetamide | 0.779 | 0.784 |
| daunorubicin | omeprazole | 0.779 | 0.692 |
| cetirizine | chlorzoxazone | 0.779 | 0.773 |
| benzylpenicillin | medrysone | 0.779 | 0.711 |
| lansoprazole | risperidone | 0.779 | 0.685 |
| cetirizine | sulfamethoxazole | 0.779 | 0.744 |
| salbutamol | urapidil | 0.779 | 0.730 |
| clonidine | methylergometrine | 0.779 | 0.738 |
| cortisone | dipyridamole | 0.779 | 0.744 |

| | | | |
|---|---|---|---|
| betaxolol | nicardipine | 0.779 | 0.748 |
| rifampicin | sisomicin | 0.779 | 0.786 |
| acepromazine | metyrapone | 0.779 | 0.696 |
| mesalazine | metoprolol | 0.779 | 0.717 |
| chloramphenicol | galantamine | 0.779 | 0.713 |
| chlorphenesin | ranitidine | 0.779 | 0.731 |
| deptropine | trazodone | 0.779 | 0.778 |
| (-)-catechin | levamisole | 0.779 | 0.677 |
| artemisinin | thiocolchicoside | 0.779 | 0.820 |
| fenoprofen | pivampicillin | 0.779 | 0.743 |
| mebendazole | perphenazine | 0.779 | 0.706 |
| dipyridamole | sulfamethoxypyridazir | 0.779 | 0.761 |
| fenoprofen | methapyrilene | 0.779 | 0.685 |
| clotrimazole | phenazopyridine | 0.779 | 0.750 |
| etamivan | imipenem | 0.779 | 0.713 |
| imipenem | trioxysalen | 0.779 | 0.720 |
| (-)-atenolol | triflusal | 0.779 | 0.731 |
| benperidol | rimexolone | 0.779 | 0.749 |
| atovaquone | propylthiouracil | 0.779 | 0.651 |
| fenoprofen | nifurtimox | 0.779 | 0.702 |
| tiapride | trimethoprim | 0.779 | 0.727 |
| suloctidil | trimipramine | 0.779 | 0.770 |
| loperamide | pizotifen | 0.779 | 0.773 |
| amoxicillin | latamoxef | 0.779 | 0.711 |
| calcium_pantothenate | carisoprodol | 0.779 | 0.789 |
| ipratropium_bromide | procaine | 0.779 | 0.693 |
| calcium_pantothenate | liothyronine | 0.779 | 0.783 |
| metacycline | metyrapone | 0.779 | 0.714 |
| benfluorex | primaquine | 0.779 | 0.698 |
| cinchocaine | fluorometholone | 0.779 | 0.752 |
| flumetasone | sulconazole | 0.779 | 0.781 |
| cycloserine | tobramycin | 0.779 | 0.871 |
| buspirone | fusidic_acid | 0.779 | 0.729 |
| imipenem | tropicamide | 0.779 | 0.711 |
| cimetidine | idoxuridine | 0.779 | 0.707 |
| fludroxycortide | meptazinol | 0.779 | 0.789 |
| diethylcarbamazine | prednisolone | 0.779 | 0.751 |
| medrysone | methazolamide | 0.779 | 0.712 |
| demecolcine | sulconazole | 0.779 | 0.700 |
| bromperidol | roxithromycin | 0.779 | 0.819 |
| flucytosine | rimexolone | 0.779 | 0.787 |
| acetylsalicylic_acid | riluzole | 0.779 | 0.675 |
| flufenamic_acid | xylometazoline | 0.779 | 0.735 |
| aminophylline | gliclazide | 0.779 | 0.777 |
| etamsylate | reserpine | 0.779 | 0.825 |
| sulfamerazine | triflusal | 0.779 | 0.698 |
| cyclobenzaprine | fluoxetine | 0.779 | 0.721 |
| methazolamide | roxithromycin | 0.779 | 0.858 |
| kanamycin | practolol | 0.779 | 0.786 |
| griseofulvin | latamoxef | 0.779 | 0.758 |

| | | | |
|---|---|---|---|
| ipratropium_bromide | irinotecan | 0.779 | 0.792 |
| meropenem | trichlormethiazide | 0.779 | 0.697 |
| fenspiride | xylometazoline | 0.779 | 0.732 |
| pipemidic_acid | pivampicillin | 0.779 | 0.727 |
| aminohippuric_acid | iopanoic_acid | 0.779 | 0.659 |
| alprenolol | sulfapyridine | 0.780 | 0.663 |
| bezafibrate | chlorzoxazone | 0.780 | 0.739 |
| bromperidol | cyproterone | 0.780 | 0.741 |
| hydrocortisone | tyloxapol | 0.780 | 0.836 |
| cefalexin | flufenamic_acid | 0.780 | 0.692 |
| diclofenac | exemestane | 0.780 | 0.699 |
| metacycline | trioxysalen | 0.780 | 0.762 |
| amiloride | ethambutol | 0.780 | 0.713 |
| bromperidol | prenylamine | 0.780 | 0.719 |
| fluspirilene | metyrapone | 0.780 | 0.719 |
| mometasone | proscillaridin | 0.780 | 0.758 |
| ipratropium_bromide | menadione | 0.780 | 0.741 |
| liothyronine | natamycin | 0.780 | 0.783 |
| desoxycortone | flufenamic_acid | 0.780 | 0.731 |
| cinchocaine | etomidate | 0.780 | 0.709 |
| nomifensine | repaglinide | 0.780 | 0.792 |
| estriol | pargyline | 0.780 | 0.751 |
| etidronic_acid | spectinomycin | 0.780 | 0.749 |
| cyanocobalamin | ganciclovir | 0.780 | 0.830 |
| capsaicin | dipyridamole | 0.780 | 0.778 |
| estriol | promazine | 0.780 | 0.720 |
| alfaxalone | etamsylate | 0.780 | 0.788 |
| apomorphine | urapidil | 0.780 | 0.722 |
| estriol | hyoscyamine | 0.780 | 0.711 |
| chlorcyclizine | digoxin | 0.780 | 0.878 |
| chloroquine | imipenem | 0.780 | 0.734 |
| ciclosporin | lovastatin | 0.780 | 0.842 |
| metyrapone | promazine | 0.780 | 0.701 |
| ethambutol | etofenamate | 0.780 | 0.731 |
| metacycline | tobramycin | 0.780 | 0.744 |
| liothyronine | zomepirac | 0.780 | 0.655 |
| glycopyrronium_bromide | isocarboxazid | 0.780 | 0.654 |
| isoconazole | neomycin | 0.780 | 0.826 |
| flunisolide | sulfaphenazole | 0.780 | 0.727 |
| chloroquine | phenindione | 0.780 | 0.670 |
| desoxycortone | glibenclamide | 0.780 | 0.773 |
| benperidol | reserpine | 0.780 | 0.682 |
| mometasone | spiramycin | 0.780 | 0.807 |
| perphenazine | reserpine | 0.780 | 0.746 |
| diltiazem | piribedil | 0.780 | 0.728 |
| hydrocortisone | ipratropium_bromide | 0.780 | 0.708 |
| amoxicillin | etamsylate | 0.780 | 0.740 |
| calcium_pantothenate | natamycin | 0.780 | 0.785 |
| piromidic_acid | proscillaridin | 0.780 | 0.786 |
| proxyphylline | trioxysalen | 0.780 | 0.676 |

| | | | |
|---|---|---|---|
| iopanoic_acid | proscillaridin | 0.780 | 0.789 |
| ketoprofen | rimexolone | 0.780 | 0.698 |
| etoposide | syrosingopine | 0.780 | 0.728 |
| econazole | ticlopidine | 0.780 | 0.716 |
| clozapine | maprotiline | 0.780 | 0.710 |
| indapamide | promazine | 0.780 | 0.716 |
| isoconazole | suloctidil | 0.780 | 0.724 |
| progesterone | tyloxapol | 0.780 | 0.870 |
| diflorasone | galantamine | 0.780 | 0.762 |
| levocabastine | molindone | 0.780 | 0.744 |
| aminocaproic_acid | gliclazide | 0.780 | 0.772 |
| molindone | promazine | 0.780 | 0.703 |
| aminocaproic_acid | tolfenamic_acid | 0.780 | 0.719 |
| fluspirilene | terconazole | 0.780 | 0.747 |
| isotretinoin | semustine | 0.780 | 0.762 |
| proxymetacaine | sulfasalazine | 0.780 | 0.698 |
| fluvoxamine | sulfaphenazole | 0.780 | 0.740 |
| aminophylline | capsaicin | 0.780 | 0.721 |
| (-)-catechin | verteporfin | 0.780 | 0.809 |
| felodipine | menadione | 0.780 | 0.719 |
| penbutolol | proguanil | 0.780 | 0.683 |
| chlorphenamine | oxybuprocaine | 0.780 | 0.738 |
| procyclidine | propantheline_bromic | 0.780 | 0.669 |
| acepromazine | zimeldine | 0.780 | 0.676 |
| cisapride | progesterone | 0.780 | 0.768 |
| clonidine | etofylline | 0.780 | 0.678 |
| dydrogesterone | quinisocaine | 0.780 | 0.762 |
| doxorubicin | glipizide | 0.780 | 0.695 |
| propofol | propranolol | 0.780 | 0.705 |
| colchicine | deptropine | 0.780 | 0.734 |
| levomepromazine | vorinostat | 0.780 | 0.744 |
| levonorgestrel | metamizole_sodium | 0.780 | 0.722 |
| alfuzosin | chlorambucil | 0.780 | 0.716 |
| doxazosin | nicotinic_acid | 0.780 | 0.751 |
| niclosamide | rifabutin | 0.780 | 0.817 |
| mesoridazine | prenylamine | 0.780 | 0.733 |
| butoconazole | mifepristone | 0.780 | 0.718 |
| levamisole | pentetrazol | 0.780 | 0.725 |
| clomipramine | fluorometholone | 0.780 | 0.773 |
| estrone | phenoxybenzamine | 0.780 | 0.763 |
| gliclazide | ioversol | 0.780 | 0.783 |
| carbachol | fluorometholone | 0.780 | 0.786 |
| apomorphine | diltiazem | 0.780 | 0.760 |
| fluphenazine | oxetacaine | 0.780 | 0.749 |
| mifepristone | oxybutynin | 0.780 | 0.766 |
| prochlorperazine | quinisocaine | 0.780 | 0.672 |
| budesonide | omeprazole | 0.780 | 0.747 |
| bromocriptine | procarbazine | 0.780 | 0.801 |
| felodipine | fluvastatin | 0.780 | 0.762 |
| levonorgestrel | papaverine | 0.780 | 0.683 |

| | | | |
|---|---|---|---|
| pipemidic_acid | prasterone | 0.780 | 0.697 |
| salbutamol | tropicamide | 0.780 | 0.706 |
| oxamic_acid | sulconazole | 0.780 | 0.896 |
| cefotaxime | diazoxide | 0.780 | 0.781 |
| clomipramine | sulfinpyrazone | 0.780 | 0.739 |
| digoxin | isoconazole | 0.780 | 0.857 |
| dipyridamole | dydrogesterone | 0.780 | 0.765 |
| glibenclamide | meclozine | 0.780 | 0.770 |
| sulfaphenazole | trazodone | 0.780 | 0.697 |
| aciclovir | finasteride | 0.780 | 0.758 |
| etofenamate | probenecid | 0.780 | 0.724 |
| clozapine | mefloquine | 0.780 | 0.718 |
| acetylsalicylic_acid | roxithromycin | 0.780 | 0.875 |
| astemizole | fluoxetine | 0.780 | 0.752 |
| flufenamic_acid | ipratropium_bromide | 0.780 | 0.705 |
| fludroxycortide | promethazine | 0.780 | 0.777 |
| bromocriptine | butoconazole | 0.780 | 0.770 |
| fluvoxamine | zalcitabine | 0.780 | 0.690 |
| gefitinib | josamycin | 0.780 | 0.806 |
| propafenone | verteporfin | 0.780 | 0.770 |
| ketanserin | sulfapyridine | 0.780 | 0.722 |
| benfluorex | mefloquine | 0.780 | 0.698 |
| cefalotin | glibenclamide | 0.780 | 0.709 |
| etidronic_acid | medrysone | 0.780 | 0.812 |
| decitabine | rosiglitazone | 0.780 | 0.744 |
| desoxycortone | levamisole | 0.780 | 0.751 |
| clomifene | maprotiline | 0.780 | 0.731 |
| chlorzoxazone | piroxicam | 0.780 | 0.747 |
| norethisterone | oxprenolol | 0.780 | 0.779 |
| betaxolol | daunorubicin | 0.780 | 0.753 |
| propantheline_bromide | scopolamine | 0.780 | 0.664 |
| bethanechol | phensuximide | 0.780 | 0.686 |
| natamycin | omeprazole | 0.780 | 0.806 |
| ketanserin | rolitetracycline | 0.780 | 0.704 |
| lymecycline | triflusal | 0.780 | 0.812 |
| letrozole | molindone | 0.780 | 0.737 |
| aminohippuric_acid | amiodarone | 0.780 | 0.747 |
| mesoridazine | trioxysalen | 0.780 | 0.705 |
| etamsylate | ursodeoxycholic_acid | 0.780 | 0.823 |
| dirithromycin | omeprazole | 0.780 | 0.817 |
| deptropine | mebendazole | 0.780 | 0.760 |
| amiodarone | metyrapone | 0.780 | 0.758 |
| colecalciferol | metixene | 0.780 | 0.782 |
| clotrimazole | dipyridamole | 0.780 | 0.803 |
| cetirizine | imipenem | 0.780 | 0.769 |
| bromocriptine | isoetarine | 0.780 | 0.782 |
| clemastine | econazole | 0.780 | 0.672 |
| dipyridamole | etomidate | 0.780 | 0.779 |
| risperidone | spaglumic_acid | 0.780 | 0.759 |
| repaglinide | tolfenamic_acid | 0.780 | 0.724 |

| | | | |
|---|---|---|---|
| imipenem | piromidic_acid | 0.780 | 0.695 |
| cycloserine | ipratropium_bromide | 0.780 | 0.808 |
| halcinonide | promazine | 0.780 | 0.793 |
| cefixime | minaprine | 0.780 | 0.697 |
| propylthiouracil | sulfamerazine | 0.780 | 0.697 |
| ivermectin | pimozide | 0.780 | 0.826 |
| levonorgestrel | metyrapone | 0.780 | 0.721 |
| azacitidine | repaglinide | 0.780 | 0.785 |
| dextromethorphan | trazodone | 0.780 | 0.793 |
| bisacodyl | tolbutamide | 0.780 | 0.744 |
| liothyronine | sulfamerazine | 0.780 | 0.738 |
| bretylium_tosilate | sulconazole | 0.780 | 0.748 |
| procaine | trimetazidine | 0.780 | 0.700 |
| clomifene | latamoxef | 0.780 | 0.779 |
| doxepin | trazodone | 0.780 | 0.758 |
| carbachol | trichlormethiazide | 0.780 | 0.776 |
| hyoscyamine | promazine | 0.781 | 0.745 |
| monobenzone | rifabutin | 0.781 | 0.859 |
| lomustine | vorinostat | 0.781 | 0.685 |
| etidronic_acid | phenoxybenzamine | 0.781 | 0.832 |
| albendazole | clindamycin | 0.781 | 0.735 |
| roxithromycin | sulfamerazine | 0.781 | 0.853 |
| betahistine | betaxolol | 0.781 | 0.687 |
| imipenem | tyloxapol | 0.781 | 0.861 |
| acepromazine | mepyramine | 0.781 | 0.707 |
| rifampicin | trihexyphenidyl | 0.781 | 0.824 |
| colchicine | pimozide | 0.781 | 0.779 |
| acepromazine | monobenzone | 0.781 | 0.698 |
| levonorgestrel | ranitidine | 0.781 | 0.757 |
| etacrynic_acid | tamoxifen | 0.781 | 0.730 |
| doxorubicin | piperidolate | 0.781 | 0.773 |
| cortisone | zimeldine | 0.781 | 0.758 |
| mecamylamine | triprolidine | 0.781 | 0.825 |
| cefalotin | propafenone | 0.781 | 0.726 |
| colchicine | fluspirilene | 0.781 | 0.751 |
| astemizole | nomifensine | 0.781 | 0.768 |
| fluorometholone | sulfamethoxypyridazir | 0.781 | 0.713 |
| flufenamic_acid | nalidixic_acid | 0.781 | 0.666 |
| astemizole | clozapine | 0.781 | 0.734 |
| benzylpenicillin | metacycline | 0.781 | 0.680 |
| etamsylate | liothyronine | 0.781 | 0.761 |
| carbachol | propafenone | 0.781 | 0.754 |
| propantheline_bromide | zimeldine | 0.781 | 0.702 |
| nomifensine | pirenzepine | 0.781 | 0.699 |
| acetylsalicylic_acid | bufexamac | 0.781 | 0.695 |
| letrozole | sotalol | 0.781 | 0.739 |
| cyproterone | ifenprodil | 0.781 | 0.709 |
| ceforanide | lynestrenol | 0.781 | 0.804 |
| fluspirilene | tyloxapol | 0.781 | 0.863 |
| droperidol | oxantel | 0.781 | 0.704 |

| | | | |
|---|---|---|---|
| mesoridazine | trazodone | 0.781 | 0.743 |
| clomipramine | flupentixol | 0.781 | 0.683 |
| meptazinol | pipemidic_acid | 0.781 | 0.737 |
| menadione | progesterone | 0.781 | 0.786 |
| acetylsalicylic_acid | spectinomycin | 0.781 | 0.730 |
| doxorubicin | flunisolide | 0.781 | 0.728 |
| etoposide | triamterene | 0.781 | 0.774 |
| lymecycline | menadione | 0.781 | 0.804 |
| disulfiram | dyclonine | 0.781 | 0.684 |
| azacitidine | omeprazole | 0.781 | 0.695 |
| mefloquine | zuclopenthixol | 0.781 | 0.747 |
| doxorubicin | guaifenesin | 0.781 | 0.705 |
| cetirizine | cinchocaine | 0.781 | 0.741 |
| bupropion | doxazosin | 0.781 | 0.744 |
| chlorambucil | etidronic_acid | 0.781 | 0.813 |
| benzocaine | felbinac | 0.781 | 0.671 |
| clotrimazole | nortriptyline | 0.781 | 0.666 |
| bezafibrate | trioxysalen | 0.781 | 0.750 |
| liothyronine | pivampicillin | 0.781 | 0.724 |
| cetirizine | remoxipride | 0.781 | 0.737 |
| flufenamic_acid | urapidil | 0.781 | 0.718 |
| daunorubicin | meclofenoxate | 0.781 | 0.716 |
| cefixime | roxithromycin | 0.781 | 0.799 |
| bromocriptine | dobutamine | 0.781 | 0.728 |
| galantamine | phenoxybenzamine | 0.781 | 0.757 |
| clindamycin | exemestane | 0.781 | 0.775 |
| ciclopirox | cisapride | 0.781 | 0.768 |
| gefitinib | sulconazole | 0.781 | 0.759 |
| etamsylate | sparteine | 0.781 | 0.777 |
| doxazosin | ethosuximide | 0.781 | 0.830 |
| liothyronine | niridazole | 0.781 | 0.758 |
| ketanserin | phenoxybenzamine | 0.781 | 0.738 |
| gliclazide | oleandomycin | 0.781 | 0.808 |
| gliclazide | oxybuprocaine | 0.781 | 0.687 |
| acepromazine | cycloserine | 0.781 | 0.873 |
| buflomedil | guanfacine | 0.781 | 0.715 |
| acepromazine | ethosuximide | 0.781 | 0.776 |
| fluvoxamine | zuclopenthixol | 0.781 | 0.732 |
| citiolone | gliclazide | 0.781 | 0.760 |
| metolazone | piribedil | 0.781 | 0.704 |
| digoxin | trimipramine | 0.781 | 0.868 |
| pridinol | vidarabine | 0.781 | 0.735 |
| meclofenoxate | pipemidic_acid | 0.781 | 0.686 |
| metergoline | practolol | 0.781 | 0.708 |
| isoconazole | propofol | 0.781 | 0.758 |
| cyproterone | meclofenoxate | 0.781 | 0.725 |
| bromocriptine | simvastatin | 0.781 | 0.755 |
| dipyridamole | propylthiouracil | 0.781 | 0.774 |
| betahistine | nifurtimox | 0.781 | 0.688 |
| fenoterol | sirolimus | 0.781 | 0.855 |

| | | | |
|---|---|---|---|
| lomefloxacin | repaglinide | 0.781 | 0.714 |
| cyclobenzaprine | oxetacaine | 0.781 | 0.776 |
| nimesulide | tiratricol | 0.781 | 0.718 |
| repaglinide | sulfinpyrazone | 0.781 | 0.739 |
| bisacodyl | pentoxyverine | 0.781 | 0.737 |
| mitoxantrone | physostigmine | 0.781 | 0.766 |
| albendazole | benzethonium_chloric | 0.781 | 0.700 |
| doxorubicin | galantamine | 0.781 | 0.739 |
| capsaicin | ganciclovir | 0.781 | 0.713 |
| ketoprofen | progesterone | 0.781 | 0.705 |
| glibenclamide | oxybutynin | 0.781 | 0.737 |
| altizide | phenazopyridine | 0.781 | 0.711 |
| felodipine | pyrvinium | 0.781 | 0.805 |
| pimozide | terbutaline | 0.781 | 0.763 |
| miconazole | semustine | 0.781 | 0.762 |
| bisoprolol | carbachol | 0.781 | 0.790 |
| etoposide | zuclopenthixol | 0.781 | 0.733 |
| oxamniquine | rimexolone | 0.781 | 0.734 |
| pridinol | sulfametoxydiazine | 0.781 | 0.699 |
| capsaicin | hymecromone | 0.781 | 0.755 |
| desoxycortone | fluspirilene | 0.781 | 0.693 |
| mebeverine | oxybutynin | 0.781 | 0.748 |
| dirithromycin | flufenamic_acid | 0.781 | 0.837 |
| deptropine | dipyridamole | 0.781 | 0.770 |
| fluticasone | letrozole | 0.781 | 0.776 |
| biotin | nefopam | 0.781 | 0.772 |
| cinchocaine | flufenamic_acid | 0.781 | 0.677 |
| dihydroergotamine | fendiline | 0.781 | 0.778 |
| etidronic_acid | flunisolide | 0.781 | 0.803 |
| benzylpenicillin | ronidazole | 0.781 | 0.727 |
| betahistine | pipemidic_acid | 0.781 | 0.673 |
| bepridil | ketoconazole | 0.781 | 0.778 |
| benzocaine | nadolol | 0.781 | 0.726 |
| talampicillin | urapidil | 0.781 | 0.712 |
| diclofenamide | profenamine | 0.781 | 0.722 |
| dihydroergotamine | syrosingopine | 0.781 | 0.721 |
| betaxolol | metamizole_sodium | 0.781 | 0.737 |
| pheneticillin | podophyllotoxin | 0.781 | 0.722 |
| enoxacin | tiapride | 0.781 | 0.658 |
| lynestrenol | suloctidil | 0.781 | 0.730 |
| niclosamide | vorinostat | 0.781 | 0.655 |
| hydrocortisone | ioversol | 0.781 | 0.736 |
| bisacodyl | clioquinol | 0.781 | 0.779 |
| bromopride | tropicamide | 0.781 | 0.696 |
| fluspirilene | prochlorperazine | 0.781 | 0.744 |
| econazole | propofol | 0.781 | 0.775 |
| cyproterone | etomidate | 0.781 | 0.751 |
| desoxycortone | omeprazole | 0.781 | 0.747 |
| omeprazole | triflusal | 0.781 | 0.728 |
| omeprazole | pivampicillin | 0.781 | 0.708 |

| | | | |
|---|---|---|---|
| apomorphine | levamisole | 0.781 | 0.724 |
| clomipramine | ipratropium_bromide | 0.781 | 0.754 |
| alprenolol | guanethidine | 0.781 | 0.693 |
| cetirizine | vidarabine | 0.781 | 0.775 |
| lisuride | maprotiline | 0.781 | 0.733 |
| bromopride | fluspirilene | 0.781 | 0.720 |
| doxylamine | naltrexone | 0.781 | 0.728 |
| chlorcyclizine | trimipramine | 0.781 | 0.656 |
| levonorgestrel | mitoxantrone | 0.781 | 0.766 |
| dyclonine | lomustine | 0.781 | 0.679 |
| benperidol | cefalexin | 0.781 | 0.692 |
| atovaquone | doxorubicin | 0.781 | 0.696 |
| labetalol | oxetacaine | 0.781 | 0.707 |
| dipivefrine | nafcillin | 0.781 | 0.712 |
| labetalol | tyloxapol | 0.781 | 0.844 |
| doxorubicin | promethazine | 0.781 | 0.759 |
| benperidol | glibenclamide | 0.781 | 0.695 |
| milrinone | tobramycin | 0.781 | 0.805 |
| cefalotin | ioversol | 0.781 | 0.762 |
| metyrapone | oxolinic_acid | 0.781 | 0.668 |
| etidronic_acid | proscillaridin | 0.781 | 0.814 |
| chloropyramine | iocetamic_acid | 0.781 | 0.732 |
| fluvoxamine | ketanserin | 0.781 | 0.758 |
| scopolamine | trioxysalen | 0.781 | 0.713 |
| bupivacaine | prednisolone | 0.781 | 0.746 |
| mometasone | pizotifen | 0.781 | 0.782 |
| doxazosin | sertaconazole | 0.781 | 0.749 |
| quinethazone | tolbutamide | 0.781 | 0.685 |
| clonidine | mepacrine | 0.781 | 0.713 |
| piperidolate | urapidil | 0.781 | 0.749 |
| oxamic_acid | thiocolchicoside | 0.781 | 0.898 |
| chloramphenicol | ipratropium_bromide | 0.781 | 0.665 |
| ethosuximide | levamisole | 0.781 | 0.739 |
| metergoline | noretynodrel | 0.781 | 0.771 |
| hydrocortisone | sulfamerazine | 0.781 | 0.758 |
| deptropine | methazolamide | 0.781 | 0.780 |
| lymecycline | sulconazole | 0.781 | 0.793 |
| ketanserin | mefloquine | 0.781 | 0.754 |
| capsaicin | carbachol | 0.781 | 0.773 |
| labetalol | menadione | 0.781 | 0.724 |
| cefalexin | methylergometrine | 0.781 | 0.680 |
| meticrane | pargyline | 0.781 | 0.738 |
| betaxolol | irinotecan | 0.781 | 0.753 |
| bromopride | ioversol | 0.781 | 0.785 |
| hydralazine | simvastatin | 0.781 | 0.793 |
| meclofenoxate | scopolamine | 0.781 | 0.676 |
| doxazosin | torasemide | 0.781 | 0.702 |
| clomipramine | phenazopyridine | 0.781 | 0.727 |
| hydroflumethiazide | sirolimus | 0.781 | 0.862 |
| rimexolone | vidarabine | 0.781 | 0.762 |

| | | | |
|---|---|---|---|
| ketanserin | levodopa | 0.781 | 0.746 |
| cinchocaine | fluspirilene | 0.781 | 0.740 |
| acepromazine | clobetasol | 0.781 | 0.757 |
| meptazinol | pyrimethamine | 0.781 | 0.714 |
| natamycin | ronidazole | 0.781 | 0.850 |
| benzylpenicillin | tyloxapol | 0.781 | 0.849 |
| chlorambucil | latamoxef | 0.781 | 0.775 |
| bromperidol | milrinone | 0.781 | 0.744 |
| amitriptyline | mefloquine | 0.781 | 0.757 |
| dorzolamide | natamycin | 0.781 | 0.796 |
| bendroflumethiazide | piperidolate | 0.781 | 0.708 |
| bufexamac | lisinopril | 0.782 | 0.750 |
| astemizole | sulconazole | 0.782 | 0.748 |
| halcinonide | tropicamide | 0.782 | 0.754 |
| domperidone | trifluoperazine | 0.782 | 0.728 |
| acepromazine | xylometazoline | 0.782 | 0.734 |
| butoconazole | chlorzoxazone | 0.782 | 0.775 |
| doxazosin | vidarabine | 0.782 | 0.723 |
| naltrexone | pralidoxime | 0.782 | 0.736 |
| azacitidine | nitrofurantoin | 0.782 | 0.666 |
| nadolol | nafcillin | 0.782 | 0.691 |
| daunorubicin | methotrexate | 0.782 | 0.724 |
| hydralazine | tiapride | 0.782 | 0.711 |
| econazole | syrosingopine | 0.782 | 0.815 |
| estriol | piromidic_acid | 0.782 | 0.688 |
| doxylamine | ofloxacin | 0.782 | 0.755 |
| bezafibrate | natamycin | 0.782 | 0.779 |
| ciclosporin | rescinnamine | 0.782 | 0.806 |
| aminocaproic_acid | remoxipride | 0.782 | 0.793 |
| betahistine | griseofulvin | 0.782 | 0.737 |
| fenbufen | medrysone | 0.782 | 0.710 |
| ivermectin | quinisocaine | 0.782 | 0.855 |
| piracetam | rimexolone | 0.782 | 0.795 |
| etoposide | pimozide | 0.782 | 0.749 |
| bromperidol | deptropine | 0.782 | 0.796 |
| benzonatate | hydroflumethiazide | 0.782 | 0.833 |
| clobetasol | gliclazide | 0.782 | 0.705 |
| ifenprodil | metamizole_sodium | 0.782 | 0.720 |
| azlocillin | sulfametoxydiazine | 0.782 | 0.742 |
| mesoridazine | proscillaridin | 0.782 | 0.784 |
| chloramphenicol | propylthiouracil | 0.782 | 0.710 |
| altizide | tyloxapol | 0.782 | 0.855 |
| deptropine | sulfametoxydiazine | 0.782 | 0.739 |
| debrisoquine | profenamine | 0.782 | 0.696 |
| felodipine | sulconazole | 0.782 | 0.724 |
| fluocinonide | pentoxyverine | 0.782 | 0.761 |
| disulfiram | quinisocaine | 0.782 | 0.727 |
| nortriptyline | rifabutin | 0.782 | 0.844 |
| podophyllotoxin | streptozocin | 0.782 | 0.704 |
| irinotecan | rimexolone | 0.782 | 0.790 |

| | | | |
|---|---|---|---|
| nortriptyline | phenoxybenzamine | 0.782 | 0.718 |
| bisacodyl | tobramycin | 0.782 | 0.771 |
| chlorcyclizine | disulfiram | 0.782 | 0.758 |
| budesonide | trazodone | 0.782 | 0.752 |
| dipyridamole | fenoprofen | 0.782 | 0.764 |
| phenoxybenzamine | vidarabine | 0.782 | 0.744 |
| cefalotin | cinchocaine | 0.782 | 0.723 |
| digoxin | vorinostat | 0.782 | 0.828 |
| talampicillin | tobramycin | 0.782 | 0.709 |
| clotrimazole | dienestrol | 0.782 | 0.740 |
| metyrapone | tiapride | 0.782 | 0.732 |
| pentoxyverine | tranylcypromine | 0.782 | 0.707 |
| etofenamate | meclozine | 0.782 | 0.756 |
| phenoxybenzamine | raloxifene | 0.782 | 0.776 |
| deptropine | suloctidil | 0.782 | 0.769 |
| pimethixene | syrosingopine | 0.782 | 0.828 |
| irinotecan | mitoxantrone | 0.782 | 0.716 |
| isradipine | ursodeoxycholic_acid | 0.782 | 0.767 |
| dipyridamole | liothyronine | 0.782 | 0.742 |
| hyoscyamine | milrinone | 0.782 | 0.714 |
| monobenzone | primaquine | 0.782 | 0.663 |
| glibenclamide | levonorgestrel | 0.782 | 0.786 |
| cefamandole | isocarboxazid | 0.782 | 0.731 |
| reserpine | theophylline | 0.782 | 0.814 |
| bromocriptine | promethazine | 0.782 | 0.775 |
| betahistine | etidronic_acid | 0.782 | 0.763 |
| mometasone | zuclopenthixol | 0.782 | 0.753 |
| bromocriptine | flupentixol | 0.782 | 0.712 |
| fluspirilene | zuclopenthixol | 0.782 | 0.723 |
| ceforanide | moroxydine | 0.782 | 0.812 |
| piperidolate | tropicamide | 0.782 | 0.690 |
| azapropazone | nomegestrol | 0.782 | 0.752 |
| bepridil | diltiazem | 0.782 | 0.718 |
| fenbendazole | phenoxybenzamine | 0.782 | 0.718 |
| desoxycortone | terbutaline | 0.782 | 0.746 |
| doxorubicin | isradipine | 0.782 | 0.740 |
| cyproterone | oxedrine | 0.782 | 0.773 |
| dacarbazine | flunarizine | 0.782 | 0.800 |
| bromocriptine | chlorzoxazone | 0.782 | 0.828 |
| dropropizine | lisinopril | 0.782 | 0.708 |
| clindamycin | iopanoic_acid | 0.782 | 0.735 |
| apomorphine | dacarbazine | 0.782 | 0.674 |
| oxybutynin | pyrvinium | 0.782 | 0.798 |
| pimozide | sulconazole | 0.782 | 0.741 |
| cefixime | piribedil | 0.782 | 0.696 |
| flunisolide | molindone | 0.782 | 0.729 |
| flunarizine | norethisterone | 0.782 | 0.764 |
| spectinomycin | sulconazole | 0.782 | 0.786 |
| metrifonate | tyloxapol | 0.782 | 0.909 |
| bepridil | butoconazole | 0.782 | 0.708 |

| | | | |
|---|---|---|---|
| chlorambucil | metacycline | 0.782 | 0.753 |
| bambuterol | clomipramine | 0.782 | 0.770 |
| sulfamethoxypyridazine | talampicillin | 0.782 | 0.710 |
| loperamide | mebhydrolin | 0.782 | 0.764 |
| calcium_folinate | cefazolin | 0.782 | 0.692 |
| iodixanol | levobunolol | 0.782 | 0.850 |
| pentoxyverine | tyloxapol | 0.782 | 0.853 |
| naloxone | vinpocetine | 0.782 | 0.729 |
| calcium_pantothenate | meclofenoxate | 0.782 | 0.797 |
| levomepromazine | prenylamine | 0.782 | 0.711 |
| cyproheptadine | fluphenazine | 0.782 | 0.707 |
| medrysone | tranylcypromine | 0.782 | 0.782 |
| cinchocaine | norethisterone | 0.782 | 0.769 |
| procaine | vinpocetine | 0.782 | 0.712 |
| cinchocaine | meropenem | 0.782 | 0.739 |
| gliclazide | picotamide | 0.782 | 0.722 |
| cortisone | gliclazide | 0.782 | 0.728 |
| chloramphenicol | propafenone | 0.782 | 0.732 |
| piperidolate | triflusal | 0.782 | 0.752 |
| cefepime | cyclopentolate | 0.782 | 0.764 |
| hyoscyamine | sulconazole | 0.782 | 0.741 |
| carbachol | cinoxacin | 0.782 | 0.706 |
| hydrocortisone | pyrantel | 0.782 | 0.802 |
| protriptyline | trazodone | 0.782 | 0.784 |
| doxorubicin | etofenamate | 0.782 | 0.696 |
| cyclizine | mometasone | 0.782 | 0.784 |
| cortisone | triflusal | 0.782 | 0.772 |
| doxazosin | proxymetacaine | 0.782 | 0.681 |
| betaxolol | niridazole | 0.782 | 0.745 |
| flecainide | haloperidol | 0.782 | 0.739 |
| ioversol | meticrane | 0.782 | 0.760 |
| fendiline | podophyllotoxin | 0.782 | 0.755 |
| levonorgestrel | meptazinol | 0.782 | 0.738 |
| ifenprodil | pimethixene | 0.782 | 0.768 |
| meptazinol | ofloxacin | 0.782 | 0.737 |
| hydroflumethiazide | imipramine | 0.782 | 0.778 |
| flunisolide | sulfamerazine | 0.782 | 0.744 |
| bromopride | talampicillin | 0.782 | 0.744 |
| chlorzoxazone | promazine | 0.782 | 0.740 |
| cyproheptadine | flupentixol | 0.782 | 0.691 |
| methylergometrine | oxetacaine | 0.782 | 0.726 |
| ifenprodil | sulpiride | 0.782 | 0.739 |
| milrinone | rolitetracycline | 0.782 | 0.805 |
| doxorubicin | imipenem | 0.782 | 0.742 |
| antazoline | mebeverine | 0.782 | 0.779 |
| aminophylline | flufenamic_acid | 0.782 | 0.695 |
| clomifene | clomipramine | 0.782 | 0.725 |
| fenbendazole | pimozide | 0.782 | 0.735 |
| decitabine | iloprost | 0.782 | 0.784 |
| monobenzone | rimexolone | 0.782 | 0.771 |

| | | | |
|---|---|---|---|
| pyrantel | trioxysalen | 0.782 | 0.692 |
| ipratropium_bromide | pyrazinamide | 0.782 | 0.755 |
| bepridil | pentoxyverine | 0.782 | 0.718 |
| terconazole | terfenadine | 0.782 | 0.713 |
| gliclazide | promazine | 0.782 | 0.771 |
| ciclopirox | maprotiline | 0.782 | 0.738 |
| fenoprofen | fluvoxamine | 0.782 | 0.732 |
| cycloserine | doxazosin | 0.782 | 0.858 |
| amiodarone | norethisterone | 0.782 | 0.772 |
| glibenclamide | proxyphylline | 0.782 | 0.758 |
| betahistine | tropicamide | 0.782 | 0.712 |
| cefazolin | salbutamol | 0.782 | 0.749 |
| metacycline | procaine | 0.782 | 0.689 |
| ethotoin | piperidolate | 0.782 | 0.683 |
| betahistine | capsaicin | 0.782 | 0.730 |
| liothyronine | nicotinic_acid | 0.782 | 0.790 |
| aminoglutethimide | ifenprodil | 0.782 | 0.744 |
| fenoterol | mepacrine | 0.782 | 0.733 |
| lisinopril | trazodone | 0.782 | 0.753 |
| fluvastatin | sulfamethoxypyridazir | 0.782 | 0.728 |
| alimemazine | bisacodyl | 0.782 | 0.727 |
| clopamide | nizatidine | 0.782 | 0.728 |
| propylthiouracil | rolitetracycline | 0.782 | 0.810 |
| medrysone | spectinomycin | 0.782 | 0.735 |
| fenoterol | roxithromycin | 0.782 | 0.821 |
| clomifene | fluphenazine | 0.782 | 0.724 |
| cetirizine | etamsylate | 0.782 | 0.810 |
| cetirizine | flufenamic_acid | 0.782 | 0.718 |
| mefloquine | thiethylperazine | 0.782 | 0.735 |
| chlorcyclizine | felodipine | 0.782 | 0.714 |
| alfaxalone | isradipine | 0.782 | 0.733 |
| mianserin | triamcinolone | 0.782 | 0.802 |
| acepromazine | midecamycin | 0.782 | 0.836 |
| azacitidine | monobenzone | 0.782 | 0.713 |
| ioxaglic_acid | rimexolone | 0.782 | 0.793 |
| flunarizine | miconazole | 0.782 | 0.731 |
| loxapine | profenamine | 0.782 | 0.658 |
| terbutaline | triflusal | 0.782 | 0.667 |
| aminohippuric_acid | carbachol | 0.782 | 0.700 |
| (-)-catechin | pentetrazol | 0.782 | 0.742 |
| bezafibrate | cefalotin | 0.782 | 0.701 |
| terfenadine | thiethylperazine | 0.782 | 0.758 |
| clindamycin | etofenamate | 0.782 | 0.740 |
| mometasone | syrosingopine | 0.782 | 0.768 |
| bufexamac | tyloxapol | 0.782 | 0.870 |
| fenoprofen | remoxipride | 0.782 | 0.709 |
| bromperidol | piperidolate | 0.782 | 0.734 |
| imipenem | sulfafurazole | 0.782 | 0.721 |
| chlorzoxazone | meclozine | 0.782 | 0.781 |
| dicoumarol | doxorubicin | 0.782 | 0.694 |

| | | | |
|---|---|---|---|
| daunorubicin | procaine | 0.782 | 0.703 |
| doxorubicin | testosterone | 0.782 | 0.775 |
| chloramphenicol | nitrofurantoin | 0.782 | 0.710 |
| miconazole | verteporfin | 0.782 | 0.802 |
| moxonidine | verteporfin | 0.782 | 0.812 |
| simvastatin | spectinomycin | 0.782 | 0.743 |
| dydrogesterone | pentoxyverine | 0.782 | 0.779 |
| griseofulvin | sulfathiazole | 0.782 | 0.665 |
| citiolone | medrysone | 0.782 | 0.801 |
| imipramine | terfenadine | 0.782 | 0.773 |
| rimexolone | sulfamethoxazole | 0.782 | 0.757 |
| bromocriptine | pentamidine | 0.782 | 0.760 |
| dextromethorphan | talampicillin | 0.782 | 0.802 |
| acetylsalicylic_acid | amiodarone | 0.782 | 0.791 |
| meptazinol | pralidoxime | 0.782 | 0.720 |
| meptazinol | sotalol | 0.782 | 0.750 |
| capsaicin | rolitetracycline | 0.783 | 0.761 |
| deptropine | succinylsulfathiazole | 0.783 | 0.794 |
| dipyridamole | fluocinonide | 0.783 | 0.746 |
| (-)-atenolol | rolitetracycline | 0.783 | 0.759 |
| acetylsalicylic_acid | metacycline | 0.783 | 0.746 |
| doxorubicin | omeprazole | 0.783 | 0.699 |
| dacarbazine | etomidate | 0.783 | 0.681 |
| cefuroxime | cinoxacin | 0.783 | 0.718 |
| ioversol | paracetamol | 0.783 | 0.833 |
| tobramycin | torasemide | 0.783 | 0.755 |
| noretynodrel | trifluoperazine | 0.783 | 0.740 |
| deptropine | meclozine | 0.783 | 0.719 |
| lymecycline | phenindione | 0.783 | 0.781 |
| pentoxyverine | talampicillin | 0.783 | 0.727 |
| gliclazide | lomefloxacin | 0.783 | 0.724 |
| azacitidine | tribenoside | 0.783 | 0.785 |
| demecolcine | pimozide | 0.783 | 0.760 |
| gliclazide | methazolamide | 0.783 | 0.718 |
| chlorzoxazone | omeprazole | 0.783 | 0.695 |
| metergoline | tamoxifen | 0.783 | 0.722 |
| carbachol | etamsylate | 0.783 | 0.673 |
| phenoxybenzamine | talampicillin | 0.783 | 0.759 |
| ifenprodil | meclofenoxate | 0.783 | 0.709 |
| daunorubicin | thiocolchicoside | 0.783 | 0.698 |
| pimozide | reserpine | 0.783 | 0.731 |
| rimexolone | urapidil | 0.783 | 0.751 |
| cyproterone | dacarbazine | 0.783 | 0.786 |
| amiodarone | gliclazide | 0.783 | 0.747 |
| etomidate | liothyronine | 0.783 | 0.729 |
| amoxapine | diltiazem | 0.783 | 0.727 |
| cefsulodin | etidronic_acid | 0.783 | 0.822 |
| budesonide | cetirizine | 0.783 | 0.739 |
| phenformin | salbutamol | 0.783 | 0.694 |
| fludroxycortide | promazine | 0.783 | 0.786 |

| | | | |
|---|---|---|---|
| dipyridamole | irinotecan | 0.783 | 0.719 |
| lomefloxacin | zimeldine | 0.783 | 0.713 |
| guanfacine | sulconazole | 0.783 | 0.726 |
| medrysone | prilocaine | 0.783 | 0.735 |
| chlorambucil | ethotoin | 0.783 | 0.723 |
| etamsylate | tiabendazole | 0.783 | 0.686 |
| demecolcine | mitoxantrone | 0.783 | 0.691 |
| naloxone | sulfamethoxypyridazir | 0.783 | 0.725 |
| medrysone | talampicillin | 0.783 | 0.727 |
| nicergoline | thioproperazine | 0.783 | 0.742 |
| altizide | benzylpenicillin | 0.783 | 0.679 |
| flunarizine | mesoridazine | 0.783 | 0.766 |
| betahistine | daunorubicin | 0.783 | 0.736 |
| chlorprothixene | terconazole | 0.783 | 0.776 |
| octopamine | trichlormethiazide | 0.783 | 0.673 |
| fenoterol | pivampicillin | 0.783 | 0.730 |
| loperamide | mepacrine | 0.783 | 0.724 |
| (-)-atenolol | dacarbazine | 0.783 | 0.727 |
| chlorprothixene | ciclopirox | 0.783 | 0.709 |
| norfloxacin | triflusal | 0.783 | 0.710 |
| fluorometholone | fluspirilene | 0.783 | 0.708 |
| meptazinol | ornidazole | 0.783 | 0.699 |
| cycloserine | oxedrine | 0.783 | 0.772 |
| ganciclovir | reserpine | 0.783 | 0.748 |
| clomipramine | promethazine | 0.783 | 0.661 |
| methylergometrine | suloctidil | 0.783 | 0.735 |
| gefitinib | paclitaxel | 0.783 | 0.799 |
| amitriptyline | oxetacaine | 0.783 | 0.772 |
| nalidixic_acid | sulconazole | 0.783 | 0.752 |
| aminophenazone | ivermectin | 0.783 | 0.892 |
| etamsylate | mitoxantrone | 0.783 | 0.767 |
| betaxolol | bezafibrate | 0.783 | 0.682 |
| disulfiram | protriptyline | 0.783 | 0.757 |
| bezafibrate | etamsylate | 0.783 | 0.776 |
| fluspirilene | latamoxef | 0.783 | 0.734 |
| fludroxycortide | metaraminol | 0.783 | 0.798 |
| ipratropium_bromide | nalbuphine | 0.783 | 0.694 |
| doxazosin | moxonidine | 0.783 | 0.699 |
| bepridil | trimipramine | 0.783 | 0.662 |
| proxyphylline | sulfafurazole | 0.783 | 0.659 |
| amoxicillin | meptazinol | 0.783 | 0.779 |
| daunorubicin | sulfamethoxazole | 0.783 | 0.736 |
| liothyronine | meropenem | 0.783 | 0.730 |
| propylthiouracil | trimethadione | 0.783 | 0.667 |
| clotrimazole | lynestrenol | 0.783 | 0.684 |
| clemastine | podophyllotoxin | 0.783 | 0.752 |
| aciclovir | biperiden | 0.783 | 0.777 |
| cetirizine | sulfamerazine | 0.783 | 0.749 |
| molindone | triflusal | 0.783 | 0.719 |
| meptazinol | sulfametoxydiazine | 0.783 | 0.717 |

| | | | |
|---|---|---|---|
| remoxipride | repaglinide | 0.783 | 0.741 |
| ketanserin | natamycin | 0.783 | 0.795 |
| amikacin | tiapride | 0.783 | 0.779 |
| ivermectin | maprotiline | 0.783 | 0.874 |
| procainamide | prochlorperazine | 0.783 | 0.746 |
| ethotoin | meclozine | 0.783 | 0.758 |
| sulfaphenazole | urapidil | 0.783 | 0.731 |
| bisacodyl | naloxone | 0.783 | 0.763 |
| glibenclamide | vinpocetine | 0.783 | 0.781 |
| lisinopril | meticrane | 0.783 | 0.751 |
| nafcillin | phenazone | 0.783 | 0.734 |
| pentetic_acid | piperidolate | 0.783 | 0.786 |
| nicotinic_acid | rolitetracycline | 0.783 | 0.796 |
| metamizole_sodium | repaglinide | 0.783 | 0.721 |
| colchicine | ketanserin | 0.783 | 0.738 |
| clotrimazole | metergoline | 0.783 | 0.767 |
| exemestane | flufenamic_acid | 0.783 | 0.696 |
| diltiazem | ethotoin | 0.783 | 0.720 |
| chlorzoxazone | diclofenac | 0.783 | 0.685 |
| spectinomycin | sulfaphenazole | 0.783 | 0.728 |
| bromocriptine | emetine | 0.783 | 0.718 |
| amoxicillin | nitrendipine | 0.783 | 0.748 |
| clonidine | trifluoperazine | 0.783 | 0.740 |
| rifampicin | trimethadione | 0.783 | 0.883 |
| miconazole | naphazoline | 0.783 | 0.703 |
| pargyline | trazodone | 0.783 | 0.766 |
| minaprine | sulfathiazole | 0.783 | 0.712 |
| proscillaridin | sulconazole | 0.783 | 0.816 |
| acetohexamide | methocarbamol | 0.783 | 0.678 |
| ivermectin | promazine | 0.783 | 0.873 |
| amiodarone | meticrane | 0.783 | 0.771 |
| proxyphylline | sulfamethoxypyridazir | 0.783 | 0.665 |
| bepridil | gliclazide | 0.783 | 0.749 |
| nialamide | tyloxapol | 0.783 | 0.857 |
| daunorubicin | remoxipride | 0.783 | 0.713 |
| reserpine | sulfamethoxypyridazir | 0.783 | 0.760 |
| labetalol | vinpocetine | 0.783 | 0.742 |
| butoconazole | imipramine | 0.783 | 0.662 |
| digoxin | miconazole | 0.783 | 0.856 |
| ketanserin | tyloxapol | 0.783 | 0.846 |
| meclozine | trazodone | 0.783 | 0.709 |
| alimemazine | estriol | 0.783 | 0.715 |
| colecalciferol | hydrocortisone | 0.783 | 0.733 |
| deptropine | repaglinide | 0.783 | 0.751 |
| fluorometholone | labetalol | 0.783 | 0.732 |
| repaglinide | ticlopidine | 0.783 | 0.765 |
| deptropine | desoxycortone | 0.783 | 0.772 |
| estriol | phenazopyridine | 0.783 | 0.684 |
| enalapril | pergolide | 0.783 | 0.750 |
| dacarbazine | urapidil | 0.783 | 0.729 |

| | | | |
|---|---|---|---|
| fenbendazole | levomepromazine | 0.783 | 0.701 |
| deptropine | dextromethorphan | 0.783 | 0.717 |
| bufexamac | rimexolone | 0.783 | 0.718 |
| milrinone | sulfafurazole | 0.783 | 0.725 |
| remoxipride | trazodone | 0.783 | 0.701 |
| metaraminol | tobramycin | 0.783 | 0.819 |
| amrinone | cefapirin | 0.783 | 0.707 |
| ifenprodil | oxybuprocaine | 0.783 | 0.701 |
| acepromazine | cortisone | 0.783 | 0.754 |
| cefalexin | colchicine | 0.783 | 0.738 |
| etofylline | sulconazole | 0.783 | 0.771 |
| medrysone | piribedil | 0.783 | 0.708 |
| biotin | famotidine | 0.783 | 0.718 |
| ronidazole | thiocolchicoside | 0.783 | 0.780 |
| cinchocaine | phenoxybenzamine | 0.783 | 0.742 |
| ticlopidine | trifluoperazine | 0.783 | 0.730 |
| altretamine | aminohippuric_acid | 0.783 | 0.686 |
| dacarbazine | deferoxamine | 0.783 | 0.852 |
| remoxipride | ursodeoxycholic_acid | 0.783 | 0.766 |
| remoxipride | zimeldine | 0.783 | 0.716 |
| isradipine | talampicillin | 0.783 | 0.755 |
| norfloxacin | pargyline | 0.783 | 0.758 |
| cefalotin | meropenem | 0.783 | 0.680 |
| ciclosporin | flupentixol | 0.783 | 0.848 |
| cycloserine | propofol | 0.783 | 0.808 |
| astemizole | etoposide | 0.783 | 0.764 |
| ifenprodil | norethisterone | 0.783 | 0.718 |
| felodipine | loxapine | 0.783 | 0.705 |
| calcium_pantothenate | mitoxantrone | 0.783 | 0.788 |
| ethosuximide | etofylline | 0.783 | 0.697 |
| felodipine | rimexolone | 0.783 | 0.751 |
| mometasone | terconazole | 0.783 | 0.761 |
| hyoscyamine | levamisole | 0.783 | 0.733 |
| dicycloverine | miconazole | 0.783 | 0.702 |
| ambroxol | decitabine | 0.783 | 0.722 |
| fluvoxamine | sotalol | 0.783 | 0.707 |
| clemastine | phenazopyridine | 0.783 | 0.742 |
| ivermectin | meclozine | 0.783 | 0.843 |
| meticrane | sulfadiazine | 0.783 | 0.685 |
| ciclopirox | pyrvinium | 0.783 | 0.810 |
| acetylsalicylic_acid | ronidazole | 0.783 | 0.662 |
| oxymetazoline | pargyline | 0.783 | 0.698 |
| bromocriptine | clonidine | 0.783 | 0.806 |
| etofylline | ipratropium_bromide | 0.783 | 0.740 |
| cyproterone | gliclazide | 0.783 | 0.752 |
| pargyline | trimetazidine | 0.783 | 0.691 |
| menadione | tropicamide | 0.783 | 0.741 |
| gliclazide | pyrazinamide | 0.784 | 0.740 |
| altizide | trioxysalen | 0.784 | 0.713 |
| cinchocaine | metyrapone | 0.784 | 0.710 |

| | | | |
|---|---|---|---|
| flunisolide | verteporfin | 0.784 | 0.803 |
| oxamniquine | promazine | 0.784 | 0.718 |
| ipratropium_bromide | sulfamethoxypyridazir | 0.784 | 0.678 |
| betaxolol | triflusal | 0.784 | 0.770 |
| chlorpropamide | dobutamine | 0.784 | 0.714 |
| cefixime | latamoxef | 0.784 | 0.693 |
| halofantrine | trapidil | 0.784 | 0.756 |
| alimemazine | latamoxef | 0.784 | 0.799 |
| alverine | xylometazoline | 0.784 | 0.722 |
| betaxolol | trimetazidine | 0.784 | 0.725 |
| moxisylyte | triflupromazine | 0.784 | 0.680 |
| imipenem | roxithromycin | 0.784 | 0.824 |
| repaglinide | sulfafurazole | 0.784 | 0.760 |
| liothyronine | meclofenoxate | 0.784 | 0.720 |
| fipexide | thiocolchicoside | 0.784 | 0.754 |
| doxorubicin | saquinavir | 0.784 | 0.693 |
| metyrapone | pivampicillin | 0.784 | 0.757 |
| disulfiram | metixene | 0.784 | 0.752 |
| chloroquine | dosulepin | 0.784 | 0.722 |
| propofol | trazodone | 0.784 | 0.761 |
| monobenzone | nilutamide | 0.784 | 0.718 |
| lomustine | perphenazine | 0.784 | 0.774 |
| clindamycin | sulfathiazole | 0.784 | 0.748 |
| bepridil | ifenprodil | 0.784 | 0.754 |
| bromocriptine | lynestrenol | 0.784 | 0.783 |
| metoprolol | zuclopenthixol | 0.784 | 0.726 |
| diphenylpyraline | trifluoperazine | 0.784 | 0.698 |
| methapyrilene | rimexolone | 0.784 | 0.754 |
| metyrapone | urapidil | 0.784 | 0.733 |
| glibenclamide | propafenone | 0.784 | 0.716 |
| reserpine | salbutamol | 0.784 | 0.778 |
| sulfathiazole | trichlormethiazide | 0.784 | 0.657 |
| fulvestrant | meclozine | 0.784 | 0.797 |
| etamsylate | glibenclamide | 0.784 | 0.767 |
| clindamycin | repaglinide | 0.784 | 0.712 |
| cyanocobalamin | etofenamate | 0.784 | 0.853 |
| loperamide | promazine | 0.784 | 0.752 |
| loperamide | testosterone | 0.784 | 0.782 |
| mefloquine | procarbazine | 0.784 | 0.714 |
| acepromazine | norethisterone | 0.784 | 0.745 |
| acetylsalicylic_acid | imipenem | 0.784 | 0.710 |
| fluocinonide | sulfadimidine | 0.784 | 0.754 |
| felodipine | iocetamic_acid | 0.784 | 0.701 |
| etomidate | medrysone | 0.784 | 0.748 |
| etodolac | fenoprofen | 0.784 | 0.668 |
| altretamine | mephenesin | 0.784 | 0.694 |
| doxorubicin | procyclidine | 0.784 | 0.775 |
| ivermectin | noscapine | 0.784 | 0.836 |
| capsaicin | metacycline | 0.784 | 0.757 |
| chloramphenicol | piracetam | 0.784 | 0.714 |

| | | | |
|---|---|---|---|
| minaprine | pyrazinamide | 0.784 | 0.710 |
| ioversol | propofol | 0.784 | 0.841 |
| haloperidol | triflupromazine | 0.784 | 0.738 |
| mephenesin | nitrofurantoin | 0.784 | 0.669 |
| chlorcyclizine | profenamine | 0.784 | 0.689 |
| cycloserine | natamycin | 0.784 | 0.910 |
| bisacodyl | tinidazole | 0.784 | 0.731 |
| oxybuprocaine | troleandomycin | 0.784 | 0.831 |
| cinoxacin | pridinol | 0.784 | 0.750 |
| estriol | picotamide | 0.784 | 0.757 |
| biotin | pilocarpine | 0.784 | 0.692 |
| cefalotin | metyrapone | 0.784 | 0.725 |
| loxapine | mometasone | 0.784 | 0.744 |
| diltiazem | labetalol | 0.784 | 0.741 |
| doxylamine | ethambutol | 0.784 | 0.757 |
| gliclazide | piribedil | 0.784 | 0.687 |
| irinotecan | scopolamine | 0.784 | 0.800 |
| adipiodone | deptropine | 0.784 | 0.810 |
| dienestrol | mefloquine | 0.784 | 0.753 |
| etoposide | vidarabine | 0.784 | 0.745 |
| pipemidic_acid | tiapride | 0.784 | 0.650 |
| cycloserine | sulfafurazole | 0.784 | 0.823 |
| imipramine | levocabastine | 0.784 | 0.800 |
| nabumetone | roxithromycin | 0.784 | 0.858 |
| pentetrazol | piroxicam | 0.784 | 0.779 |
| butoconazole | protriptyline | 0.784 | 0.662 |
| amphotericin_B | melatonin | 0.784 | 0.872 |
| clomifene | tyloxapol | 0.784 | 0.855 |
| amiodarone | tobramycin | 0.784 | 0.776 |
| acepromazine | propafenone | 0.784 | 0.701 |
| oxedrine | tetryzoline | 0.784 | 0.707 |
| rescinnamine | trimipramine | 0.784 | 0.805 |
| bromperidol | naloxone | 0.784 | 0.783 |
| dihydroergocristine | pergolide | 0.784 | 0.660 |
| bemegride | capsaicin | 0.784 | 0.792 |
| etidronic_acid | trichlormethiazide | 0.784 | 0.751 |
| citiolone | promazine | 0.784 | 0.797 |
| betahistine | pentamidine | 0.784 | 0.781 |
| etidronic_acid | theophylline | 0.784 | 0.699 |
| pentamidine | rimexolone | 0.784 | 0.778 |
| hydralazine | rolitetracycline | 0.784 | 0.789 |
| irinotecan | promethazine | 0.784 | 0.788 |
| estriol | sulfamerazine | 0.784 | 0.704 |
| betahistine | imipenem | 0.784 | 0.746 |
| monobenzone | natamycin | 0.784 | 0.860 |
| apomorphine | fenoprofen | 0.784 | 0.699 |
| medrysone | meropenem | 0.784 | 0.699 |
| repaglinide | torasemide | 0.784 | 0.750 |
| cinchocaine | omeprazole | 0.784 | 0.693 |
| tolfenamic_acid | trioxysalen | 0.784 | 0.671 |

| | | | |
|---|---|---|---|
| amitriptyline | loperamide | 0.784 | 0.767 |
| levamisole | norethisterone | 0.784 | 0.745 |
| etomidate | verteporfin | 0.784 | 0.824 |
| acetylsalicylic_acid | reserpine | 0.784 | 0.820 |
| carbachol | zomepirac | 0.784 | 0.714 |
| daunorubicin | tyloxapol | 0.784 | 0.812 |
| levamisole | natamycin | 0.784 | 0.865 |
| meropenem | ramipril | 0.784 | 0.714 |
| bepridil | trioxysalen | 0.784 | 0.785 |
| natamycin | tropicamide | 0.784 | 0.825 |
| daunorubicin | ipratropium_bromide | 0.784 | 0.747 |
| gliclazide | tranylcypromine | 0.784 | 0.720 |
| moxonidine | triflusal | 0.784 | 0.677 |
| gefitinib | tranylcypromine | 0.784 | 0.809 |
| remoxipride | ronidazole | 0.784 | 0.718 |
| ketanserin | tolazoline | 0.784 | 0.773 |
| econazole | emetine | 0.784 | 0.751 |
| meclozine | methapyrilene | 0.784 | 0.652 |
| tobramycin | urapidil | 0.784 | 0.736 |
| cycloserine | sulfamethoxypyridazir | 0.784 | 0.808 |
| atovaquone | gliclazide | 0.784 | 0.688 |
| famotidine | verteporfin | 0.784 | 0.790 |
| procaine | urapidil | 0.784 | 0.723 |
| betahistine | sulfamethoxypyridazir | 0.784 | 0.664 |
| menadione | meticrane | 0.784 | 0.708 |
| cinchocaine | nitrofurantoin | 0.784 | 0.707 |
| chloramphenicol | hyoscyamine | 0.784 | 0.680 |
| albendazole | bepridil | 0.784 | 0.759 |
| dydrogesterone | promazine | 0.784 | 0.749 |
| levonorgestrel | sulfaphenazole | 0.784 | 0.740 |
| flufenamic_acid | simvastatin | 0.784 | 0.741 |
| betaxolol | cycloserine | 0.784 | 0.803 |
| monobenzone | pyrvinium | 0.784 | 0.788 |
| cycloserine | etofenamate | 0.784 | 0.849 |
| bromocriptine | riluzole | 0.784 | 0.800 |
| cyproterone | remoxipride | 0.784 | 0.740 |
| progesterone | verteporfin | 0.784 | 0.807 |
| desoxycortone | ronidazole | 0.784 | 0.762 |
| benzethonium_chloride | latamoxef | 0.784 | 0.770 |
| chlortetracycline | oxamic_acid | 0.784 | 0.879 |
| metyrapone | prasterone | 0.784 | 0.738 |
| desoxycortone | tropicamide | 0.784 | 0.744 |
| cinchocaine | dacarbazine | 0.784 | 0.700 |
| clotrimazole | promethazine | 0.784 | 0.683 |
| capsaicin | piromidic_acid | 0.784 | 0.700 |
| fluspirilene | pentoxyverine | 0.784 | 0.731 |
| etoposide | noretynodrel | 0.784 | 0.785 |
| bromocriptine | menadione | 0.784 | 0.813 |
| aciclovir | levomepromazine | 0.784 | 0.692 |
| flurbiprofen | meglumine | 0.784 | 0.683 |

| | | | |
|---|---|---|---|
| isoxsuprine | oxetacaine | 0.784 | 0.726 |
| chlorprothixene | flunarizine | 0.784 | 0.771 |
| phenoxybenzamine | tamoxifen | 0.784 | 0.732 |
| nabumetone | ursodeoxycholic_acid | 0.784 | 0.738 |
| clofazimine | suloctidil | 0.784 | 0.736 |
| irinotecan | suloctidil | 0.784 | 0.754 |
| doxazosin | quinisocaine | 0.784 | 0.706 |
| amoxapine | bepridil | 0.784 | 0.709 |
| bromopride | scopolamine | 0.784 | 0.712 |
| lomefloxacin | mephenesin | 0.784 | 0.666 |
| biotin | glycopyrronium_brom | 0.784 | 0.769 |
| etacrynic_acid | latamoxef | 0.784 | 0.773 |
| aminoglutethimide | diethylcarbamazine | 0.784 | 0.737 |
| acetohexamide | dipyridamole | 0.784 | 0.767 |
| dipyridamole | piperidolate | 0.784 | 0.779 |
| fenoprofen | ifenprodil | 0.784 | 0.698 |
| ethosuximide | propafenone | 0.784 | 0.794 |
| levonorgestrel | omeprazole | 0.784 | 0.756 |
| cyproterone | trifluridine | 0.784 | 0.754 |
| cefalexin | propylthiouracil | 0.784 | 0.759 |
| scopolamine | spectinomycin | 0.784 | 0.691 |
| propafenone | syrosingopine | 0.784 | 0.765 |
| medrysone | oxaprozin | 0.784 | 0.746 |
| ivermectin | prenylamine | 0.784 | 0.858 |
| cefazolin | levomepromazine | 0.784 | 0.780 |
| alprenolol | pimozide | 0.784 | 0.726 |
| ethisterone | promazine | 0.784 | 0.764 |
| alimemazine | oxprenolol | 0.784 | 0.714 |
| dextromethorphan | ronidazole | 0.784 | 0.723 |
| etidronic_acid | methylergometrine | 0.784 | 0.809 |
| ivermectin | menadione | 0.784 | 0.893 |
| etidronic_acid | procyclidine | 0.784 | 0.782 |
| buflomedil | sulfapyridine | 0.784 | 0.678 |
| labetalol | mepyramine | 0.784 | 0.751 |
| menadione | rimexolone | 0.784 | 0.783 |
| reserpine | rolitetracycline | 0.784 | 0.698 |
| chlorphenesin | piroxicam | 0.785 | 0.692 |
| fenoprofen | metoprolol | 0.785 | 0.693 |
| droperidol | naftidrofuryl | 0.785 | 0.709 |
| doxylamine | isoxsuprine | 0.785 | 0.716 |
| benperidol | hydralazine | 0.785 | 0.731 |
| carbachol | terguride | 0.785 | 0.768 |
| betahistine | norethisterone | 0.785 | 0.795 |
| etofenamate | methapyrilene | 0.785 | 0.720 |
| dydrogesterone | procyclidine | 0.785 | 0.759 |
| tenoxicam | tolmetin | 0.785 | 0.671 |
| imipramine | menadione | 0.785 | 0.745 |
| hexetidine | prochlorperazine | 0.785 | 0.727 |
| acepromazine | dipyridamole | 0.785 | 0.688 |
| buflomedil | phensuximide | 0.785 | 0.732 |

| | | | |
|---|---|---|---|
| betazole | triamterene | 0.785 | 0.682 |
| dacarbazine | molindone | 0.785 | 0.678 |
| talampicillin | tropicamide | 0.785 | 0.761 |
| clioquinol | econazole | 0.785 | 0.726 |
| dexibuprofen | monobenzone | 0.785 | 0.674 |
| bufexamac | flufenamic_acid | 0.785 | 0.692 |
| benperidol | pentoxyverine | 0.785 | 0.721 |
| phenazopyridine | progesterone | 0.785 | 0.741 |
| dexpanthenol | topiramate | 0.785 | 0.715 |
| benzylpenicillin | menadione | 0.785 | 0.774 |
| amiodarone | monobenzone | 0.785 | 0.778 |
| cefalexin | dextromethorphan | 0.785 | 0.781 |
| spectinomycin | trioxysalen | 0.785 | 0.761 |
| estrone | pralidoxime | 0.785 | 0.723 |
| latamoxef | sulfaguanidine | 0.785 | 0.756 |
| fenoprofen | tyloxapol | 0.785 | 0.875 |
| clioquinol | verteporfin | 0.785 | 0.815 |
| ajmaline | dihydroergocristine | 0.785 | 0.767 |
| cefalotin | flunisolide | 0.785 | 0.720 |
| bromocriptine | dacarbazine | 0.785 | 0.790 |
| diltiazem | verteporfin | 0.785 | 0.799 |
| doxylamine | moroxydine | 0.785 | 0.736 |
| cycloserine | daunorubicin | 0.785 | 0.881 |
| pentoxyverine | promazine | 0.785 | 0.728 |
| promethazine | syrosingopine | 0.785 | 0.818 |
| atovaquone | rimexolone | 0.785 | 0.744 |
| hydralazine | sulfametoxydiazine | 0.785 | 0.682 |
| loxapine | prenylamine | 0.785 | 0.715 |
| (-)-atenolol | cefalotin | 0.785 | 0.703 |
| piromidic_acid | trimetazidine | 0.785 | 0.684 |
| oxymetazoline | tolfenamic_acid | 0.785 | 0.735 |
| etamsylate | meropenem | 0.785 | 0.773 |
| cefepime | physostigmine | 0.785 | 0.769 |
| clomifene | cortisone | 0.785 | 0.784 |
| liothyronine | simvastatin | 0.785 | 0.717 |
| ethosuximide | promethazine | 0.785 | 0.791 |
| iodixanol | tolmetin | 0.785 | 0.856 |
| flunarizine | trichlormethiazide | 0.785 | 0.796 |
| cefalexin | liothyronine | 0.785 | 0.722 |
| papaverine | verteporfin | 0.785 | 0.787 |
| calcium_folinate | indoprofen | 0.785 | 0.709 |
| rimexolone | torasemide | 0.785 | 0.741 |
| flutamide | verteporfin | 0.785 | 0.823 |
| imipenem | norfloxacin | 0.785 | 0.691 |
| piromidic_acid | terconazole | 0.785 | 0.765 |
| mebendazole | semustine | 0.785 | 0.700 |
| furazolidone | nadolol | 0.785 | 0.696 |
| clonidine | sulfinpyrazone | 0.785 | 0.769 |
| meticrane | picotamide | 0.785 | 0.751 |
| tyloxapol | vidarabine | 0.785 | 0.872 |

| | | | |
|---|---|---|---|
| methylergometrine | promazine | 0.785 | 0.669 |
| fusidic_acid | procainamide | 0.785 | 0.768 |
| estriol | scopolamine | 0.785 | 0.726 |
| naloxone | phenoxybenzamine | 0.785 | 0.752 |
| mecamylamine | talampicillin | 0.785 | 0.850 |
| pentoxyverine | verteporfin | 0.785 | 0.812 |
| buflomedil | daunorubicin | 0.785 | 0.694 |
| gefitinib | practolol | 0.785 | 0.686 |
| chlorzoxazone | ifenprodil | 0.785 | 0.800 |
| diltiazem | metamizole_sodium | 0.785 | 0.715 |
| cetirizine | oxetacaine | 0.785 | 0.752 |
| clofazimine | fluspirilene | 0.785 | 0.759 |
| mitoxantrone | piperidolate | 0.785 | 0.771 |
| flufenamic_acid | piracetam | 0.785 | 0.699 |
| bromocriptine | doxazosin | 0.785 | 0.691 |
| colchicine | digoxin | 0.785 | 0.833 |
| meticrane | sulfinpyrazone | 0.785 | 0.756 |
| chlorcyclizine | lanatoside_C | 0.785 | 0.889 |
| cyproterone | metamizole_sodium | 0.785 | 0.706 |
| hydralazine | spectinomycin | 0.785 | 0.758 |
| ioversol | triflusal | 0.785 | 0.774 |
| clidinium_bromide | labetalol | 0.785 | 0.731 |
| ketoprofen | propylthiouracil | 0.785 | 0.692 |
| clomipramine | rolitetracycline | 0.785 | 0.799 |
| ambroxol | benzathine_benzylper | 0.785 | 0.705 |
| loxapine | metergoline | 0.785 | 0.719 |
| etamsylate | trimetazidine | 0.785 | 0.700 |
| (-)-atenolol | terguride | 0.785 | 0.745 |
| omeprazole | trimetazidine | 0.785 | 0.732 |
| succinylsulfathiazole | zuclopenthixol | 0.785 | 0.719 |
| ciclosporin | lomustine | 0.785 | 0.892 |
| emetine | isotretinoin | 0.785 | 0.774 |
| dacarbazine | flumetasone | 0.785 | 0.783 |
| fluphenazine | quinisocaine | 0.785 | 0.686 |
| oxybuprocaine | sulpiride | 0.785 | 0.664 |
| fluocinonide | naloxone | 0.785 | 0.741 |
| etacrynic_acid | vorinostat | 0.785 | 0.686 |
| chlorzoxazone | meptazinol | 0.785 | 0.754 |
| ifosfamide | methazolamide | 0.785 | 0.730 |
| buflomedil | ethotoin | 0.785 | 0.726 |
| calcium_pantothenate | sulfamerazine | 0.785 | 0.805 |
| imipramine | miconazole | 0.785 | 0.668 |
| (-)-atenolol | acetylsalicylic_acid | 0.785 | 0.713 |
| doxazosin | molindone | 0.785 | 0.700 |
| clomipramine | mepacrine | 0.785 | 0.713 |
| reserpine | trimetazidine | 0.785 | 0.784 |
| clomipramine | ivermectin | 0.785 | 0.868 |
| paclitaxel | suloctidil | 0.785 | 0.822 |
| levonorgestrel | paracetamol | 0.785 | 0.752 |
| amiodarone | loperamide | 0.785 | 0.727 |

| | | | |
|---|---|---|---|
| clidinium_bromide | sulfanilamide | 0.785 | 0.730 |
| naloxone | scopolamine | 0.785 | 0.700 |
| griseofulvin | halcinonide | 0.785 | 0.743 |
| levocabastine | proguanil | 0.785 | 0.739 |
| daunorubicin | meclozine | 0.785 | 0.762 |
| carbachol | procaine | 0.785 | 0.688 |
| bisoprolol | buspirone | 0.785 | 0.712 |
| doxazosin | etofylline | 0.785 | 0.691 |
| pimethixene | suloctidil | 0.785 | 0.776 |
| oxprenolol | sulfametoxydiazine | 0.785 | 0.690 |
| trichlormethiazide | urapidil | 0.785 | 0.759 |
| fendiline | rescinnamine | 0.785 | 0.797 |
| ethotoin | tolbutamide | 0.785 | 0.656 |
| meclofenoxate | norfloxacin | 0.785 | 0.673 |
| piperidolate | procaine | 0.785 | 0.680 |
| thioproperazine | topiramate | 0.785 | 0.738 |
| acepromazine | nitrofurantoin | 0.785 | 0.669 |
| meticrane | tobramycin | 0.785 | 0.778 |
| bisacodyl | mitoxantrone | 0.785 | 0.721 |
| oxetacaine | syrosingopine | 0.785 | 0.720 |
| glafenine | levocabastine | 0.785 | 0.710 |
| beclometasone | reserpine | 0.785 | 0.741 |
| oxybuprocaine | ronidazole | 0.785 | 0.707 |
| norethisterone | verteporfin | 0.785 | 0.822 |
| bromocriptine | fluphenazine | 0.785 | 0.704 |
| desoxycortone | ipratropium_bromide | 0.785 | 0.675 |
| alprenolol | methylprednisolone | 0.785 | 0.757 |
| irinotecan | sulfametoxydiazine | 0.785 | 0.766 |
| nafcillin | piromidic_acid | 0.785 | 0.689 |
| meglumine | terguride | 0.785 | 0.773 |
| aminohippuric_acid | butoconazole | 0.785 | 0.748 |
| bisacodyl | primaquine | 0.785 | 0.686 |
| desoxycortone | trioxysalen | 0.785 | 0.752 |
| altretamine | doxorubicin | 0.785 | 0.746 |
| ciclosporin | terconazole | 0.785 | 0.845 |
| omeprazole | verteporfin | 0.785 | 0.797 |
| glibenclamide | sulfathiazole | 0.785 | 0.699 |
| deptropine | pimozide | 0.785 | 0.773 |
| gliclazide | vidarabine | 0.785 | 0.685 |
| amoxapine | trazodone | 0.785 | 0.758 |
| budesonide | gefitinib | 0.785 | 0.761 |
| latamoxef | sulfafurazole | 0.785 | 0.752 |
| dextromethorphan | medrysone | 0.785 | 0.778 |
| bromocriptine | quinisocaine | 0.785 | 0.764 |
| dacarbazine | imipenem | 0.785 | 0.687 |
| aminohippuric_acid | trioxysalen | 0.785 | 0.652 |
| fluorometholone | gliclazide | 0.785 | 0.739 |
| mefloquine | oxyphenbutazone | 0.785 | 0.703 |
| astemizole | sertaconazole | 0.785 | 0.735 |
| alprenolol | tetryzoline | 0.785 | 0.672 |

| | | | |
|---|---|---|---|
| benzocaine | topiramate | 0.785 | 0.774 |
| norethisterone | tridihexethyl | 0.785 | 0.772 |
| liothyronine | thiocolchicoside | 0.785 | 0.724 |
| clomipramine | roxithromycin | 0.785 | 0.857 |
| perphenazine | proscillaridin | 0.785 | 0.760 |
| idoxuridine | methylergometrine | 0.785 | 0.697 |
| milrinone | procyclidine | 0.785 | 0.779 |
| phenoxybenzamine | triamterene | 0.785 | 0.739 |
| carbinoxamine | letrozole | 0.785 | 0.658 |
| levocabastine | moxisylyte | 0.785 | 0.743 |
| acetylsalicylic_acid | meptazinol | 0.785 | 0.712 |
| diazoxide | mefloquine | 0.785 | 0.698 |
| troglitazone | valproic_acid | 0.785 | 0.788 |
| meptazinol | pentoxyverine | 0.785 | 0.741 |
| dipyridamole | trazodone | 0.785 | 0.789 |
| proscillaridin | terconazole | 0.785 | 0.742 |
| papaverine | rimexolone | 0.785 | 0.755 |
| bromopride | clomipramine | 0.785 | 0.719 |
| pivampicillin | verteporfin | 0.785 | 0.779 |
| altretamine | galantamine | 0.785 | 0.729 |
| promazine | sulfamethoxazole | 0.785 | 0.717 |
| cyproheptadine | danazol | 0.785 | 0.755 |
| carmustine | disulfiram | 0.785 | 0.661 |
| propylthiouracil | trimetazidine | 0.785 | 0.695 |
| acetylsalicylic_acid | irinotecan | 0.785 | 0.802 |
| lansoprazole | pivmecillinam | 0.785 | 0.698 |
| propafenone | zomepirac | 0.785 | 0.720 |
| pargyline | piribedil | 0.785 | 0.691 |
| daunorubicin | phenazopyridine | 0.785 | 0.733 |
| saquinavir | verteporfin | 0.785 | 0.752 |
| oxybuprocaine | pheniramine | 0.785 | 0.736 |
| famotidine | zimeldine | 0.785 | 0.772 |
| demecolcine | roxithromycin | 0.785 | 0.815 |
| estrone | sulconazole | 0.785 | 0.710 |
| bromopride | cycloserine | 0.785 | 0.810 |
| bepridil | disulfiram | 0.785 | 0.758 |
| levamisole | nimesulide | 0.785 | 0.682 |
| ethotoin | pramocaine | 0.785 | 0.757 |
| glibenclamide | hydrocortisone | 0.785 | 0.755 |
| doxazosin | isradipine | 0.785 | 0.778 |
| fluphenazine | menadione | 0.785 | 0.740 |
| alprenolol | dacarbazine | 0.785 | 0.719 |
| milrinone | pargyline | 0.785 | 0.717 |
| dextromethorphan | proscillaridin | 0.786 | 0.829 |
| clindamycin | tyloxapol | 0.786 | 0.838 |
| etacrynic_acid | propafenone | 0.786 | 0.734 |
| bupivacaine | diethylcarbamazine | 0.786 | 0.690 |
| (-)-atenolol | etamsylate | 0.786 | 0.731 |
| ceftazidime | chloramphenicol | 0.786 | 0.772 |
| aminophylline | dextromethorphan | 0.786 | 0.745 |

| | | | |
|---|---|---|---|
| nilutamide | omeprazole | 0.786 | 0.698 |
| midecamycin | sulconazole | 0.786 | 0.842 |
| betahistine | ifenprodil | 0.786 | 0.783 |
| cyclobenzaprine | terfenadine | 0.786 | 0.780 |
| ciclopirox | miconazole | 0.786 | 0.744 |
| moxisylyte | sulfaphenazole | 0.786 | 0.739 |
| doxorubicin | oxamic_acid | 0.786 | 0.881 |
| mitoxantrone | piribedil | 0.786 | 0.724 |
| repaglinide | terguride | 0.786 | 0.738 |
| fluphenazine | hexetidine | 0.786 | 0.765 |
| cefalexin | sulfametoxydiazine | 0.786 | 0.659 |
| idoxuridine | vorinostat | 0.786 | 0.747 |
| amoxicillin | omeprazole | 0.786 | 0.662 |
| dacarbazine | metyrapone | 0.786 | 0.699 |
| (-)-catechin | famotidine | 0.786 | 0.698 |
| doxazosin | sulfamethoxazole | 0.786 | 0.753 |
| (-)-atenolol | rimexolone | 0.786 | 0.722 |
| phenazopyridine | raloxifene | 0.786 | 0.728 |
| budesonide | remoxipride | 0.786 | 0.744 |
| fluvoxamine | perphenazine | 0.786 | 0.729 |
| levonorgestrel | phenoxybenzamine | 0.786 | 0.756 |
| albendazole | sirolimus | 0.786 | 0.861 |
| bromperidol | meclofenoxate | 0.786 | 0.651 |
| idoxuridine | trifluoperazine | 0.786 | 0.704 |
| diclofenamide | fluvastatin | 0.786 | 0.722 |
| cefixime | doxazosin | 0.786 | 0.672 |
| piribedil | sulfafurazole | 0.786 | 0.670 |
| etofenamate | pyrazinamide | 0.786 | 0.702 |
| clindamycin | proxymetacaine | 0.786 | 0.731 |
| fluorometholone | phenazopyridine | 0.786 | 0.752 |
| clomifene | phenoxybenzamine | 0.786 | 0.706 |
| butoconazole | carbachol | 0.786 | 0.745 |
| omeprazole | piperidolate | 0.786 | 0.702 |
| (-)-atenolol | meticrane | 0.786 | 0.717 |
| thiocolchicoside | triflusal | 0.786 | 0.787 |
| colecalciferol | hyoscyamine | 0.786 | 0.770 |
| sulfamerazine | verteporfin | 0.786 | 0.825 |
| etamsylate | thiocolchicoside | 0.786 | 0.804 |
| estrone | procainamide | 0.786 | 0.687 |
| naphazoline | theobromine | 0.786 | 0.711 |
| methylergometrine | nialamide | 0.786 | 0.695 |
| menadione | zimeldine | 0.786 | 0.722 |
| cycloserine | oxprenolol | 0.786 | 0.798 |
| cyproheptadine | fluoxetine | 0.786 | 0.726 |
| flufenamic_acid | pentamidine | 0.786 | 0.753 |
| etoposide | oxamic_acid | 0.786 | 0.888 |
| fenoprofen | oxamniquine | 0.786 | 0.715 |
| gliclazide | moxonidine | 0.786 | 0.737 |
| fluspirilene | propylthiouracil | 0.786 | 0.752 |
| sulfafurazole | verteporfin | 0.786 | 0.830 |

| | | | |
|---|---|---|---|
| bromopride | levamisole | 0.786 | 0.687 |
| etidronic_acid | repaglinide | 0.786 | 0.829 |
| molindone | pyrantel | 0.786 | 0.657 |
| azacitidine | trifluoperazine | 0.786 | 0.734 |
| chloramphenicol | milrinone | 0.786 | 0.703 |
| acetylsalicylic_acid | chloramphenicol | 0.786 | 0.706 |
| fluspirilene | piribedil | 0.786 | 0.686 |
| etamsylate | urapidil | 0.786 | 0.760 |
| gefitinib | oxamic_acid | 0.786 | 0.871 |
| alimemazine | metergoline | 0.786 | 0.722 |
| nortriptyline | raloxifene | 0.786 | 0.764 |
| deptropine | proxyphylline | 0.786 | 0.758 |
| amoxapine | clomipramine | 0.786 | 0.698 |
| aminocaproic_acid | ketorolac | 0.786 | 0.668 |
| diazoxide | ivermectin | 0.786 | 0.877 |
| estrone | imipramine | 0.786 | 0.726 |
| galantamine | torasemide | 0.786 | 0.716 |
| dobutamine | methylergometrine | 0.786 | 0.711 |
| ciclopirox | flupentixol | 0.786 | 0.745 |
| felodipine | mebendazole | 0.786 | 0.728 |
| pargyline | triflusal | 0.786 | 0.762 |
| felodipine | metergoline | 0.786 | 0.761 |
| tolbutamide | verteporfin | 0.786 | 0.816 |
| cefalotin | liothyronine | 0.786 | 0.732 |
| deptropine | sulfafurazole | 0.786 | 0.718 |
| disulfiram | isotretinoin | 0.786 | 0.787 |
| desoxycortone | meticrane | 0.786 | 0.738 |
| citiolone | flufenamic_acid | 0.786 | 0.742 |
| dacarbazine | diltiazem | 0.786 | 0.764 |
| meropenem | rolitetracycline | 0.786 | 0.728 |
| econazole | tribenoside | 0.786 | 0.730 |
| labetalol | nortriptyline | 0.786 | 0.763 |
| cetirizine | deferoxamine | 0.786 | 0.815 |
| ronidazole | sertaconazole | 0.786 | 0.758 |
| griseofulvin | ketanserin | 0.786 | 0.688 |
| econazole | pimethixene | 0.786 | 0.712 |
| aminophenazone | dihydroergotamine | 0.786 | 0.799 |
| ethotoin | tinidazole | 0.786 | 0.660 |
| hyoscyamine | tiratricol | 0.786 | 0.738 |
| econazole | felodipine | 0.786 | 0.718 |
| imipenem | omeprazole | 0.786 | 0.720 |
| cefoperazone | metergoline | 0.786 | 0.778 |
| gliclazide | levonorgestrel | 0.786 | 0.792 |
| piperidolate | propafenone | 0.786 | 0.678 |
| betahistine | piribedil | 0.786 | 0.653 |
| (-)-catechin | irinotecan | 0.786 | 0.726 |
| deptropine | pyrazinamide | 0.786 | 0.791 |
| dextromethorphan | prazosin | 0.786 | 0.779 |
| felodipine | natamycin | 0.786 | 0.810 |
| (-)-atenolol | gliclazide | 0.786 | 0.677 |

| | | | |
|---|---|---|---|
| repaglinide | salbutamol | 0.786 | 0.733 |
| perhexiline | proscillaridin | 0.786 | 0.818 |
| capsaicin | trioxysalen | 0.786 | 0.734 |
| bromperidol | tetracaine | 0.786 | 0.689 |
| ambroxol | ofloxacin | 0.786 | 0.661 |
| betahistine | talampicillin | 0.786 | 0.783 |
| amiloride | oxetacaine | 0.786 | 0.792 |
| gliclazide | monobenzone | 0.786 | 0.745 |
| sulconazole | urapidil | 0.786 | 0.752 |
| milrinone | scopolamine | 0.786 | 0.724 |
| oxyphenbutazone | prenylamine | 0.786 | 0.724 |
| doxycycline | pyrithyldione | 0.786 | 0.787 |
| azathioprine | lisuride | 0.786 | 0.711 |
| acepromazine | sulfamethoxypyridazir | 0.786 | 0.722 |
| clonidine | levonorgestrel | 0.786 | 0.792 |
| acetylsalicylic_acid | milrinone | 0.786 | 0.713 |
| betaxolol | diltiazem | 0.786 | 0.758 |
| moroxydine | primidone | 0.786 | 0.652 |
| ketanserin | zidovudine | 0.786 | 0.682 |
| glibenclamide | tolfenamic_acid | 0.786 | 0.754 |
| chlorzoxazone | nialamide | 0.786 | 0.731 |
| buflomedil | levonorgestrel | 0.786 | 0.741 |
| acepromazine | procaine | 0.786 | 0.721 |
| pargyline | ronidazole | 0.786 | 0.706 |
| cefalotin | digoxin | 0.786 | 0.803 |
| clotrimazole | oxybutynin | 0.786 | 0.713 |
| imipenem | pyrazinamide | 0.786 | 0.720 |
| fludroxycortide | molindone | 0.786 | 0.745 |
| betaxolol | etamsylate | 0.786 | 0.754 |
| nitrofurantoin | sulfamethoxazole | 0.786 | 0.653 |
| chlorprothixene | tamoxifen | 0.786 | 0.698 |
| doxazosin | levonorgestrel | 0.786 | 0.762 |
| bepridil | noretynodrel | 0.786 | 0.773 |
| bepridil | isoconazole | 0.786 | 0.690 |
| propylthiouracil | tolazamide | 0.786 | 0.740 |
| rescinnamine | thioproperazine | 0.786 | 0.761 |
| galantamine | theophylline | 0.786 | 0.728 |
| (-)-atenolol | repaglinide | 0.786 | 0.715 |
| bromperidol | cefalexin | 0.786 | 0.682 |
| cefixime | etamsylate | 0.786 | 0.773 |
| daunorubicin | ethotoin | 0.786 | 0.757 |
| memantine | oxamniquine | 0.786 | 0.808 |
| cinoxacin | sulfamethizole | 0.786 | 0.676 |
| econazole | fenbendazole | 0.786 | 0.713 |
| bepridil | nitrofurantoin | 0.786 | 0.800 |
| ipratropium_bromide | vidarabine | 0.786 | 0.738 |
| cetirizine | proscillaridin | 0.786 | 0.743 |
| atovaquone | tyloxapol | 0.786 | 0.862 |
| levomepromazine | penbutolol | 0.786 | 0.676 |
| flunisolide | tyloxapol | 0.786 | 0.844 |

| | | | |
|---|---|---|---|
| halcinonide | metaraminol | 0.786 | 0.788 |
| gliclazide | vinpocetine | 0.786 | 0.743 |
| oxetacaine | pyrimethamine | 0.786 | 0.781 |
| tyloxapol | ursodeoxycholic_acid | 0.786 | 0.839 |
| chlorambucil | pentetrazol | 0.786 | 0.666 |
| doxazosin | fludroxycortide | 0.786 | 0.761 |
| carmustine | dyclonine | 0.786 | 0.683 |
| fenoprofen | propantheline_bromic | 0.786 | 0.694 |
| metacycline | oxprenolol | 0.786 | 0.703 |
| etoposide | exemestane | 0.786 | 0.789 |
| liothyronine | monobenzone | 0.786 | 0.738 |
| bromperidol | meclozine | 0.786 | 0.734 |
| cefazolin | flecainide | 0.786 | 0.727 |
| norethisterone | perhexiline | 0.786 | 0.764 |
| cyclobenzaprine | prochlorperazine | 0.786 | 0.707 |
| promethazine | sulfamethoxazole | 0.786 | 0.739 |
| ifenprodil | monobenzone | 0.786 | 0.687 |
| clenbuterol | loperamide | 0.786 | 0.730 |
| aminophenazone | nicergoline | 0.786 | 0.752 |
| flucytosine | triflusal | 0.786 | 0.689 |
| ethotoin | ipratropium_bromide | 0.786 | 0.691 |
| meropenem | sulfamethoxypyridazir | 0.786 | 0.720 |
| bepridil | repaglinide | 0.786 | 0.752 |
| daunorubicin | propofol | 0.786 | 0.767 |
| chloramphenicol | pyrazinamide | 0.786 | 0.730 |
| fluoxetine | terfenadine | 0.786 | 0.744 |
| antazoline | pimozide | 0.786 | 0.758 |
| (-)-catechin | promethazine | 0.786 | 0.731 |
| phenacetin | sisomicin | 0.786 | 0.815 |
| apomorphine | nicotinic_acid | 0.786 | 0.688 |
| etofylline | hyoscyamine | 0.786 | 0.723 |
| albendazole | latamoxef | 0.786 | 0.762 |
| remoxipride | sulfafurazole | 0.786 | 0.690 |
| bepridil | fenbendazole | 0.786 | 0.724 |
| levamisole | oxprenolol | 0.786 | 0.675 |
| cefalexin | etamsylate | 0.786 | 0.714 |
| calcium_folinate | levomepromazine | 0.786 | 0.776 |
| diflorasone | propylthiouracil | 0.786 | 0.780 |
| pridinol | tyloxapol | 0.786 | 0.874 |
| dirithromycin | propafenone | 0.786 | 0.814 |
| diazoxide | meptazinol | 0.786 | 0.733 |
| omeprazole | procaine | 0.786 | 0.651 |
| milrinone | zimeldine | 0.786 | 0.678 |
| chlorzoxazone | halcinonide | 0.786 | 0.844 |
| capsaicin | triflusal | 0.786 | 0.780 |
| menadione | protriptyline | 0.786 | 0.753 |
| decitabine | isoniazid | 0.786 | 0.654 |
| buflomedil | procyclidine | 0.786 | 0.653 |
| etamsylate | sulfamerazine | 0.787 | 0.654 |
| meclozine | meticrane | 0.787 | 0.790 |

| | | | |
|---|---|---|---|
| levomepromazine | prochlorperazine | 0.787 | 0.670 |
| capsaicin | chlorzoxazone | 0.787 | 0.724 |
| astemizole | thiethylperazine | 0.787 | 0.718 |
| clioquinol | dipyridamole | 0.787 | 0.760 |
| miconazole | oxybuprocaine | 0.787 | 0.745 |
| decitabine | lomustine | 0.787 | 0.705 |
| dirithromycin | piracetam | 0.787 | 0.877 |
| deptropine | phensuximide | 0.787 | 0.743 |
| trioxysalen | ursodeoxycholic_acid | 0.787 | 0.758 |
| galantamine | natamycin | 0.787 | 0.812 |
| astemizole | saquinavir | 0.787 | 0.752 |
| daunorubicin | liothyronine | 0.787 | 0.717 |
| glibenclamide | proxymetacaine | 0.787 | 0.725 |
| amantadine | calcium_pantothenate | 0.787 | 0.857 |
| amiodarone | capsaicin | 0.787 | 0.726 |
| bisacodyl | etacrynic_acid | 0.787 | 0.725 |
| exemestane | midecamycin | 0.787 | 0.837 |
| bezafibrate | triflusal | 0.787 | 0.762 |
| (-)-atenolol | oxantel | 0.787 | 0.712 |
| decitabine | tolnaftate | 0.787 | 0.743 |
| chlortetracycline | pentoxyverine | 0.787 | 0.737 |
| mitoxantrone | tolazoline | 0.787 | 0.798 |
| benzethonium_chloride | suxibuzone | 0.787 | 0.784 |
| ketanserin | zimeldine | 0.787 | 0.754 |
| doxorubicin | oxybuprocaine | 0.787 | 0.738 |
| cefixime | meclofenoxate | 0.787 | 0.710 |
| norfloxacin | oxymetazoline | 0.787 | 0.702 |
| deptropine | phenazopyridine | 0.787 | 0.742 |
| natamycin | trazodone | 0.787 | 0.801 |
| bisacodyl | proxyphylline | 0.787 | 0.749 |
| medrysone | monobenzone | 0.787 | 0.736 |
| ketanserin | oxetacaine | 0.787 | 0.719 |
| flufenamic_acid | ifenprodil | 0.787 | 0.747 |
| isoconazole | metixene | 0.787 | 0.683 |
| dipyridamole | tobramycin | 0.787 | 0.723 |
| nafcillin | sulfathiazole | 0.787 | 0.733 |
| flucytosine | talampicillin | 0.787 | 0.820 |
| carbachol | metergoline | 0.787 | 0.759 |
| ajmaline | danazol | 0.787 | 0.732 |
| clioquinol | isoconazole | 0.787 | 0.695 |
| bromocriptine | cefalexin | 0.787 | 0.744 |
| cefalotin | pargyline | 0.787 | 0.788 |
| carbachol | nitrofurantoin | 0.787 | 0.722 |
| labetalol | phenazopyridine | 0.787 | 0.724 |
| alfaxalone | protriptyline | 0.787 | 0.775 |
| etofenamate | repaglinide | 0.787 | 0.733 |
| azacitidine | oxybuprocaine | 0.787 | 0.719 |
| bufexamac | gliclazide | 0.787 | 0.731 |
| clomipramine | tioguanine | 0.787 | 0.795 |
| dipivefrine | terfenadine | 0.787 | 0.740 |

| | | | |
|---|---|---|---|
| nilutamide | piribedil | 0.787 | 0.710 |
| pralidoxime | tiapride | 0.787 | 0.718 |
| etacrynic_acid | sulfinpyrazone | 0.787 | 0.713 |
| doxorubicin | exemestane | 0.787 | 0.767 |
| physostigmine | racecadotril | 0.787 | 0.743 |
| milrinone | sulfamerazine | 0.787 | 0.712 |
| nialamide | oxaprozin | 0.787 | 0.709 |
| fluorometholone | papaverine | 0.787 | 0.758 |
| etodolac | etofenamate | 0.787 | 0.683 |
| estriol | oxetacaine | 0.787 | 0.773 |
| griseofulvin | procainamide | 0.787 | 0.688 |
| clotrimazole | perhexiline | 0.787 | 0.670 |
| butoconazole | doxazosin | 0.787 | 0.751 |
| lomustine | suloctidil | 0.787 | 0.730 |
| benperidol | tyloxapol | 0.787 | 0.842 |
| fluspirilene | propofol | 0.787 | 0.771 |
| carbimazole | ergocalciferol | 0.787 | 0.747 |
| bupivacaine | dropropizine | 0.787 | 0.705 |
| etofylline | pyrantel | 0.787 | 0.661 |
| ethotoin | promethazine | 0.787 | 0.717 |
| azacitidine | piribedil | 0.787 | 0.711 |
| bisacodyl | buflomedil | 0.787 | 0.751 |
| acetylsalicylic_acid | niridazole | 0.787 | 0.665 |
| praziquantel | vinpocetine | 0.787 | 0.701 |
| nitrofurantoin | pyrazinamide | 0.787 | 0.667 |
| halcinonide | urapidil | 0.787 | 0.760 |
| etofylline | torasemide | 0.787 | 0.721 |
| diltiazem | etofenamate | 0.787 | 0.719 |
| (-)-catechin | gliclazide | 0.787 | 0.704 |
| doxazosin | spectinomycin | 0.787 | 0.769 |
| repaglinide | sulfaphenazole | 0.787 | 0.752 |
| ciclosporin | nortriptyline | 0.787 | 0.903 |
| dobutamine | tribenoside | 0.787 | 0.753 |
| desoxycortone | pyrvinium | 0.787 | 0.797 |
| amikacin | practolol | 0.787 | 0.800 |
| griseofulvin | meptazinol | 0.787 | 0.721 |
| hydralazine | pramocaine | 0.787 | 0.769 |
| carisoprodol | theobromine | 0.787 | 0.760 |
| chlorambucil | glibenclamide | 0.787 | 0.758 |
| carbamazepine | hyoscyamine | 0.787 | 0.714 |
| ivermectin | thioproperazine | 0.787 | 0.808 |
| cycloserine | tropicamide | 0.787 | 0.919 |
| flufenamic_acid | piromidic_acid | 0.787 | 0.659 |
| latamoxef | rifabutin | 0.787 | 0.783 |
| cefamandole | sulfadimethoxine | 0.787 | 0.750 |
| letrozole | mephentermine | 0.787 | 0.734 |
| capsaicin | promethazine | 0.787 | 0.766 |
| cyproterone | meclozine | 0.787 | 0.764 |
| latamoxef | levonorgestrel | 0.787 | 0.789 |
| amoxapine | syrosingopine | 0.787 | 0.815 |

| | | | |
|---|---|---|---|
| dipyridamole | gefitinib | 0.787 | 0.694 |
| captopril | phenazone | 0.787 | 0.693 |
| etoposide | isoconazole | 0.787 | 0.765 |
| cetirizine | proxyphylline | 0.787 | 0.777 |
| methylprednisolone | sulfinpyrazone | 0.787 | 0.728 |
| cefsulodin | simvastatin | 0.787 | 0.765 |
| clomifene | ritodrine | 0.787 | 0.747 |
| ketorolac | tyloxapol | 0.787 | 0.860 |
| clomipramine | noretynodrel | 0.787 | 0.752 |
| fenbufen | flutamide | 0.787 | 0.680 |
| probenecid | spectinomycin | 0.787 | 0.733 |
| deptropine | sulfapyridine | 0.787 | 0.725 |
| pimozide | rimexolone | 0.787 | 0.733 |
| papaverine | trazodone | 0.787 | 0.722 |
| astemizole | felodipine | 0.787 | 0.764 |
| dicoumarol | triflusal | 0.787 | 0.720 |
| acepromazine | altizide | 0.787 | 0.714 |
| aminohippuric_acid | pentoxyverine | 0.787 | 0.748 |
| gefitinib | mifepristone | 0.787 | 0.755 |
| calcium_pantothenate | zimeldine | 0.787 | 0.830 |
| desipramine | rifabutin | 0.787 | 0.843 |
| indapamide | proglumide | 0.787 | 0.706 |
| apomorphine | oleandomycin | 0.787 | 0.849 |
| ambroxol | trifluoperazine | 0.787 | 0.683 |
| clofazimine | cyproheptadine | 0.787 | 0.711 |
| chlorphenesin | cortisone | 0.787 | 0.716 |
| alfuzosin | isoniazid | 0.787 | 0.708 |
| fluspirilene | haloperidol | 0.787 | 0.655 |
| labetalol | liothyronine | 0.787 | 0.716 |
| etofenamate | trazodone | 0.787 | 0.738 |
| bromocriptine | fluoxetine | 0.787 | 0.773 |
| estriol | mepyramine | 0.787 | 0.734 |
| bisacodyl | phenazopyridine | 0.787 | 0.752 |
| (-)-catechin | naloxone | 0.787 | 0.732 |
| dipivefrine | dobutamine | 0.787 | 0.773 |
| niclosamide | propofol | 0.787 | 0.727 |
| cefalexin | cyanocobalamin | 0.787 | 0.850 |
| ketoprofen | prilocaine | 0.787 | 0.667 |
| chlorphenamine | naltrexone | 0.787 | 0.725 |
| latamoxef | liothyronine | 0.787 | 0.742 |
| griseofulvin | omeprazole | 0.787 | 0.665 |
| budesonide | tolfenamic_acid | 0.787 | 0.768 |
| oxetacaine | sulfathiazole | 0.787 | 0.767 |
| nicergoline | phenoxybenzamine | 0.787 | 0.738 |
| amodiaquine | clotrimazole | 0.787 | 0.746 |
| betahistine | bezafibrate | 0.787 | 0.745 |
| dacarbazine | tetryzoline | 0.787 | 0.701 |
| doxazosin | papaverine | 0.787 | 0.671 |
| bufexamac | chlorprothixene | 0.787 | 0.764 |
| azathioprine | perphenazine | 0.787 | 0.734 |

| | | | |
|---|---|---|---|
| cefalexin | nifurtimox | 0.787 | 0.706 |
| moxonidine | proscillaridin | 0.787 | 0.822 |
| fluspirilene | raloxifene | 0.787 | 0.740 |
| chlorprothixene | demecolcine | 0.787 | 0.688 |
| ivermectin | levomepromazine | 0.787 | 0.855 |
| flunisolide | trazodone | 0.787 | 0.770 |
| oxybuprocaine | perhexiline | 0.787 | 0.768 |
| desipramine | flupentixol | 0.787 | 0.676 |
| fipexide | mesoridazine | 0.787 | 0.756 |
| milrinone | sulfamethoxypyridazir | 0.787 | 0.691 |
| ranitidine | triflusal | 0.787 | 0.760 |
| phenoxybenzamine | trioxysalen | 0.787 | 0.767 |
| chloropyramine | piromidic_acid | 0.787 | 0.742 |
| latamoxef | syrosingopine | 0.787 | 0.734 |
| flunarizine | mecamylamine | 0.787 | 0.839 |
| etamsylate | etidronic_acid | 0.787 | 0.656 |
| gefitinib | methylergometrine | 0.787 | 0.667 |
| felodipine | phenoxybenzamine | 0.787 | 0.741 |
| dacarbazine | dipivefrine | 0.787 | 0.747 |
| ronidazole | sulfamerazine | 0.787 | 0.684 |
| metrizamide | trimipramine | 0.787 | 0.785 |
| mefloquine | troglitazone | 0.787 | 0.777 |
| betahistine | piromidic_acid | 0.787 | 0.703 |
| cortisone | repaglinide | 0.787 | 0.732 |
| tiabendazole | verteporfin | 0.787 | 0.795 |
| prochlorperazine | vorinostat | 0.787 | 0.758 |
| clonidine | cyproterone | 0.787 | 0.776 |
| memantine | zuclopenthixol | 0.787 | 0.812 |
| octopamine | remoxipride | 0.787 | 0.723 |
| latamoxef | piribedil | 0.787 | 0.736 |
| procaine | ranitidine | 0.787 | 0.725 |
| flecainide | glycopyrronium_brom | 0.787 | 0.724 |
| chlorambucil | phenelzine | 0.787 | 0.679 |
| capsaicin | promazine | 0.787 | 0.752 |
| acetylsalicylic_acid | scopolamine | 0.787 | 0.695 |
| etomidate | sulfaphenazole | 0.787 | 0.667 |
| etidronic_acid | fenoprofen | 0.787 | 0.768 |
| amiodarone | miconazole | 0.787 | 0.749 |
| chlorambucil | tiapride | 0.787 | 0.724 |
| irinotecan | loperamide | 0.787 | 0.779 |
| glibenclamide | trimetazidine | 0.787 | 0.792 |
| clioquinol | meclozine | 0.787 | 0.756 |
| fipexide | sulfametoxydiazine | 0.787 | 0.722 |
| mefloquine | nicergoline | 0.787 | 0.747 |
| dextromethorphan | tropicamide | 0.787 | 0.721 |
| amoxapine | reserpine | 0.787 | 0.794 |
| acepromazine | cinchocaine | 0.787 | 0.674 |
| dinoprost | metixene | 0.787 | 0.789 |
| metyrapone | repaglinide | 0.787 | 0.761 |
| liothyronine | proxyphylline | 0.787 | 0.701 |

| | | | |
|---|---|---|---|
| irinotecan | xylometazoline | 0.787 | 0.804 |
| oxetacaine | phenazone | 0.787 | 0.782 |
| cinchocaine | cortisone | 0.787 | 0.759 |
| ketanserin | procaine | 0.787 | 0.692 |
| dicoumarol | gliclazide | 0.787 | 0.656 |
| chlorprothixene | fluvastatin | 0.787 | 0.674 |
| dextromethorphan | promethazine | 0.787 | 0.683 |
| nortriptyline | propafenone | 0.787 | 0.716 |
| diprophylline | homatropine | 0.787 | 0.709 |
| cetirizine | fluspirilene | 0.787 | 0.714 |
| latamoxef | ronidazole | 0.787 | 0.778 |
| carbinoxamine | metaraminol | 0.787 | 0.696 |
| cetirizine | sulfaphenazole | 0.787 | 0.747 |
| metamizole_sodium | tranylcypromine | 0.787 | 0.670 |
| lymecycline | roxithromycin | 0.787 | 0.770 |
| gliclazide | ifenprodil | 0.787 | 0.702 |
| oxolinic_acid | triflusal | 0.787 | 0.656 |
| promazine | ursodeoxycholic_acid | 0.787 | 0.804 |
| meclozine | zimeldine | 0.787 | 0.699 |
| imipramine | suloctidil | 0.787 | 0.744 |
| doxazosin | fluvastatin | 0.787 | 0.699 |
| etoposide | protriptyline | 0.787 | 0.791 |
| cetirizine | ioversol | 0.788 | 0.765 |
| gefitinib | sulpiride | 0.788 | 0.714 |
| moxonidine | oxamniquine | 0.788 | 0.675 |
| ceftazidime | trimetazidine | 0.788 | 0.794 |
| levomepromazine | terconazole | 0.788 | 0.784 |
| chlorprothixene | podophyllotoxin | 0.788 | 0.729 |
| chlortetracycline | piperidolate | 0.788 | 0.732 |
| cefalotin | chloramphenicol | 0.788 | 0.729 |
| dipyridamole | oleandomycin | 0.788 | 0.743 |
| clonidine | demecolcine | 0.788 | 0.748 |
| griseofulvin | mebendazole | 0.788 | 0.691 |
| calcium_pantothenate | spectinomycin | 0.788 | 0.781 |
| chlorprothixene | syrosingopine | 0.788 | 0.816 |
| acepromazine | methapyrilene | 0.788 | 0.681 |
| etamsylate | fenoprofen | 0.788 | 0.678 |
| ramipril | verteporfin | 0.788 | 0.765 |
| fluspirilene | metamizole_sodium | 0.788 | 0.700 |
| betaxolol | ipratropium_bromide | 0.788 | 0.709 |
| estriol | hydrocortisone | 0.788 | 0.707 |
| piperidolate | pyrazinamide | 0.788 | 0.770 |
| niclosamide | profenamine | 0.788 | 0.696 |
| thiocolchicoside | xylometazoline | 0.788 | 0.797 |
| flurbiprofen | scopolamine | 0.788 | 0.672 |
| daunorubicin | oxprenolol | 0.788 | 0.721 |
| deferoxamine | roxithromycin | 0.788 | 0.819 |
| diltiazem | norethisterone | 0.788 | 0.767 |
| dirithromycin | talampicillin | 0.788 | 0.781 |
| cefalotin | ifenprodil | 0.788 | 0.700 |

| | | | |
|---|---|---|---|
| etamivan | triflusal | 0.788 | 0.707 |
| meticrane | naproxen | 0.788 | 0.669 |
| ketorolac | ursodeoxycholic_acid | 0.788 | 0.731 |
| acepromazine | aminohippuric_acid | 0.788 | 0.662 |
| galantamine | theobromine | 0.788 | 0.737 |
| gliclazide | pimozide | 0.788 | 0.739 |
| azacitidine | cefalotin | 0.788 | 0.719 |
| droperidol | ketoconazole | 0.788 | 0.723 |
| lynestrenol | miconazole | 0.788 | 0.726 |
| deptropine | meticrane | 0.788 | 0.745 |
| fenofibrate | pinacidil | 0.788 | 0.730 |
| pyrazinamide | zomepirac | 0.788 | 0.725 |
| loxapine | tacrine | 0.788 | 0.712 |
| meticrane | metrifonate | 0.788 | 0.706 |
| nalbuphine | suxibuzone | 0.788 | 0.743 |
| (-)-catechin | etofenamate | 0.788 | 0.701 |
| cetirizine | ornidazole | 0.788 | 0.786 |
| piribedil | sulfathiazole | 0.788 | 0.683 |
| oxprenolol | trimipramine | 0.788 | 0.724 |
| etoposide | monobenzone | 0.788 | 0.807 |
| oxamic_acid | tolfenamic_acid | 0.788 | 0.741 |
| flumetasone | metamizole_sodium | 0.788 | 0.711 |
| deptropine | verteporfin | 0.788 | 0.789 |
| thiocolchicoside | tolfenamic_acid | 0.788 | 0.752 |
| ethotoin | pipemidic_acid | 0.788 | 0.695 |
| meptazinol | perhexiline | 0.788 | 0.723 |
| methylergometrine | probucol | 0.788 | 0.753 |
| methazolamide | scopolamine | 0.788 | 0.728 |
| sulfathiazole | troleandomycin | 0.788 | 0.857 |
| omeprazole | propafenone | 0.788 | 0.687 |
| bisacodyl | hyoscyamine | 0.788 | 0.744 |
| etamsylate | halcinonide | 0.788 | 0.812 |
| clomipramine | danazol | 0.788 | 0.769 |
| clindamycin | labetalol | 0.788 | 0.704 |
| ethosuximide | zimeldine | 0.788 | 0.760 |
| lanatoside_C | perhexiline | 0.788 | 0.884 |
| cinchocaine | meclozine | 0.788 | 0.722 |
| chloramphenicol | urapidil | 0.788 | 0.761 |
| butoconazole | papaverine | 0.788 | 0.703 |
| mometasone | triflupromazine | 0.788 | 0.758 |
| hydrocortisone | natamycin | 0.788 | 0.765 |
| fluspirilene | ivermectin | 0.788 | 0.840 |
| irinotecan | trazodone | 0.788 | 0.729 |
| cetirizine | norfloxacin | 0.788 | 0.739 |
| (-)-atenolol | buspirone | 0.788 | 0.716 |
| podophyllotoxin | tetryzoline | 0.788 | 0.769 |
| dihydroergocristine | mefloquine | 0.788 | 0.769 |
| betaxolol | promethazine | 0.788 | 0.747 |
| oxolinic_acid | sulfaphenazole | 0.788 | 0.675 |
| triflusal | urapidil | 0.788 | 0.758 |

| | | | |
|---|---|---|---|
| colistin | trichlormethiazide | 0.788 | 0.881 |
| rimexolone | zomepirac | 0.788 | 0.735 |
| clidinium_bromide | methazolamide | 0.788 | 0.755 |
| chlortetracycline | paclitaxel | 0.788 | 0.770 |
| estrone | flucytosine | 0.788 | 0.725 |
| methylergometrine | rescinnamine | 0.788 | 0.727 |
| propafenone | pyrvinium | 0.788 | 0.764 |
| (-)-catechin | etamsylate | 0.788 | 0.666 |
| exemestane | kanamycin | 0.788 | 0.781 |
| benzylpenicillin | trazodone | 0.788 | 0.724 |
| cyproterone | ursodeoxycholic_acid | 0.788 | 0.692 |
| aminohippuric_acid | triflusal | 0.788 | 0.688 |
| cefsulodin | piromidic_acid | 0.788 | 0.761 |
| scopolamine | tyloxapol | 0.788 | 0.865 |
| methylergometrine | pyrazinamide | 0.788 | 0.756 |
| pyrazinamide | sulfamethoxypyridazir | 0.788 | 0.698 |
| hydrocortisone | levonorgestrel | 0.788 | 0.652 |
| diltiazem | exemestane | 0.788 | 0.772 |
| ethambutol | trichlormethiazide | 0.788 | 0.721 |
| bufexamac | cortisone | 0.788 | 0.715 |
| fenbufen | sulconazole | 0.788 | 0.726 |
| cloxacillin | tolfenamic_acid | 0.788 | 0.716 |
| cetirizine | piromidic_acid | 0.788 | 0.746 |
| acenocoumarol | topiramate | 0.788 | 0.732 |
| hydralazine | naloxone | 0.788 | 0.732 |
| dacarbazine | irinotecan | 0.788 | 0.775 |
| chlorzoxazone | methocarbamol | 0.788 | 0.701 |
| daunorubicin | promethazine | 0.788 | 0.737 |
| fenbufen | prilocaine | 0.788 | 0.680 |
| natamycin | nitrofurantoin | 0.788 | 0.823 |
| colecalciferol | doxorubicin | 0.788 | 0.788 |
| amiodarone | meptazinol | 0.788 | 0.760 |
| mifepristone | phenoxybenzamine | 0.788 | 0.768 |
| dacarbazine | levonorgestrel | 0.788 | 0.741 |
| irinotecan | trioxysalen | 0.788 | 0.798 |
| clioquinol | monobenzone | 0.788 | 0.671 |
| meropenem | tyloxapol | 0.788 | 0.848 |
| estrone | tyloxapol | 0.788 | 0.883 |
| capsaicin | meclozine | 0.788 | 0.730 |
| hyoscyamine | sulfaphenazole | 0.788 | 0.661 |
| fenoprofen | norfloxacin | 0.788 | 0.701 |
| amiodarone | propylthiouracil | 0.788 | 0.759 |
| budesonide | molsidomine | 0.788 | 0.763 |
| oxamniquine | pentoxyverine | 0.788 | 0.744 |
| piribedil | triflusal | 0.788 | 0.770 |
| azacitidine | benzethonium_chloric | 0.788 | 0.815 |
| cyclopentolate | terbutaline | 0.788 | 0.702 |
| chlorphenesin | tyloxapol | 0.788 | 0.876 |
| etofenamate | flumetasone | 0.788 | 0.725 |
| norethisterone | sulfametoxydiazine | 0.788 | 0.752 |

| | | | |
|---|---|---|---|
| oxetacaine | procainamide | 0.788 | 0.747 |
| mitoxantrone | sulfaphenazole | 0.788 | 0.743 |
| chlorprothixene | droperidol | 0.788 | 0.734 |
| carbachol | cinchocaine | 0.788 | 0.783 |
| fenoprofen | propylthiouracil | 0.788 | 0.694 |
| (-)-catechin | pyrvinium | 0.788 | 0.780 |
| ramipril | ronidazole | 0.788 | 0.777 |
| pargyline | tyloxapol | 0.788 | 0.903 |
| felodipine | roxithromycin | 0.788 | 0.816 |
| antazoline | fipexide | 0.788 | 0.752 |
| amiodarone | sulfafurazole | 0.788 | 0.751 |
| buflomedil | propantheline_bromic | 0.788 | 0.667 |
| benzethonium_chloride | meclozine | 0.788 | 0.712 |
| dihydroergotamine | mometasone | 0.788 | 0.754 |
| meclozine | pyrazinamide | 0.788 | 0.822 |
| atovaquone | quinisocaine | 0.788 | 0.711 |
| dydrogesterone | glimepiride | 0.788 | 0.753 |
| propafenone | scopolamine | 0.788 | 0.708 |
| estrone | metyrapone | 0.788 | 0.679 |
| cetirizine | cycloserine | 0.788 | 0.927 |
| amiodarone | menadione | 0.788 | 0.770 |
| ramipril | ticarcillin | 0.788 | 0.709 |
| hyoscyamine | sulfametoxydiazine | 0.788 | 0.672 |
| butoconazole | troglitazone | 0.788 | 0.768 |
| fluspirilene | imipramine | 0.788 | 0.768 |
| benperidol | gliclazide | 0.788 | 0.698 |
| amoxapine | levomepromazine | 0.788 | 0.684 |
| promethazine | propantheline_bromic | 0.788 | 0.701 |
| cinchocaine | sulfinpyrazone | 0.788 | 0.675 |
| deferoxamine | monobenzone | 0.788 | 0.854 |
| cetirizine | clomipramine | 0.788 | 0.733 |
| quinisocaine | sparteine | 0.788 | 0.745 |
| digoxin | perhexiline | 0.788 | 0.858 |
| benzethonium_chloride | mebendazole | 0.788 | 0.734 |
| amoxicillin | trazodone | 0.788 | 0.728 |
| doxazosin | meptazinol | 0.788 | 0.793 |
| cefmetazole | suxibuzone | 0.788 | 0.735 |
| methazolamide | verteporfin | 0.788 | 0.835 |
| fluvastatin | loperamide | 0.788 | 0.741 |
| talampicillin | trimipramine | 0.788 | 0.802 |
| chlorzoxazone | diltiazem | 0.788 | 0.807 |
| methylergometrine | raloxifene | 0.788 | 0.728 |
| ioxaglic_acid | urapidil | 0.788 | 0.750 |
| flunarizine | sulfafurazole | 0.788 | 0.746 |
| ketoprofen | propofol | 0.788 | 0.679 |
| chlortalidone | ketotifen | 0.788 | 0.738 |
| fluphenazine | pizotifen | 0.788 | 0.720 |
| fenoprofen | rolitetracycline | 0.788 | 0.754 |
| flavoxate | procaine | 0.788 | 0.694 |
| ivermectin | labetalol | 0.788 | 0.826 |

| | | | |
|---|---|---|---|
| galantamine | rescinnamine | 0.788 | 0.797 |
| meticrane | milrinone | 0.788 | 0.709 |
| amiodarone | etamsylate | 0.788 | 0.808 |
| aminohippuric_acid | capsaicin | 0.788 | 0.681 |
| fluvastatin | ioversol | 0.788 | 0.732 |
| norethisterone | sulfamethoxypyridazir | 0.788 | 0.743 |
| prazosin | promethazine | 0.788 | 0.751 |
| doxorubicin | mycophenolic_acid | 0.788 | 0.688 |
| sulfinpyrazone | vinpocetine | 0.788 | 0.705 |
| etamivan | flufenamic_acid | 0.788 | 0.670 |
| repaglinide | trifluridine | 0.788 | 0.770 |
| dextromethorphan | ipratropium_bromide | 0.788 | 0.753 |
| acetylsalicylic_acid | enoxacin | 0.788 | 0.712 |
| dyclonine | semustine | 0.788 | 0.690 |
| latamoxef | sulpiride | 0.788 | 0.764 |
| alfaxalone | procaine | 0.788 | 0.731 |
| aminophylline | nitrendipine | 0.788 | 0.742 |
| lymecycline | proscillaridin | 0.788 | 0.749 |
| nialamide | phenoxybenzamine | 0.788 | 0.741 |
| benperidol | nilutamide | 0.788 | 0.733 |
| hydroquinine | topiramate | 0.788 | 0.726 |
| meticrane | phenoxybenzamine | 0.788 | 0.762 |
| acepromazine | amiodarone | 0.788 | 0.696 |
| clomipramine | irinotecan | 0.788 | 0.792 |
| buflomedil | meclozine | 0.788 | 0.730 |
| miconazole | quinisocaine | 0.788 | 0.690 |
| cetirizine | tranylcypromine | 0.788 | 0.772 |
| diltiazem | nilutamide | 0.788 | 0.738 |
| antazoline | methylprednisolone | 0.788 | 0.757 |
| chlortetracycline | exemestane | 0.788 | 0.765 |
| digoxin | ioversol | 0.788 | 0.821 |
| oxytetracycline | terfenadine | 0.788 | 0.768 |
| molindone | urapidil | 0.788 | 0.728 |
| (-)-catechin | bromperidol | 0.788 | 0.740 |
| clonidine | zomepirac | 0.788 | 0.694 |
| methazolamide | talampicillin | 0.788 | 0.771 |
| menadione | triflusal | 0.788 | 0.662 |
| bromopride | pramocaine | 0.788 | 0.708 |
| amoxicillin | piperidolate | 0.788 | 0.733 |
| oxamniquine | pivampicillin | 0.788 | 0.751 |
| loperamide | proscillaridin | 0.789 | 0.773 |
| chlortetracycline | rimexolone | 0.789 | 0.725 |
| alverine | oxymetazoline | 0.789 | 0.750 |
| bisacodyl | pargyline | 0.789 | 0.741 |
| estriol | methazolamide | 0.789 | 0.719 |
| glibenclamide | menadione | 0.789 | 0.788 |
| griseofulvin | pyrazinamide | 0.789 | 0.732 |
| cefalexin | repaglinide | 0.789 | 0.738 |
| ioversol | pyrantel | 0.789 | 0.834 |
| benzydamine | mifepristone | 0.789 | 0.764 |

| | | | |
|---|---|---|---|
| imipenem | ipratropium_bromide | 0.789 | 0.732 |
| demecolcine | oxybuprocaine | 0.789 | 0.726 |
| podophyllotoxin | thiamphenicol | 0.789 | 0.700 |
| azacitidine | butoconazole | 0.789 | 0.780 |
| mepyramine | oxprenolol | 0.789 | 0.698 |
| fipexide | labetalol | 0.789 | 0.688 |
| citiolone | hydralazine | 0.789 | 0.672 |
| amiloride | troleandomycin | 0.789 | 0.869 |
| cefalexin | pargyline | 0.789 | 0.760 |
| estriol | piribedil | 0.789 | 0.725 |
| saquinavir | trifluoperazine | 0.789 | 0.768 |
| rescinnamine | rifabutin | 0.789 | 0.765 |
| promethazine | repaglinide | 0.789 | 0.776 |
| oxamic_acid | vinpocetine | 0.789 | 0.874 |
| thiocolchicoside | verteporfin | 0.789 | 0.729 |
| hydrocortisone | trimetazidine | 0.789 | 0.738 |
| bromocriptine | ethotoin | 0.789 | 0.788 |
| cyproterone | flufenamic_acid | 0.789 | 0.723 |
| cefamandole | ofloxacin | 0.789 | 0.701 |
| carbachol | pridinol | 0.789 | 0.757 |
| calcium_pantothenate | cefalexin | 0.789 | 0.772 |
| benzbromarone | diloxanide | 0.789 | 0.695 |
| ifosfamide | quinidine | 0.789 | 0.701 |
| calcium_pantothenate | meticrane | 0.789 | 0.785 |
| roxithromycin | tranylcypromine | 0.789 | 0.894 |
| cefoperazone | podophyllotoxin | 0.789 | 0.752 |
| etacrynic_acid | rifabutin | 0.789 | 0.815 |
| metergoline | thiethylperazine | 0.789 | 0.683 |
| theobromine | trimipramine | 0.789 | 0.788 |
| sulconazole | trimipramine | 0.789 | 0.698 |
| meclofenoxate | roxithromycin | 0.789 | 0.850 |
| dextromethorphan | propafenone | 0.789 | 0.762 |
| naloxone | verteporfin | 0.789 | 0.829 |
| albendazole | podophyllotoxin | 0.789 | 0.745 |
| (-)-atenolol | meglumine | 0.789 | 0.744 |
| phenazopyridine | tetryzoline | 0.789 | 0.681 |
| levodopa | remoxipride | 0.789 | 0.729 |
| methoxamine | omeprazole | 0.789 | 0.695 |
| cycloserine | propafenone | 0.789 | 0.855 |
| chlorzoxazone | daunorubicin | 0.789 | 0.797 |
| haloperidol | reserpine | 0.789 | 0.703 |
| perphenazine | verapamil | 0.789 | 0.737 |
| flufenamic_acid | irinotecan | 0.789 | 0.752 |
| bepridil | bromperidol | 0.789 | 0.760 |
| medrysone | procyclidine | 0.789 | 0.749 |
| galantamine | guanfacine | 0.789 | 0.718 |
| remoxipride | sulfamerazine | 0.789 | 0.675 |
| nitrofurantoin | trichlormethiazide | 0.789 | 0.696 |
| ganciclovir | sulfaphenazole | 0.789 | 0.670 |
| danazol | famotidine | 0.789 | 0.742 |

| | | | |
|---|---|---|---|
| bufexamac | carbachol | 0.789 | 0.714 |
| colchicine | protriptyline | 0.789 | 0.723 |
| estriol | halcinonide | 0.789 | 0.721 |
| metyrapone | piromidic_acid | 0.789 | 0.664 |
| roxithromycin | sulfafurazole | 0.789 | 0.851 |
| etomidate | zimeldine | 0.789 | 0.684 |
| amphotericin_B | vigabatrin | 0.789 | 0.905 |
| fluspirilene | repaglinide | 0.789 | 0.716 |
| capsaicin | proxyphylline | 0.789 | 0.691 |
| chlorprothixene | debrisoquine | 0.789 | 0.690 |
| aminohippuric_acid | colecalciferol | 0.789 | 0.777 |
| albendazole | antazoline | 0.789 | 0.726 |
| amiodarone | ticlopidine | 0.789 | 0.769 |
| hydrocortisone | levamisole | 0.789 | 0.792 |
| bromperidol | dacarbazine | 0.789 | 0.770 |
| azacitidine | miconazole | 0.789 | 0.786 |
| etofenamate | ganciclovir | 0.789 | 0.686 |
| piracetam | sulconazole | 0.789 | 0.800 |
| flucytosine | sulconazole | 0.789 | 0.829 |
| pyrantel | triflusal | 0.789 | 0.728 |
| alimemazine | repaglinide | 0.789 | 0.761 |
| fluvastatin | lymecycline | 0.789 | 0.762 |
| estrone | meptazinol | 0.789 | 0.693 |
| isotretinoin | terfenadine | 0.789 | 0.770 |
| aminohippuric_acid | piperidolate | 0.789 | 0.727 |
| chlorzoxazone | iopanoic_acid | 0.789 | 0.701 |
| famotidine | vinpocetine | 0.789 | 0.783 |
| dorzolamide | saquinavir | 0.789 | 0.794 |
| sulconazole | tolfenamic_acid | 0.789 | 0.700 |
| aminocaproic_acid | sulconazole | 0.789 | 0.805 |
| griseofulvin | hydralazine | 0.789 | 0.726 |
| deferoxamine | fenoprofen | 0.789 | 0.845 |
| cinnarizine | diazoxide | 0.789 | 0.806 |
| flupentixol | lymecycline | 0.789 | 0.767 |
| acepromazine | doxorubicin | 0.789 | 0.706 |
| betahistine | naloxone | 0.789 | 0.776 |
| glibenclamide | zidovudine | 0.789 | 0.763 |
| (-)-atenolol | tobramycin | 0.789 | 0.766 |
| bemegride | cefalexin | 0.789 | 0.771 |
| lomefloxacin | tobramycin | 0.789 | 0.734 |
| capsaicin | meptazinol | 0.789 | 0.769 |
| fenoprofen | pyrazinamide | 0.789 | 0.726 |
| iohexol | sulfapyridine | 0.789 | 0.796 |
| glibenclamide | latamoxef | 0.789 | 0.744 |
| fenspiride | zimeldine | 0.789 | 0.710 |
| lymecycline | urapidil | 0.789 | 0.760 |
| bupropion | rimexolone | 0.789 | 0.737 |
| bromperidol | liothyronine | 0.789 | 0.728 |
| sulconazole | torasemide | 0.789 | 0.728 |
| amoxapine | oxetacaine | 0.789 | 0.745 |

| | | | |
|---|---|---|---|
| levamisole | progesterone | 0.789 | 0.780 |
| torasemide | trioxysalen | 0.789 | 0.708 |
| acetylsalicylic_acid | cinchocaine | 0.789 | 0.714 |
| fluvastatin | tetryzoline | 0.789 | 0.750 |
| metacycline | norethisterone | 0.789 | 0.725 |
| budesonide | doxorubicin | 0.789 | 0.743 |
| liothyronine | menadione | 0.789 | 0.780 |
| prenylamine | raloxifene | 0.789 | 0.758 |
| metergoline | trazodone | 0.789 | 0.654 |
| omeprazole | perhexiline | 0.789 | 0.770 |
| ciclosporin | disulfiram | 0.789 | 0.882 |
| decitabine | diclofenamide | 0.789 | 0.681 |
| benfluorex | colchicine | 0.789 | 0.742 |
| ioxaglic_acid | meticrane | 0.789 | 0.809 |
| capsaicin | glibenclamide | 0.789 | 0.744 |
| cycloserine | triflusal | 0.789 | 0.740 |
| deptropine | ranitidine | 0.789 | 0.777 |
| tolbutamide | tropicamide | 0.789 | 0.674 |
| daunorubicin | methazolamide | 0.789 | 0.730 |
| iopanoic_acid | propafenone | 0.789 | 0.720 |
| progesterone | syrosingopine | 0.789 | 0.794 |
| cefalotin | clonidine | 0.789 | 0.782 |
| altretamine | niridazole | 0.789 | 0.660 |
| hydrocortisone | propafenone | 0.789 | 0.737 |
| ioversol | tyloxapol | 0.789 | 0.826 |
| adipiodone | aminocaproic_acid | 0.789 | 0.841 |
| clomipramine | praziquantel | 0.789 | 0.712 |
| meptazinol | nalbuphine | 0.789 | 0.685 |
| amiodarone | tropicamide | 0.789 | 0.764 |
| lovastatin | podophyllotoxin | 0.789 | 0.716 |
| trichlormethiazide | trimipramine | 0.789 | 0.773 |
| methylergometrine | oxybuprocaine | 0.789 | 0.700 |
| ceftazidime | pivampicillin | 0.789 | 0.696 |
| norethisterone | pyrazinamide | 0.789 | 0.792 |
| chlorzoxazone | ketoprofen | 0.789 | 0.713 |
| fendiline | labetalol | 0.789 | 0.719 |
| isoconazole | lanatoside_C | 0.789 | 0.875 |
| clomipramine | zomepirac | 0.789 | 0.746 |
| lynestrenol | mefloquine | 0.789 | 0.755 |
| cinchocaine | clomipramine | 0.789 | 0.737 |
| cyclizine | flucloxacillin | 0.789 | 0.764 |
| benfluorex | fenoprofen | 0.789 | 0.716 |
| imipenem | metyrapone | 0.789 | 0.725 |
| bisacodyl | oxaprozin | 0.789 | 0.672 |
| tenoxicam | trimethadione | 0.789 | 0.796 |
| lanatoside_C | thioridazine | 0.789 | 0.879 |
| metyrapone | nitrofurantoin | 0.789 | 0.671 |
| etamsylate | tropicamide | 0.789 | 0.742 |
| benzylpenicillin | ipratropium_bromide | 0.789 | 0.664 |
| pargyline | propylthiouracil | 0.789 | 0.694 |

| | | | |
|---|---|---|---|
| famotidine | fipexide | 0.789 | 0.729 |
| bepridil | estriol | 0.789 | 0.766 |
| ivermectin | tamoxifen | 0.789 | 0.849 |
| methazolamide | propafenone | 0.789 | 0.752 |
| bepridil | oxprenolol | 0.789 | 0.732 |
| piromidic_acid | urapidil | 0.789 | 0.664 |
| pyrvinium | tamoxifen | 0.789 | 0.775 |
| medrysone | phenformin | 0.789 | 0.747 |
| betahistine | mitoxantrone | 0.789 | 0.767 |
| bromocriptine | monobenzone | 0.789 | 0.812 |
| astemizole | propafenone | 0.789 | 0.724 |
| amoxicillin | sulfaphenazole | 0.789 | 0.699 |
| amoxapine | labetalol | 0.789 | 0.722 |
| digoxin | profenamine | 0.789 | 0.867 |
| doxorubicin | selegiline | 0.789 | 0.782 |
| suxibuzone | triflusal | 0.789 | 0.774 |
| benzethonium_chloride | raloxifene | 0.789 | 0.751 |
| sulfamethoxypyridazine | trimetazidine | 0.789 | 0.708 |
| lomustine | prenylamine | 0.789 | 0.746 |
| amoxapine | pimozide | 0.789 | 0.763 |
| natamycin | sulfaphenazole | 0.789 | 0.822 |
| meticrane | moxonidine | 0.789 | 0.688 |
| betaxolol | enoxacin | 0.789 | 0.699 |
| betaxolol | fenoprofen | 0.789 | 0.717 |
| azacitidine | ethotoin | 0.789 | 0.668 |
| paromomycin | piromidic_acid | 0.789 | 0.815 |
| flufenamic_acid | flunarizine | 0.789 | 0.766 |
| remoxipride | sulfametoxydiazine | 0.789 | 0.684 |
| benzethonium_chloride | isoconazole | 0.789 | 0.758 |
| digoxin | perphenazine | 0.789 | 0.832 |
| amphotericin_B | hexetidine | 0.789 | 0.857 |
| oxybuprocaine | triflusal | 0.789 | 0.731 |
| metamizole_sodium | nabumetone | 0.789 | 0.667 |
| phenindione | sulconazole | 0.789 | 0.748 |
| amiodarone | piromidic_acid | 0.789 | 0.696 |
| meticrane | procyclidine | 0.789 | 0.742 |
| melatonin | naltrexone | 0.789 | 0.711 |
| amoxicillin | metolazone | 0.789 | 0.684 |
| cetirizine | phenazopyridine | 0.789 | 0.755 |
| midecamycin | oxamic_acid | 0.789 | 0.928 |
| guanethidine | roxithromycin | 0.789 | 0.870 |
| procyclidine | reserpine | 0.789 | 0.792 |
| flufenamic_acid | pentoxyverine | 0.789 | 0.722 |
| dilazep | mefloquine | 0.789 | 0.801 |
| fluorometholone | metacycline | 0.789 | 0.710 |
| clopamide | nitrendipine | 0.789 | 0.711 |
| metacycline | zomepirac | 0.789 | 0.672 |
| cefalexin | piromidic_acid | 0.789 | 0.683 |
| chenodeoxycholic_acid | topiramate | 0.789 | 0.780 |
| chloramphenicol | ifenprodil | 0.789 | 0.721 |

| | | | |
|---|---|---|---|
| amoxicillin | verteporfin | 0.789 | 0.799 |
| cefaclor | tobramycin | 0.789 | 0.735 |
| amitriptyline | econazole | 0.789 | 0.708 |
| monobenzone | repaglinide | 0.789 | 0.794 |
| calcium_pantothenate | ronidazole | 0.789 | 0.820 |
| gemfibrozil | pivampicillin | 0.789 | 0.736 |
| ganciclovir | meticrane | 0.789 | 0.672 |
| metyrapone | pyrvinium | 0.789 | 0.794 |
| chlorzoxazone | ranitidine | 0.789 | 0.745 |
| mitoxantrone | trazodone | 0.789 | 0.751 |
| felodipine | ioversol | 0.789 | 0.780 |
| clomifene | droperidol | 0.789 | 0.745 |
| glibenclamide | methylprednisolone | 0.789 | 0.765 |
| tobramycin | triflusal | 0.789 | 0.774 |
| gemfibrozil | mafenide | 0.789 | 0.742 |
| bisacodyl | ioversol | 0.789 | 0.755 |
| labetalol | syrosingopine | 0.789 | 0.743 |
| clindamycin | doxazosin | 0.789 | 0.730 |
| chlorprothixene | raloxifene | 0.789 | 0.734 |
| flufenamic_acid | mesoridazine | 0.789 | 0.671 |
| roxithromycin | sulfaphenazole | 0.789 | 0.833 |
| chlorzoxazone | torasemide | 0.789 | 0.753 |
| quinisocaine | suloctidil | 0.789 | 0.726 |
| methylergometrine | metyrapone | 0.789 | 0.716 |
| diltiazem | fluspirilene | 0.789 | 0.744 |
| albendazole | clotrimazole | 0.789 | 0.771 |
| atovaquone | nitrofurantoin | 0.789 | 0.671 |
| moxonidine | repaglinide | 0.789 | 0.753 |
| methylergometrine | salbutamol | 0.789 | 0.721 |
| (-)-catechin | mefloquine | 0.789 | 0.710 |
| dipyridamole | rolitetracycline | 0.789 | 0.776 |
| etofylline | gemfibrozil | 0.790 | 0.692 |
| ifenprodil | oxamniquine | 0.790 | 0.708 |
| amoxicillin | metyrapone | 0.790 | 0.736 |
| fluoxetine | hexetidine | 0.790 | 0.754 |
| calcium_pantothenate | diltiazem | 0.790 | 0.792 |
| danazol | miconazole | 0.790 | 0.724 |
| latamoxef | phenazopyridine | 0.790 | 0.780 |
| carbachol | simvastatin | 0.790 | 0.800 |
| imipenem | repaglinide | 0.790 | 0.767 |
| dihydroergocristine | dyclonine | 0.790 | 0.767 |
| meclofenoxate | tyloxapol | 0.790 | 0.871 |
| reserpine | rifabutin | 0.790 | 0.768 |
| ketanserin | procyclidine | 0.790 | 0.740 |
| flunarizine | sulfametoxydiazine | 0.790 | 0.753 |
| cefsulodin | roxithromycin | 0.790 | 0.788 |
| liothyronine | nalidixic_acid | 0.790 | 0.709 |
| butoconazole | melatonin | 0.790 | 0.727 |
| bepridil | rifabutin | 0.790 | 0.834 |
| maprotiline | miconazole | 0.790 | 0.661 |

| | | | |
|---|---|---|---|
| artemisinin | triflusal | 0.790 | 0.730 |
| amoxicillin | buflomedil | 0.790 | 0.720 |
| acepromazine | flufenamic_acid | 0.790 | 0.667 |
| cyproheptadine | dipyridamole | 0.790 | 0.746 |
| albendazole | mifepristone | 0.790 | 0.796 |
| flunarizine | hyoscyamine | 0.790 | 0.770 |
| deferoxamine | doxazosin | 0.790 | 0.772 |
| ethotoin | promazine | 0.790 | 0.699 |
| bezafibrate | latamoxef | 0.790 | 0.720 |
| atovaquone | sulfametoxydiazine | 0.790 | 0.669 |
| cyproterone | meropenem | 0.790 | 0.719 |
| clomifene | mebendazole | 0.790 | 0.704 |
| metixene | suloctidil | 0.790 | 0.750 |
| buspirone | clomifene | 0.790 | 0.748 |
| exemestane | pimozide | 0.790 | 0.758 |
| bambuterol | gefitinib | 0.790 | 0.758 |
| etofylline | pentetrazol | 0.790 | 0.678 |
| metyrapone | talampicillin | 0.790 | 0.744 |
| ethambutol | oxybuprocaine | 0.790 | 0.753 |
| estriol | propafenone | 0.790 | 0.743 |
| bisacodyl | deptropine | 0.790 | 0.745 |
| milrinone | noscapine | 0.790 | 0.715 |
| lymecycline | ronidazole | 0.790 | 0.804 |
| danazol | rimexolone | 0.790 | 0.665 |
| famotidine | spironolactone | 0.790 | 0.765 |
| naloxone | roxithromycin | 0.790 | 0.830 |
| chlorambucil | phenoxybenzamine | 0.790 | 0.742 |
| methylergometrine | tetryzoline | 0.790 | 0.725 |
| chlorambucil | metamizole_sodium | 0.790 | 0.682 |
| propofol | tyloxapol | 0.790 | 0.893 |
| betaxolol | norfloxacin | 0.790 | 0.682 |
| hydralazine | thiocolchicoside | 0.790 | 0.816 |
| lymecycline | natamycin | 0.790 | 0.770 |
| budesonide | tyloxapol | 0.790 | 0.843 |
| bisacodyl | cycloserine | 0.790 | 0.882 |
| benfluorex | oxybutynin | 0.790 | 0.737 |
| cyanocobalamin | etidronic_acid | 0.790 | 0.918 |
| tobramycin | trioxysalen | 0.790 | 0.822 |
| dopamine | lisuride | 0.790 | 0.747 |
| omeprazole | phenoxybenzamine | 0.790 | 0.691 |
| bendroflumethiazide | deferoxamine | 0.790 | 0.802 |
| hydralazine | phenformin | 0.790 | 0.691 |
| benperidol | spectinomycin | 0.790 | 0.777 |
| bufexamac | cyproheptadine | 0.790 | 0.781 |
| fenspiride | sulfamethoxypyridazir | 0.790 | 0.683 |
| benperidol | sulfamethoxypyridazir | 0.790 | 0.729 |
| metacycline | pentetrazol | 0.790 | 0.804 |
| metyrapone | trazodone | 0.790 | 0.732 |
| fenspiride | proxyphylline | 0.790 | 0.732 |
| benzylpenicillin | sulfamethoxypyridazir | 0.790 | 0.687 |

| | | | |
|---|---|---|---|
| molindone | pentamidine | 0.790 | 0.743 |
| fenoterol | tyloxapol | 0.790 | 0.860 |
| estriol | ethotoin | 0.790 | 0.703 |
| isoetarine | zomepirac | 0.790 | 0.717 |
| meropenem | tobramycin | 0.790 | 0.721 |
| prilocaine | trimethoprim | 0.790 | 0.705 |
| ketanserin | oxybuprocaine | 0.790 | 0.729 |
| doxazosin | fluvoxamine | 0.790 | 0.702 |
| clomipramine | haloperidol | 0.790 | 0.774 |
| chlorzoxazone | metyrapone | 0.790 | 0.705 |
| flunarizine | tyloxapol | 0.790 | 0.847 |
| aminocaproic_acid | proxymetacaine | 0.790 | 0.767 |
| famotidine | trimethadione | 0.790 | 0.782 |
| bisacodyl | perhexiline | 0.790 | 0.740 |
| estriol | naloxone | 0.790 | 0.704 |
| natamycin | propafenone | 0.790 | 0.791 |
| nadolol | podophyllotoxin | 0.790 | 0.743 |
| betaxolol | oxybuprocaine | 0.790 | 0.705 |
| ipratropium_bromide | tropicamide | 0.790 | 0.678 |
| azacitidine | metyrapone | 0.790 | 0.667 |
| bumetanide | clemastine | 0.790 | 0.744 |
| fenoprofen | sulfapyridine | 0.790 | 0.677 |
| bendroflumethiazide | flecainide | 0.790 | 0.703 |
| exemestane | ketanserin | 0.790 | 0.724 |
| perhexiline | rimexolone | 0.790 | 0.754 |
| prednisone | pyrimethamine | 0.790 | 0.745 |
| ifenprodil | omeprazole | 0.790 | 0.712 |
| acetylsalicylic_acid | oxolinic_acid | 0.790 | 0.658 |
| betaxolol | latamoxef | 0.790 | 0.756 |
| midodrine | tranexamic_acid | 0.790 | 0.731 |
| fipexide | oxprenolol | 0.790 | 0.714 |
| bezafibrate | repaglinide | 0.790 | 0.714 |
| noretynodrel | triflupromazine | 0.790 | 0.745 |
| chloramphenicol | trazodone | 0.790 | 0.731 |
| pergolide | prochlorperazine | 0.790 | 0.703 |
| lanatoside_C | perphenazine | 0.790 | 0.853 |
| demecolcine | tribenoside | 0.790 | 0.707 |
| cefalexin | tranylcypromine | 0.790 | 0.711 |
| midecamycin | sulfamethoxypyridazir | 0.790 | 0.839 |
| bromocriptine | glafenine | 0.790 | 0.699 |
| levamisole | papaverine | 0.790 | 0.725 |
| aminocaproic_acid | deptropine | 0.790 | 0.814 |
| bromopride | urapidil | 0.790 | 0.682 |
| flurbiprofen | trioxysalen | 0.790 | 0.656 |
| cyproheptadine | quinisocaine | 0.790 | 0.670 |
| clomipramine | diltiazem | 0.790 | 0.725 |
| astemizole | clonidine | 0.790 | 0.781 |
| ranitidine | sulfamethoxypyridazir | 0.790 | 0.740 |
| etamsylate | lymecycline | 0.790 | 0.775 |
| liothyronine | pargyline | 0.790 | 0.769 |

| | | | |
|---|---|---|---|
| econazole | maprotiline | 0.790 | 0.656 |
| colchicine | dobutamine | 0.790 | 0.755 |
| isoconazole | loperamide | 0.790 | 0.740 |
| buflomedil | dextromethorphan | 0.790 | 0.756 |
| sulfafurazole | zimeldine | 0.790 | 0.679 |
| gliclazide | ioxaglic_acid | 0.790 | 0.786 |
| norethisterone | talampicillin | 0.790 | 0.774 |
| (-)-catechin | pyrimethamine | 0.790 | 0.656 |
| protriptyline | verteporfin | 0.790 | 0.817 |
| fenoprofen | molindone | 0.790 | 0.668 |
| acetylsalicylic_acid | carbachol | 0.790 | 0.692 |
| ritodrine | tyloxapol | 0.790 | 0.866 |
| cefalexin | methoxamine | 0.790 | 0.711 |
| galantamine | metyrapone | 0.790 | 0.675 |
| artemisinin | prenylamine | 0.790 | 0.778 |
| gliclazide | zomepirac | 0.790 | 0.687 |
| clonidine | terfenadine | 0.790 | 0.795 |
| dexibuprofen | diltiazem | 0.790 | 0.753 |
| norethisterone | piromidic_acid | 0.790 | 0.724 |
| buflomedil | monobenzone | 0.790 | 0.716 |
| phenazone | trifluoperazine | 0.790 | 0.737 |
| bupivacaine | pinacidil | 0.790 | 0.720 |
| doxepin | pinacidil | 0.790 | 0.689 |
| iocetamic_acid | terconazole | 0.790 | 0.737 |
| chloramphenicol | letrozole | 0.790 | 0.692 |
| paclitaxel | promethazine | 0.790 | 0.837 |
| etacrynic_acid | mepyramine | 0.790 | 0.710 |
| isradipine | nomegestrol | 0.790 | 0.746 |
| buflomedil | sulfafurazole | 0.790 | 0.671 |
| amoxicillin | doxazosin | 0.790 | 0.720 |
| aminohippuric_acid | remoxipride | 0.790 | 0.687 |
| ethambutol | oxamniquine | 0.790 | 0.716 |
| ketoconazole | ritodrine | 0.790 | 0.748 |
| cefalexin | triflusal | 0.790 | 0.749 |
| phenoxybenzamine | rimexolone | 0.790 | 0.762 |
| etofylline | sulfaphenazole | 0.790 | 0.707 |
| clindamycin | ticarcillin | 0.790 | 0.715 |
| doxazosin | paracetamol | 0.790 | 0.741 |
| ethotoin | flufenamic_acid | 0.790 | 0.682 |
| procaine | trioxysalen | 0.790 | 0.711 |
| etacrynic_acid | exemestane | 0.790 | 0.691 |
| bumetanide | diethylcarbamazine | 0.790 | 0.767 |
| dipyridamole | ketanserin | 0.790 | 0.753 |
| etofylline | sulfametoxydiazine | 0.790 | 0.680 |
| bepridil | glibenclamide | 0.790 | 0.773 |
| clotrimazole | mometasone | 0.790 | 0.780 |
| dextromethorphan | verteporfin | 0.790 | 0.845 |
| syrosingopine | tribenoside | 0.790 | 0.756 |
| chlorzoxazone | cyproterone | 0.790 | 0.805 |
| ipratropium_bromide | piperidolate | 0.790 | 0.660 |

| | | | |
|---|---|---|---|
| daunorubicin | tolfenamic_acid | 0.790 | 0.723 |
| cefalotin | oxantel | 0.790 | 0.760 |
| alprostadil | clenbuterol | 0.790 | 0.765 |
| etofenamate | tetryzoline | 0.790 | 0.742 |
| fludroxycortide | nitrofurantoin | 0.790 | 0.747 |
| naftifine | niclosamide | 0.790 | 0.704 |
| loxapine | phenoxybenzamine | 0.790 | 0.700 |
| deptropine | etofenamate | 0.790 | 0.720 |
| galantamine | liothyronine | 0.790 | 0.750 |
| phenoxybenzamine | piribedil | 0.790 | 0.701 |
| butoconazole | menadione | 0.790 | 0.764 |
| chlorcyclizine | paroxetine | 0.790 | 0.748 |
| hydrocortisone | hyoscyamine | 0.790 | 0.710 |
| clindamycin | meropenem | 0.790 | 0.711 |
| pivampicillin | risperidone | 0.790 | 0.712 |
| repaglinide | spectinomycin | 0.790 | 0.778 |
| cefalotin | miconazole | 0.790 | 0.778 |
| astemizole | loxapine | 0.790 | 0.721 |
| dienestrol | sulfametoxydiazine | 0.790 | 0.688 |
| deptropine | tranylcypromine | 0.790 | 0.755 |
| betahistine | chlortetracycline | 0.790 | 0.773 |
| benzylpenicillin | natamycin | 0.790 | 0.794 |
| glafenine | monobenzone | 0.790 | 0.735 |
| phenindione | tyloxapol | 0.790 | 0.885 |
| chlorphenamine | disulfiram | 0.790 | 0.731 |
| fenoprofen | pyrvinium | 0.790 | 0.788 |
| etofenamate | oxolinic_acid | 0.790 | 0.685 |
| cefixime | guanethidine | 0.790 | 0.782 |
| exemestane | racecadotril | 0.790 | 0.768 |
| milrinone | tranylcypromine | 0.790 | 0.729 |
| proxyphylline | tranylcypromine | 0.790 | 0.663 |
| mebendazole | mepacrine | 0.790 | 0.693 |
| monobenzone | zidovudine | 0.790 | 0.672 |
| cortisone | etamsylate | 0.790 | 0.774 |
| chloramphenicol | riluzole | 0.790 | 0.672 |
| bepridil | latamoxef | 0.790 | 0.793 |
| halcinonide | liothyronine | 0.790 | 0.738 |
| minoxidil | tolnaftate | 0.790 | 0.727 |
| ivermectin | sirolimus | 0.790 | 0.774 |
| clindamycin | methazolamide | 0.790 | 0.752 |
| amoxapine | quinisocaine | 0.790 | 0.689 |
| oxaprozin | spectinomycin | 0.790 | 0.740 |
| azacitidine | ticlopidine | 0.790 | 0.741 |
| metyrapone | nialamide | 0.790 | 0.696 |
| benzethonium_chloride | rifabutin | 0.790 | 0.826 |
| cortisone | levamisole | 0.790 | 0.777 |
| proxymetacaine | triflusal | 0.790 | 0.698 |
| imipenem | levamisole | 0.790 | 0.745 |
| dipyridamole | mebendazole | 0.790 | 0.726 |
| artemisinin | gliclazide | 0.790 | 0.746 |

| | | | |
|---|---|---|---|
| exemestane | sulpiride | 0.790 | 0.726 |
| bupivacaine | trazodone | 0.790 | 0.708 |
| bromperidol | propylthiouracil | 0.790 | 0.756 |
| clonidine | tyloxapol | 0.790 | 0.899 |
| ioxaglic_acid | penbutolol | 0.790 | 0.807 |
| metyrapone | pentoxyverine | 0.790 | 0.691 |
| clomifene | rescinnamine | 0.790 | 0.781 |
| dilazep | nalidixic_acid | 0.790 | 0.765 |
| sulfasalazine | thiocolchicoside | 0.790 | 0.696 |
| estriol | mesoridazine | 0.790 | 0.668 |
| cinoxacin | sulconazole | 0.790 | 0.770 |
| metyrapone | tobramycin | 0.790 | 0.791 |
| colecalciferol | estrone | 0.790 | 0.716 |
| hydrocortisone | papaverine | 0.790 | 0.754 |
| citiolone | dicoumarol | 0.790 | 0.746 |
| cinchocaine | thiocolchicoside | 0.790 | 0.733 |
| fluocinonide | gliclazide | 0.790 | 0.747 |
| bufexamac | prednisolone | 0.790 | 0.720 |
| lomefloxacin | propafenone | 0.790 | 0.737 |
| doxazosin | proxyphylline | 0.790 | 0.689 |
| (-)-atenolol | estriol | 0.790 | 0.709 |
| sulfaphenazole | ticarcillin | 0.790 | 0.724 |
| chlorzoxazone | norethisterone | 0.790 | 0.774 |
| apomorphine | sulconazole | 0.790 | 0.733 |
| glipizide | zimeldine | 0.790 | 0.783 |
| etofenamate | pyrvinium | 0.790 | 0.774 |
| ketanserin | tranylcypromine | 0.790 | 0.778 |
| ethotoin | triflusal | 0.790 | 0.702 |
| doxylamine | naphazoline | 0.790 | 0.672 |
| dacarbazine | estriol | 0.791 | 0.721 |
| buflomedil | ronidazole | 0.791 | 0.659 |
| carbamazepine | tribenoside | 0.791 | 0.745 |
| calcium_pantothenate | procaine | 0.791 | 0.807 |
| bisacodyl | clindamycin | 0.791 | 0.765 |
| piracetam | piribedil | 0.791 | 0.772 |
| etofylline | gliclazide | 0.791 | 0.735 |
| oxybuprocaine | phenazone | 0.791 | 0.725 |
| cefalexin | metacycline | 0.791 | 0.691 |
| torasemide | tyloxapol | 0.791 | 0.849 |
| colecalciferol | ursodeoxycholic_acid | 0.791 | 0.692 |
| cefsulodin | piperidolate | 0.791 | 0.771 |
| erastin | prenylamine | 0.791 | 0.764 |
| clindamycin | gefitinib | 0.791 | 0.763 |
| fluoxetine | perphenazine | 0.791 | 0.724 |
| cycloserine | hydrocortisone | 0.791 | 0.867 |
| oxprenolol | trazodone | 0.791 | 0.724 |
| sulfamethizole | trioxysalen | 0.791 | 0.734 |
| moroxydine | ofloxacin | 0.791 | 0.704 |
| clioquinol | perhexiline | 0.791 | 0.735 |
| bromopride | menadione | 0.791 | 0.697 |

| | | | |
|---|---|---|---|
| gliclazide | simvastatin | 0.791 | 0.765 |
| iopanoic_acid | risperidone | 0.791 | 0.764 |
| terbutaline | verteporfin | 0.791 | 0.840 |
| cyanocobalamin | scopolamine | 0.791 | 0.883 |
| oxamic_acid | xylometazoline | 0.791 | 0.875 |
| clioquinol | promethazine | 0.791 | 0.685 |
| dipyridamole | talampicillin | 0.791 | 0.755 |
| betaxolol | dopamine | 0.791 | 0.694 |
| albendazole | astemizole | 0.791 | 0.749 |
| azacitidine | erastin | 0.791 | 0.793 |
| estriol | tiabendazole | 0.791 | 0.732 |
| metergoline | propranolol | 0.791 | 0.664 |
| bacitracin | trapidil | 0.791 | 0.913 |
| benzylpenicillin | labetalol | 0.791 | 0.681 |
| cefalotin | etomidate | 0.791 | 0.741 |
| pentoxyverine | trioxysalen | 0.791 | 0.741 |
| fluspirilene | rimexolone | 0.791 | 0.701 |
| clonidine | meticrane | 0.791 | 0.720 |
| meclozine | protriptyline | 0.791 | 0.679 |
| butoconazole | dextromethorphan | 0.791 | 0.707 |
| pridinol | repaglinide | 0.791 | 0.744 |
| sulpiride | tetryzoline | 0.791 | 0.752 |
| acepromazine | dextromethorphan | 0.791 | 0.733 |
| cefalotin | gefitinib | 0.791 | 0.732 |
| letrozole | orciprenaline | 0.791 | 0.748 |
| meptazinol | spectinomycin | 0.791 | 0.762 |
| clobetasol | metamizole_sodium | 0.791 | 0.723 |
| calcium_pantothenate | pentoxyverine | 0.791 | 0.774 |
| ioxaglic_acid | ipratropium_bromide | 0.791 | 0.798 |
| menadione | scopolamine | 0.791 | 0.749 |
| pimethixene | propofol | 0.791 | 0.748 |
| gefitinib | prenylamine | 0.791 | 0.770 |
| cortisone | dacarbazine | 0.791 | 0.785 |
| aminocaproic_acid | nabumetone | 0.791 | 0.710 |
| ketanserin | probucol | 0.791 | 0.752 |
| cycloserine | piracetam | 0.791 | 0.685 |
| citiolone | fluocinonide | 0.791 | 0.824 |
| reserpine | terguride | 0.791 | 0.763 |
| fluorometholone | levamisole | 0.791 | 0.768 |
| albendazole | sulconazole | 0.791 | 0.745 |
| repaglinide | zimeldine | 0.791 | 0.763 |
| scopolamine | trimetazidine | 0.791 | 0.701 |
| etamsylate | nitrofurantoin | 0.791 | 0.711 |
| acepromazine | betaxolol | 0.791 | 0.748 |
| carbachol | salbutamol | 0.791 | 0.727 |
| gefitinib | oleandomycin | 0.791 | 0.803 |
| doxorubicin | sulfafurazole | 0.791 | 0.757 |
| cyproterone | dipyridamole | 0.791 | 0.742 |
| famotidine | menadione | 0.791 | 0.784 |
| norfloxacin | phenindione | 0.791 | 0.707 |

| | | | |
|---|---|---|---|
| desipramine | phenylpropanolamine | 0.791 | 0.736 |
| oxamniquine | sulconazole | 0.791 | 0.738 |
| hydralazine | repaglinide | 0.791 | 0.781 |
| amiloride | doxazosin | 0.791 | 0.683 |
| clofazimine | fluphenazine | 0.791 | 0.722 |
| betahistine | idoxuridine | 0.791 | 0.663 |
| etamivan | gliclazide | 0.791 | 0.760 |
| piracetam | xylometazoline | 0.791 | 0.727 |
| kanamycin | ketanserin | 0.791 | 0.736 |
| flurbiprofen | hydrocortisone | 0.791 | 0.717 |
| amiodarone | sulfamerazine | 0.791 | 0.756 |
| ethotoin | pargyline | 0.791 | 0.663 |
| aminocaproic_acid | sulfafurazole | 0.791 | 0.731 |
| cortisone | etomidate | 0.791 | 0.748 |
| dorzolamide | levonorgestrel | 0.791 | 0.766 |
| cycloserine | picotamide | 0.791 | 0.932 |
| amitriptyline | sulindac | 0.791 | 0.714 |
| chloropyramine | theophylline | 0.791 | 0.795 |
| dexibuprofen | procainamide | 0.791 | 0.720 |
| benperidol | rolitetracycline | 0.791 | 0.716 |
| carbachol | methazolamide | 0.791 | 0.685 |
| menadione | oxprenolol | 0.791 | 0.723 |
| acepromazine | sulfametoxydiazine | 0.791 | 0.723 |
| nabumetone | propafenone | 0.791 | 0.711 |
| daunorubicin | proscillaridin | 0.791 | 0.761 |
| clindamycin | sulfaphenazole | 0.791 | 0.728 |
| etidronic_acid | triflusal | 0.791 | 0.734 |
| methoxamine | tolazamide | 0.791 | 0.703 |
| dihydroergocristine | prenylamine | 0.791 | 0.753 |
| salbutamol | thiocolchicoside | 0.791 | 0.778 |
| meclozine | progesterone | 0.791 | 0.791 |
| deptropine | propafenone | 0.791 | 0.731 |
| molindone | piribedil | 0.791 | 0.686 |
| practolol | tolazoline | 0.791 | 0.742 |
| proscillaridin | tobramycin | 0.791 | 0.729 |
| oxprenolol | zimeldine | 0.791 | 0.699 |
| bromopride | progesterone | 0.791 | 0.741 |
| clobetasol | propafenone | 0.791 | 0.731 |
| natamycin | pargyline | 0.791 | 0.872 |
| deferoxamine | miconazole | 0.791 | 0.847 |
| proscillaridin | sulfaphenazole | 0.791 | 0.792 |
| perhexiline | thioproperazine | 0.791 | 0.750 |
| amoxicillin | betaxolol | 0.791 | 0.733 |
| estriol | ioversol | 0.791 | 0.785 |
| omeprazole | vidarabine | 0.791 | 0.656 |
| practolol | probucol | 0.791 | 0.789 |
| colecalciferol | menadione | 0.791 | 0.794 |
| dinoprost | hexetidine | 0.791 | 0.759 |
| amrinone | pridinol | 0.791 | 0.769 |
| bromopride | meticrane | 0.791 | 0.732 |

| | | | |
|---|---|---|---|
| bepridil | vorinostat | 0.791 | 0.771 |
| mephenesin | reserpine | 0.791 | 0.797 |
| cinoxacin | omeprazole | 0.791 | 0.662 |
| glibenclamide | promazine | 0.791 | 0.779 |
| alimemazine | sulfametoxydiazine | 0.791 | 0.731 |
| etodolac | norfloxacin | 0.791 | 0.659 |
| norethisterone | procaine | 0.791 | 0.742 |
| benzylpenicillin | levamisole | 0.791 | 0.734 |
| promazine | ronidazole | 0.791 | 0.704 |
| prednicarbate | risperidone | 0.791 | 0.708 |
| guanethidine | oxybutynin | 0.791 | 0.747 |
| mebendazole | roxithromycin | 0.791 | 0.837 |
| carbachol | pargyline | 0.791 | 0.734 |
| clonidine | vinpocetine | 0.791 | 0.718 |
| metoprolol | oxamniquine | 0.791 | 0.671 |
| bromopride | oxprenolol | 0.791 | 0.667 |
| rolitetracycline | sulfaphenazole | 0.791 | 0.735 |
| bromperidol | tolfenamic_acid | 0.791 | 0.733 |
| chloramphenicol | meticrane | 0.791 | 0.722 |
| isopropamide_iodide | zomepirac | 0.791 | 0.728 |
| dyclonine | topiramate | 0.791 | 0.780 |
| cefalotin | pyrvinium | 0.791 | 0.788 |
| etofenamate | ursodeoxycholic_acid | 0.791 | 0.739 |
| (-)-catechin | acepromazine | 0.791 | 0.687 |
| fludroxycortide | monobenzone | 0.791 | 0.767 |
| fluorometholone | trioxysalen | 0.791 | 0.761 |
| fenoprofen | flupentixol | 0.791 | 0.736 |
| ifenprodil | tyloxapol | 0.791 | 0.833 |
| (-)-catechin | ipratropium_bromide | 0.791 | 0.703 |
| estriol | terbutaline | 0.791 | 0.692 |
| cetirizine | fluorometholone | 0.791 | 0.744 |
| dipyridamole | levamisole | 0.791 | 0.809 |
| cefalexin | oxybuprocaine | 0.791 | 0.711 |
| colchicine | tetryzoline | 0.791 | 0.727 |
| dirithromycin | hyoscyamine | 0.791 | 0.826 |
| natamycin | pentoxyverine | 0.791 | 0.810 |
| (-)-atenolol | ronidazole | 0.791 | 0.684 |
| doxylamine | rifabutin | 0.791 | 0.828 |
| hydrocortisone | remoxipride | 0.791 | 0.742 |
| pentamidine | tobramycin | 0.791 | 0.793 |
| loperamide | terconazole | 0.791 | 0.740 |
| diltiazem | fluocinonide | 0.791 | 0.740 |
| dipivefrine | fenoprofen | 0.791 | 0.730 |
| liothyronine | methazolamide | 0.791 | 0.749 |
| amoxapine | etofylline | 0.791 | 0.686 |
| azacitidine | piromidic_acid | 0.791 | 0.660 |
| fendiline | haloperidol | 0.791 | 0.724 |
| cinchocaine | lomefloxacin | 0.791 | 0.660 |
| amoxicillin | tyloxapol | 0.791 | 0.860 |
| aceclofenac | levocabastine | 0.791 | 0.753 |

| | | | |
|---|---|---|---|
| pizotifen | prenylamine | 0.791 | 0.709 |
| dexibuprofen | meptazinol | 0.791 | 0.711 |
| digoxin | progesterone | 0.791 | 0.811 |
| bupropion | fluorometholone | 0.791 | 0.733 |
| gliclazide | ketoprofen | 0.791 | 0.704 |
| clonidine | meptazinol | 0.791 | 0.719 |
| cefalexin | procaine | 0.791 | 0.679 |
| diltiazem | oxprenolol | 0.791 | 0.721 |
| amoxapine | miconazole | 0.791 | 0.688 |
| fenbendazole | mometasone | 0.791 | 0.738 |
| natamycin | rolitetracycline | 0.791 | 0.773 |
| metoclopramide | prilocaine | 0.791 | 0.653 |
| bromperidol | dipyridamole | 0.791 | 0.772 |
| metacycline | pentoxyverine | 0.791 | 0.743 |
| clindamycin | ipratropium_bromide | 0.791 | 0.711 |
| bromperidol | norethisterone | 0.791 | 0.742 |
| demecolcine | meticrane | 0.791 | 0.736 |
| imipramine | nifedipine | 0.791 | 0.752 |
| etofenamate | hydralazine | 0.791 | 0.684 |
| cyproterone | sulfamethoxypyridazir | 0.791 | 0.744 |
| aminohippuric_acid | imipenem | 0.791 | 0.725 |
| cycloserine | piromidic_acid | 0.791 | 0.836 |
| demecolcine | tamoxifen | 0.791 | 0.731 |
| etomidate | sulfamethoxypyridazir | 0.791 | 0.669 |
| estriol | imipenem | 0.791 | 0.714 |
| fenoterol | perhexiline | 0.791 | 0.767 |
| demecolcine | raloxifene | 0.791 | 0.751 |
| acepromazine | roxithromycin | 0.791 | 0.837 |
| dipyridamole | norethisterone | 0.791 | 0.750 |
| chlorphenamine | tamoxifen | 0.791 | 0.688 |
| disulfiram | vigabatrin | 0.791 | 0.739 |
| labetalol | protriptyline | 0.791 | 0.765 |
| dexpanthenol | noretynodrel | 0.791 | 0.728 |
| cefamandole | levomepromazine | 0.791 | 0.776 |
| fluocinonide | repaglinide | 0.791 | 0.719 |
| meclozine | rimexolone | 0.791 | 0.760 |
| daunorubicin | piromidic_acid | 0.791 | 0.695 |
| ajmaline | ambroxol | 0.791 | 0.698 |
| dipyridamole | pyrvinium | 0.791 | 0.781 |
| chloramphenicol | isopropamide_iodide | 0.791 | 0.733 |
| sertaconazole | trifluoperazine | 0.791 | 0.706 |
| diclofenac | talampicillin | 0.791 | 0.775 |
| desoxycortone | ticarcillin | 0.791 | 0.723 |
| liothyronine | methylergometrine | 0.791 | 0.688 |
| azacitidine | tropicamide | 0.791 | 0.710 |
| clindamycin | oxolinic_acid | 0.791 | 0.741 |
| fenbendazole | fluspirilene | 0.792 | 0.684 |
| desoxycortone | ketanserin | 0.792 | 0.742 |
| sulconazole | syrosingopine | 0.792 | 0.803 |
| amiodarone | cyproterone | 0.792 | 0.744 |

| | | | |
|---|---|---|---|
| bromocriptine | ivermectin | 0.792 | 0.794 |
| flunisolide | talampicillin | 0.792 | 0.717 |
| meptazinol | meticrane | 0.792 | 0.742 |
| fipexide | tranylcypromine | 0.792 | 0.771 |
| chloramphenicol | promazine | 0.792 | 0.733 |
| etodolac | tiratricol | 0.792 | 0.702 |
| natamycin | vidarabine | 0.792 | 0.795 |
| phenoxybenzamine | tobramycin | 0.792 | 0.807 |
| astemizole | latamoxef | 0.792 | 0.748 |
| bupropion | irinotecan | 0.792 | 0.796 |
| chlorzoxazone | pentoxyverine | 0.792 | 0.750 |
| betaxolol | methazolamide | 0.792 | 0.746 |
| iloprost | isoniazid | 0.792 | 0.823 |
| aminohippuric_acid | omeprazole | 0.792 | 0.680 |
| isotretinoin | niclosamide | 0.792 | 0.657 |
| bepridil | liothyronine | 0.792 | 0.755 |
| practolol | tribenoside | 0.792 | 0.753 |
| labetalol | verteporfin | 0.792 | 0.789 |
| tioguanine | trifluoperazine | 0.792 | 0.744 |
| (-)-catechin | tiratricol | 0.792 | 0.706 |
| ioxaglic_acid | lymecycline | 0.792 | 0.756 |
| clomifene | dextromethorphan | 0.792 | 0.768 |
| buspirone | procainamide | 0.792 | 0.715 |
| rescinnamine | suloctidil | 0.792 | 0.754 |
| meclozine | sulfaphenazole | 0.792 | 0.724 |
| alimemazine | rimexolone | 0.792 | 0.773 |
| meticrane | oxybuprocaine | 0.792 | 0.706 |
| famotidine | propofol | 0.792 | 0.787 |
| desoxycortone | etamsylate | 0.792 | 0.807 |
| etodolac | ranitidine | 0.792 | 0.752 |
| fludroxycortide | sulfamethoxazole | 0.792 | 0.741 |
| propylthiouracil | salbutamol | 0.792 | 0.662 |
| metrifonate | naproxen | 0.792 | 0.751 |
| cefixime | naloxone | 0.792 | 0.770 |
| fenoprofen | ioversol | 0.792 | 0.792 |
| etofenamate | phenoxybenzamine | 0.792 | 0.761 |
| benfotiamine | sulfametoxydiazine | 0.792 | 0.735 |
| latamoxef | roxithromycin | 0.792 | 0.791 |
| cefalexin | propantheline_bromic | 0.792 | 0.703 |
| carbachol | moxonidine | 0.792 | 0.725 |
| naftidrofuryl | nicotinic_acid | 0.792 | 0.778 |
| felodipine | propylthiouracil | 0.792 | 0.730 |
| chlorambucil | tyloxapol | 0.792 | 0.854 |
| meclozine | triflusal | 0.792 | 0.809 |
| glibenclamide | pargyline | 0.792 | 0.795 |
| cefapirin | ribostamycin | 0.792 | 0.756 |
| scopolamine | tolmetin | 0.792 | 0.686 |
| trimetazidine | verteporfin | 0.792 | 0.823 |
| mebendazole | nortriptyline | 0.792 | 0.706 |
| betaxolol | liothyronine | 0.792 | 0.719 |

| | | | |
|---|---|---|---|
| propafenone | roxithromycin | 0.792 | 0.816 |
| bromopride | piperidolate | 0.792 | 0.669 |
| chlorphenesin | sulfamethoxypyridazir | 0.792 | 0.658 |
| estriol | ketorolac | 0.792 | 0.666 |
| flunarizine | terguride | 0.792 | 0.730 |
| dipyridamole | trimetazidine | 0.792 | 0.742 |
| biotin | bisacodyl | 0.792 | 0.721 |
| rimexolone | tribenoside | 0.792 | 0.752 |
| guanethidine | pentoxyverine | 0.792 | 0.746 |
| dilazep | tiapride | 0.792 | 0.767 |
| budesonide | thiocolchicoside | 0.792 | 0.732 |
| meclozine | piromidic_acid | 0.792 | 0.773 |
| gliclazide | lymecycline | 0.792 | 0.754 |
| liothyronine | pentoxyverine | 0.792 | 0.747 |
| bisacodyl | ketoprofen | 0.792 | 0.709 |
| cycloserine | talampicillin | 0.792 | 0.852 |
| remoxipride | sulfamethoxypyridazir | 0.792 | 0.679 |
| cyproterone | phenindione | 0.792 | 0.778 |
| acepromazine | etamivan | 0.792 | 0.689 |
| clioquinol | estrone | 0.792 | 0.721 |
| daunorubicin | ioversol | 0.792 | 0.720 |
| oxprenolol | proxymetacaine | 0.792 | 0.661 |
| benzathine_benzylpenicillin | topiramate | 0.792 | 0.728 |
| natamycin | piribedil | 0.792 | 0.822 |
| fluvastatin | propylthiouracil | 0.792 | 0.759 |
| estriol | metyrapone | 0.792 | 0.681 |
| lymecycline | sulfamethoxypyridazir | 0.792 | 0.776 |
| etomidate | flucytosine | 0.792 | 0.750 |
| flufenamic_acid | procaine | 0.792 | 0.666 |
| cetirizine | oxprenolol | 0.792 | 0.730 |
| clomipramine | fenoprofen | 0.792 | 0.727 |
| fluspirilene | nitrofurantoin | 0.792 | 0.735 |
| flunarizine | imipenem | 0.792 | 0.764 |
| fipexide | hydrocortisone | 0.792 | 0.725 |
| nafcillin | phenacetin | 0.792 | 0.750 |
| flupentixol | haloperidol | 0.792 | 0.719 |
| cefalexin | tyloxapol | 0.792 | 0.846 |
| fipexide | imipenem | 0.792 | 0.745 |
| chlorzoxazone | fluspirilene | 0.792 | 0.788 |
| ioversol | ipratropium_bromide | 0.792 | 0.780 |
| levomepromazine | sulfadimethoxine | 0.792 | 0.696 |
| griseofulvin | promazine | 0.792 | 0.711 |
| etidronic_acid | molindone | 0.792 | 0.805 |
| clomipramine | talampicillin | 0.792 | 0.795 |
| dilazep | etacrynic_acid | 0.792 | 0.801 |
| bepridil | desoxycortone | 0.792 | 0.777 |
| piromidic_acid | sulfafurazole | 0.792 | 0.707 |
| lidoflazine | tropine | 0.792 | 0.879 |
| altretamine | prasterone | 0.792 | 0.807 |
| nizatidine | vigabatrin | 0.792 | 0.771 |

| | | | |
|---|---|---|---|
| bepridil | sulfaphenazole | 0.792 | 0.732 |
| astemizole | norfloxacin | 0.792 | 0.761 |
| diclofenamide | meglumine | 0.792 | 0.708 |
| aminohippuric_acid | hyoscyamine | 0.792 | 0.687 |
| metyrapone | proxyphylline | 0.792 | 0.673 |
| alprostadil | pergolide | 0.792 | 0.803 |
| apomorphine | famotidine | 0.792 | 0.762 |
| amiodarone | ethotoin | 0.792 | 0.758 |
| cisapride | menadione | 0.792 | 0.778 |
| gefitinib | naloxone | 0.792 | 0.780 |
| desoxycortone | mesalazine | 0.792 | 0.788 |
| aminohippuric_acid | methapyrilene | 0.792 | 0.705 |
| etofenamate | sulfamerazine | 0.792 | 0.718 |
| bufexamac | propafenone | 0.792 | 0.715 |
| bezafibrate | theobromine | 0.792 | 0.771 |
| mercaptopurine | trifluoperazine | 0.792 | 0.739 |
| dihydroergotamine | etoposide | 0.792 | 0.733 |
| sulconazole | testosterone | 0.792 | 0.733 |
| amrinone | benzylpenicillin | 0.792 | 0.778 |
| dipyridamole | tranylcypromine | 0.792 | 0.803 |
| bromocriptine | tolazoline | 0.792 | 0.822 |
| ambroxol | iloprost | 0.792 | 0.755 |
| etofenamate | natamycin | 0.792 | 0.773 |
| acetylsalicylic_acid | etofenamate | 0.792 | 0.714 |
| fluoxetine | fluphenazine | 0.792 | 0.706 |
| ganciclovir | pralidoxime | 0.792 | 0.699 |
| felodipine | zuclopenthixol | 0.792 | 0.750 |
| fipexide | trimetazidine | 0.792 | 0.732 |
| bezafibrate | clindamycin | 0.792 | 0.737 |
| desoxycortone | trazodone | 0.792 | 0.733 |
| capsaicin | nalidixic_acid | 0.792 | 0.722 |
| methotrexate | monobenzone | 0.792 | 0.756 |
| acepromazine | ethotoin | 0.792 | 0.731 |
| estriol | fipexide | 0.792 | 0.735 |
| naproxen | sulfaphenazole | 0.792 | 0.677 |
| cyclobenzaprine | niclosamide | 0.792 | 0.683 |
| azacitidine | desoxycortone | 0.792 | 0.771 |
| halcinonide | tyloxapol | 0.792 | 0.846 |
| salbutamol | tolfenamic_acid | 0.792 | 0.667 |
| etomidate | sulfametoxydiazine | 0.792 | 0.669 |
| torasemide | tridihexethyl | 0.792 | 0.730 |
| estriol | ticlopidine | 0.792 | 0.734 |
| hydralazine | metoclopramide | 0.792 | 0.675 |
| pilocarpine | profenamine | 0.792 | 0.713 |
| letrozole | terconazole | 0.792 | 0.735 |
| bepridil | flunisolide | 0.792 | 0.779 |
| doxorubicin | liothyronine | 0.792 | 0.725 |
| calcium_pantothenate | piribedil | 0.792 | 0.788 |
| (-)-atenolol | oxprenolol | 0.792 | 0.676 |
| butoconazole | cefalotin | 0.792 | 0.762 |

| | | | |
|---|---|---|---|
| benzethonium_chloride | metixene | 0.792 | 0.766 |
| oxybuprocaine | practolol | 0.792 | 0.667 |
| dihydroergotamine | etacrynic_acid | 0.792 | 0.773 |
| cyproterone | meptazinol | 0.792 | 0.759 |
| clindamycin | promazine | 0.792 | 0.775 |
| estrone | trimipramine | 0.792 | 0.696 |
| aminohippuric_acid | rolitetracycline | 0.792 | 0.750 |
| flupentixol | ganciclovir | 0.792 | 0.694 |
| acebutolol | oxamic_acid | 0.792 | 0.829 |
| cetirizine | procainamide | 0.792 | 0.753 |
| bromperidol | dosulepin | 0.792 | 0.784 |
| amiodarone | ipratropium_bromide | 0.792 | 0.721 |
| dantrolene | iohexol | 0.792 | 0.771 |
| acepromazine | tropicamide | 0.792 | 0.719 |
| meropenem | simvastatin | 0.792 | 0.730 |
| exemestane | irinotecan | 0.792 | 0.769 |
| promethazine | tranylcypromine | 0.792 | 0.692 |
| beclometasone | liothyronine | 0.792 | 0.729 |
| dicycloverine | levodopa | 0.792 | 0.774 |
| clemastine | dihydroergocristine | 0.792 | 0.783 |
| famotidine | levamisole | 0.792 | 0.767 |
| chlorzoxazone | oxytetracycline | 0.792 | 0.778 |
| ioxaglic_acid | scopolamine | 0.792 | 0.818 |
| norethisterone | remoxipride | 0.792 | 0.757 |
| cefalotin | phenoxybenzamine | 0.792 | 0.752 |
| azacitidine | sulfamerazine | 0.792 | 0.653 |
| terbutaline | trioxysalen | 0.792 | 0.653 |
| menadione | promethazine | 0.792 | 0.688 |
| fenbendazole | suloctidil | 0.792 | 0.730 |
| mafenide | naltrexone | 0.792 | 0.749 |
| atovaquone | milrinone | 0.792 | 0.728 |
| hydralazine | remoxipride | 0.792 | 0.699 |
| metacycline | trimipramine | 0.792 | 0.763 |
| dopamine | oxamic_acid | 0.792 | 0.736 |
| metoprolol | zalcitabine | 0.792 | 0.693 |
| amoxapine | repaglinide | 0.792 | 0.766 |
| meticrane | sparteine | 0.792 | 0.751 |
| cefalotin | desoxycortone | 0.792 | 0.745 |
| ketoconazole | tyloxapol | 0.792 | 0.831 |
| flunisolide | trioxysalen | 0.792 | 0.792 |
| atovaquone | etidronic_acid | 0.792 | 0.797 |
| lymecycline | procaine | 0.792 | 0.776 |
| levonorgestrel | nitrendipine | 0.792 | 0.764 |
| etamsylate | scopolamine | 0.792 | 0.717 |
| cycloserine | glibenclamide | 0.792 | 0.886 |
| propafenone | urapidil | 0.792 | 0.734 |
| propylthiouracil | trichlormethiazide | 0.792 | 0.724 |
| demecolcine | fenbendazole | 0.792 | 0.714 |
| levonorgestrel | sulfamethoxypyridazir | 0.792 | 0.745 |
| bepridil | carbachol | 0.792 | 0.810 |

| | | | |
|---|---|---|---|
| imipramine | procaine | 0.792 | 0.719 |
| enoxacin | isoniazid | 0.792 | 0.676 |
| paroxetine | perphenazine | 0.792 | 0.734 |
| medrysone | promazine | 0.792 | 0.748 |
| cefalexin | meptazinol | 0.792 | 0.764 |
| alfaxalone | deptropine | 0.792 | 0.764 |
| cycloserine | tyloxapol | 0.792 | 0.925 |
| liothyronine | nafcillin | 0.792 | 0.696 |
| astemizole | fulvestrant | 0.792 | 0.763 |
| ethambutol | valproic_acid | 0.792 | 0.727 |
| bepridil | cefalexin | 0.792 | 0.761 |
| ketoprofen | meticrane | 0.792 | 0.650 |
| bepridil | mesalazine | 0.792 | 0.812 |
| colecalciferol | fluspirilene | 0.792 | 0.701 |
| benzonatate | triflupromazine | 0.792 | 0.781 |
| fluocinonide | metyrapone | 0.792 | 0.758 |
| ethotoin | ketanserin | 0.793 | 0.709 |
| chlorcyclizine | ciclopirox | 0.793 | 0.737 |
| nitrendipine | pralidoxime | 0.793 | 0.768 |
| deptropine | prenylamine | 0.793 | 0.727 |
| reserpine | trifluoperazine | 0.793 | 0.731 |
| dipivefrine | metamizole_sodium | 0.793 | 0.711 |
| doxazosin | pentamidine | 0.793 | 0.712 |
| methoxamine | nifurtimox | 0.793 | 0.730 |
| flunisolide | levamisole | 0.793 | 0.790 |
| chlorambucil | natamycin | 0.793 | 0.817 |
| felodipine | tolfenamic_acid | 0.793 | 0.727 |
| betahistine | nitrofurantoin | 0.793 | 0.664 |
| cyclobenzaprine | econazole | 0.793 | 0.664 |
| betahistine | doxorubicin | 0.793 | 0.753 |
| acetazolamide | mefenamic_acid | 0.793 | 0.701 |
| bezafibrate | chloramphenicol | 0.793 | 0.732 |
| doxazosin | flavoxate | 0.793 | 0.687 |
| etidronic_acid | simvastatin | 0.793 | 0.811 |
| sulpiride | troleandomycin | 0.793 | 0.806 |
| benzethonium_chloride | triflusal | 0.793 | 0.829 |
| butoconazole | estrone | 0.793 | 0.742 |
| omeprazole | oxybuprocaine | 0.793 | 0.692 |
| betahistine | oxybuprocaine | 0.793 | 0.699 |
| milrinone | pyrantel | 0.793 | 0.690 |
| debrisoquine | desipramine | 0.793 | 0.684 |
| tiaprofenic_acid | tobramycin | 0.793 | 0.778 |
| nitrofurantoin | rolitetracycline | 0.793 | 0.772 |
| etofenamate | levodopa | 0.793 | 0.738 |
| aminophylline | rolitetracycline | 0.793 | 0.810 |
| flupentixol | gliclazide | 0.793 | 0.706 |
| amoxicillin | meclofenoxate | 0.793 | 0.707 |
| ronidazole | tobramycin | 0.793 | 0.811 |
| iloprost | nadolol | 0.793 | 0.740 |
| clebopride | guanfacine | 0.793 | 0.714 |

| | | | |
|---|---|---|---|
| idoxuridine | quinisocaine | 0.793 | 0.704 |
| triamterene | trifluoperazine | 0.793 | 0.681 |
| flupentixol | pipemidic_acid | 0.793 | 0.660 |
| etacrynic_acid | ifenprodil | 0.793 | 0.735 |
| menadione | mesoridazine | 0.793 | 0.739 |
| syrosingopine | tioguanine | 0.793 | 0.834 |
| buspirone | ioversol | 0.793 | 0.775 |
| etofenamate | tiapride | 0.793 | 0.704 |
| adipiodone | rimexolone | 0.793 | 0.777 |
| etodolac | naltrexone | 0.793 | 0.708 |
| ethambutol | troleandomycin | 0.793 | 0.846 |
| piromidic_acid | propafenone | 0.793 | 0.710 |
| talampicillin | terguride | 0.793 | 0.738 |
| liothyronine | phenindione | 0.793 | 0.747 |
| chlorcyclizine | pyrvinium | 0.793 | 0.799 |
| proxymetacaine | trifluoperazine | 0.793 | 0.724 |
| flucytosine | metyrapone | 0.793 | 0.721 |
| ioversol | natamycin | 0.793 | 0.732 |
| dilazep | metaraminol | 0.793 | 0.825 |
| metyrapone | proscillaridin | 0.793 | 0.782 |
| dantrolene | phentolamine | 0.793 | 0.744 |
| menadione | nortriptyline | 0.793 | 0.728 |
| etoposide | mercaptopurine | 0.793 | 0.804 |
| amodiaquine | sulfinpyrazone | 0.793 | 0.730 |
| dextromethorphan | scopolamine | 0.793 | 0.740 |
| meptazinol | oxamniquine | 0.793 | 0.751 |
| bezafibrate | pentoxyverine | 0.793 | 0.734 |
| hydrocortisone | propofol | 0.793 | 0.794 |
| fluorometholone | methazolamide | 0.793 | 0.756 |
| ketanserin | propafenone | 0.793 | 0.716 |
| budesonide | dipyridamole | 0.793 | 0.754 |
| doxazosin | ethambutol | 0.793 | 0.770 |
| haloperidol | prochlorperazine | 0.793 | 0.745 |
| etomidate | ioversol | 0.793 | 0.805 |
| fenspiride | rimexolone | 0.793 | 0.699 |
| dipyridamole | levonorgestrel | 0.793 | 0.756 |
| isradipine | procaine | 0.793 | 0.751 |
| propantheline_bromide | sulpiride | 0.793 | 0.671 |
| betahistine | pentoxyverine | 0.793 | 0.716 |
| clemastine | fluspirilene | 0.793 | 0.712 |
| rolitetracycline | tranylcypromine | 0.793 | 0.810 |
| cycloserine | liothyronine | 0.793 | 0.800 |
| bromocriptine | nabumetone | 0.793 | 0.762 |
| ethambutol | tyloxapol | 0.793 | 0.886 |
| cyproterone | etidronic_acid | 0.793 | 0.792 |
| acetylsalicylic_acid | pyrvinium | 0.793 | 0.852 |
| acepromazine | buflomedil | 0.793 | 0.685 |
| mesalazine | molindone | 0.793 | 0.668 |
| pivmecillinam | risperidone | 0.793 | 0.725 |
| cinoxacin | sulfametoxydiazine | 0.793 | 0.664 |

| | | | |
|---|---|---|---|
| amiodarone | trioxysalen | 0.793 | 0.760 |
| estriol | trifluridine | 0.793 | 0.697 |
| cyproterone | scopolamine | 0.793 | 0.696 |
| labetalol | pentetrazol | 0.793 | 0.804 |
| doxazosin | estrone | 0.793 | 0.730 |
| acetylsalicylic_acid | meclofenoxate | 0.793 | 0.710 |
| suloctidil | vorinostat | 0.793 | 0.731 |
| fenoterol | zimeldine | 0.793 | 0.736 |
| amiodarone | cinchocaine | 0.793 | 0.682 |
| iopanoic_acid | molindone | 0.793 | 0.681 |
| dextromethorphan | lymecycline | 0.793 | 0.812 |
| acetohexamide | naloxone | 0.793 | 0.745 |
| lymecycline | propylthiouracil | 0.793 | 0.822 |
| labetalol | levamisole | 0.793 | 0.724 |
| aminocaproic_acid | talampicillin | 0.793 | 0.822 |
| ketanserin | milrinone | 0.793 | 0.736 |
| betahistine | trichlormethiazide | 0.793 | 0.744 |
| cyanocobalamin | sulconazole | 0.793 | 0.885 |
| spiramycin | sulfadimidine | 0.793 | 0.841 |
| benzethonium_chloride | ticlopidine | 0.793 | 0.761 |
| etidronic_acid | tiabendazole | 0.793 | 0.780 |
| doxepin | valproic_acid | 0.793 | 0.749 |
| latamoxef | terfenadine | 0.793 | 0.740 |
| ethosuximide | pentoxyverine | 0.793 | 0.771 |
| betamethasone | rolitetracycline | 0.793 | 0.704 |
| quinisocaine | sulconazole | 0.793 | 0.700 |
| hyoscyamine | latamoxef | 0.793 | 0.750 |
| cinchocaine | troleandomycin | 0.793 | 0.825 |
| ciclosporin | mefloquine | 0.793 | 0.864 |
| bromperidol | tobramycin | 0.793 | 0.758 |
| doxazosin | oxaprozin | 0.793 | 0.716 |
| diltiazem | propafenone | 0.793 | 0.698 |
| aminophenazone | benzonatate | 0.793 | 0.837 |
| pheniramine | tolnaftate | 0.793 | 0.695 |
| proscillaridin | talampicillin | 0.793 | 0.713 |
| fluspirilene | verteporfin | 0.793 | 0.788 |
| glibenclamide | labetalol | 0.793 | 0.721 |
| cetirizine | dextromethorphan | 0.793 | 0.766 |
| amoxicillin | cycloserine | 0.793 | 0.818 |
| flumequine | rimexolone | 0.793 | 0.766 |
| fenoterol | sulconazole | 0.793 | 0.758 |
| etofylline | rimexolone | 0.793 | 0.760 |
| hydroquinine | perphenazine | 0.793 | 0.703 |
| betahistine | dipyridamole | 0.793 | 0.800 |
| deferoxamine | repaglinide | 0.793 | 0.822 |
| amoxicillin | bezafibrate | 0.793 | 0.716 |
| bisoprolol | methoxsalen | 0.793 | 0.727 |
| cefalexin | dorzolamide | 0.793 | 0.747 |
| pentetrazol | trazodone | 0.793 | 0.764 |
| glibenclamide | ursodeoxycholic_acid | 0.793 | 0.745 |

| | | | |
|---|---|---|---|
| niridazole | remoxipride | 0.793 | 0.714 |
| alfaxalone | roxithromycin | 0.793 | 0.812 |
| glibenclamide | hyoscyamine | 0.793 | 0.741 |
| cycloserine | proxyphylline | 0.793 | 0.783 |
| citiolone | cyproterone | 0.793 | 0.806 |
| estriol | levamisole | 0.793 | 0.680 |
| cyproterone | ramipril | 0.793 | 0.721 |
| guanethidine | oxamic_acid | 0.793 | 0.814 |
| metamizole_sodium | trazodone | 0.793 | 0.719 |
| etidronic_acid | halcinonide | 0.793 | 0.811 |
| nitrofurantoin | norethisterone | 0.793 | 0.699 |
| bufexamac | etamsylate | 0.793 | 0.680 |
| irinotecan | trimetazidine | 0.793 | 0.785 |
| bromperidol | metergoline | 0.793 | 0.661 |
| dextromethorphan | repaglinide | 0.793 | 0.783 |
| buspirone | droperidol | 0.793 | 0.661 |
| doxylamine | isoniazid | 0.793 | 0.749 |
| decitabine | irinotecan | 0.793 | 0.772 |
| oxprenolol | triflusal | 0.793 | 0.722 |
| acetohexamide | trioxysalen | 0.793 | 0.750 |
| ranitidine | tyloxapol | 0.793 | 0.870 |
| mitoxantrone | sulfapyridine | 0.793 | 0.759 |
| benzethonium_chloride | lanatoside_C | 0.793 | 0.832 |
| chlorhexidine | dobutamine | 0.793 | 0.767 |
| isoniazid | mefloquine | 0.793 | 0.719 |
| carbachol | prilocaine | 0.793 | 0.697 |
| etamsylate | irinotecan | 0.793 | 0.790 |
| benzydamine | mepyramine | 0.793 | 0.688 |
| fenoterol | piromidic_acid | 0.793 | 0.704 |
| dextromethorphan | pargyline | 0.793 | 0.694 |
| bisacodyl | etofenamate | 0.793 | 0.691 |
| oxamniquine | zuclopenthixol | 0.793 | 0.734 |
| chlorzoxazone | zomepirac | 0.793 | 0.674 |
| lymecycline | tranylcypromine | 0.793 | 0.794 |
| fluphenazine | fluvoxamine | 0.793 | 0.711 |
| acepromazine | levamisole | 0.793 | 0.713 |
| etomidate | fipexide | 0.793 | 0.741 |
| clindamycin | isoetarine | 0.793 | 0.731 |
| azacitidine | physostigmine | 0.793 | 0.743 |
| fenoprofen | trioxysalen | 0.793 | 0.681 |
| medrysone | tobramycin | 0.793 | 0.752 |
| lidoflazine | quinidine | 0.793 | 0.772 |
| cefalotin | salbutamol | 0.793 | 0.723 |
| hyoscyamine | ifenprodil | 0.793 | 0.727 |
| glipizide | moroxydine | 0.793 | 0.779 |
| metyrapone | pralidoxime | 0.793 | 0.687 |
| fipexide | oxybutynin | 0.793 | 0.728 |
| sisomicin | trimethadione | 0.793 | 0.853 |
| amoxapine | cinchocaine | 0.793 | 0.704 |
| carbachol | felodipine | 0.793 | 0.780 |

| | | | |
|---|---|---|---|
| acepromazine | nilutamide | 0.793 | 0.687 |
| proguanil | rilmenidine | 0.793 | 0.682 |
| ioversol | verteporfin | 0.793 | 0.753 |
| benzylpenicillin | chloramphenicol | 0.793 | 0.715 |
| butoconazole | sulfaphenazole | 0.793 | 0.724 |
| ethotoin | latamoxef | 0.793 | 0.775 |
| doxepin | metergoline | 0.793 | 0.727 |
| metergoline | propafenone | 0.793 | 0.686 |
| fluphenazine | terconazole | 0.793 | 0.737 |
| meticrane | phenformin | 0.793 | 0.722 |
| nitrofurantoin | torasemide | 0.793 | 0.712 |
| carbachol | natamycin | 0.793 | 0.882 |
| phenazopyridine | phenoxybenzamine | 0.793 | 0.725 |
| bepridil | terguride | 0.793 | 0.695 |
| prasterone | sulconazole | 0.793 | 0.752 |
| liothyronine | tranylcypromine | 0.793 | 0.760 |
| astemizole | prasterone | 0.793 | 0.769 |
| ethosuximide | gliclazide | 0.793 | 0.804 |
| chloramphenicol | terconazole | 0.793 | 0.768 |
| alimemazine | clonidine | 0.793 | 0.698 |
| aminohippuric_acid | fluvastatin | 0.793 | 0.720 |
| gliclazide | papaverine | 0.793 | 0.752 |
| adipiodone | octopamine | 0.793 | 0.827 |
| amoxicillin | daunorubicin | 0.793 | 0.729 |
| metyrapone | spectinomycin | 0.793 | 0.712 |
| estriol | fluorometholone | 0.793 | 0.695 |
| albendazole | alfuzosin | 0.793 | 0.675 |
| liothyronine | sulfadiazine | 0.793 | 0.741 |
| acepromazine | cetirizine | 0.793 | 0.749 |
| amiloride | etofenamate | 0.793 | 0.679 |
| quinidine | tropine | 0.793 | 0.804 |
| astemizole | troglitazone | 0.793 | 0.714 |
| atovaquone | ronidazole | 0.793 | 0.736 |
| ambroxol | naltrexone | 0.793 | 0.705 |
| acetylsalicylic_acid | nalbuphine | 0.793 | 0.733 |
| latamoxef | pimozide | 0.793 | 0.735 |
| clonidine | mebendazole | 0.793 | 0.698 |
| isotretinoin | oxyphenbutazone | 0.793 | 0.763 |
| hyoscyamine | ivermectin | 0.793 | 0.840 |
| cefalexin | mitoxantrone | 0.793 | 0.753 |
| bezafibrate | cefalexin | 0.793 | 0.693 |
| betahistine | cefalexin | 0.793 | 0.718 |
| isoetarine | meropenem | 0.793 | 0.756 |
| letrozole | ticarcillin | 0.793 | 0.755 |
| cyproterone | piribedil | 0.793 | 0.723 |
| azacitidine | gliclazide | 0.793 | 0.704 |
| proscillaridin | repaglinide | 0.793 | 0.747 |
| ipratropium_bromide | piromidic_acid | 0.793 | 0.718 |
| flavoxate | verteporfin | 0.793 | 0.726 |
| dipyridamole | metyrapone | 0.793 | 0.784 |

| | | | |
|---|---|---|---|
| nicergoline | terfenadine | 0.793 | 0.691 |
| clonidine | pimozide | 0.793 | 0.787 |
| albendazole | liothyronine | 0.793 | 0.687 |
| nalbuphine | sulfathiazole | 0.793 | 0.720 |
| methoxsalen | metixene | 0.793 | 0.717 |
| colchicine | fendiline | 0.793 | 0.767 |
| ethosuximide | talampicillin | 0.793 | 0.824 |
| ciclosporin | raloxifene | 0.793 | 0.841 |
| bendroflumethiazide | piperacillin | 0.793 | 0.709 |
| dydrogesterone | selegiline | 0.793 | 0.758 |
| galantamine | pridinol | 0.793 | 0.734 |
| niclosamide | pimethixene | 0.793 | 0.700 |
| metyrapone | propylthiouracil | 0.793 | 0.679 |
| fipexide | propafenone | 0.793 | 0.721 |
| doxylamine | oxybuprocaine | 0.793 | 0.743 |
| tobramycin | verteporfin | 0.793 | 0.769 |
| propylthiouracil | sulfafurazole | 0.793 | 0.692 |
| gliclazide | proglumide | 0.793 | 0.693 |
| fluvastatin | meglumine | 0.793 | 0.754 |
| latamoxef | zalcitabine | 0.794 | 0.770 |
| pyrazinamide | spectinomycin | 0.794 | 0.783 |
| ethambutol | oxetacaine | 0.794 | 0.781 |
| cefalotin | ursodeoxycholic_acid | 0.794 | 0.711 |
| mitoxantrone | nitrofurantoin | 0.794 | 0.701 |
| erastin | orlistat | 0.794 | 0.755 |
| lanatoside_C | loxapine | 0.794 | 0.881 |
| propylthiouracil | remoxipride | 0.794 | 0.748 |
| clozapine | niclosamide | 0.794 | 0.678 |
| aminohippuric_acid | mesoridazine | 0.794 | 0.694 |
| desoxycortone | dipyridamole | 0.794 | 0.747 |
| estriol | tranylcypromine | 0.794 | 0.761 |
| paromomycin | tolmetin | 0.794 | 0.820 |
| ciclopirox | mefloquine | 0.794 | 0.710 |
| minoxidil | tolazoline | 0.794 | 0.664 |
| chloropyramine | etidronic_acid | 0.794 | 0.812 |
| phenazopyridine | repaglinide | 0.794 | 0.768 |
| cycloserine | thiocolchicoside | 0.794 | 0.872 |
| etilefrine | podophyllotoxin | 0.794 | 0.725 |
| medrysone | ursodeoxycholic_acid | 0.794 | 0.664 |
| hydrocortisone | propylthiouracil | 0.794 | 0.779 |
| azacitidine | isoconazole | 0.794 | 0.772 |
| flurbiprofen | testosterone | 0.794 | 0.715 |
| fenoterol | isoetarine | 0.794 | 0.681 |
| butoconazole | quinisocaine | 0.794 | 0.726 |
| pyrazinamide | tropicamide | 0.794 | 0.742 |
| amoxicillin | ursodeoxycholic_acid | 0.794 | 0.710 |
| flumetasone | ketanserin | 0.794 | 0.741 |
| estriol | flunisolide | 0.794 | 0.676 |
| acetylsalicylic_acid | phenindione | 0.794 | 0.660 |
| bromopride | natamycin | 0.794 | 0.812 |

| | | | |
|---|---|---|---|
| ciclopirox | ciclosporin | 0.794 | 0.903 |
| dienestrol | fenoprofen | 0.794 | 0.690 |
| mianserin | trifluoperazine | 0.794 | 0.748 |
| chloropyramine | propantheline_bromic | 0.794 | 0.696 |
| molindone | quinisocaine | 0.794 | 0.689 |
| cefalotin | protriptyline | 0.794 | 0.790 |
| chlorphenesin | midecamycin | 0.794 | 0.865 |
| etoposide | mefloquine | 0.794 | 0.747 |
| fenoprofen | pargyline | 0.794 | 0.701 |
| flumetasone | metaraminol | 0.794 | 0.767 |
| levamisole | propafenone | 0.794 | 0.718 |
| amiodarone | phenazopyridine | 0.794 | 0.736 |
| meclofenamic_acid | metampicillin | 0.794 | 0.733 |
| colchicine | lanatoside_C | 0.794 | 0.858 |
| benfluorex | diltiazem | 0.794 | 0.748 |
| fluorometholone | tropicamide | 0.794 | 0.733 |
| pargyline | pyrvinium | 0.794 | 0.809 |
| nilutamide | primaquine | 0.794 | 0.675 |
| colchicine | fluvoxamine | 0.794 | 0.709 |
| naltrexone | phenazone | 0.794 | 0.755 |
| ketoprofen | tropicamide | 0.794 | 0.692 |
| desoxycortone | metamizole_sodium | 0.794 | 0.720 |
| dipyridamole | miconazole | 0.794 | 0.784 |
| piracetam | pivampicillin | 0.794 | 0.803 |
| bupropion | labetalol | 0.794 | 0.680 |
| azacitidine | demecolcine | 0.794 | 0.770 |
| cinoxacin | fluorometholone | 0.794 | 0.743 |
| amoxicillin | etofenamate | 0.794 | 0.734 |
| aminophenazone | nomifensine | 0.794 | 0.700 |
| mebendazole | trimethoprim | 0.794 | 0.681 |
| hyoscyamine | metacycline | 0.794 | 0.714 |
| bepridil | imipramine | 0.794 | 0.666 |
| hydroxyzine | lisuride | 0.794 | 0.716 |
| levocabastine | liothyronine | 0.794 | 0.717 |
| dextromethorphan | remoxipride | 0.794 | 0.744 |
| metyrapone | tropicamide | 0.794 | 0.673 |
| clomipramine | propranolol | 0.794 | 0.670 |
| dihydroergocristine | maprotiline | 0.794 | 0.803 |
| succinylsulfathiazole | zalcitabine | 0.794 | 0.684 |
| miconazole | testosterone | 0.794 | 0.750 |
| natamycin | procaine | 0.794 | 0.825 |
| meropenem | meticrane | 0.794 | 0.741 |
| tobramycin | ursodeoxycholic_acid | 0.794 | 0.734 |
| cinchocaine | methazolamide | 0.794 | 0.698 |
| capsaicin | flunarizine | 0.794 | 0.727 |
| phenazopyridine | rescinnamine | 0.794 | 0.801 |
| flucytosine | phenformin | 0.794 | 0.693 |
| fendiline | vorinostat | 0.794 | 0.753 |
| irinotecan | repaglinide | 0.794 | 0.776 |
| clopamide | dinoprost | 0.794 | 0.737 |

| | | | |
|---|---|---|---|
| dihydroergotamine | pimozide | 0.794 | 0.733 |
| etoposide | nortriptyline | 0.794 | 0.784 |
| maprotiline | podophyllotoxin | 0.794 | 0.749 |
| etacrynic_acid | proscillaridin | 0.794 | 0.767 |
| metrifonate | sulfacetamide | 0.794 | 0.694 |
| benzethonium_chloride | proscillaridin | 0.794 | 0.787 |
| bisacodyl | cisapride | 0.794 | 0.773 |
| metamizole_sodium | trichlormethiazide | 0.794 | 0.674 |
| cetirizine | phenindione | 0.794 | 0.750 |
| imipenem | liothyronine | 0.794 | 0.721 |
| felbinac | vancomycin | 0.794 | 0.899 |
| benperidol | pridinol | 0.794 | 0.724 |
| levonorgestrel | procyclidine | 0.794 | 0.753 |
| aminohippuric_acid | betaxolol | 0.794 | 0.674 |
| betaxolol | desoxycortone | 0.794 | 0.738 |
| benzonatate | chloramphenicol | 0.794 | 0.833 |
| chlorzoxazone | ketanserin | 0.794 | 0.741 |
| digoxin | dilazep | 0.794 | 0.770 |
| podophyllotoxin | vinburnine | 0.794 | 0.665 |
| progesterone | sulfamethoxypyridazir | 0.794 | 0.742 |
| ethosuximide | fenspiride | 0.794 | 0.741 |
| cefalotin | clidinium_bromide | 0.794 | 0.726 |
| desoxycortone | meptazinol | 0.794 | 0.743 |
| gefitinib | terfenadine | 0.794 | 0.765 |
| benzonatate | sulfaphenazole | 0.794 | 0.784 |
| oxytetracycline | trazodone | 0.794 | 0.755 |
| cyclobenzaprine | phenoxybenzamine | 0.794 | 0.687 |
| propafenone | sulfafurazole | 0.794 | 0.719 |
| piperidolate | protriptyline | 0.794 | 0.654 |
| aminophylline | cefalexin | 0.794 | 0.749 |
| disulfiram | vorinostat | 0.794 | 0.734 |
| levamisole | propofol | 0.794 | 0.652 |
| mebendazole | promethazine | 0.794 | 0.722 |
| oxprenolol | progesterone | 0.794 | 0.767 |
| dorzolamide | ronidazole | 0.794 | 0.733 |
| calcium_pantothenate | cefadroxil | 0.794 | 0.752 |
| etilefrine | mebendazole | 0.794 | 0.665 |
| metyrapone | phenindione | 0.794 | 0.651 |
| mesalazine | ticarcillin | 0.794 | 0.774 |
| chloramphenicol | niridazole | 0.794 | 0.710 |
| cefalexin | ethotoin | 0.794 | 0.709 |
| fenoterol | rimexolone | 0.794 | 0.724 |
| bromperidol | monobenzone | 0.794 | 0.724 |
| nomifensine | terfenadine | 0.794 | 0.760 |
| hydralazine | trazodone | 0.794 | 0.734 |
| bisacodyl | fluorometholone | 0.794 | 0.746 |
| dipyridamole | zomepirac | 0.794 | 0.737 |
| acepromazine | chlortetracycline | 0.794 | 0.755 |
| bisacodyl | promethazine | 0.794 | 0.741 |
| naftidrofuryl | zuclopenthixol | 0.794 | 0.685 |

| | | | |
|---|---|---|---|
| etamsylate | meclozine | 0.794 | 0.808 |
| cetirizine | levamisole | 0.794 | 0.779 |
| levamisole | omeprazole | 0.794 | 0.699 |
| bisacodyl | levamisole | 0.794 | 0.779 |
| clioquinol | trazodone | 0.794 | 0.724 |
| bepridil | lomustine | 0.794 | 0.752 |
| isoxsuprine | miconazole | 0.794 | 0.738 |
| fenoterol | metamizole_sodium | 0.794 | 0.700 |
| aciclovir | ajmaline | 0.794 | 0.744 |
| benzonatate | urapidil | 0.794 | 0.788 |
| gliclazide | proxyphylline | 0.794 | 0.735 |
| menadione | promazine | 0.794 | 0.682 |
| flunisolide | menadione | 0.794 | 0.773 |
| pentoxyverine | propantheline_bromic | 0.794 | 0.698 |
| benperidol | promazine | 0.794 | 0.725 |
| glibenclamide | miconazole | 0.794 | 0.785 |
| glibenclamide | pentetrazol | 0.794 | 0.828 |
| triflusal | zomepirac | 0.794 | 0.688 |
| etofylline | tiratricol | 0.794 | 0.726 |
| phenacetin | roxithromycin | 0.794 | 0.884 |
| betahistine | sulfafurazole | 0.794 | 0.678 |
| metixene | thioproperazine | 0.794 | 0.718 |
| niridazole | roxithromycin | 0.794 | 0.875 |
| carbinoxamine | iopamidol | 0.794 | 0.788 |
| levonorgestrel | pyrantel | 0.794 | 0.777 |
| oxetacaine | procaine | 0.794 | 0.759 |
| budesonide | verteporfin | 0.794 | 0.799 |
| bambuterol | praziquantel | 0.794 | 0.746 |
| clidinium_bromide | zomepirac | 0.794 | 0.724 |
| desipramine | fluspirilene | 0.794 | 0.758 |
| buflomedil | piribedil | 0.794 | 0.700 |
| proxymetacaine | tyloxapol | 0.794 | 0.863 |
| bisacodyl | metacycline | 0.794 | 0.758 |
| cefalexin | clobetasol | 0.794 | 0.706 |
| demecolcine | disopyramide | 0.794 | 0.735 |
| papaverine | piperidolate | 0.794 | 0.742 |
| piromidic_acid | tropicamide | 0.794 | 0.739 |
| flunisolide | levonorgestrel | 0.794 | 0.652 |
| bezafibrate | hyoscyamine | 0.794 | 0.728 |
| flunisolide | phenoxybenzamine | 0.794 | 0.773 |
| aminohippuric_acid | talampicillin | 0.794 | 0.765 |
| acepromazine | tranylcypromine | 0.794 | 0.730 |
| bisacodyl | terbutaline | 0.794 | 0.770 |
| dipyridamole | mitoxantrone | 0.794 | 0.687 |
| acetylsalicylic_acid | pyrantel | 0.794 | 0.713 |
| biotin | rolitetracycline | 0.794 | 0.772 |
| fenoterol | sulfasalazine | 0.794 | 0.715 |
| alfuzosin | phenoxybenzamine | 0.794 | 0.754 |
| nadolol | thiamphenicol | 0.794 | 0.688 |
| lymecycline | metamizole_sodium | 0.794 | 0.751 |

| | | | |
|---|---|---|---|
| fluvoxamine | trazodone | 0.794 | 0.776 |
| simvastatin | trimetazidine | 0.794 | 0.751 |
| cisapride | ergocalciferol | 0.794 | 0.758 |
| lomefloxacin | tolfenamic_acid | 0.794 | 0.669 |
| isotretinoin | topiramate | 0.794 | 0.827 |
| etamivan | pargyline | 0.794 | 0.684 |
| etamsylate | pyrantel | 0.794 | 0.721 |
| bisacodyl | piperidolate | 0.794 | 0.665 |
| cefalotin | doxorubicin | 0.794 | 0.734 |
| pipemidic_acid | tyloxapol | 0.794 | 0.855 |
| fluvastatin | propafenone | 0.794 | 0.724 |
| alimemazine | scopolamine | 0.794 | 0.737 |
| flunarizine | piribedil | 0.794 | 0.722 |
| carbachol | molindone | 0.794 | 0.770 |
| aztreonam | pyrantel | 0.794 | 0.773 |
| rimexolone | sulfadiazine | 0.794 | 0.758 |
| aminohippuric_acid | etidronic_acid | 0.794 | 0.768 |
| flucytosine | milrinone | 0.794 | 0.674 |
| ajmaline | cimetidine | 0.794 | 0.757 |
| lomefloxacin | salbutamol | 0.794 | 0.691 |
| cefalexin | dipivefrine | 0.794 | 0.709 |
| nafcillin | trihexyphenidyl | 0.794 | 0.721 |
| perhexiline | raloxifene | 0.794 | 0.778 |
| cefalexin | pentamidine | 0.794 | 0.753 |
| ketoprofen | lymecycline | 0.794 | 0.754 |
| flumetasone | prilocaine | 0.794 | 0.769 |
| miconazole | promazine | 0.794 | 0.696 |
| etofenamate | etomidate | 0.794 | 0.729 |
| desoxycortone | fenoprofen | 0.794 | 0.703 |
| chloramphenicol | oxprenolol | 0.794 | 0.704 |
| dipivefrine | roxithromycin | 0.794 | 0.801 |
| diltiazem | doxylamine | 0.794 | 0.715 |
| guanfacine | ritodrine | 0.794 | 0.694 |
| cyproterone | etofenamate | 0.794 | 0.727 |
| oxaprozin | phenoxybenzamine | 0.794 | 0.739 |
| alfuzosin | amikacin | 0.794 | 0.782 |
| chloramphenicol | ornidazole | 0.794 | 0.687 |
| meclofenoxate | trazodone | 0.794 | 0.694 |
| cinchocaine | cycloserine | 0.794 | 0.861 |
| spectinomycin | trimetazidine | 0.794 | 0.756 |
| bromperidol | oxybutynin | 0.794 | 0.732 |
| halcinonide | repaglinide | 0.794 | 0.717 |
| gabapentin | isoetarine | 0.794 | 0.667 |
| bromocriptine | trifluridine | 0.794 | 0.779 |
| isoconazole | mianserin | 0.794 | 0.701 |
| miconazole | natamycin | 0.794 | 0.827 |
| aminophenazone | isoconazole | 0.794 | 0.696 |
| medrysone | mephenesin | 0.794 | 0.749 |
| doxylamine | prednisone | 0.794 | 0.761 |
| pipemidic_acid | repaglinide | 0.794 | 0.723 |

| | | | |
|---|---|---|---|
| metamizole_sodium | xylometazoline | 0.794 | 0.707 |
| flucytosine | meglumine | 0.794 | 0.776 |
| glibenclamide | metyrapone | 0.794 | 0.773 |
| mifepristone | oxytetracycline | 0.794 | 0.758 |
| fenofibrate | pimethixene | 0.794 | 0.741 |
| gefitinib | ipratropium_bromide | 0.794 | 0.768 |
| cetirizine | pivampicillin | 0.794 | 0.745 |
| oxprenolol | torasemide | 0.794 | 0.680 |
| bisacodyl | pimethixene | 0.794 | 0.718 |
| clomipramine | methylprednisolone | 0.794 | 0.782 |
| budesonide | miconazole | 0.794 | 0.777 |
| pyrimethamine | trifluoperazine | 0.794 | 0.691 |
| ifenprodil | milrinone | 0.795 | 0.749 |
| dipyridamole | thiocolchicoside | 0.795 | 0.720 |
| cinoxacin | reserpine | 0.795 | 0.751 |
| dirithromycin | trazodone | 0.795 | 0.818 |
| estriol | hydralazine | 0.795 | 0.744 |
| clindamycin | mifepristone | 0.795 | 0.772 |
| bupropion | cinoxacin | 0.795 | 0.655 |
| pivmecillinam | spaglumic_acid | 0.795 | 0.763 |
| flufenamic_acid | sulfinpyrazone | 0.795 | 0.727 |
| mesalazine | rimexolone | 0.795 | 0.800 |
| bumetanide | niflumic_acid | 0.795 | 0.684 |
| benzethonium_chloride | verteporfin | 0.795 | 0.820 |
| benperidol | isoxsuprine | 0.795 | 0.672 |
| pyrazinamide | talampicillin | 0.795 | 0.781 |
| fenoprofen | ganciclovir | 0.795 | 0.694 |
| acepromazine | oxamic_acid | 0.795 | 0.854 |
| econazole | levomepromazine | 0.795 | 0.686 |
| procaine | trazodone | 0.795 | 0.664 |
| flufenamic_acid | ticlopidine | 0.795 | 0.693 |
| flucloxacillin | halofantrine | 0.795 | 0.729 |
| hyoscyamine | ranitidine | 0.795 | 0.755 |
| carbachol | mesoridazine | 0.795 | 0.776 |
| cefixime | practolol | 0.795 | 0.720 |
| papaverine | pyrvinium | 0.795 | 0.754 |
| cefalotin | clomifene | 0.795 | 0.765 |
| ipratropium_bromide | molindone | 0.795 | 0.708 |
| chlorambucil | meptazinol | 0.795 | 0.747 |
| cycloserine | etomidate | 0.795 | 0.807 |
| levocabastine | triprolidine | 0.795 | 0.788 |
| gefitinib | kanamycin | 0.795 | 0.758 |
| cefalotin | sulfinpyrazone | 0.795 | 0.700 |
| podophyllotoxin | zuclopenthixol | 0.795 | 0.746 |
| glibenclamide | methazolamide | 0.795 | 0.768 |
| lisinopril | tropicamide | 0.795 | 0.717 |
| selegiline | sulconazole | 0.795 | 0.716 |
| cefmetazole | milrinone | 0.795 | 0.769 |
| medrysone | oxybuprocaine | 0.795 | 0.737 |
| budesonide | zomepirac | 0.795 | 0.732 |

| | | | |
|---|---|---|---|
| oxaprozin | oxprenolol | 0.795 | 0.695 |
| desoxycortone | methazolamide | 0.795 | 0.739 |
| etamsylate | tobramycin | 0.795 | 0.830 |
| daunorubicin | vorinostat | 0.795 | 0.718 |
| methylergometrine | repaglinide | 0.795 | 0.694 |
| altretamine | carbachol | 0.795 | 0.764 |
| bupivacaine | ifenprodil | 0.795 | 0.731 |
| omeprazole | remoxipride | 0.795 | 0.668 |
| lymecycline | sulfametoxydiazine | 0.795 | 0.775 |
| sulfamerazine | trimetazidine | 0.795 | 0.706 |
| (-)-catechin | ivermectin | 0.795 | 0.860 |
| niclosamide | oxetacaine | 0.795 | 0.738 |
| (-)-catechin | amiodarone | 0.795 | 0.746 |
| pentetrazol | pivampicillin | 0.795 | 0.840 |
| sulfametoxydiazine | trimetazidine | 0.795 | 0.700 |
| dicoumarol | fenoprofen | 0.795 | 0.704 |
| atovaquone | trimetazidine | 0.795 | 0.753 |
| oxetacaine | ticlopidine | 0.795 | 0.783 |
| chloramphenicol | progesterone | 0.795 | 0.759 |
| etodolac | pivampicillin | 0.795 | 0.754 |
| demecolcine | dextromethorphan | 0.795 | 0.730 |
| exemestane | ivermectin | 0.795 | 0.862 |
| ethambutol | sulconazole | 0.795 | 0.779 |
| alimemazine | cefoxitin | 0.795 | 0.784 |
| clofibrate | tioguanine | 0.795 | 0.746 |
| methylergometrine | procainamide | 0.795 | 0.688 |
| letrozole | sulfaphenazole | 0.795 | 0.692 |
| ganciclovir | piperidolate | 0.795 | 0.762 |
| clomifene | desipramine | 0.795 | 0.684 |
| atovaquone | metacycline | 0.795 | 0.709 |
| pimethixene | propafenone | 0.795 | 0.704 |
| clonidine | repaglinide | 0.795 | 0.807 |
| dacarbazine | methylergometrine | 0.795 | 0.700 |
| loperamide | tetryzoline | 0.795 | 0.780 |
| alfuzosin | glibenclamide | 0.795 | 0.697 |
| diltiazem | nifurtimox | 0.795 | 0.738 |
| etofenamate | nifurtimox | 0.795 | 0.691 |
| etamivan | sulconazole | 0.795 | 0.752 |
| pimozide | quinisocaine | 0.795 | 0.756 |
| metaraminol | simvastatin | 0.795 | 0.801 |
| fendiline | ticlopidine | 0.795 | 0.662 |
| latamoxef | pentetic_acid | 0.795 | 0.741 |
| naltrexone | tiapride | 0.795 | 0.747 |
| daunorubicin | syrosingopine | 0.795 | 0.718 |
| ketanserin | sulfamethoxazole | 0.795 | 0.730 |
| desipramine | etoposide | 0.795 | 0.794 |
| estriol | tropicamide | 0.795 | 0.700 |
| carbachol | tobramycin | 0.795 | 0.830 |
| cinchocaine | trimetazidine | 0.795 | 0.712 |
| ketoprofen | trioxysalen | 0.795 | 0.699 |

| | | | |
|---|---|---|---|
| procaine | progesterone | 0.795 | 0.719 |
| clofazimine | imipramine | 0.795 | 0.705 |
| daunorubicin | pyrantel | 0.795 | 0.753 |
| propafenone | suloctidil | 0.795 | 0.704 |
| bufexamac | fenoterol | 0.795 | 0.725 |
| iopanoic_acid | isradipine | 0.795 | 0.691 |
| chlortetracycline | pipemidic_acid | 0.795 | 0.715 |
| ciclosporin | troglitazone | 0.795 | 0.846 |
| carbachol | promazine | 0.795 | 0.794 |
| clomipramine | repaglinide | 0.795 | 0.757 |
| mephentermine | sulfamethoxazole | 0.795 | 0.676 |
| natamycin | proxyphylline | 0.795 | 0.834 |
| latamoxef | metamizole_sodium | 0.795 | 0.756 |
| etofenamate | talampicillin | 0.795 | 0.720 |
| imipramine | lomustine | 0.795 | 0.739 |
| ifenprodil | pyrazinamide | 0.795 | 0.807 |
| naloxone | ronidazole | 0.795 | 0.745 |
| (-)-catechin | meropenem | 0.795 | 0.726 |
| cefalexin | dydrogesterone | 0.795 | 0.737 |
| dicoumarol | mexiletine | 0.795 | 0.696 |
| meclofenoxate | meclozine | 0.795 | 0.694 |
| latamoxef | levamisole | 0.795 | 0.814 |
| haloperidol | mefloquine | 0.795 | 0.736 |
| etamsylate | molindone | 0.795 | 0.721 |
| fluocinonide | promethazine | 0.795 | 0.798 |
| metamizole_sodium | methapyrilene | 0.795 | 0.688 |
| flutamide | omeprazole | 0.795 | 0.688 |
| mephenesin | tropicamide | 0.795 | 0.694 |
| amoxicillin | milrinone | 0.795 | 0.765 |
| bromocriptine | ifenprodil | 0.795 | 0.735 |
| astemizole | gefitinib | 0.795 | 0.724 |
| (-)-catechin | chlorprothixene | 0.795 | 0.725 |
| fluocinonide | sulfamethoxypyridazir | 0.795 | 0.751 |
| levomepromazine | syrosingopine | 0.795 | 0.789 |
| clioquinol | simvastatin | 0.795 | 0.808 |
| flunarizine | piromidic_acid | 0.795 | 0.782 |
| acetylsalicylic_acid | fluorometholone | 0.795 | 0.760 |
| benzylpenicillin | milrinone | 0.795 | 0.768 |
| ioxaglic_acid | nicotinic_acid | 0.795 | 0.864 |
| meptazinol | oxetacaine | 0.795 | 0.799 |
| azacitidine | fulvestrant | 0.795 | 0.824 |
| ranitidine | rimexolone | 0.795 | 0.766 |
| betahistine | mifepristone | 0.795 | 0.826 |
| oxybutynin | practolol | 0.795 | 0.727 |
| cinchocaine | procaine | 0.795 | 0.669 |
| cefalotin | urapidil | 0.795 | 0.728 |
| proscillaridin | quinisocaine | 0.795 | 0.813 |
| doxylamine | pralidoxime | 0.795 | 0.729 |
| butoconazole | pimozide | 0.795 | 0.754 |
| capsaicin | ursodeoxycholic_acid | 0.795 | 0.745 |

| | | | |
|---|---|---|---|
| cetirizine | scopolamine | 0.795 | 0.735 |
| norethisterone | proxyphylline | 0.795 | 0.747 |
| clebopride | metolazone | 0.795 | 0.699 |
| dapsone | isometheptene | 0.795 | 0.729 |
| flecainide | galantamine | 0.795 | 0.711 |
| irinotecan | roxithromycin | 0.795 | 0.804 |
| halcinonide | mesoridazine | 0.795 | 0.758 |
| flupentixol | maprotiline | 0.795 | 0.746 |
| bepridil | iopanoic_acid | 0.795 | 0.747 |
| emetine | isoconazole | 0.795 | 0.740 |
| pivampicillin | sulfametoxydiazine | 0.795 | 0.736 |
| butoconazole | clomifene | 0.795 | 0.754 |
| cefsulodin | meticrane | 0.795 | 0.765 |
| lisinopril | pyrantel | 0.795 | 0.793 |
| capsaicin | medrysone | 0.795 | 0.743 |
| bemegride | bretylium_tosilate | 0.795 | 0.779 |
| hyoscyamine | metyrapone | 0.795 | 0.697 |
| sulconazole | terfenadine | 0.795 | 0.757 |
| clofazimine | protriptyline | 0.795 | 0.728 |
| hyoscyamine | piperidolate | 0.795 | 0.663 |
| cyproterone | glibenclamide | 0.795 | 0.759 |
| calcium_pantothenate | sulfametoxydiazine | 0.795 | 0.786 |
| talampicillin | vidarabine | 0.795 | 0.741 |
| chlorhexidine | danazol | 0.795 | 0.803 |
| isoconazole | picotamide | 0.795 | 0.748 |
| amiodarone | desoxycortone | 0.795 | 0.761 |
| apomorphine | procaine | 0.795 | 0.708 |
| felodipine | primaquine | 0.795 | 0.733 |
| flupentixol | lomustine | 0.795 | 0.776 |
| cefotetan | oxantel | 0.795 | 0.792 |
| daunorubicin | etofenamate | 0.795 | 0.683 |
| phenformin | sulfamerazine | 0.795 | 0.659 |
| citiolone | estriol | 0.795 | 0.780 |
| levamisole | rolitetracycline | 0.795 | 0.796 |
| fenoprofen | sulfamethoxazole | 0.795 | 0.679 |
| clemastine | thioproperazine | 0.795 | 0.715 |
| ambroxol | hexetidine | 0.795 | 0.765 |
| medrysone | reserpine | 0.795 | 0.770 |
| cortisone | pentoxyverine | 0.795 | 0.711 |
| primaquine | zuclopenthixol | 0.795 | 0.677 |
| ketoprofen | verteporfin | 0.795 | 0.791 |
| deptropine | methoxamine | 0.795 | 0.760 |
| mianserin | prenylamine | 0.795 | 0.687 |
| deferoxamine | etamsylate | 0.795 | 0.868 |
| labetalol | oxolinic_acid | 0.795 | 0.701 |
| lanatoside_C | pergolide | 0.795 | 0.881 |
| propafenone | tobramycin | 0.795 | 0.777 |
| methylergometrine | tribenoside | 0.795 | 0.708 |
| benfotiamine | sulfaphenazole | 0.795 | 0.732 |
| digoxin | mianserin | 0.795 | 0.883 |

| | | | |
|---|---|---|---|
| amiodarone | remoxipride | 0.795 | 0.725 |
| budesonide | phenoxybenzamine | 0.795 | 0.771 |
| ioversol | repaglinide | 0.795 | 0.766 |
| carisoprodol | naphazoline | 0.795 | 0.711 |
| flufenamic_acid | rolitetracycline | 0.795 | 0.723 |
| propafenone | proxymetacaine | 0.795 | 0.717 |
| nabumetone | proxyphylline | 0.795 | 0.653 |
| podophyllotoxin | suloctidil | 0.795 | 0.775 |
| imipenem | piperidolate | 0.795 | 0.765 |
| betaxolol | metacycline | 0.795 | 0.734 |
| buflomedil | dydrogesterone | 0.795 | 0.743 |
| dacarbazine | syrosingopine | 0.795 | 0.828 |
| deferoxamine | phenoxybenzamine | 0.795 | 0.847 |
| procaine | triflusal | 0.795 | 0.706 |
| acepromazine | trioxysalen | 0.795 | 0.678 |
| clindamycin | natamycin | 0.795 | 0.769 |
| alprostadil | benzathine_benzylper | 0.795 | 0.736 |
| betaxolol | fluocinonide | 0.795 | 0.774 |
| meclozine | pentamidine | 0.795 | 0.763 |
| oxolinic_acid | talampicillin | 0.795 | 0.766 |
| flunisolide | imipenem | 0.795 | 0.724 |
| aminohippuric_acid | levonorgestrel | 0.795 | 0.686 |
| galantamine | pivampicillin | 0.795 | 0.787 |
| nicotinic_acid | sulconazole | 0.795 | 0.805 |
| chlorcyclizine | fluphenazine | 0.795 | 0.732 |
| chlortetracycline | piribedil | 0.795 | 0.734 |
| doxorubicin | trimetazidine | 0.795 | 0.742 |
| benzylpenicillin | proscillaridin | 0.795 | 0.765 |
| bepridil | mepacrine | 0.795 | 0.741 |
| aminohippuric_acid | doxorubicin | 0.795 | 0.685 |
| enoxacin | etofenamate | 0.795 | 0.674 |
| hydrocortisone | meropenem | 0.795 | 0.695 |
| capsaicin | molindone | 0.795 | 0.713 |
| captopril | sulpiride | 0.795 | 0.750 |
| aminohippuric_acid | meclozine | 0.795 | 0.761 |
| capsaicin | pentoxyverine | 0.795 | 0.736 |
| diflorasone | letrozole | 0.795 | 0.755 |
| deferoxamine | meticrane | 0.795 | 0.854 |
| metamizole_sodium | naloxone | 0.795 | 0.685 |
| etofylline | natamycin | 0.795 | 0.846 |
| methocarbamol | triflusal | 0.795 | 0.698 |
| cinnarizine | sulfinpyrazone | 0.795 | 0.709 |
| benzylpenicillin | reserpine | 0.795 | 0.743 |
| bisacodyl | zomepirac | 0.795 | 0.712 |
| naftidrofuryl | urapidil | 0.795 | 0.737 |
| benzylpenicillin | sulfanilamide | 0.795 | 0.748 |
| cefalotin | cyproterone | 0.795 | 0.753 |
| cefuroxime | quinidine | 0.795 | 0.722 |
| doxazosin | pentetrazol | 0.795 | 0.801 |
| cinchocaine | pentoxyverine | 0.795 | 0.722 |

| | | | |
|---|---|---|---|
| bezafibrate | estrone | 0.795 | 0.737 |
| mometasone | oxyphenbutazone | 0.795 | 0.724 |
| mitoxantrone | piracetam | 0.795 | 0.799 |
| acetylsalicylic_acid | diltiazem | 0.795 | 0.752 |
| acetylsalicylic_acid | atovaquone | 0.795 | 0.673 |
| amoxicillin | estriol | 0.795 | 0.731 |
| chlorpropamide | methapyrilene | 0.795 | 0.701 |
| dextromethorphan | estriol | 0.795 | 0.726 |
| etacrynic_acid | tyloxapol | 0.795 | 0.852 |
| bromopride | ipratropium_bromide | 0.795 | 0.712 |
| chlorcyclizine | clioquinol | 0.795 | 0.752 |
| mianserin | terfenadine | 0.795 | 0.762 |
| sulindac | tacrolimus | 0.795 | 0.809 |
| daunorubicin | lymecycline | 0.795 | 0.677 |
| theophylline | trimipramine | 0.795 | 0.785 |
| benfotiamine | urapidil | 0.795 | 0.693 |
| fenoterol | sulfafurazole | 0.795 | 0.682 |
| apomorphine | sulfafurazole | 0.795 | 0.726 |
| amoxicillin | dacarbazine | 0.795 | 0.749 |
| ciclosporin | clemastine | 0.795 | 0.880 |
| acetohexamide | tolfenamic_acid | 0.795 | 0.721 |
| bezafibrate | omeprazole | 0.795 | 0.680 |
| citiolone | liothyronine | 0.795 | 0.811 |
| pivampicillin | rolitetracycline | 0.795 | 0.681 |
| clomipramine | fenoterol | 0.795 | 0.770 |
| aminophenazone | octopamine | 0.795 | 0.711 |
| fenoprofen | fludroxycortide | 0.795 | 0.739 |
| fluspirilene | medrysone | 0.795 | 0.724 |
| pyrantel | ranitidine | 0.795 | 0.745 |
| capsaicin | minaprine | 0.795 | 0.675 |
| cetirizine | labetalol | 0.795 | 0.678 |
| betahistine | pridinol | 0.795 | 0.683 |
| oxybutynin | terfenadine | 0.795 | 0.714 |
| menadione | metyrapone | 0.795 | 0.660 |
| bepridil | ipratropium_bromide | 0.795 | 0.734 |
| mometasone | pimozide | 0.795 | 0.750 |
| etofylline | trimetazidine | 0.795 | 0.651 |
| latamoxef | norethisterone | 0.795 | 0.785 |
| metoprolol | tetryzoline | 0.795 | 0.748 |
| clioquinol | trifluoperazine | 0.795 | 0.724 |
| glibenclamide | procaine | 0.795 | 0.746 |
| propylthiouracil | vinpocetine | 0.795 | 0.747 |
| dacarbazine | dextromethorphan | 0.795 | 0.761 |
| butoconazole | metixene | 0.795 | 0.680 |
| bezafibrate | gliclazide | 0.795 | 0.700 |
| reserpine | sulfafurazole | 0.795 | 0.779 |
| dosulepin | econazole | 0.795 | 0.707 |
| hexetidine | rescinnamine | 0.795 | 0.788 |
| benperidol | sulfametoxydiazine | 0.795 | 0.713 |
| propylthiouracil | zomepirac | 0.795 | 0.704 |

| | | | |
|---|---|---|---|
| estriol | lymecycline | 0.795 | 0.779 |
| ganciclovir | metaraminol | 0.795 | 0.673 |
| aminohippuric_acid | lymecycline | 0.795 | 0.771 |
| bepridil | vidarabine | 0.795 | 0.775 |
| propylthiouracil | ursodeoxycholic_acid | 0.795 | 0.796 |
| flunisolide | omeprazole | 0.796 | 0.728 |
| deferoxamine | oxetacaine | 0.796 | 0.789 |
| hydralazine | meclozine | 0.796 | 0.786 |
| levonorgestrel | prilocaine | 0.796 | 0.759 |
| chloramphenicol | procaine | 0.796 | 0.673 |
| bisacodyl | protriptyline | 0.796 | 0.710 |
| chlortetracycline | clopamide | 0.796 | 0.723 |
| calcium_pantothenate | levonorgestrel | 0.796 | 0.799 |
| piracetam | sulfametoxydiazine | 0.796 | 0.695 |
| hyoscyamine | repaglinide | 0.796 | 0.749 |
| fluphenazine | trimipramine | 0.796 | 0.715 |
| remoxipride | tyloxapol | 0.796 | 0.862 |
| desoxycortone | fluvoxamine | 0.796 | 0.715 |
| erastin | ivermectin | 0.796 | 0.802 |
| (-)-catechin | meclofenoxate | 0.796 | 0.678 |
| pridinol | propylthiouracil | 0.796 | 0.734 |
| hyoscyamine | pivampicillin | 0.796 | 0.687 |
| fluspirilene | urapidil | 0.796 | 0.696 |
| artemisinin | budesonide | 0.796 | 0.759 |
| ioversol | medrysone | 0.796 | 0.766 |
| diethylcarbamazine | doxepin | 0.796 | 0.705 |
| flunisolide | labetalol | 0.796 | 0.741 |
| dipivefrine | simvastatin | 0.796 | 0.695 |
| azacitidine | metacycline | 0.796 | 0.671 |
| hydrocortisone | vidarabine | 0.796 | 0.746 |
| (-)-isoprenaline | astemizole | 0.796 | 0.769 |
| fenspiride | verteporfin | 0.796 | 0.791 |
| lomustine | miconazole | 0.796 | 0.745 |
| dipivefrine | mebendazole | 0.796 | 0.738 |
| calcium_pantothenate | repaglinide | 0.796 | 0.799 |
| amiodarone | flumetasone | 0.796 | 0.771 |
| amoxicillin | gliclazide | 0.796 | 0.666 |
| acetohexamide | cortisone | 0.796 | 0.724 |
| doxorubicin | nitrendipine | 0.796 | 0.721 |
| aminohippuric_acid | liothyronine | 0.796 | 0.735 |
| homatropine | topiramate | 0.796 | 0.739 |
| flumetasone | metoclopramide | 0.796 | 0.737 |
| bepridil | metixene | 0.796 | 0.678 |
| dacarbazine | oxamniquine | 0.796 | 0.663 |
| maprotiline | rescinnamine | 0.796 | 0.808 |
| colecalciferol | promazine | 0.796 | 0.752 |
| cyanocobalamin | ronidazole | 0.796 | 0.877 |
| pentamidine | tiabendazole | 0.796 | 0.740 |
| chloramphenicol | metamizole_sodium | 0.796 | 0.714 |
| cinchocaine | pargyline | 0.796 | 0.752 |

| | | | |
|---|---|---|---|
| gabapentin | vidarabine | 0.796 | 0.726 |
| gliclazide | phenazopyridine | 0.796 | 0.691 |
| ioversol | piribedil | 0.796 | 0.790 |
| hydrocortisone | tropicamide | 0.796 | 0.743 |
| hyoscyamine | sulfadiazine | 0.796 | 0.655 |
| cyclopentolate | sulfametoxydiazine | 0.796 | 0.694 |
| cefixime | metamizole_sodium | 0.796 | 0.717 |
| capsaicin | piribedil | 0.796 | 0.691 |
| mepacrine | phenoxybenzamine | 0.796 | 0.747 |
| dipivefrine | dosulepin | 0.796 | 0.739 |
| capsaicin | propylthiouracil | 0.796 | 0.736 |
| meropenem | remoxipride | 0.796 | 0.727 |
| bufexamac | trioxysalen | 0.796 | 0.700 |
| apomorphine | flucytosine | 0.796 | 0.749 |
| glycopyrronium_bromide | sulfadimethoxine | 0.796 | 0.693 |
| amoxapine | pyrvinium | 0.796 | 0.799 |
| betamethasone | oxantel | 0.796 | 0.757 |
| alimemazine | clomifene | 0.796 | 0.729 |
| calcium_pantothenate | metyrapone | 0.796 | 0.806 |
| flucytosine | zomepirac | 0.796 | 0.778 |
| lynestrenol | oxetacaine | 0.796 | 0.764 |
| deptropine | econazole | 0.796 | 0.668 |
| metamizole_sodium | pyrazinamide | 0.796 | 0.707 |
| apomorphine | scopolamine | 0.796 | 0.753 |
| irinotecan | propofol | 0.796 | 0.822 |
| dacarbazine | promazine | 0.796 | 0.727 |
| gliclazide | tiabendazole | 0.796 | 0.731 |
| ifenprodil | practolol | 0.796 | 0.715 |
| mianserin | nicergoline | 0.796 | 0.759 |
| fenoterol | trazodone | 0.796 | 0.688 |
| mepyramine | meticrane | 0.796 | 0.747 |
| bromopride | chlorzoxazone | 0.796 | 0.702 |
| norethisterone | sertaconazole | 0.796 | 0.734 |
| alimemazine | pyrithyldione | 0.796 | 0.756 |
| glafenine | hydroflumethiazide | 0.796 | 0.746 |
| terconazole | trazodone | 0.796 | 0.729 |
| carmustine | orlistat | 0.796 | 0.804 |
| proxyphylline | remoxipride | 0.796 | 0.691 |
| oxamniquine | phenacetin | 0.796 | 0.680 |
| practolol | remoxipride | 0.796 | 0.715 |
| iopanoic_acid | metoprolol | 0.796 | 0.687 |
| procyclidine | sulfamethoxazole | 0.796 | 0.684 |
| phenelzine | tinidazole | 0.796 | 0.652 |
| tobramycin | tolfenamic_acid | 0.796 | 0.796 |
| maprotiline | noretynodrel | 0.796 | 0.721 |
| terfenadine | verapamil | 0.796 | 0.712 |
| budesonide | fenoterol | 0.796 | 0.714 |
| cycloserine | propylthiouracil | 0.796 | 0.752 |
| repaglinide | ticarcillin | 0.796 | 0.746 |
| clobetasol | piperidolate | 0.796 | 0.728 |

| | | | |
|---|---|---|---|
| lynestrenol | perphenazine | 0.796 | 0.760 |
| azapropazone | ribavirin | 0.796 | 0.706 |
| mebendazole | primaquine | 0.796 | 0.678 |
| estriol | ticarcillin | 0.796 | 0.729 |
| dirithromycin | repaglinide | 0.796 | 0.783 |
| biotin | naftidrofuryl | 0.796 | 0.767 |
| chlorambucil | methylergometrine | 0.796 | 0.681 |
| cetirizine | sulpiride | 0.796 | 0.742 |
| enoxacin | pralidoxime | 0.796 | 0.705 |
| atovaquone | chlorzoxazone | 0.796 | 0.745 |
| ketoprofen | tyloxapol | 0.796 | 0.864 |
| cinoxacin | sulfamethoxypyridazir | 0.796 | 0.687 |
| pivampicillin | tiapride | 0.796 | 0.741 |
| gliclazide | terbutaline | 0.796 | 0.730 |
| methapyrilene | monobenzone | 0.796 | 0.705 |
| dipivefrine | remoxipride | 0.796 | 0.717 |
| piribedil | ursodeoxycholic_acid | 0.796 | 0.734 |
| pyrithyldione | zuclopenthixol | 0.796 | 0.811 |
| semustine | terfenadine | 0.796 | 0.786 |
| betahistine | cycloserine | 0.796 | 0.773 |
| acetylsalicylic_acid | chlorambucil | 0.796 | 0.773 |
| metacycline | piperidolate | 0.796 | 0.756 |
| demecolcine | natamycin | 0.796 | 0.785 |
| bupropion | triflusal | 0.796 | 0.745 |
| cycloserine | dacarbazine | 0.796 | 0.737 |
| felodipine | sulfamethoxypyridazir | 0.796 | 0.705 |
| riluzole | tropicamide | 0.796 | 0.698 |
| (-)-catechin | hydroxyzine | 0.796 | 0.763 |
| ciclosporin | loperamide | 0.796 | 0.829 |
| amiodarone | tinidazole | 0.796 | 0.747 |
| betahistine | pyrvinium | 0.796 | 0.835 |
| diltiazem | ethosuximide | 0.796 | 0.801 |
| guanfacine | procainamide | 0.796 | 0.683 |
| procaine | ronidazole | 0.796 | 0.652 |
| galantamine | sulfaphenazole | 0.796 | 0.718 |
| etofenamate | norethisterone | 0.796 | 0.756 |
| oxprenolol | pridinol | 0.796 | 0.681 |
| ethotoin | roxithromycin | 0.796 | 0.870 |
| oxyphenbutazone | zuclopenthixol | 0.796 | 0.715 |
| flutamide | tolbutamide | 0.796 | 0.696 |
| baclofen | clopamide | 0.796 | 0.706 |
| dyclonine | flunarizine | 0.796 | 0.751 |
| rimexolone | tridihexethyl | 0.796 | 0.719 |
| deferoxamine | flunisolide | 0.796 | 0.812 |
| cefalexin | methapyrilene | 0.796 | 0.755 |
| clioquinol | proscillaridin | 0.796 | 0.846 |
| mitoxantrone | propylthiouracil | 0.796 | 0.720 |
| cetirizine | flunisolide | 0.796 | 0.752 |
| etofenamate | iopanoic_acid | 0.796 | 0.715 |
| flumetasone | promethazine | 0.796 | 0.785 |

| | | | |
|---|---|---|---|
| cefalexin | thiocolchicoside | 0.796 | 0.746 |
| cefadroxil | cinchocaine | 0.796 | 0.719 |
| altizide | chlorphenesin | 0.796 | 0.709 |
| benzocaine | vancomycin | 0.796 | 0.907 |
| levamisole | metacycline | 0.796 | 0.768 |
| cortisone | ifenprodil | 0.796 | 0.727 |
| bromopride | rolitetracycline | 0.796 | 0.759 |
| pentamidine | pyrvinium | 0.796 | 0.761 |
| bromperidol | piromidic_acid | 0.796 | 0.724 |
| meticrane | thiocolchicoside | 0.796 | 0.778 |
| etofenamate | procainamide | 0.796 | 0.696 |
| dacarbazine | sulfamethoxazole | 0.796 | 0.688 |
| irinotecan | metamizole_sodium | 0.796 | 0.721 |
| cyproterone | flunisolide | 0.796 | 0.656 |
| cortisone | omeprazole | 0.796 | 0.736 |
| ifenprodil | medrysone | 0.796 | 0.704 |
| pargyline | propafenone | 0.796 | 0.746 |
| bepridil | metacycline | 0.796 | 0.756 |
| clemastine | fenofibrate | 0.796 | 0.737 |
| picotamide | rimexolone | 0.796 | 0.746 |
| daunorubicin | imipenem | 0.796 | 0.744 |
| dinoprost | trifluoperazine | 0.796 | 0.728 |
| molsidomine | pralidoxime | 0.796 | 0.729 |
| ethosuximide | imipenem | 0.796 | 0.756 |
| fluorometholone | tyloxapol | 0.796 | 0.853 |
| etamsylate | latamoxef | 0.796 | 0.813 |
| clemastine | clofazimine | 0.796 | 0.722 |
| acetylsalicylic_acid | trichlormethiazide | 0.796 | 0.707 |
| azlocillin | sulfanilamide | 0.796 | 0.793 |
| butoconazole | medrysone | 0.796 | 0.762 |
| cefalotin | medrysone | 0.796 | 0.717 |
| budesonide | roxithromycin | 0.796 | 0.791 |
| amitriptyline | fenoterol | 0.796 | 0.757 |
| fluvastatin | piperidolate | 0.796 | 0.735 |
| dipyridamole | methazolamide | 0.796 | 0.750 |
| decitabine | enalapril | 0.796 | 0.758 |
| lymecycline | tyloxapol | 0.796 | 0.802 |
| bisacodyl | iopanoic_acid | 0.796 | 0.748 |
| aminohippuric_acid | clindamycin | 0.796 | 0.756 |
| flumetasone | monobenzone | 0.796 | 0.784 |
| metyrapone | piribedil | 0.796 | 0.688 |
| chloramphenicol | levocabastine | 0.796 | 0.739 |
| bromperidol | pimozide | 0.796 | 0.675 |
| chlorprothixene | dyclonine | 0.796 | 0.733 |
| diltiazem | methapyrilene | 0.796 | 0.720 |
| omeprazole | pentamidine | 0.796 | 0.727 |
| etomidate | scopolamine | 0.796 | 0.696 |
| menadione | trimipramine | 0.796 | 0.755 |
| gefitinib | isoxsuprine | 0.796 | 0.728 |
| cyproheptadine | fendiline | 0.796 | 0.679 |

| | | | |
|---|---|---|---|
| amiodarone | imipenem | 0.796 | 0.763 |
| meclofenoxate | phenformin | 0.796 | 0.655 |
| dipyridamole | propofol | 0.796 | 0.788 |
| fludroxycortide | practolol | 0.796 | 0.759 |
| sulfafurazole | trimetazidine | 0.796 | 0.710 |
| fenoprofen | ranitidine | 0.796 | 0.739 |
| diltiazem | sulfamethoxypyridazir | 0.796 | 0.711 |
| amiodarone | propafenone | 0.796 | 0.726 |
| fluvoxamine | levonorgestrel | 0.796 | 0.735 |
| cycloserine | promethazine | 0.796 | 0.929 |
| cefalotin | etofylline | 0.796 | 0.740 |
| levamisole | miconazole | 0.796 | 0.729 |
| altizide | labetalol | 0.796 | 0.681 |
| meptazinol | rimexolone | 0.796 | 0.764 |
| (-)-catechin | fenoprofen | 0.796 | 0.697 |
| acepromazine | methylergometrine | 0.796 | 0.669 |
| ganciclovir | tropicamide | 0.796 | 0.690 |
| econazole | mebendazole | 0.796 | 0.737 |
| dacarbazine | gefitinib | 0.796 | 0.728 |
| capsaicin | desoxycortone | 0.796 | 0.759 |
| glibenclamide | nialamide | 0.796 | 0.714 |
| benzylpenicillin | ioxaglic_acid | 0.796 | 0.800 |
| alfaxalone | zimeldine | 0.796 | 0.757 |
| protriptyline | quinisocaine | 0.796 | 0.682 |
| meclofenoxate | monobenzone | 0.796 | 0.684 |
| cetirizine | fenspiride | 0.796 | 0.687 |
| metacycline | oxetacaine | 0.796 | 0.725 |
| mebendazole | metaraminol | 0.796 | 0.726 |
| ceftazidime | prenylamine | 0.796 | 0.769 |
| iocetamic_acid | oxolamine | 0.796 | 0.750 |
| bromopride | liothyronine | 0.796 | 0.724 |
| bromperidol | piribedil | 0.796 | 0.687 |
| irinotecan | nilutamide | 0.796 | 0.721 |
| carbachol | ipratropium_bromide | 0.796 | 0.742 |
| dobutamine | xylometazoline | 0.796 | 0.756 |
| clopamide | dobutamine | 0.796 | 0.730 |
| cefalotin | flufenamic_acid | 0.796 | 0.713 |
| aminohippuric_acid | pivampicillin | 0.796 | 0.747 |
| gliquidone | ketorolac | 0.796 | 0.740 |
| metacycline | mitoxantrone | 0.796 | 0.691 |
| quinisocaine | talampicillin | 0.796 | 0.766 |
| calcium_pantothenate | pargyline | 0.797 | 0.855 |
| ethotoin | trioxysalen | 0.797 | 0.673 |
| fenspiride | tyloxapol | 0.797 | 0.866 |
| carbachol | fipexide | 0.797 | 0.768 |
| omeprazole | tiapride | 0.797 | 0.694 |
| clofazimine | levomepromazine | 0.797 | 0.659 |
| flunisolide | proscillaridin | 0.797 | 0.748 |
| promethazine | propafenone | 0.797 | 0.714 |
| cefuroxime | tropine | 0.797 | 0.852 |

| | | | |
|---|---|---|---|
| amiodarone | selegiline | 0.797 | 0.759 |
| hydralazine | irinotecan | 0.797 | 0.792 |
| benfluorex | methoxamine | 0.797 | 0.744 |
| cyproterone | irinotecan | 0.797 | 0.757 |
| fludroxycortide | metamizole_sodium | 0.797 | 0.694 |
| metolazone | oleandomycin | 0.797 | 0.811 |
| ifenprodil | piracetam | 0.797 | 0.770 |
| benfluorex | propantheline_bromic | 0.797 | 0.738 |
| desoxycortone | pivampicillin | 0.797 | 0.755 |
| bumetanide | pergolide | 0.797 | 0.718 |
| cyanocobalamin | sulfamethoxazole | 0.797 | 0.879 |
| (-)-catechin | trimetazidine | 0.797 | 0.657 |
| sirolimus | sodium_phenylbutyrat | 0.797 | 0.889 |
| cyanocobalamin | tiapride | 0.797 | 0.874 |
| decitabine | pyrvinium | 0.797 | 0.833 |
| carbachol | ticlopidine | 0.797 | 0.756 |
| clindamycin | levonorgestrel | 0.797 | 0.775 |
| niclosamide | promethazine | 0.797 | 0.707 |
| acetylsalicylic_acid | promethazine | 0.797 | 0.720 |
| etamsylate | flunisolide | 0.797 | 0.759 |
| dydrogesterone | tropicamide | 0.797 | 0.716 |
| gefitinib | hydroxyzine | 0.797 | 0.739 |
| aminophenazone | astemizole | 0.797 | 0.730 |
| (-)-atenolol | nicotinic_acid | 0.797 | 0.714 |
| flumetasone | propylthiouracil | 0.797 | 0.778 |
| primaquine | scopolamine | 0.797 | 0.689 |
| fipexide | talampicillin | 0.797 | 0.709 |
| flupentixol | spiramycin | 0.797 | 0.801 |
| metyrapone | naloxone | 0.797 | 0.701 |
| oxybuprocaine | sulfaguanidine | 0.797 | 0.666 |
| bisoprolol | zidovudine | 0.797 | 0.706 |
| moroxydine | practolol | 0.797 | 0.683 |
| acetylsalicylic_acid | cetirizine | 0.797 | 0.799 |
| buflomedil | zimeldine | 0.797 | 0.721 |
| pentoxyverine | sulfaphenazole | 0.797 | 0.727 |
| ciprofibrate | trimipramine | 0.797 | 0.719 |
| meclozine | niclosamide | 0.797 | 0.744 |
| sulfaphenazole | troleandomycin | 0.797 | 0.831 |
| fenoprofen | fluocinonide | 0.797 | 0.772 |
| levonorgestrel | ronidazole | 0.797 | 0.737 |
| glibenclamide | mifepristone | 0.797 | 0.791 |
| fluvastatin | remoxipride | 0.797 | 0.694 |
| hyoscyamine | lisinopril | 0.797 | 0.729 |
| bromperidol | methapyrilene | 0.797 | 0.761 |
| papaverine | repaglinide | 0.797 | 0.748 |
| ketorolac | repaglinide | 0.797 | 0.714 |
| ifenprodil | verteporfin | 0.797 | 0.791 |
| acepromazine | reserpine | 0.797 | 0.764 |
| fenoterol | proxymetacaine | 0.797 | 0.666 |
| colchicine | proxymetacaine | 0.797 | 0.738 |

| | | | |
|---|---|---|---|
| lanatoside_C | trimipramine | 0.797 | 0.886 |
| haloperidol | metergoline | 0.797 | 0.670 |
| molsidomine | triflusal | 0.797 | 0.734 |
| alclometasone | phenazopyridine | 0.797 | 0.761 |
| fendiline | irinotecan | 0.797 | 0.803 |
| loperamide | rifabutin | 0.797 | 0.782 |
| oxybuprocaine | ticlopidine | 0.797 | 0.753 |
| ivermectin | meptazinol | 0.797 | 0.884 |
| meptazinol | nifurtimox | 0.797 | 0.694 |
| deptropine | oxybuprocaine | 0.797 | 0.752 |
| imipenem | pyrvinium | 0.797 | 0.797 |
| clomipramine | mometasone | 0.797 | 0.787 |
| clotrimazole | emetine | 0.797 | 0.789 |
| dipyridamole | oxamic_acid | 0.797 | 0.884 |
| bufexamac | bupivacaine | 0.797 | 0.724 |
| betahistine | spectinomycin | 0.797 | 0.782 |
| acetylsalicylic_acid | sulfamethoxypyridazir | 0.797 | 0.684 |
| felbinac | pyrithyldione | 0.797 | 0.775 |
| haloperidol | imipramine | 0.797 | 0.778 |
| galantamine | isoxsuprine | 0.797 | 0.747 |
| fipexide | ronidazole | 0.797 | 0.742 |
| (-)-isoprenaline | piretanide | 0.797 | 0.695 |
| raloxifene | tamoxifen | 0.797 | 0.723 |
| dacarbazine | propafenone | 0.797 | 0.754 |
| benfotiamine | simvastatin | 0.797 | 0.729 |
| amiodarone | meclofenoxate | 0.797 | 0.744 |
| ciclopirox | econazole | 0.797 | 0.741 |
| nystatin | primaquine | 0.797 | 0.864 |
| demecolcine | flufenamic_acid | 0.797 | 0.701 |
| guanfacine | ketoconazole | 0.797 | 0.800 |
| phenazopyridine | talampicillin | 0.797 | 0.775 |
| tobramycin | tropicamide | 0.797 | 0.781 |
| sulfapyridine | xylometazoline | 0.797 | 0.729 |
| ceforanide | nitrofural | 0.797 | 0.784 |
| cyproterone | torasemide | 0.797 | 0.735 |
| etidronic_acid | reserpine | 0.797 | 0.840 |
| etamivan | talampicillin | 0.797 | 0.783 |
| menadione | mepacrine | 0.797 | 0.733 |
| betaxolol | hydrocortisone | 0.797 | 0.746 |
| amiodarone | ketanserin | 0.797 | 0.711 |
| clioquinol | megestrol | 0.797 | 0.796 |
| methylergometrine | niclosamide | 0.797 | 0.671 |
| ifenprodil | piribedil | 0.797 | 0.711 |
| benfotiamine | pivampicillin | 0.797 | 0.724 |
| amiloride | tyloxapol | 0.797 | 0.885 |
| desoxycortone | meclofenoxate | 0.797 | 0.758 |
| phenelzine | tolbutamide | 0.797 | 0.658 |
| cefalexin | ifenprodil | 0.797 | 0.701 |
| dipyridamole | methylergometrine | 0.797 | 0.728 |
| clomifene | promethazine | 0.797 | 0.761 |

| | | | |
|---|---|---|---|
| bepridil | trazodone | 0.797 | 0.738 |
| ambroxol | furosemide | 0.797 | 0.697 |
| clofazimine | miconazole | 0.797 | 0.730 |
| mitoxantrone | sulfamethoxypyridazir | 0.797 | 0.753 |
| astemizole | bisacodyl | 0.797 | 0.697 |
| ivermectin | profenamine | 0.797 | 0.866 |
| galantamine | omeprazole | 0.797 | 0.728 |
| cinoxacin | dextromethorphan | 0.797 | 0.703 |
| guanethidine | ketanserin | 0.797 | 0.747 |
| etamsylate | zomepirac | 0.797 | 0.732 |
| dipyridamole | piromidic_acid | 0.797 | 0.677 |
| apomorphine | phenoxybenzamine | 0.797 | 0.747 |
| isopropamide_iodide | levothyroxine_sodium | 0.797 | 0.759 |
| metixene | pilocarpine | 0.797 | 0.725 |
| aztreonam | rimexolone | 0.797 | 0.737 |
| etidronic_acid | piperidolate | 0.797 | 0.808 |
| labetalol | progesterone | 0.797 | 0.748 |
| dirithromycin | triflusal | 0.797 | 0.853 |
| clebopride | dextromethorphan | 0.797 | 0.761 |
| bromperidol | verteporfin | 0.797 | 0.788 |
| acepromazine | piribedil | 0.797 | 0.725 |
| risperidone | torasemide | 0.797 | 0.730 |
| bendroflumethiazide | dicoumarol | 0.797 | 0.676 |
| clindamycin | milrinone | 0.797 | 0.793 |
| hyoscyamine | trioxysalen | 0.797 | 0.742 |
| benzylpenicillin | etomidate | 0.797 | 0.666 |
| etoposide | reserpine | 0.797 | 0.736 |
| protriptyline | remoxipride | 0.797 | 0.725 |
| netilmicin | pivmecillinam | 0.797 | 0.724 |
| rolitetracycline | zidovudine | 0.797 | 0.736 |
| cefoxitin | tetryzoline | 0.797 | 0.795 |
| reserpine | terconazole | 0.797 | 0.749 |
| beclometasone | simvastatin | 0.797 | 0.741 |
| acepromazine | alfaxalone | 0.797 | 0.714 |
| cefalexin | rolitetracycline | 0.797 | 0.713 |
| clonidine | fipexide | 0.797 | 0.747 |
| ketanserin | trimetazidine | 0.797 | 0.747 |
| irinotecan | mefloquine | 0.797 | 0.779 |
| levonorgestrel | nitrofurantoin | 0.797 | 0.710 |
| minaprine | sulconazole | 0.797 | 0.719 |
| galantamine | verteporfin | 0.797 | 0.809 |
| latamoxef | ornidazole | 0.797 | 0.736 |
| iodixanol | trimipramine | 0.797 | 0.868 |
| oxybuprocaine | suprofen | 0.797 | 0.706 |
| (-)-atenolol | felodipine | 0.797 | 0.747 |
| etamivan | repaglinide | 0.797 | 0.766 |
| etomidate | felodipine | 0.797 | 0.697 |
| thiocolchicoside | tranylcypromine | 0.797 | 0.827 |
| benzylpenicillin | fenoterol | 0.797 | 0.662 |
| etofylline | trazodone | 0.797 | 0.723 |

| | | | |
|---|---|---|---|
| dihydroergotamine | niclosamide | 0.797 | 0.764 |
| repaglinide | simvastatin | 0.797 | 0.723 |
| amiodarone | bepridil | 0.797 | 0.726 |
| cyproheptadine | ifenprodil | 0.797 | 0.784 |
| latamoxef | pivampicillin | 0.797 | 0.699 |
| cetirizine | cyproheptadine | 0.797 | 0.744 |
| cefalotin | propofol | 0.797 | 0.808 |
| practolol | tranylcypromine | 0.797 | 0.692 |
| dienestrol | terfenadine | 0.797 | 0.809 |
| dacarbazine | ursodeoxycholic_acid | 0.797 | 0.800 |
| bepridil | hyoscyamine | 0.797 | 0.750 |
| gliclazide | metamizole_sodium | 0.797 | 0.686 |
| sulfametoxydiazine | urapidil | 0.797 | 0.735 |
| galantamine | meticrane | 0.797 | 0.700 |
| benzethonium_chloride | econazole | 0.797 | 0.734 |
| acepromazine | rolitetracycline | 0.797 | 0.766 |
| doxepin | flunarizine | 0.797 | 0.743 |
| cycloserine | hydralazine | 0.797 | 0.742 |
| benperidol | irinotecan | 0.797 | 0.711 |
| benzylpenicillin | ketoprofen | 0.797 | 0.661 |
| cefadroxil | propylthiouracil | 0.797 | 0.746 |
| (-)-isoprenaline | benzethonium_chloric | 0.797 | 0.783 |
| clobetasol | roxithromycin | 0.797 | 0.789 |
| fendiline | phenazopyridine | 0.797 | 0.720 |
| streptomycin | topiramate | 0.797 | 0.778 |
| diltiazem | zomepirac | 0.797 | 0.729 |
| menadione | nitrofurantoin | 0.797 | 0.668 |
| estriol | oxolinic_acid | 0.797 | 0.672 |
| nomifensine | pyrantel | 0.797 | 0.704 |
| amitriptyline | phenoxybenzamine | 0.797 | 0.686 |
| clomipramine | etamivan | 0.797 | 0.723 |
| fluspirilene | gefitinib | 0.797 | 0.747 |
| altretamine | reserpine | 0.797 | 0.794 |
| cefalexin | cycloserine | 0.797 | 0.902 |
| etacrynic_acid | podophyllotoxin | 0.797 | 0.724 |
| iproniazid | tolfenamic_acid | 0.797 | 0.657 |
| perphenazine | semustine | 0.797 | 0.760 |
| simvastatin | trioxysalen | 0.797 | 0.777 |
| ivermectin | oxetacaine | 0.797 | 0.802 |
| glibenclamide | ioversol | 0.797 | 0.791 |
| clomipramine | cyproterone | 0.797 | 0.756 |
| irinotecan | mebendazole | 0.797 | 0.715 |
| acetylsalicylic_acid | fenoprofen | 0.797 | 0.665 |
| buflomedil | chlortetracycline | 0.797 | 0.717 |
| flunisolide | nitrofurantoin | 0.797 | 0.737 |
| ambroxol | pyrimethamine | 0.797 | 0.697 |
| fluphenazine | phenazopyridine | 0.797 | 0.736 |
| spectinomycin | sulfametoxydiazine | 0.797 | 0.709 |
| meropenem | nitrofurantoin | 0.797 | 0.732 |
| suxibuzone | terazosin | 0.797 | 0.711 |

| | | | |
|---|---|---|---|
| alprenolol | cefalexin | 0.797 | 0.711 |
| butoconazole | verteporfin | 0.797 | 0.798 |
| miconazole | terguride | 0.797 | 0.731 |
| norfloxacin | piroxicam | 0.797 | 0.673 |
| gliclazide | urapidil | 0.797 | 0.723 |
| piracetam | roxithromycin | 0.797 | 0.886 |
| dienestrol | disulfiram | 0.797 | 0.776 |
| budesonide | meticrane | 0.797 | 0.730 |
| naloxone | trioxysalen | 0.797 | 0.724 |
| clonidine | miconazole | 0.797 | 0.751 |
| cefalexin | remoxipride | 0.797 | 0.691 |
| buspirone | estriol | 0.797 | 0.774 |
| etofenamate | idoxuridine | 0.797 | 0.704 |
| levonorgestrel | piperidolate | 0.797 | 0.778 |
| calcium_pantothenate | dextromethorphan | 0.797 | 0.844 |
| calcium_pantothenate | daunorubicin | 0.797 | 0.772 |
| sulfamethoxypyridazine | tobramycin | 0.797 | 0.767 |
| fenoterol | terfenadine | 0.797 | 0.748 |
| terfenadine | trifluridine | 0.797 | 0.799 |
| alfuzosin | doxylamine | 0.797 | 0.735 |
| estrone | miconazole | 0.797 | 0.730 |
| clioquinol | trifluridine | 0.797 | 0.737 |
| flutamide | tiapride | 0.797 | 0.676 |
| dacarbazine | labetalol | 0.797 | 0.698 |
| oxetacaine | pentoxyverine | 0.797 | 0.724 |
| bupropion | quinidine | 0.797 | 0.658 |
| betahistine | ipratropium_bromide | 0.797 | 0.720 |
| hydrocortisone | sulfamethoxypyridazir | 0.797 | 0.735 |
| chlorprothixene | nicotinic_acid | 0.797 | 0.738 |
| bisacodyl | metamizole_sodium | 0.797 | 0.720 |
| clomipramine | papaverine | 0.797 | 0.724 |
| mitoxantrone | sulfaguanidine | 0.797 | 0.753 |
| prenylamine | thioridazine | 0.797 | 0.723 |
| minaprine | monobenzone | 0.798 | 0.668 |
| bromocriptine | fluvoxamine | 0.798 | 0.785 |
| clemastine | penbutolol | 0.798 | 0.693 |
| doxepin | idoxuridine | 0.798 | 0.724 |
| mepacrine | suloctidil | 0.798 | 0.750 |
| fenoprofen | propafenone | 0.798 | 0.704 |
| amikacin | pipemidic_acid | 0.798 | 0.784 |
| danazol | perphenazine | 0.798 | 0.730 |
| acetylsalicylic_acid | halcinonide | 0.798 | 0.791 |
| rifabutin | trazodone | 0.798 | 0.815 |
| pyrvinium | semustine | 0.798 | 0.806 |
| ioversol | propylthiouracil | 0.798 | 0.830 |
| felodipine | scopolamine | 0.798 | 0.717 |
| cefalexin | trioxysalen | 0.798 | 0.769 |
| clofazimine | proscillaridin | 0.798 | 0.807 |
| cinchocaine | latamoxef | 0.798 | 0.756 |
| imipramine | mianserin | 0.798 | 0.656 |

| | | | |
|---|---|---|---|
| acetazolamide | nifedipine | 0.798 | 0.717 |
| flumetasone | tobramycin | 0.798 | 0.700 |
| isradipine | sulconazole | 0.798 | 0.739 |
| fipexide | sulconazole | 0.798 | 0.733 |
| econazole | trimipramine | 0.798 | 0.655 |
| cyproterone | tropicamide | 0.798 | 0.732 |
| etomidate | hydrocortisone | 0.798 | 0.742 |
| cyclopenthiazide | tolazoline | 0.798 | 0.781 |
| prenylamine | profenamine | 0.798 | 0.700 |
| disulfiram | hydroquinine | 0.798 | 0.687 |
| apomorphine | triflusal | 0.798 | 0.691 |
| citiolone | talampicillin | 0.798 | 0.797 |
| oxetacaine | phenoxybenzamine | 0.798 | 0.769 |
| flunarizine | promazine | 0.798 | 0.750 |
| citiolone | piperidolate | 0.798 | 0.747 |
| cefalotin | terguride | 0.798 | 0.739 |
| cefalexin | norethisterone | 0.798 | 0.723 |
| gefitinib | thioridazine | 0.798 | 0.697 |
| doxorubicin | fludroxycortide | 0.798 | 0.715 |
| desipramine | proguanil | 0.798 | 0.725 |
| procaine | tobramycin | 0.798 | 0.790 |
| ivermectin | triamterene | 0.798 | 0.872 |
| apomorphine | etomidate | 0.798 | 0.726 |
| cisapride | talampicillin | 0.798 | 0.715 |
| azacitidine | flunisolide | 0.798 | 0.693 |
| doxazosin | oxamniquine | 0.798 | 0.710 |
| ethisterone | fluocinonide | 0.798 | 0.664 |
| acepromazine | trimetazidine | 0.798 | 0.693 |
| norethisterone | sulfafurazole | 0.798 | 0.766 |
| felodipine | oxaprozin | 0.798 | 0.726 |
| clomipramine | cortisone | 0.798 | 0.786 |
| ceftazidime | triflusal | 0.798 | 0.796 |
| buflomedil | norfloxacin | 0.798 | 0.650 |
| dipivefrine | methylergometrine | 0.798 | 0.720 |
| carbachol | prenylamine | 0.798 | 0.774 |
| aciclovir | topiramate | 0.798 | 0.730 |
| butoconazole | glibenclamide | 0.798 | 0.770 |
| emetine | irinotecan | 0.798 | 0.761 |
| syrosingopine | tetryzoline | 0.798 | 0.844 |
| clotrimazole | rescinnamine | 0.798 | 0.821 |
| astemizole | sirolimus | 0.798 | 0.813 |
| azacitidine | carbachol | 0.798 | 0.663 |
| clioquinol | disulfiram | 0.798 | 0.767 |
| bezafibrate | methapyrilene | 0.798 | 0.760 |
| amitriptyline | fluphenazine | 0.798 | 0.661 |
| desipramine | haloperidol | 0.798 | 0.788 |
| ronidazole | saquinavir | 0.798 | 0.811 |
| cyproheptadine | paclitaxel | 0.798 | 0.858 |
| cyproheptadine | monobenzone | 0.798 | 0.748 |
| dicoumarol | fenoterol | 0.798 | 0.673 |

| | | | |
|---|---|---|---|
| cortisone | doxazosin | 0.798 | 0.743 |
| cyproterone | metacycline | 0.798 | 0.708 |
| benfotiamine | piperidolate | 0.798 | 0.751 |
| sertaconazole | zomepirac | 0.798 | 0.701 |
| fluphenazine | rescinnamine | 0.798 | 0.746 |
| chlorzoxazone | medrysone | 0.798 | 0.787 |
| etodolac | meptazinol | 0.798 | 0.711 |
| cetirizine | glibenclamide | 0.798 | 0.735 |
| glibenclamide | natamycin | 0.798 | 0.785 |
| ethotoin | metamizole_sodium | 0.798 | 0.664 |
| chlorzoxazone | sulpiride | 0.798 | 0.762 |
| clindamycin | pivampicillin | 0.798 | 0.725 |
| minaprine | tolazoline | 0.798 | 0.735 |
| citiolone | repaglinide | 0.798 | 0.824 |
| nimodipine | prazosin | 0.798 | 0.749 |
| famotidine | loperamide | 0.798 | 0.760 |
| atovaquone | meticrane | 0.798 | 0.767 |
| lincomycin | lymecycline | 0.798 | 0.757 |
| cefalotin | lisinopril | 0.798 | 0.715 |
| disulfiram | dosulepin | 0.798 | 0.745 |
| nystatin | roxithromycin | 0.798 | 0.793 |
| oxybuprocaine | promazine | 0.798 | 0.734 |
| bromocriptine | miconazole | 0.798 | 0.760 |
| benzethonium_chloride | nicergoline | 0.798 | 0.736 |
| doxorubicin | prilocaine | 0.798 | 0.717 |
| imipenem | remoxipride | 0.798 | 0.744 |
| cetirizine | etomidate | 0.798 | 0.731 |
| clomifene | rifabutin | 0.798 | 0.822 |
| fenoprofen | sulfafurazole | 0.798 | 0.676 |
| diltiazem | metacycline | 0.798 | 0.728 |
| fenoprofen | primaquine | 0.798 | 0.670 |
| carbachol | flurbiprofen | 0.798 | 0.706 |
| piperidolate | zomepirac | 0.798 | 0.706 |
| ivermectin | levonorgestrel | 0.798 | 0.861 |
| capsaicin | etofenamate | 0.798 | 0.718 |
| dacarbazine | scopolamine | 0.798 | 0.740 |
| daunorubicin | sulfaphenazole | 0.798 | 0.711 |
| cinchocaine | sulfametoxydiazine | 0.798 | 0.707 |
| meclozine | ticlopidine | 0.798 | 0.654 |
| sulfametoxydiazine | ursodeoxycholic_acid | 0.798 | 0.763 |
| amantadine | iohexol | 0.798 | 0.867 |
| dacarbazine | dydrogesterone | 0.798 | 0.777 |
| dipyridamole | doxorubicin | 0.798 | 0.728 |
| omeprazole | propantheline_bromic | 0.798 | 0.687 |
| hydrocortisone | lymecycline | 0.798 | 0.759 |
| imipramine | rescinnamine | 0.798 | 0.797 |
| fendiline | syrosingopine | 0.798 | 0.811 |
| flufenamic_acid | fluocinonide | 0.798 | 0.774 |
| ivermectin | neomycin | 0.798 | 0.769 |
| bepridil | desipramine | 0.798 | 0.700 |

| | | | |
|---|---|---|---|
| altizide | doxazosin | 0.798 | 0.726 |
| dacarbazine | guanfacine | 0.798 | 0.682 |
| ketanserin | suloctidil | 0.798 | 0.708 |
| amitriptyline | benzonatate | 0.798 | 0.846 |
| amodiaquine | vinpocetine | 0.798 | 0.744 |
| nifurtimox | pyrazinamide | 0.798 | 0.707 |
| exemestane | piroxicam | 0.798 | 0.750 |
| bromperidol | chlorzoxazone | 0.798 | 0.755 |
| dilazep | irinotecan | 0.798 | 0.750 |
| amoxapine | rescinnamine | 0.798 | 0.797 |
| clomifene | ketoconazole | 0.798 | 0.758 |
| deptropine | levodopa | 0.798 | 0.777 |
| lanatoside_C | levomepromazine | 0.798 | 0.879 |
| meptazinol | nitrofurantoin | 0.798 | 0.739 |
| noretynodrel | protriptyline | 0.798 | 0.733 |
| ethambutol | liothyronine | 0.798 | 0.759 |
| isradipine | oxprenolol | 0.798 | 0.747 |
| moxonidine | talampicillin | 0.798 | 0.783 |
| fenoterol | sulfaphenazole | 0.798 | 0.688 |
| atovaquone | estriol | 0.798 | 0.719 |
| amoxapine | clioquinol | 0.798 | 0.697 |
| lidocaine | sulpiride | 0.798 | 0.713 |
| cefsulodin | salbutamol | 0.798 | 0.763 |
| clindamycin | phenoxybenzamine | 0.798 | 0.751 |
| atovaquone | carbachol | 0.798 | 0.764 |
| nomifensine | tyloxapol | 0.798 | 0.890 |
| citiolone | trioxysalen | 0.798 | 0.737 |
| gefitinib | phenoxybenzamine | 0.798 | 0.773 |
| bromopride | pargyline | 0.798 | 0.730 |
| carbachol | ursodeoxycholic_acid | 0.798 | 0.792 |
| enoxacin | mafenide | 0.798 | 0.679 |
| clomifene | etacrynic_acid | 0.798 | 0.746 |
| benperidol | monobenzone | 0.798 | 0.746 |
| ethisterone | phentolamine | 0.798 | 0.754 |
| phenindione | vidarabine | 0.798 | 0.662 |
| meticrane | nabumetone | 0.798 | 0.715 |
| benperidol | etofenamate | 0.798 | 0.726 |
| iopanoic_acid | procarbazine | 0.798 | 0.681 |
| daunorubicin | papaverine | 0.798 | 0.724 |
| famotidine | tolfenamic_acid | 0.798 | 0.739 |
| bromopride | trioxysalen | 0.798 | 0.716 |
| liothyronine | mepyramine | 0.798 | 0.711 |
| deptropine | methylergometrine | 0.798 | 0.726 |
| oxybutynin | suloctidil | 0.798 | 0.722 |
| phenazopyridine | piperidolate | 0.798 | 0.731 |
| lomefloxacin | reserpine | 0.798 | 0.734 |
| fenoprofen | levodopa | 0.798 | 0.673 |
| clomifene | fenoterol | 0.798 | 0.759 |
| isoconazole | triflusal | 0.798 | 0.769 |
| buflomedil | clonidine | 0.798 | 0.705 |

| | | | |
|---|---|---|---|
| diphenylpyraline | metergoline | 0.798 | 0.750 |
| astemizole | phentolamine | 0.798 | 0.737 |
| butoconazole | nitrendipine | 0.798 | 0.716 |
| chlorcyclizine | oxaprozin | 0.798 | 0.669 |
| bromperidol | demecolcine | 0.798 | 0.730 |
| ketanserin | sulfathiazole | 0.798 | 0.728 |
| etofenamate | triflusal | 0.798 | 0.740 |
| naftifine | spaglumic_acid | 0.798 | 0.803 |
| clotrimazole | danazol | 0.798 | 0.745 |
| oxamic_acid | trimetazidine | 0.798 | 0.834 |
| dipyridamole | etofylline | 0.798 | 0.708 |
| meclofenoxate | oxybuprocaine | 0.798 | 0.665 |
| metacycline | tiabendazole | 0.798 | 0.679 |
| meticrane | sulfamethoxazole | 0.798 | 0.681 |
| meticrane | sertaconazole | 0.798 | 0.776 |
| piribedil | trioxysalen | 0.798 | 0.772 |
| clotrimazole | demecolcine | 0.798 | 0.716 |
| amoxapine | midecamycin | 0.798 | 0.854 |
| carbachol | piromidic_acid | 0.798 | 0.743 |
| emetine | felodipine | 0.798 | 0.765 |
| salbutamol | tobramycin | 0.798 | 0.776 |
| bufexamac | dyclonine | 0.798 | 0.651 |
| atovaquone | glibenclamide | 0.798 | 0.727 |
| amodiaquine | phenoxybenzamine | 0.798 | 0.737 |
| citiolone | doxorubicin | 0.798 | 0.832 |
| ciclopirox | fendiline | 0.798 | 0.774 |
| clobetasol | syrosingopine | 0.798 | 0.770 |
| medrysone | piracetam | 0.798 | 0.798 |
| dipyridamole | pyrazinamide | 0.798 | 0.790 |
| alfaxalone | ursodeoxycholic_acid | 0.798 | 0.693 |
| fluspirilene | pimethixene | 0.798 | 0.777 |
| naloxone | thiocolchicoside | 0.798 | 0.767 |
| amantadine | mebhydrolin | 0.798 | 0.810 |
| cefixime | doxorubicin | 0.798 | 0.693 |
| estrone | trifluoperazine | 0.798 | 0.732 |
| sisomicin | suprofen | 0.798 | 0.766 |
| bepridil | clofazimine | 0.798 | 0.736 |
| dacarbazine | norfloxacin | 0.798 | 0.663 |
| protriptyline | tyloxapol | 0.798 | 0.868 |
| chlorcyclizine | mebendazole | 0.798 | 0.727 |
| apomorphine | remoxipride | 0.798 | 0.700 |
| perhexiline | zalcitabine | 0.798 | 0.781 |
| betahistine | levocabastine | 0.798 | 0.797 |
| alfaxalone | aminocaproic_acid | 0.798 | 0.784 |
| carbachol | ganciclovir | 0.798 | 0.706 |
| cortisone | ketanserin | 0.798 | 0.715 |
| pyrithyldione | ribostamycin | 0.798 | 0.838 |
| clonidine | oxytetracycline | 0.798 | 0.794 |
| citiolone | tobramycin | 0.798 | 0.839 |
| flufenamic_acid | methylprednisolone | 0.798 | 0.716 |

| | | | |
|---|---|---|---|
| minaprine | nifurtimox | 0.798 | 0.657 |
| bromopride | milrinone | 0.798 | 0.692 |
| liothyronine | sulfafurazole | 0.798 | 0.729 |
| promethazine | talampicillin | 0.798 | 0.789 |
| clemastine | mefloquine | 0.798 | 0.750 |
| labetalol | papaverine | 0.798 | 0.730 |
| noretynodrel | oxetacaine | 0.798 | 0.768 |
| clindamycin | triflusal | 0.798 | 0.749 |
| atovaquone | levamisole | 0.798 | 0.727 |
| amoxicillin | repaglinide | 0.798 | 0.721 |
| pentoxyverine | remoxipride | 0.798 | 0.682 |
| chlorambucil | flufenamic_acid | 0.798 | 0.698 |
| benzethonium_chloride | droperidol | 0.798 | 0.743 |
| clofazimine | podophyllotoxin | 0.798 | 0.761 |
| astemizole | dihydroergocristine | 0.798 | 0.733 |
| bisacodyl | isoconazole | 0.798 | 0.729 |
| aminocaproic_acid | cinchocaine | 0.798 | 0.790 |
| methazolamide | zomepirac | 0.798 | 0.678 |
| bromocriptine | mifepristone | 0.798 | 0.773 |
| phenoxybenzamine | pizotifen | 0.798 | 0.744 |
| liothyronine | trimetazidine | 0.798 | 0.720 |
| labetalol | oxybutynin | 0.798 | 0.738 |
| moxonidine | tyloxapol | 0.798 | 0.886 |
| cefalexin | flupentixol | 0.798 | 0.716 |
| profenamine | protriptyline | 0.798 | 0.686 |
| meclofenoxate | propylthiouracil | 0.798 | 0.722 |
| decitabine | estradiol | 0.798 | 0.714 |
| propofol | repaglinide | 0.798 | 0.809 |
| cinchocaine | ethosuximide | 0.798 | 0.810 |
| azacitidine | dacarbazine | 0.798 | 0.654 |
| chlorzoxazone | trifluridine | 0.798 | 0.733 |
| gliclazide | testosterone | 0.798 | 0.778 |
| estriol | reserpine | 0.798 | 0.755 |
| (-)-atenolol | metyrapone | 0.798 | 0.725 |
| meticrane | phenazopyridine | 0.798 | 0.722 |
| cefalexin | chlortetracycline | 0.798 | 0.683 |
| estriol | ketoprofen | 0.798 | 0.657 |
| benfotiamine | chlorcyclizine | 0.798 | 0.753 |
| lomefloxacin | sulfamethoxazole | 0.798 | 0.709 |
| deptropine | etamsylate | 0.798 | 0.801 |
| iopanoic_acid | ranitidine | 0.798 | 0.755 |
| liothyronine | theophylline | 0.798 | 0.800 |
| aminohippuric_acid | metacycline | 0.798 | 0.687 |
| acepromazine | cyproterone | 0.798 | 0.713 |
| aciclovir | clemastine | 0.798 | 0.785 |
| ethambutol | mycophenolic_acid | 0.798 | 0.723 |
| cyproterone | sulfafurazole | 0.798 | 0.755 |
| ganciclovir | roxithromycin | 0.798 | 0.847 |
| omeprazole | procyclidine | 0.798 | 0.731 |
| mebendazole | propafenone | 0.798 | 0.708 |

| | | | |
|---|---|---|---|
| bisacodyl | fenoprofen | 0.798 | 0.720 |
| bromopride | fluorometholone | 0.798 | 0.743 |
| labetalol | medrysone | 0.798 | 0.732 |
| sulconazole | suloctidil | 0.798 | 0.758 |
| dinoprost | lanatoside_C | 0.798 | 0.836 |
| levonorgestrel | miconazole | 0.798 | 0.740 |
| deptropine | methapyrilene | 0.798 | 0.701 |
| disulfiram | fluspirilene | 0.798 | 0.729 |
| clofazimine | fendiline | 0.799 | 0.710 |
| amiodarone | clioquinol | 0.799 | 0.709 |
| atovaquone | lansoprazole | 0.799 | 0.668 |
| chlorzoxazone | mifepristone | 0.799 | 0.818 |
| meclozine | nitrofurantoin | 0.799 | 0.794 |
| bezafibrate | pentetrazol | 0.799 | 0.784 |
| acebutolol | lidocaine | 0.799 | 0.744 |
| spectinomycin | tyloxapol | 0.799 | 0.867 |
| acepromazine | propofol | 0.799 | 0.749 |
| fenoprofen | rifabutin | 0.799 | 0.834 |
| chlorprothixene | rifabutin | 0.799 | 0.828 |
| deferoxamine | rimexolone | 0.799 | 0.832 |
| azacitidine | mefloquine | 0.799 | 0.693 |
| prochlorperazine | ritodrine | 0.799 | 0.729 |
| felodipine | flunarizine | 0.799 | 0.753 |
| iopanoic_acid | ioxaglic_acid | 0.799 | 0.787 |
| norethisterone | promazine | 0.799 | 0.756 |
| doxorubicin | phenoxybenzamine | 0.799 | 0.761 |
| meptazinol | pargyline | 0.799 | 0.697 |
| flunarizine | ursodeoxycholic_acid | 0.799 | 0.745 |
| cyproheptadine | primaquine | 0.799 | 0.699 |
| galantamine | milrinone | 0.799 | 0.713 |
| propylthiouracil | sulfaphenazole | 0.799 | 0.703 |
| reserpine | talampicillin | 0.799 | 0.720 |
| irinotecan | oleandomycin | 0.799 | 0.804 |
| flufenamic_acid | paracetamol | 0.799 | 0.692 |
| omeprazole | oxamniquine | 0.799 | 0.689 |
| deptropine | mefloquine | 0.799 | 0.757 |
| dihydroergocristine | levomepromazine | 0.799 | 0.781 |
| clioquinol | fendiline | 0.799 | 0.767 |
| clonidine | methylprednisolone | 0.799 | 0.797 |
| fluvastatin | hydralazine | 0.799 | 0.751 |
| phenazone | pipemidic_acid | 0.799 | 0.663 |
| felodipine | fenbendazole | 0.799 | 0.740 |
| iohexol | nilutamide | 0.799 | 0.768 |
| nalidixic_acid | oxetacaine | 0.799 | 0.780 |
| etamsylate | moxonidine | 0.799 | 0.675 |
| enalapril | glafenine | 0.799 | 0.692 |
| betahistine | fludroxycortide | 0.799 | 0.822 |
| levocabastine | mesoridazine | 0.799 | 0.764 |
| acetohexamide | diltiazem | 0.799 | 0.722 |
| calcium_pantothenate | trioxysalen | 0.799 | 0.835 |

| | | | |
|---|---|---|---|
| fluoxetine | thioproperazine | 0.799 | 0.701 |
| cimetidine | ticlopidine | 0.799 | 0.729 |
| sodium_phenylbutyrate | vorinostat | 0.799 | 0.650 |
| etofylline | tropicamide | 0.799 | 0.716 |
| ifenprodil | promazine | 0.799 | 0.766 |
| meptazinol | sulfaphenazole | 0.799 | 0.721 |
| budesonide | estriol | 0.799 | 0.703 |
| betahistine | repaglinide | 0.799 | 0.805 |
| astemizole | bufexamac | 0.799 | 0.773 |
| meclocycline | oxolamine | 0.799 | 0.693 |
| dihydroergocristine | prochlorperazine | 0.799 | 0.745 |
| cinchocaine | promazine | 0.799 | 0.671 |
| dipyridamole | remoxipride | 0.799 | 0.760 |
| erastin | maprotiline | 0.799 | 0.781 |
| capsaicin | cinchocaine | 0.799 | 0.718 |
| selegiline | triflusal | 0.799 | 0.782 |
| benperidol | cinchocaine | 0.799 | 0.719 |
| cefalexin | oxybutynin | 0.799 | 0.706 |
| estriol | ifenprodil | 0.799 | 0.716 |
| sulfafurazole | talampicillin | 0.799 | 0.752 |
| norfloxacin | sulfaguanidine | 0.799 | 0.697 |
| acepromazine | bromperidol | 0.799 | 0.760 |
| mesoridazine | methazolamide | 0.799 | 0.723 |
| cetirizine | menadione | 0.799 | 0.815 |
| dextromethorphan | hydrocortisone | 0.799 | 0.779 |
| mepyramine | talampicillin | 0.799 | 0.771 |
| levomepromazine | lisuride | 0.799 | 0.704 |
| piribedil | rolitetracycline | 0.799 | 0.745 |
| midecamycin | naloxone | 0.799 | 0.833 |
| hydrocortisone | terguride | 0.799 | 0.755 |
| oxolinic_acid | repaglinide | 0.799 | 0.747 |
| mitoxantrone | sulfafurazole | 0.799 | 0.748 |
| idoxuridine | ivermectin | 0.799 | 0.861 |
| betaxolol | proxyphylline | 0.799 | 0.689 |
| benzethonium_chloride | clomifene | 0.799 | 0.654 |
| decitabine | diazoxide | 0.799 | 0.687 |
| bepridil | procaine | 0.799 | 0.730 |
| benzethonium_chloride | papaverine | 0.799 | 0.756 |
| butoconazole | flupentixol | 0.799 | 0.735 |
| capsaicin | pivampicillin | 0.799 | 0.751 |
| methazolamide | sulpiride | 0.799 | 0.650 |
| bromperidol | talampicillin | 0.799 | 0.691 |
| pivampicillin | triflusal | 0.799 | 0.778 |
| atovaquone | cetirizine | 0.799 | 0.741 |
| betahistine | flunarizine | 0.799 | 0.787 |
| doxorubicin | nialamide | 0.799 | 0.714 |
| papaverine | protriptyline | 0.799 | 0.703 |
| galantamine | oxamniquine | 0.799 | 0.737 |
| amoxapine | hydroxyzine | 0.799 | 0.704 |
| betaxolol | norethisterone | 0.799 | 0.766 |

| | | | |
|---|---|---|---|
| cinchocaine | ifenprodil | 0.799 | 0.746 |
| bromopride | fipexide | 0.799 | 0.729 |
| cefoperazone | dinoprost | 0.799 | 0.737 |
| decitabine | mifepristone | 0.799 | 0.810 |
| chlorzoxazone | vinpocetine | 0.799 | 0.747 |
| dipivefrine | oxybutynin | 0.799 | 0.705 |
| hydralazine | propafenone | 0.799 | 0.697 |
| pentetrazol | sulfaphenazole | 0.799 | 0.758 |
| pipemidic_acid | sulconazole | 0.799 | 0.742 |
| hydralazine | sulfadimidine | 0.799 | 0.689 |
| dirithromycin | trimetazidine | 0.799 | 0.848 |
| fluspirilene | noretynodrel | 0.799 | 0.704 |
| liothyronine | oxprenolol | 0.799 | 0.734 |
| chlorcyclizine | syrosingopine | 0.799 | 0.821 |
| doxazosin | pyrantel | 0.799 | 0.713 |
| econazole | phenazopyridine | 0.799 | 0.712 |
| dipyridamole | fenspiride | 0.799 | 0.784 |
| ethambutol | niclosamide | 0.799 | 0.739 |
| bemegride | promazine | 0.799 | 0.786 |
| betaxolol | butoconazole | 0.799 | 0.754 |
| dipyridamole | scopolamine | 0.799 | 0.778 |
| apomorphine | trimetazidine | 0.799 | 0.704 |
| (-)-atenolol | aminohippuric_acid | 0.799 | 0.660 |
| etofenamate | saquinavir | 0.799 | 0.782 |
| betaxolol | talampicillin | 0.799 | 0.750 |
| doxorubicin | fenbufen | 0.799 | 0.699 |
| doxazosin | zimeldine | 0.799 | 0.756 |
| methotrexate | verapamil | 0.799 | 0.728 |
| chlortetracycline | demecolcine | 0.799 | 0.759 |
| phenazopyridine | sulfinpyrazone | 0.799 | 0.716 |
| azacitidine | flufenamic_acid | 0.799 | 0.673 |
| fluocinonide | pyrazinamide | 0.799 | 0.826 |
| colchicine | vorinostat | 0.799 | 0.751 |
| mecamylamine | topiramate | 0.799 | 0.813 |
| isoetarine | topiramate | 0.799 | 0.734 |
| fluorometholone | pyrvinium | 0.799 | 0.806 |
| bromopride | propafenone | 0.799 | 0.689 |
| betaxolol | cyproterone | 0.799 | 0.730 |
| bupropion | oxytetracycline | 0.799 | 0.740 |
| apomorphine | sulfamethoxypyridazir | 0.799 | 0.720 |
| cefazolin | tiapride | 0.799 | 0.747 |
| bromperidol | metamizole_sodium | 0.799 | 0.724 |
| bepridil | raloxifene | 0.799 | 0.754 |
| benfotiamine | pyrazinamide | 0.799 | 0.779 |
| phenformin | propofol | 0.799 | 0.733 |
| gliclazide | oxymetazoline | 0.799 | 0.745 |
| procainamide | talampicillin | 0.799 | 0.758 |
| ioversol | sulfamethoxypyridazir | 0.799 | 0.776 |
| mephentermine | ofloxacin | 0.799 | 0.751 |
| natamycin | remoxipride | 0.799 | 0.816 |

| | | | |
|---|---|---|---|
| metergoline | podophyllotoxin | 0.799 | 0.763 |
| flumequine | levamisole | 0.799 | 0.697 |
| chlorpropamide | ketanserin | 0.799 | 0.704 |
| fendiline | raloxifene | 0.799 | 0.759 |
| etacrynic_acid | imipramine | 0.799 | 0.730 |
| cefalexin | vinpocetine | 0.799 | 0.756 |
| letrozole | memantine | 0.799 | 0.779 |
| flunarizine | vidarabine | 0.799 | 0.783 |
| cefalotin | clobetasol | 0.799 | 0.726 |
| altretamine | pralidoxime | 0.799 | 0.699 |
| fipexide | procaine | 0.799 | 0.706 |
| acetylsalicylic_acid | azacitidine | 0.799 | 0.664 |
| ifenprodil | metergoline | 0.799 | 0.720 |
| bisacodyl | trazodone | 0.799 | 0.749 |
| cyproterone | phenoxybenzamine | 0.799 | 0.748 |
| gliclazide | tioguanine | 0.799 | 0.781 |
| carbimazole | nadolol | 0.799 | 0.736 |
| (-)-atenolol | trioxysalen | 0.799 | 0.707 |
| labetalol | oxytetracycline | 0.799 | 0.734 |
| fenbendazole | fendiline | 0.799 | 0.731 |
| ketanserin | miconazole | 0.799 | 0.757 |
| betaxolol | dirithromycin | 0.799 | 0.832 |
| desoxycortone | meclozine | 0.799 | 0.781 |
| betahistine | metacycline | 0.799 | 0.731 |
| captopril | ketanserin | 0.799 | 0.771 |
| chlorcyclizine | flupentixol | 0.799 | 0.724 |
| ioversol | liothyronine | 0.799 | 0.755 |
| etacrynic_acid | fipexide | 0.799 | 0.718 |
| dacarbazine | piromidic_acid | 0.799 | 0.669 |
| trifluridine | vorinostat | 0.799 | 0.730 |
| spectinomycin | sulfafurazole | 0.799 | 0.726 |
| imipramine | lanatoside_C | 0.799 | 0.891 |
| isoconazole | reserpine | 0.799 | 0.772 |
| doxazosin | hydralazine | 0.799 | 0.770 |
| metamizole_sodium | reserpine | 0.799 | 0.742 |
| doxazosin | testosterone | 0.799 | 0.767 |
| piribedil | propantheline_bromic | 0.799 | 0.711 |
| cefsulodin | spectinomycin | 0.799 | 0.770 |
| glafenine | tribenoside | 0.799 | 0.715 |
| iohexol | succinylsulfathiazole | 0.799 | 0.756 |
| liothyronine | nalbuphine | 0.799 | 0.751 |
| chloramphenicol | proxyphylline | 0.799 | 0.676 |
| doxorubicin | oxaprozin | 0.799 | 0.728 |
| capsaicin | chloramphenicol | 0.799 | 0.741 |
| atovaquone | sulfaphenazole | 0.799 | 0.691 |
| levonorgestrel | methylergometrine | 0.799 | 0.723 |
| clobetasol | verteporfin | 0.799 | 0.792 |
| atovaquone | colistin | 0.799 | 0.874 |
| felodipine | levamisole | 0.799 | 0.748 |
| cetirizine | clonidine | 0.799 | 0.793 |

| | | | |
|---|---|---|---|
| clindamycin | torasemide | 0.799 | 0.715 |
| benperidol | triflusal | 0.799 | 0.785 |
| piribedil | verteporfin | 0.799 | 0.803 |
| mepacrine | terbutaline | 0.799 | 0.743 |
| piromidic_acid | repaglinide | 0.799 | 0.715 |
| dextromethorphan | suloctidil | 0.799 | 0.768 |
| phenoxybenzamine | troglitazone | 0.799 | 0.756 |
| riluzole | theophylline | 0.799 | 0.661 |
| clonidine | estriol | 0.799 | 0.771 |
| lisinopril | triprolidine | 0.799 | 0.766 |
| gefitinib | tamoxifen | 0.799 | 0.762 |
| flutamide | hydralazine | 0.799 | 0.696 |
| acetylsalicylic_acid | procaine | 0.799 | 0.683 |
| clonidine | omeprazole | 0.799 | 0.753 |
| dipyridamole | trifluridine | 0.799 | 0.744 |
| debrisoquine | phenylpropanolamine | 0.799 | 0.672 |
| gliclazide | mebendazole | 0.799 | 0.663 |
| exemestane | famotidine | 0.799 | 0.775 |
| bepridil | sulfamethoxypyridazir | 0.799 | 0.754 |
| iloprost | naltrexone | 0.799 | 0.756 |
| calcium_pantothenate | ipratropium_bromide | 0.799 | 0.779 |
| fenoprofen | meglumine | 0.799 | 0.726 |
| semustine | suloctidil | 0.799 | 0.734 |
| etidronic_acid | piracetam | 0.799 | 0.682 |
| cefixime | ronidazole | 0.799 | 0.747 |
| aminoglutethimide | pimethixene | 0.799 | 0.766 |
| etofenamate | lymecycline | 0.799 | 0.752 |
| altizide | cinchocaine | 0.799 | 0.723 |
| nitrofurantoin | propofol | 0.799 | 0.755 |
| domperidone | procainamide | 0.799 | 0.690 |
| etoposide | verapamil | 0.799 | 0.757 |
| buflomedil | desoxycortone | 0.799 | 0.739 |
| flunarizine | ketoprofen | 0.799 | 0.764 |
| etofenamate | hydrocortisone | 0.799 | 0.736 |
| ioversol | zomepirac | 0.799 | 0.775 |
| levamisole | sulfinpyrazone | 0.799 | 0.737 |
| metaraminol | risperidone | 0.799 | 0.797 |
| doxazosin | sotalol | 0.799 | 0.739 |
| metoclopramide | tropicamide | 0.799 | 0.704 |
| imipramine | triamterene | 0.799 | 0.712 |
| roxithromycin | ursodeoxycholic_acid | 0.799 | 0.802 |
| clobetasol | fluspirilene | 0.799 | 0.709 |
| bisacodyl | ronidazole | 0.799 | 0.745 |
| roxithromycin | succinylsulfathiazole | 0.799 | 0.819 |
| cyclobenzaprine | sertaconazole | 0.799 | 0.706 |
| aminoglutethimide | trazodone | 0.799 | 0.768 |
| betahistine | proscillaridin | 0.799 | 0.836 |
| dobutamine | vigabatrin | 0.799 | 0.786 |
| benfluorex | dextromethorphan | 0.799 | 0.760 |
| amoxicillin | cetirizine | 0.799 | 0.747 |

| | | | |
|---|---|---|---|
| iopamidol | isopropamide_iodide | 0.799 | 0.798 |
| benzethonium_chloride | fluorometholone | 0.799 | 0.809 |
| bisacodyl | dextromethorphan | 0.799 | 0.749 |
| acetylsalicylic_acid | latamoxef | 0.799 | 0.810 |
| fenbendazole | thioproperazine | 0.799 | 0.731 |
| dorzolamide | verteporfin | 0.799 | 0.821 |
| procarbazine | tribenoside | 0.799 | 0.787 |
| acetohexamide | atovaquone | 0.799 | 0.680 |
| daunorubicin | trazodone | 0.799 | 0.726 |
| progesterone | talampicillin | 0.799 | 0.738 |
| liothyronine | piperidolate | 0.799 | 0.742 |
| benperidol | oxybutynin | 0.799 | 0.749 |
| milrinone | urapidil | 0.799 | 0.714 |
| flavoxate | ifenprodil | 0.799 | 0.707 |
| doxorubicin | flavoxate | 0.799 | 0.659 |
| isoniazid | trihexyphenidyl | 0.799 | 0.741 |
| dydrogesterone | oxetacaine | 0.799 | 0.771 |
| chlorprothixene | phenazopyridine | 0.799 | 0.669 |
| cycloserine | fipexide | 0.799 | 0.851 |
| ipratropium_bromide | picotamide | 0.799 | 0.767 |
| demecolcine | levodopa | 0.799 | 0.724 |
| metyrapone | procyclidine | 0.799 | 0.676 |
| lisinopril | pimethixene | 0.799 | 0.790 |
| benfotiamine | capsaicin | 0.799 | 0.734 |
| (-)-catechin | roxithromycin | 0.799 | 0.848 |
| pimozide | ticlopidine | 0.799 | 0.778 |
| cyclopentolate | dienestrol | 0.799 | 0.775 |
| imipenem | nicotinic_acid | 0.799 | 0.726 |
| meptazinol | pyrvinium | 0.799 | 0.805 |
| fluvastatin | propofol | 0.799 | 0.756 |
| naftifine | risperidone | 0.799 | 0.717 |
| nafcillin | spectinomycin | 0.799 | 0.719 |
| cefalexin | sulfaguanidine | 0.799 | 0.701 |
| bupropion | fipexide | 0.799 | 0.707 |
| cetirizine | fludroxycortide | 0.799 | 0.762 |
| deferoxamine | tyloxapol | 0.799 | 0.844 |
| colecalciferol | triflusal | 0.799 | 0.813 |
| etacrynic_acid | rescinnamine | 0.799 | 0.786 |
| levocabastine | naftifine | 0.799 | 0.747 |
| cinnarizine | fipexide | 0.800 | 0.712 |
| gliclazide | guanfacine | 0.800 | 0.695 |
| azapropazone | rilmenidine | 0.800 | 0.740 |
| liothyronine | protriptyline | 0.800 | 0.749 |
| benzydamine | streptomycin | 0.800 | 0.804 |
| cefsulodin | sulconazole | 0.800 | 0.795 |
| propylthiouracil | simvastatin | 0.800 | 0.760 |
| nilutamide | xylometazoline | 0.800 | 0.724 |
| amitriptyline | clotrimazole | 0.800 | 0.665 |
| niridazole | talampicillin | 0.800 | 0.776 |
| methylergometrine | oxytetracycline | 0.800 | 0.689 |

| | | | |
|---|---|---|---|
| remoxipride | tropicamide | 0.800 | 0.728 |
| alfuzosin | meclozine | 0.800 | 0.744 |
| estriol | etidronic_acid | 0.800 | 0.761 |
| cinoxacin | tobramycin | 0.800 | 0.777 |
| pizotifen | prochlorperazine | 0.800 | 0.721 |
| procaine | verteporfin | 0.800 | 0.816 |
| haloperidol | proscillaridin | 0.800 | 0.738 |
| clonidine | metoprolol | 0.800 | 0.729 |
| benfluorex | econazole | 0.800 | 0.747 |
| ciclopirox | rescinnamine | 0.800 | 0.810 |
| isoconazole | levonorgestrel | 0.800 | 0.706 |
| benzonatate | procaine | 0.800 | 0.731 |
| pyrantel | ursodeoxycholic_acid | 0.800 | 0.804 |
| cyclobenzaprine | mefloquine | 0.800 | 0.745 |
| mesoridazine | verteporfin | 0.800 | 0.737 |
| cyproheptadine | propafenone | 0.800 | 0.740 |
| oxymetazoline | zimeldine | 0.800 | 0.714 |
| methazolamide | trimetazidine | 0.800 | 0.681 |
| meglumine | metyrapone | 0.800 | 0.712 |
| menadione | urapidil | 0.800 | 0.707 |
| levobunolol | nafcillin | 0.800 | 0.679 |
| pipemidic_acid | tropicamide | 0.800 | 0.721 |
| fendiline | ketotifen | 0.800 | 0.767 |
| cefotetan | chloramphenicol | 0.800 | 0.772 |
| medrysone | phenazopyridine | 0.800 | 0.739 |
| ketanserin | pivampicillin | 0.800 | 0.709 |
| cefalexin | sulfathiazole | 0.800 | 0.702 |
| daunorubicin | desoxycortone | 0.800 | 0.746 |
| capsaicin | etamsylate | 0.800 | 0.772 |
| demecolcine | lanatoside_C | 0.800 | 0.863 |
| propafenone | tioguanine | 0.800 | 0.774 |
| methazolamide | sulfamethoxypyridazir | 0.800 | 0.658 |
| clioquinol | syrosingopine | 0.800 | 0.833 |
| acetylsalicylic_acid | clioquinol | 0.800 | 0.654 |
| bufexamac | nitrofurantoin | 0.800 | 0.685 |
| riluzole | tobramycin | 0.800 | 0.806 |
| niflumic_acid | norethisterone | 0.800 | 0.735 |
| clomipramine | natamycin | 0.800 | 0.829 |
| bemegride | pentoxyverine | 0.800 | 0.757 |
| deptropine | naloxone | 0.800 | 0.726 |
| chlorzoxazone | dydrogesterone | 0.800 | 0.794 |
| bromocriptine | dydrogesterone | 0.800 | 0.768 |
| cyproterone | thiocolchicoside | 0.800 | 0.723 |
| bufexamac | dipyridamole | 0.800 | 0.758 |
| pyrimethamine | rifabutin | 0.800 | 0.841 |
| clotrimazole | mebendazole | 0.800 | 0.779 |
| chlorprothixene | oxantel | 0.800 | 0.669 |
| benperidol | medrysone | 0.800 | 0.715 |
| amiodarone | nilutamide | 0.800 | 0.739 |
| flunisolide | hyoscyamine | 0.800 | 0.730 |

| | | | |
|---|---|---|---|
| labetalol | loperamide | 0.800 | 0.704 |
| bromperidol | latamoxef | 0.800 | 0.713 |
| naloxone | piromidic_acid | 0.800 | 0.730 |
| perphenazine | tioguanine | 0.800 | 0.780 |
| altizide | rimexolone | 0.800 | 0.732 |
| paroxetine | sulconazole | 0.800 | 0.738 |
| molindone | pyrazinamide | 0.800 | 0.710 |
| cyclopentolate | verteporfin | 0.800 | 0.819 |
| propofol | reserpine | 0.800 | 0.827 |
| famotidine | methotrexate | 0.800 | 0.714 |
| oxprenolol | zomepirac | 0.800 | 0.706 |
| milrinone | sulfaphenazole | 0.800 | 0.712 |
| bezafibrate | fenoprofen | 0.800 | 0.695 |
| alfaxalone | doxorubicin | 0.800 | 0.754 |
| metyrapone | zomepirac | 0.800 | 0.678 |
| loxapine | terfenadine | 0.800 | 0.699 |
| gliclazide | lisinopril | 0.800 | 0.714 |
| galantamine | metaraminol | 0.800 | 0.733 |
| metrifonate | noretynodrel | 0.800 | 0.765 |
| levamisole | salbutamol | 0.800 | 0.666 |
| dipyridamole | omeprazole | 0.800 | 0.746 |
| butoconazole | ciclosporin | 0.800 | 0.880 |
| ifenprodil | scopolamine | 0.800 | 0.715 |
| citalopram | nialamide | 0.800 | 0.734 |
| phenoxybenzamine | trazodone | 0.800 | 0.722 |
| probenecid | selegiline | 0.800 | 0.727 |
| promazine | rifabutin | 0.800 | 0.835 |
| glycopyrronium_bromide | mephentermine | 0.800 | 0.715 |
| meticrane | prilocaine | 0.800 | 0.707 |
| protriptyline | rimexolone | 0.800 | 0.784 |
| cefixime | griseofulvin | 0.800 | 0.720 |
| betaxolol | levonorgestrel | 0.800 | 0.746 |
| astemizole | fluorometholone | 0.800 | 0.776 |
| labetalol | meticrane | 0.800 | 0.712 |
| amoxapine | prochlorperazine | 0.800 | 0.703 |
| ipratropium_bromide | sulfamerazine | 0.800 | 0.679 |
| dihydroergotamine | thioproperazine | 0.800 | 0.745 |
| aminohippuric_acid | isradipine | 0.800 | 0.741 |
| fluocinonide | meticrane | 0.800 | 0.772 |
| ambroxol | clindamycin | 0.800 | 0.740 |
| ajmaline | clemastine | 0.800 | 0.727 |
| mephenesin | ofloxacin | 0.800 | 0.663 |
| levonorgestrel | repaglinide | 0.800 | 0.738 |
| felodipine | nicotinic_acid | 0.800 | 0.756 |
| promethazine | trimetazidine | 0.800 | 0.691 |
| cefalotin | meptazinol | 0.800 | 0.791 |
| flunarizine | imipramine | 0.800 | 0.771 |
| ofloxacin | phenazone | 0.800 | 0.700 |
| ioversol | milrinone | 0.800 | 0.812 |
| cyproterone | pyrantel | 0.800 | 0.784 |

| | | | |
|---|---|---|---|
| alimemazine | deptropine | 0.800 | 0.675 |
| aminohippuric_acid | tobramycin | 0.800 | 0.800 |
| amiloride | piribedil | 0.800 | 0.671 |
| iopamidol | ribostamycin | 0.800 | 0.709 |
| dihydroergotamine | ivermectin | 0.800 | 0.782 |
| cefalotin | pentetrazol | 0.800 | 0.778 |
| calcium_pantothenate | methazolamide | 0.800 | 0.802 |
| ampicillin | baclofen | 0.800 | 0.718 |
| fluvastatin | roxithromycin | 0.800 | 0.784 |
| acepromazine | pyrazinamide | 0.800 | 0.722 |
| maprotiline | reserpine | 0.800 | 0.795 |
| gliclazide | midecamycin | 0.800 | 0.831 |
| propofol | urapidil | 0.800 | 0.781 |
| altizide | torasemide | 0.800 | 0.708 |
| cinoxacin | ioversol | 0.800 | 0.775 |
| chloramphenicol | salbutamol | 0.800 | 0.691 |
| nitrofurantoin | spectinomycin | 0.800 | 0.759 |
| proscillaridin | sulfametoxydiazine | 0.800 | 0.787 |
| hydralazine | metyrapone | 0.800 | 0.680 |
| ursodeoxycholic_acid | verteporfin | 0.800 | 0.768 |
| aminohippuric_acid | cefalexin | 0.800 | 0.706 |
| heptaminol | topiramate | 0.800 | 0.739 |
| pimozide | tribenoside | 0.800 | 0.758 |
| capsaicin | sulfafurazole | 0.800 | 0.709 |
| monobenzone | tyloxapol | 0.800 | 0.901 |
| azacitidine | promethazine | 0.800 | 0.721 |
| benperidol | miconazole | 0.800 | 0.736 |
| benzylpenicillin | trimetazidine | 0.800 | 0.739 |
| phenoxybenzamine | sertaconazole | 0.800 | 0.732 |
| dextromethorphan | metolazone | 0.800 | 0.759 |
| metacycline | talampicillin | 0.800 | 0.700 |
| cinchocaine | ronidazole | 0.800 | 0.680 |
| oxprenolol | propofol | 0.800 | 0.725 |
| octopamine | theophylline | 0.800 | 0.656 |
| fenofibrate | prednisolone | 0.800 | 0.737 |
| cyanocobalamin | ramipril | 0.800 | 0.854 |
| pyrazinamide | triflusal | 0.800 | 0.651 |
| pentoxyverine | propylthiouracil | 0.800 | 0.764 |
| cyproheptadine | mometasone | 0.800 | 0.790 |
