# Supplementary material for: Comparing structural and transcriptional drug networks reveals signatures of drug activity and toxicity in transcriptional responses
Source: NPJ Syst Biol Appl. 2017 Aug 25;3:23. doi: 10.1038/s41540-017-0022-3 (PMC5572457; doi:10.1038/s41540-017-0022-3)
Supplement: Supplementary file 7 — Supplementary Table 5 [file 41540_2017_22_MOESM7_ESM.pdf]

Names	LogP	pKa	TV
astemizole	5.39	8.75	0.48
suloctidil	5.61	9.76	0.57
perhexiline	5.53	10.58	0.61
pimozide	5.83	8.38	0.66
fendiline	5.83	10.07	0.67
thioridazine	5.47	8.93	0.70
desipramine	3.9	10.02	0.74
perphenazine	3.69	8.21	0.74
tomatidine	5.02	9.54	0.75
dilazep	2.88	9.54	0.76
clomipramine	4.88	9.2	0.76
trimipramine	4.76	9.42	0.77
amiodarone	7.64	8.47	0.77
nortriptyline	4.43	10.47	0.77
bromperidol	3.83	8.07	0.78
trifluoperazine	4.66	8.39	0.78
triflupromazine	4.81	9.2	0.80
fluphenazine	3.97	8.21	0.80
promethazine	4.29	9.05	0.82
loperamide	4.77	9.41	0.82
chlorprothixene	5.07	9.76	0.84
imipramine	4.28	9.2	0.85
maprotiline	4.37	10.54	0.85
fluoxetine	4.17	9.8	0.88
tacrine	2.63	8.95	0.88
fluvoxamine	2.8	9.16	0.89
clemastine	4.92	9.55	0.89
amodiaquine	3.76	10.23	0.90
chlorpromazine	4.54	9.2	0.90
chloroquine	3.93	10.32	0.91
raloxifene	5.69	7.95	0.91
paroxetine	3.15	9.77	0.91
tamoxifen	6.35	8.76	0.92
haloperidol	3.66	8.05	0.95
amitriptyline	4.81	9.76	0.95
cyclobenzaprine	4.61	9.76	1.10
