# Supplementary material for: Comparing structural and transcriptional drug networks reveals signatures of drug activity and toxicity in transcriptional responses
Source: NPJ Syst Biol Appl. 2017 Aug 25;3:23. doi: 10.1038/s41540-017-0022-3 (PMC5572457; doi:10.1038/s41540-017-0022-3)
Supplement: Supplementary file 8 — Supplementary Table 6 [file 41540_2017_22_MOESM8_ESM.pdf]

ID	Set name	Set descr	ES	p
GO:00057	lysosome	A small lyt	0.583246	9.05E-11
GO:00004	pre-autophagosomal structure	A punctate	0.553963	9.32E-10
GO:00314	cytoplasmic vesicle	A vesicle f	0.500415	4.85E-08
GO:00057	mitochondrial matrix	The gel-lik	-0.48305	1.61E-07
GO:00057	nucleolus	A small, de	-0.47905	2.1E-07
GO:00425	MCM complex	A hexame	-0.46963	3.93E-07
GO:00426	mitochondrial nucleoid	The regior	-0.46434	5.55E-07
GO:00057	smooth endoplasmic reticulum	The smoot	0.452627	1.18E-06
GO:00100	endosome membrane	The lipid b	0.444772	1.93E-06
GO:00004	autophagic vacuole membrane	The lipid b	0.44056	2.5E-06
GO:00001	Golgi membrane	The lipid b	0.439775	2.63E-06
GO:00301	COPII vesicle coat	One of twc	0.438771	2.8E-06
GO:00056	nuclear pore	Any of the	-0.43613	3.29E-06
GO:00056	spliceosomal complex	Any of a se	-0.42814	5.33E-06
GO:00432	Fanconi anaemia nuclear complex	A protein c	-0.42799	5.38E-06
GO:00301	ER to Golgi transport vesicle	A vesicle t	0.427861	5.42E-06
GO:00057	mitochondrial intermembrane space	The regior	-0.42199	7.69E-06
GO:00057	endosome	A membra	0.411146	1.45E-05
GO:00484	perinuclear region of cytoplasm	Cytoplasm	0.407502	1.78E-05
GO:00166	nuclear speck	A discrete	-0.40707	1.83E-05
GO:00057	mitochondrial inner membrane	The inner,	-0.40621	1.92E-05
GO:00057	endoplasmic reticulum membrane	The lipid b	0.403945	2.18E-05
GO:00057	lysosomal membrane	The lipid b	0.402221	2.41E-05
GO:00057	autophagic vacuole	A double-r	0.401872	2.45E-05
GO:00340	pre-autophagosomal structure membrane	A cellular r	0.399014	2.88E-05
GO:00001	ubiquitin ligase complex	A protein c	-0.3972	3.19E-05
GO:00163	basolateral plasma membrane	The regior	0.395719	3.46E-05
GO:00021	zona pellucida receptor complex	A multisub	-0.395	3.6E-05
GO:00058	mRNA cap binding complex	Any protei	-0.39179	4.3E-05
GO:00325	Golgi cisterna membrane	The lipid b	0.389805	4.79E-05
GO:00057	mitochondrial ribosome	A ribosom	-0.38937	4.91E-05
GO:00057	late endosome	A prelysos	0.388147	5.24E-05
GO:00125	ER to Golgi transport vesicle membrane	The lipid b	0.388147	5.24E-05
GO:00975	Gemini of coiled bodies	Nuclear bc	-0.38607	5.87E-05
GO:00301	COPI-coated vesicle	A vesicle v	0.385223	6.14E-05
GO:00320	small-subunit processome	A large rib	-0.385	6.22E-05
GO:00319	TORC2 complex	A protein c	-0.38158	7.47E-05
GO:00343	nuclear periphery	The portio	-0.38016	8.06E-05
GO:00001	exosome (RNase complex)	Complex c	-0.37329	0.000116
GO:00007	condensed chromosome	A highly cc	-0.37322	0.000116
GO:00150	Cajal body	A class of	-0.37279	0.000119
GO:00710	catalytic step 2 spliceosome	A spliceos	-0.37237	0.000121
GO:00056	telomerase holoenzyme complex	Telomeras	-0.37071	0.000132
GO:00425	azurophil granule	Primary lys	0.367657	0.000155
GO:00430	neuronal cell body	The portio	0.365082	0.000177
GO:00056	nuclear origin of replication recognition complex	A multisub	-0.36373	0.00019
GO:00301	COPI vesicle coat	One of twc	0.36301	0.000197
GO:00057	vacuole	A closed s	0.360806	0.00022
GO:00466	anchored to plasma membrane	Tethered t	0.360216	0.000227
GO:00425	specific granule	Granule w	0.358864	0.000243
GO:00057	mitochondrial small ribosomal subunit	The smalle	-0.35716	0.000264
GO:00351	exon-exon junction complex	A multi-sul	-0.35574	0.000284
GO:00056	DNA replication factor C complex	A complex	-0.35515	0.000292
GO:00190	viral nucleocapsid	The compl	-0.35494	0.000295
GO:00099	cell surface	The exterr	0.350506	0.000367

GO:00319	early endosome membrane	The lipid b	0.34887	0.000398
GO:00163	nuclear matrix	The dense	-0.34787	0.000418
GO:00319	late endosome membrane	The lipid b	0.347015	0.000436
GO:00057	early endosome	A membra	0.346862	0.000439
GO:00057	mitochondrial proton-transporting ATP synthase complex	A proton-tr	-0.34588	0.00046
GO:00305	ribonucleoprotein complex	A macrom	-0.34523	0.000475
GO:00057	endoplasmic reticulum-Golgi intermediate compartment	A complex	0.344942	0.000482
GO:00056	basement membrane	A thin laye	0.343524	0.000516
GO:00056	DNA replication factor A complex	A conserv	-0.3428	0.000534
GO:00427	mitochondrial intermembrane space protein transporter compl	Soluble co	-0.34252	0.000541
GO:00328	dendrite cytoplasm	All of the c	0.341734	0.000562
GO:00016	male germ cell nucleus	The nucle	-0.33853	0.000655
GO:00008	lateral element	A proteina	-0.33523	0.000765
GO:00430	neuron projection	A prolonga	0.33401	0.00081
GO:00306	cytoplasmic vesicle membrane	The lipid b	0.331086	0.000928
GO:00431	ATP-binding cassette (ABC) transporter complex	A complex	-0.33008	0.000972
GO:00057	endoplasmic reticulum lumen	The volum	0.329951	0.000978
GO:00305	small nuclear ribonucleoprotein complex	A complex	-0.32938	0.001004
GO:00057	mitochondrial respiratory chain complex III	A protein c	-0.32923	0.001011
GO:00007	heterochromatin	A compact	-0.32659	0.001142
GO:00331	endoplasmic reticulum-Golgi intermediate compartment memb	The lipid b	0.326394	0.001152
GO:00451	apical part of cell	The region	0.326023	0.001172
GO:00310	nuclear pore outer ring	A subcom	-0.32517	0.001218
GO:00019	female pronucleus	The pronu	-0.3231	0.001338
GO:00009	spindle pole	Either of th	-0.32282	0.001355
GO:00164	proton-transporting two-sector ATPase complex	A large pr	0.322096	0.0014
GO:00058	trans-Golgi network	The netwo	0.321157	0.001461
GO:00314	cullin-RING ubiquitin ligase complex	Any ubiqui	-0.32061	0.001497
GO:00002	nuclear chromosome	A chromos	-0.3198	0.001552
GO:00058	microtubule associated complex	Any multin	0.317753	0.001701
GO:00059	caveola	A membra	0.314524	0.001962
GO:00056	DNA-directed RNA polymerase II, core complex	RNA polyn	-0.31324	0.002077
GO:00007	condensed nuclear chromosome	A highly co	-0.3123	0.002164
GO:00451	membrane raft	Any of the	0.311382	0.002252
GO:00451	pronucleus	The nucle	-0.3109	0.0023
GO:00007	chromosome, centromeric region	The region	-0.31088	0.002302
GO:00058	polar microtubule	Any of the	-0.30988	0.002405
GO:00312	SNARE complex	A protein c	0.309527	0.002441
GO:00007	condensed chromosome kinetochore	A multisub	-0.30896	0.002502
GO:00059	adherens junction	A cell junc	0.305665	0.002883
GO:00058	Arp2/3 protein complex	A stable pr	-0.30396	0.0031
GO:00057	integral to peroxisomal membrane	Penetratin	-0.30359	0.00315
GO:00301	endocytic vesicle	A membra	0.302872	0.003248
GO:00314	Cul4A-RING ubiquitin ligase complex	A ubiquitin	-0.30261	0.003284
GO:00058	cis-Golgi network	The netwo	0.300035	0.003661
GO:00707	pre-snoRNP complex	A ribonucle	-0.29966	0.003718
GO:00058	chaperonin-containing T-complex	A multisub	-0.2996	0.003728
GO:00163	apical plasma membrane	The region	0.297809	0.004018
GO:00021	semaphorin receptor complex	A stable bi	0.296587	0.004228
GO:00162	eukaryotic translation initiation factor 4F complex	The eukar	-0.29602	0.004328
GO:00007	chromatin	The ordere	-0.29495	0.004524
GO:00016	acrosomal vesicle	A structure	0.292179	0.00507
GO:00009	cytoplasmic mRNA processing body	A focus in	-0.29183	0.005143
GO:00706	HAUS complex	A protein c	-0.2908	0.005363
GO:00007	synaptonemal complex	A proteina	-0.29059	0.005411
GO:00453	phagocytic vesicle	A membra	0.289736	0.005601

GO:00301	integral to endoplasmic reticulum membrane	Penetratin	0.288972	0.005777
GO:00007	kinetochore	A multisub	-0.28744	0.006145
GO:00059	cell cortex	The regior	0.28703	0.006248
GO:00005	proteasome complex	A large mu	-0.2864	0.006409
GO:00301	membrane coat	Any of sev	0.285393	0.006671
GO:00009	condensed chromosome outer kinetochore	The regior	-0.28417	0.007005
GO:00306	axolemma	The portio	0.284019	0.007047
GO:00319	Golgi cisterna	Any of the	0.283102	0.007308
GO:00452	synapse	The junctio	0.282884	0.007372
GO:00140	postsynaptic density	The postsy	0.279523	0.008415
GO:00313	integral to mitochondrial inner membrane	Located su	-0.27924	0.008509
GO:00147	intercalated disc	A complex	0.278825	0.008648
GO:00432	lysosomal lumen	The volum	0.278171	0.008872
GO:00601	ciliary membrane	The portio	0.278171	0.008872
GO:00058	centrosome	A structure	-0.27776	0.009016
GO:00017	XY body	A structure	-0.27732	0.00917
GO:00098	cytoplasmic side of plasma membrane	The side (I	0.275661	0.009778
GO:00197	proteasome core complex, alpha-subunit complex	The protea	-0.27553	0.009828
GO:00163	apicolateral plasma membrane	The apical	0.27481	0.010104
GO:00008	origin recognition complex	A multisub	-0.27446	0.010241
GO:00717	WINAC complex	A SWI/SN	-0.27381	0.010501
GO:00056	U12-type spliceosomal complex	Any splice	-0.27339	0.01067
GO:00056	chromosome	A structure	-0.27191	0.011292
GO:00056	nuclear envelope	The double	-0.27125	0.011576
GO:00306	Golgi-associated vesicle membrane	The lipid b	-0.27104	0.011673
GO:00330	sarcoplasmic reticulum membrane	The lipid b	-0.26997	0.012155
GO:00330	sarcoplasmic reticulum lumen	The volum	0.268962	0.012624
GO:00162	eukaryotic 43S preinitiation complex	A protein c	-0.26711	0.013534
GO:00726	mitotic spindle	A spindle t	-0.26617	0.014017
GO:00451	intercellular bridge	A direct cc	-0.26589	0.014166
GO:00056	nucleolar ribonuclease P complex	A ribonucle	-0.26547	0.014386
GO:00057	small nucleolar ribonucleoprotein complex	A ribonucle	-0.26475	0.014776
GO:00082	gamma-tubulin ring complex	A multiprot	-0.26346	0.015497
GO:00226	proteasome accessory complex	A protein c	-0.26246	0.01608
GO:00718	MMXD complex	A protein c	-0.26218	0.016248
GO:00325	neuron projection membrane	The portio	0.260168	0.017486
GO:00058	kinetochore microtubule	Any of the	-0.25849	0.018587
GO:00056	anaphase-promoting complex	A ubiquitin	-0.2579	0.018987
GO:00431	dendritic shaft	Cylindric p	0.257746	0.019092
GO:00056	replication fork	The Y-sha	-0.25753	0.019243
GO:00332	paranode region of axon	An axon pa	0.257179	0.019486
GO:00332	node of Ranvier	An axon pa	0.256743	0.019795
GO:00059	axoneme	The bundle	0.255979	0.020345
GO:00147	spectrin-associated cytoskeleton	The part o	0.255826	0.020457
GO:00058	eukaryotic translation initiation factor 3 complex	A complex	-0.2557	0.020553
GO:00059	microvillus	Thin cylinc	0.254626	0.021353
GO:00306	synaptic vesicle membrane	The lipid b	0.254386	0.021537
GO:00125	endomembrane system	A collectio	0.252597	0.022949
GO:00059	tight junction	An occludi	0.252313	0.02318
GO:00487	presynaptic active zone	A specializ	-0.25103	0.024256
GO:00310	extracellular matrix	A structure	0.250262	0.024915
GO:00315	motile primary cilium	A primary	-0.24991	0.025222
GO:00059	phosphatidylinositol 3-kinase complex	A complex	0.24976	0.025357
GO:00304	growth cone	The migra	0.249105	0.025944
GO:00056	U5 snRNP	A ribonucle	-0.24825	0.026724
GO:00312	leading edge membrane	The portio	0.24784	0.027112

GO:00125	vesicle membrane	The lipid b	0.245483	0.029411
GO:00058	lipid particle	Any particl	0.245461	0.029433
GO:00001	nuclear exosome (RNase complex)	Complex c	-0.24533	0.029566
GO:00081	COP9 signalosome	A protein c	-0.24518	0.029721
GO:00314	Cul2-RING ubiquitin ligase complex	A ubiquitin	-0.24446	0.030464
GO:00058	eukaryotic translation initiation factor 2B complex	A multisub	-0.24433	0.030601
GO:00056	transcription factor TFIID complex	A complex	-0.2442	0.030738
GO:00431	terminal bouton	Terminal in	0.243912	0.031038
GO:00309	retromer complex	A conserve	0.243192	0.031809
GO:00017	sex chromatin	A condens	-0.24267	0.032381
GO:00164	vacuolar proton-transporting V-type ATPase complex	A proton-tr	0.241686	0.033478
GO:00705	BRCA1-A complex	A protein c	-0.24147	0.033726
GO:00058	spindle microtubule	Any microt	-0.24103	0.034227
GO:00332	eukaryotic 48S preinitiation complex	A protein c	-0.24068	0.034632
GO:00800	Cul4-RING ubiquitin ligase complex	A ubiquitin	-0.24005	0.035378
GO:00432	perikaryon	The portion	0.23981	0.035664
GO:00056	laminin-1 complex	A laminin c	0.238893	0.036777
GO:00713	MLL1 complex	A protein c	-0.23861	0.037127
GO:00059	brush border	Dense cov	0.236689	0.039577
GO:00303	T-tubule	Invaginatio	0.236624	0.039663
GO:00360	slit diaphragm	A specializ	0.235533	0.04112
GO:00007	condensin complex	A multisub	-0.23383	0.043485
GO:00008	ESCRT I complex	An endosc	0.233765	0.043579
GO:00057	DNA-directed RNA polymerase I complex	RNA polym	-0.23374	0.04361
GO:00974	neuronal postsynaptic density	A postsyna	-0.23267	0.04516
GO:00331	proton-transporting V-type ATPase, V1 domain	A protein c	0.232172	0.045903
GO:00353	transcriptionally active chromatin	The ordere	-0.23189	0.046328
GO:00056	Ada2/Gcn5/Ada3 transcription activator complex	A multiprot	-0.23154	0.046856
GO:00057	mitochondrial respiratory chain complex I	A protein c	-0.23139	0.047089
GO:00424	melanosome	A tissue-sp	0.230841	0.047927
GO:00319	nuclear membrane	Either of th	-0.23054	0.048403
GO:00300	cell junction	A cellular c	0.229336	0.05031
GO:00344	BBSome	A protein c	0.228899	0.051019
GO:00056	basal lamina	A thin shee	0.228397	0.051846
GO:00451	basal part of cell	The region	0.227328	0.053645
GO:00306	transport vesicle membrane	The lipid b	0.22711	0.054019
GO:00058	proteasome core complex	A multisub	-0.22689	0.054395
GO:00443	neuron projection terminus	The specia	0.226739	0.054659
GO:00059	cell-cell adherens junction	An adhere	0.22639	0.055268
GO:00319	vesicle	Any small,	0.226172	0.055651
GO:00310	Ino80 complex	A multisub	-0.22589	0.056153
GO:00512	spindle midzone	The area in	-0.22569	0.056503
GO:00304	axon	The long p	0.223641	0.060269
GO:00017	ruffle	Projection	0.223619	0.060311
GO:00018	phagocytic cup	An invagin	-0.2234	0.060724
GO:00057	peroxisomal membrane	The lipid b	-0.2215	0.064423
GO:00082	F-actin capping protein complex	A heterodi	0.22148	0.064467
GO:00352	ciliary rootlet	A cytoskel	0.220389	0.06668
GO:00057	mitochondrial large ribosomal subunit	The larger	-0.22019	0.067085
GO:00301	clathrin coat of coated pit	The coat fo	0.219778	0.067947
GO:00451	apical cortex	The region	0.219756	0.067993
GO:00163	lateral plasma membrane	The memb	0.219691	0.06813
GO:00432	myelin sheath	An electric	0.219538	0.068451
GO:00306	clathrin-coated vesicle membrane	The lipid b	0.219342	0.068865
GO:00003	transcription export complex	The transc	0.218884	0.06984
GO:00715	npBAF complex	A SWI/SN	-0.21884	0.069933

GO:00435	nuclear replication fork	The Y-sha	-0.2184	0.070874
GO:00430	NADPH oxidase complex	A enzyme	-0.21805	0.071634
GO:00310	platelet dense tubular network membrane	The lipid b	0.217967	0.071825
GO:00309	intraciliary transport particle B	The larger	0.217771	0.072257
GO:00098	external side of plasma membrane	The side (l	0.216963	0.074054
GO:00704	nonhomologous end joining complex	A protein c	-0.21557	0.077253
GO:00344	centriolar satellite	A small (7(	0.214781	0.079103
GO:00304	dendrite	A neuron p	0.21441	0.07999
GO:00059	zonula adherens	A cell-cell	0.212927	0.08362
GO:00058	dynactin complex	A 20S mul	0.212097	0.085709
GO:00001	histone deacetylase complex	A protein c	0.211901	0.08621
GO:00055	cellular_component	The part o	-0.21146	0.087332
GO:00171	Golgi transport complex	A complex	0.21105	0.08841
GO:00431	axon initial segment	Portion of	0.210832	0.088981
GO:00970	synaptic membrane	A specializ	-0.21033	0.090308
GO:00321	cleavage furrow	In animal c	0.21033	0.090308
GO:00723	primary cilium	A cilium fo	0.210112	0.09089
GO:00301	AP-1 adaptor complex	A heterote	0.210112	0.09089
GO:00332	transcription factor TFTC complex	A protein c	-0.2097	0.092004
GO:00164	RISC complex	A ribonucle	-0.20904	0.093786
GO:00160	sarcoglycan complex	A protein c	0.208846	0.094326
GO:00465	intercellular canaliculus	An extrem	0.208475	0.095354
GO:00347	calcium channel complex	An ion cha	-0.20834	0.095719
GO:00162	prefoldin complex	A multisub	-0.20804	0.096575
GO:00007	nuclear telomere cap complex	A complex	-0.20747	0.098182
GO:00058	spindle	The array	-0.20684	0.1
GO:00331	melanosome membrane	The lipid b	0.206773	0.10019
GO:00055	proteinaceous extracellular matrix	A layer cor	0.206402	0.101271
GO:00452	proton-transporting ATP synthase complex, coupling factor F(c	All non-F1	-0.20614	0.102039
GO:00300	Z disc	Platelike re	0.205835	0.102942
GO:00058	cytoskeleton	Any of the	0.20518	0.104899
GO:00057	nuclear heterochromatin	A condens	-0.20511	0.105096
GO:00432	apical junction complex	A function	0.204853	0.105889
GO:00703	exocytic vesicle	A transpor	-0.20413	0.108094
GO:00080	spectrin	Membrane	0.203849	0.108973
GO:00300	lamellipodium	A thin shee	0.203042	0.111508
GO:00301	AP-type membrane coat adaptor complex	Any of sev	0.202933	0.111854
GO:00323	photoreceptor connecting cilium	A nonmotil	0.20254	0.113107
GO:00431	P granule	A small cy	0.202474	0.113317
GO:00421	T cell receptor complex	A protein c	-0.20219	0.114231
GO:00005	glycosylphosphatidylinositol-N-acetylglucosaminyltransferase (	An enzyme	0.201493	0.116507
GO:00059	cell-cell junction	A cell junc	0.201405	0.116794
GO:00058	striated muscle thin filament	Filaments	-0.20058	0.11955
GO:00019	photoreceptor inner segment	The inner s	0.200118	0.121096
GO:00431	dendritic spine	Protrusion	0.1999	0.121838
GO:00423	sarcolemma	The outer	0.199354	0.123709
GO:00301	integral to Golgi membrane	Located su	0.19918	0.124313
GO:00165	DNA-directed RNA polymerase II, holoenzyme	Large prot	0.198678	0.126062
GO:00080	synaptic vesicle	A secretor	0.198634	0.126215
GO:00058	proteasome regulatory particle	A multisub	-0.19726	0.131113
GO:00165	sarcoplasm	The cytopl	-0.19713	0.131588
GO:00001	transcription factor TFIIIC complex	A heterotri	-0.19689	0.132461
GO:00315	motile cilium	A cilium wl	0.196757	0.13294
GO:00059	fascia adherens	A cell-cell	-0.19669	0.133179
GO:00099	basal plasma membrane	The regior	0.195055	0.139288
GO:00423	paraspeckles	Discrete su	-0.19471	0.14062

GO:00426	actomyosin	Any compl	-0.19455	0.141206
GO:00103	chromocenter	A region ir	-0.19377	0.144251
GO:00057	Golgi stack	The set of	0.193048	0.147089
GO:00427	presynaptic membrane	A specializ	0.19283	0.147958
GO:00424	nuclear inclusion body	An intranu	-0.19183	0.152007
GO:00198	extrinsic to membrane	Loosely bo	-0.19132	0.154065
GO:00306	endocytic vesicle membrane	The lipid b	-0.19019	0.158799
GO:00453	clathrin-coated endocytic vesicle	A clathrin-	0.189753	0.160651
GO:00198	extrinsic to plasma membrane	Loosely bo	0.188924	0.164216
GO:00425	chromaffin granule membrane	The lipid b	0.186131	0.17669
GO:00700	clathrin-sculpted monoamine transport vesicle membrane	The lipid b	0.186043	0.177092
GO:00057	peroxisome	A small org	-0.18554	0.179414
GO:00315	brush border membrane	The portio	-0.18465	0.183614
GO:00159	small ribosomal subunit	The smalle	-0.18419	0.185794
GO:00160	cytoplasmic membrane-bounded vesicle	A membra	0.183469	0.189262
GO:00001	histone acetyltransferase complex	A protein c	0.183272	0.190216
GO:00058	cytoplasmic microtubule	Any microt	0.18325	0.190323
GO:00007	chromosome, telomeric region	The termir	-0.18299	0.191602
GO:00357	myelin sheath adaxonal region	The regior	0.182072	0.196131
GO:00147	junctional sarcoplasmic reticulum membrane	The part o	0.181156	0.200742
GO:00308	beta-catenin destruction complex	A cytoplas	0.180414	0.204536
GO:00312	intrinsic to membrane	Located in	0.178995	0.211943
GO:00329	protein-DNA complex	A macrom	0.178864	0.212637
GO:00301	clathrin-coated vesicle	A vesicle v	0.178624	0.213914
GO:00165	SWI/SNF complex	A SWI/SN	-0.17856	0.214263
GO:00716	endocytic vesicle lumen	The volum	0.178559	0.214263
GO:00301	clathrin coat of trans-Golgi network vesicle	A clathrin c	0.178363	0.215313
GO:00104	cytoplasmic stress granule	A dense ag	-0.17832	0.215547
GO:00436	axon terminus	Terminal ir	0.177795	0.218369
GO:00315	filopodium membrane	The portio	0.176988	0.222775
GO:00316	I band	A region o	0.176966	0.222895
GO:00164	myosin complex	A protein c	0.176551	0.225184
GO:00350	ESC/E(Z) complex	A multimer	-0.17605	0.22798
GO:00425	glycogen granule	Cytoplasm	-0.17585	0.22908
GO:00429	cell projection	A prolonga	0.175548	0.230801
GO:00972	alveolar lamellar body	A specializ	0.175482	0.231171
GO:00314	Cul3-RING ubiquitin ligase complex	A ubiquitin	0.175417	0.231541
GO:00055	collagen	Any of the	0.175351	0.231912
GO:00712	WASH complex	A protein c	0.174937	0.234271
GO:00002	pericentriolar material	A network	-0.17483	0.234895
GO:00164	myosin II complex	A myosin c	-0.17419	0.238537
GO:00310	platelet alpha granule lumen	The volum	0.173846	0.240565
GO:00056	DNA-directed RNA polymerase III complex	RNA polym	-0.17343	0.242989
GO:00432	intracellular membrane-bounded organelle	Organized	0.173278	0.243887
GO:00973	photoreceptor disc membrane	Ovally-sha	-0.17326	0.244015
GO:00003	cyclin-dependent protein kinase holoenzyme complex	Cyclin-dep	-0.1723	0.249717
GO:00612	clathrin-sculpted gamma-aminobutyric acid transport vesicle m	The lipid b	0.172056	0.251158
GO:00007	condensed nuclear chromosome, centromeric region	The regior	0.17186	0.252341
GO:00056	cell	The basic	0.17186	0.252341
GO:00347	sodium channel complex	An ion cha	0.171489	0.254588
GO:00057	mitochondrial inner membrane presequence translocase comp	The protei	-0.17142	0.254986
GO:00312	cell projection membrane	The portio	0.171358	0.255384
GO:00058	ribosome	An intracel	-0.16998	0.263857
GO:00442	dendrite terminus	A structure	0.169612	0.266178
GO:00704	respiratory chain	The protei	-0.16883	0.27118
GO:00331	proton-transporting two-sector ATPase complex, catalytic dom	A protein c	0.168041	0.276207

GO:00165	NuRD complex	An approx	-0.16726	0.281302
GO:00304	midbody	A thin cyto	0.167038	0.282729
GO:00306	phagocytic vesicle membrane	The lipid b	0.166492	0.28632
GO:00723	PTW/PP1 phosphatase complex	A protein s	0.166427	0.286753
GO:00351	PRC1 complex	An insect r	0.164986	0.296399
GO:00308	rough endoplasmic reticulum membrane	The lipid b	0.164485	0.299814
GO:00315	AMP-activated protein kinase complex	A protein c	0.164005	0.303107
GO:00058	acetylcholine-gated channel complex	A homo- o	-0.1635	0.306576
GO:00055	collagen type IV	A collagen	0.163284	0.308093
GO:00310	BLOC-1 complex	A protein c	0.162477	0.313751
GO:00170	transcriptional repressor complex	A protein c	-0.16198	0.317304
GO:00313	integral to mitochondrial outer membrane	Located su	-0.16191	0.31777
GO:00082	oligosaccharyltransferase complex	A protein c	0.161779	0.318702
GO:00315	neuromuscular junction	The junctio	0.16071	0.326387
GO:00057	rough endoplasmic reticulum	The rough	0.159924	0.332113
GO:00057	Golgi-associated vesicle	Any vesicle	0.159575	0.334679
GO:00306	clathrin-coated endocytic vesicle membrane	The lipid b	-0.15933	0.336452
GO:00058	voltage-gated calcium channel complex	A protein c	0.159204	0.337421
GO:00343	low-density lipoprotein particle	A lipoprote	0.15892	0.339527
GO:00452	postsynaptic membrane	A specializ	-0.15885	0.340015
GO:00324	filopodium tip	The end of	0.158353	0.343767
GO:00550	recycling endosome	Organelle	-0.15833	0.34393
GO:00058	actin filament	A filament	0.158004	0.346393
GO:00325	dendrite membrane	The portio	0.15748	0.350357
GO:00056	nuclear envelope lumen	The region	-0.15735	0.351353
GO:00347	chloride channel complex	An ion cha	0.156564	0.357366
GO:00166	nuclear body	Extra-nucle	-0.15648	0.358038
GO:00300	desmosome	A cell-cell	-0.15621	0.36006
GO:00226	cytosolic large ribosomal subunit	The large s	0.156062	0.361242
GO:00432	receptor complex	Any protein	0.155844	0.362936
GO:00165	Sin3 complex	A multipro	-0.15554	0.365316
GO:00430	costamere	Regular pe	0.155342	0.366851
GO:00719	cell periphery	The part o	0.154993	0.369591
GO:00314	M band	The midlin	0.154774	0.37131
GO:00057	centromeric heterochromatin	A region of	0.154163	0.376149
GO:00001	exocyst	A protein c	-0.15412	0.376497
GO:00301	transport vesicle	Any of the	0.153858	0.378584
GO:00357	myelin sheath abaxonal region	The region	-0.15364	0.380329
GO:00199	cyclin-dependent protein kinase activating kinase holoenzyme	A protein c	-0.15357	0.380854
GO:00312	intrinsic to cytoplasmic side of plasma membrane	Located in	-0.15344	0.381904
GO:00056	interstitial matrix	A type of e	-0.1534	0.382254
GO:00017	immunological synapse	An area of	0.153138	0.384362
GO:00080	voltage-gated potassium channel complex	A protein c	-0.15251	0.389484
GO:00007	nucleosome	A complex	0.152221	0.391794
GO:00319	endosome lumen	The volum	-0.15141	0.398413
GO:00302	dynein complex	Any of sev	0.151261	0.399673
GO:00300	sarcomere	The repea	-0.15122	0.400034
GO:00057	signal recognition particle, endoplasmic reticulum targeting	A ribonucle	0.150148	0.408925
GO:00426	MHC class I protein complex	A transme	0.149734	0.412403
GO:00311	pseudopodium	A tempora	-0.14969	0.41277
GO:00057	peroxisomal matrix	The volum	-0.14947	0.414609
GO:00300	CCR4-NOT complex	The evolut	0.148665	0.421454
GO:00056	integral to nuclear inner membrane	Located su	0.14657	0.439512
GO:00058	neurofilament	A type of i	0.146482	0.440274
GO:00361	sperm flagellum	A microtub	-0.14637	0.441227
GO:00315	microvillus membrane	The portio	0.146308	0.441799

GO:00316	A band	The dark-s	0.146112	0.443518
GO:00056	nuclear inner membrane	The inner,	-0.14578	0.446392
GO:00058	troponin complex	A complex	-0.14565	0.447545
GO:00310	platelet alpha granule	A secretor	0.145588	0.448121
GO:00325	trans-Golgi network membrane	The lipid b	0.145195	0.45159
GO:00057	vacuolar membrane	The lipid b	0.14478	0.455267
GO:00058	polysome	Several rib	-0.14452	0.457598
GO:00358	ciliary transition zone	A region o	-0.14413	0.461105
GO:00001	PCAF complex	A large mu	-0.14334	0.46816
GO:00485	signal recognition particle	A complex	0.143231	0.469145
GO:00426	MHC class II protein complex	A transme	-0.14262	0.474676
GO:00432	contractile fiber	Fibers, cor	-0.14207	0.479641
GO:00358	site of double-strand break	A region o	0.141507	0.484832
GO:00319	membrane-bounded vesicle	Any small,	0.141355	0.486234
GO:00017	photoreceptor outer segment	The outer	0.141355	0.486234
GO:00058	axonemal dynein complex	A dynein c	-0.14133	0.486434
GO:00059	cAMP-dependent protein kinase complex	An enzyme	-0.14116	0.488039
GO:00308	cortical actin cytoskeleton	The portio	0.140853	0.490854
GO:00055	membrane attack complex	A protein c	-0.14037	0.495292
GO:00360	ciliary basal body	A membra	0.13998	0.498937
GO:00426	chylomicron	A large lip	0.139434	0.50402
GO:00425	zymogen granule membrane	The lipid b	0.139434	0.50402
GO:00426	photoreceptor outer segment membrane	The memb	0.138409	0.513635
GO:00315	nonmotile primary cilium	A primary	-0.13834	0.514252
GO:00059	focal adhesion	Small regi	0.138191	0.515691
GO:00308	cortical cytoskeleton	The portio	-0.13791	0.518369
GO:00309	STAGA complex	A large mu	-0.13786	0.518781
GO:00431	varicosity	Non-termi	-0.13784	0.518987
GO:00312	extrinsic to external side of plasma membrane	Loosely bo	0.137689	0.520432
GO:00442	cell body	The portio	-0.13756	0.521672
GO:00700	extracellular vesicular exosome	A membra	0.137187	0.525191
GO:00056	lamin filament	Any of a g	0.13662	0.530592
GO:00328	neuronal cell body membrane	The plasm	0.136009	0.536431
GO:00058	mRNA cleavage factor complex	Any macro	-0.13586	0.537895
GO:00715	integral to lumenal side of endoplasmic reticulum membrane	Penetratin	0.135856	0.537895
GO:00190	SCF ubiquitin ligase complex	A ubiquitin	-0.13572	0.539151
GO:00300	myofibril	The contra	-0.13555	0.540827
GO:00004	THO complex part of transcription export complex	The THO c	-0.13548	0.541456
GO:00003	THO complex	The THO c	-0.13548	0.541456
GO:00059	gap junction	A cell-cell	0.13531	0.543134
GO:00019	uropod	A membra	0.135114	0.545025
GO:00550	recycling endosome membrane	The lipid b	0.134437	0.551554
GO:00451	intermediate filament cytoskeleton	Cytoskelet	-0.13431	0.552821
GO:00312	intrinsic to plasma membrane	Located in	0.134263	0.553243
GO:00057	nuclear euchromatin	The disper	0.132495	0.570439
GO:00007	nuclear chromatin	The ordere	0.13219	0.573427
GO:00347	secretory granule lumen	The volum	0.132146	0.573854
GO:00300	hemidesmosome	A cell-subs	-0.13191	0.576205
GO:00308	Mre11 complex	Trimeric pr	-0.13184	0.576847
GO:00001	protein phosphatase type 2A complex	A protein c	-0.1318	0.577275
GO:00312	anchored to membrane	Tethered t	-0.13127	0.582416
GO:00080	transcription elongation factor complex	Any protei	0.130728	0.587784
GO:00058	muscle myosin complex	A filament	0.129921	0.595749
GO:00329	myosin filament	A protein c	-0.12837	0.611094
GO:00313	anchored to external side of plasma membrane	Tethered t	-0.128	0.614778
GO:00343	high-density lipoprotein particle	A lipoprote	0.127586	0.6189

GO:00226	cytosolic small ribosomal subunit	The small	-0.12756	0.619117
GO:00324	stereocilium	An actin-b	0.127215	0.62259
GO:00312	cell leading edge	The area c	0.126778	0.626936
GO:00083	integrin complex	A protein c	0.126451	0.630197
GO:00715	nBAF complex	A SWI/SN	-0.12595	0.6352
GO:00333	chromatoid body	A ribonucle	-0.12584	0.636288
GO:00707	MOZ/MORF histone acetyltransferase complex	A histone a	-0.12492	0.645431
GO:00481	Set1C/COMPASS complex	A conserve	-0.12392	0.655449
GO:00311	septin complex	Any of sev	0.12392	0.655449
GO:00432	Schmidt-Lanterman incisure	Regions w	0.123549	0.659151
GO:00021	podosome	An actin-ri	-0.12287	0.6659
GO:00725	ER membrane protein complex	A transme	-0.12178	0.676774
GO:00325	integral to mitochondrial membrane	Located su	0.121585	0.678729
GO:00156	actin cytoskeleton	The part o	0.121061	0.68394
GO:00058	cytoplasmic dynein complex	Any dynein	-0.12056	0.688928
GO:00059	cilium	A specializ	0.12045	0.690011
GO:00421	alpha-beta T cell receptor complex	A T cell re	0.12045	0.690011
GO:00360	TCTN-B9D complex	A protein c	-0.12012	0.693259
GO:00320	bleb	A cell exte	0.120014	0.694341
GO:00057	multivesicular body	A type of la	0.11973	0.697152
GO:00015	cornified envelope	An insolub	-0.11942	0.700177
GO:00432	protein complex	Any macro	0.119338	0.70104
GO:00301	clathrin coat	A membra	0.118923	0.705138
GO:00162	inclusion body	A discrete	0.118879	0.705569
GO:00319	filamentous actin	A two-stra	-0.1186	0.708369
GO:00600	excitatory synapse	A synapse	-0.11849	0.709445
GO:00320	integrator complex	A protein c	-0.11676	0.726367
GO:00314	Cul5-RING ubiquitin ligase complex	A ubiquitin	-0.11563	0.737415
GO:00308	meiotic cohesin complex	A cohesin	-0.11534	0.740163
GO:00316	spindle pole centrosome	A centroso	-0.1148	0.745434
GO:00057	Golgi lumen	The volum	-0.11469	0.746485
GO:00972	sperm midpiece	The highly	-0.11467	0.746695
GO:00058	kinesin complex	Any compl	-0.11436	0.749634
GO:00163	catenin complex	Complex c	-0.11428	0.750472
GO:00057	mitochondrial respiratory chain	The protei	0.114122	0.751938
GO:00156	microtubule cytoskeleton	The part o	-0.11399	0.753193
GO:00353	microtubule plus-end	The growir	0.113773	0.755281
GO:00160	dystrophin-associated glycoprotein complex	A multipro	0.113075	0.761935
GO:00009	gamma-tubulin complex	A multipro	-0.11299	0.762764
GO:00058	sodium:potassium-exchanging ATPase complex	Sodium:po	-0.11246	0.767722
GO:00171	N-methyl-D-aspartate selective glutamate receptor complex	An assem	0.112071	0.771424
GO:00015	voltage-gated sodium channel complex	A sodium c	0.111438	0.777355
GO:00056	nuclear outer membrane	The outer,	-0.11041	0.786878
GO:00331	proton-transporting two-sector ATPase complex, proton-transp	A protein c	-0.10969	0.793493
GO:00057	mitochondrial outer membrane	The outer,	0.109344	0.796678
GO:00319	mitochondrial membrane	Either of th	-0.10913	0.798661
GO:00058	mRNA cleavage and polyadenylation specificity factor complex	A multisub	-0.10899	0.799848
GO:00973	ripoptosome	A protein c	0.108667	0.802806
GO:00308	HOPS complex	A multimer	0.108056	0.80829
GO:00166	PML body	A class of	0.107991	0.808875
GO:00301	clathrin adaptor complex	A membra	-0.10795	0.809264
GO:00056	holo TFIIH complex	A complex	0.107773	0.810819
GO:00059	coated pit	A part of th	0.107074	0.816997
GO:00165	Cdc73/Paf1 complex	A multipro	-0.10642	0.822724
GO:00058	hemoglobin complex	An iron-co	0.106289	0.823862
GO:00450	keratin filament	A filament	-0.10616	0.824997

GO:00310	dense core granule	Electron-d	0.106136	0.825186
GO:00056	transcription factor complex	A protein c	0.105787	0.828199
GO:00165	mediator complex	A protein c	0.105656	0.829325
GO:00058	microtubule organizing center	An intracel	0.105023	0.834724
GO:00306	secretory granule membrane	The lipid b	-0.1045	0.839143
GO:00059	microtubule basal body	A short cyl	0.104499	0.839143
GO:00315	PcG protein complex	A chromat	-0.10441	0.839875
GO:00017	stress fiber	A contracti	-0.10393	0.843879
GO:00058	centriole	A cellular c	0.103648	0.846226
GO:00442	juxtaparanode region of axon	A region o	0.103648	0.846226
GO:00056	nuclear lamina	The fibrou	-0.10328	0.849274
GO:00310	platelet alpha granule membrane	The lipid b	0.103125	0.850522
GO:00165	sarcoplasmic reticulum	A fine retic	0.102426	0.856173
GO:00301	trans-Golgi network transport vesicle	A vesicle t	-0.10188	0.860525
GO:00324	stereocilium bundle	An actin-ba	-0.10142	0.864137
GO:00015	microfibril	Extracellul	-0.10094	0.867877
GO:00164	unconventional myosin complex	A portman	-0.10072	0.869563
GO:00356	CD40 receptor complex	A protein c	0.099808	0.876535
GO:00162	aggresome	An inclusio	0.099786	0.876699
GO:00325	dendritic spine membrane	The portio	0.098673	0.884927
GO:00312	CD95 death-inducing signaling complex	A protein c	0.097866	0.89073
GO:00343	spherical high-density lipoprotein particle	A mature h	0.097648	0.892274
GO:00343	very-low-density lipoprotein particle	A triglyceri	0.097015	0.896691
GO:00308	smooth endoplasmic reticulum membrane	The lipid b	0.096338	0.901313
GO:00325	growth cone membrane	The portio	-0.09579	0.904963
GO:00058	microtubule	Any of the	-0.09555	0.906547
GO:00310	platelet dense granule membrane	The lipid b	0.095444	0.907263
GO:00312	death-inducing signaling complex	A protein c	0.094724	0.911914
GO:00432	intracellular organelle	Organized	0.094658	0.912331
GO:00322	alpha-amino-3-hydroxy-5-methyl-4-isoxazolepropionic acid sel	An assemb	-0.09461	0.912609
GO:00310	organelle membrane	The lipid b	-0.09451	0.9133
GO:00057	mitochondrial envelope	The double	0.093873	0.917252
GO:00055	fibrinogen complex	A highly sc	0.093415	0.920053
GO:00312	extrinsic to cytoplasmic side of plasma membrane	Loosely bo	0.092956	0.922802
GO:00007	nuclear chromosome, telomeric region	The termir	0.091494	0.931224
GO:00312	lamellipodium membrane	The portio	-0.08709	0.953319
GO:00350	histone methyltransferase complex	A multimer	0.086345	0.956546
GO:00059	connexon complex	An assemb	-0.08589	0.958469
GO:00058	heterotrimeric G-protein complex	Any of a fa	-0.08582	0.958739
GO:00325	ruffle membrane	The portio	0.085515	0.959986
GO:00352	NuA4 histone acetyltransferase complex	A complex	0.085494	0.960074
GO:00083	ionotropic glutamate receptor complex	A multimer	0.084424	0.964244
GO:00020	acrosomal membrane	The memb	0.084053	0.965623
GO:00083	IkappaB kinase complex	A trimeric	0.083922	0.966101
GO:00312	intrinsic to endoplasmic reticulum membrane	Located in	-0.08261	0.970651
GO:00301	AP-2 adaptor complex	A heterote	-0.08189	0.972974
GO:00301	filopodium	Thin, stiff p	-0.08146	0.97432
GO:00442	axonal growth cone	The migra	0.080148	0.978089
GO:00301	secretory granule	A small su	-0.0791	0.980819
GO:00706	MLL5-L complex	A protein c	0.072488	0.992851
GO:00058	intermediate filament	A cytoskel	-0.0712	0.994302
GO:00056	chromatin silencing complex	Any protei	0.071114	0.994391
GO:00331	proton-transporting V-type ATPase, V0 domain	A protein c	0.069346	0.995986

corr. pval	amiodaro	amitriptyli	amodiaqu	astemizol	bromperic	chloroqui	chlorpron	chlorprotl	clemastin
5.03E-08	143	74	1183	50	160	312	187	216	206
5.18E-07	404	121	96	72	735	931	854	6	33
2.7E-05	228	586	917	128	675	533	396	55	272
8.93E-05	261	1057	593	1278	1146	972	1126	1266	1147
0.000117	621	1188	1243	1274	1063	856	859	974	1259
0.000218	838	1177	440	1306	1135	172	1006	1167	1053
0.000309	9	1274	1007	1167	1127	774	1233	1210	1221
0.000655	57	494	551	238	328	228	386	950	889
0.001073	215	286	112	254	1013	96	616	561	421
0.001393	283	714	594	122	205	659	264	404	530
0.001462	24	86	990	400	1176	868	379	290	51
0.001555	499	595	53	6	531	999	218	67	30
0.001828	521	1113	864	1238	1262	193	1030	998	767
0.002964	803	1135	220	1081	1072	577	306	509	1218
0.002991	148	609	911	851	662	96	1116	993	1207
0.003015	563	72	408	449	283	514	243	242	37
0.004275	1147	580	869	1254	825	552	853	1200	1252
0.008048	536	572	200	506	1045	661	1033	268	251
0.009918	1079	1308	731	192	975	309	1191	35	44
0.010168	1077	1089	153	1216	759	564	1023	937	657
0.010673	101	1152	313	1189	863	1015	579	1255	1259
0.012139	484	432	588	24	684	1098	332	177	64
0.013379	551	350	806	61	268	223	628	227	127
0.013645	70	894	252	114	559	749	871	192	112
0.016017	211	98	378	439	672	996	806	36	10
0.017719	747	968	413	1034	970	648	726	839	887
0.019241	327	402	517	228	993	1246	474	368	296
0.020023	603	989	1142	918	216	932	824	906	1301
0.023893	430	737	904	1148	981	961	874	1130	505
0.026636	13	49	709	1021	1129	713	790	737	192
0.027278	960	1048	516	640	87	1001	751	1268	1261
0.029155	770	481	870	69	460	603	524	333	371
0.029155	18	274	1121	7	255	361	383	685	354
0.032622	627	1170	1066	1290	1005	1235	1299	1164	948
0.034156	1080	280	825	86	310	132	272	67	111
0.034561	843	1181	1200	1141	1035	782	253	1075	787
0.041538	744	1256	993	814	1002	1006	508	654	539
0.044802	694	1153	995	1196	1060	324	566	1178	696
0.064385	1194	916	98	1065	852	958	713	685	1280
0.064605	1220	1204	785	1293	1256	450	611	1247	546
0.066095	895	965	737	860	1144	1101	621	937	1160
0.06754	655	1013	1016	1284	1211	374	500	757	1130
0.073627	1017	713	1302	746	817	141	979	1124	949
0.086224	707	273	68	1146	769	413	82	310	52
0.098402	295	1215	186	390	32	471	786	822	468
0.105435	1226	1185	361	922	1268	899	331	867	732
0.10937	996	204	140	69	635	470	553	286	4
0.122295	175	55	133	633	120	341	926	526	132
0.125987	383	287	406	543	18	858	85	90	401
0.134868	776	920	482	423	460	1208	179	343	36
0.146882	1000	522	1100	1018	507	426	980	1305	1201
0.157658	782	1285	253	1224	1234	587	1130	879	555
0.16235	768	883	374	1182	1181	882	1118	1206	1110
0.164121	1117	971	1030	1020	99	690	550	944	800
0.204263	591	105	380	273	679	773	508	455	261

0.221307	248	795	1248	390	1122	464	384	649	190
0.232413	509	1300	1229	1226	253	774	1039	824	836
0.242239	240	731	961	115	330	420	474	540	29
0.244043	333	1175	716	296	780	812	516	722	34
0.255948	390	1126	515	829	1011	723	147	937	1034
0.264186	110	1238	791	1197	283	436	709	212	1280
0.267832	1059	382	1078	165	579	963	225	457	419
0.286785	456	735	622	337	136	944	367	84	605
0.296883	832	869	829	1154	1031	266	661	1204	1288
0.300952	86	1015	1183	837	511	948	416	1012	512
0.312495	1102	101	979	346	248	409	775	82	340
0.36408	1286	886	490	1281	1126	430	1081	1263	811
0.425309	1135	1070	1304	912	345	314	1148	1274	763
0.450374	750	299	367	326	1087	834	57	463	470
0.516072	473	791	621	72	569	318	1193	614	107
0.540619	279	434	1210	1289	1205	700	780	979	716
0.543901	580	103	917	292	389	1134	1255	248	364
0.558335	484	525	719	1263	319	1000	696	969	1302
0.562281	815	391	1126	1212	849	269	570	758	1227
0.63476	1215	780	397	1257	296	747	781	744	357
0.640485	923	499	1102	72	687	1041	50	450	83
0.651431	41	801	494	240	88	357	1272	75	646
0.677206	692	799	205	1082	532	943	30	1283	130
0.744022	1267	1260	468	1089	228	888	936	958	994
0.753629	1125	1222	923	1195	1124	222	951	777	666
0.778537	797	499	864	486	352	459	1269	422	131
0.812144	928	37	1019	546	21	570	1106	1055	49
0.832299	1216	1230	623	1301	1202	176	1109	1290	627
0.862984	903	1203	1125	881	944	865	845	1280	595
0.945735	233	1028	107	673	419	550	724	928	9
1.091095	335	104	155	607	1252	1243	330	532	295
1.15462	844	1211	759	919	254	159	1112	1288	1221
1.20306	881	1131	457	1309	1104	70	1059	1278	577
1.252186	1136	240	1028	447	288	1155	1153	75	1090
1.278654	1154	1081	394	1287	601	1251	605	808	1218
1.279869	1211	1234	789	1232	870	366	752	1197	908
1.336944	969	1192	409	764	666	657	1280	1273	45
1.357342	253	415	294	362	449	850	212	507	5
1.391104	1070	1005	801	1133	1017	328	100	1046	867
1.603034	567	144	990	1019	498	891	300	162	47
1.723833	503	615	621	1262	1200	20	1238	1023	1017
1.751252	1051	179	642	1187	1306	82	1005	742	853
1.805629	288	1142	704	607	537	739	271	8	605
1.825785	514	829	1056	1142	1242	5	997	1140	1019
2.035336	1111	184	924	297	235	813	338	142	43
2.067288	1187	744	838	910	1159	927	487	1107	1267
2.072974	625	1014	1193	600	71	1119	827	952	1301
2.234084	351	241	688	480	1024	1100	639	161	465
2.350643	746	853	720	235	525	104	697	88	140
2.406637	847	1309	1190	289	958	707	773	1039	1045
2.515506	1174	895	631	952	495	584	1086	1112	254
2.819054	1109	14	104	19	259	759	41	832	702
2.85959	1305	1136	316	1197	1160	659	754	868	1199
2.981772	1109	553	927	833	1089	1112	1054	1255	68
3.008377	1112	1207	801	1271	1285	513	896	1031	926
3.114226	149	651	553	20	700	117	244	259	227

3.212108	280	683	573	24	725	763	190	230	1032
3.416362	600	1282	817	1226	885	483	214	1120	835
3.473814	765	811	870	1069	1168	288	101	203	56
3.563207	155	1226	483	1131	667	402	415	935	1291
3.709317	349	914	266	553	129	959	830	438	13
3.894559	1283	538	788	1023	818	297	738	989	852
3.918298	545	167	413	785	596	272	45	310	778
4.063522	1056	126	525	140	319	949	258	331	174
4.098815	1100	414	658	475	454	595	588	463	368
4.67874	587	407	786	620	223	723	622	582	702
4.730956	1080	1029	526	996	641	997	1243	1178	655
4.808224	111	831	294	551	1290	825	192	119	547
4.932562	223	333	834	391	849	431	782	386	200
4.932562	41	232	609	361	1215	1057	560	432	299
5.012812	998	1133	1139	1043	1240	483	762	900	95
5.098563	358	659	1281	947	1262	754	875	1035	1106
5.43669	88	1009	524	274	1128	588	448	103	649
5.464231	107	994	582	465	684	991	108	962	951
5.617977	157	1094	423	512	896	522	733	622	1137
5.693921	1225	864	218	735	1273	812	298	907	538
5.838827	860	1189	637	1159	523	857	1112	98	86
5.932317	450	945	910	1273	119	495	239	777	1307
6.278113	590	852	656	1231	524	264	606	654	234
6.436378	946	893	37	1309	1053	489	973	1054	248
6.489929	232	398	372	961	482	686	852	1233	768
6.758184	488	128	193	1075	948	978	788	871	787
7.019076	536	349	475	599	596	1097	768	403	381
7.525151	546	1273	741	399	830	309	943	501	796
7.793473	890	639	1097	1110	1036	558	229	863	63
7.876271	974	1144	938	847	1296	620	1118	372	1051
7.998705	560	852	1021	1181	176	72	930	1282	1292
8.215426	322	1287	338	1297	1171	1011	98	540	1301
8.616073	416	1219	534	1107	61	807	262	512	962
8.94051	728	1281	101	935	139	328	633	694	1255
9.034162	230	980	1018	1121	279	1034	718	567	1253
9.72244	646	705	252	498	403	38	773	345	310
10.33416	713	1148	101	554	1203	315	407	1194	145
10.55666	451	1277	1132	622	1061	252	879	1117	870
10.61504	73	447	449	481	109	1179	6	18	19
10.69894	804	593	1048	1046	784	1001	1015	876	366
10.83441	477	313	933	558	384	83	60	1119	546
11.0059	562	642	944	586	214	716	601	352	697
11.31184	1147	56	704	172	154	65	88	761	102
11.37393	1084	352	261	547	79	860	90	666	854
11.42739	842	1287	1096	343	666	246	981	619	720
11.87243	1007	529	1083	1037	772	251	722	653	699
11.97443	227	1085	1144	950	665	1140	54	687	332
12.75969	880	595	823	596	561	599	1039	536	755
12.8883	183	377	806	356	471	1116	383	511	734
13.48656	1088	1151	302	1038	631	487	811	680	704
13.85298	421	240	419	409	250	540	167	721	1245
14.0234	1010	695	1237	519	1061	635	1080	1147	1164
14.09854	1054	996	367	278	482	978	50	54	329
14.42463	697	1246	281	405	674	1195	945	434	257
14.85852	1041	767	786	1248	717	335	1202	307	636
15.07406	592	762	130	232	936	643	871	6	495

16.35246	535	244	897	1044	863	987	98	1047	11
16.36473	105	1025	729	317	364	742	70	572	493
16.43852	786	559	94	674	458	990	629	1145	935
16.52499	790	717	1246	1008	961	229	623	40	1306
16.93802	505	1254	29	992	755	400	1108	1215	507
17.01409	360	960	65	1082	712	1164	780	1043	1288
17.09045	1305	817	511	480	1001	1115	689	1254	1095
17.25695	206	207	180	344	1009	852	261	942	878
17.68602	449	454	707	343	1128	1055	1222	775	291
18.00394	1032	597	419	943	1165	973	997	1114	1026
18.61358	799	1174	910	68	370	244	1038	475	365
18.7515	264	760	430	742	173	340	1034	917	892
19.03001	1101	1139	1296	992	716	114	335	886	526
19.25543	495	1281	793	372	753	374	895	621	886
19.66998	218	852	1024	1193	1308	978	1293	777	325
19.82925	710	348	82	621	904	524	713	123	817
20.44783	57	287	464	639	704	496	613	642	712
20.64268	1198	1282	579	1056	640	650	689	1225	1028
22.0049	187	445	739	527	65	446	1045	402	763
22.05269	947	596	103	1169	602	1171	114	828	526
22.86263	747	1082	434	392	1219	147	1037	607	715
24.17789	1018	1127	299	967	1067	194	1246	770	15
24.22977	685	227	398	131	353	49	1225	449	643
24.24708	583	585	470	1289	466	444	319	1227	1125
25.10869	789	278	833	374	890	487	1240	1077	509
25.5222	905	791	868	125	469	287	818	510	124
25.75853	1254	709	123	816	977	1065	798	1148	308
26.052	1121	669	796	1076	762	1154	454	429	735
26.1813	179	733	366	1168	861	467	296	1267	1265
26.64764	543	828	966	108	743	321	375	337	898
26.91191	322	859	790	1163	758	295	1157	737	187
27.97222	250	962	921	267	1005	949	844	497	505
28.36664	44	1199	886	151	928	527	645	354	494
28.82615	123	301	365	438	857	117	600	208	635
29.82657	824	125	65	279	477	1077	1120	498	626
30.03438	178	97	648	187	507	547	378	864	77
30.24343	127	1189	454	964	381	626	254	637	1196
30.3905	984	602	774	931	831	265	415	626	686
30.729	839	836	241	809	518	631	649	155	208
30.9422	884	82	198	19	621	1008	719	79	310
31.22126	706	1190	901	1101	905	469	932	1045	1247
31.41573	934	761	1089	1195	523	114	471	828	922
33.50978	113	1140	1278	27	49	820	624	1071	245
33.53269	949	981	477	455	812	950	376	48	196
33.76248	1090	1208	553	970	1262	724	331	523	1297
35.81919	1293	690	395	1192	1267	102	1202	1196	928
35.84344	469	328	1104	138	1018	407	361	250	91
37.0739	633	298	875	679	860	1182	467	218	246
37.29916	874	171	109	1179	253	63	560	1169	1266
37.77856	881	798	843	213	302	532	188	617	889
37.80394	849	322	702	384	32	701	560	566	462
37.88015	200	897	394	1058	593	99	261	568	1187
38.05849	712	615	593	1180	495	257	710	634	279
38.28885	136	1000	1094	499	548	1259	1207	989	350
38.83095	19	730	265	582	696	628	603	944	424
38.88292	11	825	989	1012	142	291	1301	262	706

39.40585	400	911	11	81	802	583	722	1224	929
39.8285	1130	10	1043	55	1134	1117	628	1140	1014
39.93476	1096	518	135	685	777	693	1037	36	348
40.17472	964	421	601	498	921	450	1102	245	151
41.17415	484	103	171	394	695	851	359	553	1122
42.95266	1099	743	516	1177	652	584	1079	1179	925
43.98133	113	227	316	832	803	951	1167	442	89
44.47427	230	1117	2	370	263	42	950	389	940
46.49294	837	1266	434	598	1119	225	22	661	207
47.65424	821	476	77	724	6	1037	472	694	155
47.93282	642	856	132	444	35	814	140	214	136
48.55681	713	1007	870	487	101	285	544	1166	595
49.1559	355	444	1282	517	483	974	337	945	99
49.47369	665	358	1021	19	53	335	677	1224	1265
50.21115	1084	211	1292	1168	1158	615	692	1299	206
50.21115	1094	899	621	576	152	244	425	376	30
50.53465	1171	281	767	1288	358	1198	206	631	259
50.53465	491	262	573	483	261	340	612	741	76
51.1541	1238	813	369	846	603	863	86	1213	777
52.14511	357	1038	959	1252	1106	1162	1144	840	789
52.44553	1217	256	72	82	1068	27	83	915	674
53.01692	727	494	218	283	846	649	547	15	1024
53.21982	456	141	1101	861	939	993	933	705	515
53.69579	892	1007	493	649	618	569	180	1088	689
54.58914	1257	563	150	739	248	600	1085	805	951
55.60014	1183	971	871	746	139	770	446	977	152
55.70561	890	1278	2	801	728	335	1296	1174	1077
56.30643	478	171	68	334	1104	700	168	984	1170
56.73376	565	421	303	705	51	830	129	1283	585
57.23572	511	1117	61	314	438	1038	419	75	756
58.32377	866	1141	224	812	1007	525	733	46	645
58.43352	1105	338	934	743	553	804	154	1271	222
58.8742	259	1081	143	939	147	643	1118	55	256
60.10028	912	93	975	1187	268	771	1165	1264	684
60.58905	1272	256	611	1189	224	222	166	1052	1218
61.99817	796	763	744	1151	716	956	601	70	74
62.19065	84	982	847	1164	7	1279	1088	1089	104
62.88766	810	833	533	1100	163	323	85	400	228
63.00445	1181	401	551	969	498	328	518	239	1289
63.51262	1241	268	773	911	207	292	894	1261	1024
64.77789	1308	19	664	398	384	308	199	203	13
64.93749	469	888	985	581	861	944	662	516	158
66.46992	495	1130	482	1289	791	97	1139	1135	1129
67.32944	155	335	727	444	579	17	288	133	354
67.74193	630	187	354	570	1042	379	68	376	756
68.7822	243	903	64	270	890	918	98	318	1281
69.11784	752	273	725	588	308	1038	959	837	536
70.09026	644	567	596	88	837	1275	77	947	760
70.17534	414	408	1283	1118	199	1236	722	923	259
72.89893	220	1245	92	546	184	340	792	897	1118
73.16274	1211	1246	1201	860	771	1186	325	506	593
73.64842	592	1023	422	1006	882	600	323	327	1153
73.91444	745	695	975	644	753	747	935	432	610
74.04774	626	1009	1100	982	1272	942	385	282	890
77.44425	329	393	824	1115	172	339	266	1274	772
78.18491	1228	963	553	659	867	780	1138	684	83

78.51074	719	442	800	267	1299	685	739	855	1230
80.20383	260	917	1193	1254	684	623	715	1281	605
81.78158	510	895	228	936	956	662	1083	654	952
82.26458	187	1124	423	68	646	1006	809	790	927
84.51601	564	877	530	639	1267	427	133	1041	1229
85.66011	1062	912	594	1074	971	661	1229	392	395
88.29248	632	1009	562	647	387	721	173	681	1223
89.32195	3	865	683	702	411	893	1175	780	716
91.30428	618	1009	223	535	279	644	793	210	851
98.23968	466	1031	32	374	862	1226	757	53	617
98.46292	1025	62	1237	96	287	667	744	492	284
99.75431	1057	374	443	1242	1061	795	782	1232	775
102.0893	451	1299	1214	386	217	1223	598	1075	1103
103.3017	308	953	317	396	646	492	890	516	1309
105.2297	225	84	1157	418	468	136	1099	786	921
105.7603	804	1056	85	40	308	1110	718	1115	578
105.8194	864	921	697	531	798	401	105	363	1003
106.5305	769	1145	431	1153	139	270	809	743	179
109.0486	1232	434	538	1160	287	95	299	472	335
111.6126	395	96	666	92	1289	1223	77	127	970
113.7221	945	389	406	617	1211	1272	589	497	65
117.8403	619	49	244	171	1292	463	518	64	778
118.2261	808	1095	348	704	245	217	644	129	112
118.9359	516	503	636	588	955	647	899	927	55
119.1301	112	941	709	894	86	312	1301	98	988
119.1301	47	1162	1060	609	185	1053	511	262	676
119.714	839	873	899	334	282	145	190	621	586
119.8441	1092	407	349	857	937	485	926	841	313
121.4132	1131	495	148	1	550	190	950	513	296
123.8629	610	526	404	400	457	1101	134	19	343
123.9296	1004	23	415	178	923	861	696	556	955
125.2025	384	431	506	1218	1214	517	1296	339	739
126.7567	1047	582	580	689	793	1057	913	941	603
127.3688	369	1015	184	1074	966	843	545	980	345
128.3253	1192	414	469	737	650	1126	741	1268	915
128.531	468	1100	1042	262	847	7	624	391	1087
128.7369	294	418	688	1087	434	112	254	606	609
128.9431	697	411	617	371	971	582	493	33	1165
130.2547	1017	748	172	307	854	857	209	426	250
130.6015	633	664	881	796	1091	641	1218	1245	64
132.6266	181	601	317	953	224	930	1185	622	657
133.7539	587	678	1211	548	1060	1077	246	429	771
135.1019	958	237	432	1224	680	523	661	1177	949
135.6011	1237	958	328	399	205	527	977	807	69
135.6725	370	956	619	694	182	130	404	428	846
138.8428	1093	1132	959	1308	339	392	409	535	818
139.6439	1013	1115	1023	406	205	121	1069	414	690
140.3018	857	507	790	930	1165	1078	13	213	205
140.3018	500	1056	593	135	1174	708	651	804	1062
141.5509	1022	1055	957	580	104	618	539	180	910
141.7721	931	843	202	160	1132	1170	697	654	449
141.9937	800	1287	63	1241	621	322	101	305	64
146.7047	140	1105	172	721	486	599	937	251	1298
147.9952	189	111	605	904	675	1271	121	708	610
150.7759	481	654	516	1045	239	1104	496	900	1291
153.5713	666	1122	775	888	820	765	795	96	1097

156.404	463	1069	1144	1229	730	332	1083	627	920
157.1975	559	825	819	733	93	54	203	846	878
159.194	77	587	1254	32	1247	98	802	1085	701
159.4348	1134	702	641	707	1032	346	88	1219	479
164.7981	260	1084	176	819	217	179	627	600	637
166.6968	237	478	481	680	1207	88	734	306	94
168.5274	803	531	880	340	762	1250	895	15	347
170.4562	251	900	1041	954	604	1000	538	49	477
171.2996	878	189	1097	474	819	681	419	249	167
174.4455	82	838	504	553	1242	156	802	50	472
176.4211	48	1174	1205	469	389	436	501	716	1065
176.6799	930	58	663	1184	689	94	818	548	498
177.1983	493	1087	1180	278	908	742	1130	310	323
181.4711	1097	529	72	517	623	1195	252	280	544
184.6547	195	80	1278	259	148	333	447	517	345
186.0817	720	415	1254	51	964	646	351	913	891
187.067	858	1025	664	464	583	79	32	942	925
187.606	609	125	476	308	28	259	81	1244	432
188.7772	1234	240	176	163	257	589	634	458	143
189.0482	55	916	618	443	536	1043	871	1155	981
191.1343	461	662	1065	349	916	892	258	562	243
191.2253	1175	883	405	925	715	745	1277	793	438
192.5945	310	1000	61	989	417	47	649	203	777
194.7986	352	403	512	302	684	1207	89	59	1105
195.3522	234	343	596	1019	627	381	1011	1073	674
198.6954	388	1031	1250	112	136	752	1046	372	939
199.0692	701	762	60	733	803	479	469	1198	970
200.1932	885	240	374	1171	1200	754	289	191	1087
200.8507	42	1187	481	281	422	500	625	45	1257
201.7925	110	952	1020	613	813	1080	1172	747	165
203.1158	544	761	836	651	405	352	960	1297	207
203.9694	267	142	955	781	1175	902	572	47	1191
205.4926	233	985	1158	521	374	878	1048	251	454
206.4482	321	260	29	425	576	572	703	87	1148
209.139	131	825	547	847	851	92	122	13	2
209.3321	1061	485	580	547	1194	1142	235	1244	1079
210.4927	495	419	405	1112	209	459	114	560	111
211.4629	288	1283	847	1245	843	880	885	833	470
211.7546	1051	508	1298	322	781	965	548	425	1091
212.3386	1120	642	1123	777	594	1233	1122	527	85
212.5334	909	405	1112	315	481	1009	646	665	1209
213.7051	1249	742	1194	140	1120	508	338	605	408
216.5531	1010	371	352	614	242	1158	560	674	942
217.8373	996	1069	374	192	371	894	1278	98	1118
221.5178	1233	951	1079	1269	938	1058	883	575	550
222.2183	352	48	954	577	539	359	156	529	770
222.4187	929	464	1064	650	848	567	87	143	1092
227.3621	1025	679	378	780	110	559	288	700	158
229.2962	69	903	1273	140	768	930	893	1074	839
229.5004	418	600	85	979	1293	772	886	440	1250
230.5228	1292	436	465	814	1028	816	734	1234	519
234.3285	931	903	1068	446	872	474	404	796	65
244.3687	1300	95	415	1068	989	896	272	628	111
244.7922	281	327	1065	132	709	637	640	630	162
245.322	1154	752	241	438	1188	569	355	95	338
245.6402	141	1272	952	673	539	849	1077	120	658

246.5962	78	24	226	905	1067	1123	833	11	891
248.1941	795	917	191	1193	574	1051	602	902	107
248.8348	322	611	831	1244	1065	96	792	959	967
249.1554	342	876	814	385	715	677	84	246	230
251.0841	1105	980	1267	33	965	204	476	625	740
253.1285	58	20	544	1122	84	588	844	1279	144
254.4242	498	1291	646	493	326	1200	1106	276	344
256.3742	515	328	443	454	921	334	583	446	439
260.2972	883	387	771	258	907	1202	535	169	627
260.8445	953	674	441	894	317	254	122	791	98
263.9197	510	205	890	636	244	107	404	1277	569
266.6805	1010	429	95	800	636	877	1244	924	1092
269.5665	1263	30	1210	554	666	598	405	119	1203
270.346	939	932	577	260	952	1089	620	760	783
270.346	742	615	294	932	455	206	492	1015	618
270.4575	1022	556	1256	496	56	416	376	768	135
271.3499	688	573	520	881	56	814	170	1255	1166
272.9148	944	1132	404	853	532	214	586	954	302
275.3825	657	406	843	470	563	797	635	370	1016
277.4091	763	1102	380	860	614	1211	489	289	206
280.2349	1166	294	1084	176	1209	163	1056	278	456
280.2349	948	412	839	72	477	824	1006	897	54
285.5811	633	128	167	770	867	854	514	610	494
285.9239	643	1076	501	1255	1067	785	534	1187	45
286.7242	1259	423	859	968	567	808	1024	333	302
288.213	1101	660	1214	853	858	739	417	486	690
288.4423	831	936	305	933	420	867	139	40	1024
288.557	1014	742	950	112	661	911	1192	874	1267
289.3604	260	116	418	157	626	1039	828	993	287
290.0497	604	227	476	217	1153	738	902	1171	1042
292.0064	826	1074	664	623	1150	204	910	71	1078
295.009	654	227	75	994	315	415	974	923	897
298.2558	1187	1030	608	270	445	727	410	330	1272
299.0696	977	227	711	819	933	44	1197	1084	23
299.0696	23	513	1277	311	377	190	627	1175	482
299.7678	629	529	316	1302	628	999	1251	150	991
300.6996	883	593	764	282	1251	977	1106	385	1261
301.0493	21	1077	233	732	315	1082	674	663	113
301.0493	21	1077	233	732	315	1082	675	663	113
301.9825	1014	390	947	607	709	1198	383	975	1228
303.0336	535	360	492	514	1088	1072	1061	816	1039
306.6639	110	1056	245	868	1048	298	1018	576	374
307.3682	1008	832	155	307	1158	431	1211	27	118
307.6031	301	456	21	688	578	1022	959	20	1003
317.1639	279	863	1167	647	1099	237	1026	746	346
318.8253	177	615	75	844	110	164	859	749	1236
319.0628	1185	1020	521	401	540	686	559	354	849
320.3701	725	1162	488	362	858	497	1121	204	389
320.7269	1013	387	234	642	568	818	759	508	1169
320.9648	611	1145	76	700	370	287	1054	921	1208
323.8233	945	132	603	397	1002	796	675	483	1140
326.8078	496	783	1087	827	801	1006	863	212	217
331.2364	820	373	65	768	1050	632	537	199	1110
339.7683	928	549	548	1180	1045	444	1070	288	723
341.8167	272	203	336	919	276	970	56	1138	710
344.1082	665	1009	1024	17	523	915	842	1130	72

344.2289	380	1133	196	176	564	527	375	230	1275
346.1602	914	715	577	638	324	1263	490	380	1015
348.5762	775	758	1037	1083	997	9	1279	778	84
350.3893	1123	209	537	22	917	958	1012	495	72
353.1711	131	919	980	806	161	328	1243	262	1055
353.776	1252	1003	412	590	654	1068	371	402	1301
358.8597	755	1124	879	1017	560	758	1062	775	803
364.4297	780	1223	1140	1069	91	63	1080	315	791
364.4297	547	251	182	571	722	142	71	976	19
366.488	902	1188	1024	385	244	146	908	612	377
370.2403	1260	1128	326	510	1028	1004	1096	1258	211
376.2864	994	165	33	556	1193	400	610	1165	799
377.3736	754	662	667	908	811	776	422	271	1214
380.2707	1206	1146	758	646	708	246	380	231	1049
383.0437	245	907	409	701	1196	778	110	564	1185
383.6461	283	675	215	1028	885	1139	247	1227	50
383.6461	194	34	508	715	684	917	356	974	989
385.452	263	217	939	393	1279	1090	485	276	893
386.0535	1277	140	471	314	1202	826	410	1038	538
387.6167	497	125	442	160	252	172	175	386	1031
389.2983	375	6	707	65	980	733	391	101	1233
389.7785	894	787	796	672	550	220	1008	905	1099
392.057	1092	1125	990	247	849	120	290	565	783
392.2966	934	942	254	263	540	84	117	146	285
393.8531	226	261	1248	1251	708	508	1153	1061	315
394.4512	668	664	781	886	447	225	376	496	379
403.86	1035	659	638	1031	226	402	1147	319	711
410.0025	265	732	144	876	985	435	1299	1004	164
411.5309	1125	745	1274	586	117	407	837	1077	380
414.4612	625	1102	926	838	463	302	850	315	368
415.0458	555	409	1067	513	817	926	697	1190	199
415.1626	38	1136	1056	97	119	150	865	1083	212
416.7966	735	1144	955	1152	431	746	547	1187	68
417.2627	426	1206	1007	600	1143	283	126	454	266
418.0775	14	962	241	942	819	229	598	732	897
418.7751	853	1288	584	576	388	132	966	803	805
419.9361	897	1236	817	254	499	605	135	500	150
423.6361	1265	911	20	180	455	89	115	601	1214
424.0969	1179	1290	973	621	430	53	852	700	386
426.8536	669	589	275	518	732	387	560	177	1296
428.9117	276	425	579	608	577	513	691	730	681
432.2095	1085	663	899	219	106	547	682	223	1194
437.5042	304	885	287	1235	974	859	444	662	1026
441.1823	177	590	415	209	286	633	497	1177	820
442.9532	428	967	618	1117	729	301	647	179	734
444.0558	250	953	451	1071	452	895	963	1236	1021
444.7157	851	753	284	1034	79	254	546	1207	221
446.3603	462	971	1030	599	1041	474	1075	101	1062
449.4093	715	481	782	377	697	751	426	148	696
449.7344	1220	696	759	621	276	95	917	897	153
449.9509	748	970	284	1186	63	864	902	1079	247
450.8155	820	363	813	504	755	1192	565	248	497
454.2501	293	933	638	1145	830	433	1277	174	865
457.4346	1159	204	1033	819	741	484	746	1246	150
458.0673	458	138	265	636	564	1272	701	316	1287
458.6984	964	728	881	136	75	1052	1165	299	227

458.8035	1174	184	1304	318	711	1247	883	63	760
460.4789	282	998	318	532	515	734	886	270	1078
461.1044	438	856	1178	383	1176	106	594	392	439
464.1066	1266	843	158	647	1211	1023	1050	528	97
466.5637	1069	1005	726	561	891	935	634	1273	313
466.5637	504	970	327	935	1048	1186	414	667	502
466.9708	53	1094	939	536	847	573	1075	670	495
469.1967	88	660	333	988	400	832	1106	994	1033
470.5017	1166	1083	568	1014	1082	466	1147	600	244
470.5017	1173	594	668	971	906	990	187	362	411
472.1964	734	997	118	942	124	949	1292	1148	1193
472.8903	1053	286	290	301	244	691	858	444	154
476.0322	198	85	722	179	584	1270	629	477	926
478.452	491	1206	637	503	396	70	1139	1066	285
480.4602	497	1227	421	206	600	1131	455	340	1004
482.5399	1096	1284	416	713	606	465	191	804	229
483.4768	523	600	849	825	543	148	1085	310	803
487.3537	365	1145	1015	554	1154	1083	371	176	1181
487.4449	1110	450	699	15	768	824	690	190	392
492.0196	48	1225	833	614	692	409	203	220	977
495.2461	415	639	1025	1064	1243	422	563	82	1057
496.1045	674	758	559	704	197	1013	1231	25	621
498.5604	1260	292	531	229	434	491	1157	313	467
501.1299	921	516	503	659	145	1040	461	398	332
503.1594	1024	13	490	648	117	583	273	1042	134
504.0401	448	1085	1012	939	179	184	941	1128	108
504.438	530	973	513	79	1155	405	1023	725	507
507.0244	464	927	939	725	1272	744	1014	242	1113
507.2562	922	477	1253	704	321	957	532	217	367
507.4103	363	919	133	772	960	918	155	1175	966
507.7946	971	795	1139	628	944	1277	833	181	635
509.992	96	1218	504	896	730	535	412	454	471
511.5493	268	1081	1211	1307	680	175	1039	358	306
513.0779	1306	272	841	318	774	1062	461	70	1161
517.7605	786	1128	117	994	301	165	873	539	1208
530.0456	569	26	514	951	1062	1187	947	87	1135
531.8398	284	661	1177	1090	137	191	782	323	553
532.9087	1005	1081	1298	498	643	1247	223	316	1272
533.0589	1191	912	116	59	942	1206	500	381	793
533.752	1090	360	582	1175	551	1056	962	2	288
533.801	115	982	1270	459	882	813	976	141	254
536.1195	714	82	924	920	461	734	1112	58	925
536.8862	410	577	538	596	746	1008	253	1169	506
537.1523	111	1246	468	1191	1109	516	791	958	908
539.6819	76	498	827	1284	798	1253	655	390	1114
540.9733	186	915	994	770	885	1189	1224	1002	573
541.722	443	1256	952	218	1303	1219	1007	342	389
543.8175	533	977	87	269	67	1305	432	1137	22
545.3356	127	10	1146	566	1132	482	938	1124	98
552.0253	1124	1214	558	1055	519	532	550	1144	38
552.8318	556	621	267	369	128	281	1247	821	314
552.8816	1058	473	964	1247	420	712	1017	908	37
553.7683	446	838	90	463	696	43	774	937	561

clomipran	cyclobenz	desiprami	dilazep	fendiline	fluoxetine	fluphenaz	fluvoxami	haloperid	imipramin
124	189	230	62	201	142	89	31	56	800
54	288	63	538	46	79	330	401	422	113
127	219	144	552	69	130	5	183	252	418
1275	1229	1096	1121	1237	784	927	1016	1204	1191
1083	885	1177	1151	1041	1038	1088	1143	1174	880
1174	1185	920	854	1222	836	875	917	939	1264
1152	1026	713	955	1078	930	1308	1226	1187	1209
276	226	124	1245	283	154	532	192	42	470
345	151	491	302	219	29	541	118	173	365
3	369	18	11	10	471	486	498	391	270
163	105	48	474	475	252	349	80	73	188
87	140	3	877	268	51	463	148	512	97
1202	983	776	922	1161	1037	1122	1278	1166	993
1103	1119	1231	607	1176	970	1240	426	946	1173
772	1202	834	50	869	1017	764	893	1247	1267
152	289	191	781	188	272	958	1191	841	175
1223	728	1127	935	1279	591	885	1289	1293	1011
686	279	4	166	57	345	366	421	214	349
16	1077	126	260	34	558	101	410	139	422
1040	800	706	709	883	1011	1097	945	810	1118
1222	1106	1127	128	1244	961	751	899	809	1150
80	66	30	691	998	192	184	206	45	101
208	652	149	21	177	43	387	662	23	907
21	755	56	741	11	13	96	55	650	625
121	631	258	431	38	362	720	503	163	131
1045	687	746	782	984	689	955	853	615	826
284	462	161	987	137	120	816	705	357	597
1178	852	819	991	1127	1084	796	373	722	969
1163	707	941	1073	1243	913	846	827	1172	985
231	127	154	1159	65	172	1089	587	835	68
1264	933	976	121	1309	874	981	1054	630	1140
58	150	196	434	157	41	118	674	336	640
599	524	206	323	40	138	522	732	160	288
747	617	869	514	768	1194	1158	775	291	1016
204	260	121	406	420	283	916	455	450	19
1266	1041	908	1189	500	890	566	858	832	849
1252	1067	1298	882	843	1009	1150	1163	378	1249
1289	574	934	860	1076	559	1123	1306	678	1218
883	878	1101	1185	1242	1010	526	1247	704	680
1210	1262	1103	827	1006	1231	1291	595	843	1254
1222	641	891	871	1056	1255	663	936	1297	467
1031	1204	1280	875	1193	895	964	779	926	1183
831	800	653	661	887	1130	1162	1299	1251	621
31	248	1163	190	48	268	59	676	294	588
28	378	260	349	517	352	147	233	3	1246
1301	906	1130	935	560	540	1161	1254	999	1034
94	680	33	708	197	404	245	811	430	46
82	197	338	107	333	591	34	1024	295	112
250	137	122	521	399	692	82	554	256	598
26	61	585	329	43	152	268	547	330	891
1156	1040	935	742	906	735	900	892	618	1071
1302	246	829	1072	682	1127	1103	534	571	1225
1171	833	804	91	987	791	483	573	1144	1173
1308	756	1209	1190	1126	1261	1219	1066	1287	990
203	675	474	238	312	828	107	166	65	161

1043	412	140	402	568	404	535	50	45	102
1205	906	1026	728	117	726	710	1233	685	1147
403	328	338	421	158	23	1080	657	304	722
560	206	341	349	597	48	267	347	161	592
1074	1062	1117	248	1220	1112	914	159	1032	665
772	1178	1281	619	1107	1060	1209	1040	936	977
88	301	122	860	478	314	1090	572	453	143
124	134	399	292	266	398	908	200	827	27
752	886	264	130	1222	885	904	1262	810	605
920	155	1133	917	180	943	912	1269	1117	702
20	312	236	1206	61	934	113	103	387	259
1267	1082	643	269	967	699	1260	742	941	424
1260	895	932	1262	1221	1219	1068	1096	1173	211
90	548	31	427	1088	302	552	115	19	518
584	466	41	104	425	633	199	525	113	927
1095	307	1295	790	971	812	1258	603	1089	942
329	71	224	1164	1254	417	137	66	80	477
1233	747	1246	1001	1197	245	979	931	938	507
886	1154	739	102	1049	1048	660	959	797	609
1176	1069	874	1229	779	1003	1180	695	527	1077
198	738	58	223	555	186	233	510	409	26
172	337	123	1150	791	618	454	156	87	473
1137	199	98	1164	1291	1057	1305	1135	1077	1140
372	1233	1281	1098	969	722	1193	910	976	940
655	1169	1205	518	335	641	921	1055	1135	1148
562	793	339	180	479	438	8	420	199	688
213	543	46	598	768	430	64	729	426	383
1218	1201	504	489	1176	1268	1263	1171	1141	1194
1025	1225	763	1224	274	571	964	1187	911	1064
180	605	86	42	296	186	692	35	123	927
258	41	1197	350	253	703	278	141	78	316
727	947	883	439	1281	811	273	198	308	535
1268	1282	878	684	1241	644	1178	982	544	753
200	641	24	415	285	855	186	483	38	163
432	598	1276	1185	444	101	859	705	1170	1280
940	1209	724	1083	198	869	836	743	961	1126
1282	1204	1028	1183	902	507	1112	920	1184	517
397	639	4	354	722	402	1157	930	65	414
1111	1037	353	505	1025	1157	1231	914	1160	964
629	872	594	76	424	1069	112	535	18	493
933	1284	766	784	897	1255	1005	285	1196	455
1300	834	442	1023	1303	700	45	1103	798	111
21	71	96	1061	289	239	1115	386	236	440
1011	759	556	235	686	1088	1105	329	1141	1151
418	238	46	132	662	696	278	151	484	65
701	776	1265	1199	919	1279	430	1307	975	461
1199	773	643	823	1150	1094	782	290	644	927
244	484	535	1269	57	882	380	566	32	1179
20	671	764	1066	583	786	685	741	53	562
596	608	1192	1258	703	1297	1298	655	928	1251
1140	902	223	1275	932	1277	760	795	689	1198
428	841	517	107	420	681	1154	20	61	349
1235	1284	799	1079	1027	438	1060	345	1101	875
1136	977	1307	1193	457	708	1031	1161	727	581
1298	480	605	1103	1265	1270	1068	8	856	573
690	498	889	1066	291	229	770	174	997	37

137	888	261	996	304	825	189	876	441	1040
849	1192	609	476	737	985	1274	980	939	1011
292	373	8	483	276	580	632	537	437	413
700	1190	1123	332	1024	1075	1072	911	783	973
86	843	74	109	472	1101	336	1216	164	436
569	1158	1148	1163	596	905	1272	1089	1211	1117
429	583	577	23	436	663	492	1060	851	802
689	284	28	731	199	1264	781	115	493	287
643	75	107	1006	1057	572	264	556	139	268
451	578	168	923	441	564	694	586	385	904
696	841	764	845	726	348	334	811	303	806
1145	85	10	914	827	315	606	110	118	388
20	348	898	112	664	873	363	603	177	588
31	255	664	95	202	238	504	381	568	765
990	1216	939	903	391	1235	653	560	1045	995
877	925	765	356	1195	395	991	858	1141	1245
215	801	293	849	627	180	777	842	98	241
683	1064	1048	816	940	1095	895	252	918	271
741	313	49	636	675	127	665	158	550	452
1298	1028	1155	714	450	770	1082	1070	790	839
920	1170	1169	764	935	677	1266	1026	1276	1086
1187	623	919	188	1101	1252	755	632	596	751
1038	1044	1065	1195	1141	1054	1262	1255	1092	712
819	1035	305	1127	727	562	1267	1083	1118	1285
824	158	1171	1130	244	1264	1007	979	1237	150
1092	651	980	1225	1031	1219	861	1204	730	382
105	655	165	446	826	412	338	266	330	8
459	953	1207	754	993	1167	1018	211	1165	1028
802	1306	1269	987	866	115	873	1148	855	1227
1044	744	901	741	594	651	1195	259	1104	983
1035	861	130	1173	675	363	919	1283	601	520
1064	522	365	1025	911	987	840	817	872	470
758	1299	931	862	943	725	378	1003	1033	572
635	1194	879	374	803	886	1073	437	872	626
1185	1006	809	84	1306	818	857	323	976	1128
531	955	173	796	405	1122	1022	179	947	617
1016	1191	502	1275	531	140	1156	68	1130	805
1156	632	433	147	1232	477	802	1266	1161	458
673	676	630	322	127	236	517	747	47	1309
1200	813	769	807	591	928	1064	719	385	1049
65	365	618	800	793	307	154	1037	131	412
444	411	336	481	1055	1181	437	916	14	95
9	1171	827	783	66	642	616	1101	205	96
53	413	249	706	274	486	197	276	393	1010
480	1047	1266	642	695	1236	1138	345	1090	1115
27	247	1094	738	773	946	14	1239	2	599
406	743	350	966	436	191	417	514	964	341
401	633	344	551	72	128	414	954	119	467
995	43	783	629	800	28	770	271	303	500
537	649	701	965	1181	964	632	385	596	564
190	107	1082	315	1153	570	187	332	988	482
146	1287	103	1011	1025	1282	954	1299	649	896
19	1305	1076	1148	760	471	792	261	306	108
13	899	510	351	259	530	5	516	73	1298
1215	1220	1016	851	608	103	1243	758	899	1057
285	339	102	213	43	21	1159	806	422	549

538	15	212	664	43	737	1083	1284	124	172
527	634	412	421	685	467	705	76	189	653
162	577	1078	1000	994	1208	704	934	356	1147
850	1129	1157	506	1123	886	1183	107	904	1127
765	865	494	634	1052	601	1255	419	877	1090
890	1026	1055	850	549	391	402	1064	598	526
962	930	301	532	1078	871	579	1163	1111	588
103	85	89	154	671	698	227	1127	86	186
1057	1059	350	368	243	1278	380	2	1098	174
603	697	1074	530	636	799	441	751	680	1004
538	322	539	434	323	720	679	152	310	517
1249	1226	969	684	817	962	1244	1007	452	672
1200	1273	857	536	660	743	955	650	596	1028
444	849	1240	671	816	1209	1072	217	1088	1062
1207	1038	371	1247	936	10	1128	895	494	983
78	141	334	696	198	711	900	521	330	162
1079	532	714	528	662	265	456	553	875	588
1045	205	1158	722	1116	556	658	1016	655	802
228	405	501	535	96	982	72	208	192	283
184	463	283	1218	358	1225	58	1131	28	264
601	250	157	791	349	731	724	934	931	185
1054	1284	857	650	946	1162	1095	232	795	1252
870	59	1113	299	16	736	860	466	364	464
817	531	963	518	1288	1022	501	549	798	589
796	1039	982	938	681	1249	525	632	479	537
678	848	396	271	552	639	167	448	277	974
1252	744	996	773	1090	478	599	1060	780	862
623	1251	822	1221	658	204	1166	118	1104	1228
1184	1010	864	441	1066	993	677	302	1071	926
279	647	151	708	781	484	1165	29	219	303
1257	1108	651	1221	792	904	1209	1130	1194	1096
75	454	81	809	598	245	248	1136	356	166
1049	1063	1164	578	189	482	575	760	654	397
497	207	943	408	1138	1028	1038	645	678	549
624	815	656	1062	315	992	18	1048	429	423
191	145	810	69	1035	1295	454	313	209	240
597	1113	950	631	906	1155	955	328	663	883
209	132	58	691	471	832	516	530	151	880
1003	832	401	1070	212	710	667	448	237	149
220	1025	912	383	737	700	835	36	496	89
1296	728	271	1107	931	700	242	1214	1135	1114
766	1104	1154	1042	359	980	953	787	206	1026
161	704	24	1181	1110	1285	77	458	18	312
23	1073	1009	976	192	464	553	637	506	167
649	951	689	217	584	929	1305	1163	936	865
1212	561	1284	464	802	911	6	173	769	530
1044	721	256	447	76	16	1195	396	568	1216
195	118	8	1013	558	874	530	777	356	669
1019	866	510	892	802	343	363	1122	1142	934
387	37	247	546	1168	156	116	950	244	41
121	780	312	2	1085	214	364	899	501	62
169	483	830	312	1114	254	1066	567	68	937
300	85	447	349	985	351	244	721	817	54
834	327	805	565	647	187	98	1245	88	447
650	411	978	487	813	301	887	587	448	615
1153	1024	895	631	987	433	983	917	1062	1185

1125	1251	1078	760	535	679	944	921	1165	1098
831	833	148	776	205	291	1208	1103	451	559
559	314	695	522	1069	193	771	332	758	563
419	1175	33	903	162	339	177	647	296	180
320	822	798	281	445	906	221	265	299	413
1218	787	149	1129	1187	776	1039	202	1091	1053
1015	670	1251	498	44	531	272	847	663	980
327	527	62	1023	232	1002	313	682	697	1182
718	966	14	177	98	179	347	667	796	788
581	246	53	779	1039	834	49	651	455	962
310	553	585	1300	709	641	72	1032	332	712
1187	1266	1189	367	220	694	1078	879	621	882
558	905	425	396	24	147	698	604	256	339
115	607	450	566	1089	988	419	600	482	389
673	491	1023	941	1119	912	502	1003	887	1115
674	1084	708	669	506	1262	955	582	135	453
1	239	902	108	787	871	309	172	96	77
892	787	149	439	770	953	536	832	277	1082
1172	1163	955	1195	860	578	1009	1267	1159	485
924	856	318	1271	911	517	1270	1179	941	1216
218	196	345	1282	387	747	1089	638	1134	893
1094	14	392	611	1044	1063	930	23	912	575
670	973	992	239	319	1258	164	983	1016	879
484	701	865	386	1033	1136	693	992	260	351
582	713	701	382	281	1063	278	610	1042	626
1144	1138	984	283	651	116	1157	1143	857	986
428	156	365	730	194	350	1123	711	464	154
164	182	1036	128	1243	328	170	419	820	124
1068	1035	1144	260	1169	1153	1148	496	758	536
334	245	127	1179	1229	623	647	634	34	810
365	771	803	433	646	94	912	460	844	450
1305	1172	235	1292	625	1102	579	1123	865	730
1233	295	1046	1209	321	1024	234	1111	153	974
1169	1306	1272	191	619	1086	664	1156	649	942
519	295	714	220	388	1072	363	97	296	855
587	530	605	417	384	523	1142	373	102	114
440	278	743	1	893	23	142	24	751	852
130	1290	291	802	617	1179	630	227	515	365
3	988	363	504	670	101	423	1201	160	368
694	181	808	1243	652	622	1096	636	673	220
1059	159	509	791	94	922	516	1221	1132	749
818	1107	655	409	689	348	666	312	28	329
1279	1076	615	774	450	875	307	1251	527	1157
1082	631	723	91	257	543	1096	173	703	1003
679	778	208	1209	223	1253	367	598	752	1112
239	177	26	522	635	328	300	608	791	505
637	95	484	1282	910	383	532	866	634	581
841	459	431	1091	477	486	930	455	689	389
336	641	152	913	310	576	128	1102	761	253
1152	825	1243	429	1229	665	604	543	775	443
785	468	1119	1007	1256	308	948	246	1145	367
594	898	932	632	1045	222	1298	502	505	676
155	717	547	738	1220	149	375	794	114	168
642	1062	215	352	1123	1186	881	980	615	428
228	154	212	1199	843	1248	96	880	540	698
1247	1119	934	428	1011	87	939	485	623	1245

766	989	817	1264	1171	778	1235	1224	959	751
944	1177	1135	171	833	919	186	375	1069	828
200	712	277	568	1082	832	525	736	17	720
974	694	771	424	145	198	457	682	174	447
1088	13	347	1068	674	731	734	1263	662	861
670	590	445	857	1084	524	1018	922	482	404
566	758	996	376	1220	603	568	868	119	991
35	856	405	922	571	634	481	1110	403	1174
59	1228	342	313	214	1134	942	246	39	414
87	159	477	984	313	1168	497	184	756	161
727	458	41	1139	124	1031	519	95	936	854
1047	711	1143	211	424	541	7	419	581	1170
807	332	631	1173	601	610	1218	866	762	1293
256	1225	1045	412	985	1287	926	96	637	500
930	819	1101	629	184	103	471	654	139	1118
118	995	1229	717	591	190	76	387	1018	1281
973	714	1001	135	586	668	161	738	382	225
986	1289	747	434	375	1077	681	1096	1082	373
350	388	48	37	506	1234	710	874	148	97
329	417	227	1047	17	840	512	1066	766	525
235	773	973	195	794	771	511	1145	183	264
120	882	402	334	140	1101	366	273	167	109
28	467	420	855	61	674	850	433	1289	489
46	564	272	717	718	43	57	1109	406	1049
1111	1038	702	399	877	550	1127	1070	597	1192
595	311	125	393	817	321	497	304	588	685
216	95	235	682	1282	464	630	547	70	146
1242	1030	343	362	638	698	818	1118	1112	1197
82	978	108	34	1119	1270	404	392	255	698
120	1108	820	1058	416	611	956	972	551	569
236	742	596	427	957	340	60	775	686	110
27	148	1293	1066	209	752	668	1091	377	187
728	850	1150	733	622	565	1086	215	1217	1172
1299	169	1240	875	905	254	16	888	1067	51
195	1234	295	234	610	1114	41	543	726	416
1306	600	497	472	1015	25	716	430	69	24
1298	613	371	510	904	652	1255	1288	1042	57
443	91	1283	171	1260	164	457	137	977	821
874	753	10	173	224	385	1095	236	867	427
1281	984	758	1059	524	663	329	795	631	394
889	822	624	584	1229	111	804	211	347	1253
133	367	325	875	1157	559	451	132	141	170
656	271	1077	1084	748	1113	206	803	1039	404
1184	801	486	227	497	139	609	417	253	314
1192	1018	1109	489	788	435	684	367	566	582
513	640	797	157	1114	289	1265	925	973	1126
437	444	505	1196	968	887	824	92	268	102
1101	784	271	901	484	516	597	355	224	143
28	200	363	91	192	919	740	52	230	358
16	966	442	820	384	475	545	1268	5	1057
708	1073	434	1255	271	964	226	1160	238	469
344	1028	221	1284	1200	188	72	1133	514	201
203	1223	1005	664	951	1264	630	271	581	653
181	108	815	658	42	836	565	960	120	765
1056	1238	815	391	1252	569	648	257	908	847
631	1244	740	22	975	856	138	722	422	392

1031	964	1260	1258	1180	169	1105	926	453	768
1028	1059	749	521	511	498	697	97	390	1060
469	1131	429	526	515	307	588	83	404	285
1085	402	942	409	1211	312	38	294	440	332
465	422	777	751	1073	679	216	170	665	906
402	486	986	239	1001	338	1259	419	163	482
683	1174	91	114	324	936	869	1305	122	628
243	901	1262	1105	1023	316	262	1197	1112	428
589	351	1211	471	772	720	125	305	817	868
765	830	679	496	229	583	648	1265	270	884
925	1066	851	1195	1004	526	235	1078	581	890
1079	1088	522	1245	538	342	622	525	389	1164
495	709	41	896	681	565	136	386	175	346
508	455	101	738	1063	231	243	795	57	402
1133	305	17	598	401	1258	1093	547	827	648
855	1267	306	92	989	1247	229	722	333	409
769	540	1084	249	1019	1016	678	447	560	905
1141	621	483	406	974	909	846	189	162	637
1103	180	1274	39	759	980	851	617	189	1010
1089	455	1105	1109	673	95	185	785	679	251
15	30	962	839	546	1056	275	276	105	441
815	869	97	588	945	443	348	1070	503	1164
578	98	1197	162	355	87	278	1143	70	1238
177	724	704	1219	273	516	8	142	850	284
214	320	947	913	993	56	750	819	1006	834
516	873	290	359	579	587	343	767	541	631
532	700	382	957	1079	280	303	85	814	1044
20	818	1182	831	868	130	210	930	986	1101
88	1175	777	411	866	1147	674	272	552	530
856	458	36	571	1276	718	239	640	114	298
1185	251	292	1131	1151	509	254	1290	420	829
704	247	398	81	655	481	28	127	159	1185
974	604	333	405	1054	228	932	542	90	924
445	577	424	655	1053	712	183	447	795	1134
755	1003	732	567	770	244	894	308	1153	385
76	474	923	215	409	43	1020	379	1065	1257
407	472	711	336	378	1248	628	1188	914	100
1131	1171	486	567	887	247	755	1146	297	301
1061	633	742	256	952	985	869	865	997	42
35	810	1208	1295	116	1069	778	1028	1166	561
37	269	1282	928	1159	1001	1019	873	305	76
531	350	296	864	553	573	1259	665	161	61
1215	1160	333	1096	1193	897	179	718	623	965
264	1213	1238	87	11	1119	444	1175	803	793
278	159	914	1142	460	1025	887	1290	2	603
130	1048	183	930	67	648	971	393	493	471
853	859	1085	344	341	1234	388	607	1111	676
654	1079	62	414	675	1270	1303	78	1217	1033
885	1191	1093	701	90	877	6	173	16	446
1121	1093	755	798	546	1197	791	596	462	850
1255	721	670	62	1093	161	291	264	528	1054
1112	927	502	1193	134	378	1215	79	138	1007
1126	1085	41	553	402	901	1105	526	86	286
215	843	113	1258	782	286	165	294	547	657
459	1125	1162	1196	829	753	1060	349	927	1293
116	428	1227	672	422	722	304	628	682	1214

962	381	1084	1024	1062	707	390	952	801	1105
1208	75	132	1097	1005	874	1199	1055	187	1092
1089	624	483	103	1216	1202	598	323	995	270
1017	225	57	143	1031	476	72	1058	510	1282
793	775	784	252	1268	539	549	852	211	532
973	337	597	1124	195	303	628	308	858	154
81	1129	1109	42	1094	1161	434	789	919	1043
699	599	593	1224	194	1245	649	869	491	1221
123	1216	807	81	765	945	836	1140	1186	693
532	1208	118	566	711	1223	1282	145	1149	1125
740	405	1278	393	953	1001	550	691	749	578
957	206	1280	111	256	1188	1215	54	1223	834
438	431	757	832	1090	418	272	1028	514	317
835	1142	486	1246	1028	596	665	1180	595	352
620	1297	500	33	96	1228	331	236	837	1191
562	532	1309	648	621	323	397	1052	993	637
1017	833	146	1071	713	235	336	1263	226	674
286	933	1211	76	293	314	1296	633	430	601
1240	1268	244	432	1271	48	873	132	1161	124
186	1210	474	908	794	639	491	181	1264	949
806	271	458	873	184	1276	369	858	477	1029
21	707	117	1212	1197	1164	233	941	249	1241
1006	1118	142	20	33	738	333	1276	549	371
177	1104	887	616	1267	251	511	680	273	735
223	696	517	578	539	835	1184	793	674	137
362	102	245	223	377	159	1033	980	810	855
1050	704	898	892	1076	373	389	1085	619	958
138	177	1243	291	385	544	626	873	538	952
244	320	835	586	706	731	252	563	284	288
1017	647	123	737	518	467	338	51	259	1209
219	629	878	323	947	1098	918	149	424	673
637	861	1142	1169	230	694	826	193	417	144
135	678	845	355	959	91	41	282	1232	231
860	370	683	362	1229	260	875	526	451	1301
711	1068	832	58	800	982	399	180	32	433
345	639	211	500	1222	1283	1129	403	745	641
10	925	956	342	778	1090	581	466	609	364
443	737	824	815	1135	459	874	609	428	511
443	737	824	815	1135	459	874	609	428	511
610	53	923	275	898	726	785	466	424	151
446	37	862	246	90	1032	786	526	150	136
938	409	46	1117	1301	340	811	671	1095	433
965	1245	660	32	1142	1205	489	3	230	1255
257	1174	534	1179	324	553	262	82	784	680
280	356	33	1107	277	273	1073	1091	673	1196
369	963	856	1153	732	1068	873	973	668	665
407	158	873	49	259	1208	246	432	696	116
1160	1280	336	1095	1009	1265	1027	68	1231	534
512	676	893	1114	630	592	1098	1182	1028	968
502	1168	662	555	558	990	911	858	666	1073
459	358	883	493	1194	947	169	962	621	698
968	1160	379	465	1214	771	244	346	133	1267
957	593	1288	416	299	754	739	533	672	1006
107	332	1259	138	1084	966	726	1224	643	490
13	885	673	1135	877	780	83	1079	719	741
960	539	257	314	307	1289	460	652	210	280

239	1293	875	559	392	1180	698	65	656	732
268	1149	568	882	739	956	29	912	985	526
498	1086	768	451	734	620	1298	412	662	520
474	137	65	232	77	617	239	745	1081	585
1056	1282	1110	265	783	479	1220	707	1109	1265
11	180	556	1223	559	378	907	1050	915	364
792	580	1021	156	353	690	311	1098	854	668
786	40	828	973	692	980	1217	505	684	331
561	460	765	540	1043	820	940	438	824	269
231	970	856	555	485	832	973	761	849	587
123	1160	954	483	159	280	262	474	803	945
740	258	599	908	135	241	360	776	588	580
949	301	340	332	1303	119	78	1170	401	29
19	1179	663	272	660	1016	838	864	1215	473
108	715	924	142	455	541	1284	551	484	435
27	715	529	84	1276	1114	59	138	775	484
173	2	959	1108	642	1109	428	594	890	247
496	432	648	1212	19	1129	842	1071	450	749
353	886	986	712	345	16	1117	125	272	218
1106	551	1191	312	883	1186	34	1066	90	284
944	493	947	772	609	968	852	554	1250	342
544	1150	1030	502	373	478	841	122	216	877
780	376	49	465	1239	478	543	1005	199	358
229	1223	589	120	681	601	791	771	211	1095
186	968	1175	449	926	735	101	1137	361	349
1086	503	371	1145	704	1105	320	89	925	574
793	186	317	395	925	1283	526	192	538	720
573	811	621	598	1304	1044	626	961	448	330
1019	480	601	892	86	703	1034	978	1028	403
880	1294	1083	901	533	1291	1207	246	715	960
207	170	978	169	1247	1087	357	922	370	209
973	247	631	999	493	1106	1100	1200	406	1058
697	1122	876	692	357	863	822	440	848	394
947	1008	401	896	713	756	1029	1028	654	1083
1085	1294	840	482	790	1089	486	826	801	545
1121	1197	1071	31	450	1070	308	793	437	1122
554	1254	1115	1174	208	664	658	647	9	489
997	336	763	628	1264	584	636	714	426	454
547	618	395	305	1186	1240	661	1265	47	403
841	208	868	1169	219	1275	1235	327	1302	405
939	700	462	922	803	1241	225	278	5	59
340	384	720	929	141	549	322	861	114	990
606	1140	418	332	671	209	1183	102	367	992
682	1252	336	341	1149	919	1273	565	575	878
1137	854	721	1308	751	1075	110	934	304	921
540	1211	442	978	1230	1184	534	113	341	565
1137	1223	1077	689	82	621	1293	545	912	688
99	1268	182	1116	769	125	260	1270	479	1040
197	1126	1084	145	620	523	1026	602	193	903
1170	1124	554	82	615	484	420	1166	978	684
980	1006	931	25	1304	166	402	1085	495	1010
990	535	163	850	954	612	921	637	885	107
749	252	118	570	1164	19	394	452	323	1006
748	1135	888	692	818	778	478	145	572	453
35	537	592	955	1024	755	644	524	279	40
383	634	871	1114	1265	1160	618	589	157	477

315	110	298	878	341	751	603	740	1000	1134
598	428	879	1054	172	80	92	698	130	307
479	781	1100	999	307	413	901	1139	722	448
320	1209	1135	787	604	243	301	488	253	339
676	542	34	716	1280	202	599	378	1125	739
215	776	302	1115	1121	978	180	948	492	119
424	243	747	373	1261	713	448	1050	866	403
401	529	877	112	698	94	1017	606	641	678
785	1010	1108	661	148	668	298	191	1119	570
272	598	359	42	1171	666	144	887	1162	486
342	1177	776	677	11	1082	405	838	72	487
1268	410	116	65	1105	876	337	760	743	362
171	366	1218	1019	815	1264	695	570	698	401
99	967	22	631	804	1087	1230	1030	826	570
250	1010	1212	1123	689	537	486	570	109	596
950	366	790	285	1262	1144	340	577	131	1282
457	1161	1100	599	86	1241	798	1201	435	122
1103	1148	512	759	830	39	1279	379	742	152
1024	1245	246	242	605	1230	524	1001	736	1051
755	861	78	1301	151	302	925	852	626	575
542	1182	309	1268	18	60	770	884	15	721
860	1186	316	423	326	1259	592	813	151	290
977	841	528	387	189	1303	120	681	411	942
99	870	919	448	348	1150	849	241	714	84
935	632	784	205	1155	527	638	125	390	1021
272	858	1260	213	607	704	1204	522	481	490
1230	950	933	274	1209	26	651	87	1064	330
101	1078	34	1159	343	174	253	1129	395	586
1213	479	576	801	971	650	114	186	257	630
1218	388	448	495	1298	1172	958	1026	94	1285
872	672	288	555	1096	70	644	330	757	1283
248	345	236	1114	386	1151	522	65	864	163
699	114	32	473	1049	129	1055	809	657	513
184	950	1301	399	627	705	317	899	572	104
532	1022	570	731	205	897	938	718	380	1094
737	448	114	1207	746	730	1220	981	832	201
212	106	1245	602	887	821	863	784	459	428
524	167	281	1092	1163	763	983	183	1123	74
208	569	1306	200	526	579	109	654	1276	819
759	891	470	869	165	1060	1089	1113	267	293
226	609	954	1178	753	253	300	852	354	1196
947	1164	308	1063	938	1096	220	63	12	304
254	694	216	1163	565	846	389	371	878	1104
762	1291	984	60	236	137	463	639	405	448
1290	562	696	5	1264	745	1099	265	614	151
625	1087	155	416	1126	237	840	1134	565	308
746	204	770	1037	1188	146	1030	972	819	255
508	143	739	766	166	1149	1154	457	674	706
176	700	868	322	1254	826	545	1008	203	452
375	653	399	855	1302	712	113	312	951	388
192	372	802	752	1224	242	733	37	154	1104
734	1096	250	673	86	1277	97	550	69	620
642	745	321	282	894	173	1091	1031	580	1016

loperamid	maprotilir	nortriptyli	paroxetin	perhexilin	perphena:	pimozide	prometha	raloxifene	suloctidil
68	45	25	445	185	196	292	511	103	710
157	257	57	126	232	71	39	83	128	47
37	94	74	483	120	202	77	1059	104	1121
1075	672	1301	783	1299	1261	1235	1148	916	1300
1191	1227	1288	594	953	1254	1213	705	1263	1203
863	1077	1272	651	1041	1187	1137	1213	1195	1291
1306	1091	1259	1046	1066	1194	1242	654	745	1205
339	8	457	419	197	244	190	914	36	444
234	634	53	431	250	83	95	527	11	521
395	213	49	1226	162	42	62	184	251	135
52	64	12	245	98	4	663	66	81	125
317	92	10	394	364	18	518	349	34	68
828	746	1276	636	947	1144	1096	1017	738	1243
1150	1266	1284	845	1226	1222	1169	774	963	1297
1108	1233	1246	793	953	1099	1157	983	1049	1259
297	23	174	91	13	90	657	116	111	328
1288	952	1189	720	1264	1243	1091	1038	1265	1297
171	324	411	312	163	289	373	422	176	1090
288	103	15	437	372	463	321	249	17	105
907	1158	1196	701	956	1170	1012	558	915	1145
1279	1200	1114	822	1281	1254	1206	932	1040	1297
207	74	2	473	547	14	527	61	287	213
111	176	105	237	397	50	6	981	114	877
147	12	95	207	178	35	2	479	5	227
112	77	158	31	54	226	2	292	84	235
1140	543	1164	995	569	634	986	1117	1136	1091
169	276	117	272	11	290	177	289	279	146
1201	1268	1240	724	1303	1089	1253	575	1259	1298
176	515	1097	581	893	1128	1257	1135	1230	1157
649	116	141	66	196	1	1222	51	151	208
1274	1299	1226	293	1195	1042	1244	151	1231	1270
622	45	433	102	291	61	100	518	77	256
813	346	8	466	180	108	210	420	101	137
981	1173	908	953	1268	803	1281	1047	482	1211
1179	48	84	1141	840	168	584	315	633	551
911	1061	1233	1047	1207	809	1007	215	1260	1203
858	1260	1051	469	249	865	1059	824	935	943
1162	1233	1074	1102	440	1036	609	994	1079	1035
1303	914	1296	445	1174	1216	741	283	1004	1069
845	328	1280	1091	216	948	1035	1079	1142	1180
1307	680	923	896	1264	1242	774	857	1226	1002
1200	1217	1285	395	680	1274	1226	168	1161	1268
1129	1020	654	1014	279	1267	1200	275	1143	839
217	8	169	222	556	342	1140	297	441	733
504	748	141	1160	457	114	103	140	1083	60
1079	1146	782	1053	274	1264	1118	1182	705	1300
609	17	18	533	424	34	979	483	170	209
180	294	442	39	829	232	212	711	115	461
248	467	1128	1181	64	89	646	27	705	592
197	108	258	49	495	126	436	634	996	162
1102	960	1192	135	1287	1093	1246	1126	636	991
320	1275	1283	171	728	1245	1178	1277	1116	422
1140	1030	1205	601	1239	821	1251	1195	237	1304
1307	958	868	761	170	1299	1235	673	603	1268
367	1242	365	318	241	621	681	549	145	100

166	177	6	256	226	52	167	325	89	757
1063	136	1115	69	1072	1044	817	1098	702	1162
446	320	24	513	333	10	14	472	204	1196
184	305	86	673	141	66	344	68	193	498
1246	1285	919	388	1045	1076	933	182	951	1302
975	1063	1232	818	972	868	1277	704	1057	1264
380	23	521	193	174	83	628	289	184	362
55	541	222	1175	96	168	992	572	245	333
1124	1146	1053	801	1303	332	1285	1268	1092	1308
1055	1166	1200	530	643	1252	862	925	1004	1152
820	563	70	441	984	376	323	1069	202	474
1037	1201	1089	988	1220	1293	1031	1139	313	1135
1271	1277	1028	755	510	337	1268	826	433	824
199	702	52	613	700	120	434	89	465	608
851	382	73	540	34	155	92	526	57	999
1153	441	776	1259	255	1302	673	987	753	632
117	1019	18	760	623	30	357	178	409	132
1281	944	1214	1107	1301	1308	924	690	1123	1290
1114	232	1208	770	1233	1275	991	955	295	1297
919	1258	68	935	133	973	759	392	785	1202
421	239	53	605	974	45	139	935	101	466
1	98	36	192	230	295	248	565	316	980
863	1225	1033	924	1015	1288	1264	232	1000	1220
530	678	991	418	742	953	609	979	885	453
369	1088	1262	457	521	1001	1076	1002	735	492
286	430	212	659	800	87	213	1160	291	916
32	312	291	635	562	447	188	294	24	554
323	324	1100	986	743	916	1236	658	678	1275
798	936	1109	177	55	1049	1232	1018	453	281
51	4	32	1068	15	18	427	971	39	47
288	824	471	179	429	501	212	100	697	545
1236	875	1305	792	1292	1182	1293	588	1234	1205
789	1149	1100	1145	979	1274	1216	903	553	1184
53	174	549	367	434	775	374	327	762	229
383	408	1124	989	662	1169	299	1210	230	607
1148	967	1012	381	905	916	753	1092	265	383
759	936	1253	1216	992	650	1047	935	734	909
250	316	10	83	673	30	725	859	210	697
1149	1201	1114	309	1030	1174	1129	1267	885	1199
365	57	869	904	510	111	610	8	559	372
1058	954	1207	983	901	908	1163	145	1188	907
788	677	724	803	695	951	1282	1307	115	1305
107	40	461	93	216	823	614	715	394	525
400	255	968	466	1147	969	1038	493	1064	1235
857	192	83	150	261	200	554	357	39	525
757	1169	1118	1081	1144	722	980	1163	178	1248
1214	1219	1089	746	1306	1179	1079	500	1269	1302
29	749	420	378	104	129	584	146	136	116
570	484	779	630	389	121	251	1061	403	159
1081	549	1024	133	687	929	1201	489	220	923
1185	900	1063	781	395	1235	879	1119	533	1181
119	766	145	62	267	671	691	114	1080	163
618	838	906	198	1171	822	1178	573	262	459
662	660	888	626	573	1012	391	1190	114	1127
1063	1290	1268	435	1170	737	1198	1222	555	989
868	195	476	186	663	564	329	722	606	597

323	55	284	250	307	25	1120	1	799	157
585	905	827	202	497	1135	793	1263	1009	878
333	303	201	120	149	482	375	357	137	65
917	1117	913	412	1271	1270	1018	743	1211	841
201	34	62	127	410	137	809	1197	26	835
307	1046	1209	422	445	1206	946	1075	411	706
1082	384	35	38	419	948	106	788	136	706
487	302	411	1222	221	154	398	205	329	585
997	30	228	619	19	61	785	650	978	521
670	139	1202	500	279	322	493	668	529	527
847	982	763	297	751	1074	1068	1021	955	840
397	218	955	420	616	759	445	71	117	884
18	284	231	629	635	600	876	1131	375	349
101	15	119	981	248	1111	449	1040	435	1070
696	226	1059	251	1084	541	1099	1261	166	1161
195	986	1253	1009	884	784	595	1134	488	759
70	581	44	510	773	318	29	374	219	535
685	1273	903	818	1099	1281	1196	737	1275	679
83	91	250	560	272	149	144	530	40	797
715	1088	832	68	481	1099	1176	1264	669	1257
561	412	1107	659	538	519	1177	264	872	1293
1076	677	1286	893	1193	1304	916	428	1255	1166
547	877	1293	350	259	1287	1157	1128	802	958
846	169	1172	664	495	1055	1225	1047	670	1223
1089	1031	364	746	500	770	952	1198	330	1302
474	498	1182	336	332	1036	885	1235	227	1109
120	11	902	25	376	70	842	993	28	603
1164	1242	1248	1191	258	543	839	376	1228	1118
607	1232	637	945	89	689	1153	815	1107	234
652	216	644	220	730	807	1178	1072	1294	162
1216	1232	1229	780	1190	1206	938	1122	848	879
1286	644	1198	193	1176	1234	1208	792	1134	1230
766	1135	1270	289	1275	1202	700	799	1300	1024
550	1151	1004	224	1296	1153	1195	724	1005	1113
1115	430	665	713	1061	827	276	794	1233	1023
1	749	58	240	172	484	296	491	421	298
1228	887	699	711	159	768	1254	1247	1274	1160
944	849	1133	1256	1120	571	1111	1216	1005	808
168	690	167	264	164	969	53	321	811	83
703	1177	547	351	754	1145	734	855	627	982
617	761	414	1024	264	816	12	34	789	9
652	257	244	86	397	764	544	81	548	206
877	347	365	1111	143	622	1027	296	316	912
1059	714	74	909	82	391	338	435	365	863
1107	1203	1221	1239	410	519	1014	438	1263	1102
156	196	381	870	464	177	250	696	10	280
23	68	744	349	614	58	326	476	91	134
21	556	82	760	1154	182	200	456	842	690
41	172	317	642	146	620	384	313	493	1183
708	1133	1166	247	1010	902	969	1043	795	955
251	1109	164	1250	367	229	1150	591	153	74
290	786	1247	1035	497	793	863	1226	310	1285
291	153	149	326	111	841	164	357	87	464
19	332	408	701	680	10	280	122	692	860
297	1005	1096	188	517	1277	1134	308	1026	1126
250	955	132	1253	635	296	335	66	766	506

190	183	203	1	857	46	86	414	323	590
1098	782	86	855	111	973	25	1	300	90
1287	874	1126	1020	976	589	890	428	787	821
710	1288	1286	409	1273	1296	1090	442	749	604
922	580	1042	612	914	520	1212	121	705	1304
1301	940	1004	383	1252	1253	741	795	1103	905
1253	245	1150	5	628	1226	1081	1108	623	1293
1161	1171	44	1283	447	827	472	763	890	329
72	141	168	247	898	691	252	276	42	1036
7	1142	379	1152	200	608	1135	426	552	1272
312	913	384	342	1107	504	565	236	613	381
561	1151	1302	101	284	889	952	680	978	1089
375	436	1075	203	649	1212	1226	1021	643	802
1216	1187	1262	1222	149	698	881	317	1265	1031
129	14	668	149	390	77	1134	1154	353	1180
931	425	887	94	1064	481	238	258	42	485
210	659	44	1000	319	396	1158	1244	478	825
1055	129	1170	632	954	1223	487	684	716	391
302	1127	777	176	847	422	133	1245	1100	358
426	417	1233	118	1256	315	278	337	378	205
913	502	312	310	611	622	765	368	191	694
311	104	776	407	57	1132	1011	1182	882	762
47	585	382	831	621	11	26	837	407	430
1222	587	1266	1161	1307	499	1306	606	1188	1029
1296	1019	756	980	870	520	626	1074	508	90
140	340	73	664	765	219	821	1219	218	295
1128	973	1160	480	217	598	786	519	344	1251
1306	1187	1186	699	887	930	1247	234	366	649
1234	714	1254	450	1295	1164	1046	597	572	1282
397	235	473	66	950	239	969	295	593	913
614	748	865	349	1033	948	408	1102	906	1244
739	124	602	814	63	152	458	276	508	178
423	206	278	933	181	279	306	172	235	795
532	654	520	244	255	323	1181	638	514	816
305	618	361	511	1036	356	114	897	30	906
414	729	251	955	364	406	722	671	471	895
1115	1190	1050	508	1165	1284	1141	658	1299	527
302	1089	171	500	445	16	401	419	916	437
108	367	706	1092	266	880	476	431	497	1113
756	481	248	703	249	799	7	868	635	705
1233	783	677	541	1059	568	468	434	343	1299
38	1156	1146	1073	194	440	946	1128	1233	211
397	669	617	739	91	150	188	509	545	179
184	107	541	111	79	146	588	1196	719	229
652	885	301	677	404	962	698	1019	1292	990
610	751	175	500	965	1211	536	1218	48	1199
63	1028	43	878	797	474	356	685	1133	438
670	215	405	87	1241	718	1217	883	701	445
830	754	1150	492	1267	1246	1245	637	1078	1065
671	452	194	96	1048	609	663	1195	830	1282
421	464	154	574	676	859	64	477	374	127
29	204	23	144	557	780	407	1149	310	841
632	113	569	758	222	775	541	904	1284	746
77	339	233	382	164	252	448	273	235	1049
1153	68	898	994	920	283	161	44	406	579
851	739	577	396	379	888	1295	1129	893	1259

1128	442	258	1240	557	687	1259	1073	886	1182
594	1053	737	1196	583	881	838	824	1128	1001
310	476	10	881	236	660	423	772	508	329
649	210	107	344	127	172	122	1287	95	759
673	1272	441	680	222	1060	489	357	820	419
207	627	1258	298	1261	426	1073	1232	491	1250
244	781	1224	1142	260	201	87	153	125	56
922	902	412	315	222	679	187	234	917	1101
611	5	238	772	97	970	905	133	189	113
415	128	349	315	474	711	598	1029	122	921
876	56	810	1199	3	392	1038	98	598	475
826	880	1195	1021	1146	452	741	1099	843	59
939	406	403	930	156	657	495	530	1030	261
823	179	307	229	213	293	87	132	506	282
49	456	83	993	1048	405	762	777	699	991
319	275	394	773	177	241	710	1304	163	990
851	253	654	1284	776	746	601	539	62	316
183	108	49	148	662	66	1168	658	103	562
839	471	445	57	895	1281	1243	1205	453	1148
1217	558	739	341	568	711	602	644	446	1079
220	1010	71	179	746	385	652	290	344	146
370	138	637	264	570	273	1256	617	209	485
53	1057	1208	429	543	1027	1136	1185	272	1179
867	831	1236	261	1259	598	1278	896	991	934
176	1011	1241	49	808	834	556	1116	588	995
737	85	964	433	384	1076	1168	1077	528	123
707	775	806	262	315	689	202	248	463	1112
583	1120	271	910	488	317	1262	335	602	83
1167	1270	482	384	1212	1166	1060	608	835	1294
1142	567	366	909	134	332	947	514	1270	489
86	402	70	453	215	681	672	592	967	403
1241	347	1004	634	342	1008	1062	776	567	1152
547	175	149	224	780	644	763	878	377	691
193	632	1190	951	879	160	748	1177	18	366
1246	34	229	277	1119	395	574	288	1177	1137
32	210	670	75	774	93	1098	1104	90	1203
693	231	4	106	178	1114	841	159	47	744
715	54	302	525	74	274	940	822	12	1170
72	46	492	440	1061	712	607	1197	687	65
938	1068	1222	359	647	1289	1069	1136	1249	1158
1093	135	349	123	1014	390	271	667	341	672
91	574	927	545	134	743	142	82	327	832
773	1179	763	1241	378	509	1228	276	300	28
814	512	392	971	135	66	519	525	790	72
851	215	1160	800	182	684	232	106	1142	763
778	1280	282	285	1039	480	160	120	790	124
561	772	325	541	43	83	1132	80	461	397
224	377	819	96	647	524	43	1088	371	893
653	105	125	747	215	438	888	418	1191	83
674	676	690	374	981	1192	777	762	978	905
1084	795	304	761	118	181	1276	403	798	990
380	577	288	526	774	532	860	1070	986	953
192	333	1009	296	667	56	330	866	336	314
1063	676	351	1169	61	1184	827	95	678	422
11	94	184	43	774	296	965	1156	588	555
927	474	1226	265	882	1036	1242	1306	336	1174

626	832	844	591	418	49	1001	162	447	1232
1127	525	1195	697	387	855	706	837	723	180
328	124	1004	56	143	577	276	646	621	610
1089	514	234	1040	462	207	96	442	36	607
283	558	991	583	497	489	449	468	629	936
521	577	390	1225	568	959	555	263	1287	337
871	1069	364	904	344	902	979	829	994	1026
838	417	776	297	594	191	656	651	581	346
101	625	986	790	301	1299	673	944	403	764
889	838	1131	743	7	216	547	563	454	424
101	787	112	473	758	154	93	445	337	172
834	458	124	659	826	1080	486	1299	612	1303
294	1289	1292	1052	462	648	753	996	374	821
806	1230	914	1076	777	668	583	79	1109	803
368	513	68	844	8	158	398	379	577	688
77	590	460	401	18	224	501	603	70	362
569	449	152	418	1116	721	433	1245	31	626
356	1114	1271	274	482	1237	569	898	546	1225
28	181	233	339	1141	968	62	1248	1098	183
624	913	1200	228	470	28	1182	765	131	702
141	580	107	87	179	547	309	385	218	1013
919	179	97	1226	757	978	1075	611	322	637
580	767	12	154	106	172	777	198	124	1068
190	395	67	1054	476	108	378	868	11	1295
980	701	352	85	405	831	1149	907	756	1100
196	724	514	1185	792	548	938	169	259	526
928	272	147	424	1231	387	924	774	1290	1283
720	468	1240	560	351	996	1235	1221	235	1195
1013	1079	358	785	784	570	139	210	113	147
505	921	248	189	354	349	1167	977	662	196
583	1011	734	853	443	383	419	1114	559	528
11	757	249	1119	328	512	274	608	734	9
516	1274	125	1090	75	159	1164	403	451	1297
512	181	1265	10	1159	28	1308	1164	616	1244
831	418	468	818	277	517	725	785	222	81
1157	241	838	614	396	668	378	175	961	775
1099	26	79	190	1266	460	1172	1280	82	976
393	1087	663	1276	1096	213	1055	504	403	75
128	158	134	506	472	248	973	1018	94	723
662	369	560	474	729	717	622	429	786	1188
435	1143	634	948	571	1136	740	1006	550	277
458	151	831	307	553	1296	1164	357	615	537
1227	530	1281	821	1297	603	1272	453	790	993
935	362	9	78	1073	25	748	950	336	1307
666	1126	1028	255	1223	516	561	554	841	499
876	266	1142	92	791	1293	1251	115	1305	903
423	1183	961	661	427	669	681	76	164	456
275	417	345	806	771	182	676	1205	84	8
329	1058	691	71	575	405	585	293	881	195
765	611	351	292	1086	243	234	206	181	52
1271	1232	1301	544	904	1079	986	229	1157	499
1065	274	57	119	697	229	1198	1215	400	506
1126	1216	863	761	906	1033	918	96	1244	931
957	170	451	466	47	756	152	984	476	377
1269	970	946	363	800	1000	972	328	1042	527
684	414	439	834	986	750	593	671	143	958

587	500	542	446	351	772	1220	414	967	902
493	146	126	987	225	744	732	1184	343	215
758	654	355	957	1132	211	66	187	969	1225
837	28	153	41	134	214	1248	118	457	1265
311	415	330	1171	43	1026	568	174	699	318
4	169	656	1190	24	510	889	904	674	1024
417	197	1185	66	693	24	373	1309	249	331
938	1191	1160	645	1295	1065	806	466	386	273
932	1293	1111	1294	425	9	880	576	231	1014
295	204	1089	91	644	670	677	1174	606	596
947	655	1077	931	63	594	1046	410	1072	1062
825	558	845	142	802	833	1082	73	952	1075
183	53	85	642	257	89	594	532	1057	973
1115	1295	1018	559	675	993	543	213	539	870
1151	187	459	560	220	1156	898	997	1088	349
579	148	1037	381	324	29	590	900	112	1178
467	792	55	344	723	715	641	1121	992	1295
450	670	1128	1273	298	167	460	431	1110	447
521	19	42	776	308	723	954	468	1093	210
1096	1228	921	795	1225	897	928	484	259	512
172	539	966	1028	527	1201	1030	739	908	81
1104	632	412	663	419	570	993	491	14	1144
118	791	792	289	684	1064	848	871	460	55
963	762	272	622	1052	839	1183	292	1005	2
1202	816	737	684	773	960	1223	337	429	1037
128	1078	630	1177	437	570	1117	637	221	15
685	823	1021	551	384	724	521	421	187	1108
1016	315	597	1163	53	608	1043	312	978	896
666	1014	415	490	555	636	181	35	1033	753
898	723	460	693	984	131	493	554	297	595
1222	785	513	453	321	320	754	244	199	1143
309	696	717	679	640	183	448	19	1029	281
639	1089	370	367	909	434	318	944	311	11
1039	632	51	1149	759	313	966	985	697	928
800	1090	721	445	383	452	1065	125	1068	875
699	390	837	905	399	654	741	746	939	830
755	208	1152	1023	474	376	931	1238	28	610
324	329	223	1027	541	1200	756	464	155	742
212	367	628	547	886	1296	1264	924	1106	307
434	192	155	341	389	172	1092	768	220	1128
915	1124	394	1279	651	350	751	953	959	591
270	920	30	76	845	747	1119	459	949	986
1169	1150	1066	1054	606	827	886	187	1065	322
388	346	1192	83	297	101	59	1249	24	22
521	1298	53	548	761	782	698	854	796	290
72	877	269	581	633	760	403	910	561	1190
909	1152	189	167	907	669	640	872	289	299
607	998	888	328	980	490	351	418	866	768
951	480	19	708	624	382	40	386	756	325
406	337	869	339	1014	480	364	816	229	696
977	1051	409	421	1270	682	648	1279	276	1296
83	21	819	461	31	93	1299	1227	1046	966
545	577	79	734	525	400	1173	539	105	993
118	834	972	227	1254	646	754	868	23	688
369	466	1260	222	1150	680	572	1226	1095	1279
91	259	714	807	67	35	730	36	940	1219

852	1051	209	1079	518	443	978	296	244	340
760	382	1058	732	744	347	1119	1304	418	1263
312	950	225	715	264	51	696	412	1279	1200
916	1294	1077	1285	251	1032	937	117	59	556
641	569	50	122	305	568	463	567	701	1138
147	312	652	1137	1014	480	1289	241	725	942
359	370	1186	650	968	1111	630	63	930	854
65	841	891	1158	1266	746	151	988	660	1247
741	1247	577	446	923	478	1268	980	842	1101
352	909	1042	226	889	601	237	641	792	722
871	1227	965	762	348	786	328	1010	940	364
1248	824	326	78	177	40	1046	506	962	516
296	164	628	9	415	1031	393	960	257	586
23	744	360	575	574	588	866	241	600	47
509	106	1134	609	1224	5	41	536	1141	251
794	1060	647	803	209	1218	337	1192	290	1047
1081	1033	1113	328	821	654	886	1242	1182	551
321	205	287	1252	1095	328	299	703	1265	578
458	1017	1302	1046	91	1053	782	1180	713	1233
946	55	416	509	842	94	422	671	545	890
722	448	1185	1060	710	58	256	145	1104	857
561	15	431	454	590	384	1189	415	437	336
407	225	941	1036	467	264	342	1062	291	700
291	245	951	1035	854	733	1145	576	594	934
476	923	79	493	19	1117	931	151	139	544
749	565	760	384	894	474	500	1204	725	1049
47	683	179	200	1001	849	1199	942	527	603
1069	624	1273	1229	677	431	551	1290	457	164
1061	1029	991	1210	1004	664	341	291	1106	518
1085	1052	813	677	1295	235	1008	114	1126	1194
261	449	601	862	481	603	1065	660	604	694
549	426	357	929	321	843	976	589	307	921
439	367	792	172	1305	693	1142	535	60	152
1214	435	450	240	709	1132	1255	1139	132	1114
746	948	149	519	182	712	207	467	318	453
1267	340	854	681	1046	357	901	43	971	1076
492	1000	95	319	569	679	941	875	684	211
1210	134	988	700	1024	536	39	145	1100	716
1210	134	988	700	1024	536	39	145	1100	716
796	519	241	956	463	806	145	1	90	110
67	260	723	410	652	841	717	1137	1025	209
447	91	1	412	1069	288	723	140	214	485
1000	110	1096	666	475	167	1285	1300	702	1221
536	902	1029	601	708	1198	378	441	593	974
691	182	875	1015	82	347	704	378	596	428
32	201	645	876	36	409	307	613	1016	273
1025	1096	1132	973	975	1004	965	461	4	16
298	597	797	722	379	448	1282	1079	653	1123
862	389	1286	92	323	1083	165	1128	400	97
520	910	239	912	376	302	1153	896	436	83
556	885	1069	1246	524	228	547	696	1001	79
328	304	65	1095	603	60	361	1289	376	565
495	1278	35	780	197	266	90	723	343	15
1056	953	46	1000	491	765	1139	1198	669	66
685	701	1167	837	534	858	983	347	994	786
373	351	1036	1128	65	642	1120	1048	69	916

707	1227	934	1024	648	731	455	152	911	792
66	434	541	239	629	139	611	1175	134	295
26	62	483	784	97	415	1018	687	667	1290
128	1276	849	809	100	1302	605	848	282	986
444	509	427	187	419	711	1212	1228	670	1034
494	457	1028	1060	1096	980	723	1254	365	154
116	57	891	720	120	206	243	1222	323	272
643	486	853	366	1235	1293	28	82	525	724
1056	904	506	778	264	1138	788	1122	490	1294
26	1144	961	84	14	1078	294	502	942	960
649	921	616	1036	379	1084	338	1024	258	226
676	1135	302	330	1304	1041	699	1249	1046	576
823	188	1053	305	1225	1092	844	1230	1099	1029
214	126	289	926	282	65	856	1027	131	378
982	861	425	32	1094	992	559	1201	744	776
1134	643	1038	940	226	810	10	261	569	1063
847	574	232	283	1213	1179	266	1184	1007	139
371	517	1187	1236	1116	896	54	395	732	781
19	1195	1275	1092	1263	271	409	1260	3	391
1264	1241	353	262	589	228	860	351	971	640
601	288	881	914	1230	553	636	628	1170	196
1047	365	297	269	958	964	303	637	1048	1020
722	74	191	260	1113	627	794	1276	1041	1096
647	1183	854	953	930	1188	816	207	492	74
29	770	1199	276	491	1104	840	561	421	402
370	1308	666	639	518	54	1169	382	1006	167
1119	883	702	294	462	561	776	718	351	615
375	361	1242	966	702	376	1109	944	28	1245
1231	1283	802	204	431	74	207	166	671	484
609	536	797	105	40	865	167	1006	239	407
253	1179	162	1015	250	362	1037	1192	540	147
87	675	1134	317	513	95	285	593	1309	818
612	241	1280	113	219	1107	996	1201	153	50
423	38	432	749	6	1126	980	193	671	430
821	430	644	158	591	852	741	1091	705	1292
773	797	1109	455	634	356	137	742	260	570
883	394	186	198	109	811	257	1151	25	252
953	1247	398	618	1142	605	390	194	1127	472
1222	448	814	615	704	860	1253	647	201	1104
920	700	1246	337	796	734	775	221	838	382
1160	1002	1278	1292	379	283	391	1225	1173	154
785	1042	440	542	1270	862	198	1144	285	490
682	407	170	769	899	429	94	664	630	921
235	1234	805	427	1056	725	747	238	1163	1286
857	988	230	793	687	467	767	232	510	1193
582	528	163	299	804	673	898	654	751	1069
479	210	1154	196	543	732	796	481	467	1098
798	1118	192	879	1113	601	184	1063	831	130
75	846	517	969	804	933	815	333	166	683
914	1022	573	184	29	818	738	785	456	618
1	515	378	193	452	1179	991	646	22	1196
181	81	486	377	970	770	1046	1234	1293	514
521	667	47	51	680	99	1013	1206	700	1291
481	115	735	421	457	391	1052	1263	220	651
1065	689	283	1247	260	1115	620	308	1152	783
883	1186	941	976	1182	422	902	587	59	507

848	844	1154	299	329	17	816	566	149	757
870	35	1267	132	37	384	738	563	1155	643
520	90	65	142	699	81	312	841	364	563
394	166	792	478	39	810	485	267	149	729
840	218	137	826	904	116	1078	244	193	1274
704	591	1180	546	217	608	225	522	115	1277
691	583	287	883	128	610	352	126	517	1276
156	1086	970	786	123	1213	276	1299	272	929
658	604	632	234	267	419	742	1239	74	897
1299	1166	181	386	631	1078	1090	366	977	542
432	387	721	1205	199	482	995	289	492	918
798	1064	892	990	823	625	93	1089	1212	347
340	164	423	207	250	45	781	770	430	924
621	260	661	426	1145	602	1124	1174	295	1002
1	1142	953	328	698	167	801	939	1261	1024
345	1251	52	24	356	994	839	450	921	1038
42	1239	620	1075	711	95	332	366	1232	937
382	507	353	1285	880	704	21	268	997	220
304	571	1031	657	553	442	782	435	203	777
828	1226	636	1222	445	335	791	232	1137	1129
466	379	390	75	828	328	602	1245	1168	189
373	609	301	365	1124	932	1270	606	200	823
473	77	1268	984	688	333	898	852	1208	934
900	994	826	1122	842	1098	474	1305	162	508
757	945	695	1096	779	739	1146	227	996	26
159	502	1182	305	745	352	828	1086	570	323
671	375	402	514	593	136	228	856	1268	931
612	912	319	643	983	635	162	1093	993	485
298	1083	111	413	534	233	1099	252	1180	266
339	79	1089	804	576	432	1308	265	595	492
1039	220	594	686	612	805	333	268	379	916
1001	935	455	1103	1027	1194	1268	253	336	840
609	683	944	466	915	1067	1280	512	520	137
1184	621	959	217	102	608	457	920	915	284
931	83	1139	99	372	607	460	1202	269	63
350	1197	733	520	484	924	1107	385	361	1106
977	321	1089	127	1061	1063	135	112	687	471
1211	721	275	1111	1004	735	277	9	462	259
1185	854	1092	629	497	321	1122	864	1093	548
775	730	675	260	52	467	1299	1225	1146	571
961	425	831	230	359	910	330	1284	495	1110
601	453	932	640	455	295	812	1191	903	95
621	227	795	1174	387	1187	70	456	1126	1202
850	1142	826	120	308	571	923	940	19	878
542	1023	673	1230	958	258	659	6	145	923
245	690	336	816	1125	1248	1103	755	53	1171
257	941	1149	318	132	163	1289	160	752	1130
807	1075	722	743	1201	41	880	178	563	498
1179	1109	345	560	967	592	939	88	1065	510
1062	163	461	289	121	167	1020	617	1077	1133
412	1168	880	1049	622	1071	975	966	496	469
412	164	996	789	481	1161	128	671	751	510
252	878	563	1128	1202	873	504	186	1068	1236

tacrine	tamoxifen	thioridazi	tomatidin	trifluopera	triflupron	trimipramine
453	271	22	754	5	282	39
238	302	43	766	45	342	260
56	321	230	88	13	43	3
889	725	1159	870	1254	741	1213
251	829	1102	761	1296	722	1199
312	817	1124	208	1262	919	582
470	277	1268	563	1123	883	916
289	100	185	1298	325	111	110
736	476	175	435	74	706	109
271	151	452	911	15	526	721
1079	531	62	518	33	646	20
590	605	76	785	20	663	24
148	881	984	518	1249	1109	924
80	1147	1120	1109	1230	504	960
1074	1052	974	866	1273	501	1149
609	126	132	686	29	663	26
1030	483	1031	455	896	771	1165
1271	1204	375	56	265	127	10
925	375	421	222	32	1227	116
279	895	1067	1058	1207	1129	1173
873	355	1021	365	1057	552	1140
1277	1095	35	1140	23	565	34
1086	363	197	563	29	905	138
554	322	373	955	210	822	353
1046	766	128	540	8	647	16
729	722	837	1184	739	1128	1005
263	1087	3	578	122	478	38
790	768	751	35	1086	1280	436
1006	1178	900	881	598	993	370
1276	515	53	532	191	1101	204
993	569	1086	405	1308	247	1239
891	124	13	1018	10	446	377
1165	172	340	601	82	1161	126
515	1155	1119	1122	1197	181	1127
841	350	166	401	54	79	102
644	654	1294	827	1298	448	557
1105	1232	374	1072	1190	617	1191
307	1179	1297	577	891	1152	1295
306	1307	930	673	1301	658	1297
185	826	1243	254	767	1236	1278
649	402	1294	510	1303	233	1270
240	503	1235	562	1227	998	1152
590	759	1252	989	1226	582	1239
1151	386	161	777	366	324	183
1119	310	177	898	117	336	173
232	1234	775	864	1295	447	1289
556	770	448	460	49	252	19
1003	621	377	1294	276	3	724
515	614	871	637	665	292	387
565	599	117	1016	465	188	145
535	418	901	1207	1264	272	770
515	616	1087	347	1250	1273	1104
105	815	996	15	991	744	1261
865	427	559	671	1105	1051	826
481	743	142	834	55	473	244

476	262	567	789	126	574	650	
84	916	1015	1287	662	1024	863	
1271	134	135	1241	64	1093	245	
1207	607	19	702	236	667	216	
1041	366	619	599	1077	1039	989	
65	799	695	139	1267	1213	1002	
817	383	65	385	44	698	19	
275	1075	158	829	94	281	100	
1287	147	1272	275	945	262	968	
1091	778	1160	158	1216	869	1249	
45	237	31	749	315	253	532	
170	647	778	217	1057	1083	1259	
779	966	364	684	216	886	878	
508	683	43	938	3	169	294	
369	734	358	1204	415	470	62	
959	877	1135	968	704	1209	814	
1193	850	272	200	144	489	10	
648	502	1141	597	1101	399	893	
1082	915	1179	711	996	771	1104	
1039	1237	1113	139	820	934	883	
939	1026	73	131	16	1091	8	
298	1002	58	1137	117	671	477	
529	1208	982	285	1200	577	608	
481	1117	1282	123	1092	1152	65	
1039	1071	937	591	1179	554	1004	
233	278	843	130	338	400	309	
682	168	78	435	51	665	236	
690	103	995	188	813	549	465	
473	802	1115	959	722	1266	699	
582	912	432	1239	61	1120	250	
929	225	405	144	1143	19	912	
1030	1213	938	464	1304	740	1057	
154	981	566	368	687	582	1150	
1053	352	34	533	213	529	171	
273	1309	1247	896	1205	1207	1267	
953	609	768	382	872	486	611	
500	813	760	338	985	456	811	
969	515	33	1252	14	502	456	
464	652	673	127	672	605	733	
169	695	266	16	198	582	210	
329	1060	1160	305	677	607	720	
1210	940	827	709	749	439	1014	
357	398	113	778	615	569	750	
1258	1042	1027	656	463	840	544	
1062	915	406	624	233	769	888	
191	655	1255	473	1233	392	637	
836	373	838	63	1031	1147	513	
428	1010	235	621	436	86	467	
880	771	522	87	613	574	47	
1150	949	849	171	963	1022	712	
97	653	853	687	982	1029	815	
327	958	156	29	418	311	850	
1278	434	1071	309	1190	1056	1222	
986	512	1149	908	649	1060	917	
75	810	612	886	653	528	817	
421	331	377	1028	66	667	82	

1105	1172	384	212	106	620	68	
413	1025	658	663	836	1030	541	
50	824	689	573	310	901	711	
823	372	1098	423	1187	1035	647	
81	1283	762	902	443	473	264	
690	959	1196	328	919	1197	1045	
736	379	118	723	424	250	215	
1176	467	557	975	161	864	422	
14	1064	443	1035	304	219	135	
99	735	38	410	383	1137	1079	
1214	611	690	145	1171	278	864	
52	262	92	54	79	67	961	
454	928	75	141	189	440	17	
576	32	418	1100	210	335	653	
831	1035	626	448	247	1208	954	
583	126	776	242	963	1143	708	
662	308	312	713	473	1132	113	
507	598	779	240	1062	924	785	
847	132	312	705	247	112	1042	
418	1039	571	923	1210	212	1294	
632	586	1088	560	902	495	827	
193	1082	1158	749	1189	980	1036	
415	1063	745	897	1165	837	1286	
989	837	441	923	390	1075	757	
1058	1165	1204	1163	987	1199	880	
829	642	781	840	1034	1102	799	
895	1103	47	159	299	736	10	
931	360	781	851	406	791	1255	
660	1022	677	449	588	1026	1131	
721	360	611	1060	148	755	1154	
379	596	1080	661	1261	351	425	
770	738	801	202	1288	34	785	
301	1146	1121	210	755	797	981	
771	757	649	300	1233	747	1015	
662	273	766	984	721	699	410	
618	381	495	783	6	994	69	
774	492	1166	682	1056	559	1209	
168	207	1279	825	601	546	1124	
48	1038	952	464	339	639	1159	
249	579	587	253	737	916	363	
51	184	336	1260	518	146	292	
37	391	123	1132	66	1188	729	
269	145	272	874	188	517	771	
521	537	467	414	278	958	724	
706	435	745	1018	146	684	1043	
281	294	22	185	159	91	511	
600	767	246	610	620	49	365	
778	476	295	759	601	681	383	
12	916	90	270	803	1094	239	
1123	1081	466	469	789	157	807	
725	842	445	846	258	266	330	
1129	539	223	175	439	1275	172	
535	644	1003	1241	202	480	1246	
845	700	244	430	364	213	751	
452	973	922	931	1031	748	287	
599	688	320	92	185	233	912	

336	918	461	1131	211	342	681	
1016	859	136	641	430	478	337	
469	1242	198	1097	1176	1306	1197	
608	746	1205	455	1024	688	462	
1021	1041	1169	536	1070	367	775	
261	1198	619	952	878	1127	575	
92	1038	1211	11	1006	124	847	
1128	1206	221	42	311	574	273	
278	426	1048	154	29	1282	863	
481	908	841	510	583	733	1130	
377	511	194	699	258	1061	1043	
433	195	1219	1057	1232	794	877	
485	617	985	530	814	829	768	
830	300	828	912	380	713	1232	
1103	1122	765	1140	460	861	886	
492	372	259	1004	390	920	580	
474	1142	604	306	309	152	752	
94	1012	1217	212	1208	273	685	
1197	943	243	788	500	252	704	
1092	295	100	794	75	893	40	
344	98	257	372	234	541	141	
185	566	735	1137	373	1080	1303	
1083	27	537	1122	765	216	155	
1012	1071	1212	87	1294	74	360	
894	634	20	1304	259	998	1288	
172	190	341	276	387	432	1209	
190	1193	1250	503	629	670	1211	
948	1014	718	364	1113	514	552	
1121	519	1034	601	634	841	622	
1026	249	57	537	7	379	114	
433	549	410	702	901	586	553	
11	923	627	456	394	231	1041	
570	52	601	1231	14	495	410	
347	737	691	1043	100	169	176	
203	451	242	102	106	63	290	
913	300	655	129	439	1034	705	
1001	533	1106	257	773	1066	612	
326	725	951	971	981	85	458	
283	654	406	187	77	172	807	
720	601	684	367	395	1093	708	
629	441	1252	383	1198	1213	667	
784	1170	243	790	597	649	358	
1197	443	301	884	136	434	59	
323	104	338	357	334	1000	686	
428	395	773	166	1030	1039	1238	
106	1294	1045	124	842	981	925	
362	518	494	168	261	867	1201	
318	715	493	531	456	264	275	
863	212	782	975	1004	197	167	
143	1073	920	91	16	292	45	
1080	968	18	1047	594	824	624	
494	153	192	658	434	303	1192	
399	1269	358	870	213	360	561	
20	1291	865	615	127	338	44	
269	801	400	844	743	721	970	
985	354	627	611	982	1003	225	

79	1101	1245	111	804	1013	57	
1094	169	699	531	863	993	1115	
733	893	953	539	1230	500	238	
818	812	1023	886	528	218	195	
623	548	566	1185	437	817	364	
102	735	746	337	502	220	449	
108	637	596	191	205	1204	1129	
522	193	447	396	506	341	628	
972	170	684	1029	520	626	710	
117	981	250	418	238	813	648	
193	169	411	560	563	1157	219	
765	476	558	1105	1204	1263	770	
1182	29	768	1066	595	508	460	
907	174	175	1296	38	927	825	
747	596	905	154	634	746	816	
656	183	331	306	551	714	970	
57	579	622	453	151	754	389	
623	767	114	1173	166	1225	75	
333	1138	786	87	242	549	573	
1055	162	471	226	1088	747	562	
420	658	425	1276	753	910	98	
71	65	782	124	680	169	247	
848	668	635	303	997	1186	122	
584	705	298	517	847	1193	1271	
1256	1161	718	116	841	768	1057	
778	788	766	910	301	779	656	
403	138	83	863	79	265	235	
487	641	517	719	98	686	549	
538	147	581	632	1163	368	1027	
435	108	247	580	360	343	968	
5	760	495	641	688	1142	292	
263	929	1061	319	753	1057	1022	
76	480	163	152	177	962	849	
945	566	1280	404	966	441	1223	
842	809	262	269	485	936	154	
28	410	235	372	641	1214	95	
377	543	383	1301	310	1105	676	
615	1010	485	586	392	597	737	
270	412	913	724	178	258	809	
1239	57	59	571	940	699	133	
1023	367	1013	183	1145	294	829	
173	519	24	499	675	1155	650	
343	620	235	220	350	1134	512	
289	693	596	436	1001	856	931	
365	658	178	443	667	563	622	
390	593	665	1125	1020	476	142	
329	199	853	114	904	773	48	
613	260	735	858	285	820	516	
50	843	69	892	133	436	806	
558	968	1021	836	1048	280	816	
335	1275	1253	48	1165	624	1106	
297	664	925	928	653	446	334	
371	613	1242	804	1079	639	399	
753	175	850	627	1218	1241	1122	
972	481	419	257	552	259	194	
253	622	890	335	1266	785	140	

364	171	239	716	308	769	1165	
420	1152	973	118	1058	1191	318	
251	1096	622	71	99	93	524	
755	344	299	1201	561	576	151	
954	318	475	1304	816	708	779	
438	572	183	962	1233	398	162	
490	534	808	176	1039	1063	1184	
1204	559	305	854	103	501	762	
125	533	378	227	297	1292	295	
447	93	1143	21	969	480	518	
776	88	953	689	706	829	457	
887	892	872	202	421	928	1180	
619	1215	187	559	1036	1189	177	
918	683	693	490	913	877	1106	
436	586	229	661	15	873	836	
531	1100	273	368	140	1227	859	
464	662	800	790	477	866	77	
31	883	758	1047	884	817	1216	
745	311	942	887	860	787	880	
1110	111	772	105	133	700	885	
479	643	358	596	939	1213	919	
849	427	114	909	360	682	1122	
523	1088	350	1054	988	1092	82	
953	593	336	1056	3	102	414	
743	240	723	350	1170	1103	93	
1131	1008	413	897	422	276	487	
112	1153	620	732	209	271	54	
228	648	946	291	1152	1046	546	
793	206	67	669	575	680	992	
24	850	712	209	440	143	1125	
167	678	1268	683	749	963	265	
1260	698	341	632	225	37	1304	
851	528	288	419	610	618	681	
574	951	1095	1252	806	173	629	
3	129	984	63	493	255	822	
654	328	449	338	722	709	694	
124	293	512	333	427	225	176	
208	225	287	767	202	816	255	
979	559	654	1106	139	744	865	
158	1102	644	512	287	962	1230	
1097	738	1147	799	219	813	1105	
110	510	197	1038	394	973	1012	
846	1198	1194	10	1305	117	377	
1258	543	325	1174	287	1112	317	
772	156	979	915	1104	1031	1019	
332	685	1233	12	632	669	1101	
570	214	511	775	286	689	442	
117	363	991	680	267	764	211	
167	89	823	1024	1172	321	553	
978	172	323	1020	149	1029	464	
1103	801	1054	876	836	102	597	
943	1292	284	365	607	1169	485	
373	393	465	949	1015	729	1001	
797	776	980	448	881	1263	1160	
504	325	969	108	718	622	416	
681	26	400	673	187	540	724	

479	146	1237	63	1294	707	323	
920	122	716	306	158	980	684	
400	543	548	391	125	1108	856	
451	607	761	1029	471	763	554	
1021	1291	529	569	651	130	975	
1004	269	937	646	65	736	701	
265	946	1026	937	138	558	314	
139	948	836	502	536	450	1243	
131	230	483	232	93	313	309	
1140	864	1096	466	235	415	262	
289	713	546	1263	240	773	1093	
747	533	163	17	870	1043	1145	
1152	828	133	807	14	514	279	
29	818	681	158	474	221	1255	
274	1000	276	1218	483	779	342	
145	480	151	1170	201	567	266	
602	683	1107	24	726	422	434	
820	570	1179	99	1010	814	572	
514	273	386	80	633	1097	1229	
204	846	314	326	855	849	1151	
173	337	684	522	373	150	1290	
1299	378	78	633	482	84	430	
387	195	349	1109	1179	1302	589	
854	344	727	559	785	424	217	
450	196	374	1283	1051	1100	863	
51	510	494	684	1048	245	934	
1229	1032	473	951	853	778	1001	
10	982	857	737	679	328	953	
196	313	363	799	282	828	990	
691	193	48	742	234	1271	203	
258	1092	1170	304	622	835	212	
72	1089	346	1266	784	1123	353	
67	875	88	688	625	113	618	
889	182	286	136	7	63	464	
12	418	916	101	778	1156	121	
1182	520	1053	515	1190	270	495	
250	1076	1176	306	653	469	1148	
955	531	898	513	1047	956	1114	
1172	441	851	1250	490	304	151	
832	387	1071	808	145	1129	140	
1044	780	375	1005	492	464	461	
321	598	232	1219	89	604	227	
934	23	979	16	555	120	720	
991	222	583	616	995	766	1130	
216	251	1226	635	1146	1231	82	
1075	754	1215	162	813	136	29	
323	860	363	858	1012	1034	1056	
389	189	427	398	58	254	132	
1131	438	402	108	63	1129	827	
1009	882	282	750	1236	18	1229	
813	889	969	338	655	415	1178	
1145	851	765	285	24	180	251	
837	1010	25	540	85	1073	311	
722	498	1015	632	1309	1221	449	
317	242	874	943	845	303	292	
746	466	57	794	1285	799	44	

92	233	143	539	31	829	717	
652	925	223	811	505	442	97	
451	1165	984	1242	1235	765	548	
205	1307	473	890	221	540	1169	
826	906	573	685	504	876	433	
1211	602	484	102	227	392	1084	
65	771	524	550	611	1267	1138	
634	1039	1115	633	748	284	240	
29	570	647	49	438	1065	797	
489	117	588	152	146	84	82	
1032	861	1108	87	1259	977	639	
331	1246	610	730	999	1083	393	
192	917	238	1054	281	1261	1061	
382	237	509	373	522	619	303	
561	461	777	220	924	1139	1275	
427	1203	1013	493	727	398	503	
151	1076	1237	411	586	1094	18	
473	348	537	737	793	657	383	
734	434	545	874	1273	1111	133	
444	480	375	1287	348	538	913	
517	6	406	446	737	132	647	
1267	321	515	978	41	361	761	
812	846	662	880	374	951	529	
201	1252	824	468	867	1201	272	
770	440	990	689	728	838	748	
1250	946	580	322	594	1078	331	
119	1067	591	231	427	1158	954	
460	71	900	579	619	848	1053	
917	433	280	997	279	50	658	
623	572	583	671	989	931	866	
242	559	799	380	956	722	200	
812	854	1211	960	1026	586	37	
859	391	132	937	1289	566	922	
1017	737	243	317	749	1268	855	
1235	555	618	162	973	1230	977	
936	1169	123	1109	168	1242	112	
431	1104	434	1089	1167	734	926	
441	1008	508	1028	669	996	1223	
441	1008	508	1028	669	996	1223	
690	1035	33	146	155	1115	257	
1122	975	88	480	687	523	625	
1169	689	603	1237	554	26	182	
509	328	8	816	4	1081	734	
938	829	409	957	316	702	243	
1094	567	197	684	35	703	67	
20	1064	168	550	683	1056	297	
298	372	299	244	630	345	1027	
104	663	838	278	529	881	346	
683	629	505	456	236	1196	838	
1002	414	441	242	1052	1003	1006	
785	738	691	926	838	212	246	
41	1040	695	775	267	240	538	
1134	732	282	1159	181	1103	708	
1068	212	13	551	788	857	1236	
90	1133	158	361	304	832	27	
877	268	383	251	236	1095	1015	

604	663	620	987	752	930	859	
506	1104	908	51	636	123	1173	
655	282	823	343	435	1266	391	
1165	1147	343	797	18	353	979	
721	157	608	297	1173	745	268	
267	580	1187	343	720	145	477	
820	902	748	1278	603	687	317	
555	1046	38	1170	537	839	834	
132	707	395	413	508	87	945	
795	287	1041	1079	306	340	786	
66	939	342	518	450	317	1077	
861	454	982	1219	1260	481	1016	
331	295	1266	427	405	177	616	
814	16	829	631	589	563	676	
1093	586	906	1296	344	1270	59	
1033	819	1174	901	971	531	47	
1024	176	72	480	497	703	19	
619	978	534	315	796	856	544	
764	867	700	429	924	418	1027	
1019	393	738	1051	1020	1190	744	
75	793	640	1014	1189	212	650	
104	427	1214	338	902	851	182	
550	669	593	522	33	642	182	
493	636	1074	426	949	1289	247	
1066	415	1237	826	866	884	1075	
1116	774	854	158	1287	1161	1009	
139	970	931	409	1159	1307	1033	
1200	751	117	847	441	974	619	
1088	1170	366	691	425	865	1091	
916	432	848	530	1076	564	964	
523	925	974	326	1097	423	664	
418	1211	536	693	1268	394	400	
1094	744	139	374	845	1175	638	
720	624	263	466	659	590	1295	
359	372	293	1014	110	357	489	
470	971	1133	726	713	1043	167	
940	1198	946	482	351	914	905	
58	965	480	1151	1097	656	112	
849	418	875	285	398	363	218	
1140	107	882	353	1252	392	746	
892	724	189	319	287	285	861	
1240	222	476	889	354	676	483	
785	1165	1126	865	929	447	716	
764	651	441	1025	1175	115	648	
299	750	259	462	58	235	970	
1281	162	249	891	302	409	515	
980	925	270	287	673	701	902	
867	629	661	298	147	60	268	
1147	378	853	1261	86	307	995	
693	292	542	1259	117	403	839	
225	294	678	794	1090	1056	91	
287	630	138	1082	569	187	116	
1062	598	488	568	236	1030	332	
276	873	1006	1007	179	1290	913	
938	36	202	544	604	1102	1165	
827	498	1161	397	833	125	547	

674	99	1227	567	1066	161	421	
9	1004	1124	462	1039	439	958	
931	1104	492	425	777	780	1189	
1161	798	64	948	246	1139	855	
1281	55	700	1014	69	534	770	
440	678	1157	560	324	769	742	
332	1296	299	256	767	1012	986	
962	299	1273	920	828	643	307	
481	73	557	356	107	1216	953	
175	361	1116	724	295	529	400	
422	935	615	650	906	527	520	
617	1020	624	869	435	648	386	
711	1170	1207	249	984	1117	136	
1205	111	143	1017	444	26	764	
809	620	796	165	990	929	1076	
435	800	1003	784	612	831	1131	
1306	722	890	801	757	138	868	
748	201	1028	291	190	1276	838	
1150	78	1290	75	255	1300	691	
199	154	138	1185	1095	238	660	
159	625	847	1095	681	243	631	
936	665	712	229	113	212	1192	
874	244	246	21	733	1110	630	
663	133	155	1081	326	934	872	
1057	1242	730	425	881	247	1076	
693	859	921	629	511	932	806	
766	1102	533	49	366	1162	410	
394	599	642	971	501	6	523	
485	1246	1223	750	1084	443	1040	
555	794	473	50	715	1291	777	
218	875	376	829	124	190	1187	
879	594	196	704	647	533	932	
287	841	603	57	374	772	885	
39	1018	1002	1159	260	1168	254	
803	759	436	760	425	932	691	
68	787	1238	103	256	285	596	
601	982	367	1226	630	873	881	
412	754	203	765	556	859	863	
278	909	473	303	852	220	1192	
268	194	489	344	960	185	523	
77	654	1180	736	445	672	5	
331	784	111	1269	546	215	1161	
1001	1085	154	19	659	151	190	
594	290	1135	567	162	978	799	
1197	1018	334	567	436	208	793	
447	131	384	864	266	91	214	
588	584	92	491	686	301	496	
1251	927	779	259	746	923	201	
762	1141	277	1034	242	520	861	
891	720	1069	759	931	231	78	
747	448	1212	931	666	1160	164	
1177	1010	1233	129	1114	482	220	
398	662	145	880	174	1148	1122	
