# Supplementary material for: Comparing structural and transcriptional drug networks reveals signatures of drug activity and toxicity in transcriptional responses
Source: NPJ Syst Biol Appl. 2017 Aug 25;3:23. doi: 10.1038/s41540-017-0022-3 (PMC5572457; doi:10.1038/s41540-017-0022-3)
Supplement: Supplementary file 9 — Supplementary Table 7 [file 41540_2017_22_MOESM9_ESM.pdf]

ID	Names	LogP	pKa	TV	TFEB EC50 (uM) (3h)	TFEB NT (24h) - 1st repl.			TFEB NT
						0.1uM	1uM	10uM	0.1uM
1	triflupromazine	4.81	9.20	0.80	8.35	-0.10	-0.26	10.78	0.51
2	chlorpromazine	4.54	9.20	0.90	11.46	-0.37	-0.49	6.82	-0.27
3	imipramine	4.28	9.20	0.85	>20	-0.15	-0.58	2.45	-0.16
4	amitriptyline	4.81	9.76	0.95	18.44	0.07	0.12	4.23	-0.74
5	thioridazine	5.47	8.93	0.70	10.25	0.08	-0.23	9.18	0.13
6	haloperidol	3.66	8.05	0.95	>20	-0.92	1.00	9.01	0.19
7	perphenazine	3.69	8.21	0.74	6.63	-0.29	0.21	17.46	-0.41
8	astemizole	5.39	8.75	0.48	4.58	-0.50	0.56	-0.24	-0.62
9	loperamide	4.77	9.41	0.82	6.58	0.44	0.57	36.42	-0.61
10	dilazep	2.88	9.54	0.76	10.25	-0.84	-0.53	17.96	0.09
11	nortriptyline	4.43	10.47	0.77	10.04	-0.63	-0.20	18.77	-1.71
12	clomipramine	4.88	9.20	0.76	>20	-0.11	0.06	6.60	-0.54
13	fendiline	5.83	10.07	0.67	>20	-0.11	0.29	17.61	-0.21
14	perhexiline	5.53	10.58	0.61	8.24	0.30	1.64	48.77	-0.42
15	clemastine	4.92	9.55	0.89	11.60	0.59	0.44	27.81	0.86
16	pimozide	5.83	8.38	0.66	>20	0.71	0.11	0.37	-0.57
17	amodiaquine	3.76	10.23	0.90	>20	1.26	0.33	0.58	-0.28
18	trifluoperazine	4.66	8.39	0.78	9.33	0.73	0.03	11.03	-0.83
19	fluphenazine	3.97	8.21	0.80	>20	0.22	0.15	16.19	-0.27
20	tacrine	2.63	8.95	0.88	>20	0.65	0.04	0.57	0.54
21	desipramine	3.90	10.02	0.74	18.07	-0.46	1.43	16.84	-0.40
22	maprotiline	4.37	10.54	0.85	11.58	0.34	3.87	46.17	-0.10
23	chlorprothixene	5.07	9.76	0.84	>20	0.50	0.92	12.94	-0.62
24	amiodarone	7.64	8.47	0.77	6.32	0.10	1.23	45.20	-0.03
25	suloctidil	5.61	9.76	0.57	>20	0.09	0.46	13.55	0.84
26	cyclobenzaprine	4.61	9.76	1.10	6.67	-0.08	1.24	16.75	-0.82
27	bromperidol	3.83	8.07	0.78	>20	0.07	0.68	8.82	0.19
28	fluoxetine	4.17	9.80	0.88	7.79	-0.35	0.91	12.33	-0.55
29	chloroquine	3.93	10.32	0.91	>20	0.33	0.27	1.46	-0.26
30	trimipramine	4.76	9.42	0.77	>20	0.28	1.18	15.35	1.05
31	paroxetine	3.15	9.77	0.91	>20	0.25	1.26	9.53	-0.29
32	raloxifene	5.69	7.95	0.91	14.28	0.99	2.09	34.48	-0.30
33	promethazine	4.29	9.05	0.82	>20	0.56	0.39	8.35	0.01
34	fluvoxamine	2.80	9.16	0.89	>20	1.14	1.05	1.09	0.32

**DMSO**							0.6719	
**SD**							0.0283	

Normalization: Xi-DMSO/σDMSO>3

(24h) - 2nd repl.		TFEB NT (24h) - 3rd repl.			MEAN TFEB 24h			LAMP-1 24h NORM			Lysotra
1uM	10uM	0.1uM	1uM	10uM	0.1uM	1uM	10uM	0.1uM	1uM	10uM	0.1uM
0.47	9.14	1.32	-1.16	2.42	0.58	-0.32	7.45	1.81	1.32	4.00	-0.15
0.40	3.22	-0.30	0.83	2.62	-0.31	0.25	4.22	0.80	2.37	3.85	2.37
-0.36	0.35	1.21	-0.05	3.86	0.30	-0.33	2.22	1.09	1.26	2.44	-0.41
-0.12	3.47	-1.28	0.42	5.81	-0.65	0.14	4.50	1.11	0.91	3.97	1.09
-0.09	4.44	1.77	-0.91	5.47	0.66	-0.41	6.36	1.49	0.81	5.02	-1.16
1.00	4.78	-0.40	1.89	0.58	-0.38	1.30	4.79	1.93	0.76	1.63	1.49
-0.31	18.75	-0.53	-0.13	17.41	-0.41	-0.08	17.87	1.41	2.38	9.26	0.81
9.18	24.89	0.33	9.52	24.88	-0.27	6.42	16.51	1.47	2.08	9.04	-0.02
0.44	46.67	-0.94	0.01	57.97	-0.37	0.34	47.02	1.00	2.32	7.57	0.14
0.62	10.77	0.17	-0.66	12.11	-0.19	-0.19	13.61	1.59	1.50	3.43	0.79
-0.71	12.34	-0.84	1.05	34.64	-1.06	0.05	21.92	1.02	1.64	5.65	-0.04
-1.37	4.78	0.16	-0.62	9.32	-0.16	-0.64	6.90	1.38	1.71	3.37	-0.57
0.41	16.02	-0.81	-0.73	19.60	-0.38	-0.01	17.74	1.29	0.58	2.98	0.95
3.03	56.29	0.25	4.84	89.21	0.04	3.17	64.76	0.06	1.15	3.72	0.30
3.56	51.69	0.69	3.43	67.60	0.72	2.48	49.03	0.65	1.06	2.95	0.45
-0.77	-0.80	0.41	0.42	0.01	0.18	-0.08	-0.14	1.08	0.23	0.93	0.70
-1.05	-0.62	-0.07	-0.32	0.51	0.31	-0.35	0.15	0.25	0.89	1.25	0.78
0.53	16.35	0.30	-0.40	15.63	0.07	0.05	14.34	0.59	0.36	5.35	2.60
-0.53	19.15	-0.10	-0.64	14.71	-0.05	-0.34	16.69	0.93	1.33	5.55	1.22
0.57	4.32	0.12	-0.83	0.37	0.44	-0.07	1.75	0.86	0.56	0.32	0.29
-0.36	4.75	-0.29	0.51	12.67	-0.39	0.53	11.42	1.21	0.76	4.40	2.22
0.89	28.63	0.26	1.20	51.13	0.17	1.99	41.97	0.06	1.28	5.69	2.26
-0.32	3.28	-0.48	0.24	4.67	-0.20	0.28	6.96	1.09	0.46	3.37	2.29
-1.04	13.87	-0.27	-0.29	0.10	-0.06	-0.04	19.72	0.35	1.29	7.11	0.77
1.78	2.85	0.51	0.40	1.15	0.48	0.88	5.85	1.47	0.51	1.60	1.09
0.28	9.99	-0.23	0.38	21.23	-0.38	0.64	15.99	0.48	0.80	5.80	3.09
0.04	7.05	-0.06	-0.03	-0.48	0.06	0.23	5.13	0.04	1.04	0.94	1.83
1.50	5.19	-0.64	-0.07	10.54	-0.51	0.78	9.35	0.54	0.43	2.52	2.74
-0.31	-0.74	0.00	-0.26	0.01	0.03	-0.10	0.25	1.26	0.78	1.60	1.22
1.77	8.63	0.66	-0.51	7.10	0.66	0.81	10.36	0.87	1.23	3.10	1.01
0.16	5.28	0.72	0.91	7.87	0.23	0.78	7.56	1.32	-0.15	3.37	2.82
-0.29	66.80	0.58	0.20	9.48	0.42	0.67	36.92	0.13	0.39	8.85	0.98
0.62	4.75	-0.34	0.06	9.50	0.08	0.36	7.53	0.39	0.17	3.53	2.94
9.14	69.13	-0.54	24.54	77.48	0.31	11.58	49.23	1.24	1.01	2.16	0.27

	0.6643			0.6892						17.596	
	0.0189			0.0184						1.0691	

cker 24h NORM		ER-PDI 24h NORM			Golgi 24h NORM			LipidTox 48h (1st repl.)			LipidTo
1uM	10uM	0.1uM	1uM	10uM	0.1uM	1uM	10uM	0.1uM	1uM	10uM	0.1uM
2.34	2.10	2.25	1.20	2.05	-0.67	-0.50	-0.90	2.24	1.22	1.68	7.16
0.40	5.78	2.60	1.82	1.29	0.12	-0.42	0.45	2.09	3.56	3.75	4.54
1.77	3.50	0.74	2.40	1.07	0.32	-0.06	0.55	1.71	1.53	4.22	8.85
-0.07	3.23	1.29	0.65	2.01	0.07	0.21	0.58	2.34	3.91	4.40	6.01
0.50	3.12	0.31	1.22	0.93	0.37	0.22	0.74	2.55	3.25	3.05	3.09
2.46	4.57	2.44	2.69	1.53	-0.52	0.24	-1.24	2.39	1.35	1.61	7.51
2.09	9.49	3.50	2.27	2.48	-0.20	-0.10	0.98	3.62	1.86	15.59	8.32
0.66	-0.45	0.58	2.50	1.30	-0.02	0.37	2.56	3.79	4.15	12.34	
0.79	8.43	0.92	1.32	2.70	0.60	-0.02	0.40	1.60	2.64	9.95	5.96
3.44	4.51	2.50	2.39	3.73	-0.31	-0.77	-0.85	2.11	3.99	5.08	5.90
1.38	7.34	2.49	2.51	3.92	-0.31	-0.22	-0.42	1.98	4.63	5.99	10.11
0.66	3.89	1.79	1.80	0.91	0.13	-0.15	0.45	2.54	2.50	3.07	6.28
0.63	5.48	1.83	1.14	4.43	0.39	-0.07	0.78	1.82	2.13	2.05	6.21
0.59	6.95	1.23	1.88	4.99	0.18	0.39	0.40	2.80	2.32	7.07	3.19
2.20	5.20	2.55	2.44	4.95	-0.67	-0.77	-0.80	3.18	1.60	4.36	8.49
0.41	0.55	2.28	2.05	2.19	-0.50	-0.68	-0.09	2.36	1.78	0.43	14.63
1.01	1.83	1.23	2.17	0.92	-0.37	-0.20	-0.30	2.45	1.50	3.83	5.09
0.88	8.54	2.22	0.11	2.05	-0.01	-0.31	0.66	2.21	2.25	7.39	1.84
1.31	10.71	-0.14	2.44	1.86	0.58	0.65	0.86	2.09	2.51	9.57	3.87
0.93	1.28	1.37	0.65	0.60	-0.91	-0.72	-0.69	2.79	1.63	0.78	4.27
2.85	7.39	1.65	1.50	2.01	-0.07	-0.32	0.00	1.47	2.10	6.77	9.70
4.14	13.00	0.95	1.03	2.92	-0.34	-0.27	-0.14	3.59	3.93	16.79	8.14
1.60	7.62	1.14	0.64	2.04	-0.17	-0.30	0.40	2.54	2.93	5.01	3.93
1.72	1.79	0.50	1.18	2.71	0.23	0.21	1.18	5.08	2.64	6.40	2.72
1.24	1.40	2.18	1.13	1.41	-0.52	-1.63	-0.80	0.95	3.39	1.82	3.82
2.93	5.23	0.95	1.62	1.83	-0.57	-0.58	0.17	2.60	3.26	3.16	10.94
2.75	3.94	1.14	1.87	2.01	0.09	-0.36	0.04	2.33	3.02	2.15	4.24
2.52	7.48	0.75	1.18	1.85	0.37	-0.48	-0.18	3.09	2.28	7.95	5.33
1.88	1.93	1.00	1.35	1.32	0.26	0.14	0.57	3.40	3.56	2.05	3.17
2.57	3.22	0.58	1.28	1.97	-1.00	-0.96	-0.81	2.85	2.29	3.95	6.57
2.24	5.86	3.11	1.90	3.20	-0.84	-0.22	-0.45	1.41	3.58	5.84	7.34
2.27	3.64	-0.40	2.12	2.28	-0.51	-0.13	0.98	2.08	3.80	10.42	3.98
1.42	5.30	1.52	0.39	0.60	0.42	-0.24	0.74	2.67	2.11	4.76	8.87
1.24	0.25	0.06	0.71	0.88	0.25	0.42	0.46	3.22	3.13	4.82	4.62

	14.288			22.322			0.6206			191.12	
	1.1919			3.103			0.0334			11.35	

x 48h (2nd repl.)		MEAN LipidTox NORM		
1uM	10uM	0.1uM	1uM	10uM
2.13	9.93	4.70	1.68	5.80
14.48	19.07	3.31	9.02	11.41
2.54	20.26	5.28	2.03	12.24
7.33	23.37	4.17	5.62	13.89
4.78	24.03	2.82	4.01	13.54
6.54	58.85	4.95	3.94	30.23
14.54	89.24	5.97	8.20	52.42
		3.79	4.15	12.34
12.46	64.38	3.78	7.55	37.17
8.72	52.19	4.00	6.36	28.63
16.24	63.36	6.05	10.44	34.68
8.51	28.76	4.41	5.50	15.91
3.77	21.85	4.02	2.95	11.95
15.73	53.67	2.99	9.02	30.37
9.58	33.00	5.84	5.59	18.68
13.04	8.74	8.50	7.41	4.59
8.32	13.64	3.77	4.91	8.73
9.40	61.48	2.02	5.82	34.44
6.00	76.96	2.98	4.25	43.26
4.61	8.80	3.53	3.12	4.79
12.64	55.30	5.59	7.37	31.04
24.76	100.78	5.87	14.34	58.79
3.89	46.45	3.23	3.41	25.73
1.65	7.99	3.90	2.15	7.20
2.49	42.79	2.38	2.94	22.30
15.27	48.98	6.77	9.27	26.07
11.38	28.07	3.29	7.20	15.11
5.93	57.88	4.21	4.10	32.91
9.31	11.11	3.29	6.43	6.58
6.55	16.21	4.71	4.42	10.08
12.59	49.03	4.37	8.08	27.44
11.23	30.06	3.03	7.51	20.24
8.44	37.72	5.77	5.28	21.24
6.21	4.14	3.92	4.67	4.48

	226.95
	5.5086
