# Supplementary material for: Comparing structural and transcriptional drug networks reveals signatures of drug activity and toxicity in transcriptional responses
Source: NPJ Syst Biol Appl. 2017 Aug 25;3:23. doi: 10.1038/s41540-017-0022-3 (PMC5572457; doi:10.1038/s41540-017-0022-3)
Supplement: Supplementary file 11 — Supplementary Table 9 [file 41540_2017_22_MOESM11_ESM.pdf]

rank	distance	Name	LIPIDTOX (48h)			logP	pKa
			10uM	1uM	0.1uM		
1	0.309	terfenadine	8.76	10.84	1.30	6.48	9.02
2	0.388	metergoline	3.71	1.64	0.77	3.99	11.17
5	0.408	protriptyline	16.81	1.31	0.93	4.5	10.54
7	0.415	mefloquine	22.23	4.20	0.99	4.11	9.46
8	0.422	niclosamide	-3.26	-2.18	1.89	3.91	-4.43
10	0.434	norcyclobenzaprine	18.05	1.72	0.45	4.23	10.47
11	0.454	cloperastine	1.02	-0.05	0.37	5.11	12.9
12	0.477	alexidine	18.62	4.92	1.02	5.53	9.59
15	0.489	piperacetazine	9.79	1.22	0.24	3.63	8.21
17	0.491	metixene	26.15	2.91	1.95	5.06	9.34
18	0.491	gossypol	-3.53	0.98	1.66	8.02	-6.15
23	0.507	benzethonium_chloride	7.28	3.37	1.30	2.88	-4.97
25	0.511	hexetidine	1.01	1.13	0.58	5.74	9.12
27	0.517	tonzonium_bromide	6.74	2.60	0.94	4.92	-3.08
30	0.526	pyrvinium	-3.20	-2.91	0.51	1.41	1.48
34	0.535	tyrphostin_AG-1478	1.55	-0.56	1.70	3.75	6.29
37	0.541	phenazopyridine	0.99	1.22	0.78	2.69	6.86
41	0.559	azacyclonol	2.69	0.49	1.06	2.88	-3.58
44	0.564	econazole	2.13	0.71	0.41	5.35	6.77
47	0.577	monensin	2.33	3.62	4.20	6.16	-2.93
50	0.585	benzamil	-0.82	0.18	0.76	1.11	5.47
59	0.603	thioproperazine	25.33	0.60	0.25	2.83	8.36

CAD
YES
YES
YES
YES
NO
YES
YES
YES
YES
YES
NO
NO
YES
NO
NO
NO
NO
NO
NO
NO
NO
NO
